# Supplementary figures and images for: Axon-dependent expression of YAP/TAZ mediates Schwann cell remyelination but not proliferation after nerve injury (part 1 of 4)
Source: eLife. 2020 May 21;9:e50138. doi: 10.7554/eLife.50138 (PMC7259960; doi:10.7554/eLife.50138)

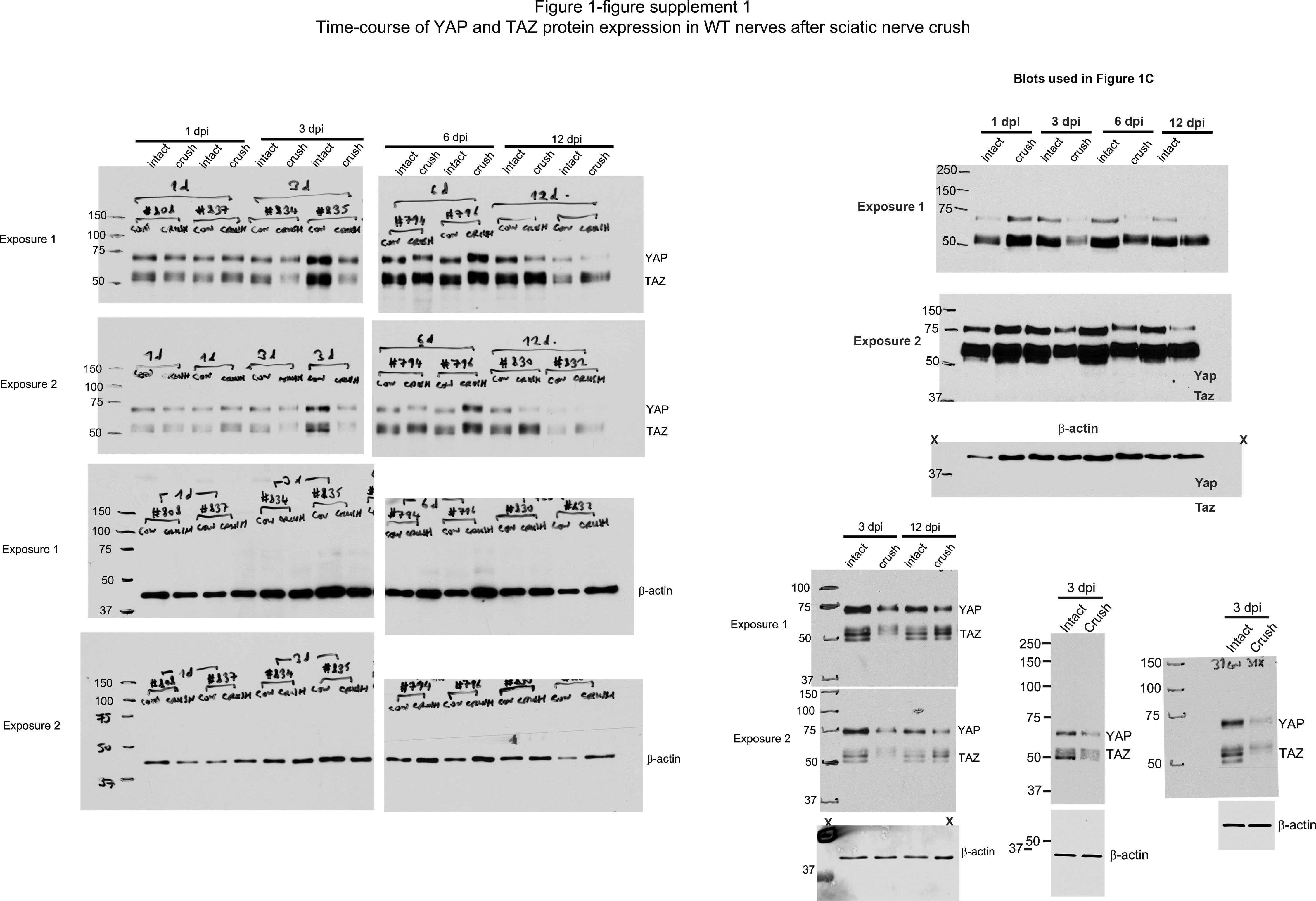

Supplement: Figure 1—source data 2. — Uncropped Western blots of images used to make Figure 1C. Individually processed samples from 6 mice at three dpi, 5 mice at 12 dpi, and three mice at both 1 dpi and six dpi are shown and used for quantification. X…….X denotes the line along which membranes were cut prior to probing with anti-beta actin antibody. Two exposures of anti-YAP/TAZ blot are shown. [file elife-50138-fig1-data2.jpg]

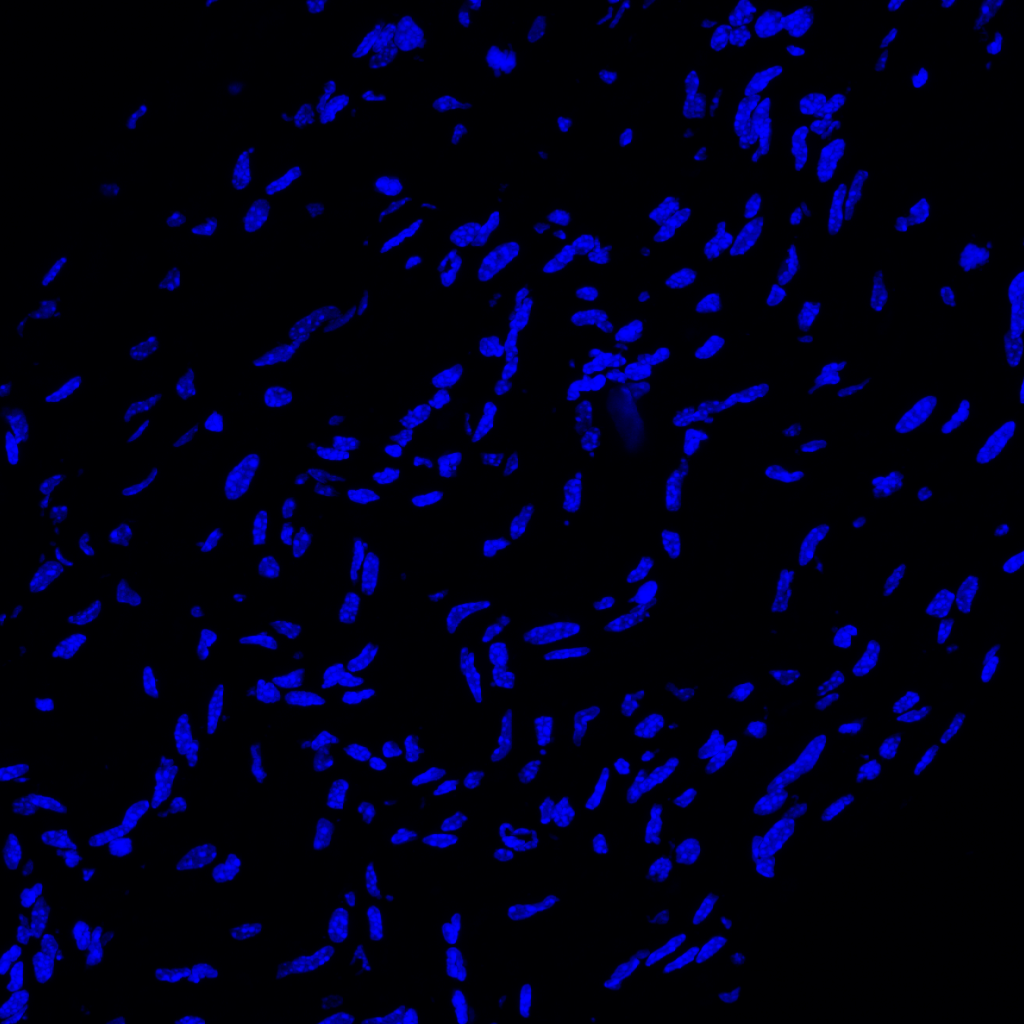

Supplement: Figure 3—source data 1. — This zip archive contains the IHC images for one WT and one iDKO used for the quantitative analysis shown in Figure 3G. Leica SP8 confocal lif images were processed using Imaris software and saved as tiffs. [file elife-50138-fig3-data1.zip › Figure 3 source data 1/iDKO #916 EdU/RHS a DAPI.tif]

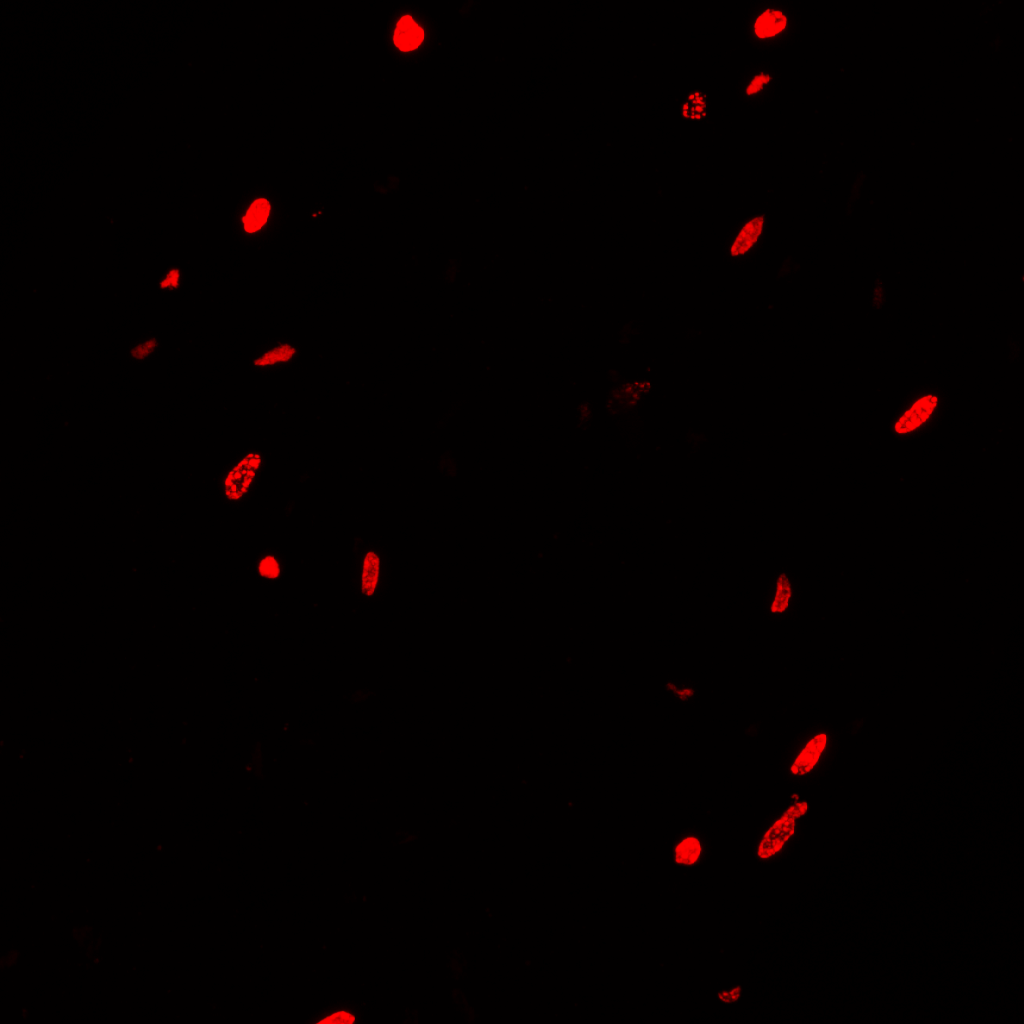

Supplement: Figure 3—source data 1. — This zip archive contains the IHC images for one WT and one iDKO used for the quantitative analysis shown in Figure 3G. Leica SP8 confocal lif images were processed using Imaris software and saved as tiffs. [file elife-50138-fig3-data1.zip › Figure 3 source data 1/iDKO #916 EdU/RHS a EdU.tif]

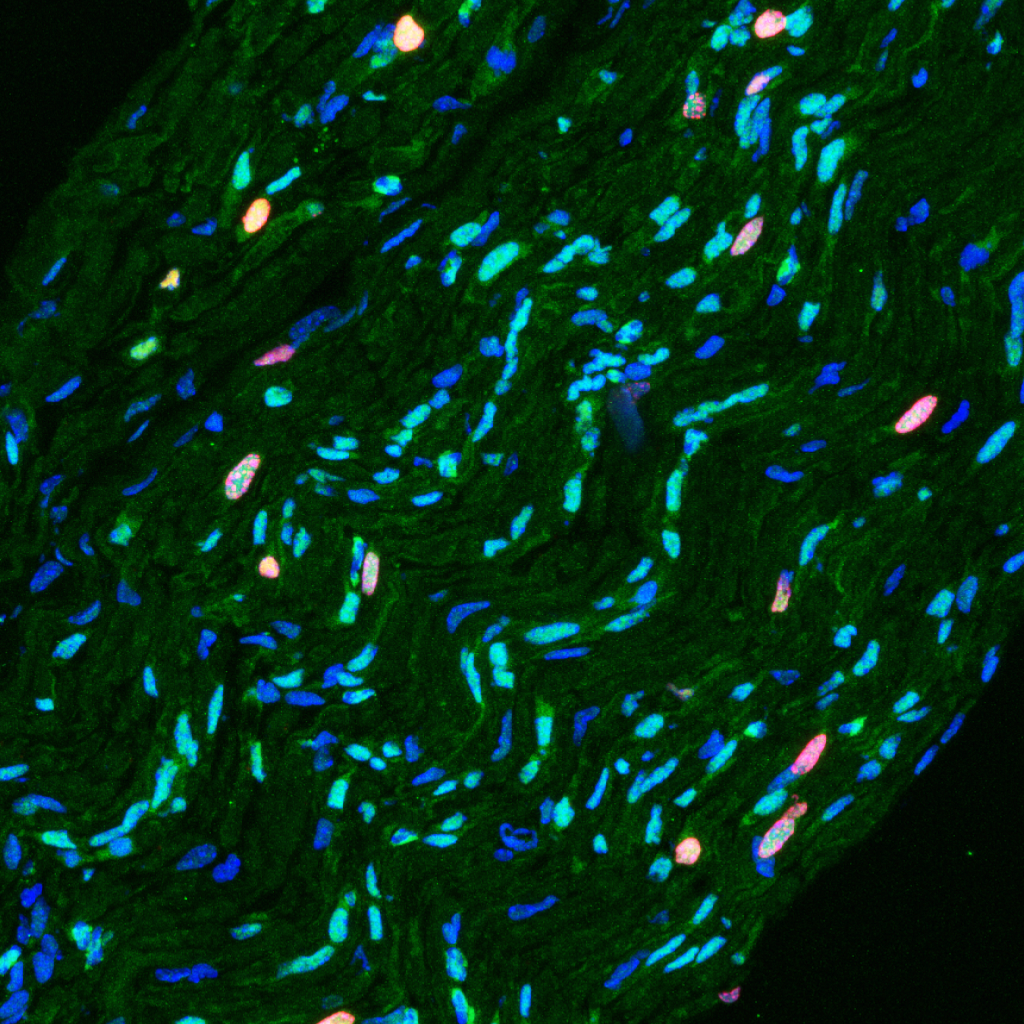

Supplement: Figure 3—source data 1. — This zip archive contains the IHC images for one WT and one iDKO used for the quantitative analysis shown in Figure 3G. Leica SP8 confocal lif images were processed using Imaris software and saved as tiffs. [file elife-50138-fig3-data1.zip › Figure 3 source data 1/iDKO #916 EdU/RHS a merge.tif]

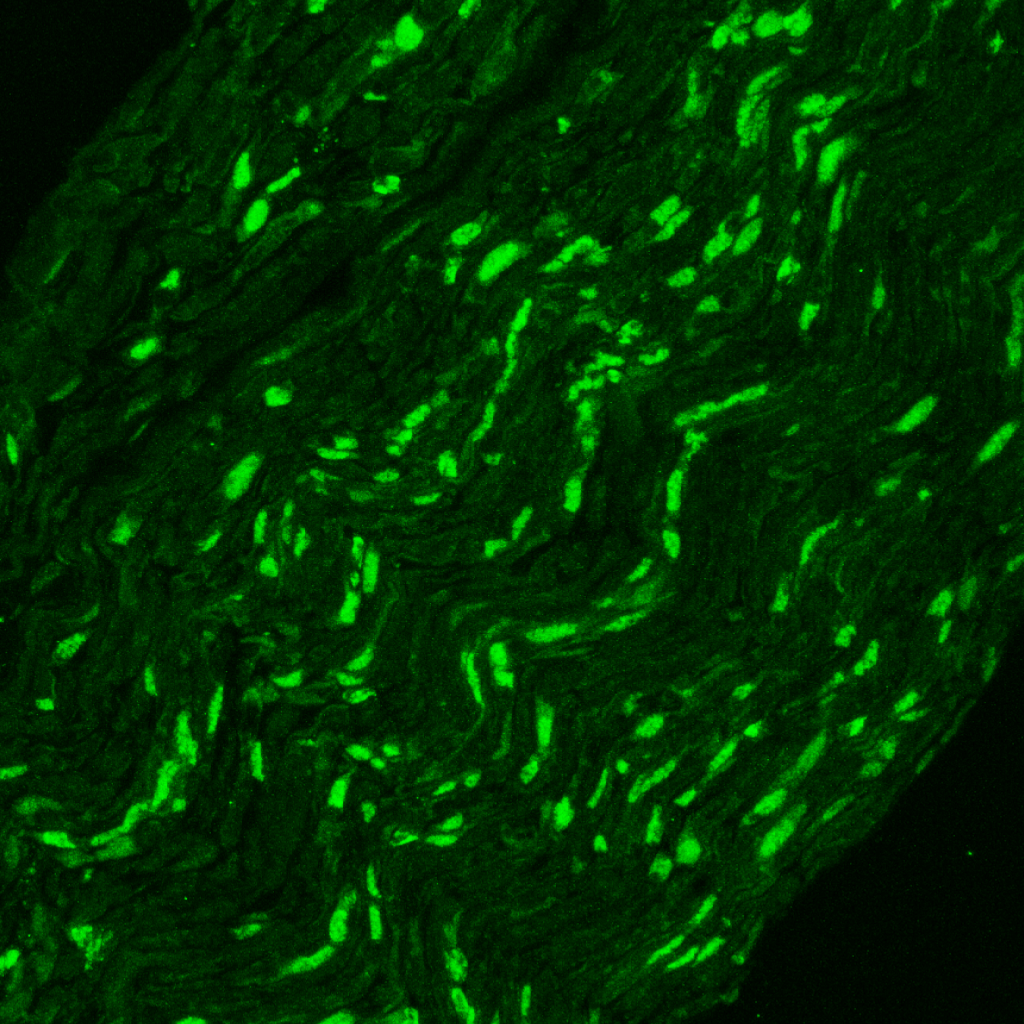

Supplement: Figure 3—source data 1. — This zip archive contains the IHC images for one WT and one iDKO used for the quantitative analysis shown in Figure 3G. Leica SP8 confocal lif images were processed using Imaris software and saved as tiffs. [file elife-50138-fig3-data1.zip › Figure 3 source data 1/iDKO #916 EdU/RHS a Sox10.tif]

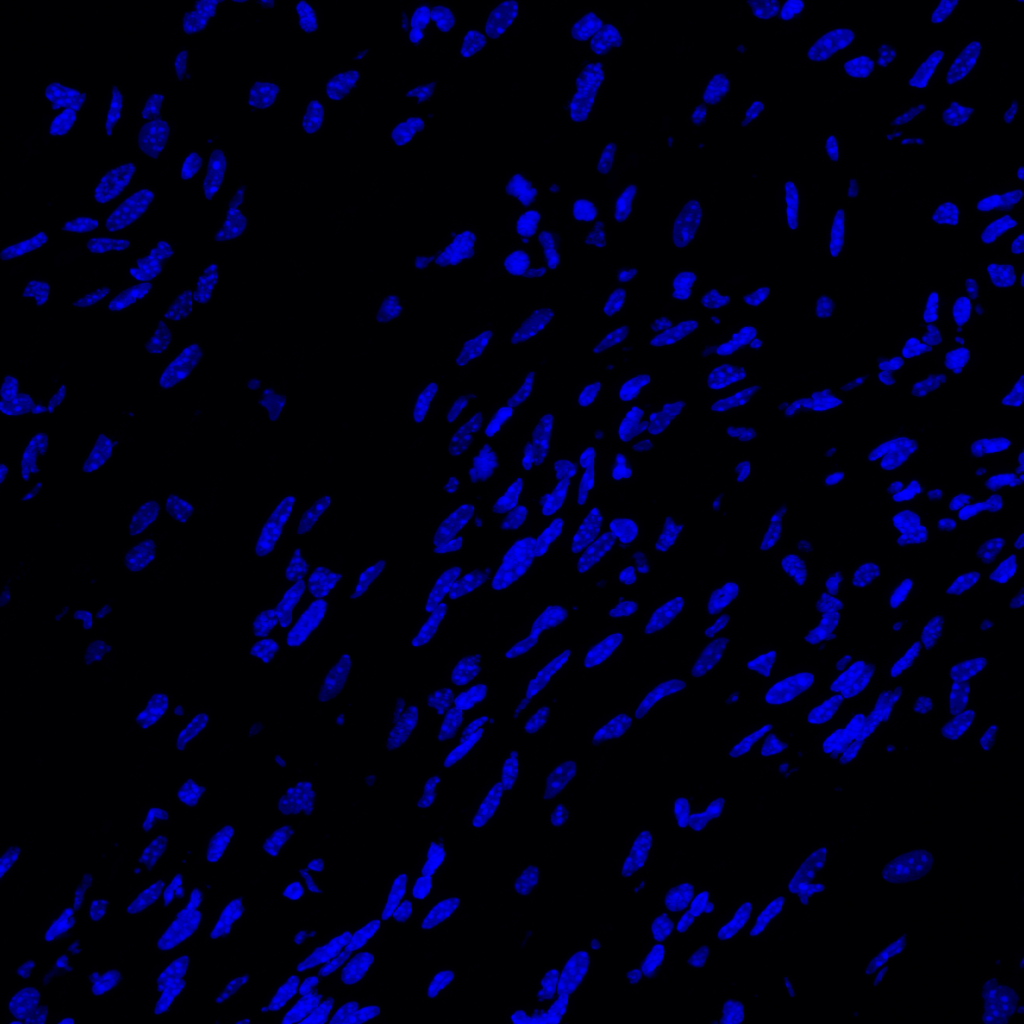

Supplement: Figure 3—source data 1. — This zip archive contains the IHC images for one WT and one iDKO used for the quantitative analysis shown in Figure 3G. Leica SP8 confocal lif images were processed using Imaris software and saved as tiffs. [file elife-50138-fig3-data1.zip › Figure 3 source data 1/iDKO #916 EdU/RHS b DAPI.tif]

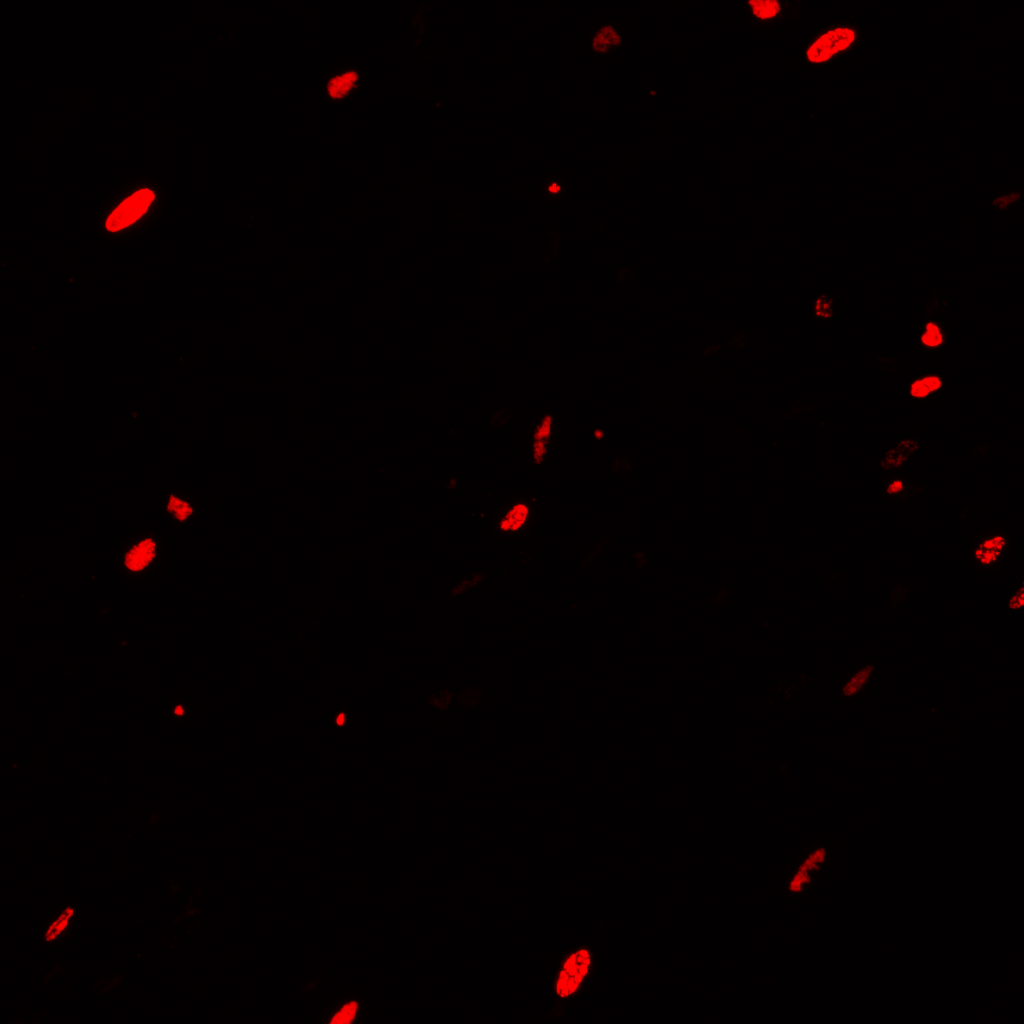

Supplement: Figure 3—source data 1. — This zip archive contains the IHC images for one WT and one iDKO used for the quantitative analysis shown in Figure 3G. Leica SP8 confocal lif images were processed using Imaris software and saved as tiffs. [file elife-50138-fig3-data1.zip › Figure 3 source data 1/iDKO #916 EdU/RHS b EdU.tif]

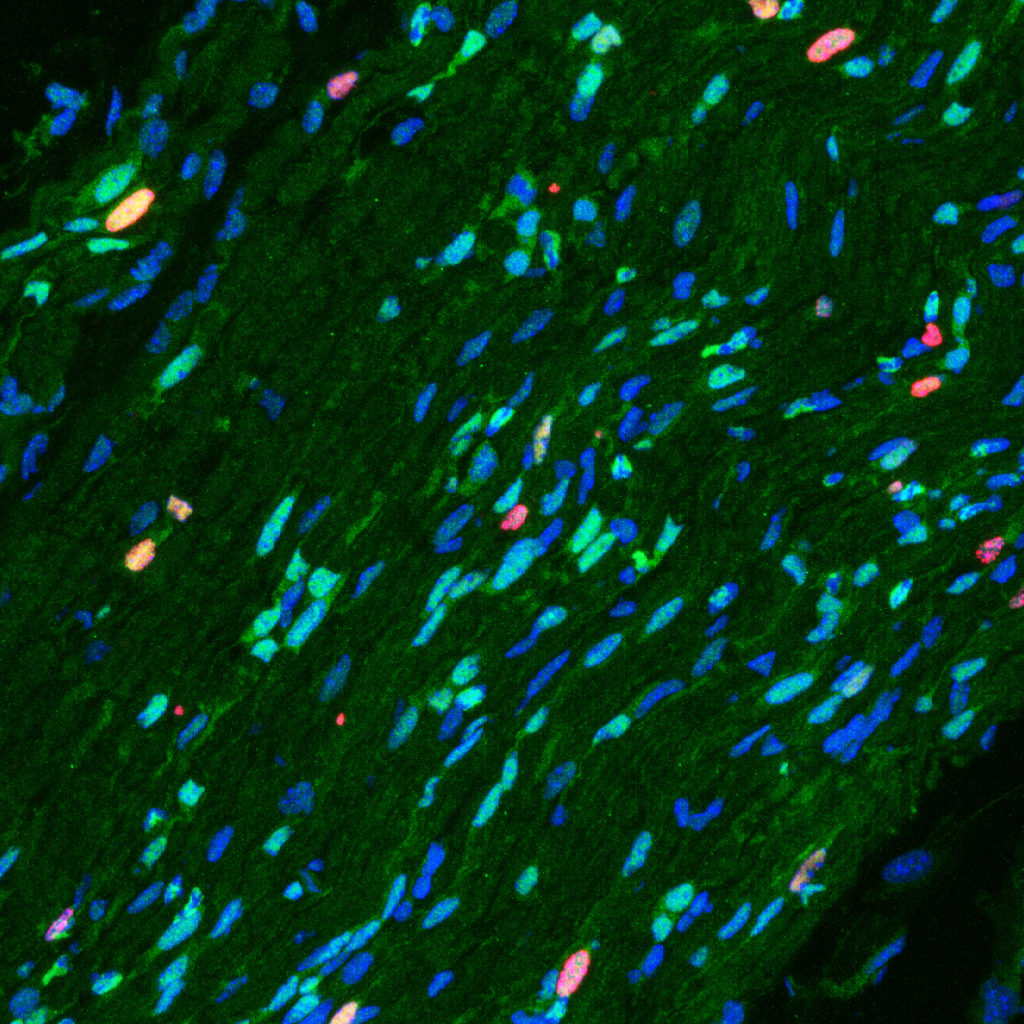

Supplement: Figure 3—source data 1. — This zip archive contains the IHC images for one WT and one iDKO used for the quantitative analysis shown in Figure 3G. Leica SP8 confocal lif images were processed using Imaris software and saved as tiffs. [file elife-50138-fig3-data1.zip › Figure 3 source data 1/iDKO #916 EdU/RHS b merge.tif]

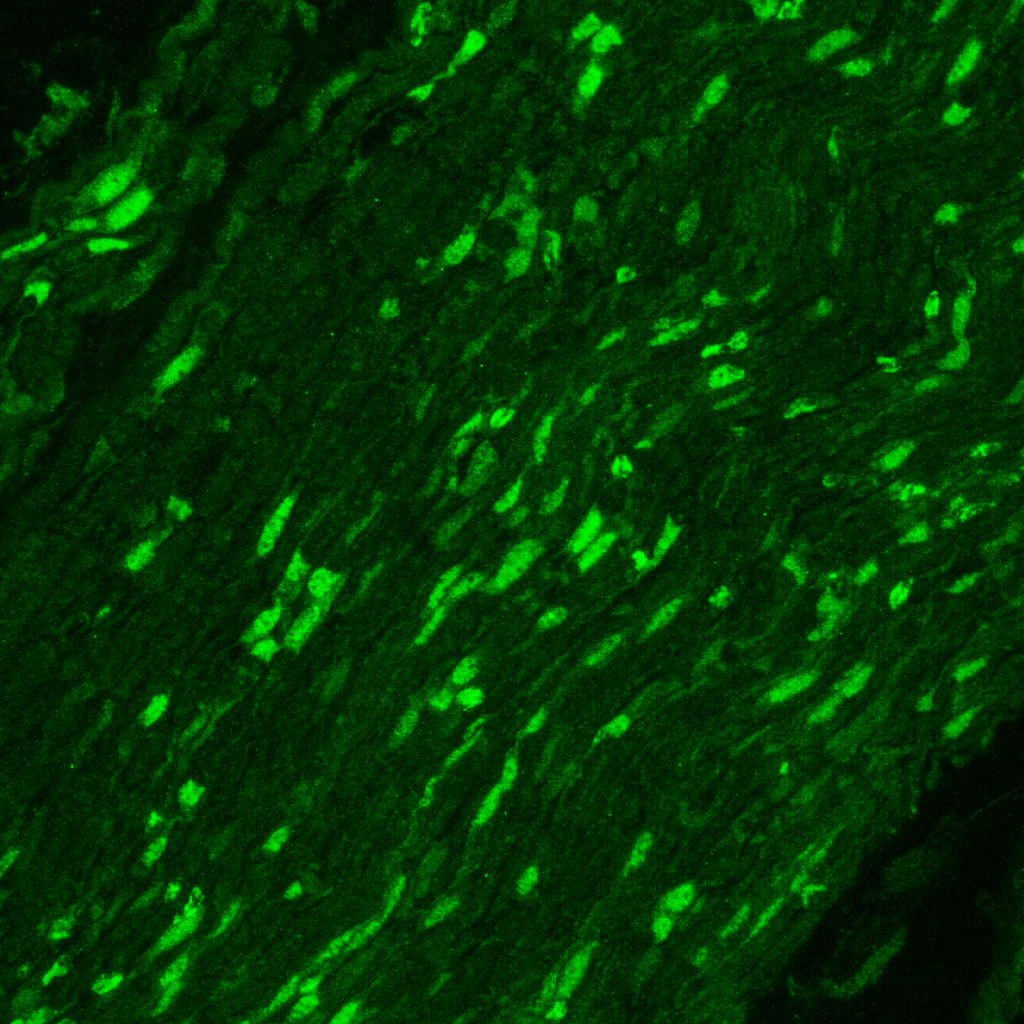

Supplement: Figure 3—source data 1. — This zip archive contains the IHC images for one WT and one iDKO used for the quantitative analysis shown in Figure 3G. Leica SP8 confocal lif images were processed using Imaris software and saved as tiffs. [file elife-50138-fig3-data1.zip › Figure 3 source data 1/iDKO #916 EdU/RHS b Sox10.tif]

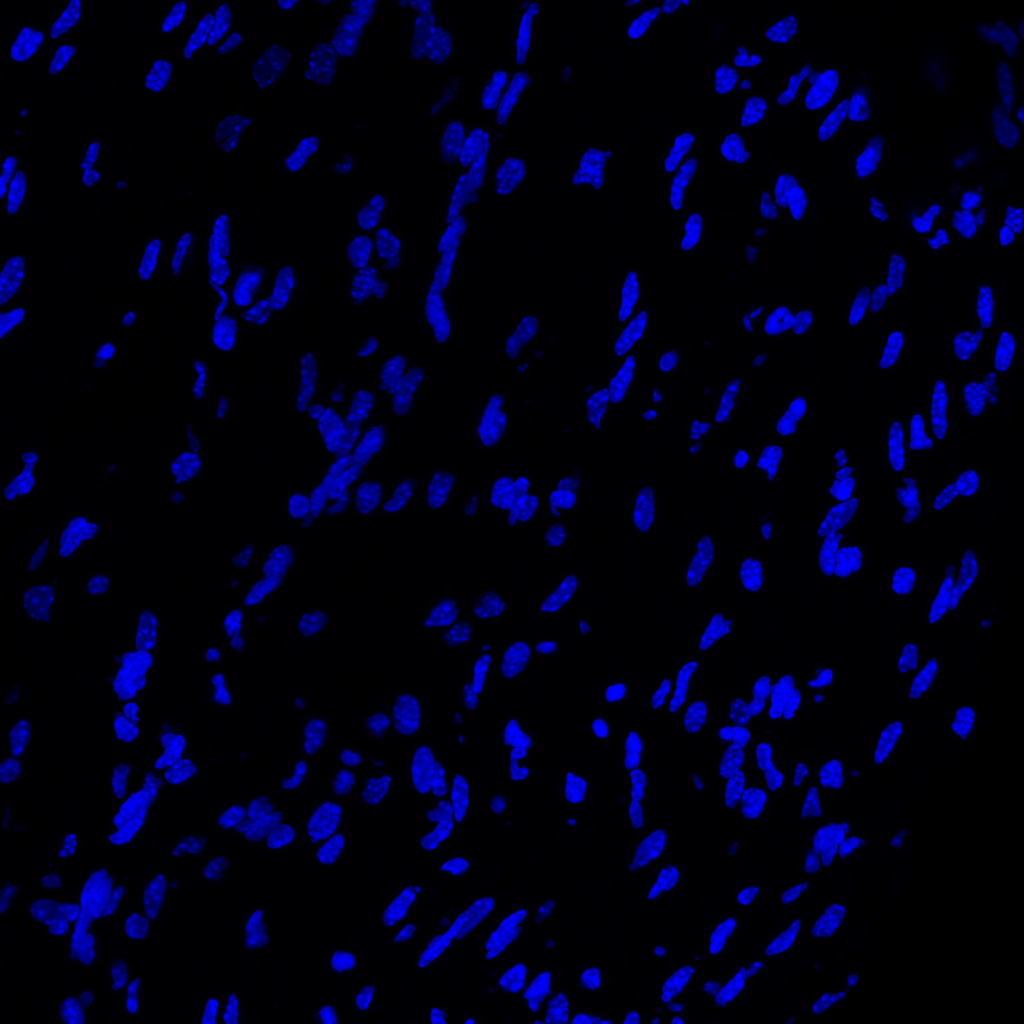

Supplement: Figure 3—source data 1. — This zip archive contains the IHC images for one WT and one iDKO used for the quantitative analysis shown in Figure 3G. Leica SP8 confocal lif images were processed using Imaris software and saved as tiffs. [file elife-50138-fig3-data1.zip › Figure 3 source data 1/iDKO #916 EdU/RHS c DAPI.tif]

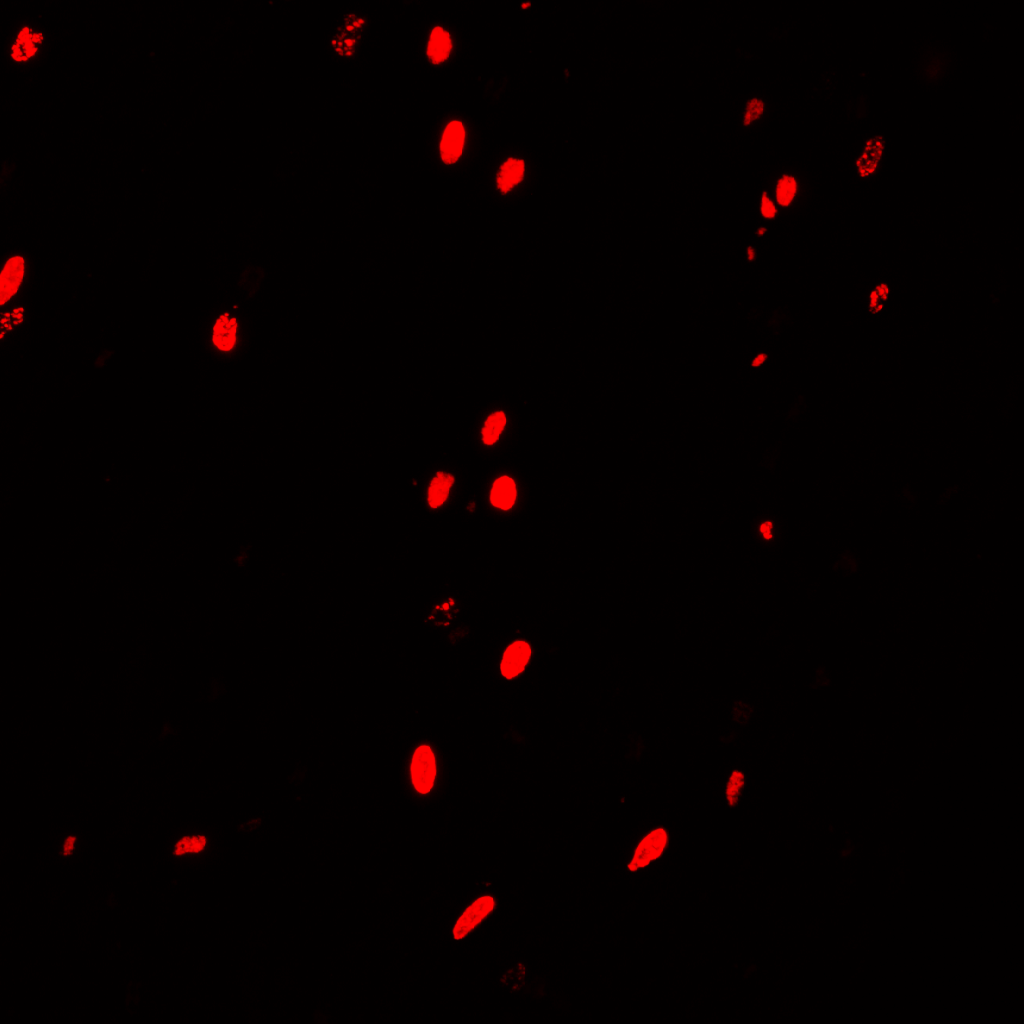

Supplement: Figure 3—source data 1. — This zip archive contains the IHC images for one WT and one iDKO used for the quantitative analysis shown in Figure 3G. Leica SP8 confocal lif images were processed using Imaris software and saved as tiffs. [file elife-50138-fig3-data1.zip › Figure 3 source data 1/iDKO #916 EdU/RHS c EdU.tif]

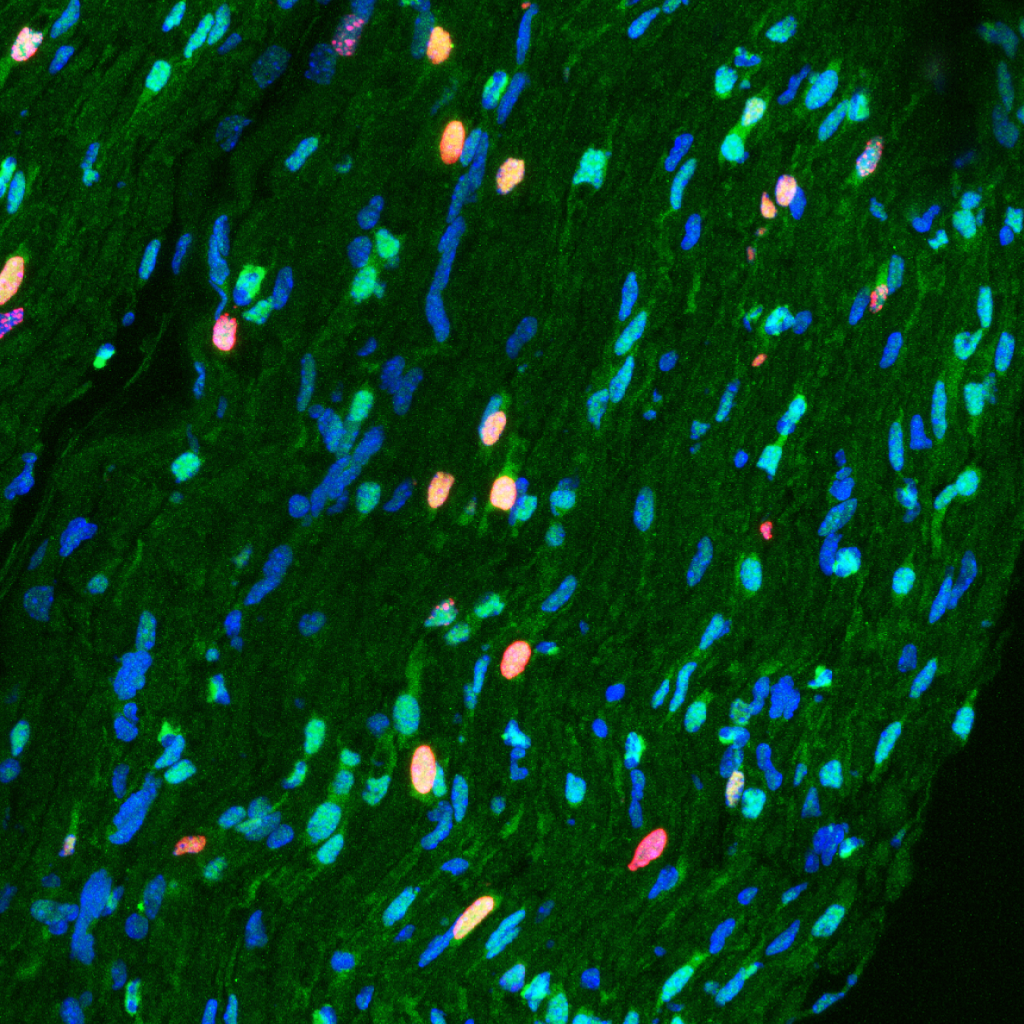

Supplement: Figure 3—source data 1. — This zip archive contains the IHC images for one WT and one iDKO used for the quantitative analysis shown in Figure 3G. Leica SP8 confocal lif images were processed using Imaris software and saved as tiffs. [file elife-50138-fig3-data1.zip › Figure 3 source data 1/iDKO #916 EdU/RHS c merge.tif]

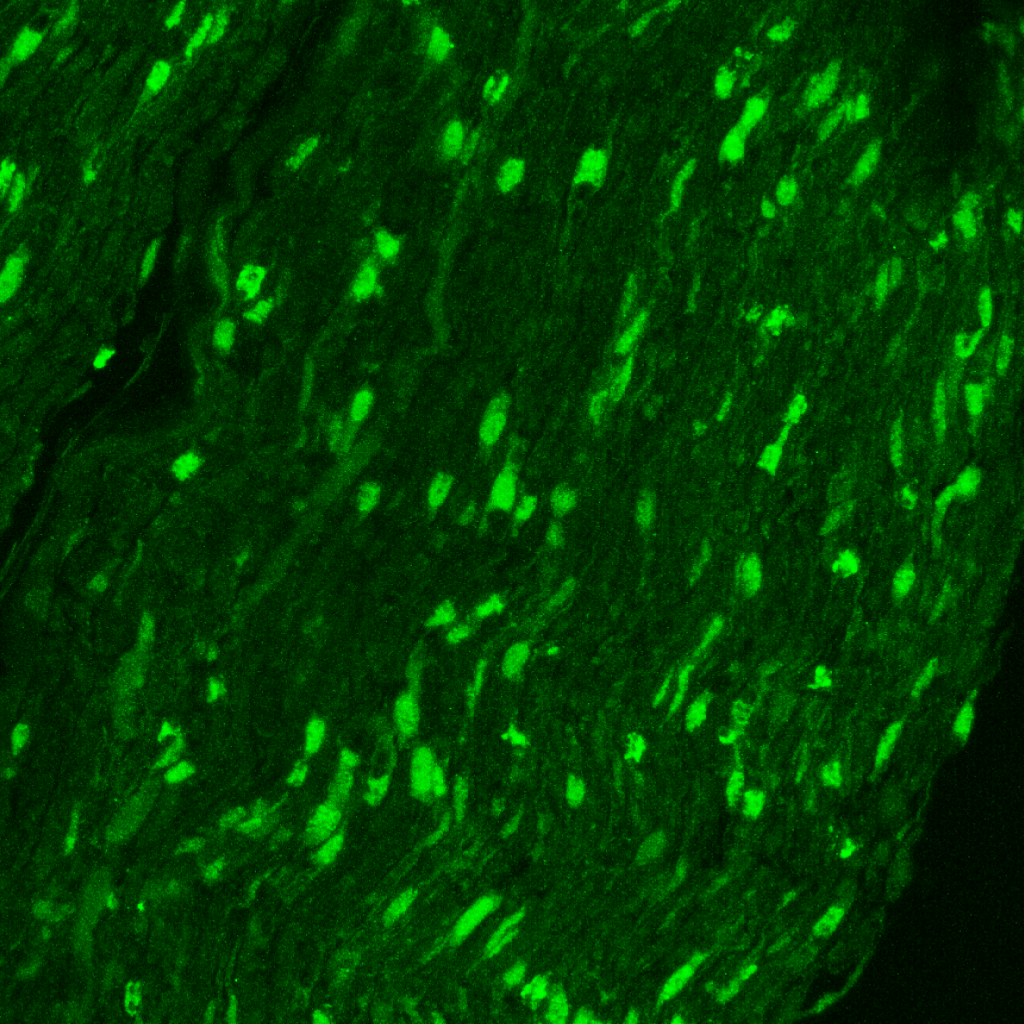

Supplement: Figure 3—source data 1. — This zip archive contains the IHC images for one WT and one iDKO used for the quantitative analysis shown in Figure 3G. Leica SP8 confocal lif images were processed using Imaris software and saved as tiffs. [file elife-50138-fig3-data1.zip › Figure 3 source data 1/iDKO #916 EdU/RHS c Sox10.tif]

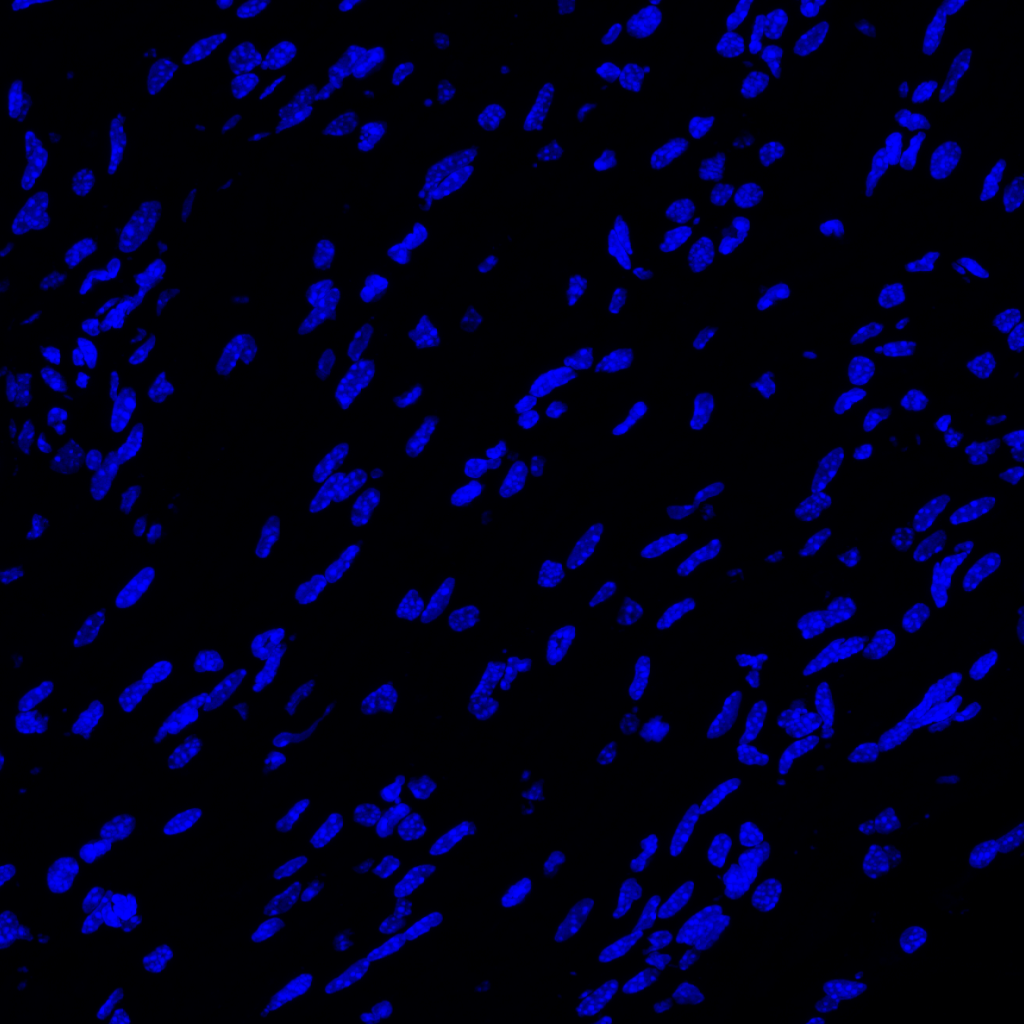

Supplement: Figure 3—source data 1. — This zip archive contains the IHC images for one WT and one iDKO used for the quantitative analysis shown in Figure 3G. Leica SP8 confocal lif images were processed using Imaris software and saved as tiffs. [file elife-50138-fig3-data1.zip › Figure 3 source data 1/iDKO #916 EdU/RHS d DAPI.tif]

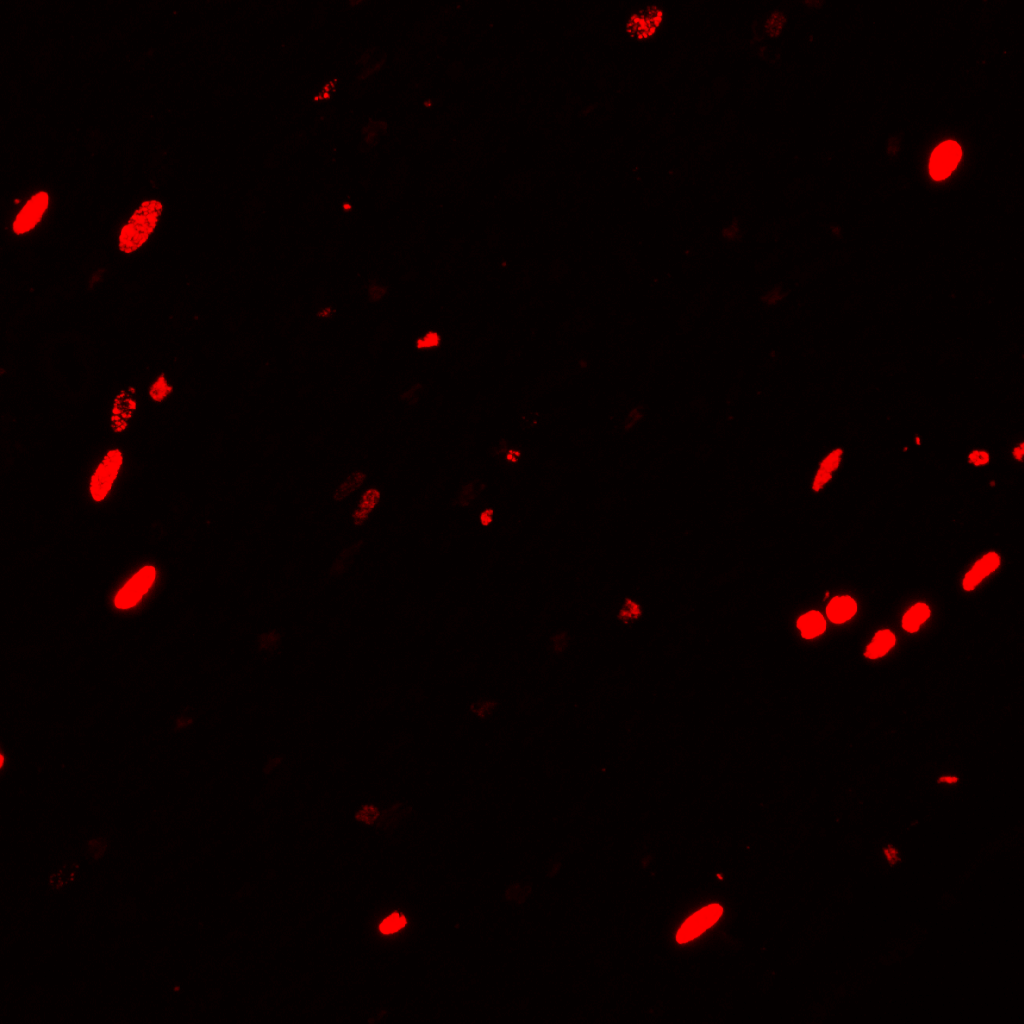

Supplement: Figure 3—source data 1. — This zip archive contains the IHC images for one WT and one iDKO used for the quantitative analysis shown in Figure 3G. Leica SP8 confocal lif images were processed using Imaris software and saved as tiffs. [file elife-50138-fig3-data1.zip › Figure 3 source data 1/iDKO #916 EdU/RHS d EdU.tif]

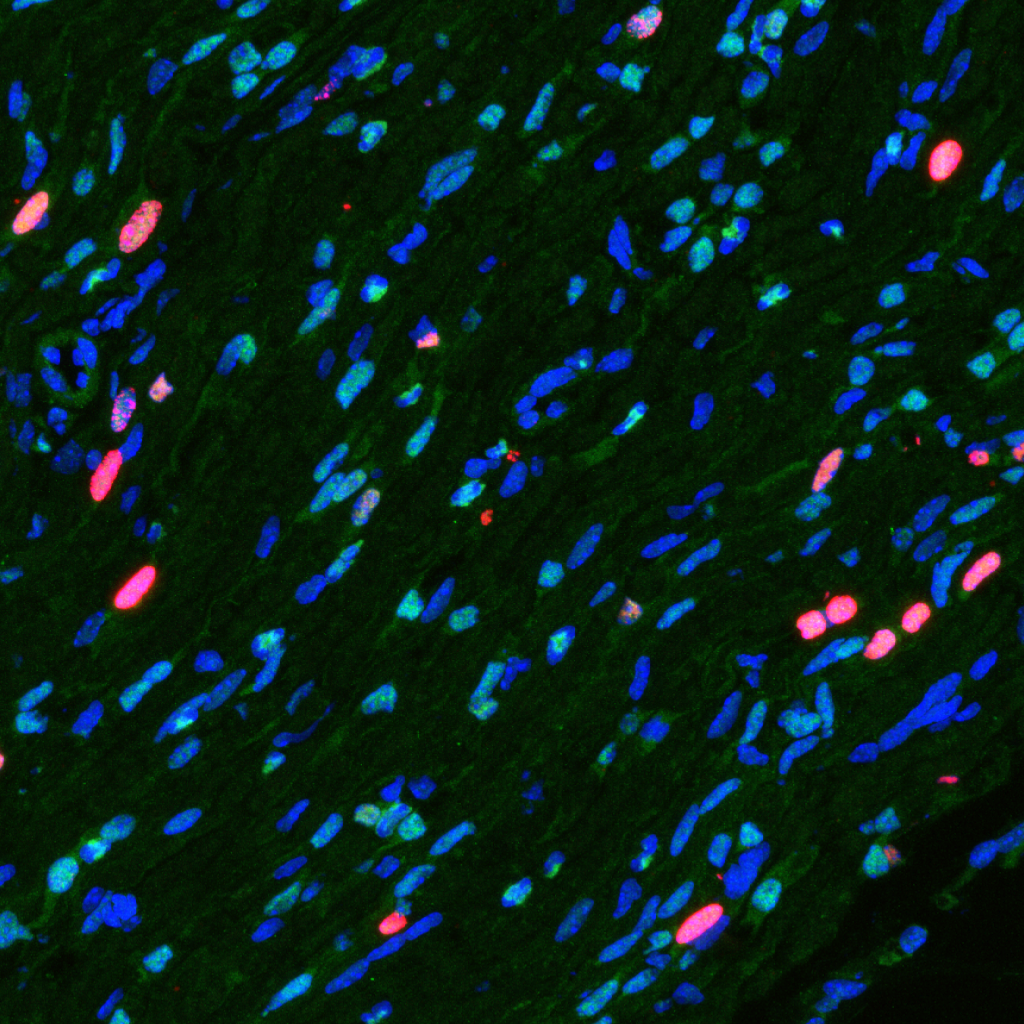

Supplement: Figure 3—source data 1. — This zip archive contains the IHC images for one WT and one iDKO used for the quantitative analysis shown in Figure 3G. Leica SP8 confocal lif images were processed using Imaris software and saved as tiffs. [file elife-50138-fig3-data1.zip › Figure 3 source data 1/iDKO #916 EdU/RHS d merge.tif]

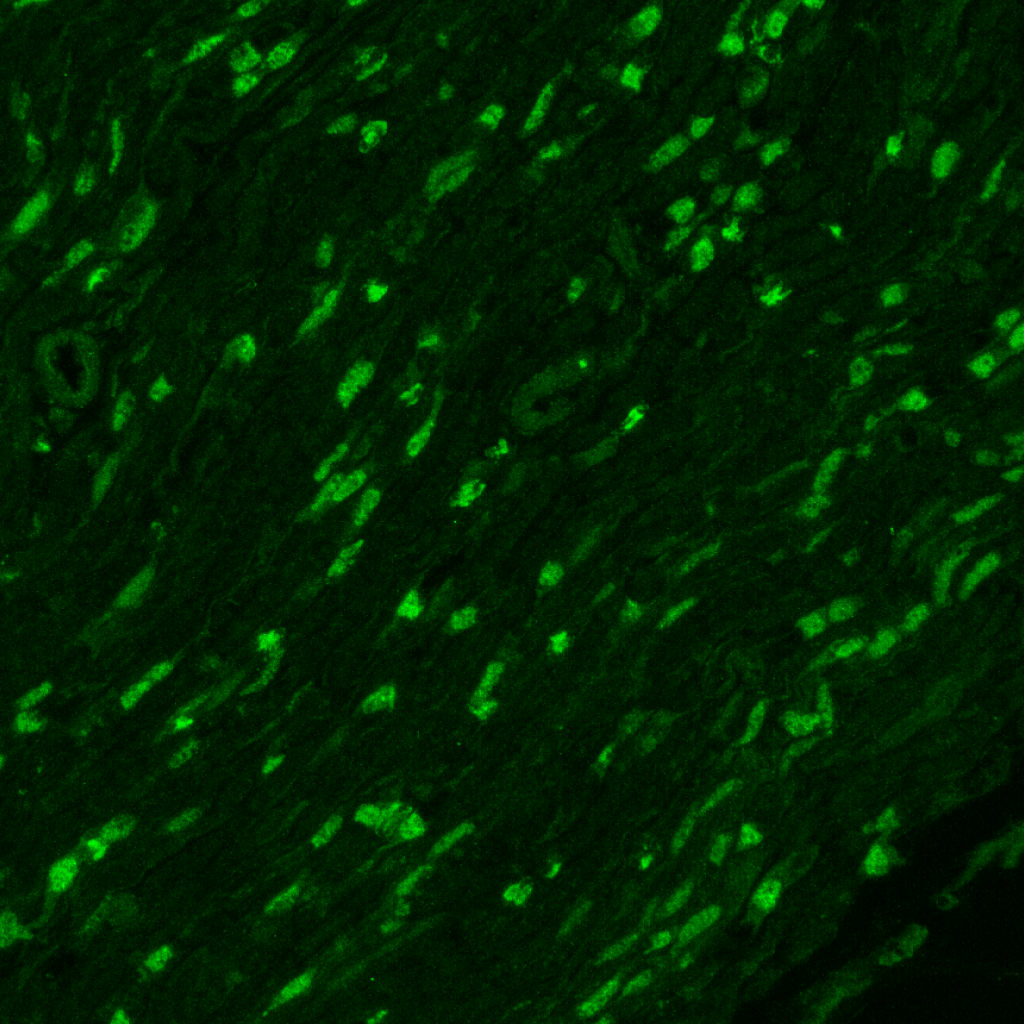

Supplement: Figure 3—source data 1. — This zip archive contains the IHC images for one WT and one iDKO used for the quantitative analysis shown in Figure 3G. Leica SP8 confocal lif images were processed using Imaris software and saved as tiffs. [file elife-50138-fig3-data1.zip › Figure 3 source data 1/iDKO #916 EdU/RHS d Sox10.tif]

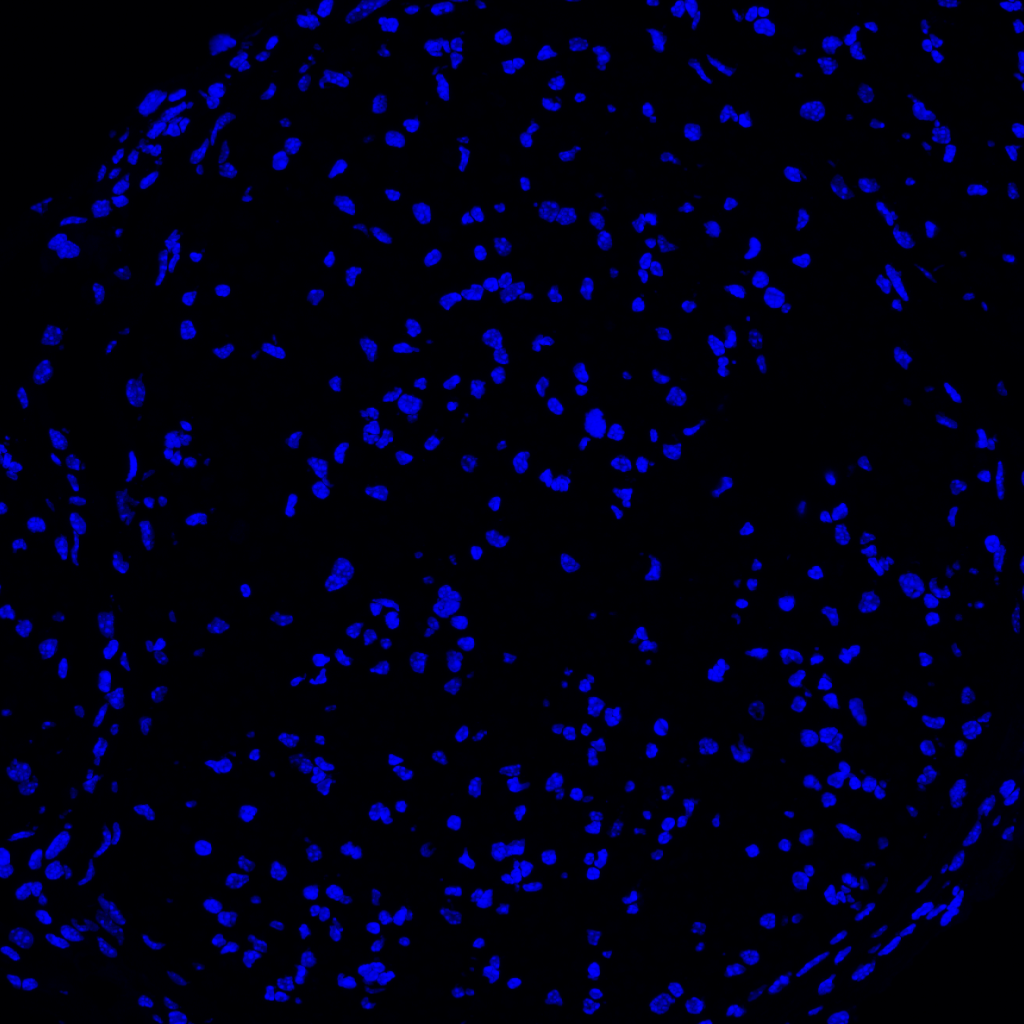

Supplement: Figure 3—source data 1. — This zip archive contains the IHC images for one WT and one iDKO used for the quantitative analysis shown in Figure 3G. Leica SP8 confocal lif images were processed using Imaris software and saved as tiffs. [file elife-50138-fig3-data1.zip › Figure 3 source data 1/iDKO #916 EdU/RHS xs a DAPI.tif]

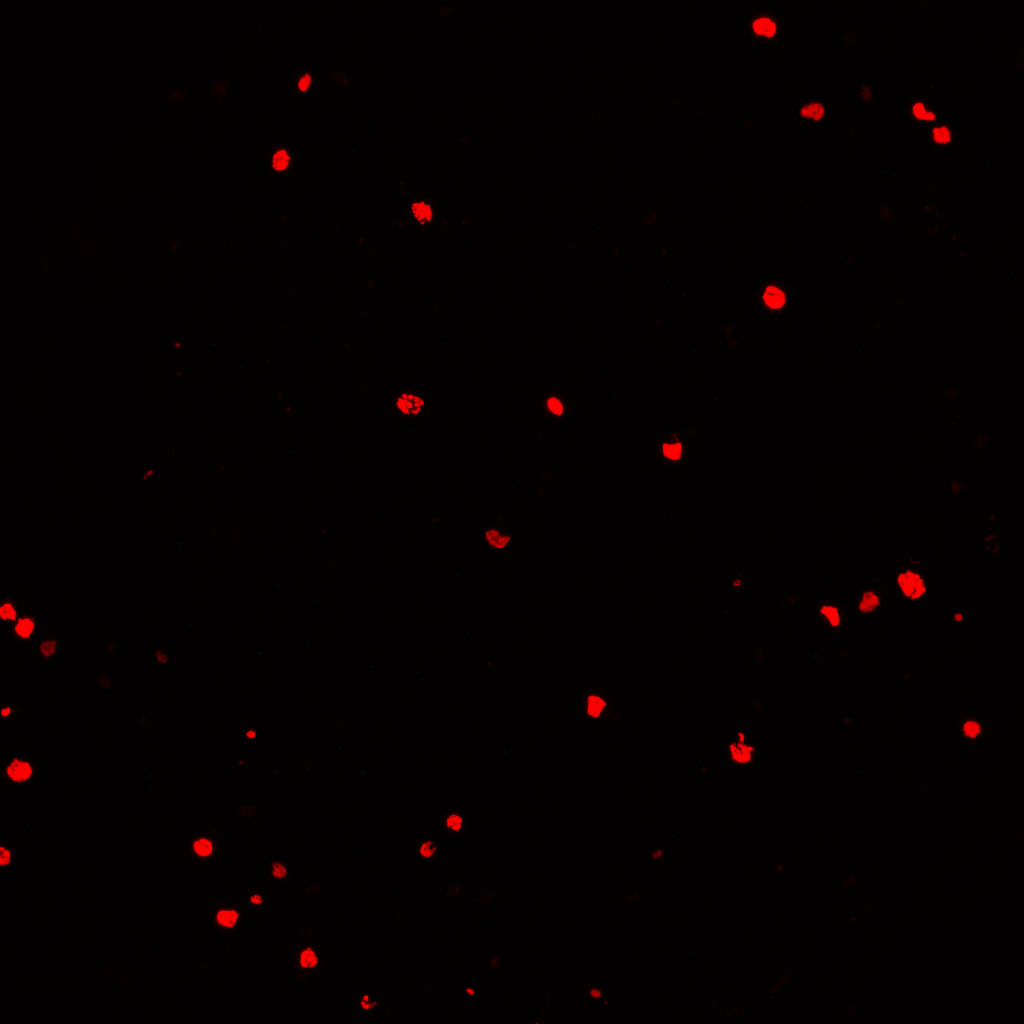

Supplement: Figure 3—source data 1. — This zip archive contains the IHC images for one WT and one iDKO used for the quantitative analysis shown in Figure 3G. Leica SP8 confocal lif images were processed using Imaris software and saved as tiffs. [file elife-50138-fig3-data1.zip › Figure 3 source data 1/iDKO #916 EdU/RHS xs a EdU.tif]

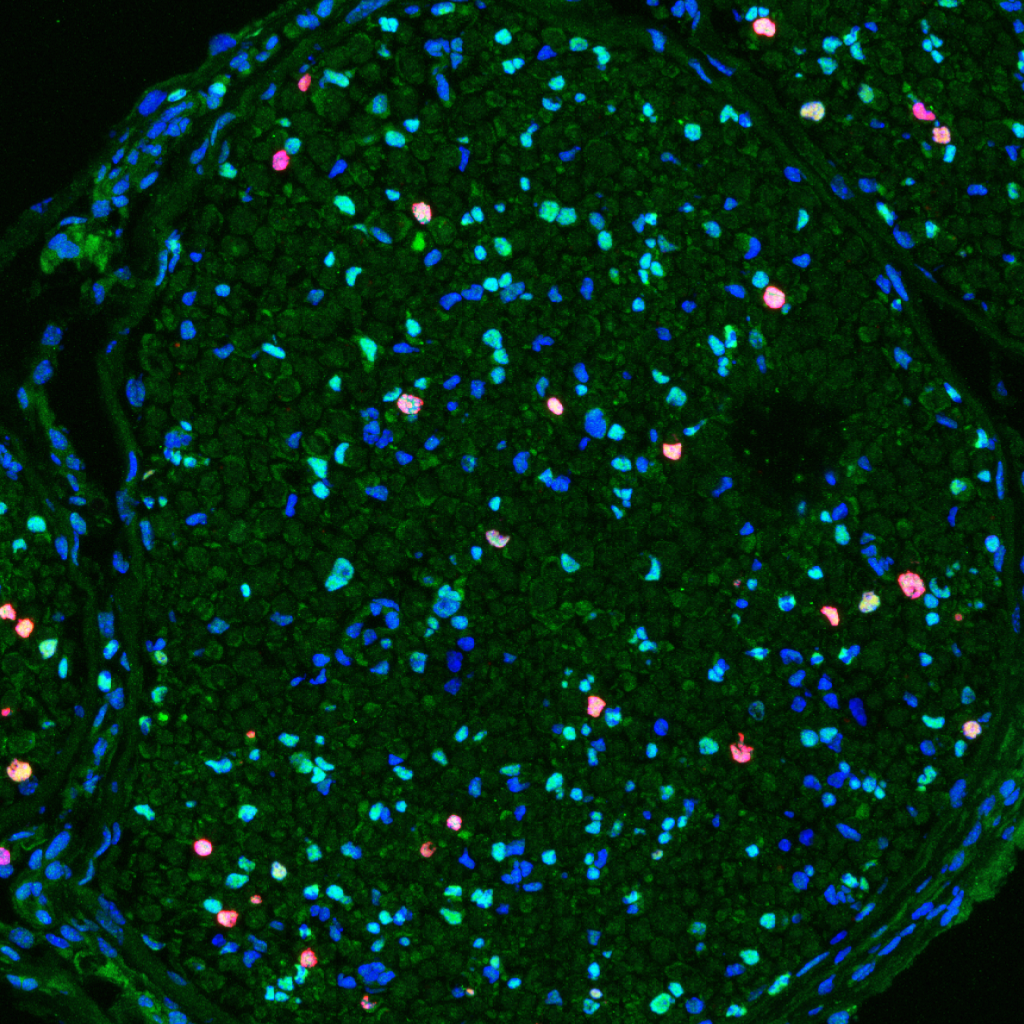

Supplement: Figure 3—source data 1. — This zip archive contains the IHC images for one WT and one iDKO used for the quantitative analysis shown in Figure 3G. Leica SP8 confocal lif images were processed using Imaris software and saved as tiffs. [file elife-50138-fig3-data1.zip › Figure 3 source data 1/iDKO #916 EdU/RHS xs a merge.tif]

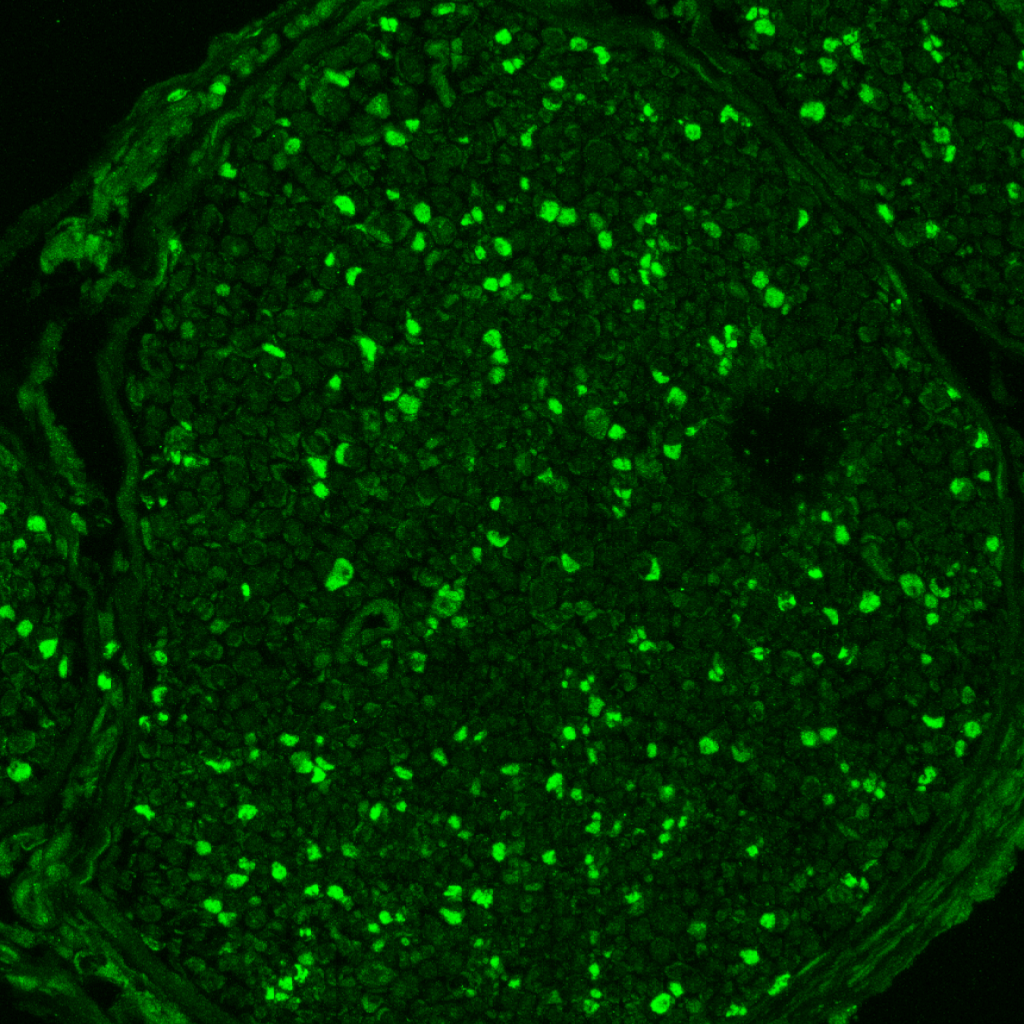

Supplement: Figure 3—source data 1. — This zip archive contains the IHC images for one WT and one iDKO used for the quantitative analysis shown in Figure 3G. Leica SP8 confocal lif images were processed using Imaris software and saved as tiffs. [file elife-50138-fig3-data1.zip › Figure 3 source data 1/iDKO #916 EdU/RHS xs a Sox10.tif]

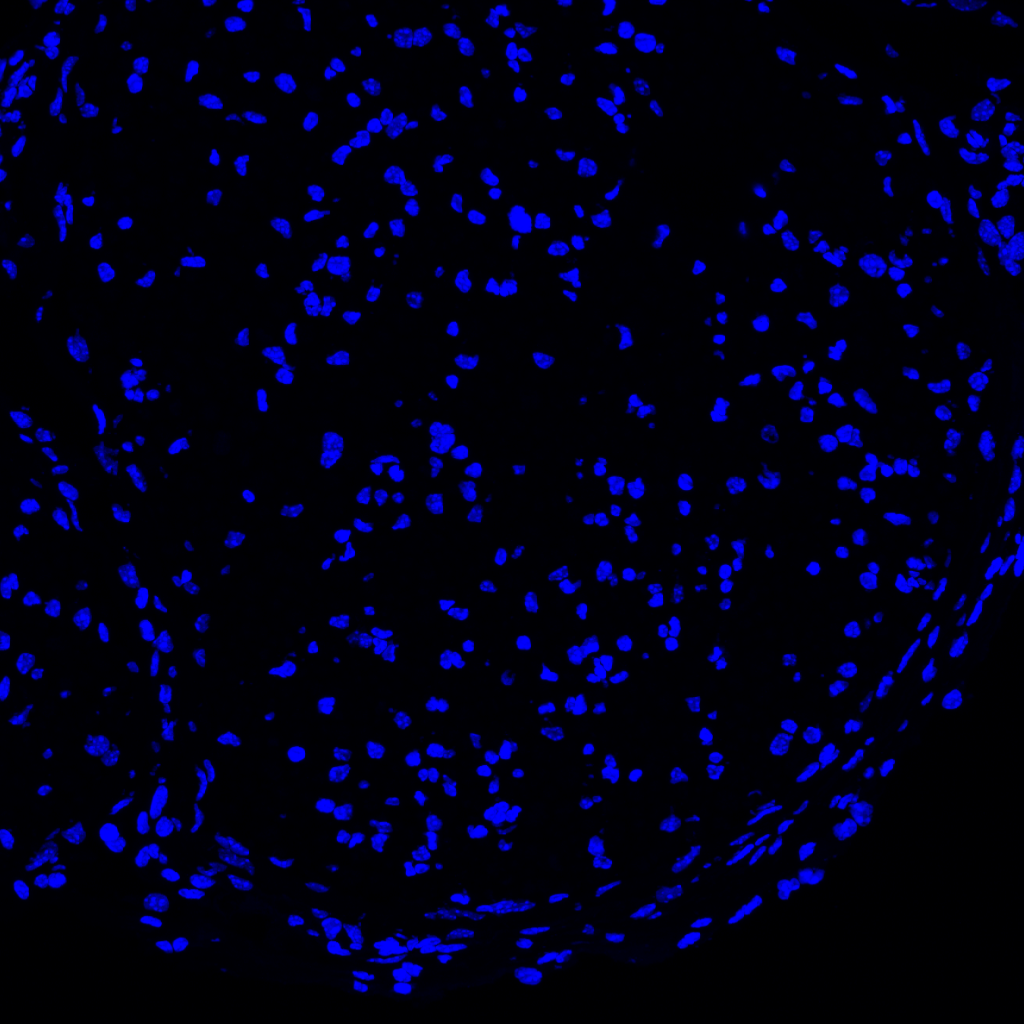

Supplement: Figure 3—source data 1. — This zip archive contains the IHC images for one WT and one iDKO used for the quantitative analysis shown in Figure 3G. Leica SP8 confocal lif images were processed using Imaris software and saved as tiffs. [file elife-50138-fig3-data1.zip › Figure 3 source data 1/iDKO #916 EdU/RHS xs b DAPI.tif]

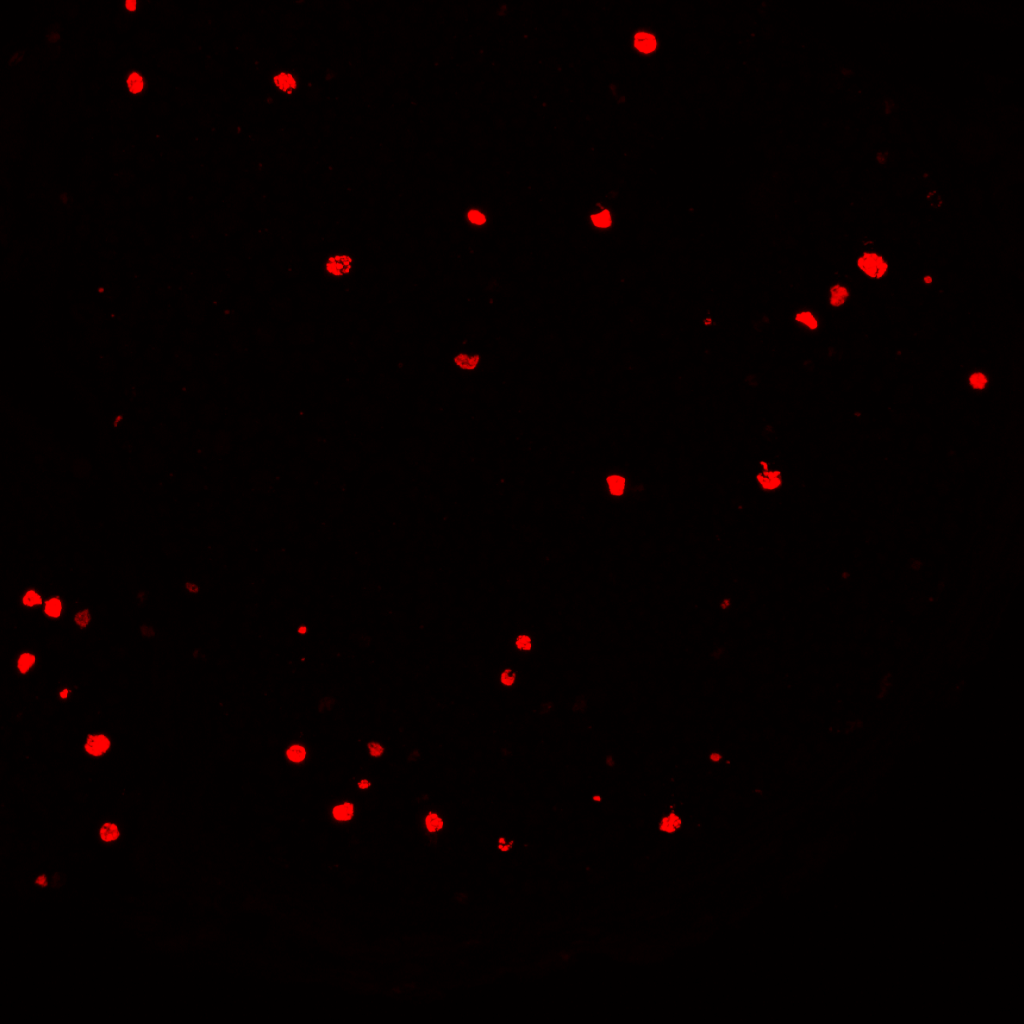

Supplement: Figure 3—source data 1. — This zip archive contains the IHC images for one WT and one iDKO used for the quantitative analysis shown in Figure 3G. Leica SP8 confocal lif images were processed using Imaris software and saved as tiffs. [file elife-50138-fig3-data1.zip › Figure 3 source data 1/iDKO #916 EdU/RHS xs b EdU.tif]

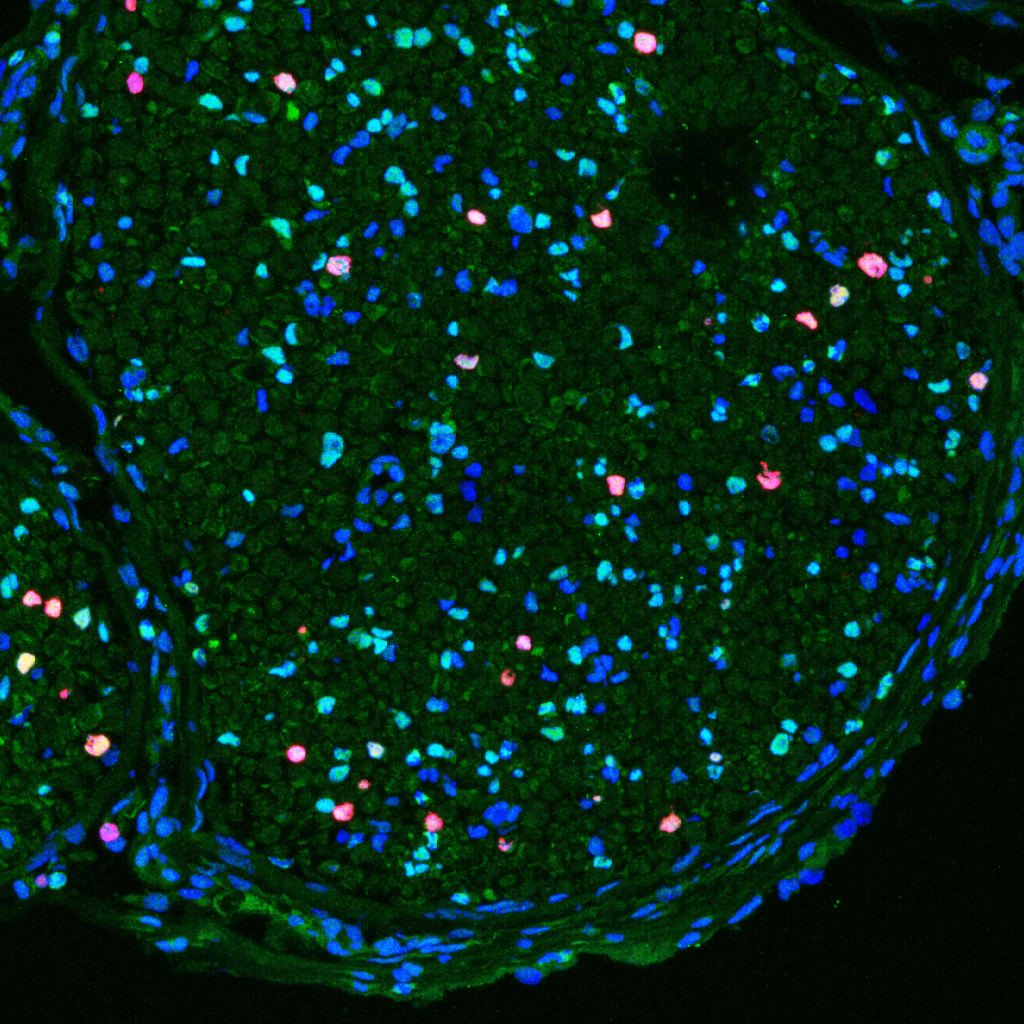

Supplement: Figure 3—source data 1. — This zip archive contains the IHC images for one WT and one iDKO used for the quantitative analysis shown in Figure 3G. Leica SP8 confocal lif images were processed using Imaris software and saved as tiffs. [file elife-50138-fig3-data1.zip › Figure 3 source data 1/iDKO #916 EdU/RHS xs b merge.tif]

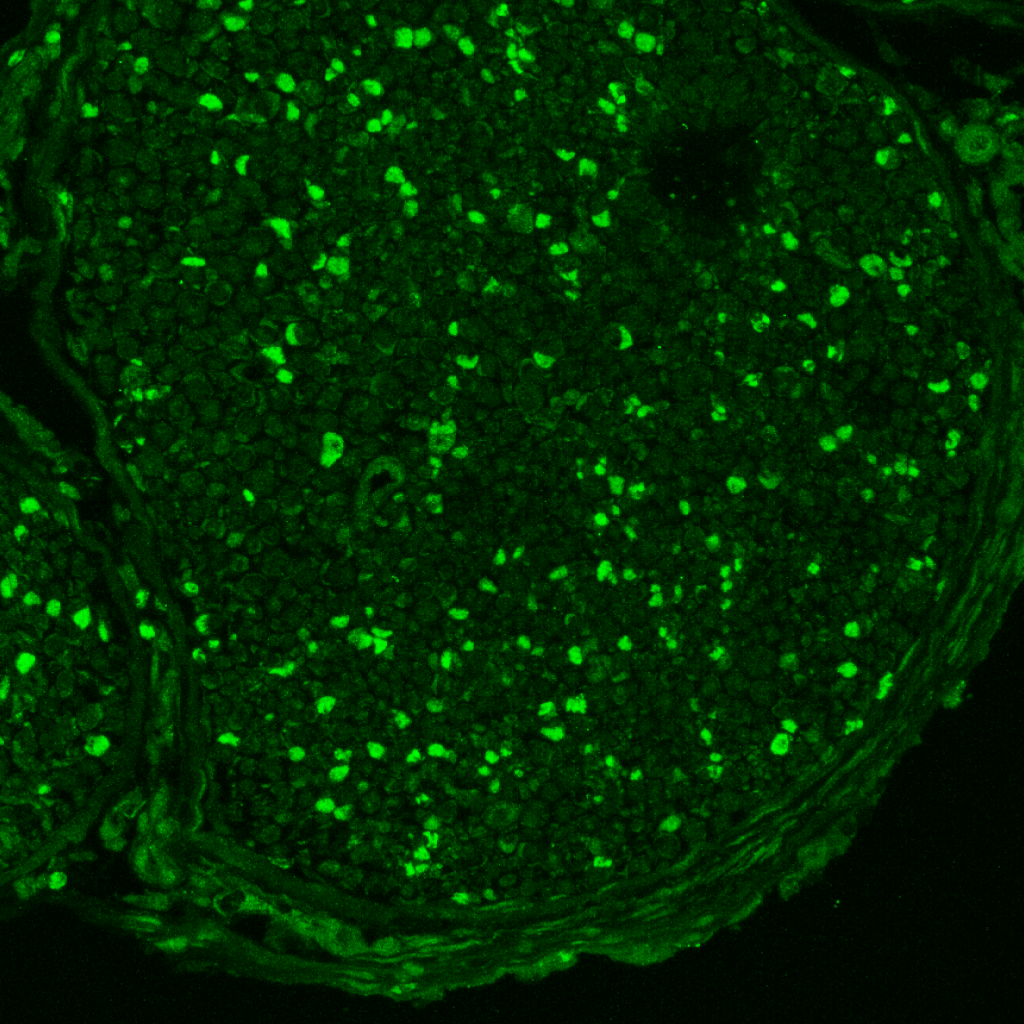

Supplement: Figure 3—source data 1. — This zip archive contains the IHC images for one WT and one iDKO used for the quantitative analysis shown in Figure 3G. Leica SP8 confocal lif images were processed using Imaris software and saved as tiffs. [file elife-50138-fig3-data1.zip › Figure 3 source data 1/iDKO #916 EdU/RHS xs b Sox10.tif]

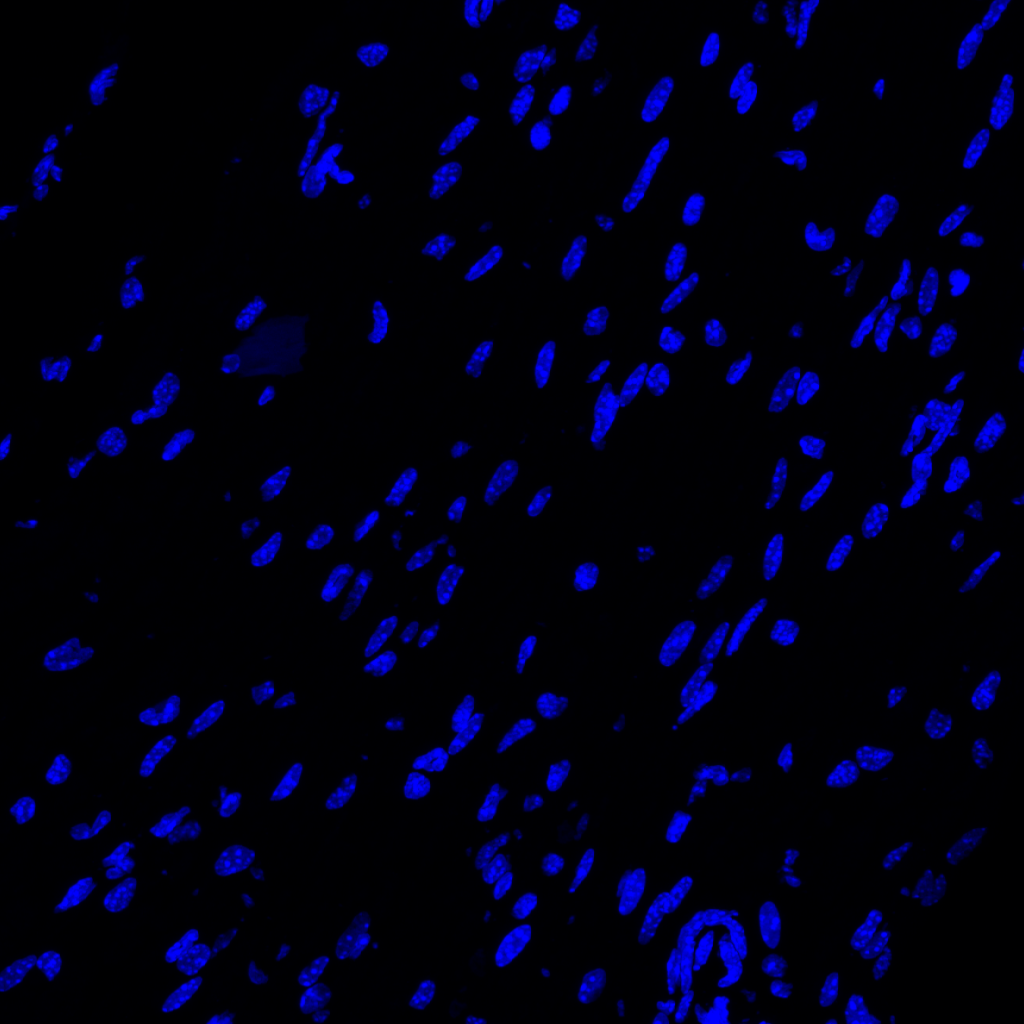

Supplement: Figure 3—source data 1. — This zip archive contains the IHC images for one WT and one iDKO used for the quantitative analysis shown in Figure 3G. Leica SP8 confocal lif images were processed using Imaris software and saved as tiffs. [file elife-50138-fig3-data1.zip › Figure 3 source data 1/WT #942 EdU/RHS a DAPI.tif]

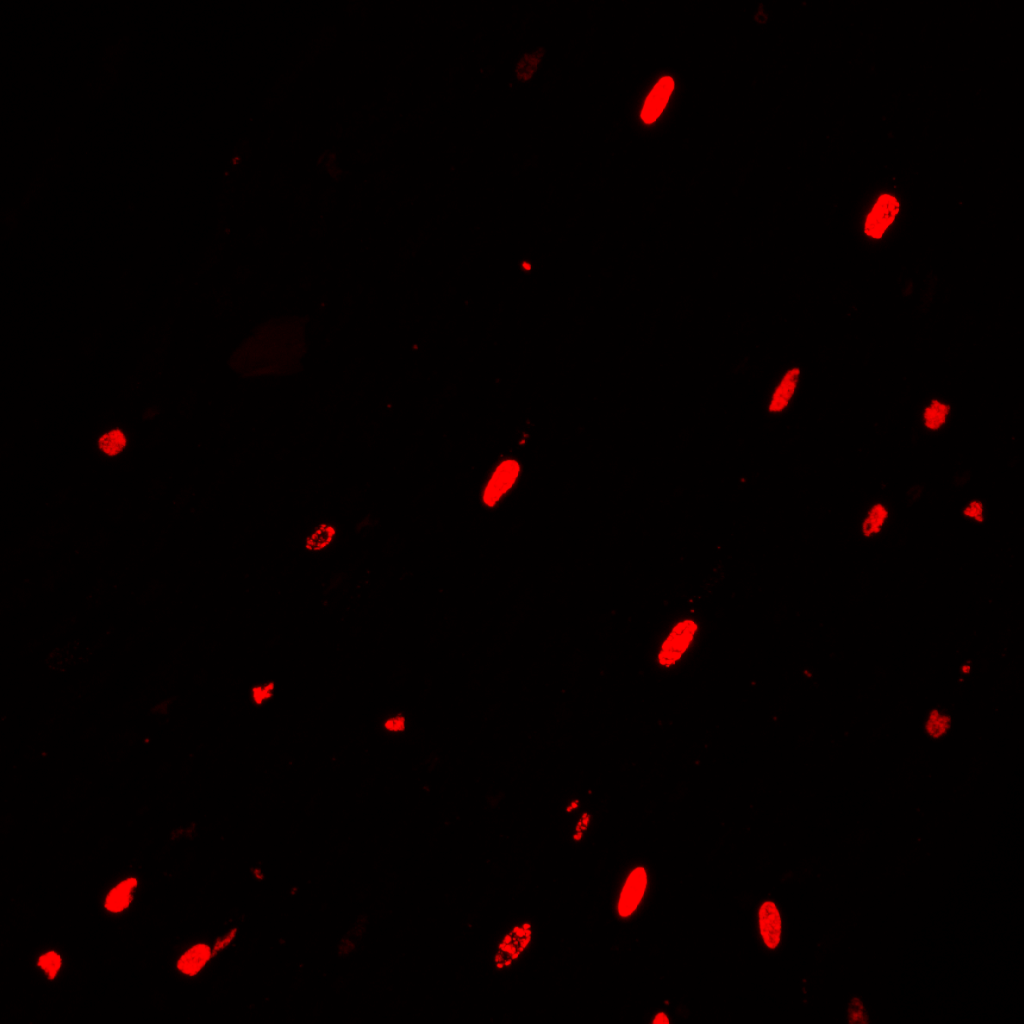

Supplement: Figure 3—source data 1. — This zip archive contains the IHC images for one WT and one iDKO used for the quantitative analysis shown in Figure 3G. Leica SP8 confocal lif images were processed using Imaris software and saved as tiffs. [file elife-50138-fig3-data1.zip › Figure 3 source data 1/WT #942 EdU/RHS a EdU.tif]

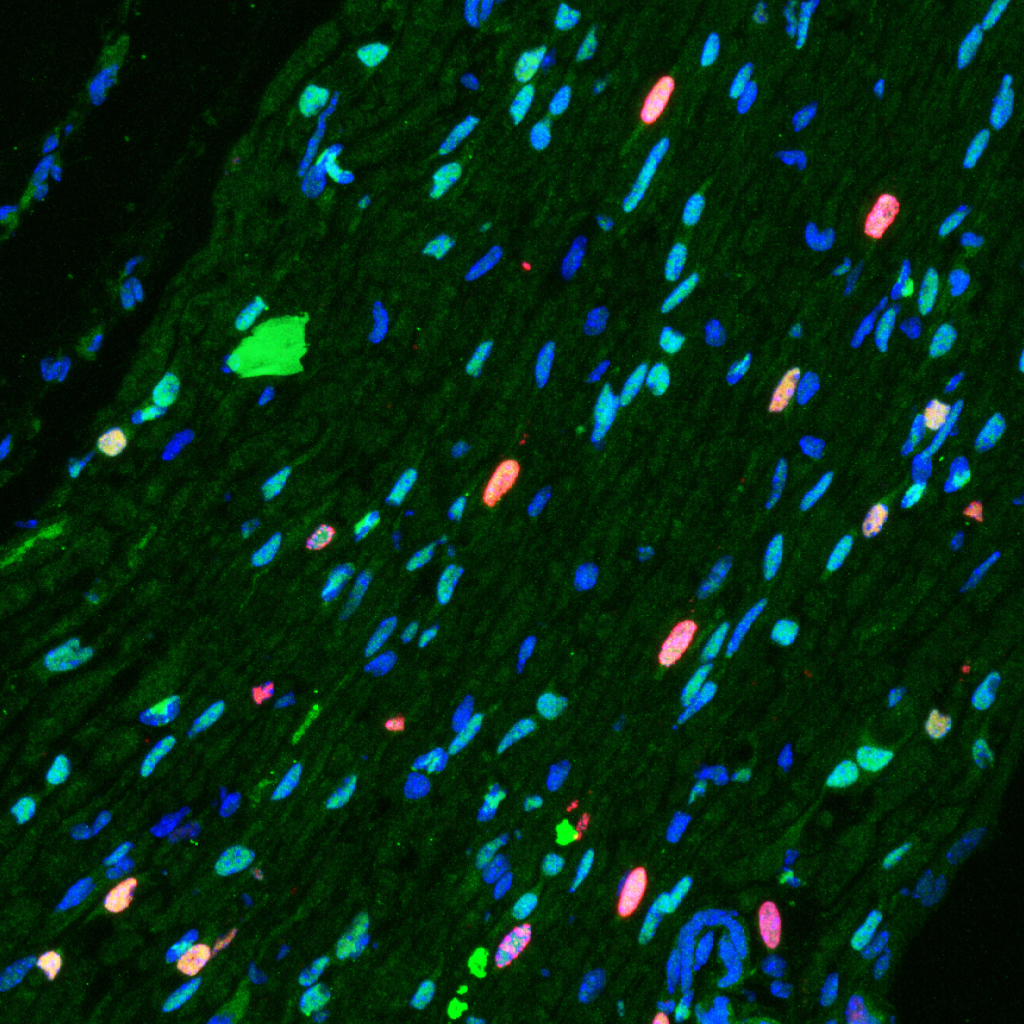

Supplement: Figure 3—source data 1. — This zip archive contains the IHC images for one WT and one iDKO used for the quantitative analysis shown in Figure 3G. Leica SP8 confocal lif images were processed using Imaris software and saved as tiffs. [file elife-50138-fig3-data1.zip › Figure 3 source data 1/WT #942 EdU/RHS a merge.tif]

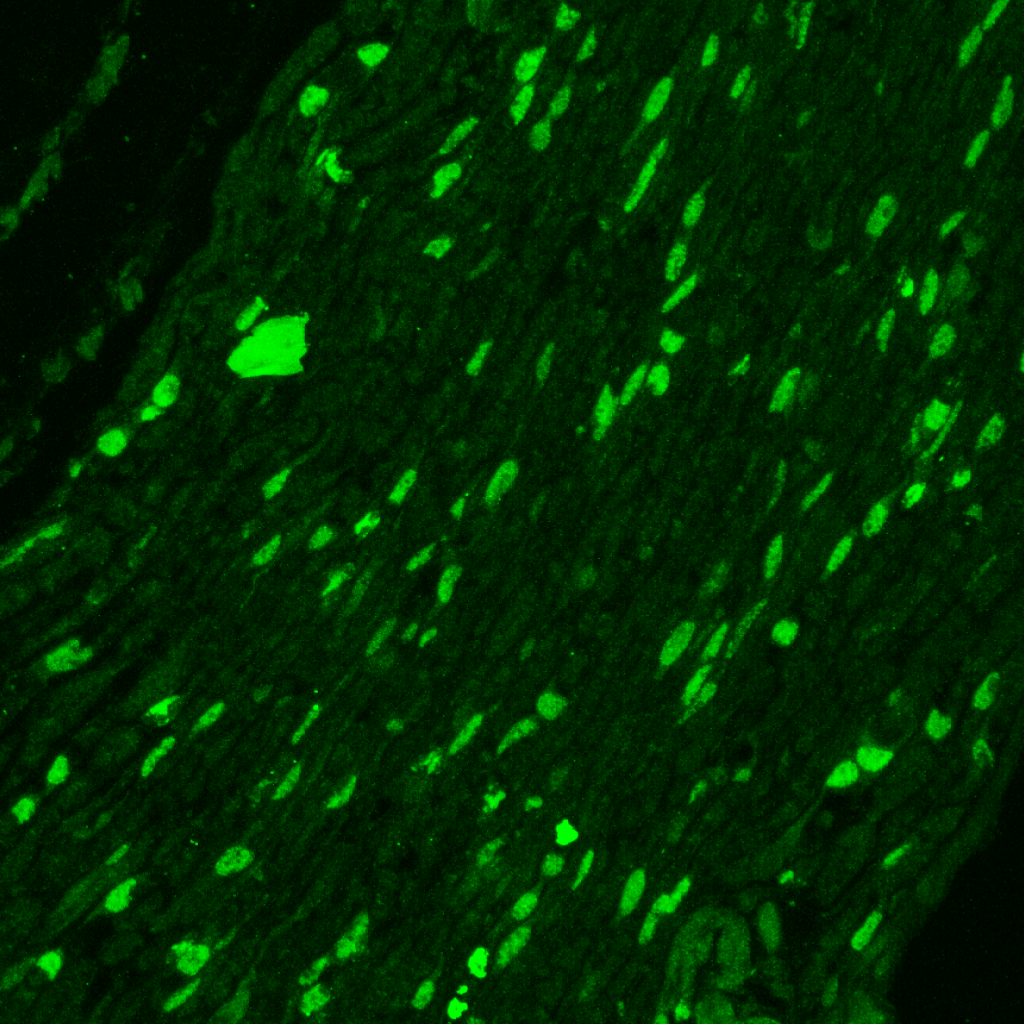

Supplement: Figure 3—source data 1. — This zip archive contains the IHC images for one WT and one iDKO used for the quantitative analysis shown in Figure 3G. Leica SP8 confocal lif images were processed using Imaris software and saved as tiffs. [file elife-50138-fig3-data1.zip › Figure 3 source data 1/WT #942 EdU/RHS a Sox10.tif]

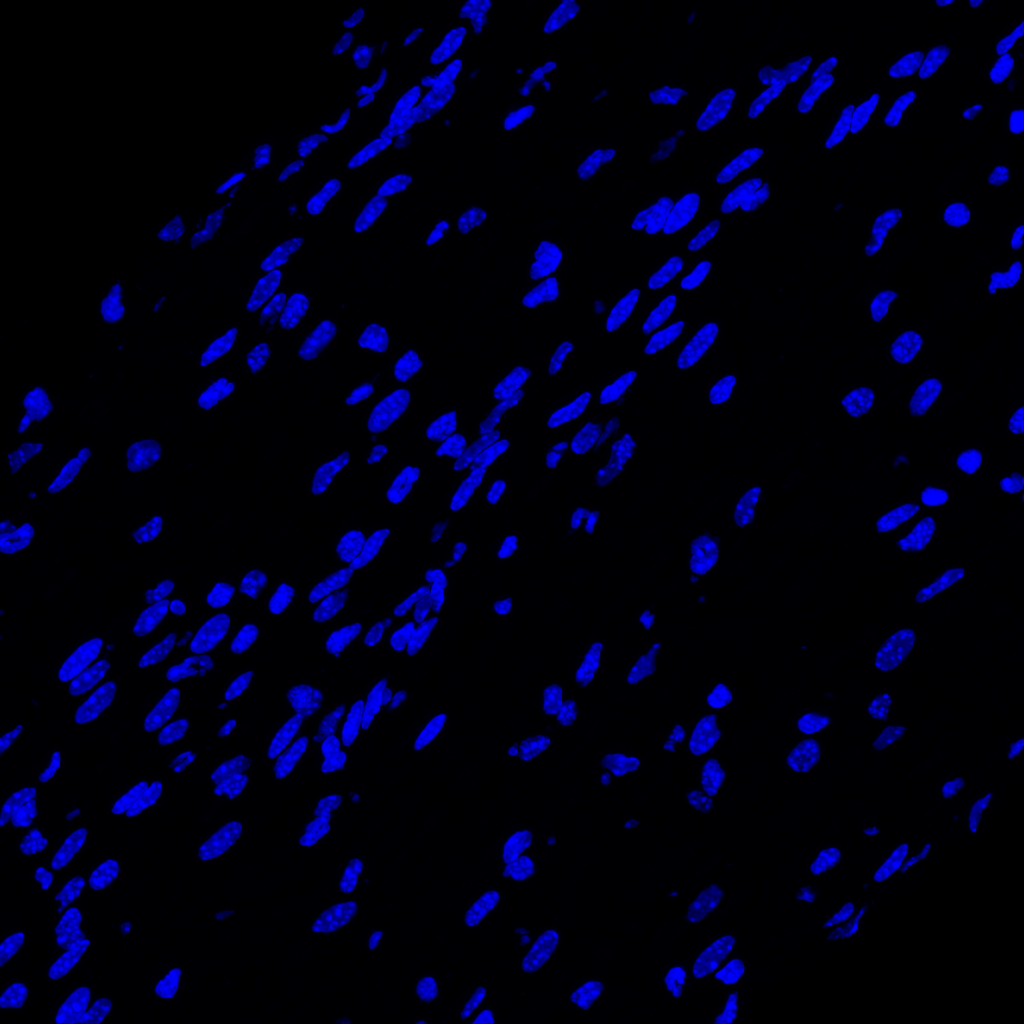

Supplement: Figure 3—source data 1. — This zip archive contains the IHC images for one WT and one iDKO used for the quantitative analysis shown in Figure 3G. Leica SP8 confocal lif images were processed using Imaris software and saved as tiffs. [file elife-50138-fig3-data1.zip › Figure 3 source data 1/WT #942 EdU/RHS b DAPI.tif]

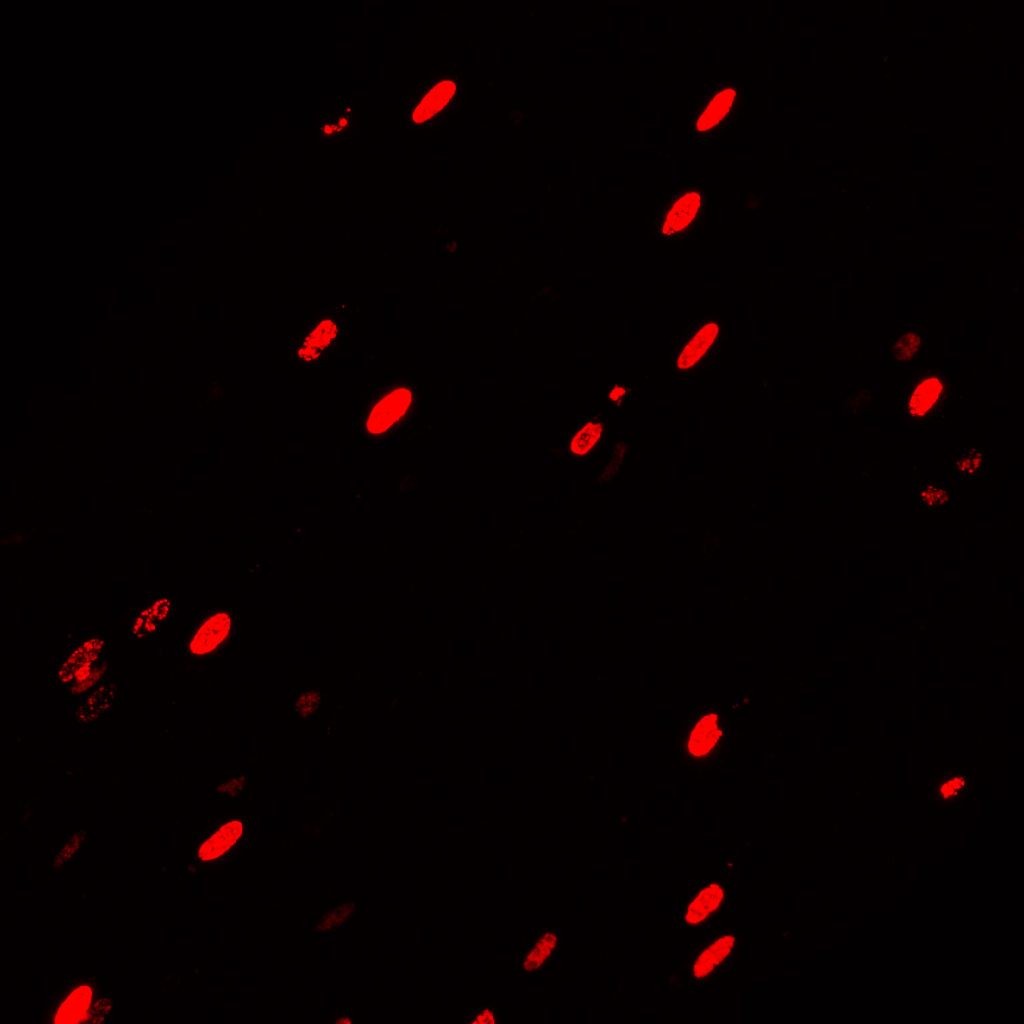

Supplement: Figure 3—source data 1. — This zip archive contains the IHC images for one WT and one iDKO used for the quantitative analysis shown in Figure 3G. Leica SP8 confocal lif images were processed using Imaris software and saved as tiffs. [file elife-50138-fig3-data1.zip › Figure 3 source data 1/WT #942 EdU/RHS b EdU.tif]

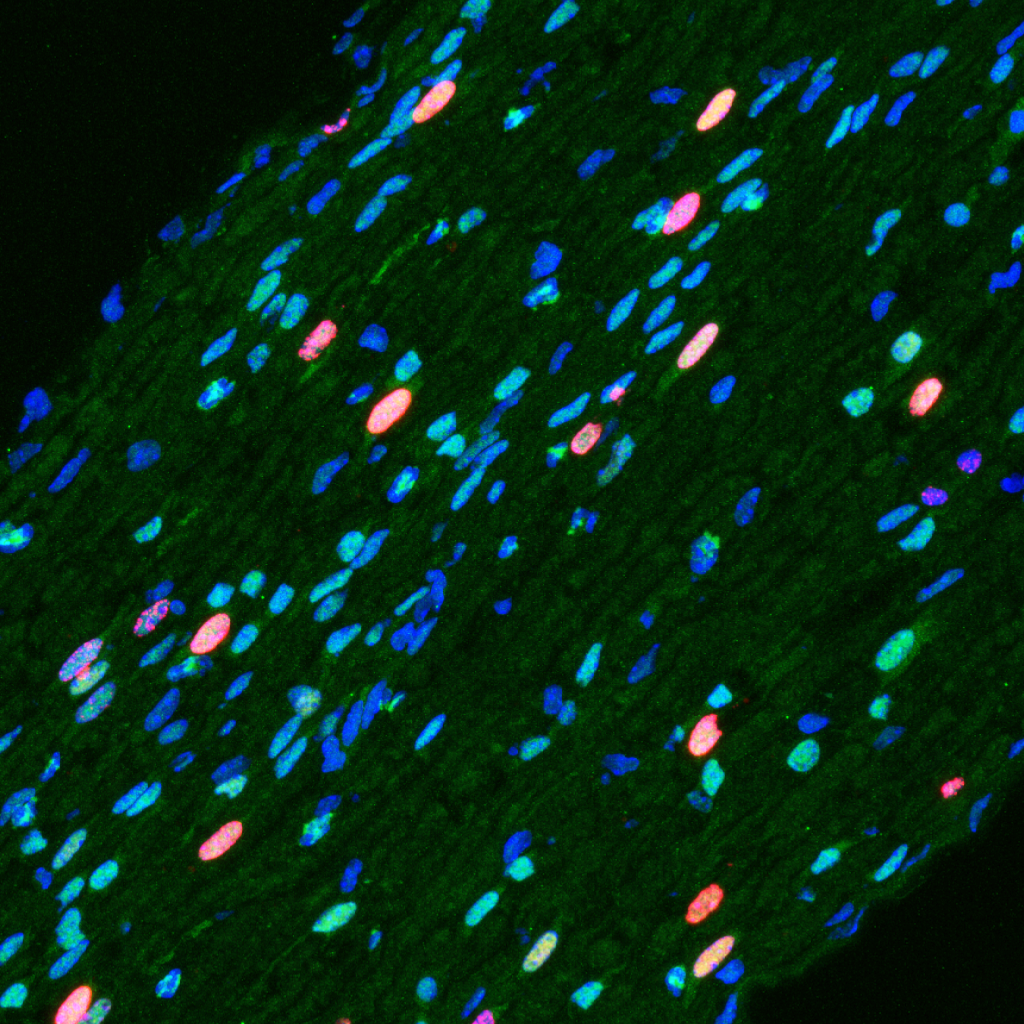

Supplement: Figure 3—source data 1. — This zip archive contains the IHC images for one WT and one iDKO used for the quantitative analysis shown in Figure 3G. Leica SP8 confocal lif images were processed using Imaris software and saved as tiffs. [file elife-50138-fig3-data1.zip › Figure 3 source data 1/WT #942 EdU/RHS b merge.tif]

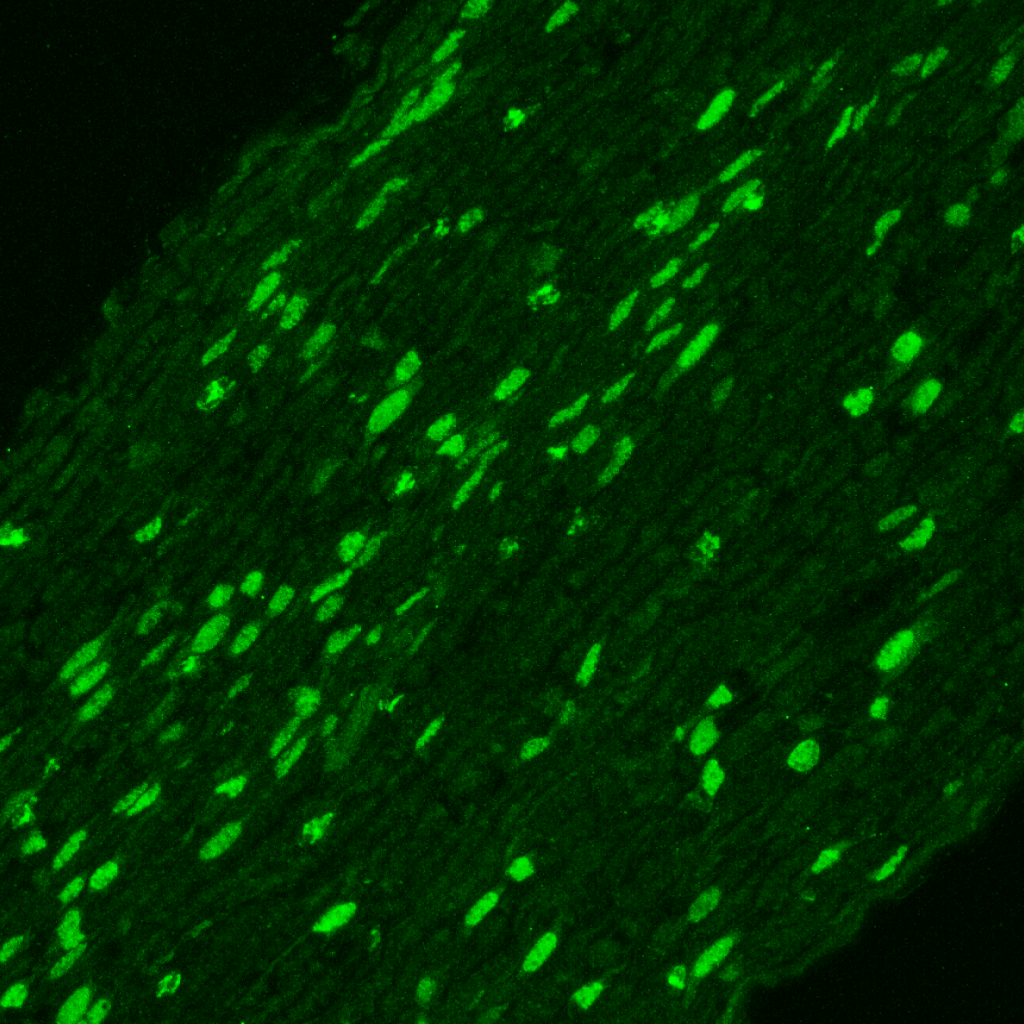

Supplement: Figure 3—source data 1. — This zip archive contains the IHC images for one WT and one iDKO used for the quantitative analysis shown in Figure 3G. Leica SP8 confocal lif images were processed using Imaris software and saved as tiffs. [file elife-50138-fig3-data1.zip › Figure 3 source data 1/WT #942 EdU/RHS b Sox10.tif]

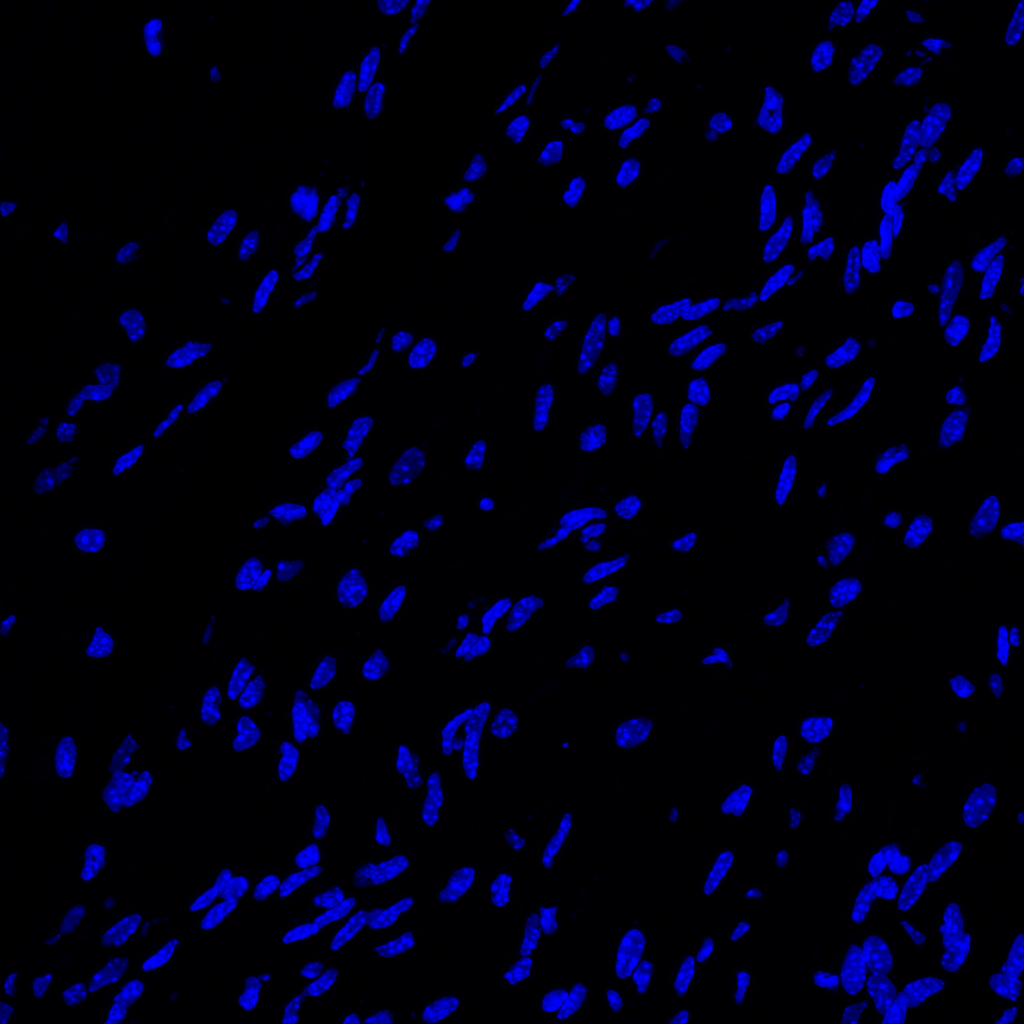

Supplement: Figure 3—source data 1. — This zip archive contains the IHC images for one WT and one iDKO used for the quantitative analysis shown in Figure 3G. Leica SP8 confocal lif images were processed using Imaris software and saved as tiffs. [file elife-50138-fig3-data1.zip › Figure 3 source data 1/WT #942 EdU/RHS c DAPI.tif]

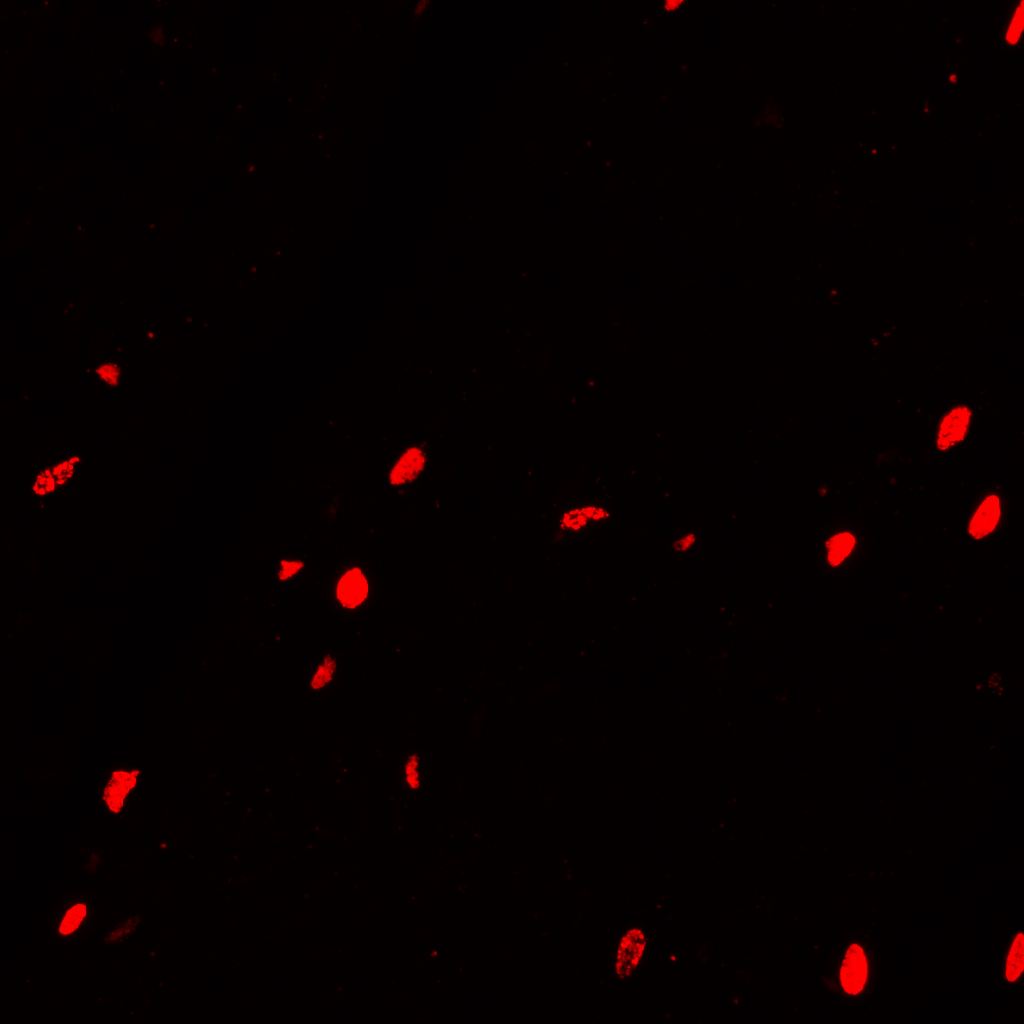

Supplement: Figure 3—source data 1. — This zip archive contains the IHC images for one WT and one iDKO used for the quantitative analysis shown in Figure 3G. Leica SP8 confocal lif images were processed using Imaris software and saved as tiffs. [file elife-50138-fig3-data1.zip › Figure 3 source data 1/WT #942 EdU/RHS c EdU.tif]

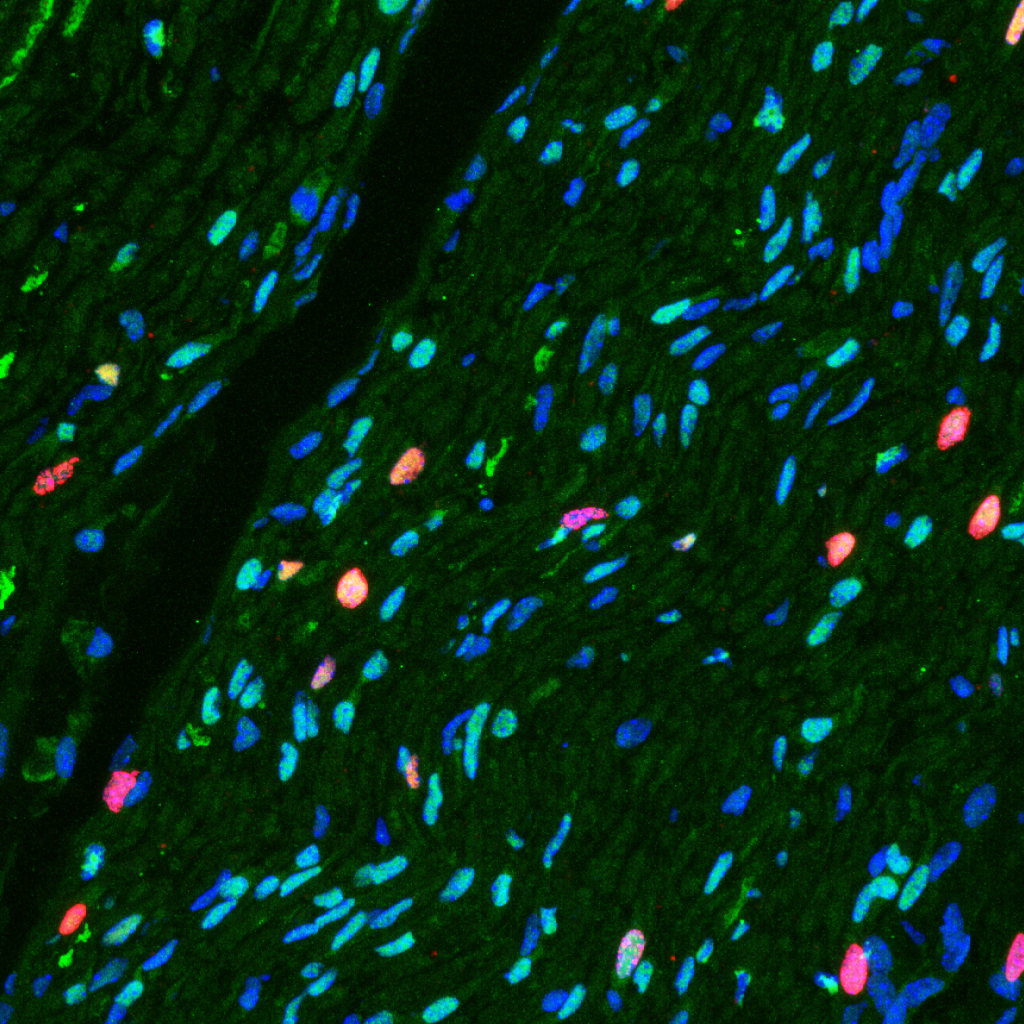

Supplement: Figure 3—source data 1. — This zip archive contains the IHC images for one WT and one iDKO used for the quantitative analysis shown in Figure 3G. Leica SP8 confocal lif images were processed using Imaris software and saved as tiffs. [file elife-50138-fig3-data1.zip › Figure 3 source data 1/WT #942 EdU/RHS c merge.tif]

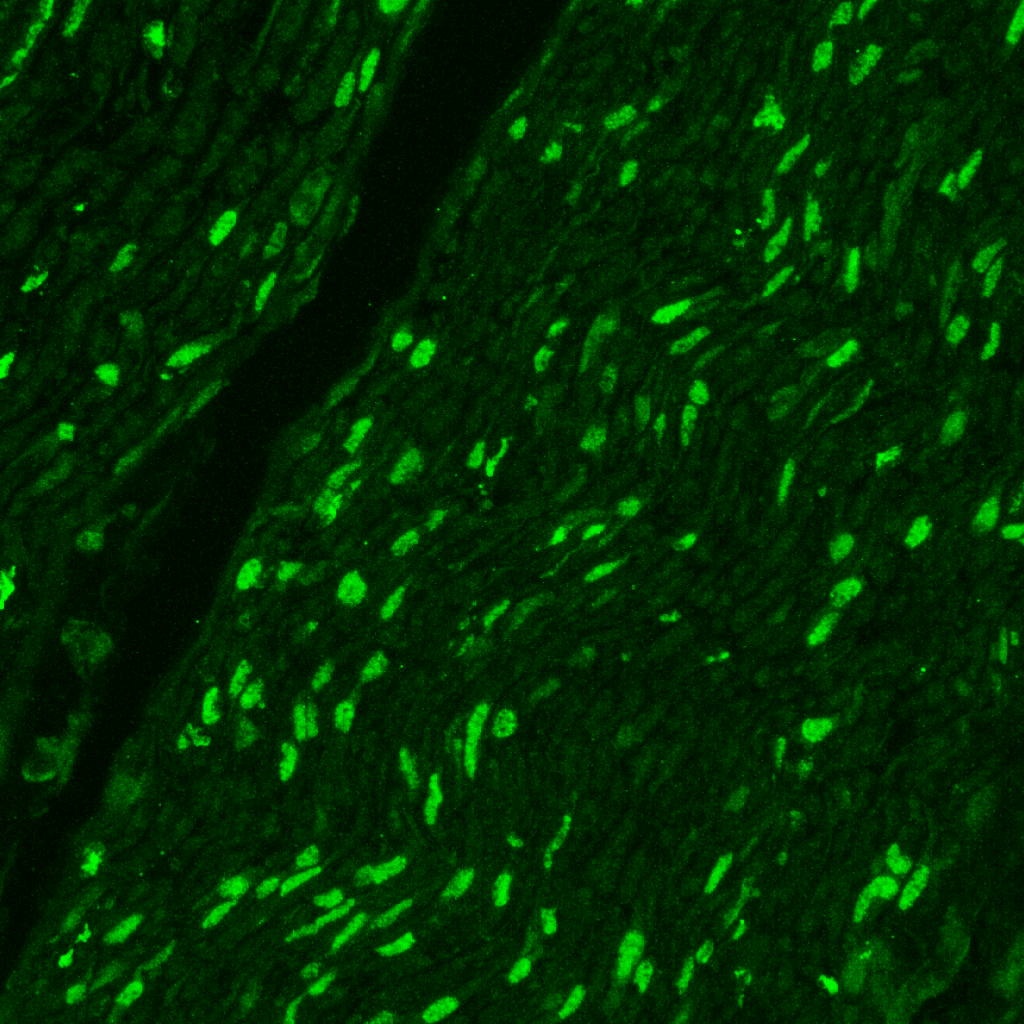

Supplement: Figure 3—source data 1. — This zip archive contains the IHC images for one WT and one iDKO used for the quantitative analysis shown in Figure 3G. Leica SP8 confocal lif images were processed using Imaris software and saved as tiffs. [file elife-50138-fig3-data1.zip › Figure 3 source data 1/WT #942 EdU/RHS c Sox10.tif]

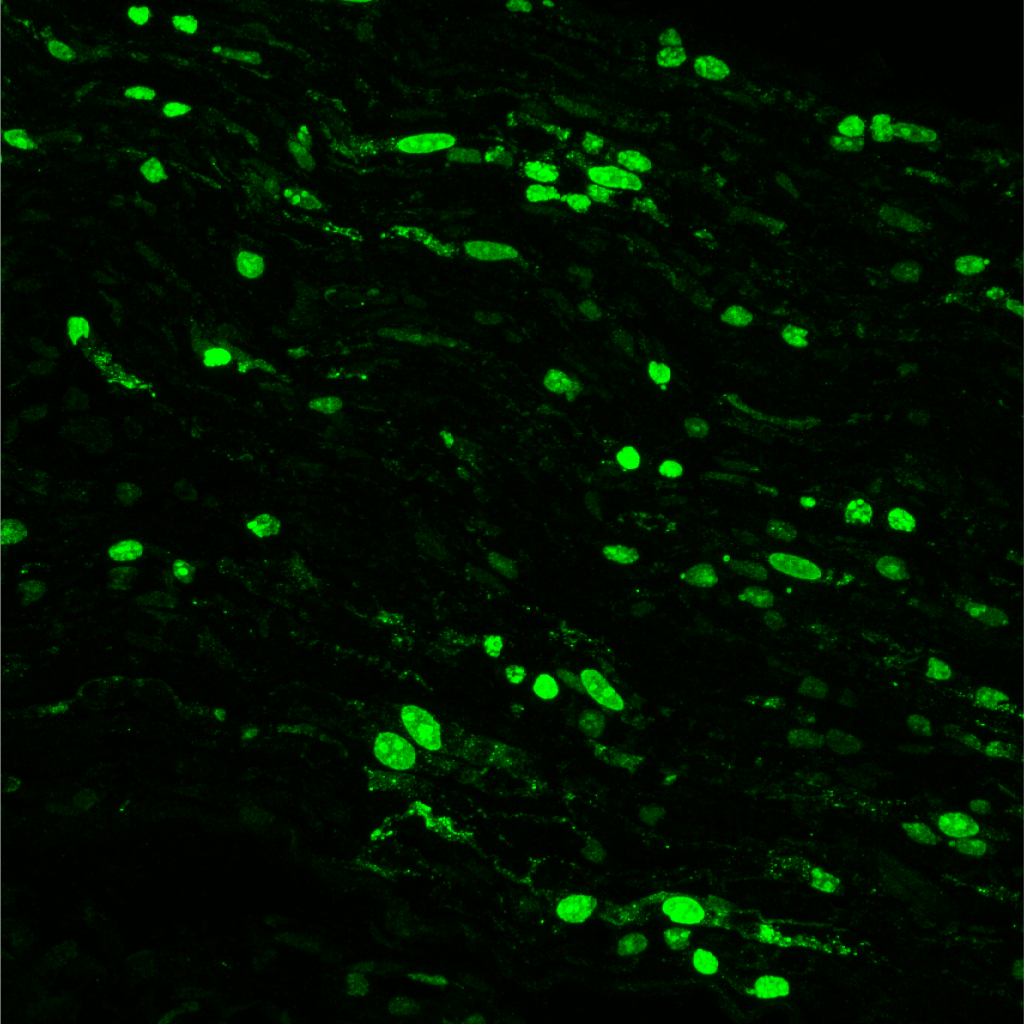

Supplement: Figure 3—source data 2. — This zip archive contains the IHC for one WT and one iDKO used for quantitative analysis shown in Figure 3H. Results and quantitation shown in the Figure used BD #550609 anti-Ki67. These results were confirmed using a second antibody, Abcam #ab15580 anti-Ki67. Images using both antibodies are included in the zip archive, in the indicated folders. Leica SP8 confocal lif images were processed using Imaris software and saved as tiffs. [file elife-50138-fig3-data2.zip › Figure 3 source data 2/iDKO #916 Ki67/Abcam #15580 Ki67/Series 16 Ki67.tif]

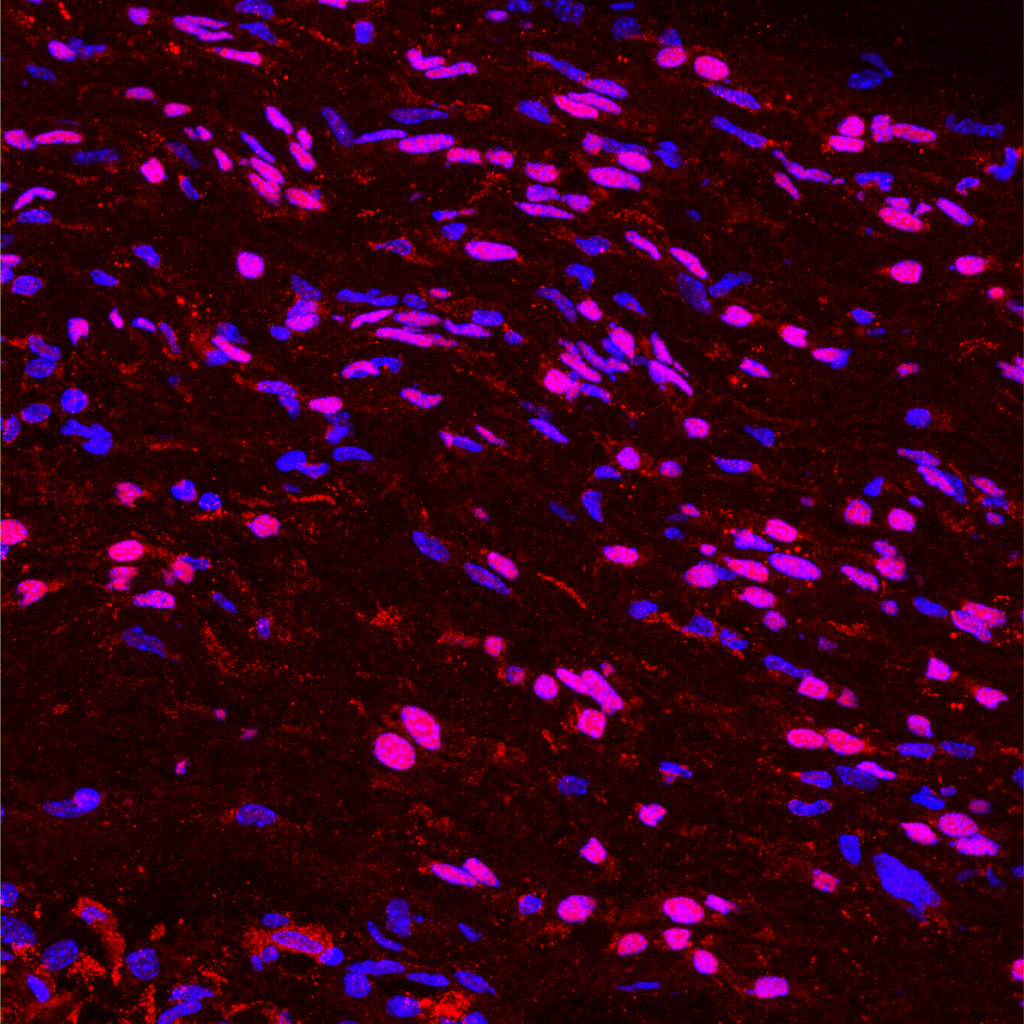

Supplement: Figure 3—source data 2. — This zip archive contains the IHC for one WT and one iDKO used for quantitative analysis shown in Figure 3H. Results and quantitation shown in the Figure used BD #550609 anti-Ki67. These results were confirmed using a second antibody, Abcam #ab15580 anti-Ki67. Images using both antibodies are included in the zip archive, in the indicated folders. Leica SP8 confocal lif images were processed using Imaris software and saved as tiffs. [file elife-50138-fig3-data2.zip › Figure 3 source data 2/iDKO #916 Ki67/Abcam #15580 Ki67/Series 16 Sox10 + DAPI.tif]

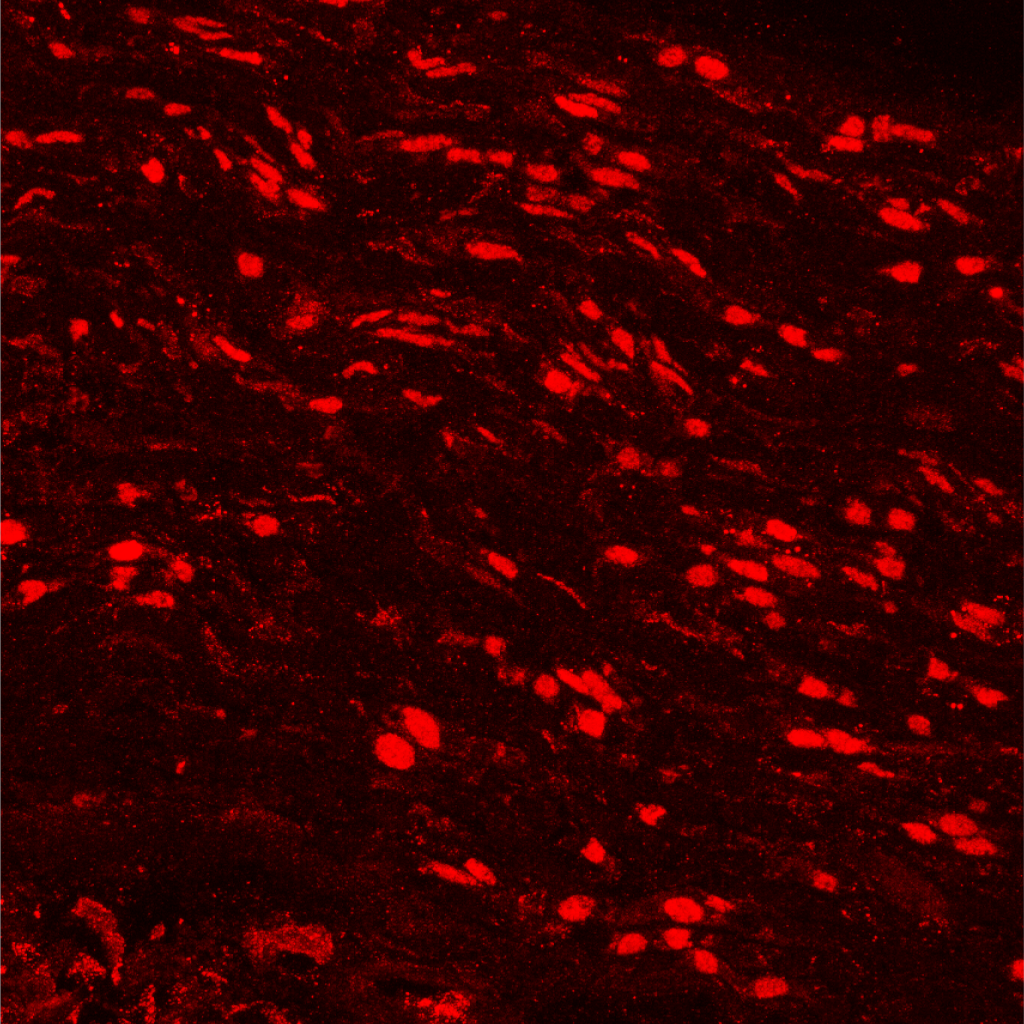

Supplement: Figure 3—source data 2. — This zip archive contains the IHC for one WT and one iDKO used for quantitative analysis shown in Figure 3H. Results and quantitation shown in the Figure used BD #550609 anti-Ki67. These results were confirmed using a second antibody, Abcam #ab15580 anti-Ki67. Images using both antibodies are included in the zip archive, in the indicated folders. Leica SP8 confocal lif images were processed using Imaris software and saved as tiffs. [file elife-50138-fig3-data2.zip › Figure 3 source data 2/iDKO #916 Ki67/Abcam #15580 Ki67/Series 16 Sox10.tif]

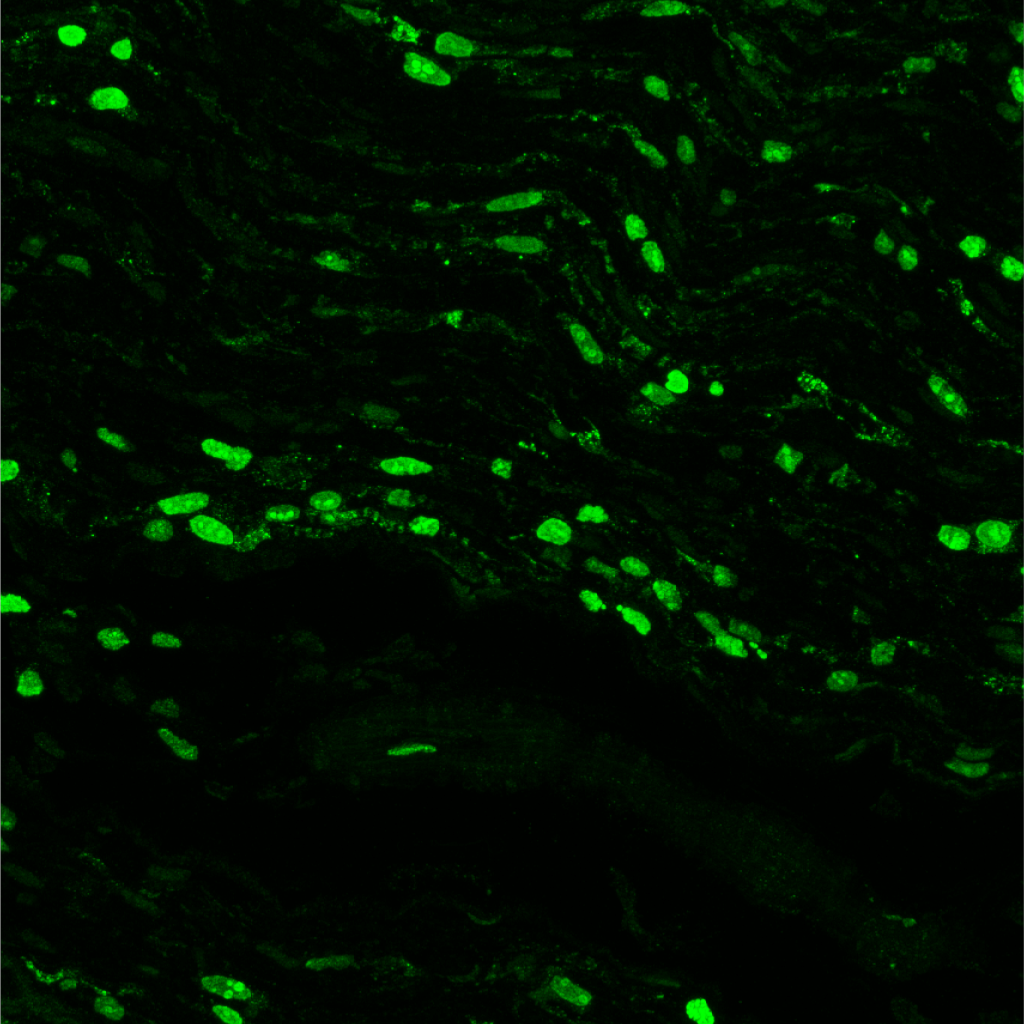

Supplement: Figure 3—source data 2. — This zip archive contains the IHC for one WT and one iDKO used for quantitative analysis shown in Figure 3H. Results and quantitation shown in the Figure used BD #550609 anti-Ki67. These results were confirmed using a second antibody, Abcam #ab15580 anti-Ki67. Images using both antibodies are included in the zip archive, in the indicated folders. Leica SP8 confocal lif images were processed using Imaris software and saved as tiffs. [file elife-50138-fig3-data2.zip › Figure 3 source data 2/iDKO #916 Ki67/Abcam #15580 Ki67/Series 24 Ki67.tif]

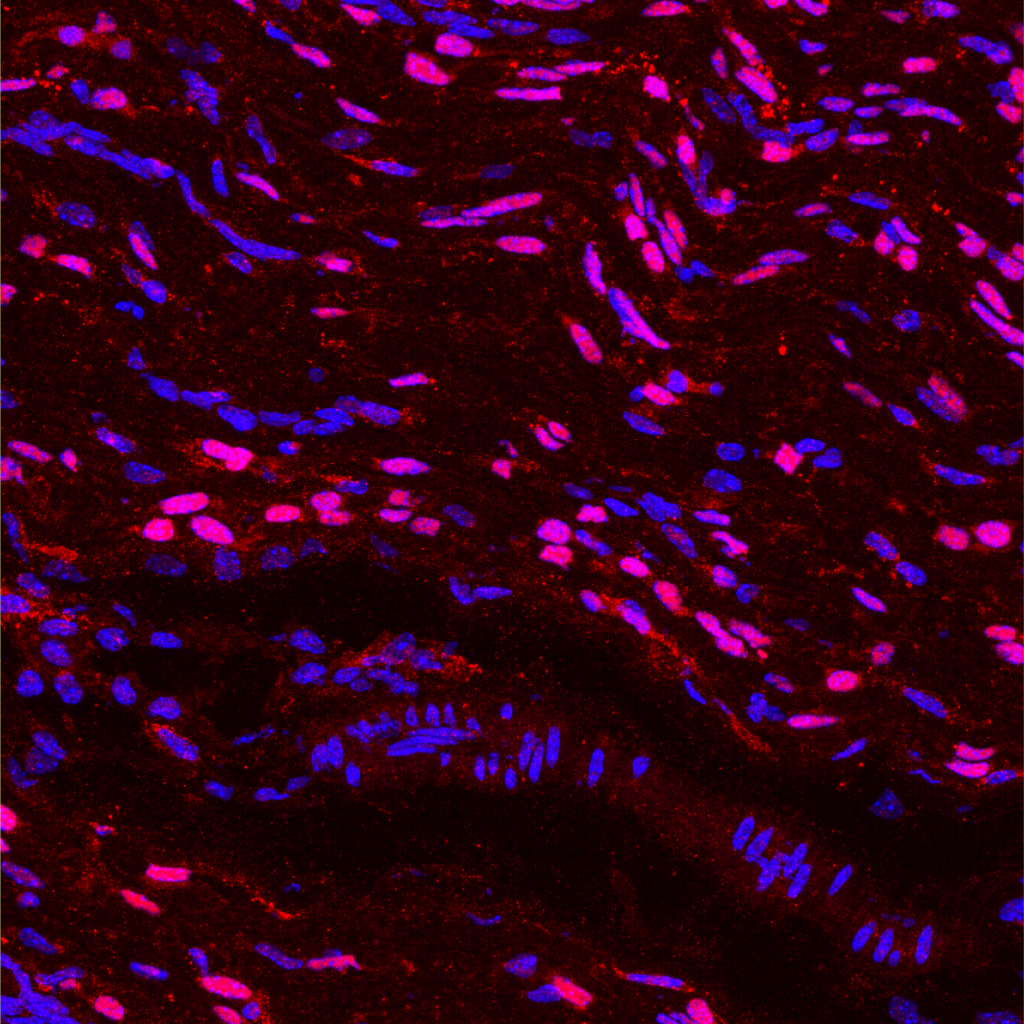

Supplement: Figure 3—source data 2. — This zip archive contains the IHC for one WT and one iDKO used for quantitative analysis shown in Figure 3H. Results and quantitation shown in the Figure used BD #550609 anti-Ki67. These results were confirmed using a second antibody, Abcam #ab15580 anti-Ki67. Images using both antibodies are included in the zip archive, in the indicated folders. Leica SP8 confocal lif images were processed using Imaris software and saved as tiffs. [file elife-50138-fig3-data2.zip › Figure 3 source data 2/iDKO #916 Ki67/Abcam #15580 Ki67/Series 24 Sox10 + DAPI.tif]

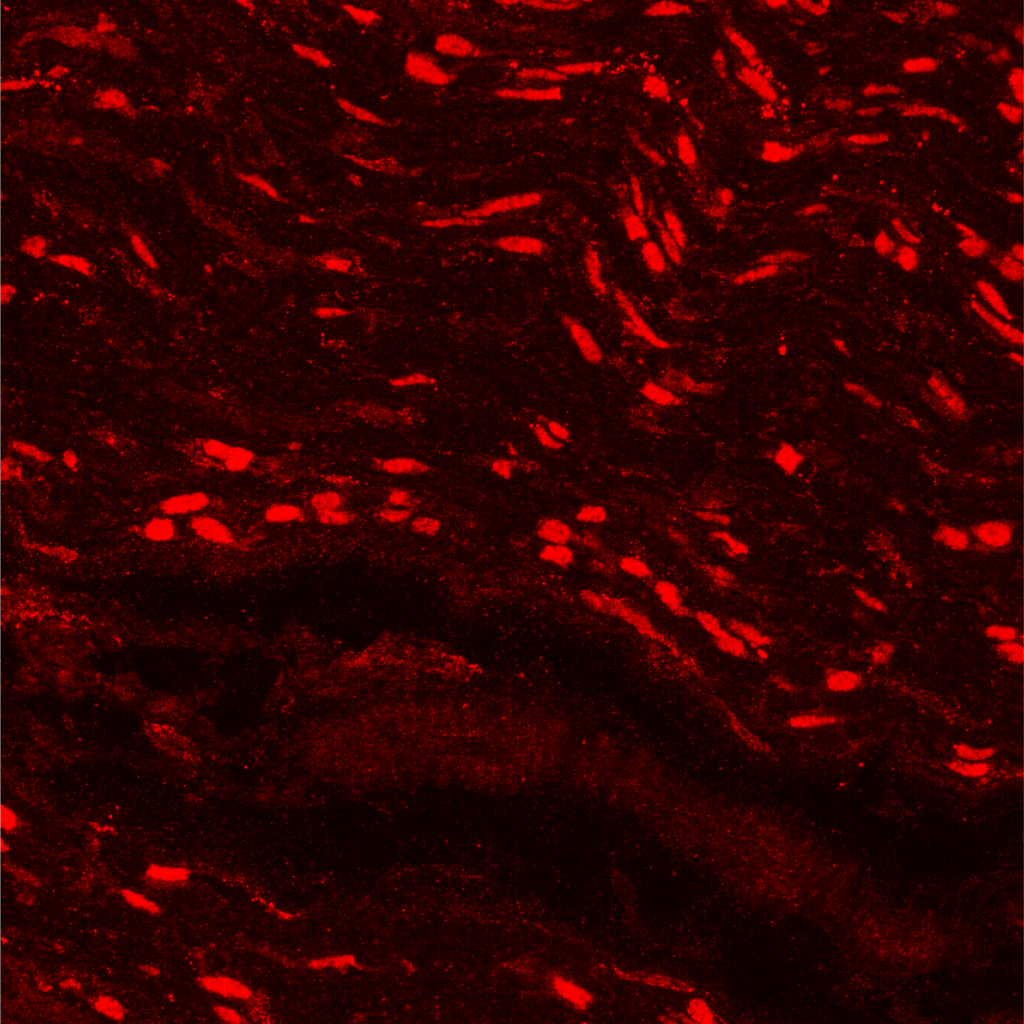

Supplement: Figure 3—source data 2. — This zip archive contains the IHC for one WT and one iDKO used for quantitative analysis shown in Figure 3H. Results and quantitation shown in the Figure used BD #550609 anti-Ki67. These results were confirmed using a second antibody, Abcam #ab15580 anti-Ki67. Images using both antibodies are included in the zip archive, in the indicated folders. Leica SP8 confocal lif images were processed using Imaris software and saved as tiffs. [file elife-50138-fig3-data2.zip › Figure 3 source data 2/iDKO #916 Ki67/Abcam #15580 Ki67/Series 24 Sox10.tif]

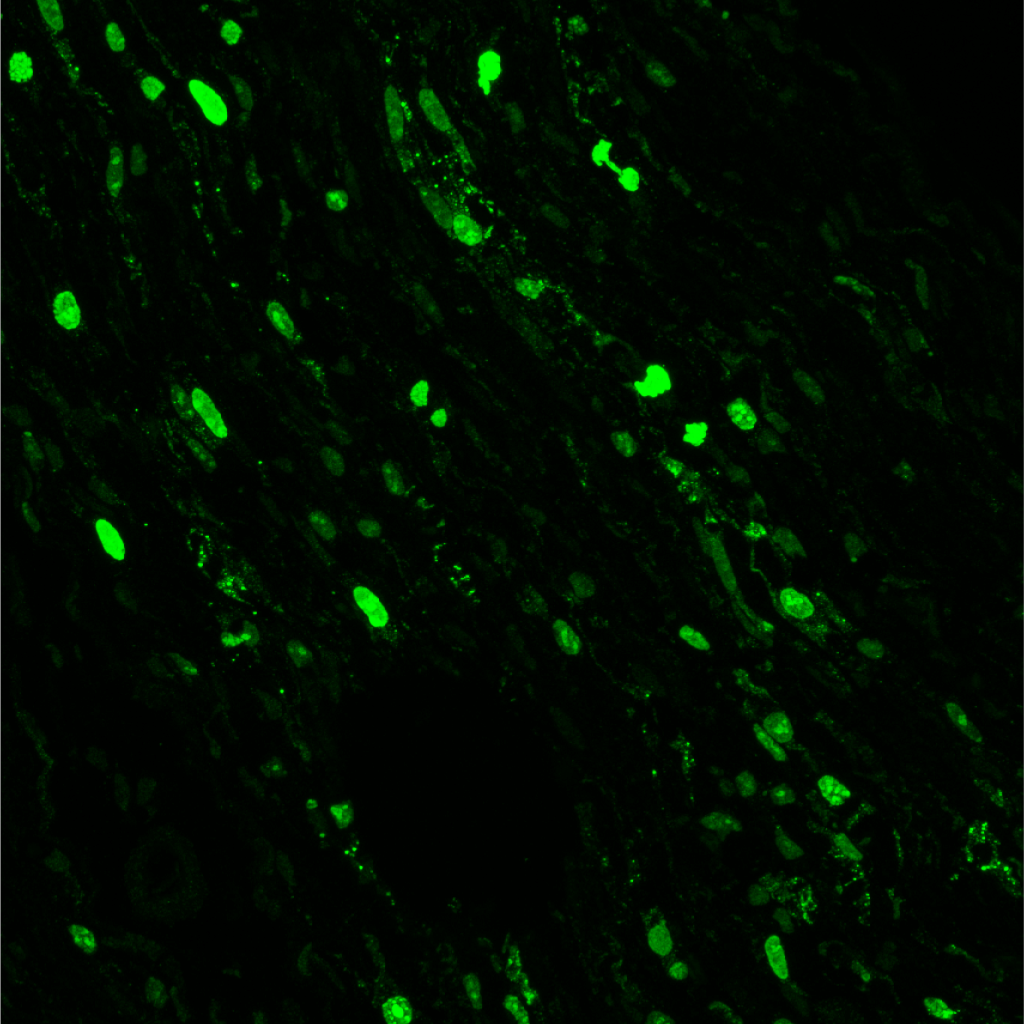

Supplement: Figure 3—source data 2. — This zip archive contains the IHC for one WT and one iDKO used for quantitative analysis shown in Figure 3H. Results and quantitation shown in the Figure used BD #550609 anti-Ki67. These results were confirmed using a second antibody, Abcam #ab15580 anti-Ki67. Images using both antibodies are included in the zip archive, in the indicated folders. Leica SP8 confocal lif images were processed using Imaris software and saved as tiffs. [file elife-50138-fig3-data2.zip › Figure 3 source data 2/iDKO #916 Ki67/Abcam #15580 Ki67/Series 36 Ki67.tif]

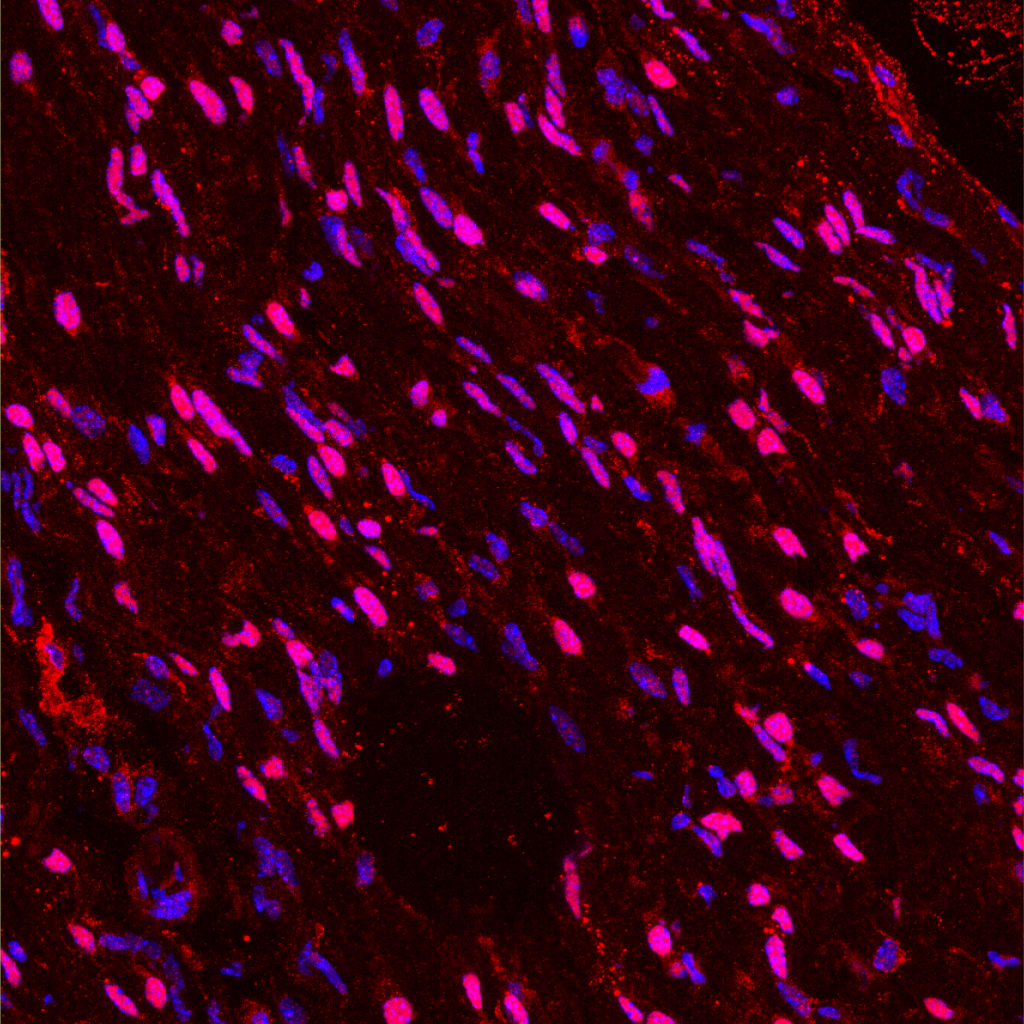

Supplement: Figure 3—source data 2. — This zip archive contains the IHC for one WT and one iDKO used for quantitative analysis shown in Figure 3H. Results and quantitation shown in the Figure used BD #550609 anti-Ki67. These results were confirmed using a second antibody, Abcam #ab15580 anti-Ki67. Images using both antibodies are included in the zip archive, in the indicated folders. Leica SP8 confocal lif images were processed using Imaris software and saved as tiffs. [file elife-50138-fig3-data2.zip › Figure 3 source data 2/iDKO #916 Ki67/Abcam #15580 Ki67/Series 36 Sox10 + DAPI.tif]

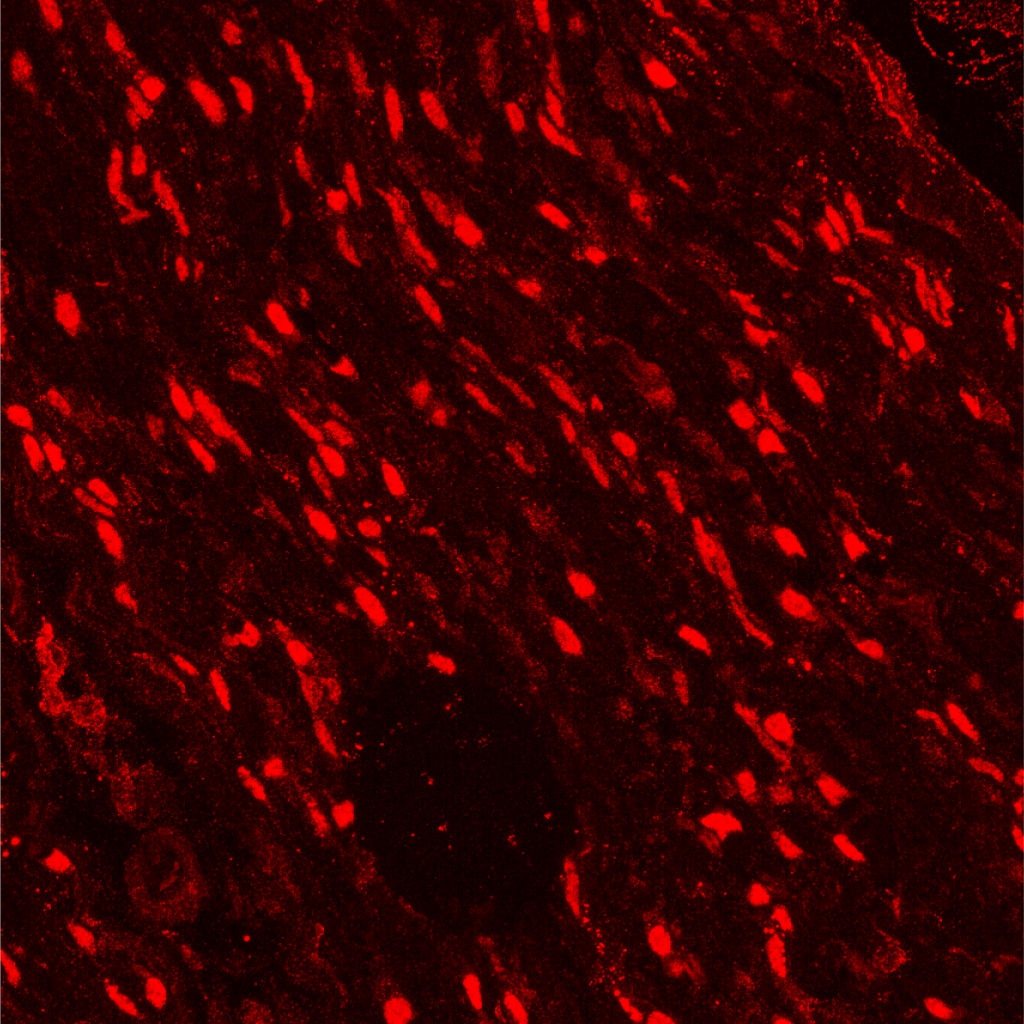

Supplement: Figure 3—source data 2. — This zip archive contains the IHC for one WT and one iDKO used for quantitative analysis shown in Figure 3H. Results and quantitation shown in the Figure used BD #550609 anti-Ki67. These results were confirmed using a second antibody, Abcam #ab15580 anti-Ki67. Images using both antibodies are included in the zip archive, in the indicated folders. Leica SP8 confocal lif images were processed using Imaris software and saved as tiffs. [file elife-50138-fig3-data2.zip › Figure 3 source data 2/iDKO #916 Ki67/Abcam #15580 Ki67/Series 36 Sox10.tif]

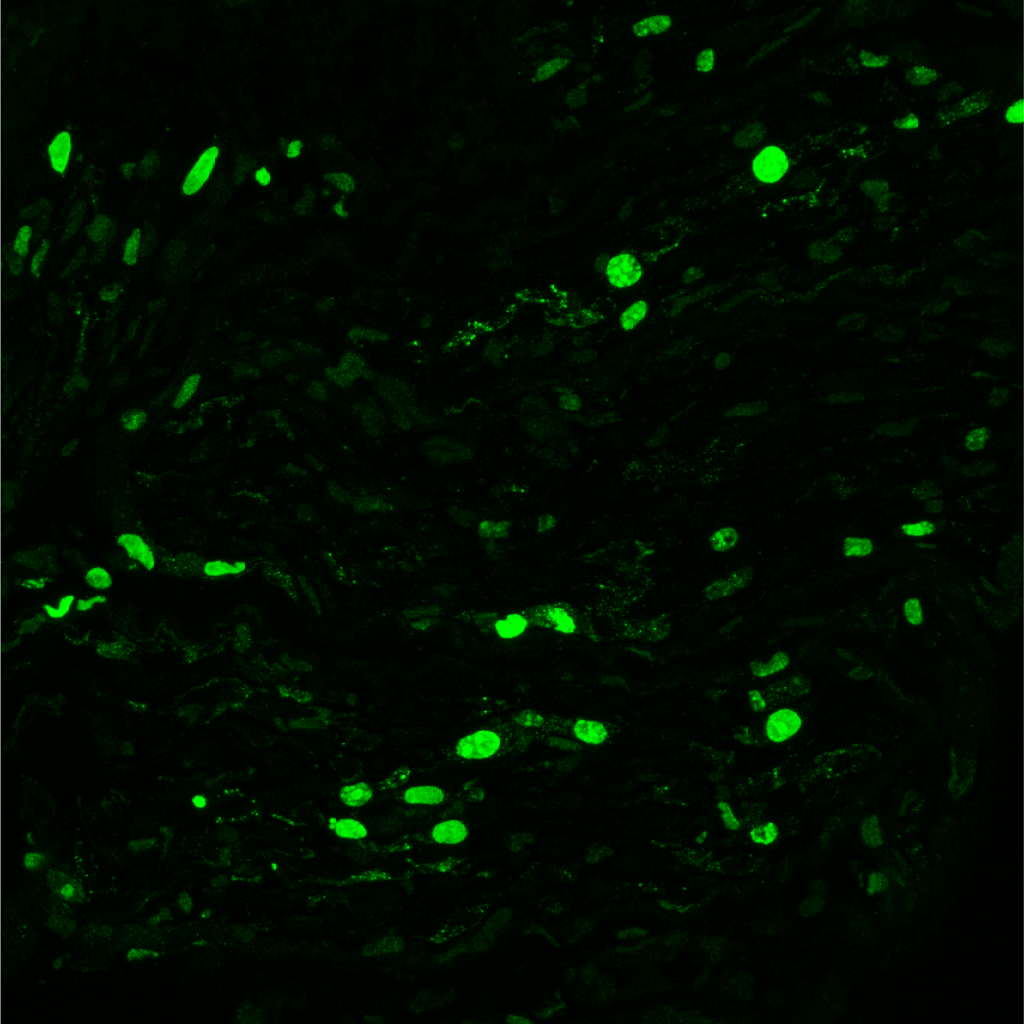

Supplement: Figure 3—source data 2. — This zip archive contains the IHC for one WT and one iDKO used for quantitative analysis shown in Figure 3H. Results and quantitation shown in the Figure used BD #550609 anti-Ki67. These results were confirmed using a second antibody, Abcam #ab15580 anti-Ki67. Images using both antibodies are included in the zip archive, in the indicated folders. Leica SP8 confocal lif images were processed using Imaris software and saved as tiffs. [file elife-50138-fig3-data2.zip › Figure 3 source data 2/iDKO #916 Ki67/Abcam #15580 Ki67/Series 8 Ki67.tif]

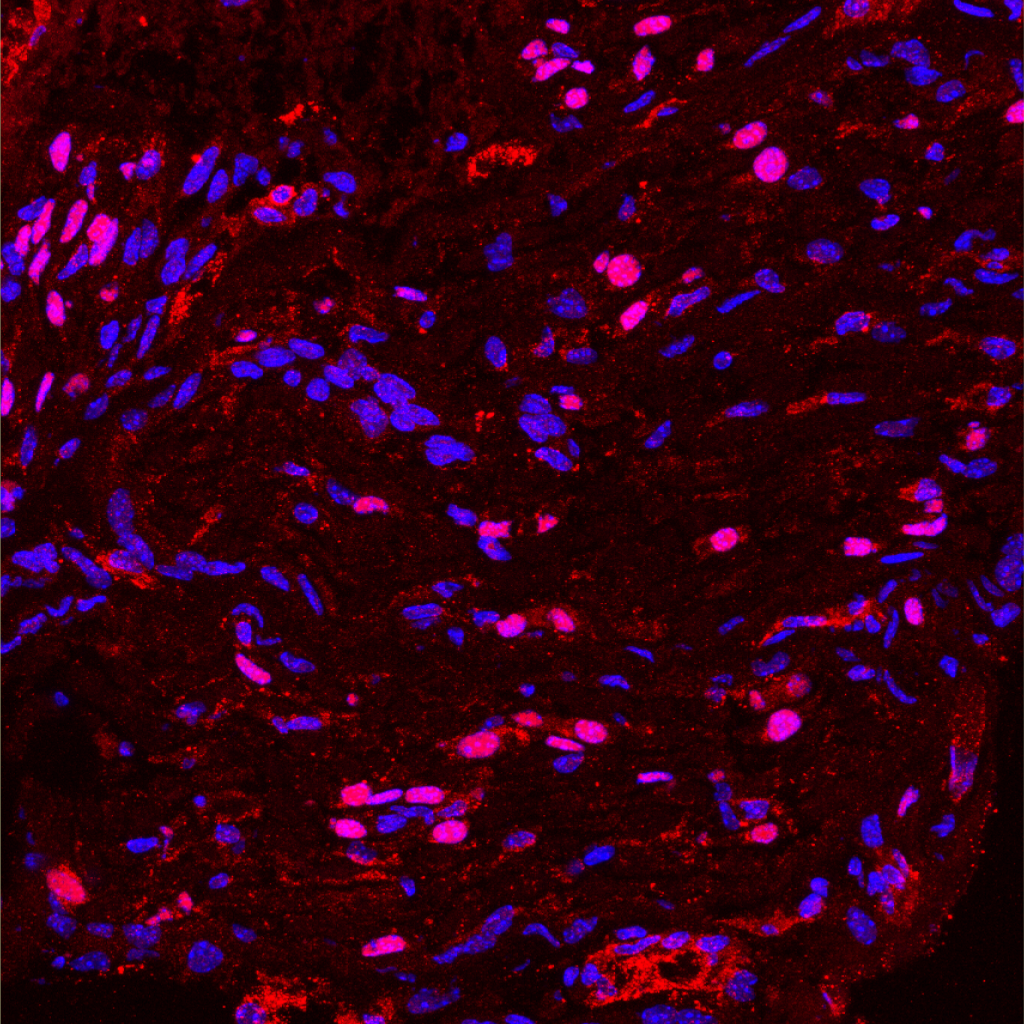

Supplement: Figure 3—source data 2. — This zip archive contains the IHC for one WT and one iDKO used for quantitative analysis shown in Figure 3H. Results and quantitation shown in the Figure used BD #550609 anti-Ki67. These results were confirmed using a second antibody, Abcam #ab15580 anti-Ki67. Images using both antibodies are included in the zip archive, in the indicated folders. Leica SP8 confocal lif images were processed using Imaris software and saved as tiffs. [file elife-50138-fig3-data2.zip › Figure 3 source data 2/iDKO #916 Ki67/Abcam #15580 Ki67/Series 8 Sox10 + DAPI.tif]

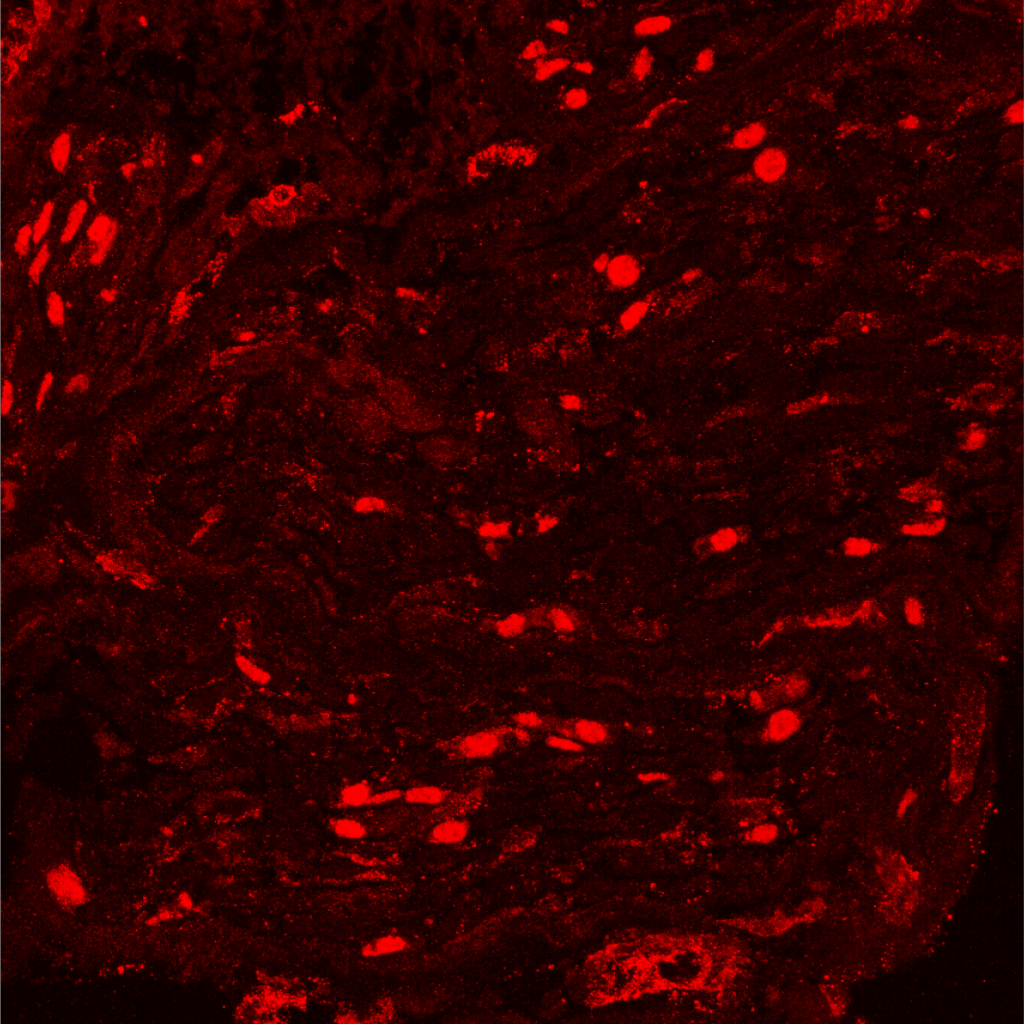

Supplement: Figure 3—source data 2. — This zip archive contains the IHC for one WT and one iDKO used for quantitative analysis shown in Figure 3H. Results and quantitation shown in the Figure used BD #550609 anti-Ki67. These results were confirmed using a second antibody, Abcam #ab15580 anti-Ki67. Images using both antibodies are included in the zip archive, in the indicated folders. Leica SP8 confocal lif images were processed using Imaris software and saved as tiffs. [file elife-50138-fig3-data2.zip › Figure 3 source data 2/iDKO #916 Ki67/Abcam #15580 Ki67/Series 8 Sox10.tif]

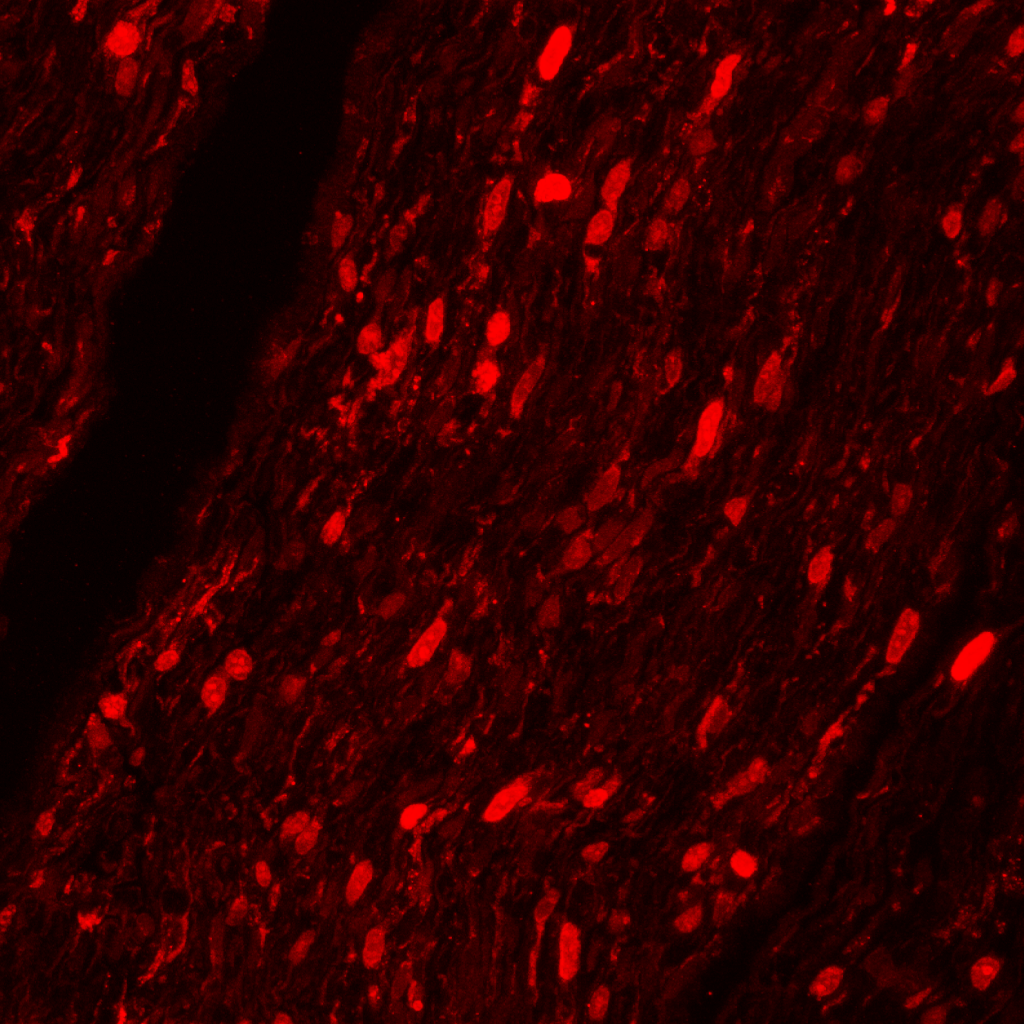

Supplement: Figure 3—source data 2. — This zip archive contains the IHC for one WT and one iDKO used for quantitative analysis shown in Figure 3H. Results and quantitation shown in the Figure used BD #550609 anti-Ki67. These results were confirmed using a second antibody, Abcam #ab15580 anti-Ki67. Images using both antibodies are included in the zip archive, in the indicated folders. Leica SP8 confocal lif images were processed using Imaris software and saved as tiffs. [file elife-50138-fig3-data2.zip › Figure 3 source data 2/iDKO #916 Ki67/BD #550609 Ki67/LHS a Ki67.tif]

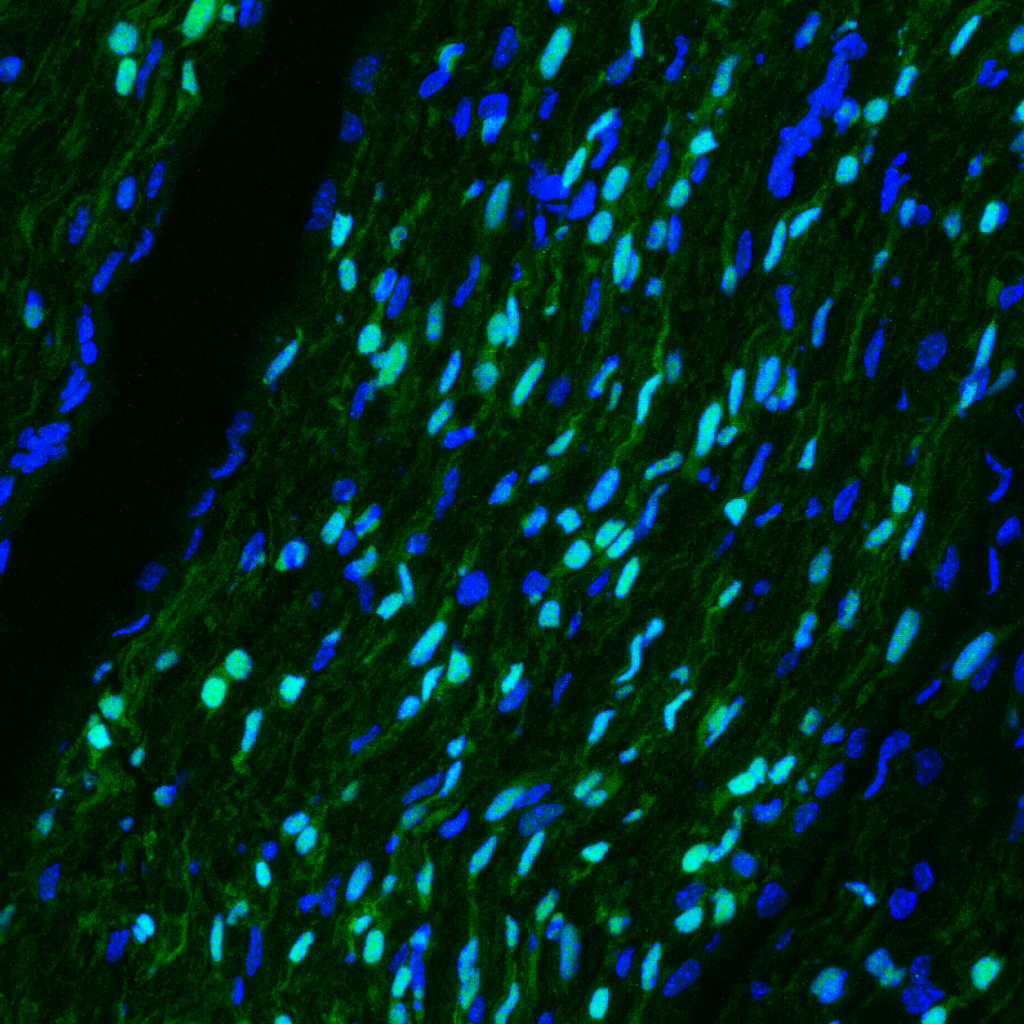

Supplement: Figure 3—source data 2. — This zip archive contains the IHC for one WT and one iDKO used for quantitative analysis shown in Figure 3H. Results and quantitation shown in the Figure used BD #550609 anti-Ki67. These results were confirmed using a second antibody, Abcam #ab15580 anti-Ki67. Images using both antibodies are included in the zip archive, in the indicated folders. Leica SP8 confocal lif images were processed using Imaris software and saved as tiffs. [file elife-50138-fig3-data2.zip › Figure 3 source data 2/iDKO #916 Ki67/BD #550609 Ki67/LHS a Sox10 + DAPI.tif]

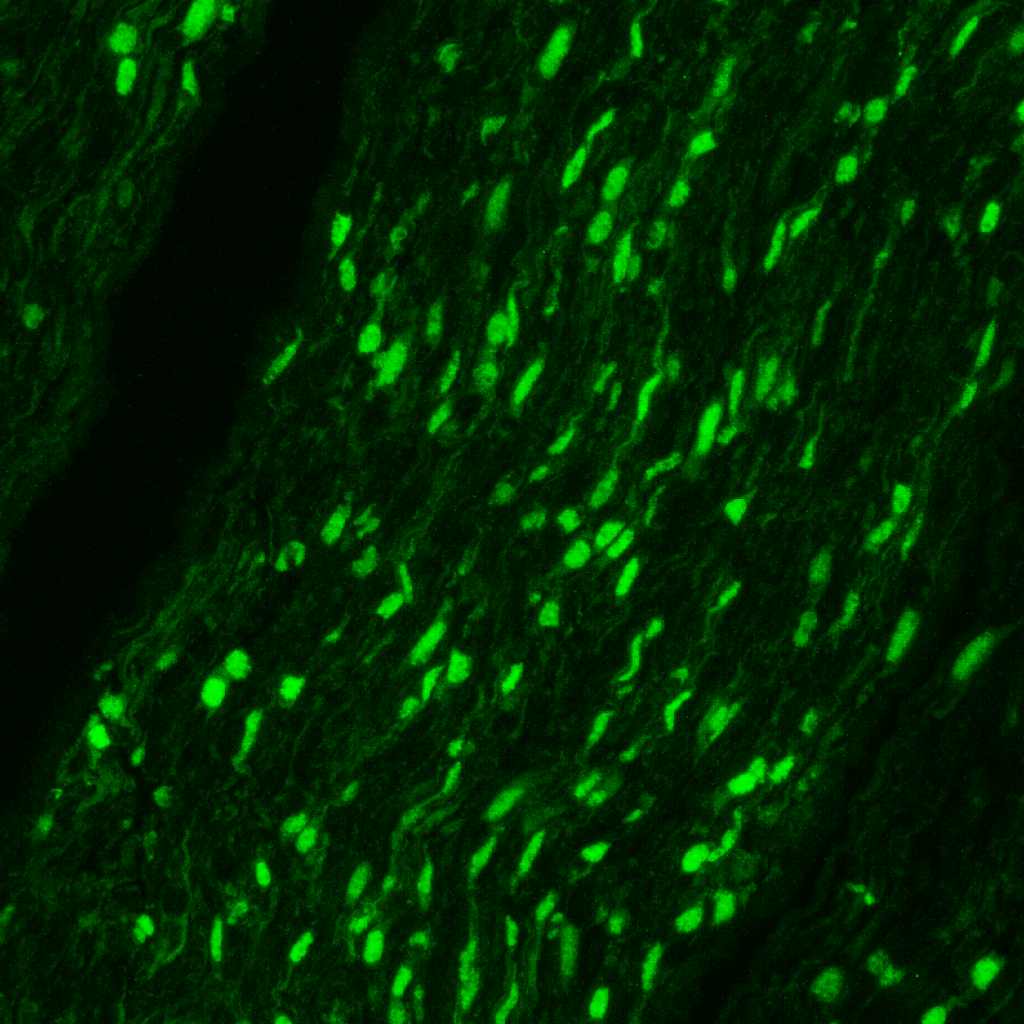

Supplement: Figure 3—source data 2. — This zip archive contains the IHC for one WT and one iDKO used for quantitative analysis shown in Figure 3H. Results and quantitation shown in the Figure used BD #550609 anti-Ki67. These results were confirmed using a second antibody, Abcam #ab15580 anti-Ki67. Images using both antibodies are included in the zip archive, in the indicated folders. Leica SP8 confocal lif images were processed using Imaris software and saved as tiffs. [file elife-50138-fig3-data2.zip › Figure 3 source data 2/iDKO #916 Ki67/BD #550609 Ki67/LHS a Sox10.tif]

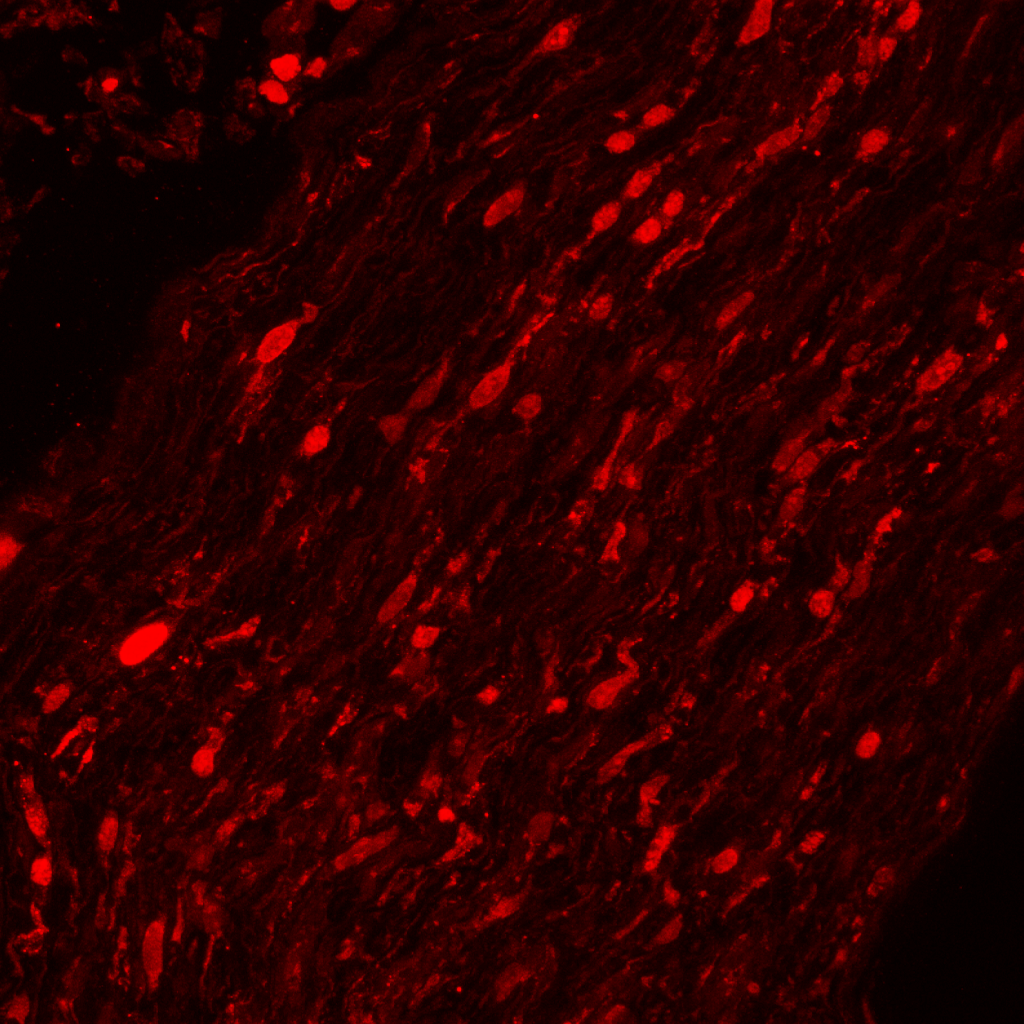

Supplement: Figure 3—source data 2. — This zip archive contains the IHC for one WT and one iDKO used for quantitative analysis shown in Figure 3H. Results and quantitation shown in the Figure used BD #550609 anti-Ki67. These results were confirmed using a second antibody, Abcam #ab15580 anti-Ki67. Images using both antibodies are included in the zip archive, in the indicated folders. Leica SP8 confocal lif images were processed using Imaris software and saved as tiffs. [file elife-50138-fig3-data2.zip › Figure 3 source data 2/iDKO #916 Ki67/BD #550609 Ki67/LHS b Ki67.tif]

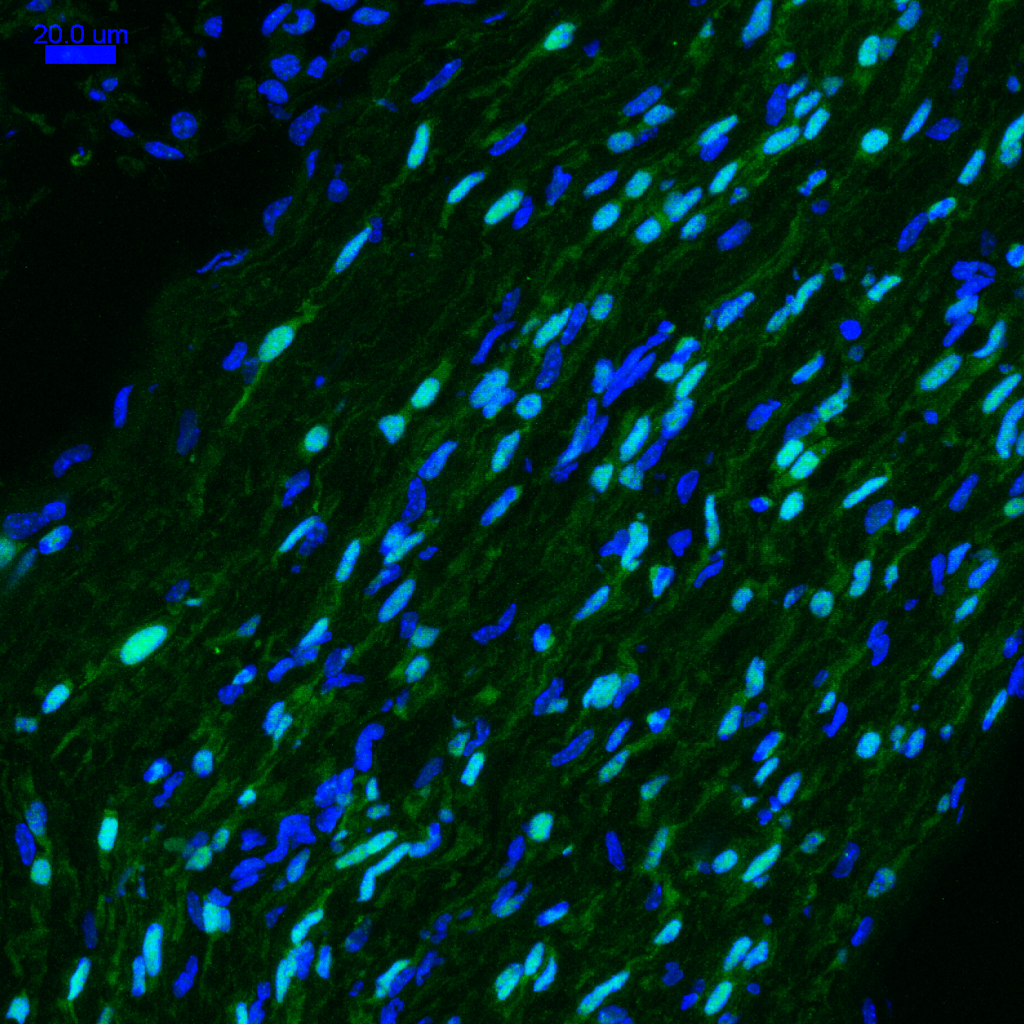

Supplement: Figure 3—source data 2. — This zip archive contains the IHC for one WT and one iDKO used for quantitative analysis shown in Figure 3H. Results and quantitation shown in the Figure used BD #550609 anti-Ki67. These results were confirmed using a second antibody, Abcam #ab15580 anti-Ki67. Images using both antibodies are included in the zip archive, in the indicated folders. Leica SP8 confocal lif images were processed using Imaris software and saved as tiffs. [file elife-50138-fig3-data2.zip › Figure 3 source data 2/iDKO #916 Ki67/BD #550609 Ki67/LHS b Sox10 + DAPI.tif]

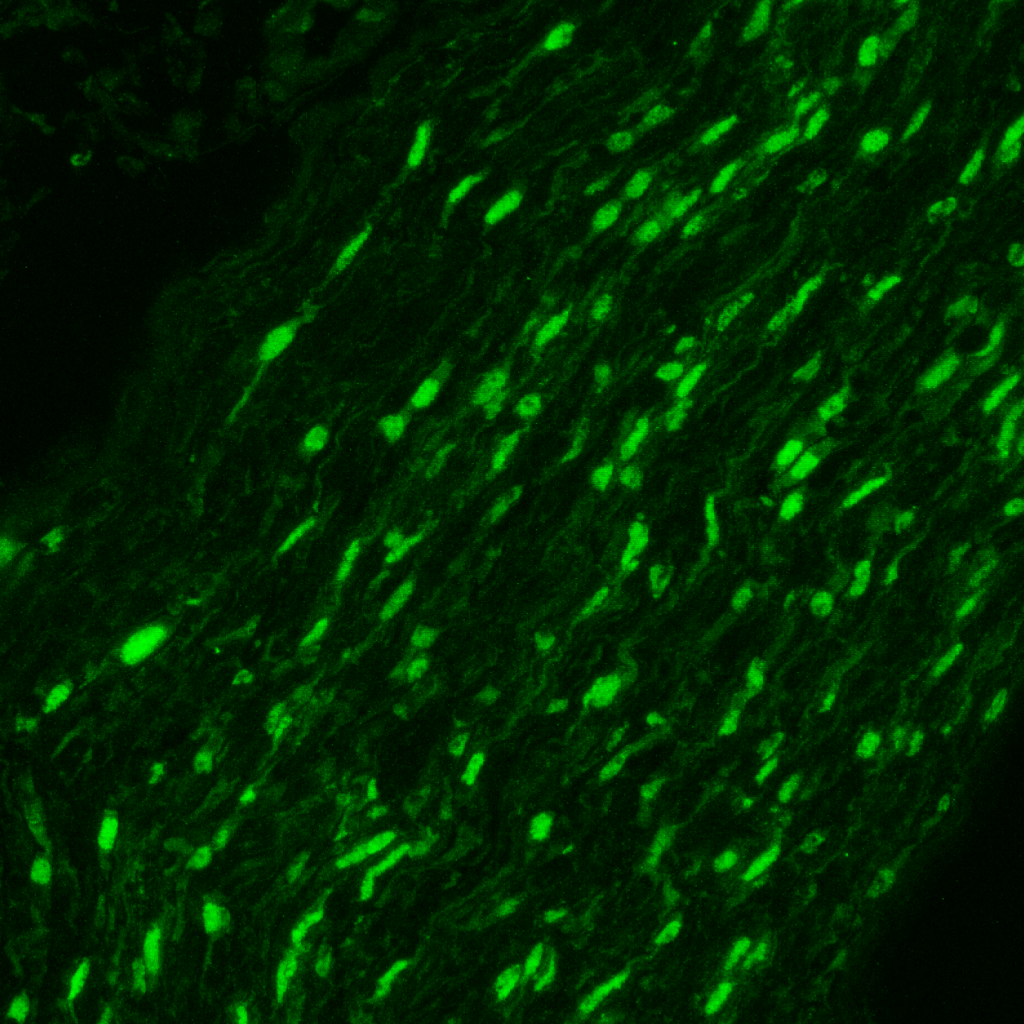

Supplement: Figure 3—source data 2. — This zip archive contains the IHC for one WT and one iDKO used for quantitative analysis shown in Figure 3H. Results and quantitation shown in the Figure used BD #550609 anti-Ki67. These results were confirmed using a second antibody, Abcam #ab15580 anti-Ki67. Images using both antibodies are included in the zip archive, in the indicated folders. Leica SP8 confocal lif images were processed using Imaris software and saved as tiffs. [file elife-50138-fig3-data2.zip › Figure 3 source data 2/iDKO #916 Ki67/BD #550609 Ki67/LHS b Sox10.tif]

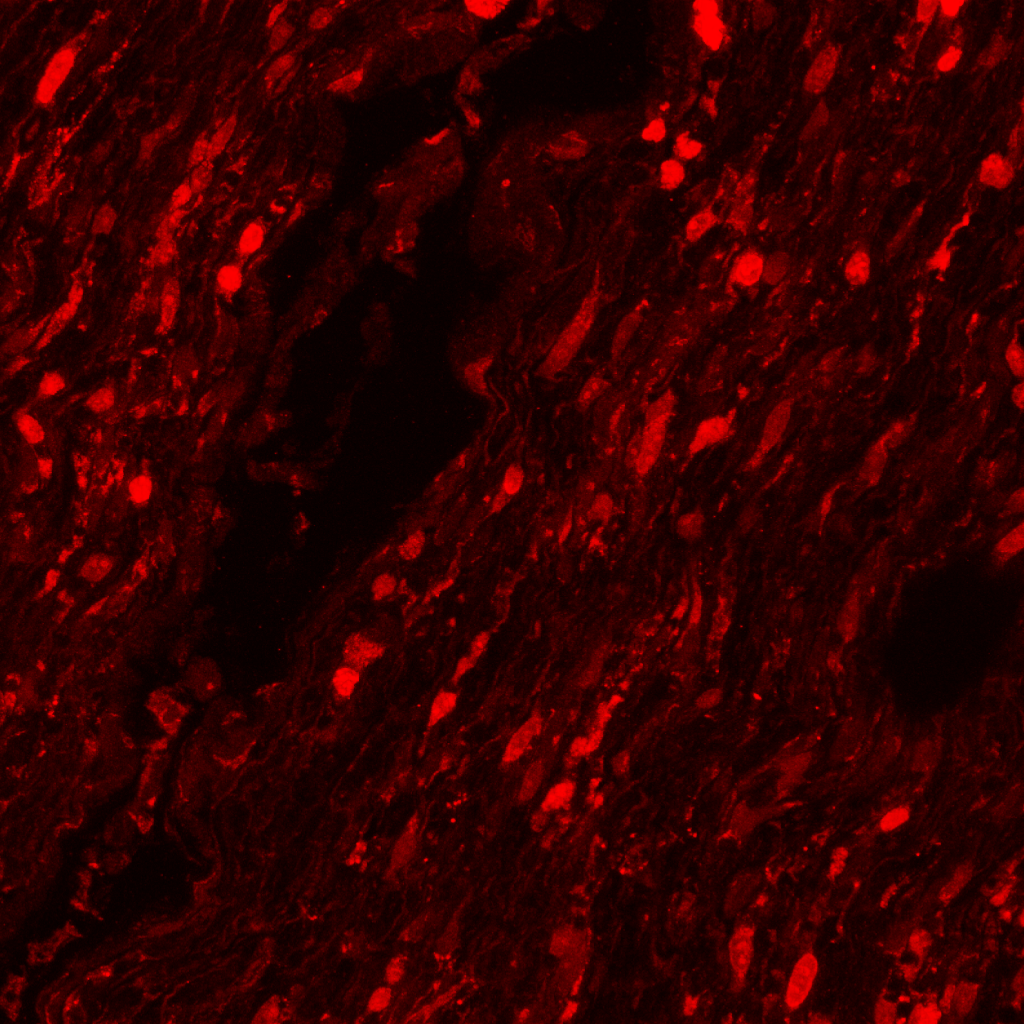

Supplement: Figure 3—source data 2. — This zip archive contains the IHC for one WT and one iDKO used for quantitative analysis shown in Figure 3H. Results and quantitation shown in the Figure used BD #550609 anti-Ki67. These results were confirmed using a second antibody, Abcam #ab15580 anti-Ki67. Images using both antibodies are included in the zip archive, in the indicated folders. Leica SP8 confocal lif images were processed using Imaris software and saved as tiffs. [file elife-50138-fig3-data2.zip › Figure 3 source data 2/iDKO #916 Ki67/BD #550609 Ki67/LHS c Ki67.tif]

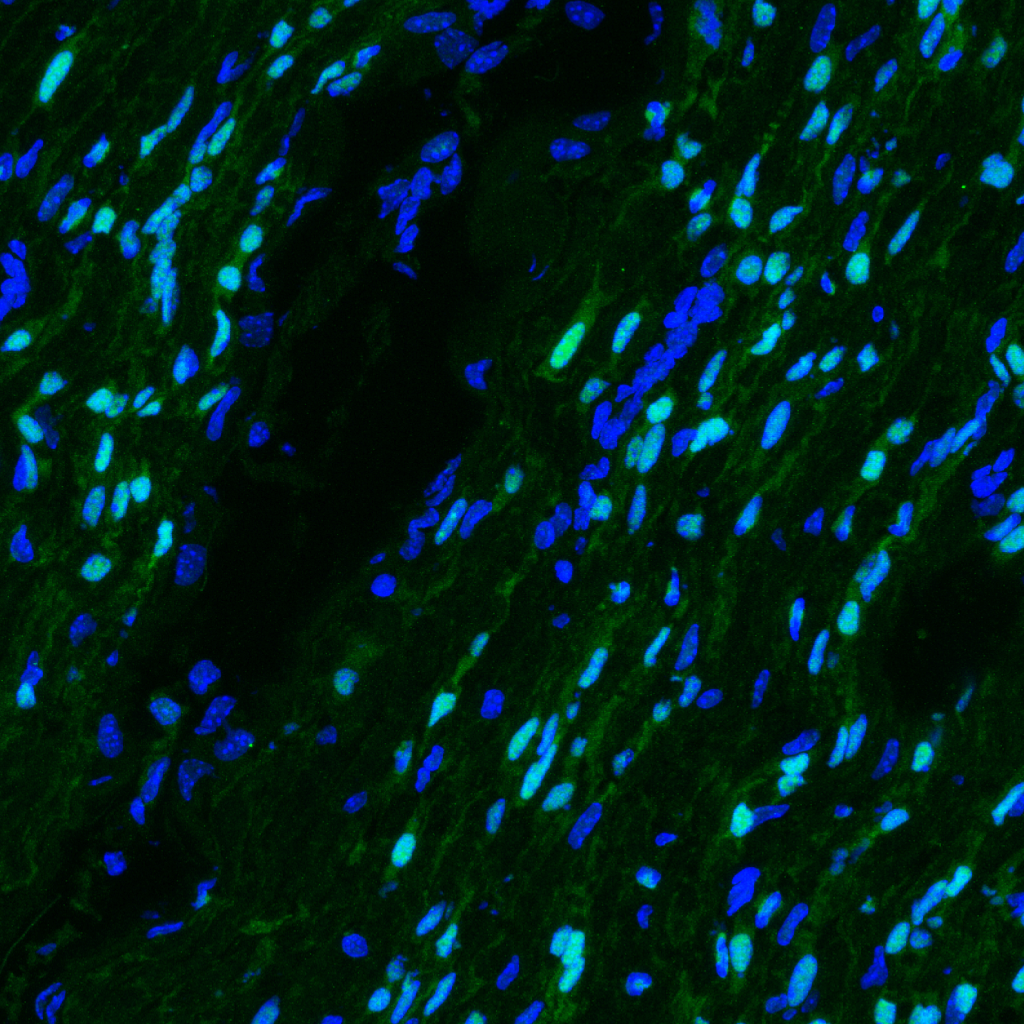

Supplement: Figure 3—source data 2. — This zip archive contains the IHC for one WT and one iDKO used for quantitative analysis shown in Figure 3H. Results and quantitation shown in the Figure used BD #550609 anti-Ki67. These results were confirmed using a second antibody, Abcam #ab15580 anti-Ki67. Images using both antibodies are included in the zip archive, in the indicated folders. Leica SP8 confocal lif images were processed using Imaris software and saved as tiffs. [file elife-50138-fig3-data2.zip › Figure 3 source data 2/iDKO #916 Ki67/BD #550609 Ki67/LHS c Sox10 + DAPI.tif]

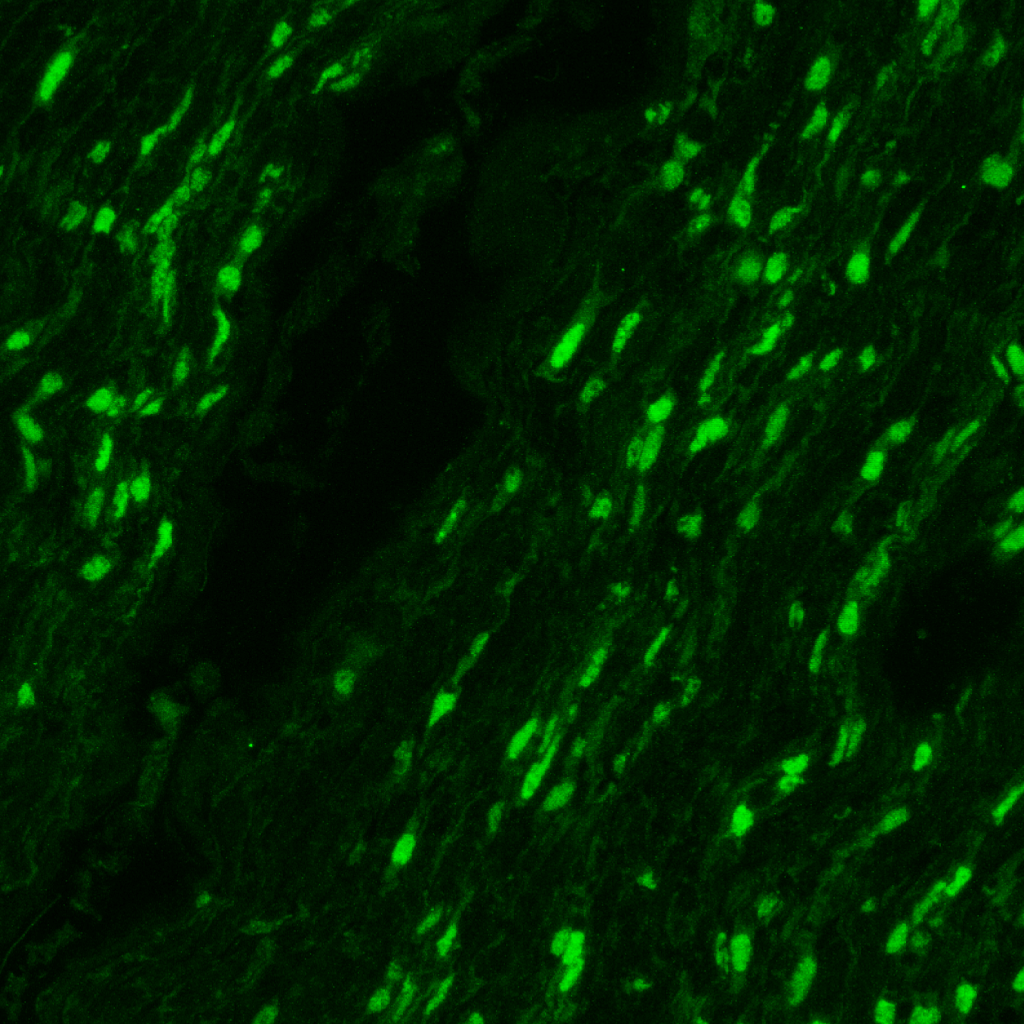

Supplement: Figure 3—source data 2. — This zip archive contains the IHC for one WT and one iDKO used for quantitative analysis shown in Figure 3H. Results and quantitation shown in the Figure used BD #550609 anti-Ki67. These results were confirmed using a second antibody, Abcam #ab15580 anti-Ki67. Images using both antibodies are included in the zip archive, in the indicated folders. Leica SP8 confocal lif images were processed using Imaris software and saved as tiffs. [file elife-50138-fig3-data2.zip › Figure 3 source data 2/iDKO #916 Ki67/BD #550609 Ki67/LHS c Sox10.tif]

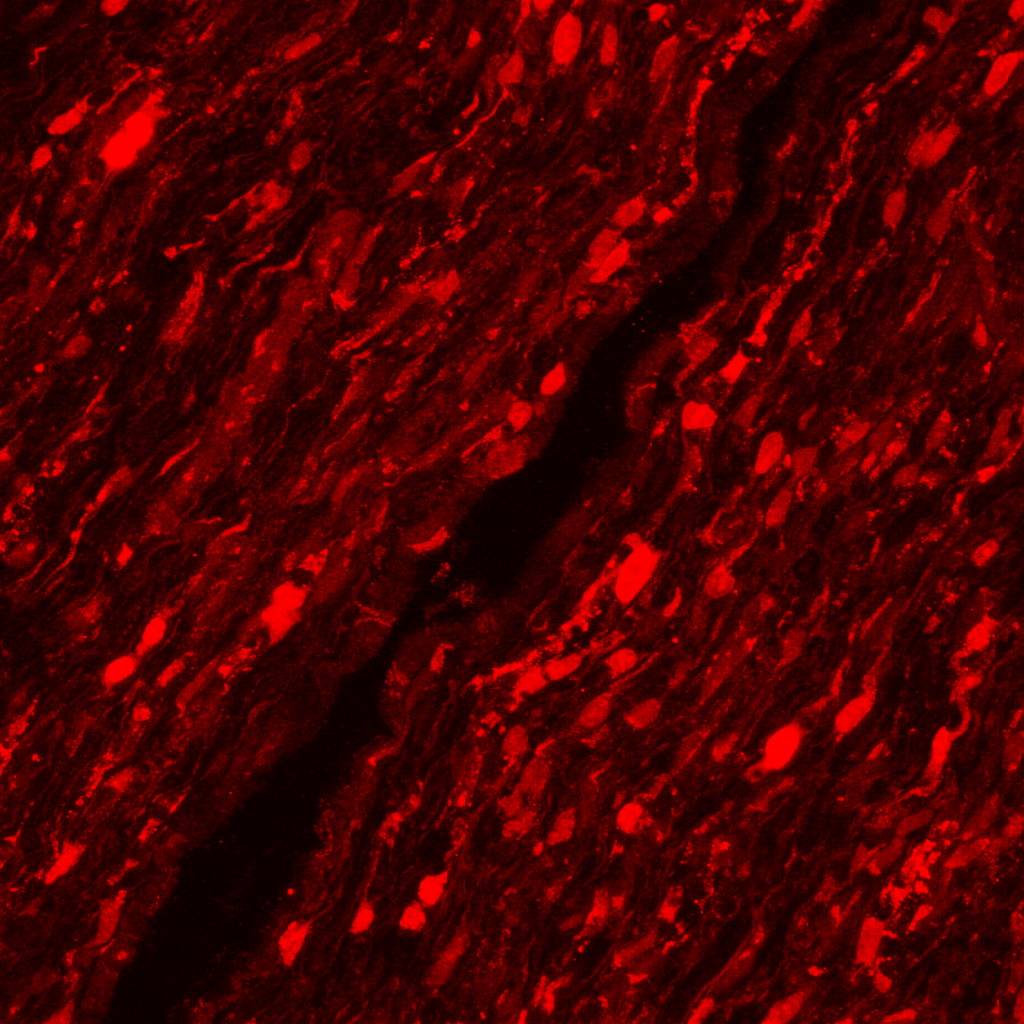

Supplement: Figure 3—source data 2. — This zip archive contains the IHC for one WT and one iDKO used for quantitative analysis shown in Figure 3H. Results and quantitation shown in the Figure used BD #550609 anti-Ki67. These results were confirmed using a second antibody, Abcam #ab15580 anti-Ki67. Images using both antibodies are included in the zip archive, in the indicated folders. Leica SP8 confocal lif images were processed using Imaris software and saved as tiffs. [file elife-50138-fig3-data2.zip › Figure 3 source data 2/iDKO #916 Ki67/BD #550609 Ki67/LHS d Ki67.tif]

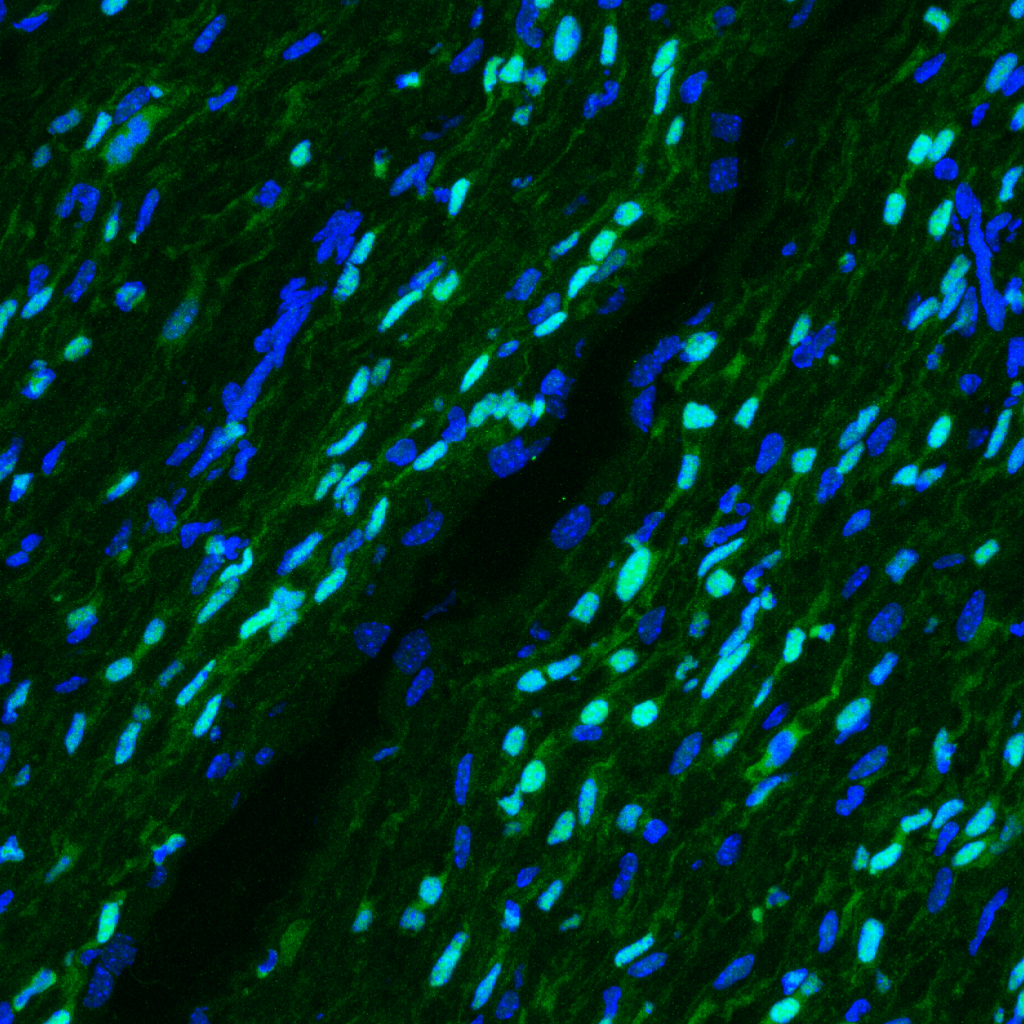

Supplement: Figure 3—source data 2. — This zip archive contains the IHC for one WT and one iDKO used for quantitative analysis shown in Figure 3H. Results and quantitation shown in the Figure used BD #550609 anti-Ki67. These results were confirmed using a second antibody, Abcam #ab15580 anti-Ki67. Images using both antibodies are included in the zip archive, in the indicated folders. Leica SP8 confocal lif images were processed using Imaris software and saved as tiffs. [file elife-50138-fig3-data2.zip › Figure 3 source data 2/iDKO #916 Ki67/BD #550609 Ki67/LHS d Sox10 + DAPI.tif]

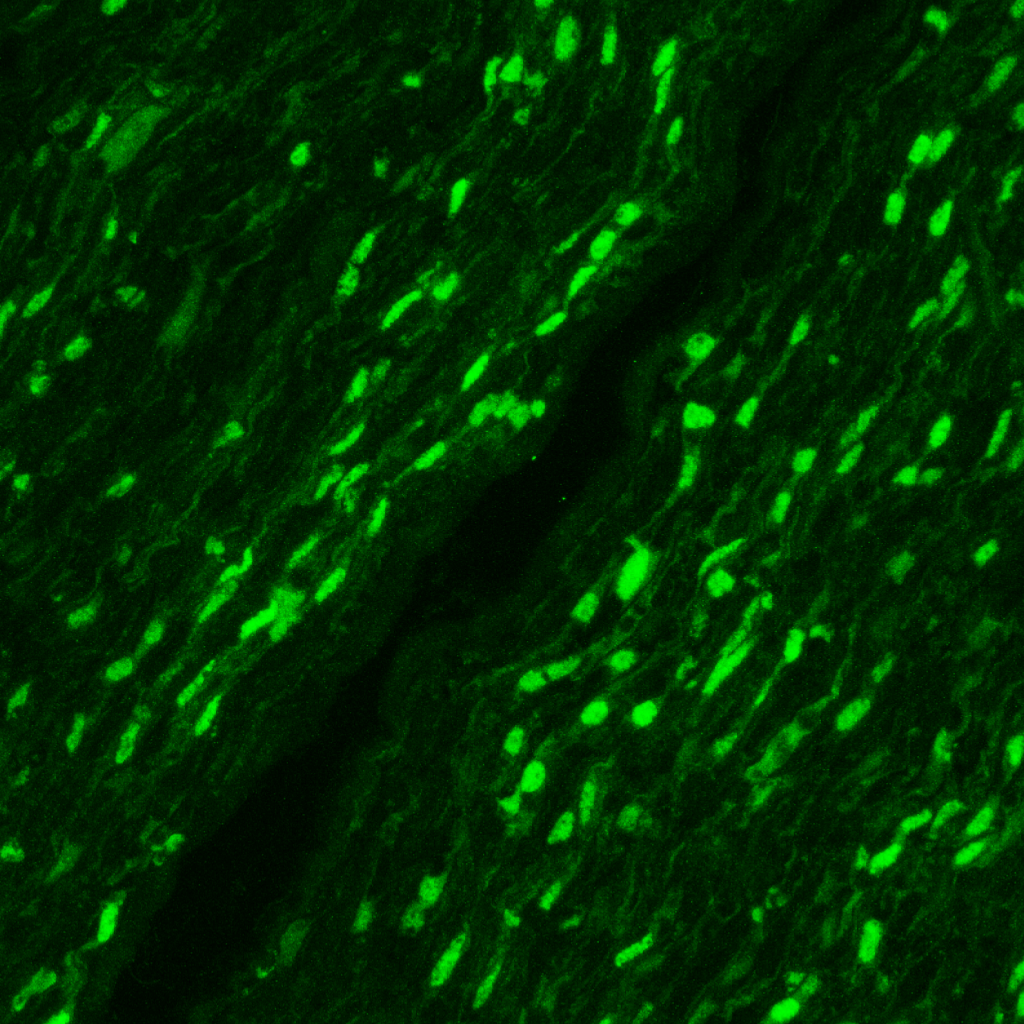

Supplement: Figure 3—source data 2. — This zip archive contains the IHC for one WT and one iDKO used for quantitative analysis shown in Figure 3H. Results and quantitation shown in the Figure used BD #550609 anti-Ki67. These results were confirmed using a second antibody, Abcam #ab15580 anti-Ki67. Images using both antibodies are included in the zip archive, in the indicated folders. Leica SP8 confocal lif images were processed using Imaris software and saved as tiffs. [file elife-50138-fig3-data2.zip › Figure 3 source data 2/iDKO #916 Ki67/BD #550609 Ki67/LHS d Sox10.tif]

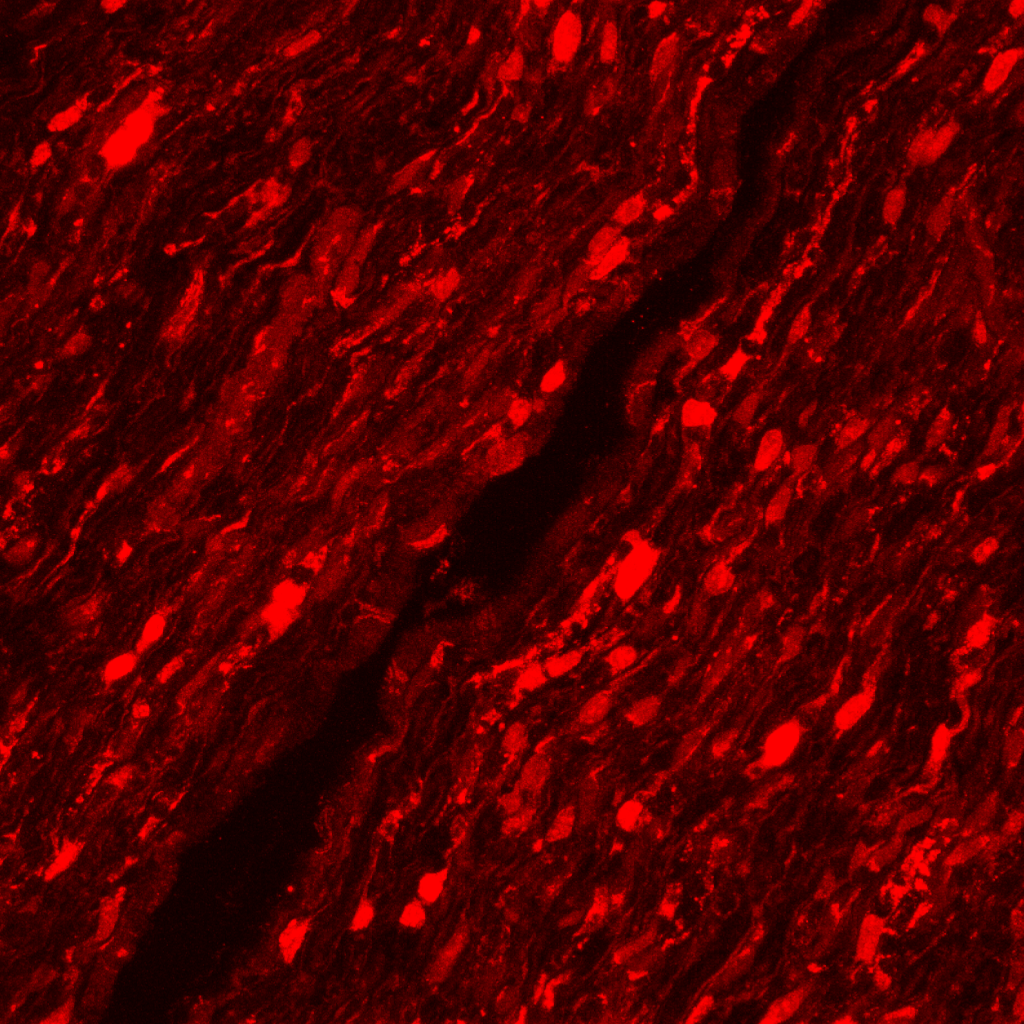

Supplement: Figure 3—source data 2. — This zip archive contains the IHC for one WT and one iDKO used for quantitative analysis shown in Figure 3H. Results and quantitation shown in the Figure used BD #550609 anti-Ki67. These results were confirmed using a second antibody, Abcam #ab15580 anti-Ki67. Images using both antibodies are included in the zip archive, in the indicated folders. Leica SP8 confocal lif images were processed using Imaris software and saved as tiffs. [file elife-50138-fig3-data2.zip › Figure 3 source data 2/iDKO #916 Ki67/BD #550609 Ki67/LHS e Ki67.tif]

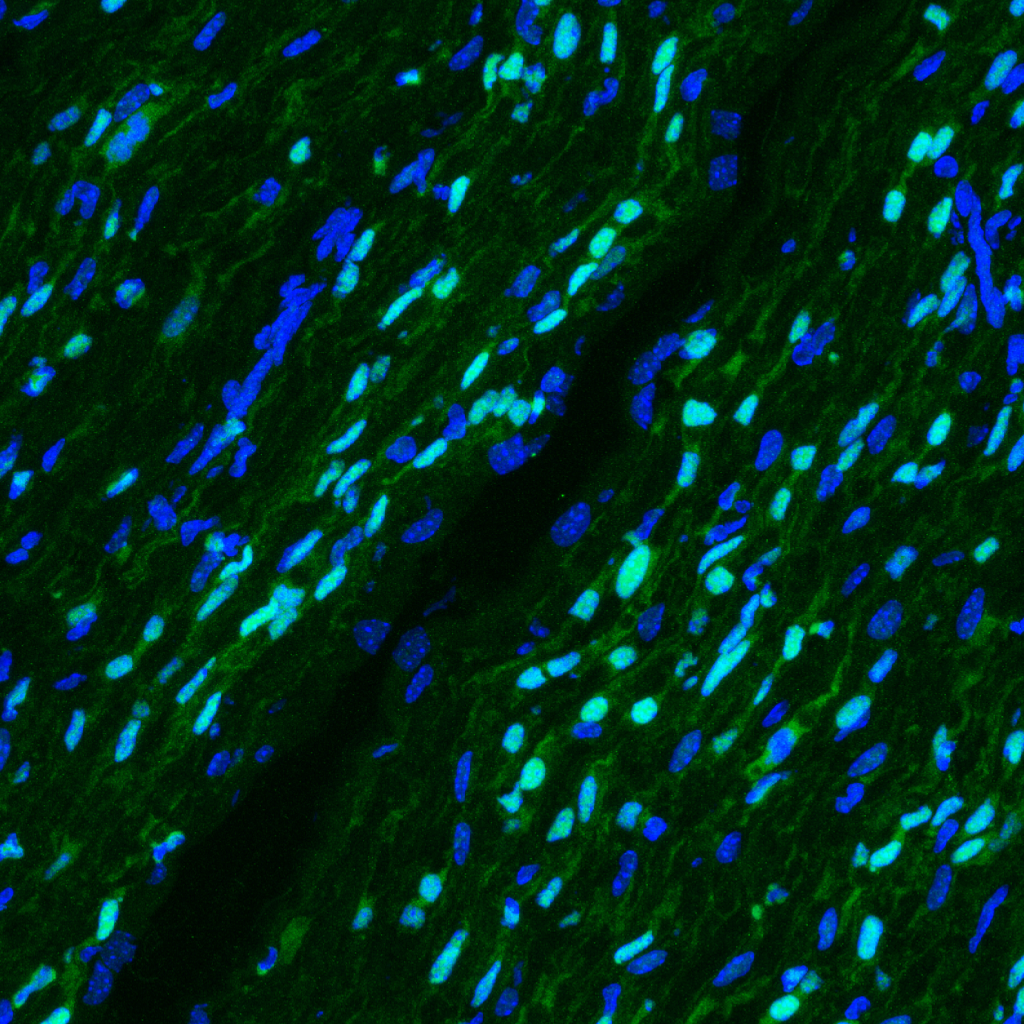

Supplement: Figure 3—source data 2. — This zip archive contains the IHC for one WT and one iDKO used for quantitative analysis shown in Figure 3H. Results and quantitation shown in the Figure used BD #550609 anti-Ki67. These results were confirmed using a second antibody, Abcam #ab15580 anti-Ki67. Images using both antibodies are included in the zip archive, in the indicated folders. Leica SP8 confocal lif images were processed using Imaris software and saved as tiffs. [file elife-50138-fig3-data2.zip › Figure 3 source data 2/iDKO #916 Ki67/BD #550609 Ki67/LHS e Sox10 + DAPI.tif]

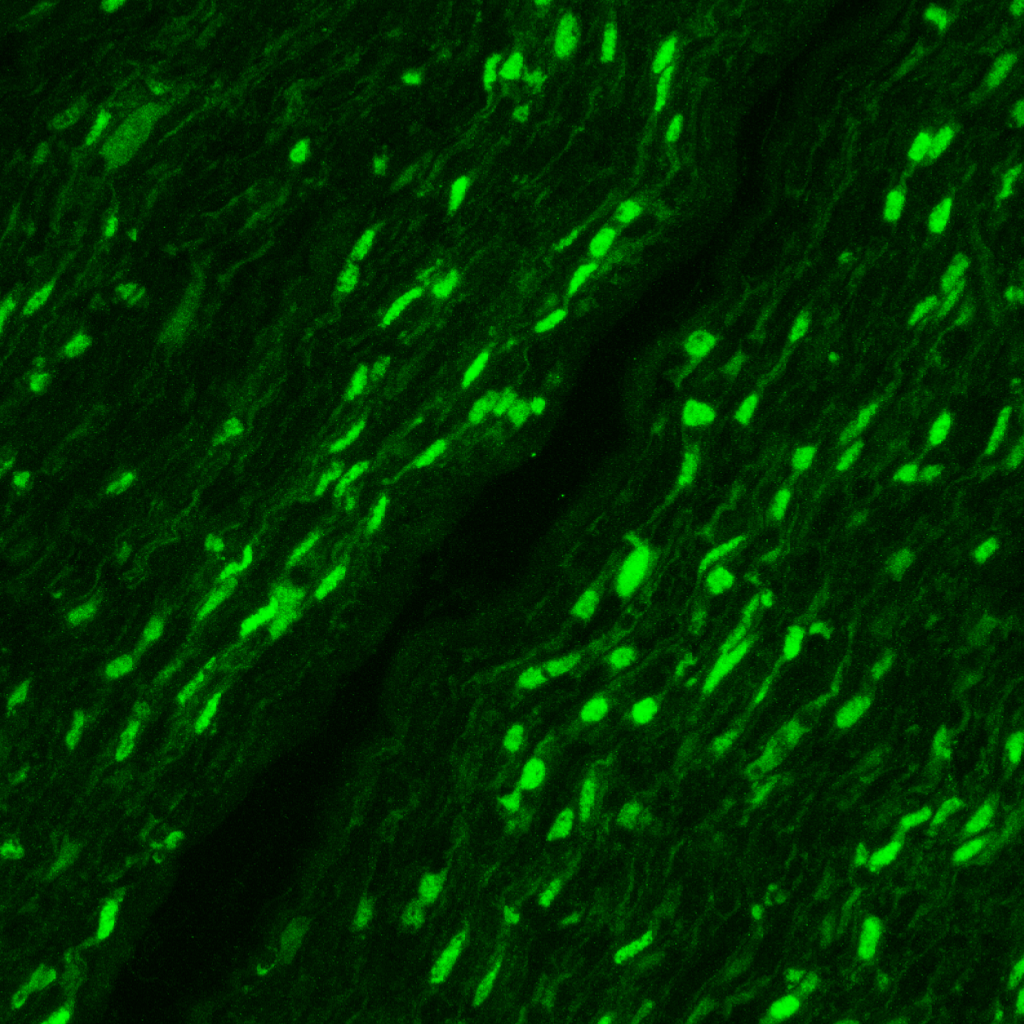

Supplement: Figure 3—source data 2. — This zip archive contains the IHC for one WT and one iDKO used for quantitative analysis shown in Figure 3H. Results and quantitation shown in the Figure used BD #550609 anti-Ki67. These results were confirmed using a second antibody, Abcam #ab15580 anti-Ki67. Images using both antibodies are included in the zip archive, in the indicated folders. Leica SP8 confocal lif images were processed using Imaris software and saved as tiffs. [file elife-50138-fig3-data2.zip › Figure 3 source data 2/iDKO #916 Ki67/BD #550609 Ki67/LHS e Sox10.tif]

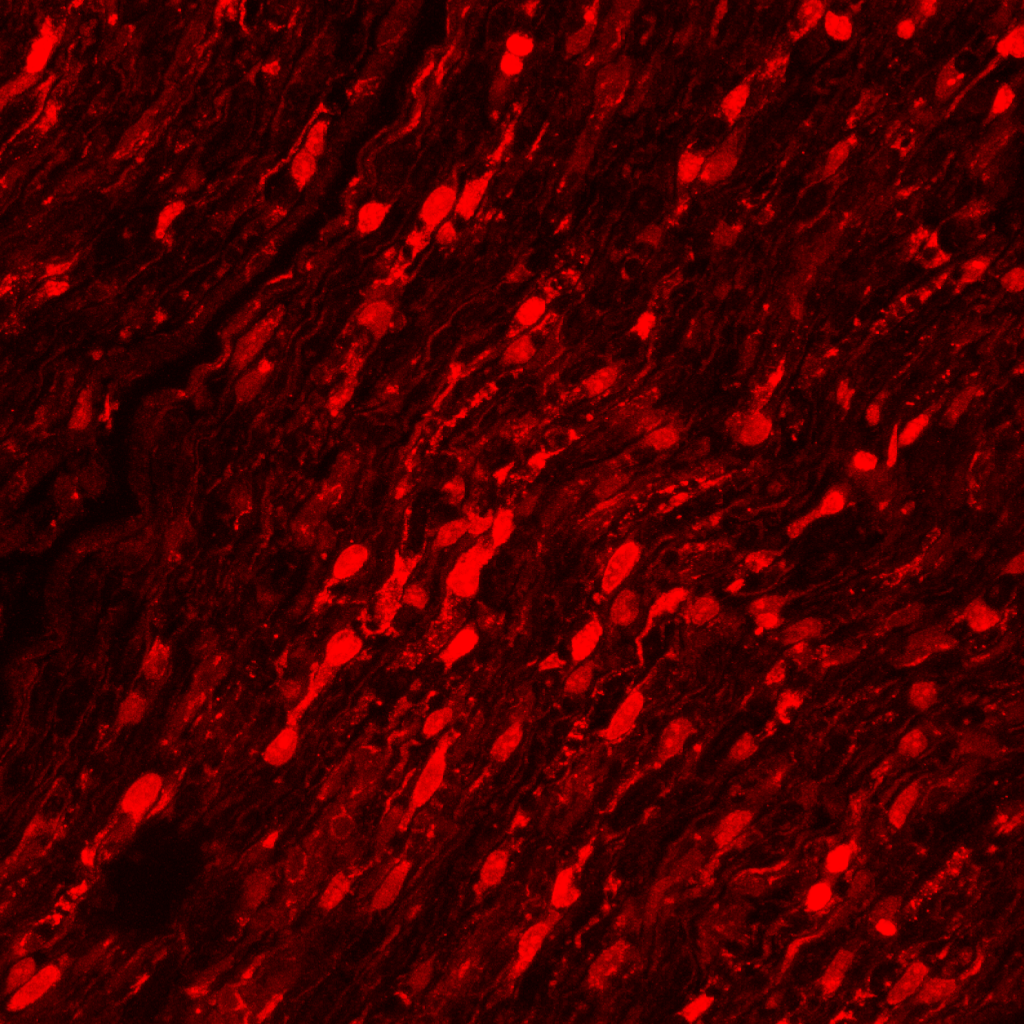

Supplement: Figure 3—source data 2. — This zip archive contains the IHC for one WT and one iDKO used for quantitative analysis shown in Figure 3H. Results and quantitation shown in the Figure used BD #550609 anti-Ki67. These results were confirmed using a second antibody, Abcam #ab15580 anti-Ki67. Images using both antibodies are included in the zip archive, in the indicated folders. Leica SP8 confocal lif images were processed using Imaris software and saved as tiffs. [file elife-50138-fig3-data2.zip › Figure 3 source data 2/iDKO #916 Ki67/BD #550609 Ki67/LHS f Ki67.tif]

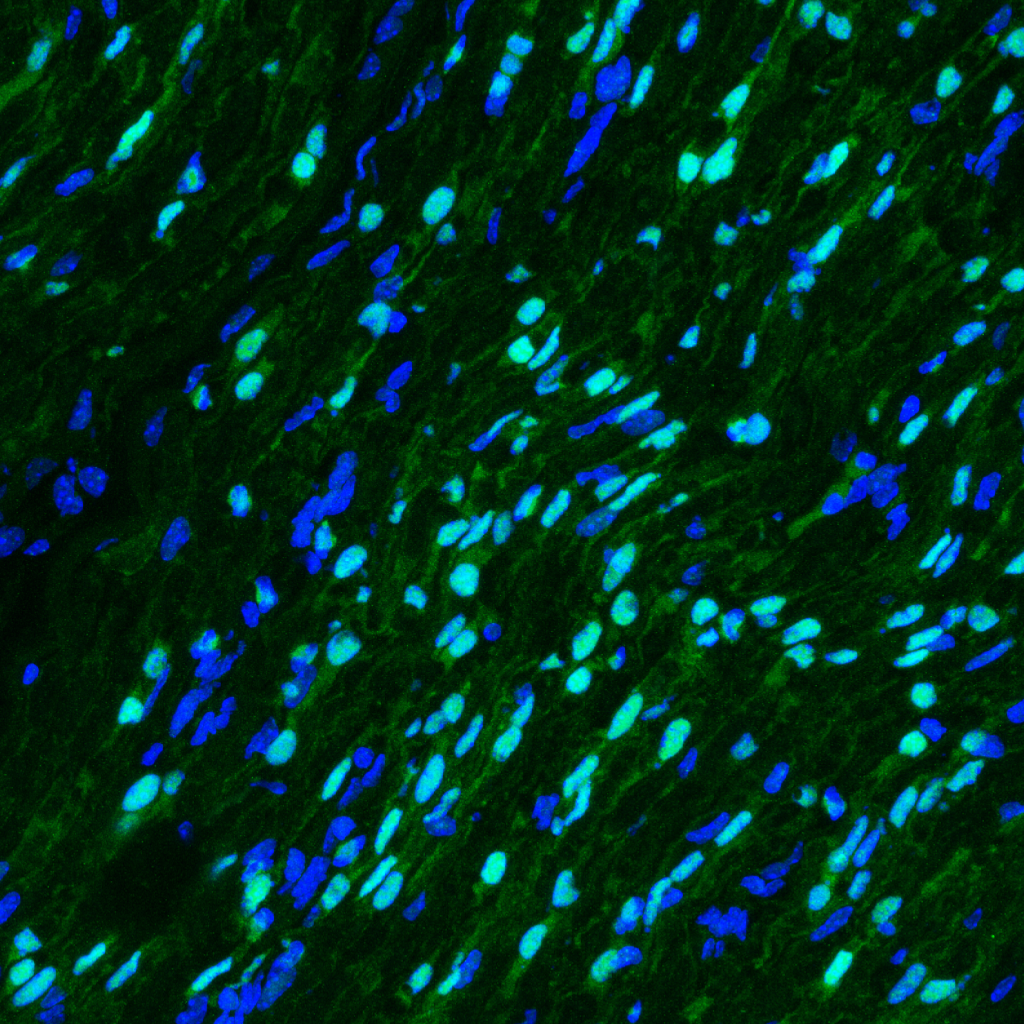

Supplement: Figure 3—source data 2. — This zip archive contains the IHC for one WT and one iDKO used for quantitative analysis shown in Figure 3H. Results and quantitation shown in the Figure used BD #550609 anti-Ki67. These results were confirmed using a second antibody, Abcam #ab15580 anti-Ki67. Images using both antibodies are included in the zip archive, in the indicated folders. Leica SP8 confocal lif images were processed using Imaris software and saved as tiffs. [file elife-50138-fig3-data2.zip › Figure 3 source data 2/iDKO #916 Ki67/BD #550609 Ki67/LHS f Sox10 + DAPI.tif]

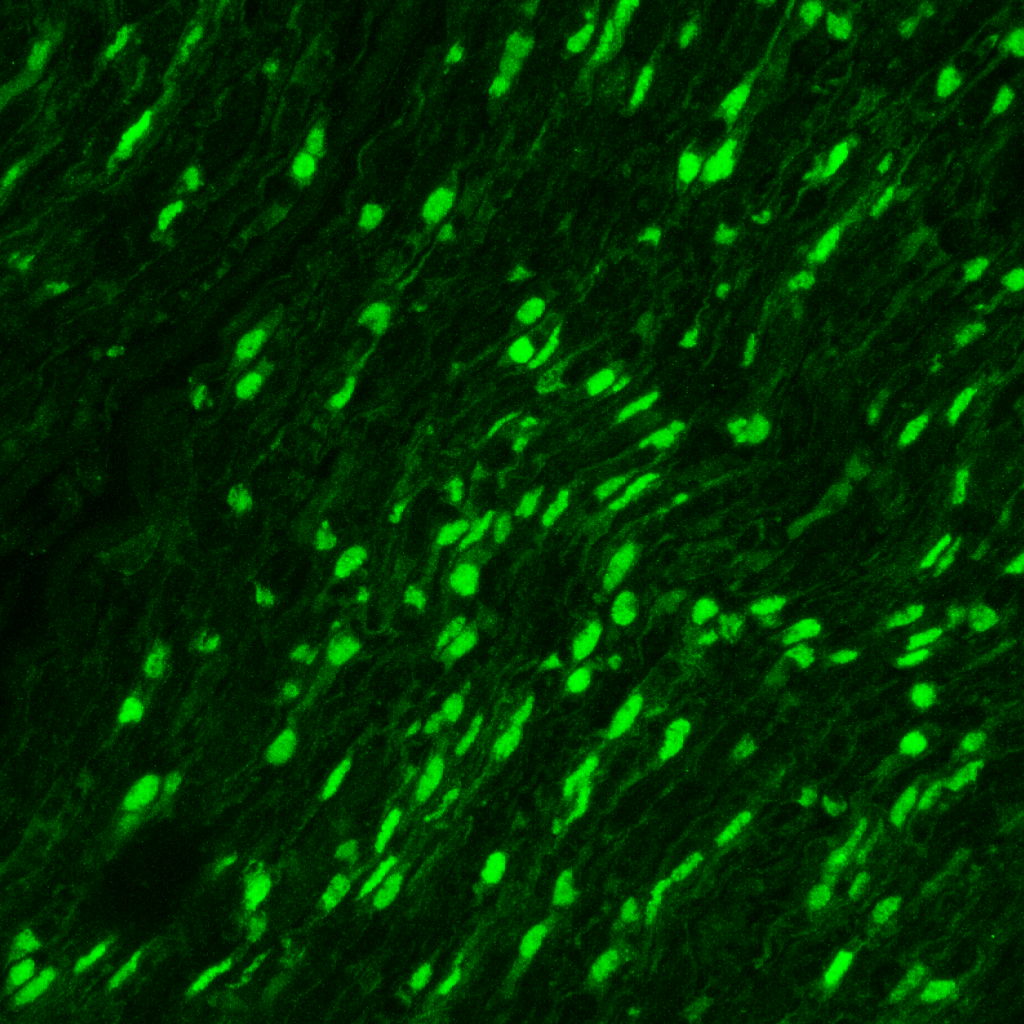

Supplement: Figure 3—source data 2. — This zip archive contains the IHC for one WT and one iDKO used for quantitative analysis shown in Figure 3H. Results and quantitation shown in the Figure used BD #550609 anti-Ki67. These results were confirmed using a second antibody, Abcam #ab15580 anti-Ki67. Images using both antibodies are included in the zip archive, in the indicated folders. Leica SP8 confocal lif images were processed using Imaris software and saved as tiffs. [file elife-50138-fig3-data2.zip › Figure 3 source data 2/iDKO #916 Ki67/BD #550609 Ki67/LHS f Sox10.tif]

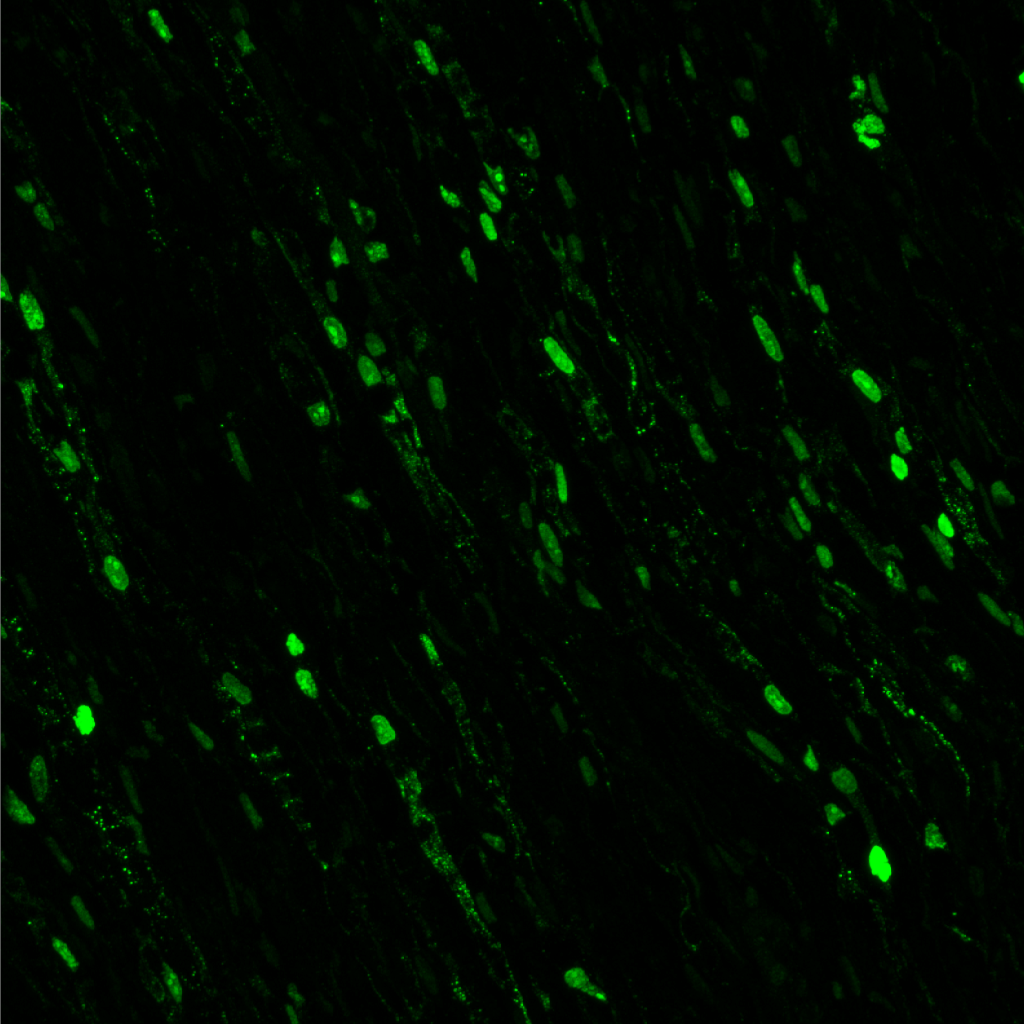

Supplement: Figure 3—source data 2. — This zip archive contains the IHC for one WT and one iDKO used for quantitative analysis shown in Figure 3H. Results and quantitation shown in the Figure used BD #550609 anti-Ki67. These results were confirmed using a second antibody, Abcam #ab15580 anti-Ki67. Images using both antibodies are included in the zip archive, in the indicated folders. Leica SP8 confocal lif images were processed using Imaris software and saved as tiffs. [file elife-50138-fig3-data2.zip › Figure 3 source data 2/WT #943 Ki67/Abcam #ab15580 Ki67/Series 16 Ki67.tif]

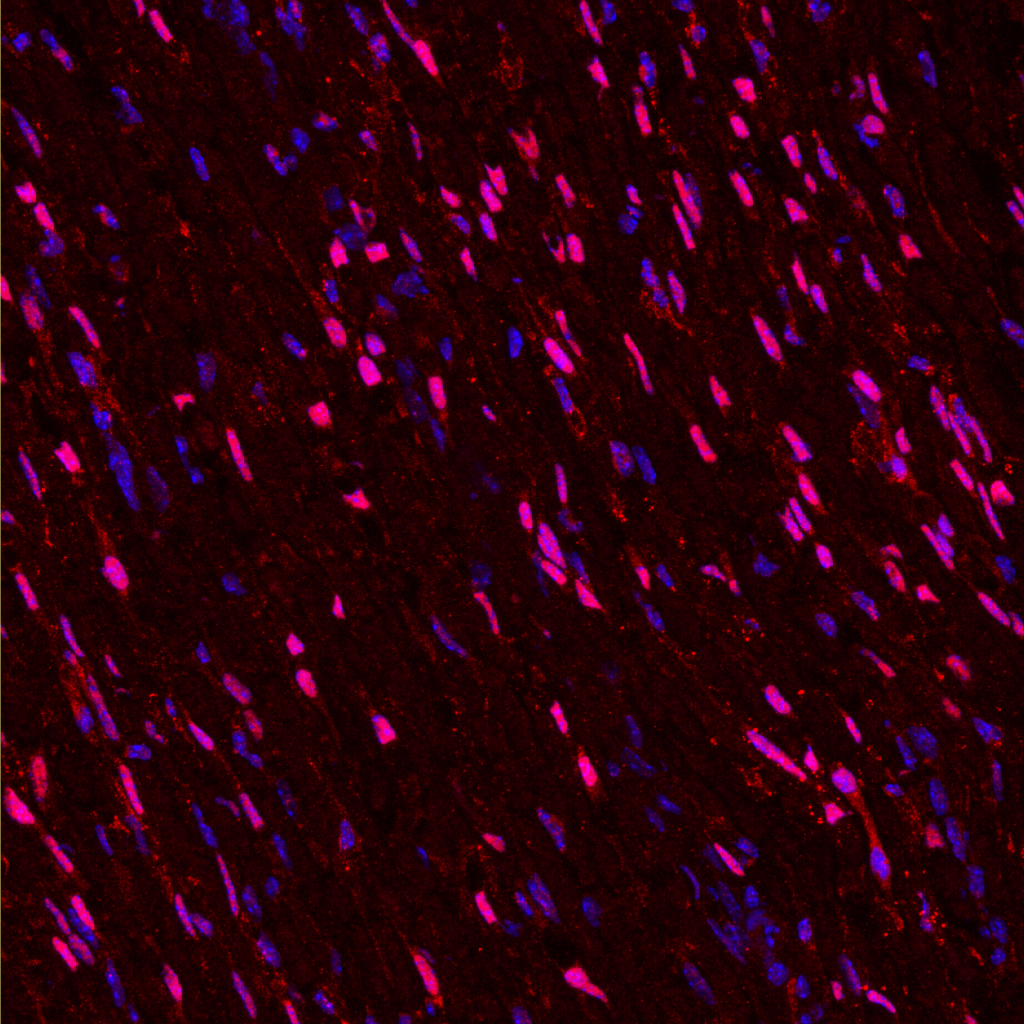

Supplement: Figure 3—source data 2. — This zip archive contains the IHC for one WT and one iDKO used for quantitative analysis shown in Figure 3H. Results and quantitation shown in the Figure used BD #550609 anti-Ki67. These results were confirmed using a second antibody, Abcam #ab15580 anti-Ki67. Images using both antibodies are included in the zip archive, in the indicated folders. Leica SP8 confocal lif images were processed using Imaris software and saved as tiffs. [file elife-50138-fig3-data2.zip › Figure 3 source data 2/WT #943 Ki67/Abcam #ab15580 Ki67/Series 16 Sox10 + DAPI.tif]

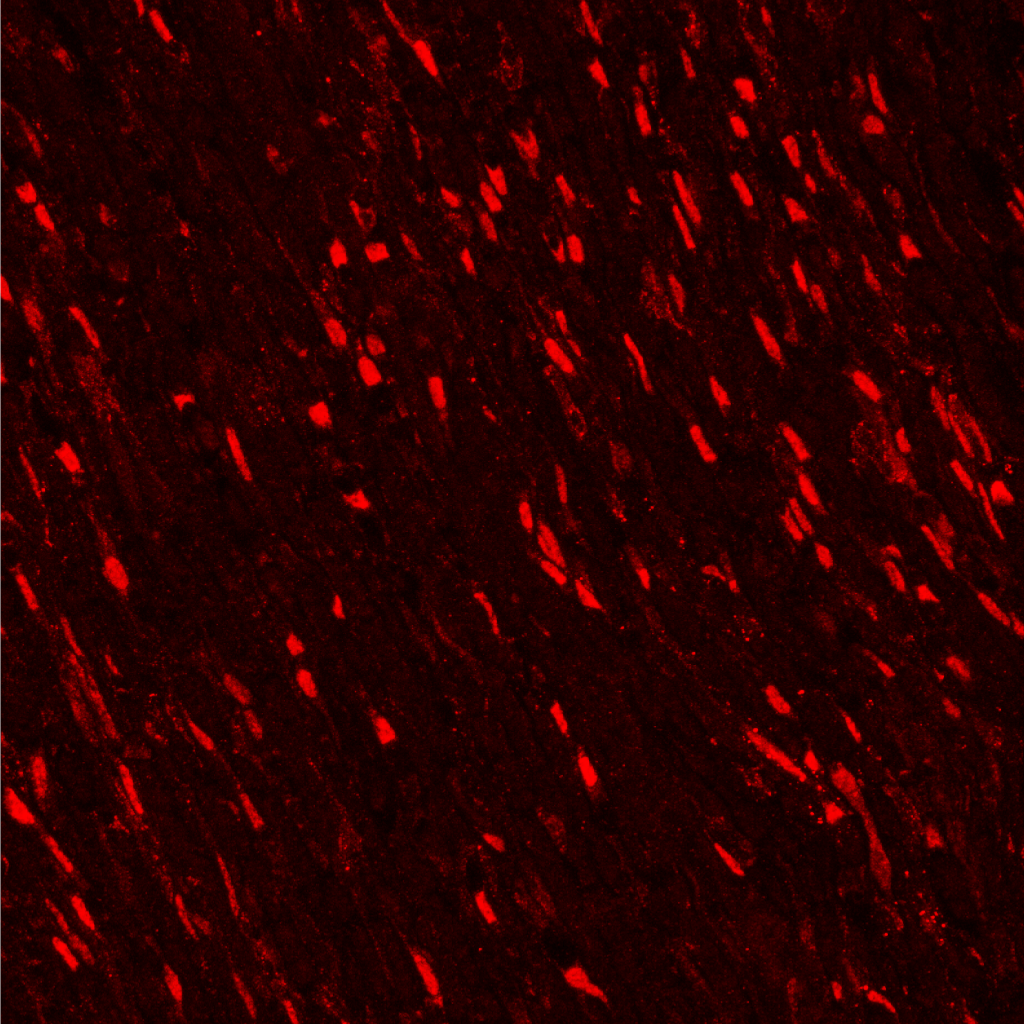

Supplement: Figure 3—source data 2. — This zip archive contains the IHC for one WT and one iDKO used for quantitative analysis shown in Figure 3H. Results and quantitation shown in the Figure used BD #550609 anti-Ki67. These results were confirmed using a second antibody, Abcam #ab15580 anti-Ki67. Images using both antibodies are included in the zip archive, in the indicated folders. Leica SP8 confocal lif images were processed using Imaris software and saved as tiffs. [file elife-50138-fig3-data2.zip › Figure 3 source data 2/WT #943 Ki67/Abcam #ab15580 Ki67/Series 16 Sox10.tif]

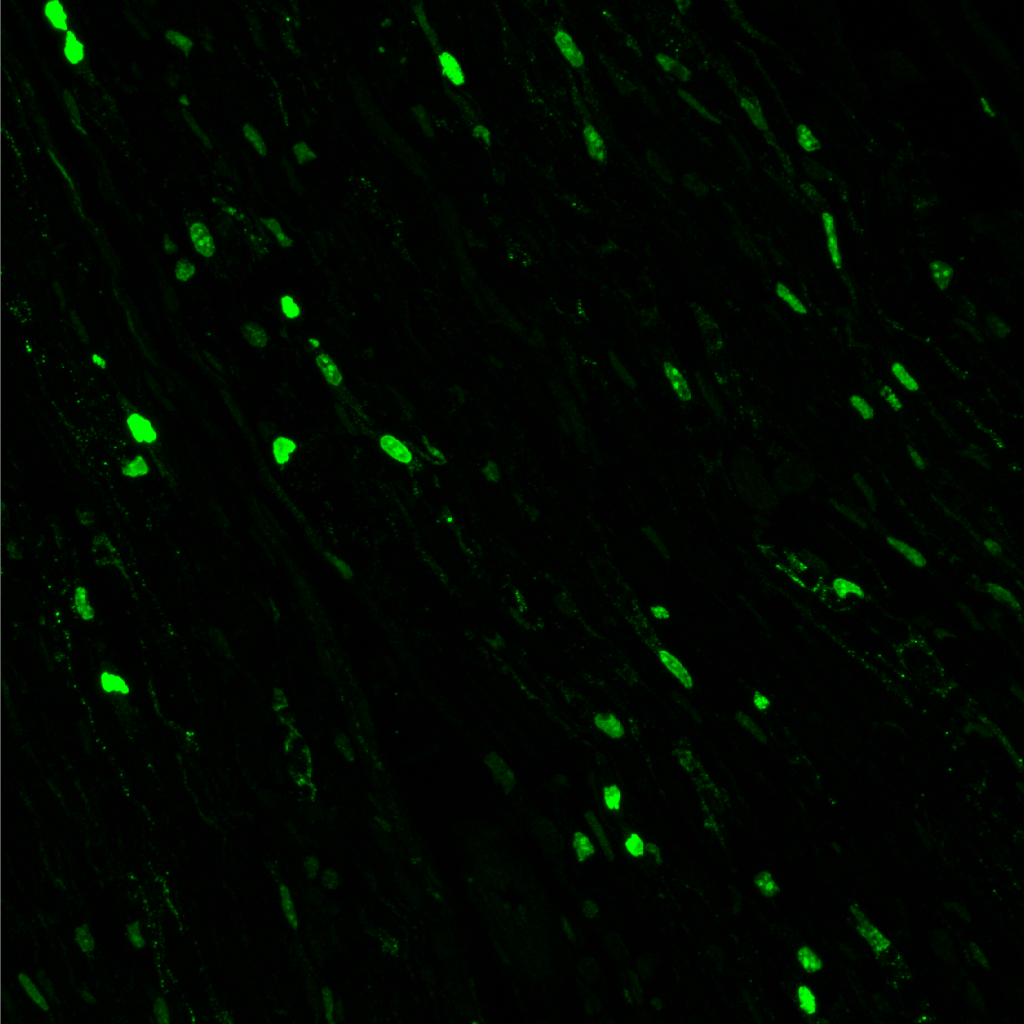

Supplement: Figure 3—source data 2. — This zip archive contains the IHC for one WT and one iDKO used for quantitative analysis shown in Figure 3H. Results and quantitation shown in the Figure used BD #550609 anti-Ki67. These results were confirmed using a second antibody, Abcam #ab15580 anti-Ki67. Images using both antibodies are included in the zip archive, in the indicated folders. Leica SP8 confocal lif images were processed using Imaris software and saved as tiffs. [file elife-50138-fig3-data2.zip › Figure 3 source data 2/WT #943 Ki67/Abcam #ab15580 Ki67/Series 27 Ki67.tif]

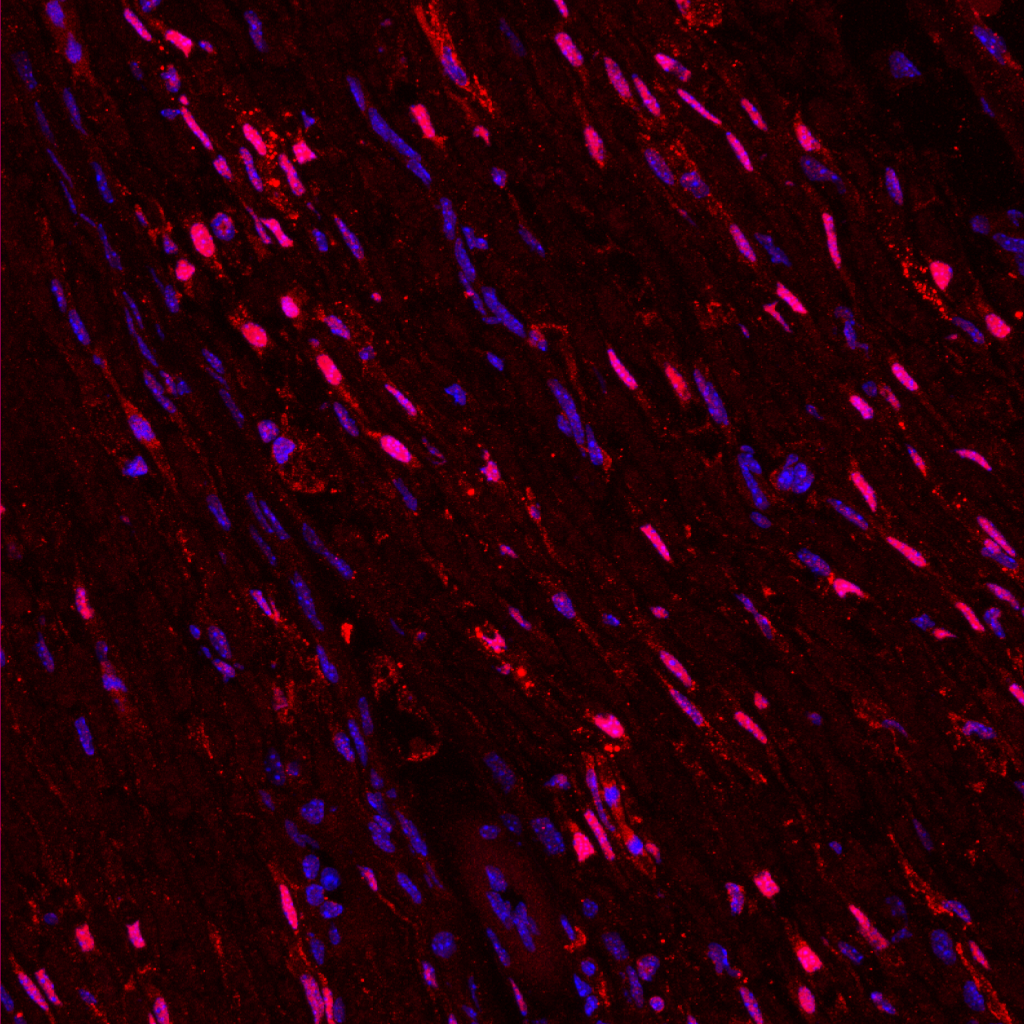

Supplement: Figure 3—source data 2. — This zip archive contains the IHC for one WT and one iDKO used for quantitative analysis shown in Figure 3H. Results and quantitation shown in the Figure used BD #550609 anti-Ki67. These results were confirmed using a second antibody, Abcam #ab15580 anti-Ki67. Images using both antibodies are included in the zip archive, in the indicated folders. Leica SP8 confocal lif images were processed using Imaris software and saved as tiffs. [file elife-50138-fig3-data2.zip › Figure 3 source data 2/WT #943 Ki67/Abcam #ab15580 Ki67/Series 27 Sox10 + DAPI.tif]

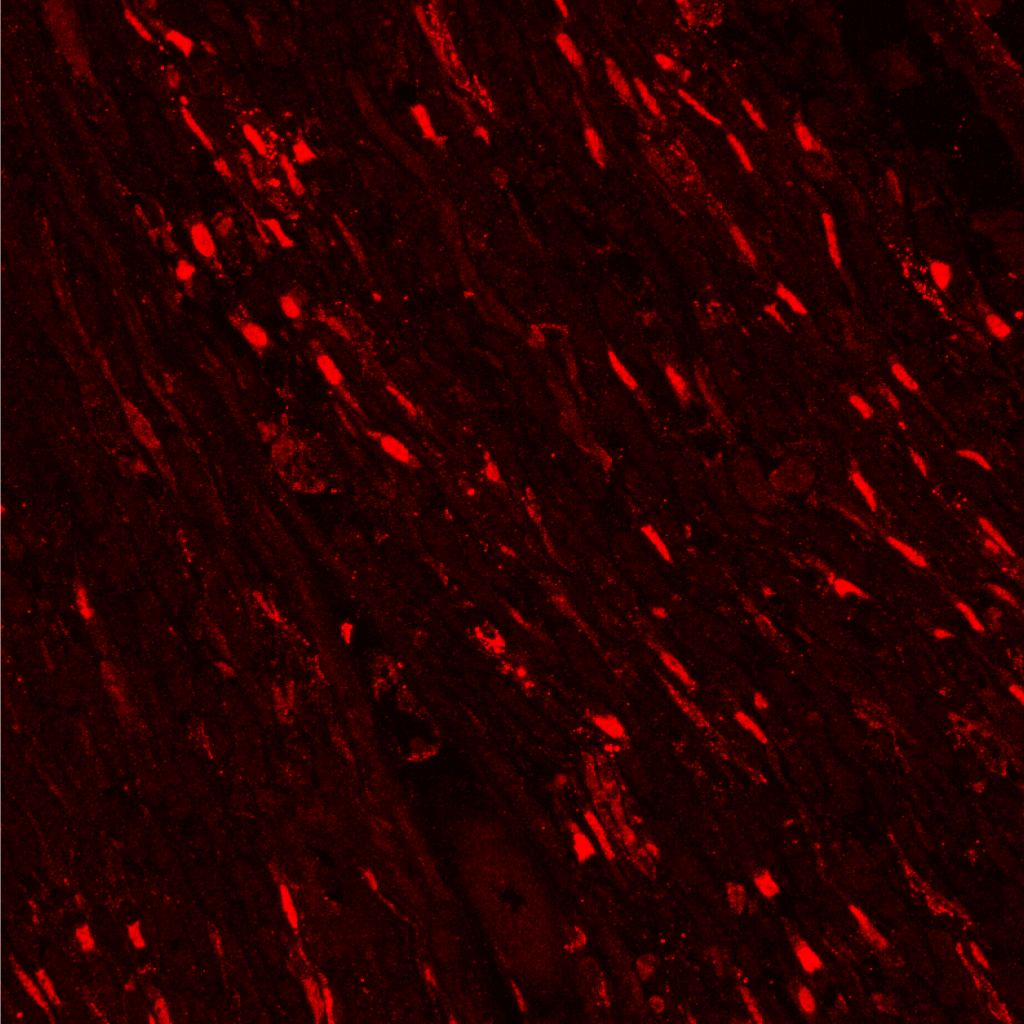

Supplement: Figure 3—source data 2. — This zip archive contains the IHC for one WT and one iDKO used for quantitative analysis shown in Figure 3H. Results and quantitation shown in the Figure used BD #550609 anti-Ki67. These results were confirmed using a second antibody, Abcam #ab15580 anti-Ki67. Images using both antibodies are included in the zip archive, in the indicated folders. Leica SP8 confocal lif images were processed using Imaris software and saved as tiffs. [file elife-50138-fig3-data2.zip › Figure 3 source data 2/WT #943 Ki67/Abcam #ab15580 Ki67/Series 27 Sox10.tif]

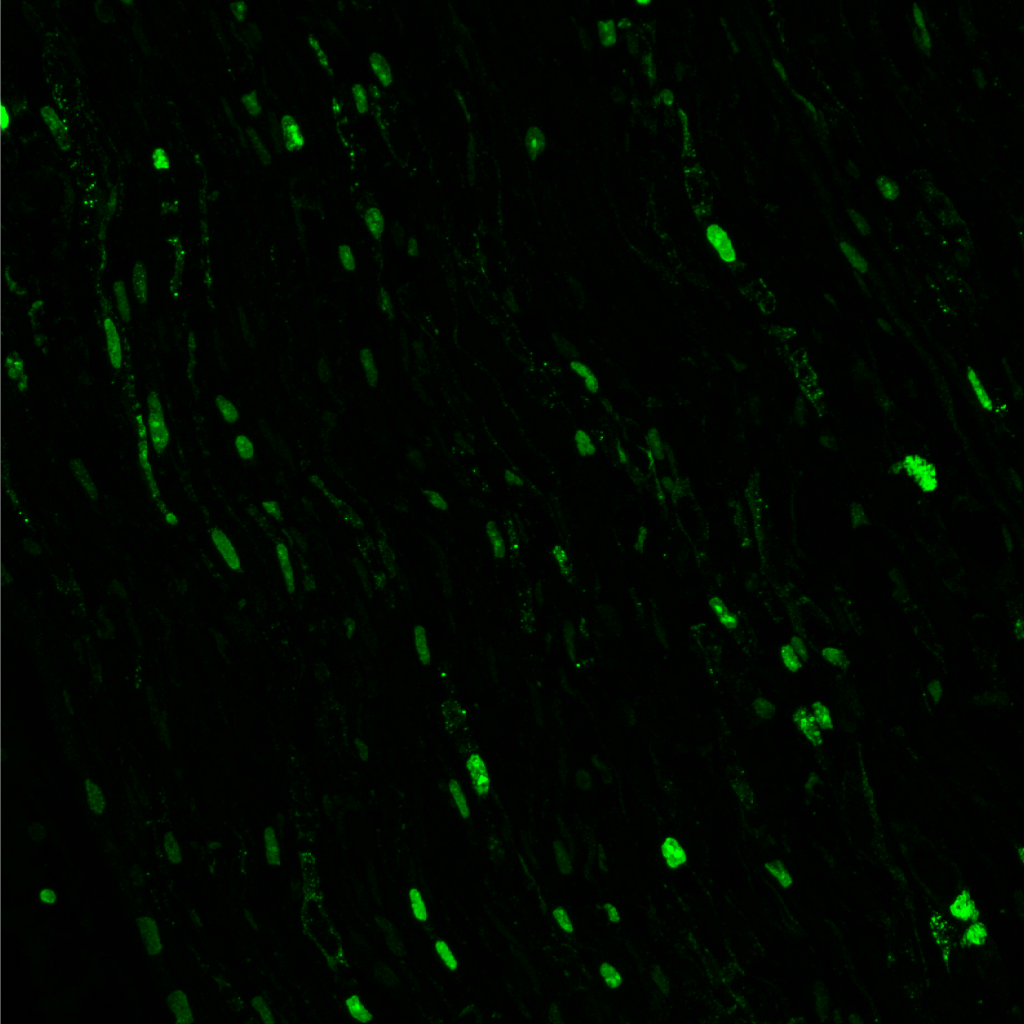

Supplement: Figure 3—source data 2. — This zip archive contains the IHC for one WT and one iDKO used for quantitative analysis shown in Figure 3H. Results and quantitation shown in the Figure used BD #550609 anti-Ki67. These results were confirmed using a second antibody, Abcam #ab15580 anti-Ki67. Images using both antibodies are included in the zip archive, in the indicated folders. Leica SP8 confocal lif images were processed using Imaris software and saved as tiffs. [file elife-50138-fig3-data2.zip › Figure 3 source data 2/WT #943 Ki67/Abcam #ab15580 Ki67/Series 35 Ki67.tif]

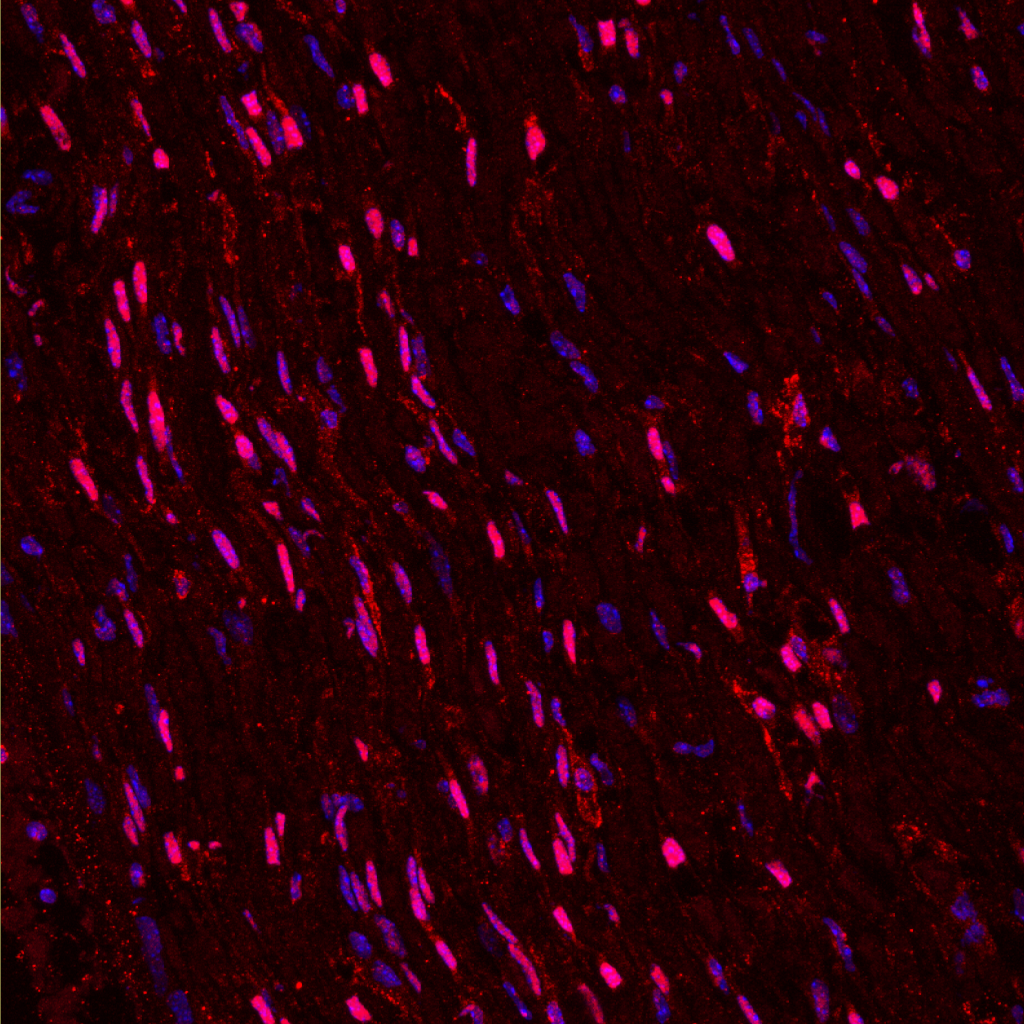

Supplement: Figure 3—source data 2. — This zip archive contains the IHC for one WT and one iDKO used for quantitative analysis shown in Figure 3H. Results and quantitation shown in the Figure used BD #550609 anti-Ki67. These results were confirmed using a second antibody, Abcam #ab15580 anti-Ki67. Images using both antibodies are included in the zip archive, in the indicated folders. Leica SP8 confocal lif images were processed using Imaris software and saved as tiffs. [file elife-50138-fig3-data2.zip › Figure 3 source data 2/WT #943 Ki67/Abcam #ab15580 Ki67/Series 35 Sox10 + DAPI.tif]

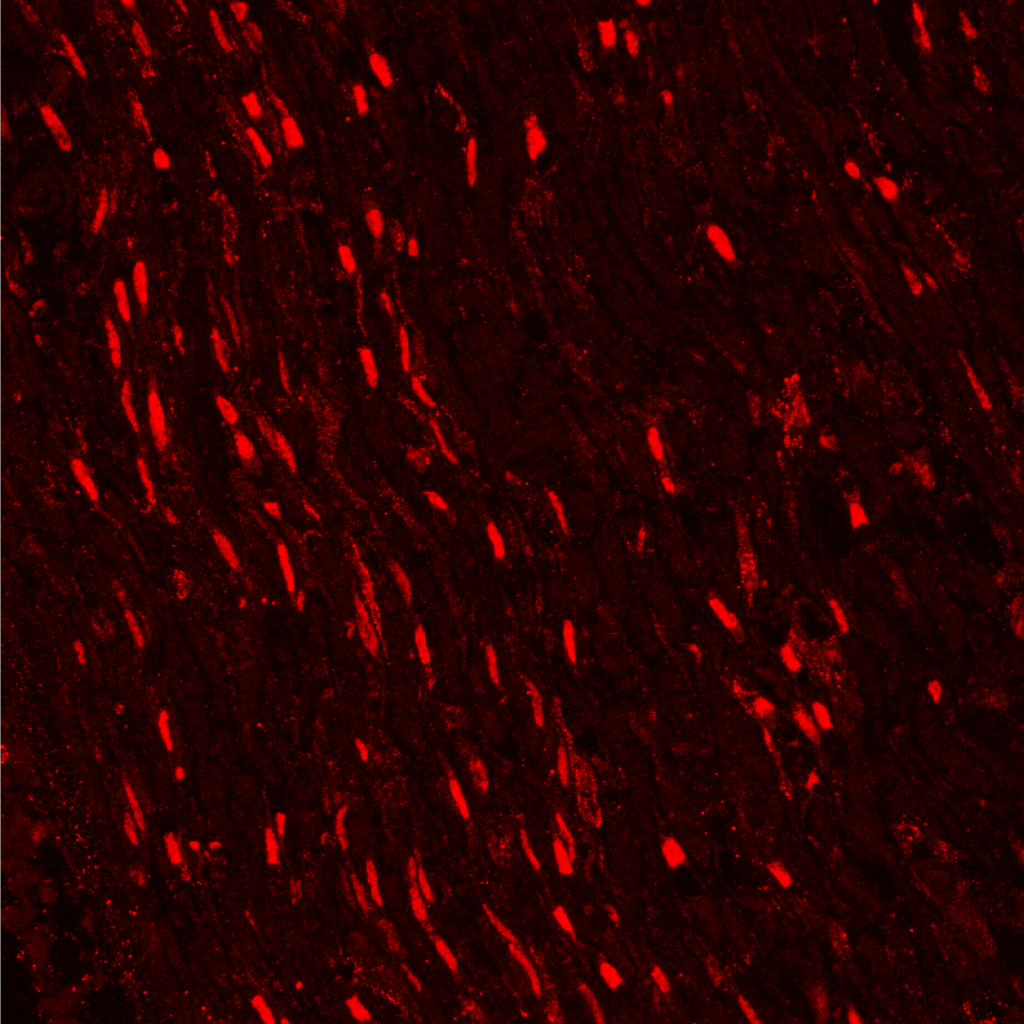

Supplement: Figure 3—source data 2. — This zip archive contains the IHC for one WT and one iDKO used for quantitative analysis shown in Figure 3H. Results and quantitation shown in the Figure used BD #550609 anti-Ki67. These results were confirmed using a second antibody, Abcam #ab15580 anti-Ki67. Images using both antibodies are included in the zip archive, in the indicated folders. Leica SP8 confocal lif images were processed using Imaris software and saved as tiffs. [file elife-50138-fig3-data2.zip › Figure 3 source data 2/WT #943 Ki67/Abcam #ab15580 Ki67/Series 35 Sox10.tif]

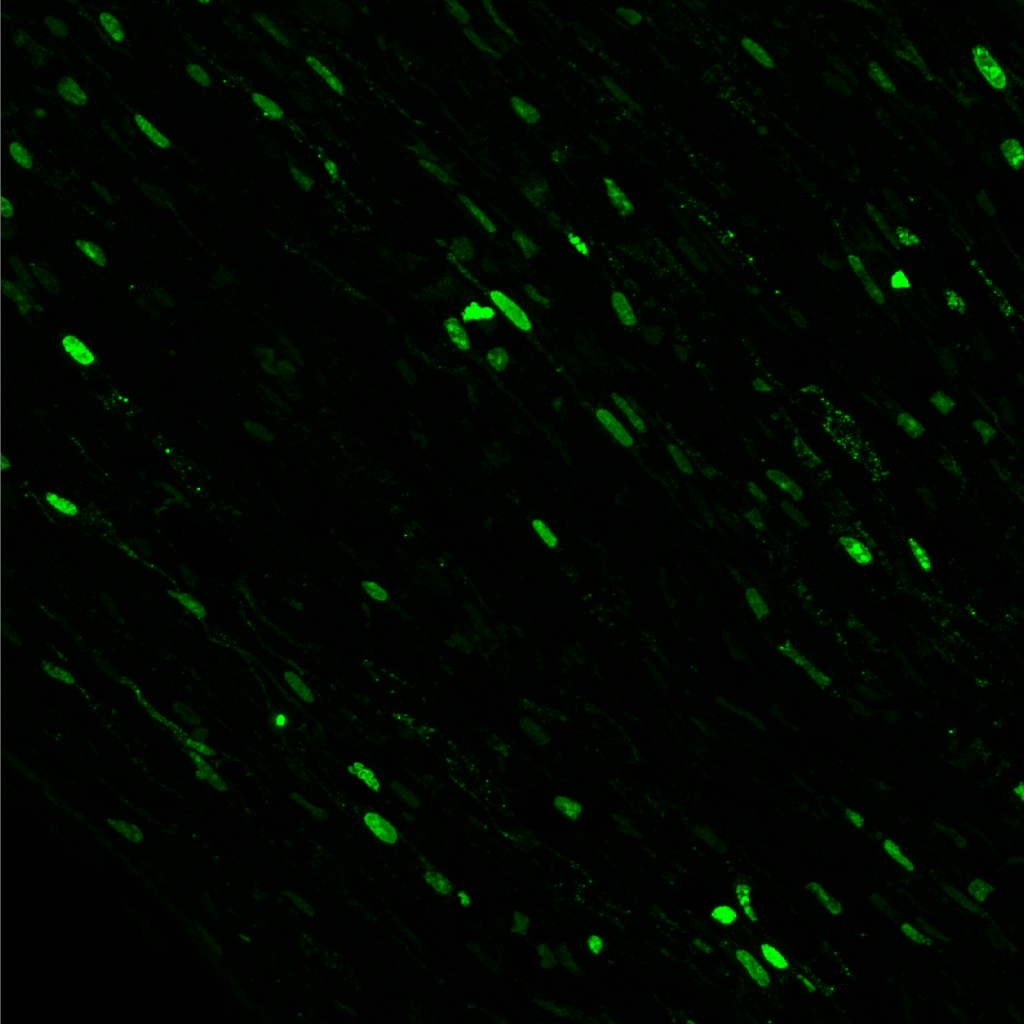

Supplement: Figure 3—source data 2. — This zip archive contains the IHC for one WT and one iDKO used for quantitative analysis shown in Figure 3H. Results and quantitation shown in the Figure used BD #550609 anti-Ki67. These results were confirmed using a second antibody, Abcam #ab15580 anti-Ki67. Images using both antibodies are included in the zip archive, in the indicated folders. Leica SP8 confocal lif images were processed using Imaris software and saved as tiffs. [file elife-50138-fig3-data2.zip › Figure 3 source data 2/WT #943 Ki67/Abcam #ab15580 Ki67/Series 8 Ki67.tif]

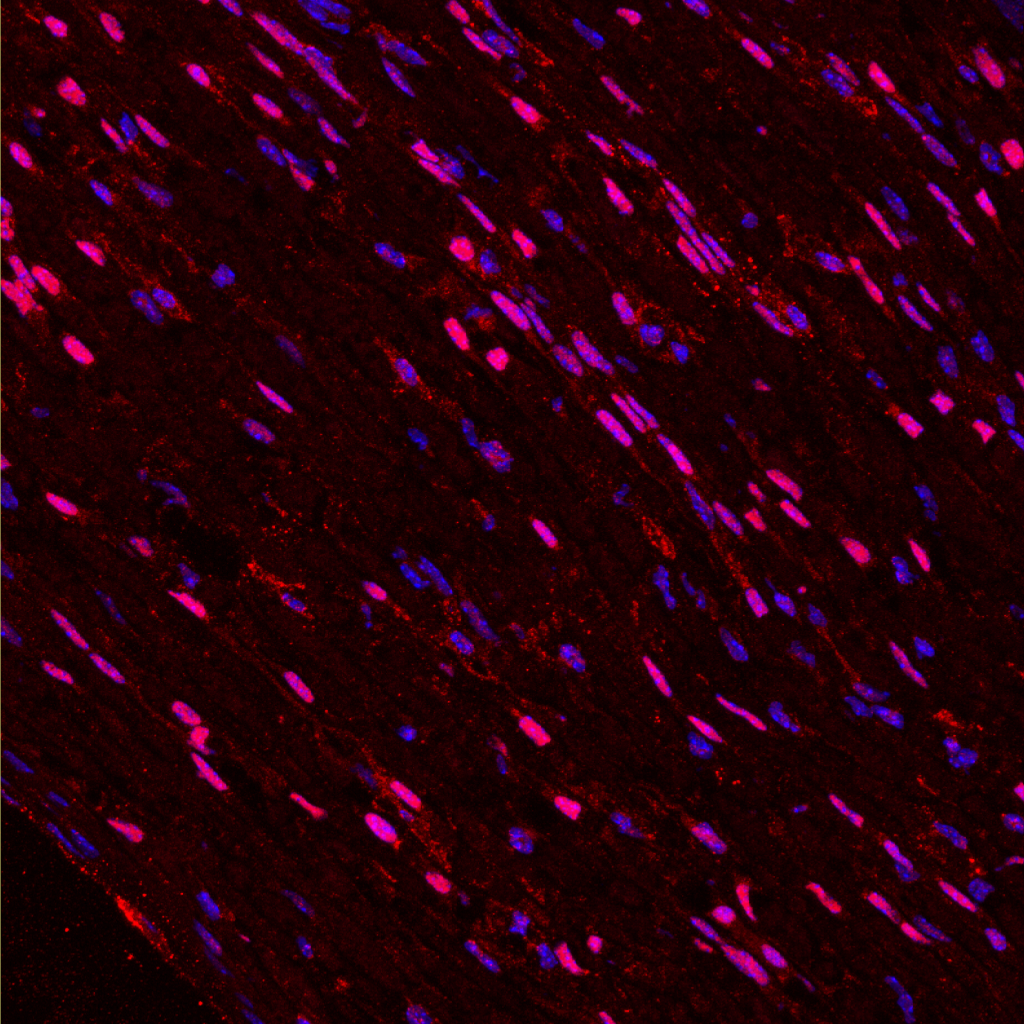

Supplement: Figure 3—source data 2. — This zip archive contains the IHC for one WT and one iDKO used for quantitative analysis shown in Figure 3H. Results and quantitation shown in the Figure used BD #550609 anti-Ki67. These results were confirmed using a second antibody, Abcam #ab15580 anti-Ki67. Images using both antibodies are included in the zip archive, in the indicated folders. Leica SP8 confocal lif images were processed using Imaris software and saved as tiffs. [file elife-50138-fig3-data2.zip › Figure 3 source data 2/WT #943 Ki67/Abcam #ab15580 Ki67/Series 8 Sox10 + DAPI.tif]

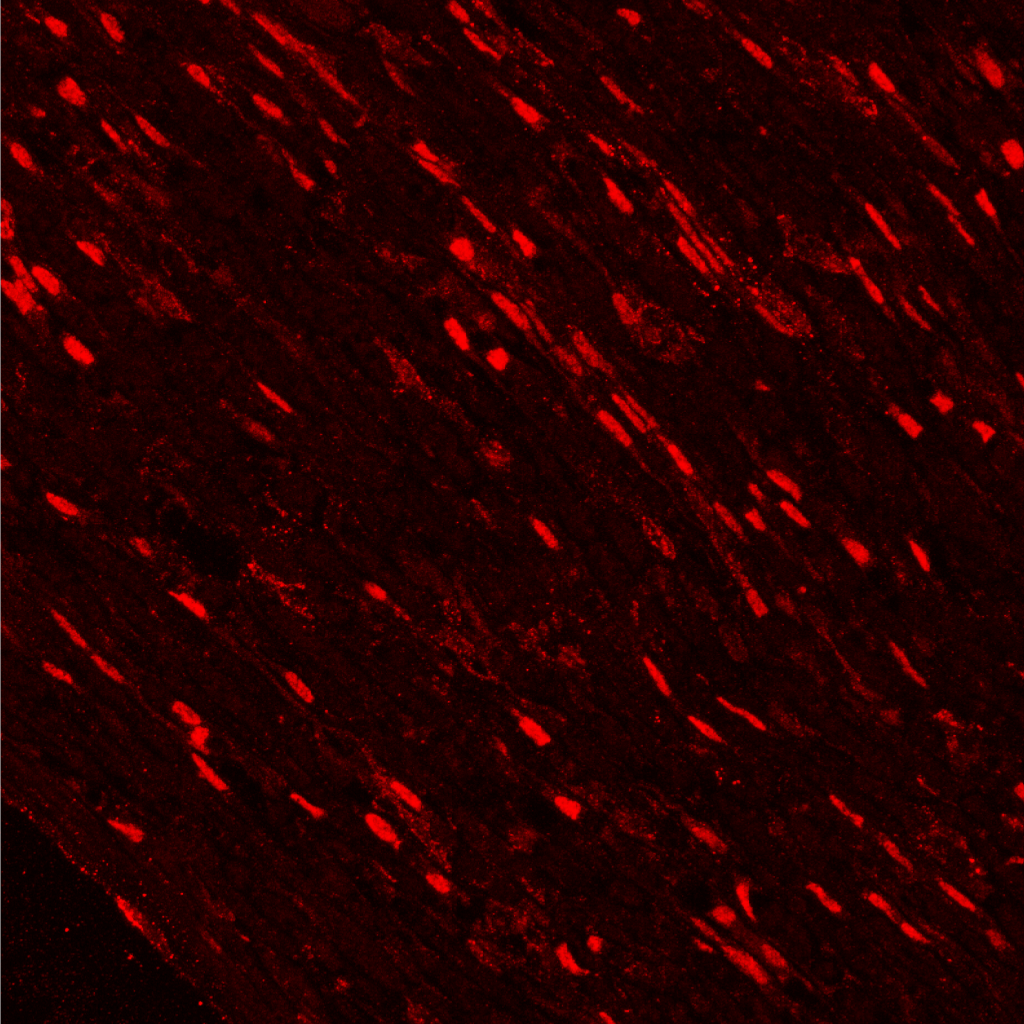

Supplement: Figure 3—source data 2. — This zip archive contains the IHC for one WT and one iDKO used for quantitative analysis shown in Figure 3H. Results and quantitation shown in the Figure used BD #550609 anti-Ki67. These results were confirmed using a second antibody, Abcam #ab15580 anti-Ki67. Images using both antibodies are included in the zip archive, in the indicated folders. Leica SP8 confocal lif images were processed using Imaris software and saved as tiffs. [file elife-50138-fig3-data2.zip › Figure 3 source data 2/WT #943 Ki67/Abcam #ab15580 Ki67/Series 8 Sox10.tif]

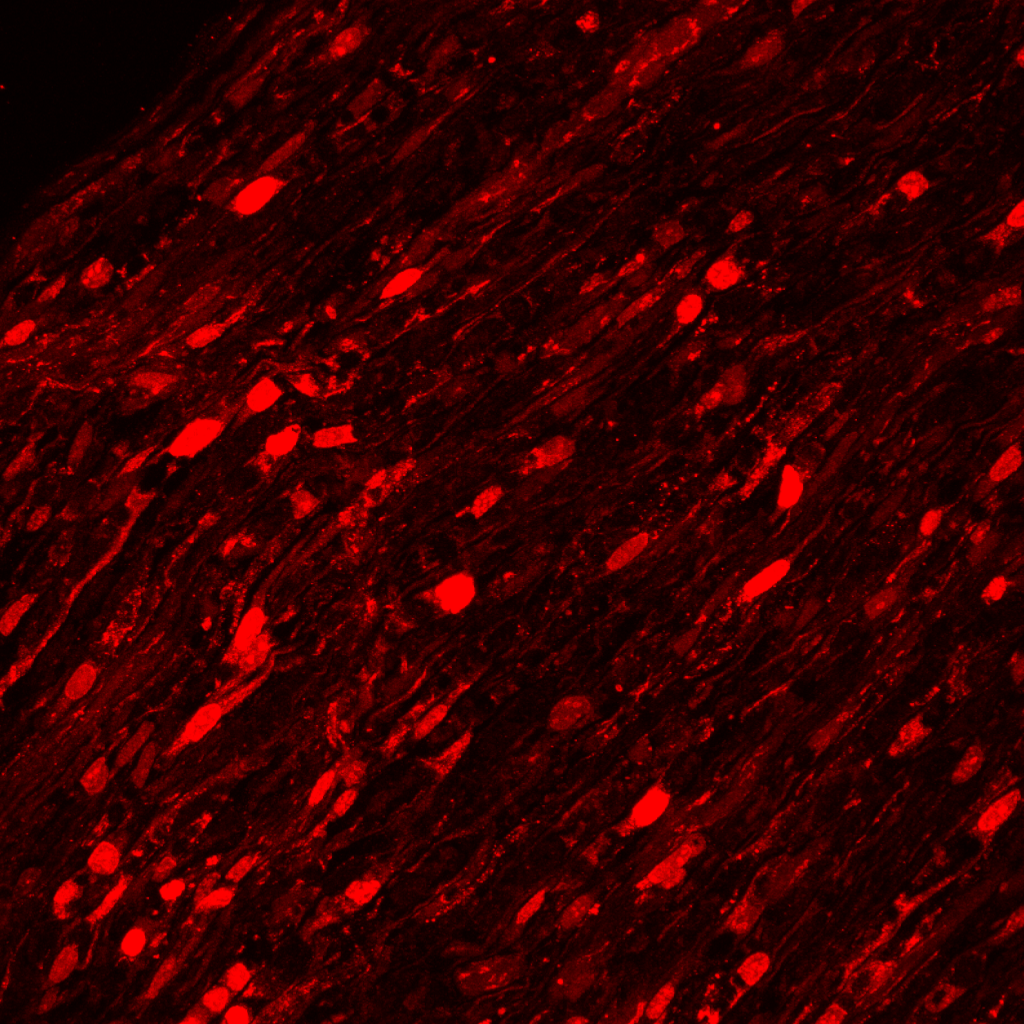

Supplement: Figure 3—source data 2. — This zip archive contains the IHC for one WT and one iDKO used for quantitative analysis shown in Figure 3H. Results and quantitation shown in the Figure used BD #550609 anti-Ki67. These results were confirmed using a second antibody, Abcam #ab15580 anti-Ki67. Images using both antibodies are included in the zip archive, in the indicated folders. Leica SP8 confocal lif images were processed using Imaris software and saved as tiffs. [file elife-50138-fig3-data2.zip › Figure 3 source data 2/WT #943 Ki67/BD #550609 Ki67/Series 10 Ki67.tif]

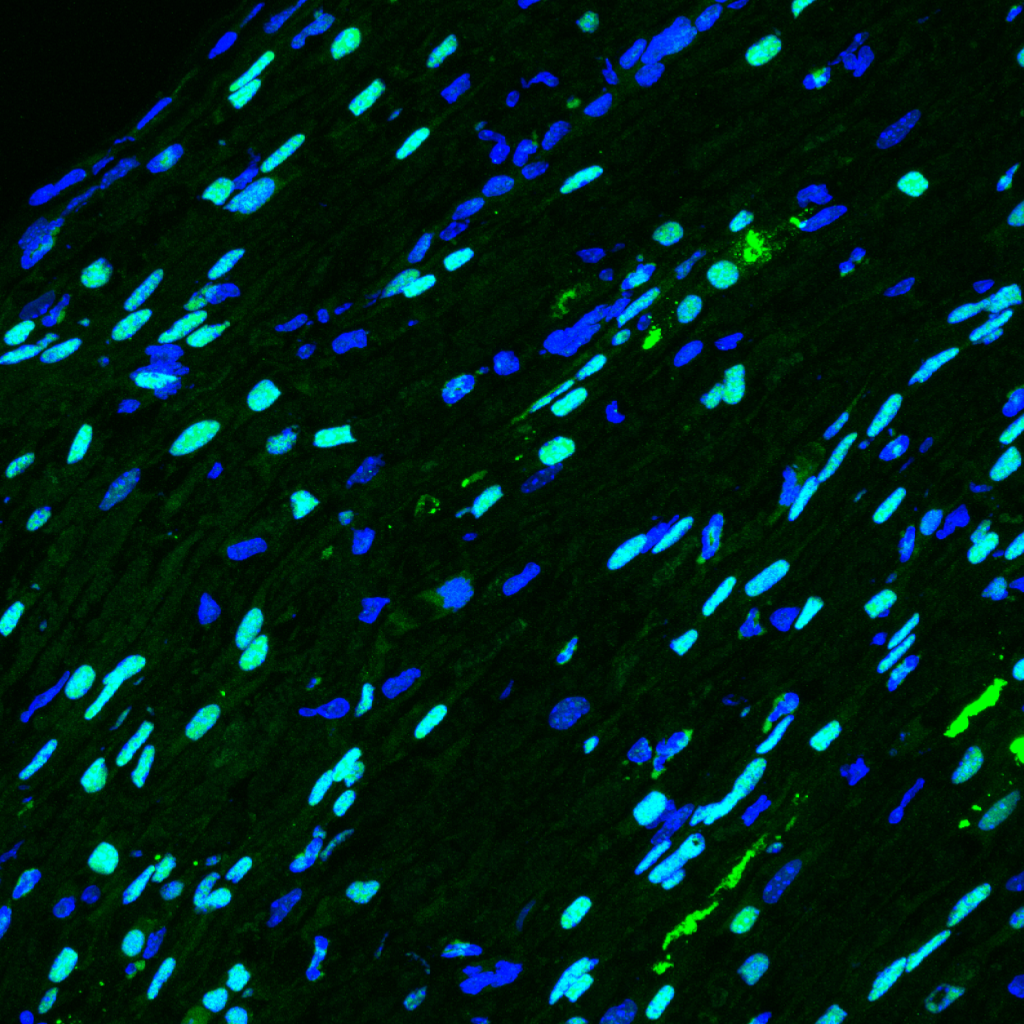

Supplement: Figure 3—source data 2. — This zip archive contains the IHC for one WT and one iDKO used for quantitative analysis shown in Figure 3H. Results and quantitation shown in the Figure used BD #550609 anti-Ki67. These results were confirmed using a second antibody, Abcam #ab15580 anti-Ki67. Images using both antibodies are included in the zip archive, in the indicated folders. Leica SP8 confocal lif images were processed using Imaris software and saved as tiffs. [file elife-50138-fig3-data2.zip › Figure 3 source data 2/WT #943 Ki67/BD #550609 Ki67/Series 10 Sox10 + DAPI.tif]

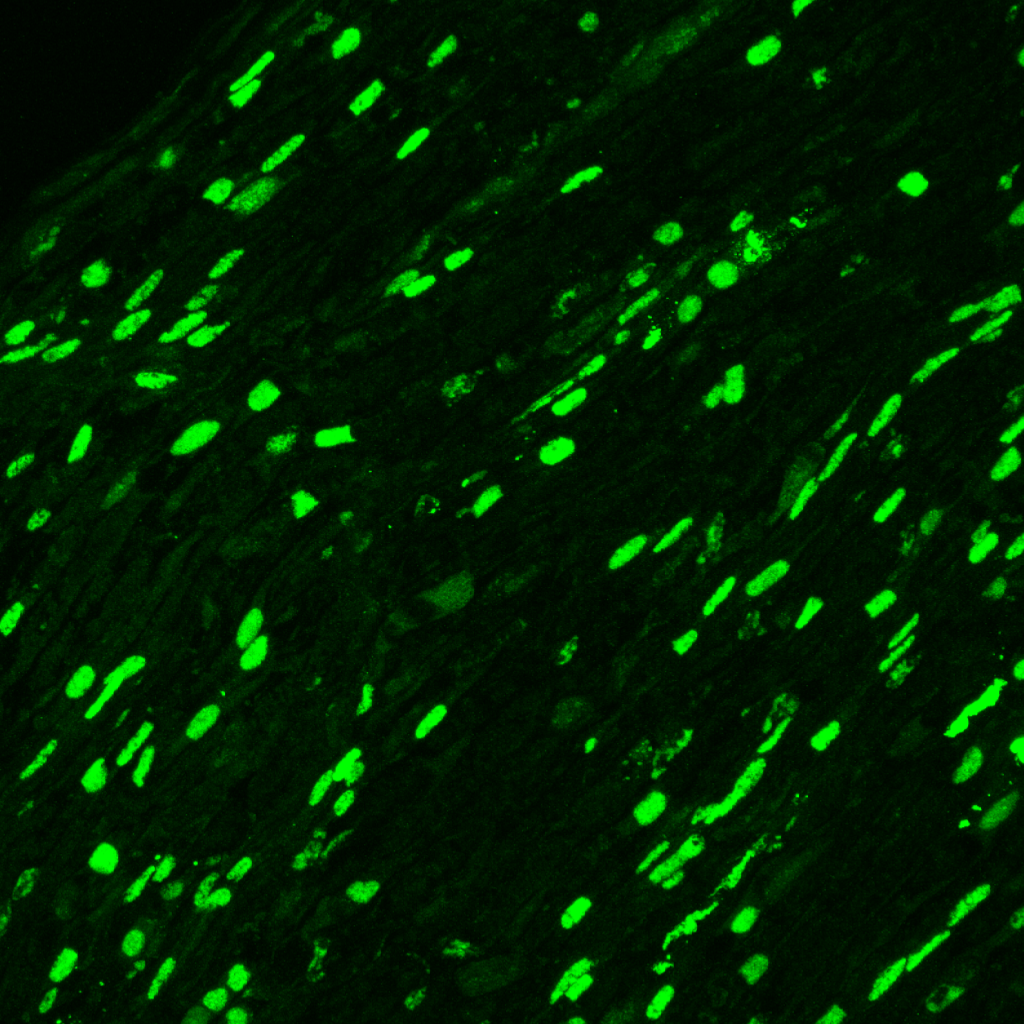

Supplement: Figure 3—source data 2. — This zip archive contains the IHC for one WT and one iDKO used for quantitative analysis shown in Figure 3H. Results and quantitation shown in the Figure used BD #550609 anti-Ki67. These results were confirmed using a second antibody, Abcam #ab15580 anti-Ki67. Images using both antibodies are included in the zip archive, in the indicated folders. Leica SP8 confocal lif images were processed using Imaris software and saved as tiffs. [file elife-50138-fig3-data2.zip › Figure 3 source data 2/WT #943 Ki67/BD #550609 Ki67/Series 10 Sox10.tif]

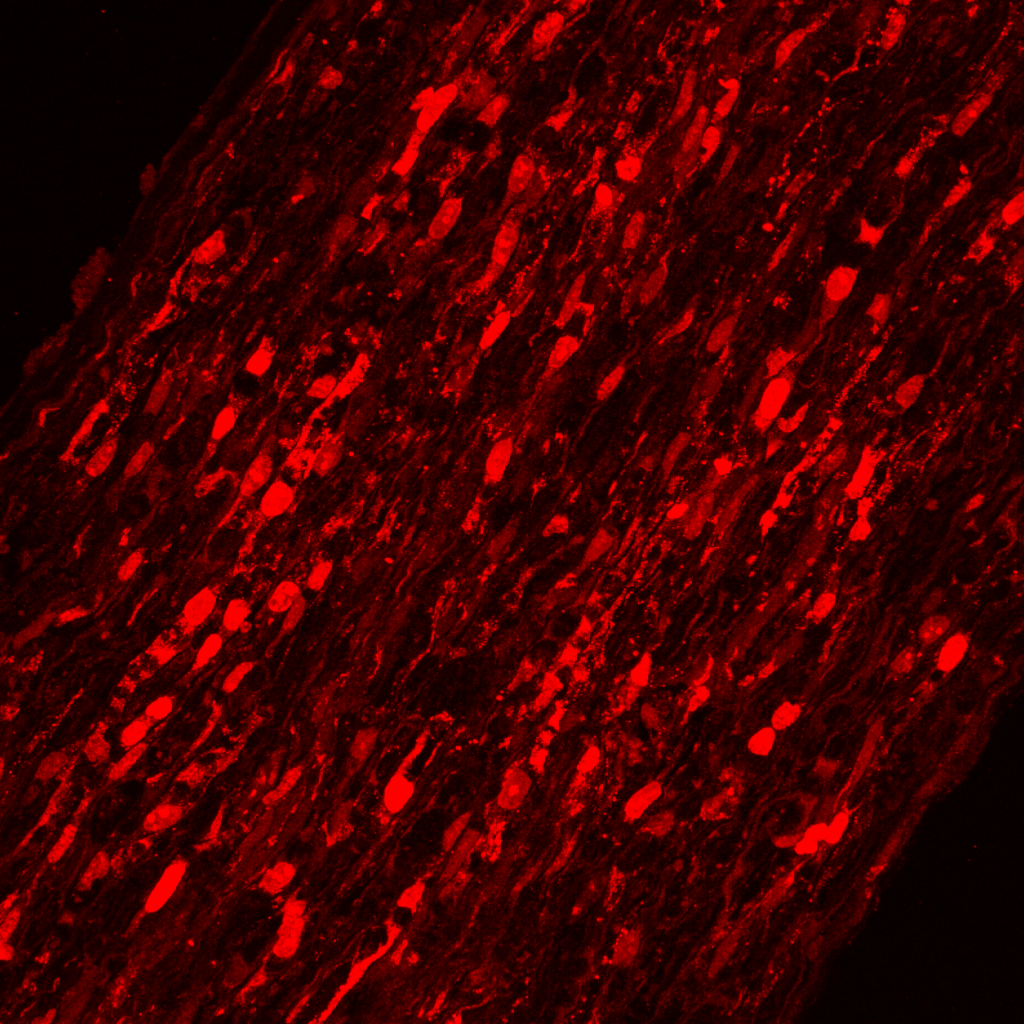

Supplement: Figure 3—source data 2. — This zip archive contains the IHC for one WT and one iDKO used for quantitative analysis shown in Figure 3H. Results and quantitation shown in the Figure used BD #550609 anti-Ki67. These results were confirmed using a second antibody, Abcam #ab15580 anti-Ki67. Images using both antibodies are included in the zip archive, in the indicated folders. Leica SP8 confocal lif images were processed using Imaris software and saved as tiffs. [file elife-50138-fig3-data2.zip › Figure 3 source data 2/WT #943 Ki67/BD #550609 Ki67/Series 18 Ki67.tif]

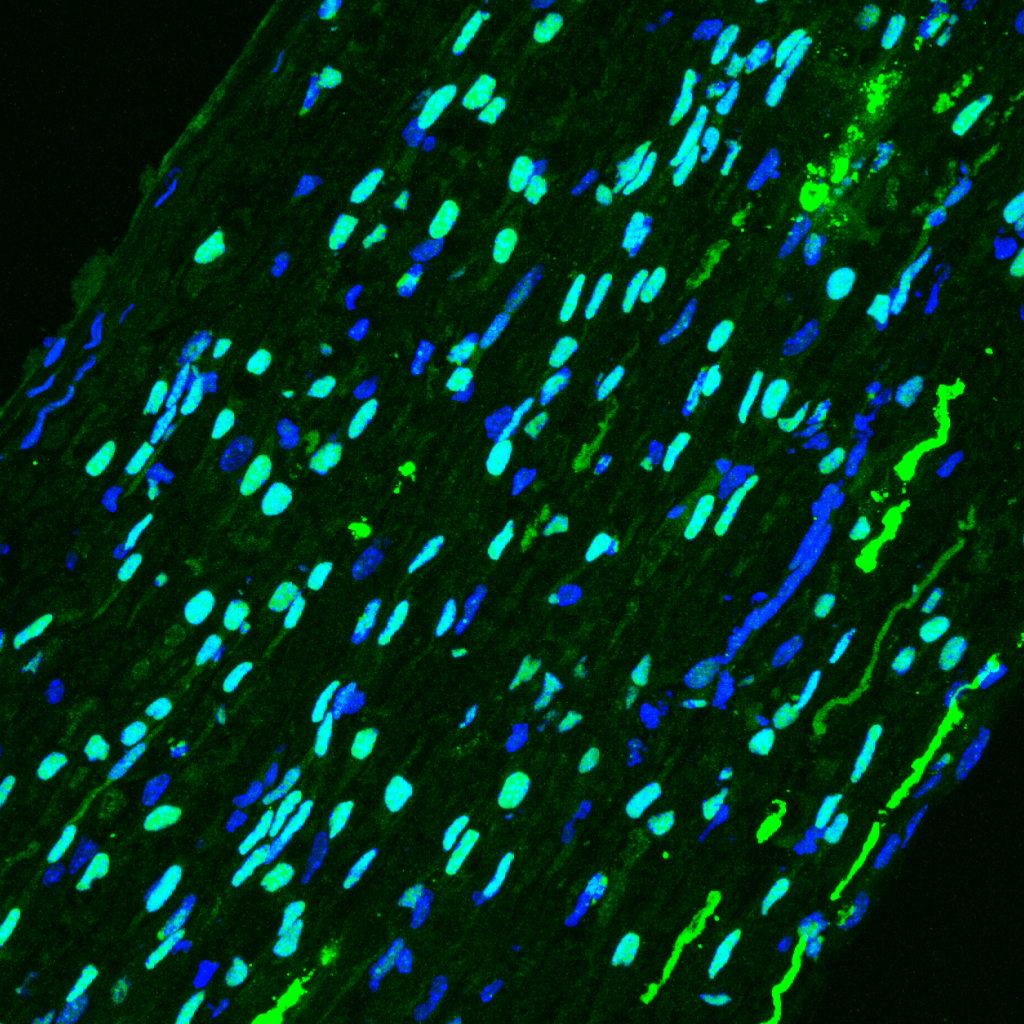

Supplement: Figure 3—source data 2. — This zip archive contains the IHC for one WT and one iDKO used for quantitative analysis shown in Figure 3H. Results and quantitation shown in the Figure used BD #550609 anti-Ki67. These results were confirmed using a second antibody, Abcam #ab15580 anti-Ki67. Images using both antibodies are included in the zip archive, in the indicated folders. Leica SP8 confocal lif images were processed using Imaris software and saved as tiffs. [file elife-50138-fig3-data2.zip › Figure 3 source data 2/WT #943 Ki67/BD #550609 Ki67/Series 18 Sox10 + DAPI.tif]

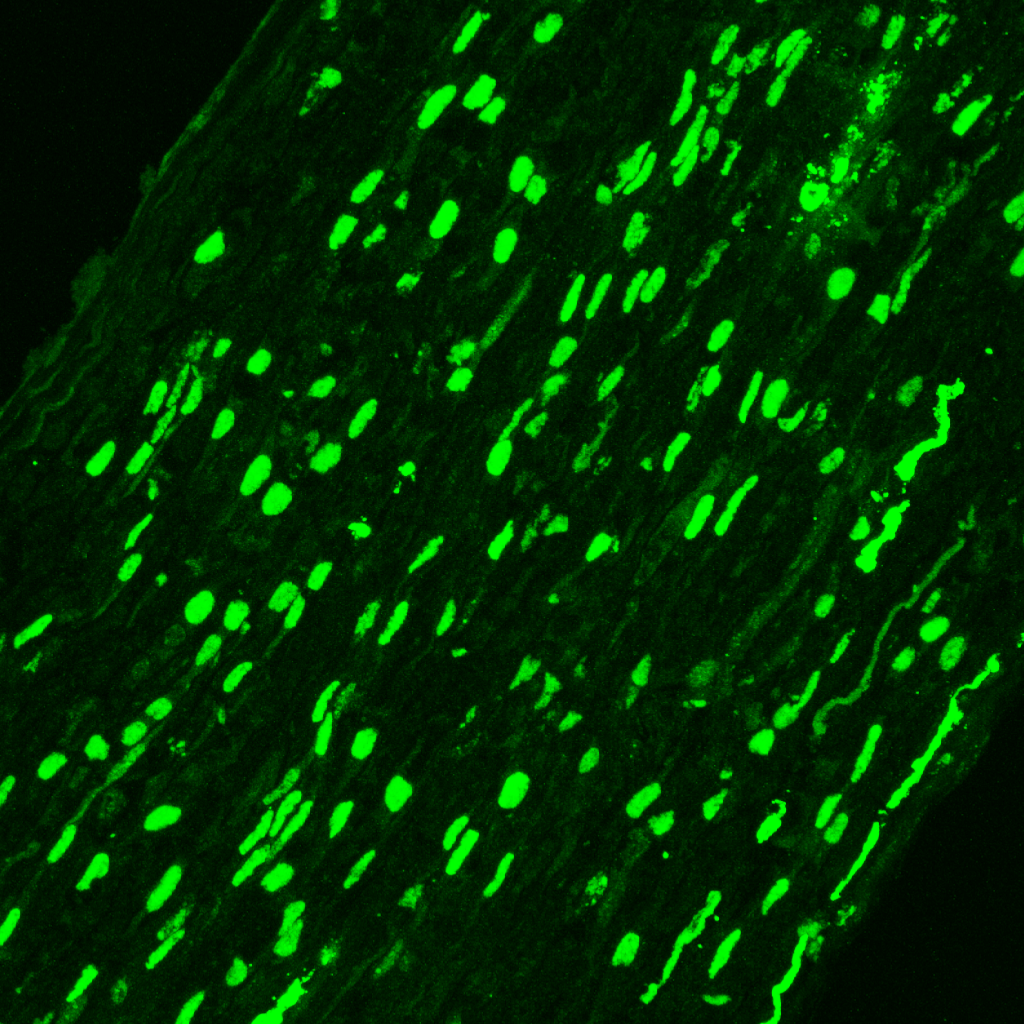

Supplement: Figure 3—source data 2. — This zip archive contains the IHC for one WT and one iDKO used for quantitative analysis shown in Figure 3H. Results and quantitation shown in the Figure used BD #550609 anti-Ki67. These results were confirmed using a second antibody, Abcam #ab15580 anti-Ki67. Images using both antibodies are included in the zip archive, in the indicated folders. Leica SP8 confocal lif images were processed using Imaris software and saved as tiffs. [file elife-50138-fig3-data2.zip › Figure 3 source data 2/WT #943 Ki67/BD #550609 Ki67/Series 18 Sox10.tif]

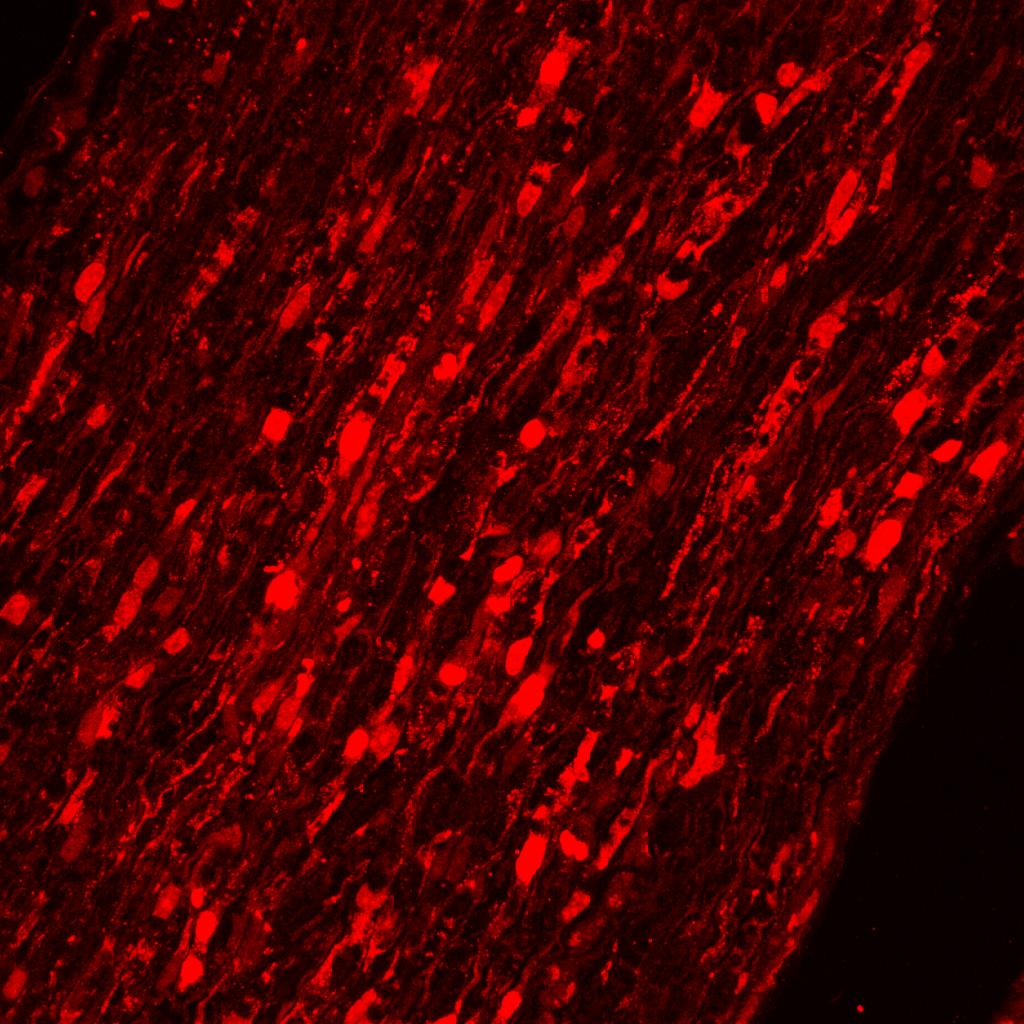

Supplement: Figure 3—source data 2. — This zip archive contains the IHC for one WT and one iDKO used for quantitative analysis shown in Figure 3H. Results and quantitation shown in the Figure used BD #550609 anti-Ki67. These results were confirmed using a second antibody, Abcam #ab15580 anti-Ki67. Images using both antibodies are included in the zip archive, in the indicated folders. Leica SP8 confocal lif images were processed using Imaris software and saved as tiffs. [file elife-50138-fig3-data2.zip › Figure 3 source data 2/WT #943 Ki67/BD #550609 Ki67/Series 26 Ki67.tif]

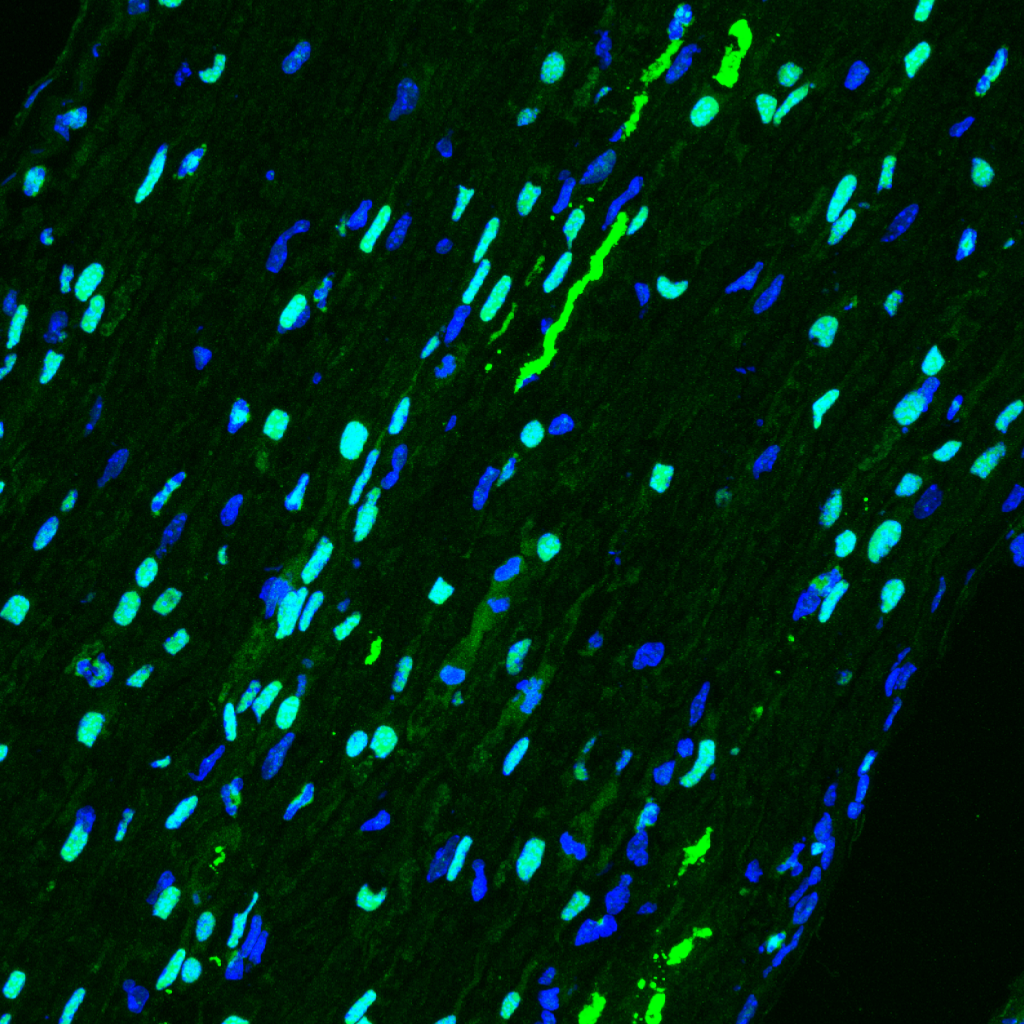

Supplement: Figure 3—source data 2. — This zip archive contains the IHC for one WT and one iDKO used for quantitative analysis shown in Figure 3H. Results and quantitation shown in the Figure used BD #550609 anti-Ki67. These results were confirmed using a second antibody, Abcam #ab15580 anti-Ki67. Images using both antibodies are included in the zip archive, in the indicated folders. Leica SP8 confocal lif images were processed using Imaris software and saved as tiffs. [file elife-50138-fig3-data2.zip › Figure 3 source data 2/WT #943 Ki67/BD #550609 Ki67/Series 26 Sox10 + DAPI.tif]

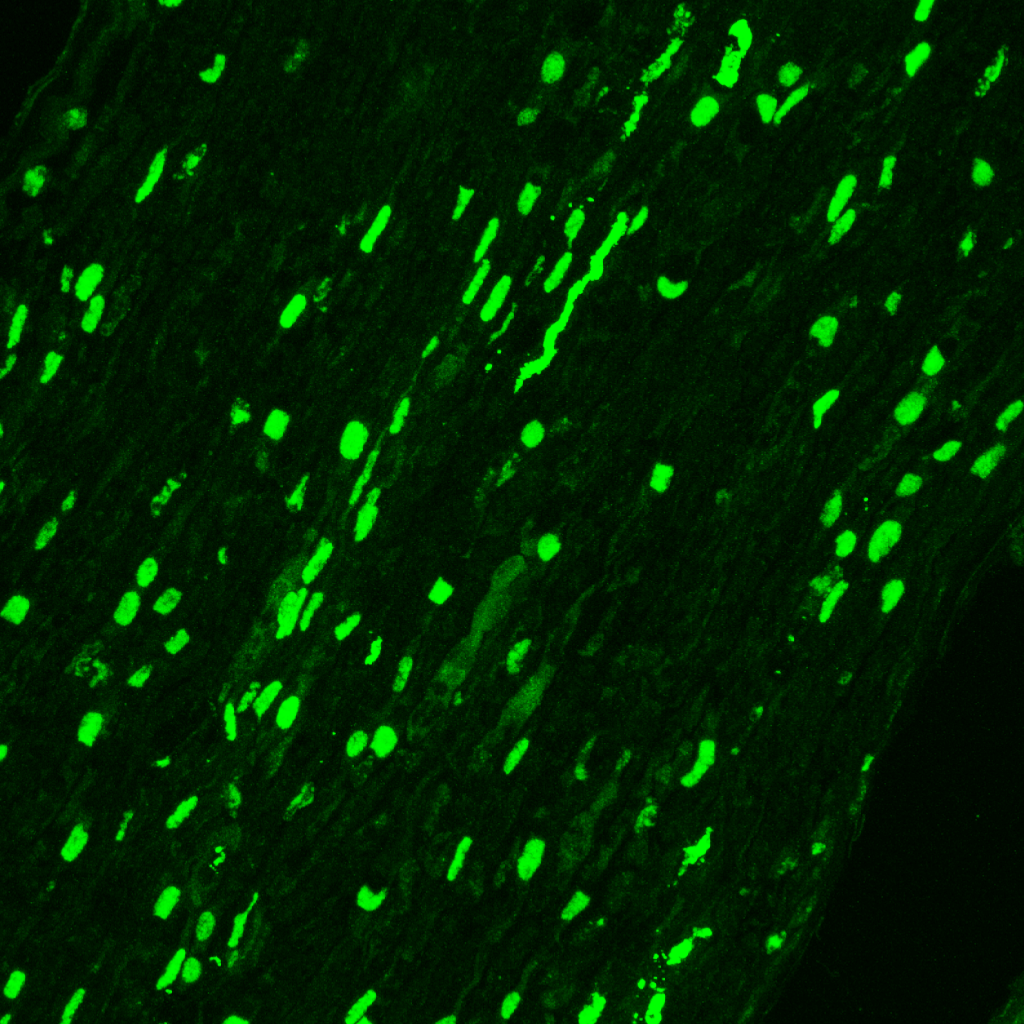

Supplement: Figure 3—source data 2. — This zip archive contains the IHC for one WT and one iDKO used for quantitative analysis shown in Figure 3H. Results and quantitation shown in the Figure used BD #550609 anti-Ki67. These results were confirmed using a second antibody, Abcam #ab15580 anti-Ki67. Images using both antibodies are included in the zip archive, in the indicated folders. Leica SP8 confocal lif images were processed using Imaris software and saved as tiffs. [file elife-50138-fig3-data2.zip › Figure 3 source data 2/WT #943 Ki67/BD #550609 Ki67/Series 26 Sox10.tif]

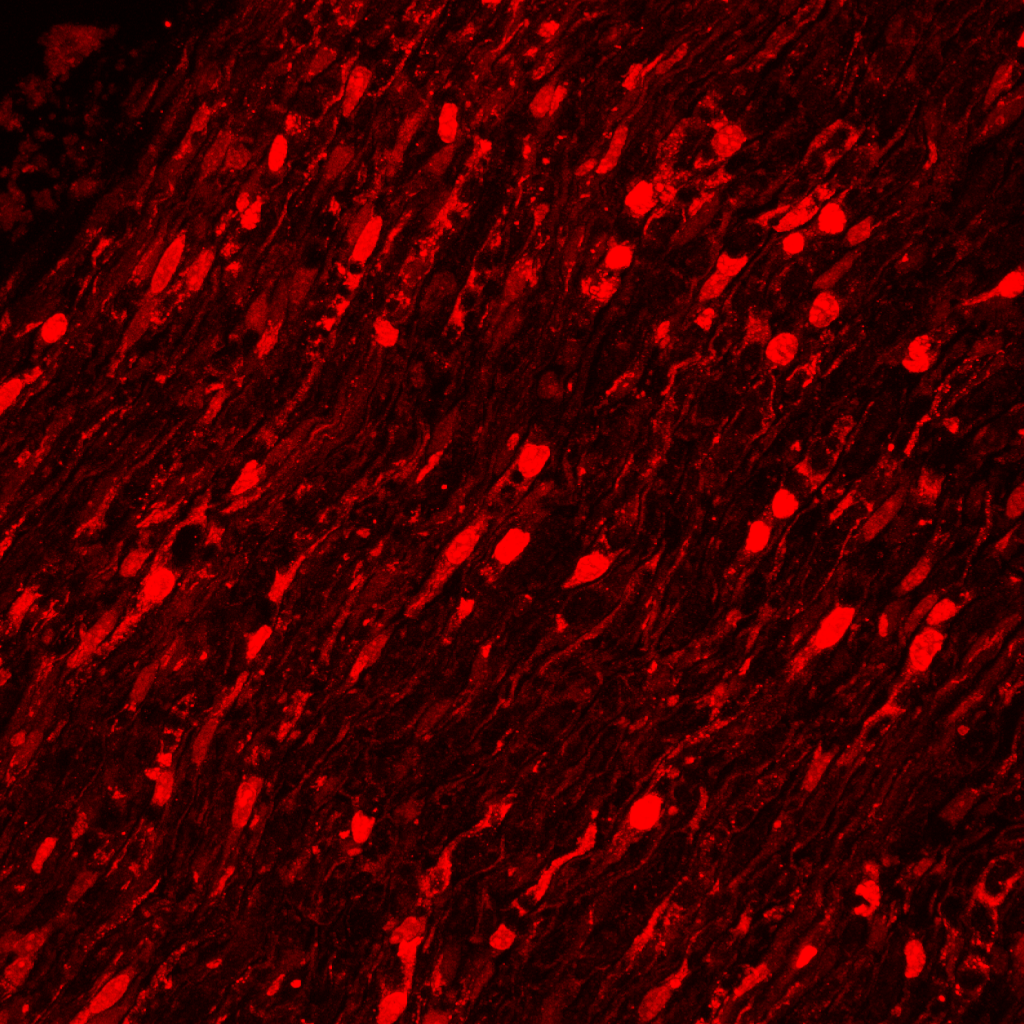

Supplement: Figure 3—source data 2. — This zip archive contains the IHC for one WT and one iDKO used for quantitative analysis shown in Figure 3H. Results and quantitation shown in the Figure used BD #550609 anti-Ki67. These results were confirmed using a second antibody, Abcam #ab15580 anti-Ki67. Images using both antibodies are included in the zip archive, in the indicated folders. Leica SP8 confocal lif images were processed using Imaris software and saved as tiffs. [file elife-50138-fig3-data2.zip › Figure 3 source data 2/WT #943 Ki67/BD #550609 Ki67/Series 34 Ki67.tif]

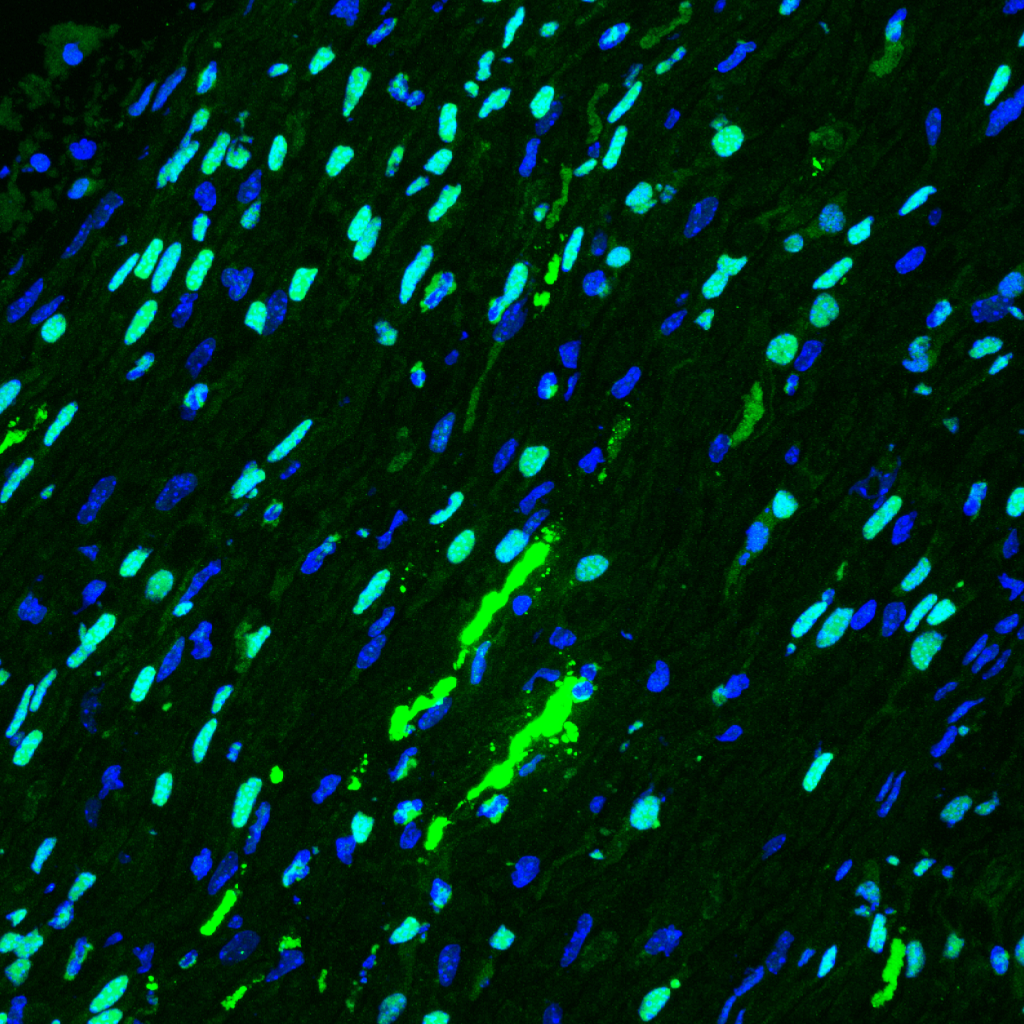

Supplement: Figure 3—source data 2. — This zip archive contains the IHC for one WT and one iDKO used for quantitative analysis shown in Figure 3H. Results and quantitation shown in the Figure used BD #550609 anti-Ki67. These results were confirmed using a second antibody, Abcam #ab15580 anti-Ki67. Images using both antibodies are included in the zip archive, in the indicated folders. Leica SP8 confocal lif images were processed using Imaris software and saved as tiffs. [file elife-50138-fig3-data2.zip › Figure 3 source data 2/WT #943 Ki67/BD #550609 Ki67/Series 34 Sox10 + DAPI.tif]

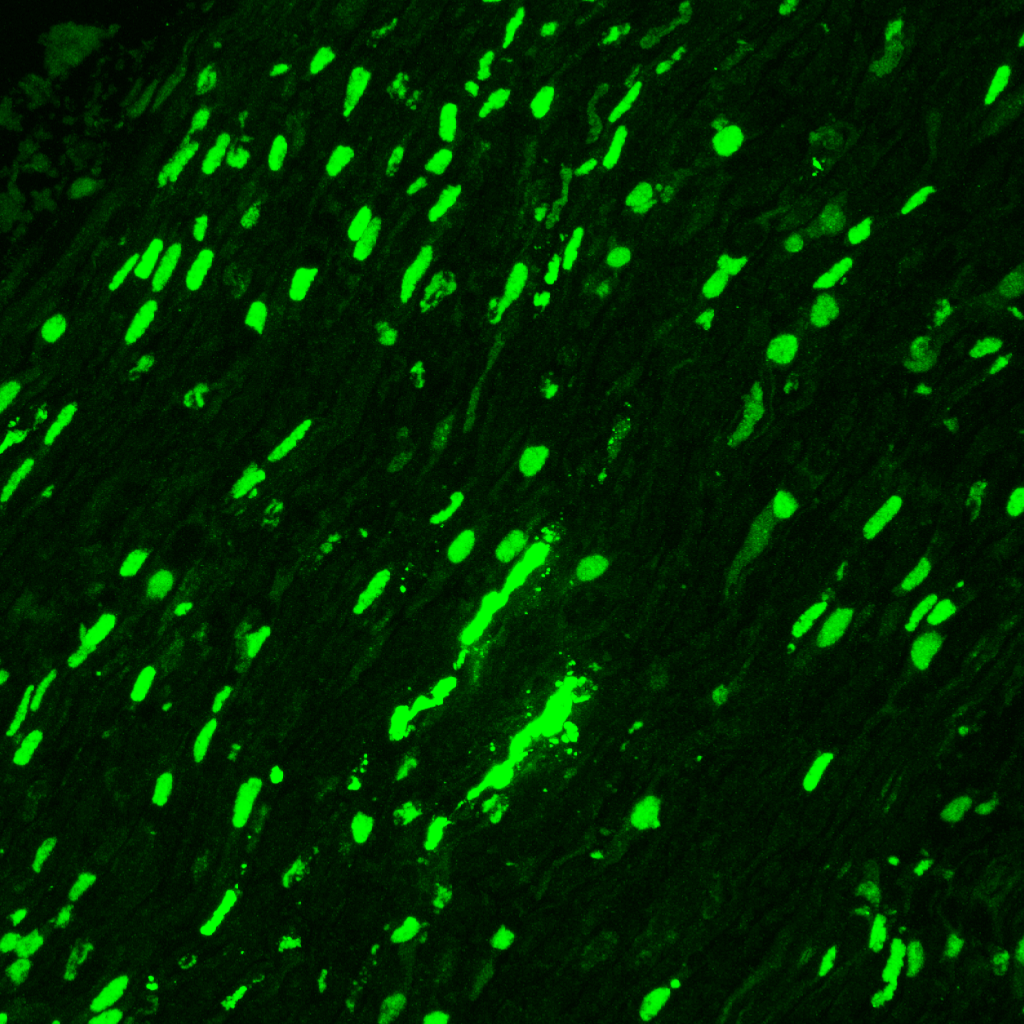

Supplement: Figure 3—source data 2. — This zip archive contains the IHC for one WT and one iDKO used for quantitative analysis shown in Figure 3H. Results and quantitation shown in the Figure used BD #550609 anti-Ki67. These results were confirmed using a second antibody, Abcam #ab15580 anti-Ki67. Images using both antibodies are included in the zip archive, in the indicated folders. Leica SP8 confocal lif images were processed using Imaris software and saved as tiffs. [file elife-50138-fig3-data2.zip › Figure 3 source data 2/WT #943 Ki67/BD #550609 Ki67/Series 34 Sox10.tif]

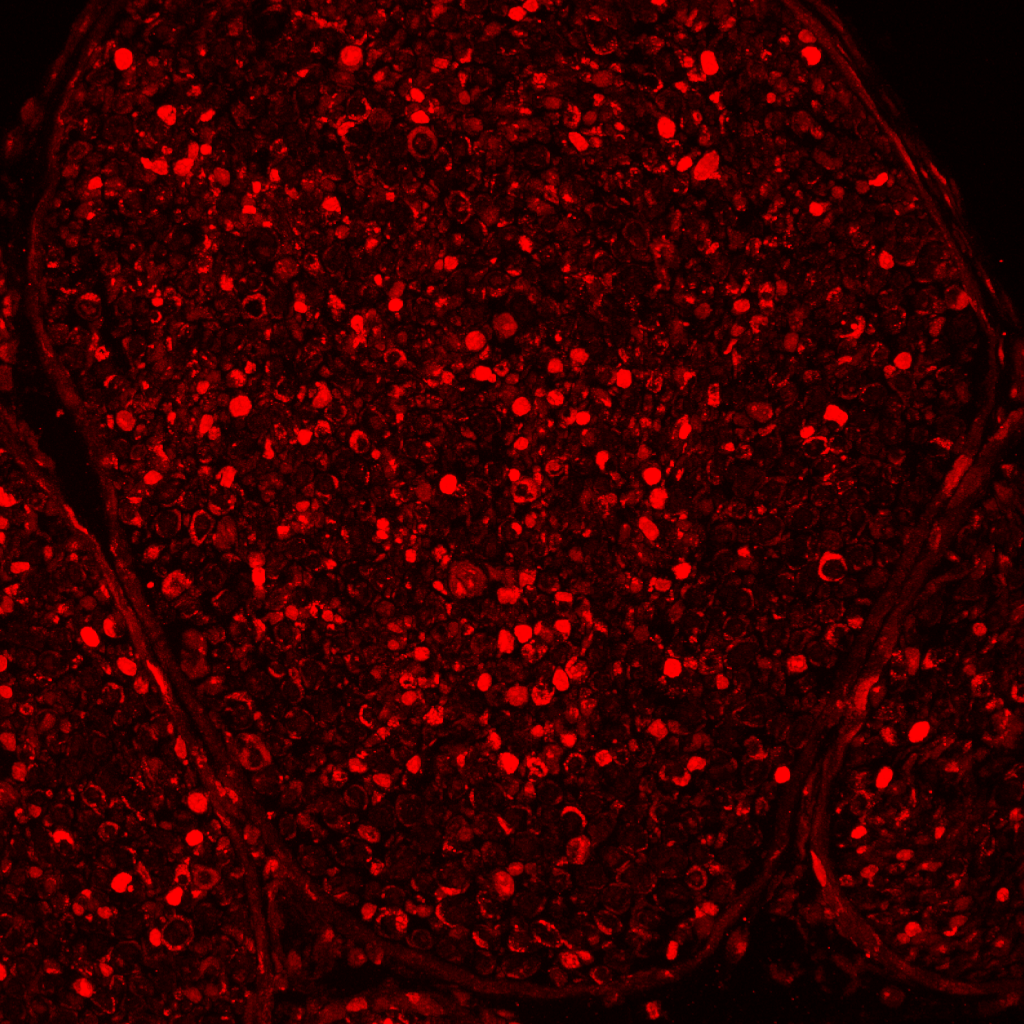

Supplement: Figure 3—source data 2. — This zip archive contains the IHC for one WT and one iDKO used for quantitative analysis shown in Figure 3H. Results and quantitation shown in the Figure used BD #550609 anti-Ki67. These results were confirmed using a second antibody, Abcam #ab15580 anti-Ki67. Images using both antibodies are included in the zip archive, in the indicated folders. Leica SP8 confocal lif images were processed using Imaris software and saved as tiffs. [file elife-50138-fig3-data2.zip › Figure 3 source data 2/WT #943 Ki67/BD #550609 Ki67/XS 1a Ki67.tif]

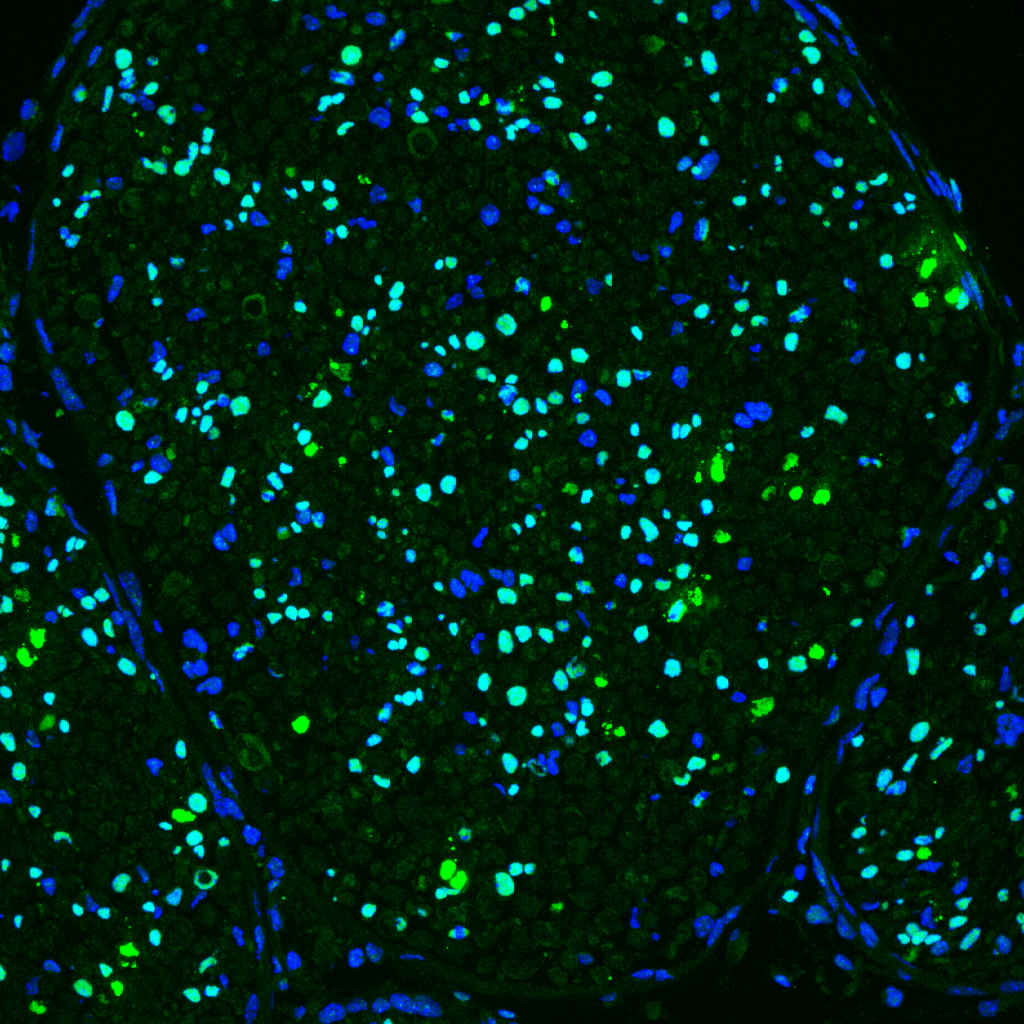

Supplement: Figure 3—source data 2. — This zip archive contains the IHC for one WT and one iDKO used for quantitative analysis shown in Figure 3H. Results and quantitation shown in the Figure used BD #550609 anti-Ki67. These results were confirmed using a second antibody, Abcam #ab15580 anti-Ki67. Images using both antibodies are included in the zip archive, in the indicated folders. Leica SP8 confocal lif images were processed using Imaris software and saved as tiffs. [file elife-50138-fig3-data2.zip › Figure 3 source data 2/WT #943 Ki67/BD #550609 Ki67/XS 1a Sox10 + DAPI.tif]

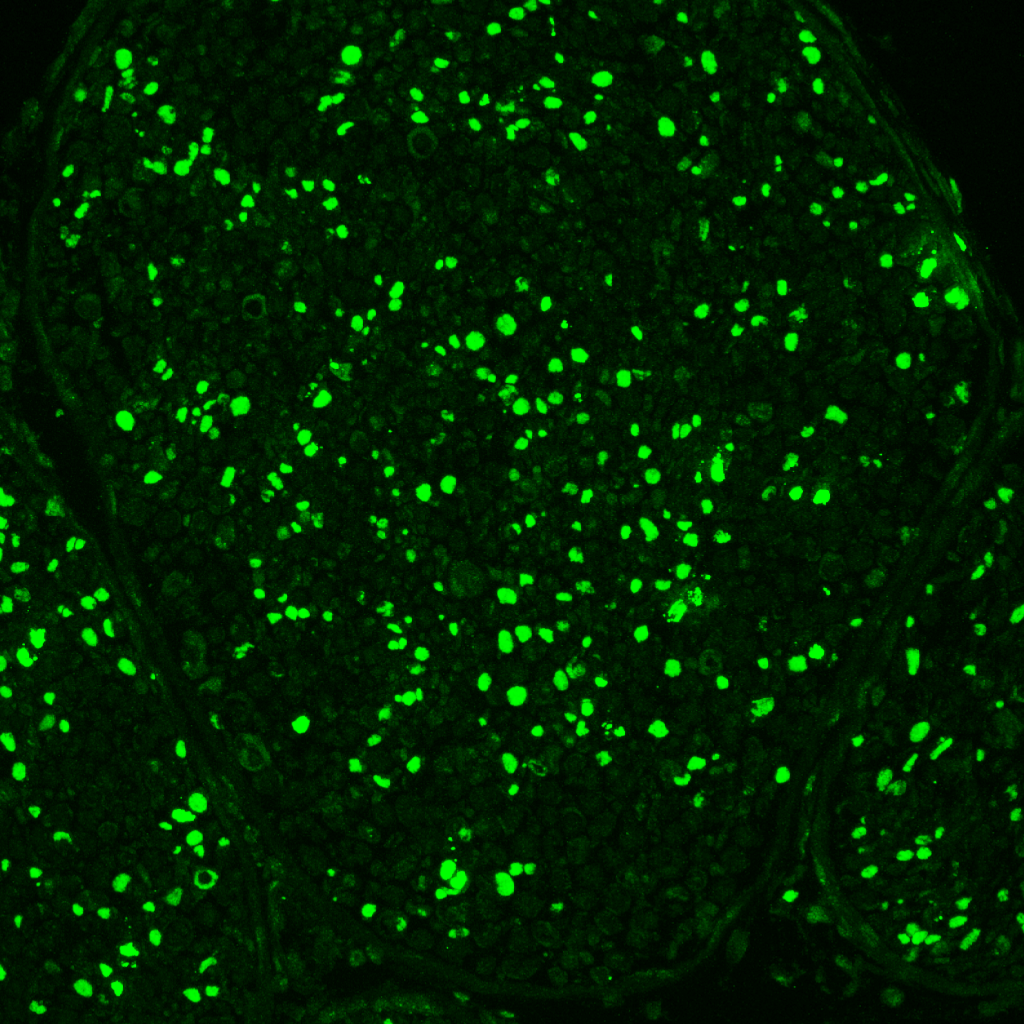

Supplement: Figure 3—source data 2. — This zip archive contains the IHC for one WT and one iDKO used for quantitative analysis shown in Figure 3H. Results and quantitation shown in the Figure used BD #550609 anti-Ki67. These results were confirmed using a second antibody, Abcam #ab15580 anti-Ki67. Images using both antibodies are included in the zip archive, in the indicated folders. Leica SP8 confocal lif images were processed using Imaris software and saved as tiffs. [file elife-50138-fig3-data2.zip › Figure 3 source data 2/WT #943 Ki67/BD #550609 Ki67/XS 1a Sox10.tif]

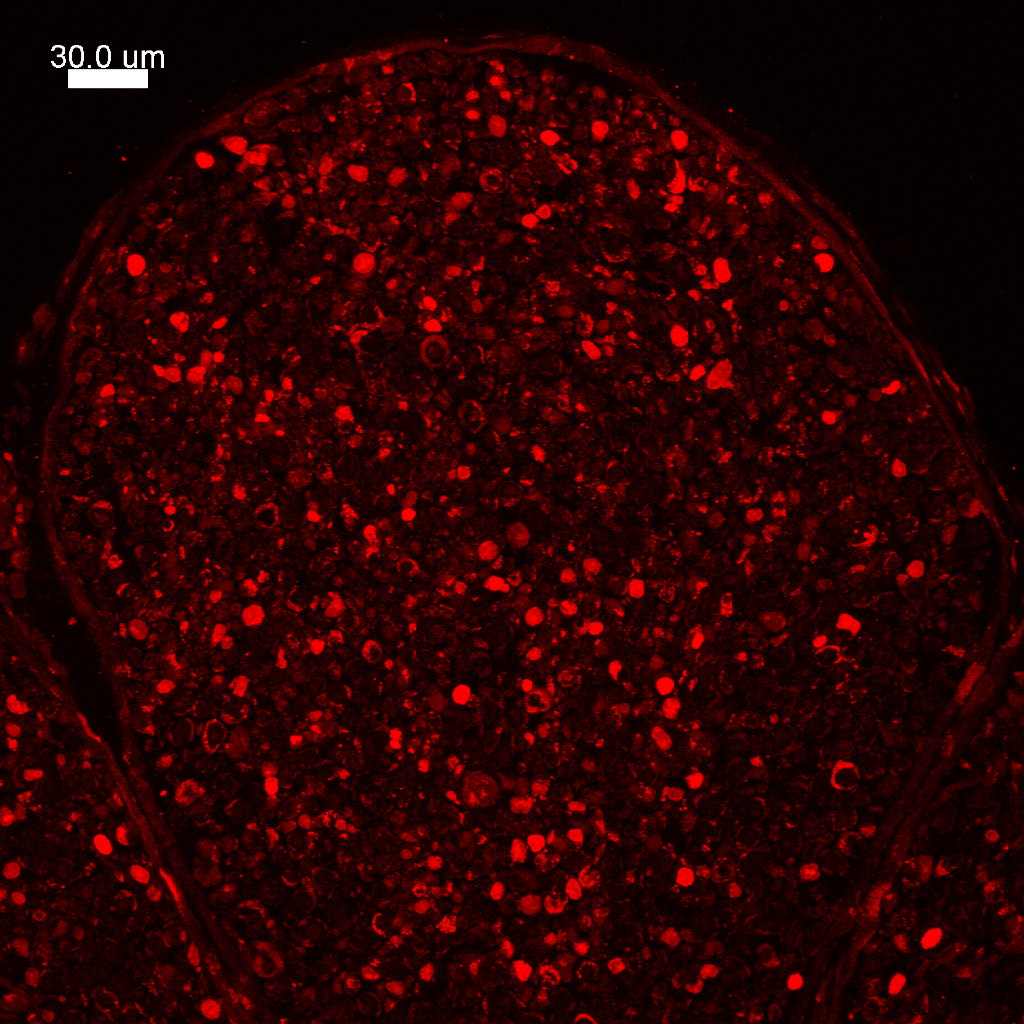

Supplement: Figure 3—source data 2. — This zip archive contains the IHC for one WT and one iDKO used for quantitative analysis shown in Figure 3H. Results and quantitation shown in the Figure used BD #550609 anti-Ki67. These results were confirmed using a second antibody, Abcam #ab15580 anti-Ki67. Images using both antibodies are included in the zip archive, in the indicated folders. Leica SP8 confocal lif images were processed using Imaris software and saved as tiffs. [file elife-50138-fig3-data2.zip › Figure 3 source data 2/WT #943 Ki67/BD #550609 Ki67/XS 1b Ki67.tif]

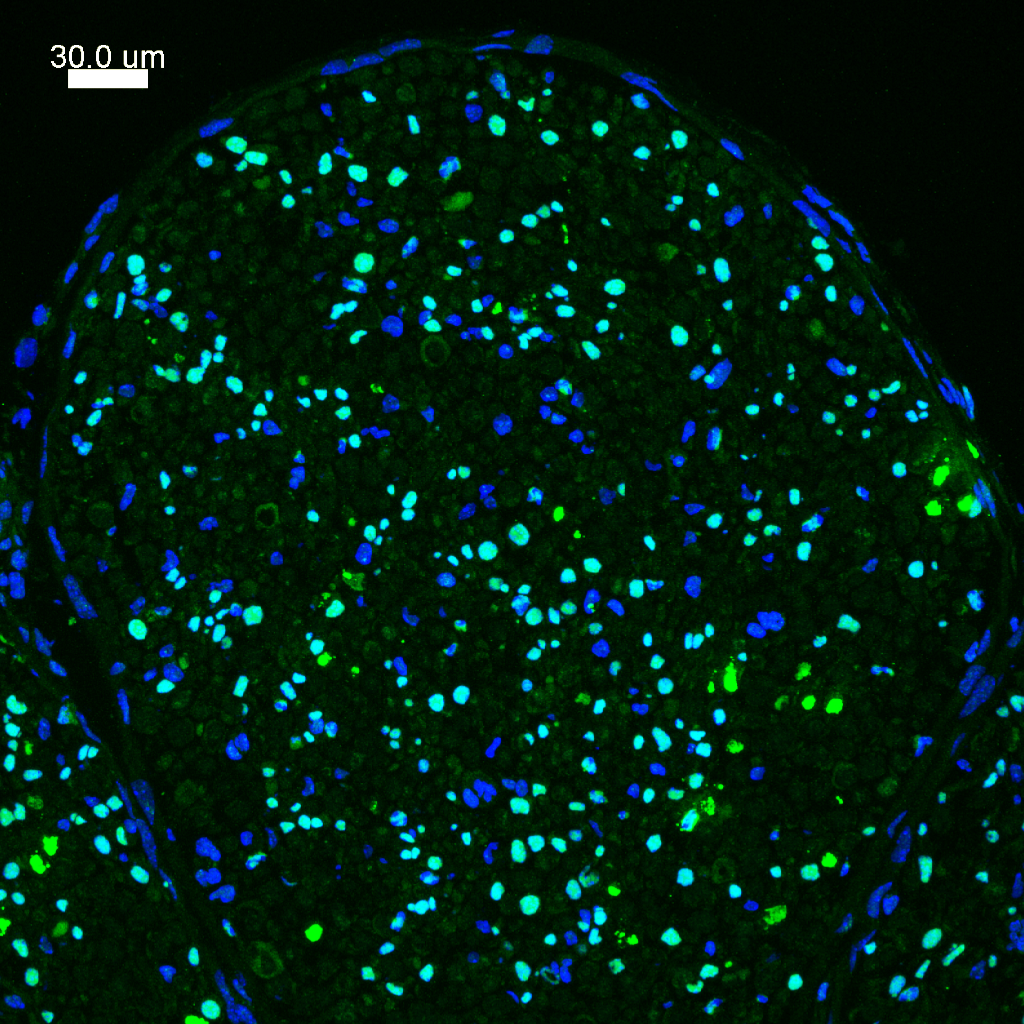

Supplement: Figure 3—source data 2. — This zip archive contains the IHC for one WT and one iDKO used for quantitative analysis shown in Figure 3H. Results and quantitation shown in the Figure used BD #550609 anti-Ki67. These results were confirmed using a second antibody, Abcam #ab15580 anti-Ki67. Images using both antibodies are included in the zip archive, in the indicated folders. Leica SP8 confocal lif images were processed using Imaris software and saved as tiffs. [file elife-50138-fig3-data2.zip › Figure 3 source data 2/WT #943 Ki67/BD #550609 Ki67/XS 1b Sox10 + DAPI.tif]

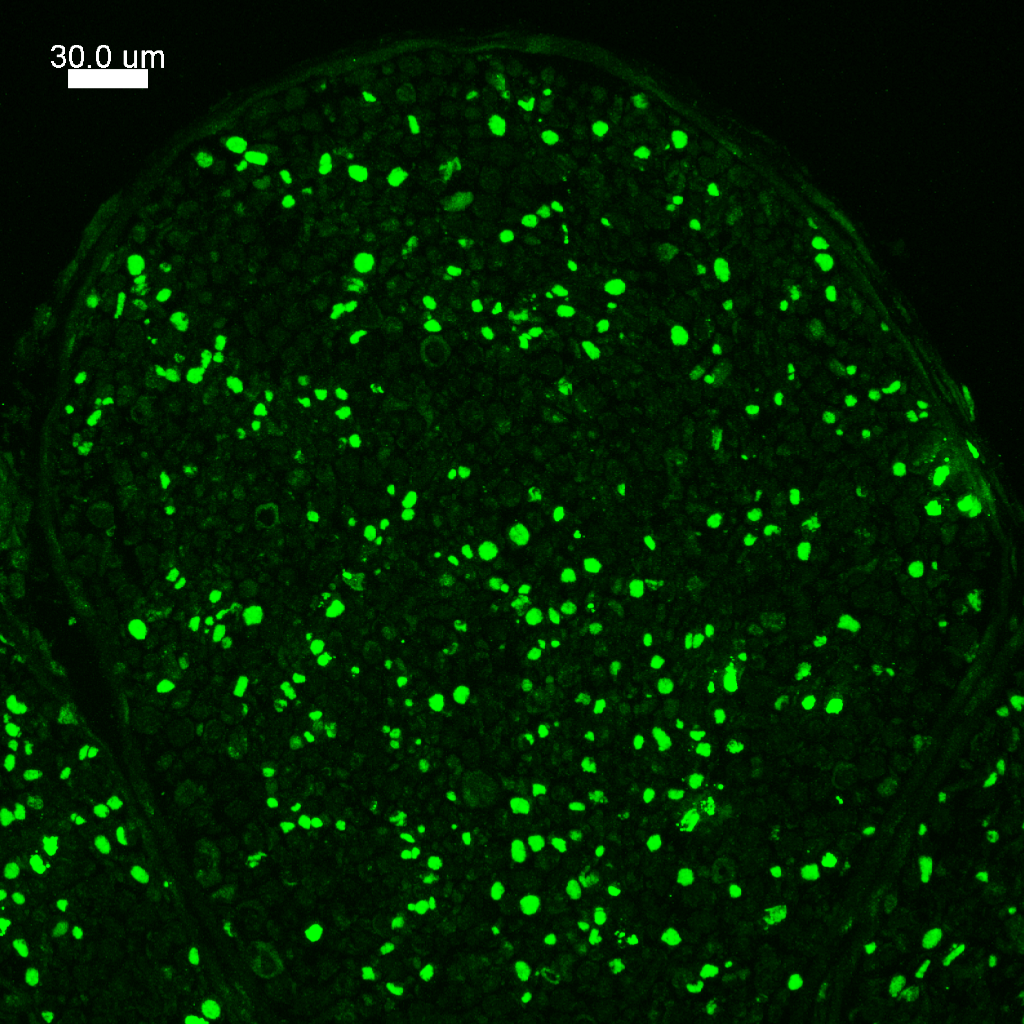

Supplement: Figure 3—source data 2. — This zip archive contains the IHC for one WT and one iDKO used for quantitative analysis shown in Figure 3H. Results and quantitation shown in the Figure used BD #550609 anti-Ki67. These results were confirmed using a second antibody, Abcam #ab15580 anti-Ki67. Images using both antibodies are included in the zip archive, in the indicated folders. Leica SP8 confocal lif images were processed using Imaris software and saved as tiffs. [file elife-50138-fig3-data2.zip › Figure 3 source data 2/WT #943 Ki67/BD #550609 Ki67/XS 1b Sox10.tif]

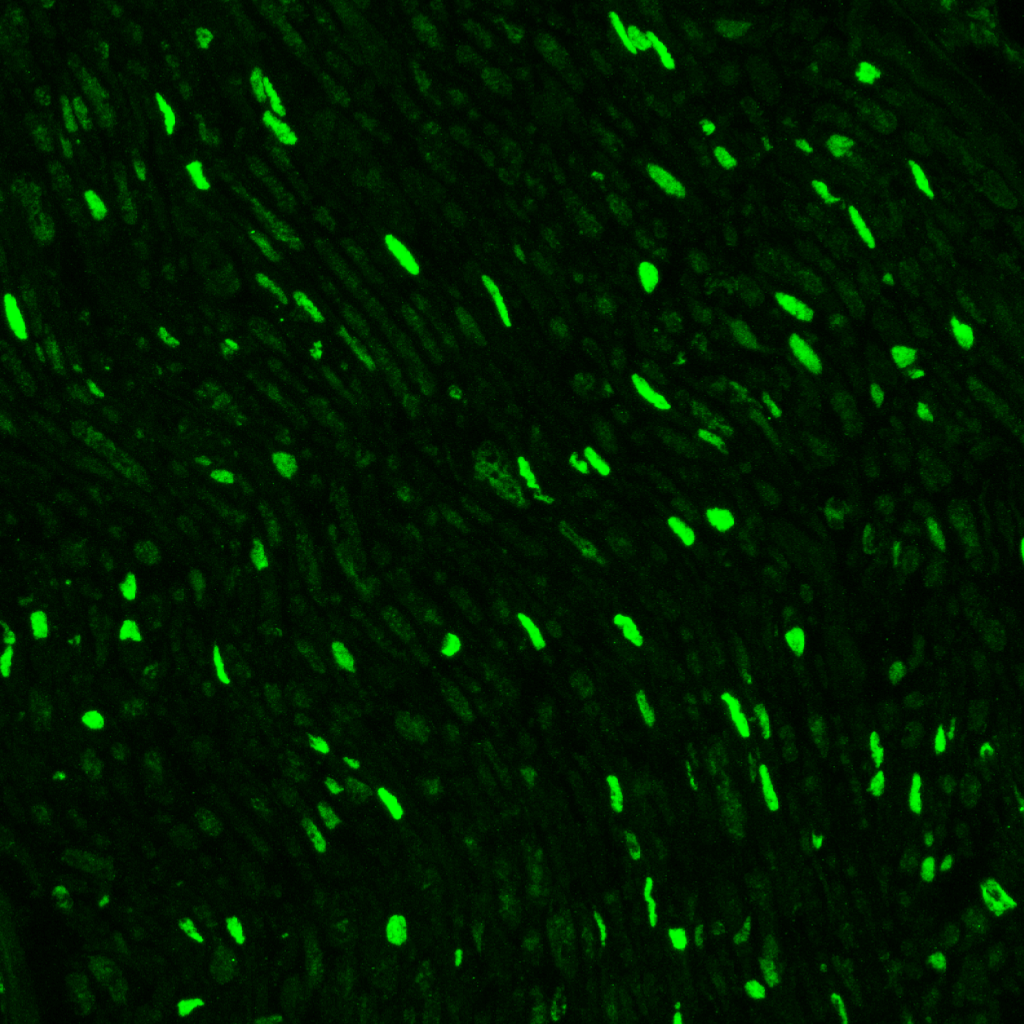

Supplement: Figure 4—source data 1. — This zip archive contains the IHC for one WT and one iDKO used for quantitative analysis shown in Figure 4E. Leica SP8 confocal lif images were processed using Imaris software and saved as tiffs. [file elife-50138-fig4-data1.zip › Figure 4 source data 1/iDKO #918 cJun/LHS a cJun.tif]

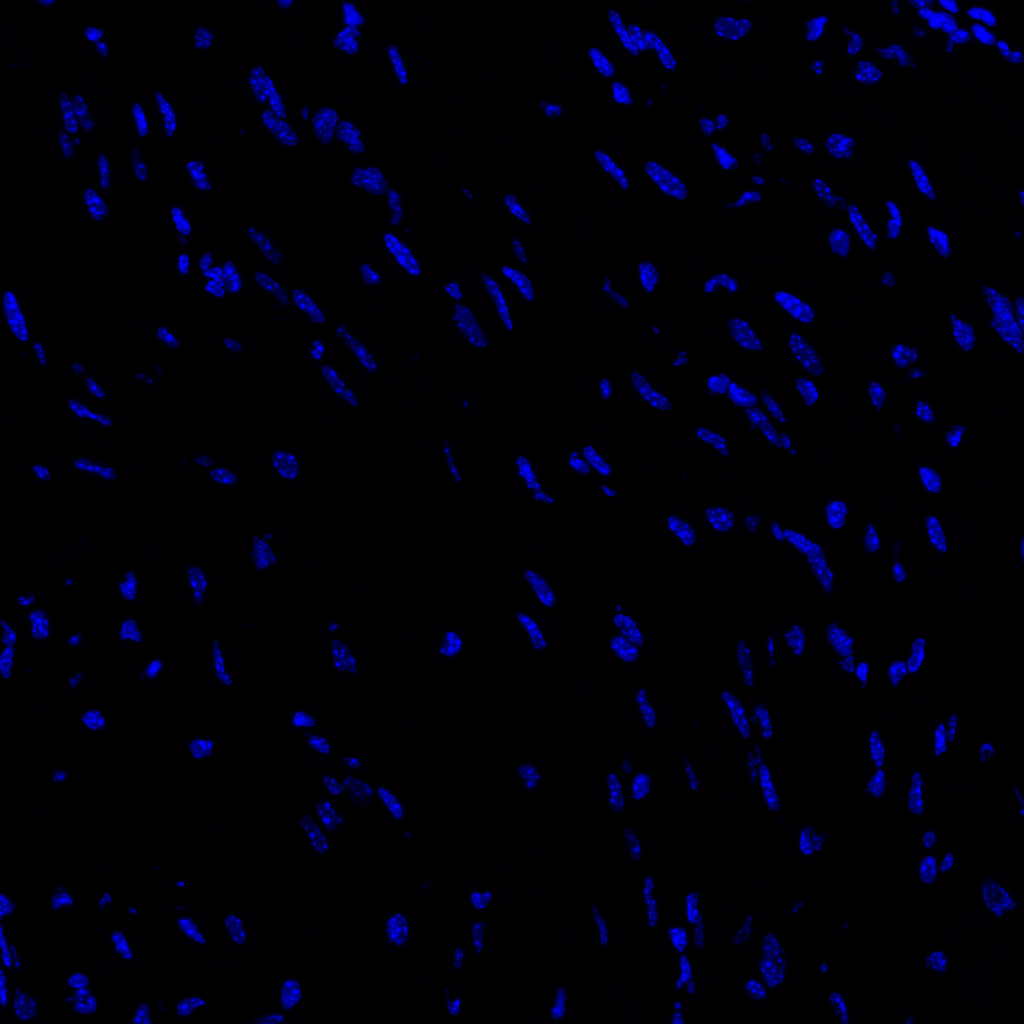

Supplement: Figure 4—source data 1. — This zip archive contains the IHC for one WT and one iDKO used for quantitative analysis shown in Figure 4E. Leica SP8 confocal lif images were processed using Imaris software and saved as tiffs. [file elife-50138-fig4-data1.zip › Figure 4 source data 1/iDKO #918 cJun/LHS a DAPI.tif]

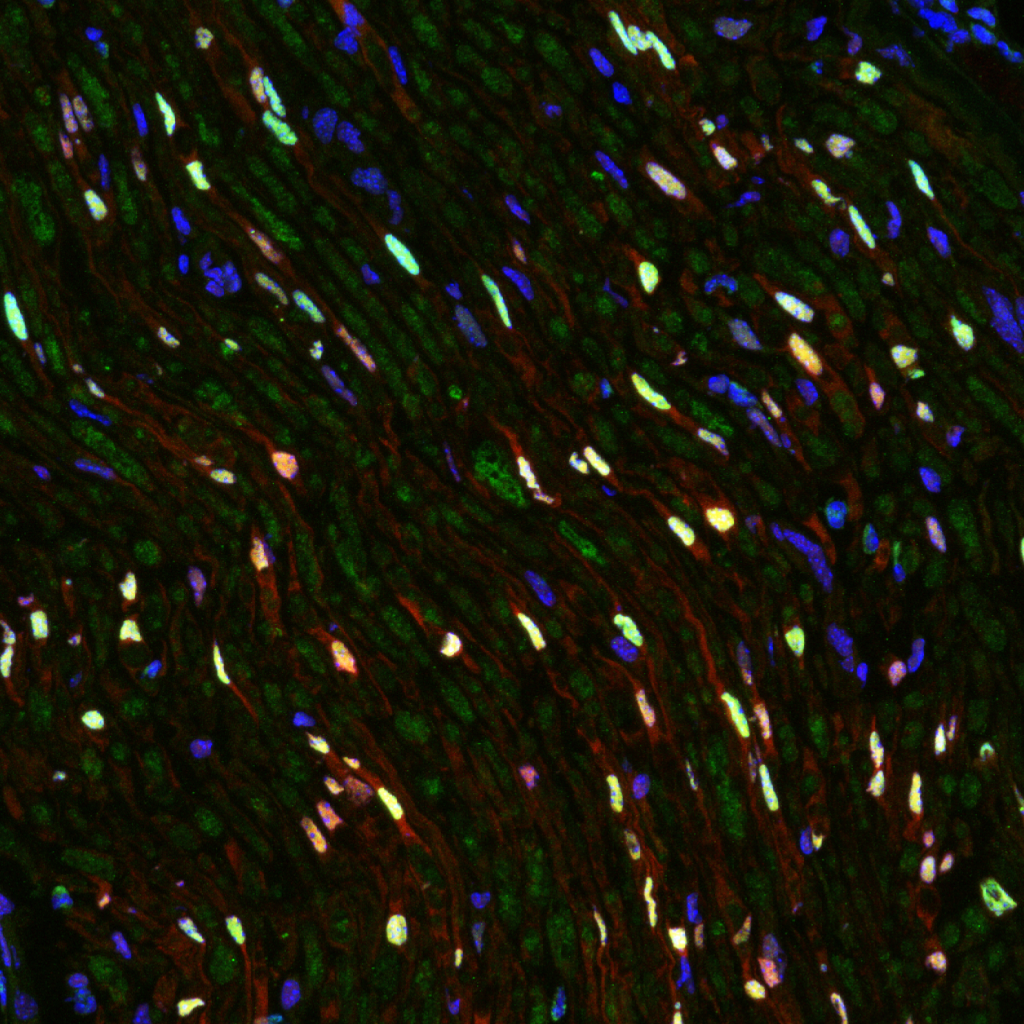

Supplement: Figure 4—source data 1. — This zip archive contains the IHC for one WT and one iDKO used for quantitative analysis shown in Figure 4E. Leica SP8 confocal lif images were processed using Imaris software and saved as tiffs. [file elife-50138-fig4-data1.zip › Figure 4 source data 1/iDKO #918 cJun/LHS a merge.tif]
