# Supplementary figures and images for: Axon-dependent expression of YAP/TAZ mediates Schwann cell remyelination but not proliferation after nerve injury (part 2 of 4)
Source: eLife. 2020 May 21;9:e50138. doi: 10.7554/eLife.50138 (PMC7259960; doi:10.7554/eLife.50138)

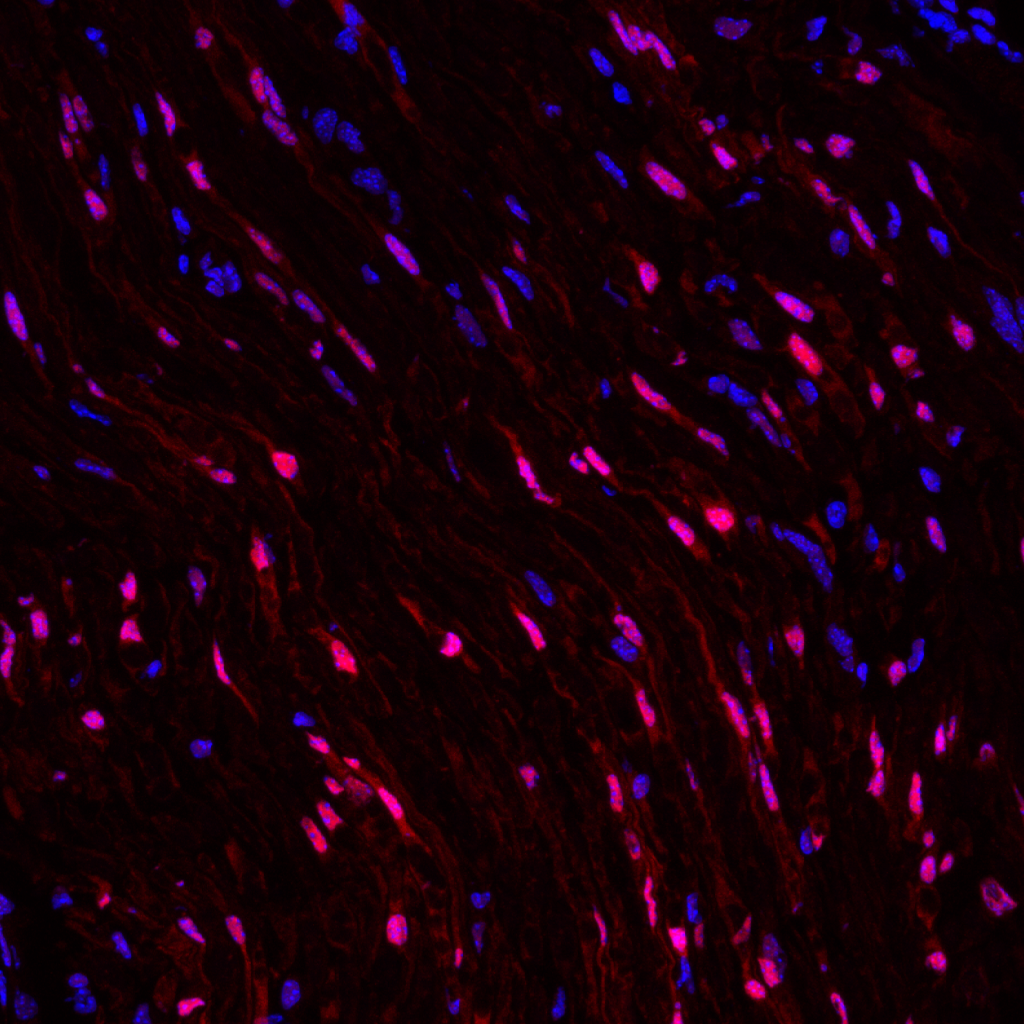

Supplement: Figure 4—source data 1. — This zip archive contains the IHC for one WT and one iDKO used for quantitative analysis shown in Figure 4E. Leica SP8 confocal lif images were processed using Imaris software and saved as tiffs. [file elife-50138-fig4-data1.zip › Figure 4 source data 1/iDKO #918 cJun/LHS a Sox10 + DAPI.tif]

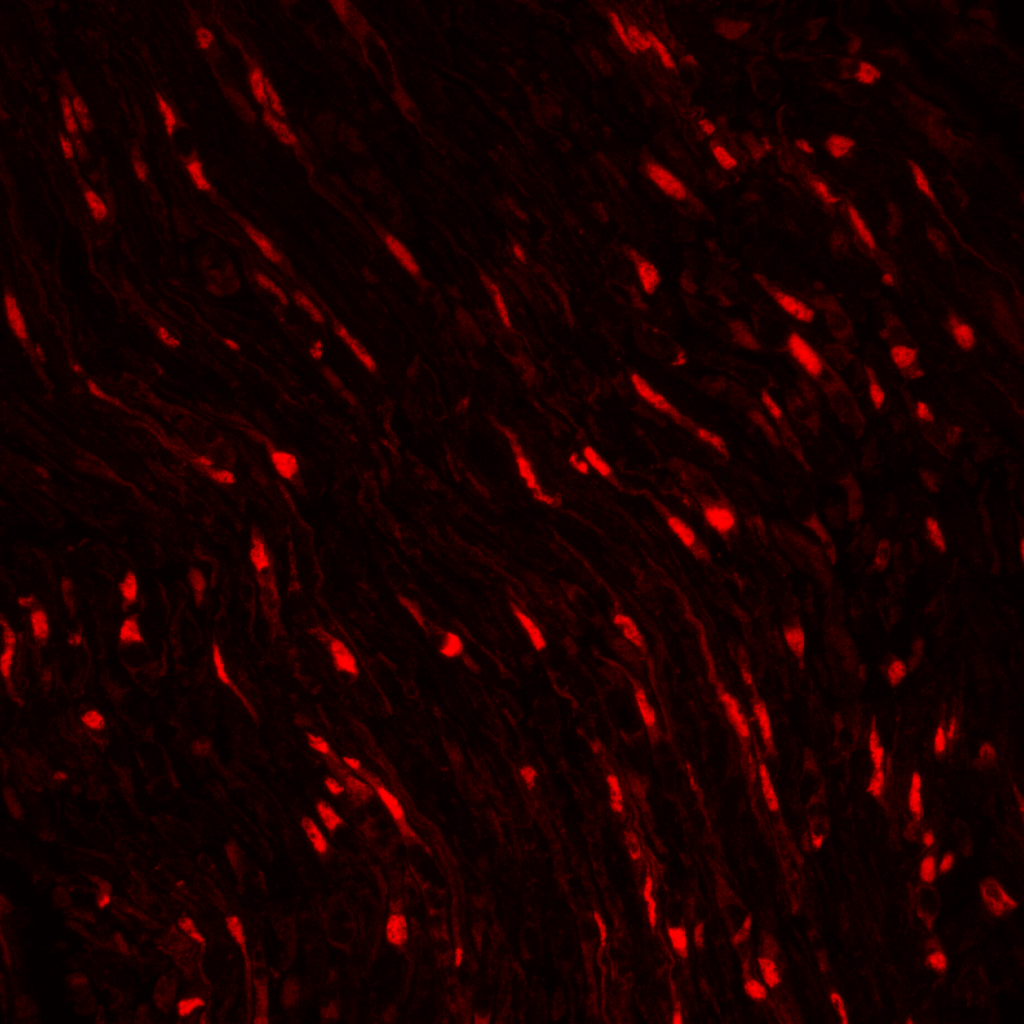

Supplement: Figure 4—source data 1. — This zip archive contains the IHC for one WT and one iDKO used for quantitative analysis shown in Figure 4E. Leica SP8 confocal lif images were processed using Imaris software and saved as tiffs. [file elife-50138-fig4-data1.zip › Figure 4 source data 1/iDKO #918 cJun/LHS a Sox10.tif]

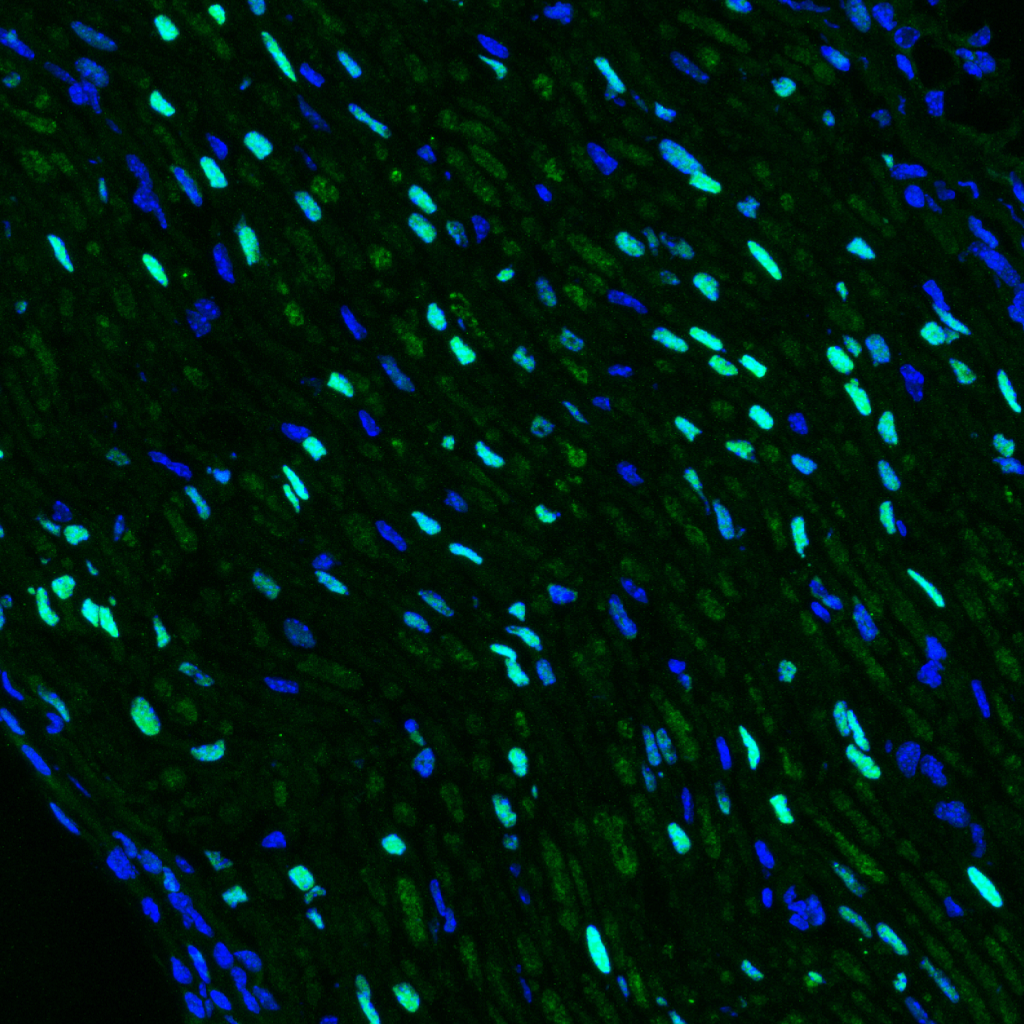

Supplement: Figure 4—source data 1. — This zip archive contains the IHC for one WT and one iDKO used for quantitative analysis shown in Figure 4E. Leica SP8 confocal lif images were processed using Imaris software and saved as tiffs. [file elife-50138-fig4-data1.zip › Figure 4 source data 1/iDKO #918 cJun/LHS b cJun + DAPI.tif]

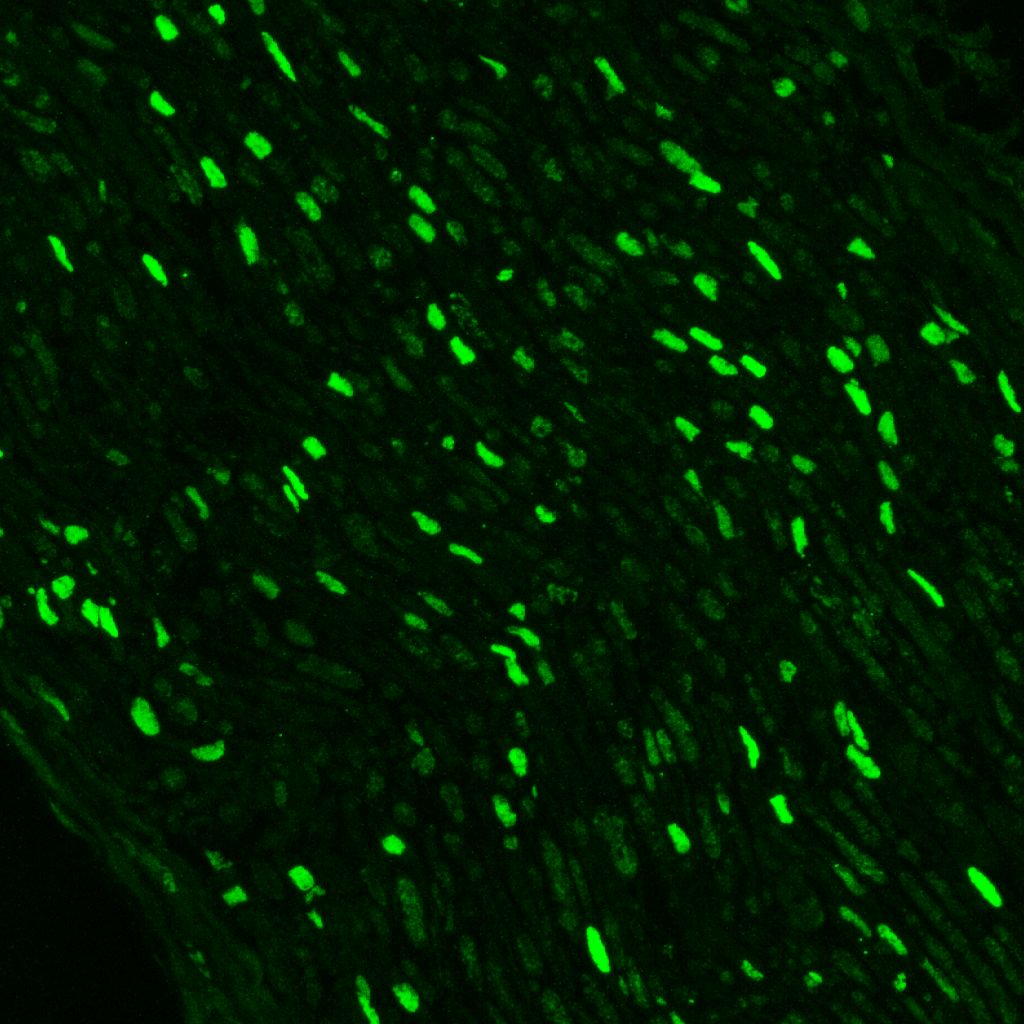

Supplement: Figure 4—source data 1. — This zip archive contains the IHC for one WT and one iDKO used for quantitative analysis shown in Figure 4E. Leica SP8 confocal lif images were processed using Imaris software and saved as tiffs. [file elife-50138-fig4-data1.zip › Figure 4 source data 1/iDKO #918 cJun/LHS b cJun.tif]

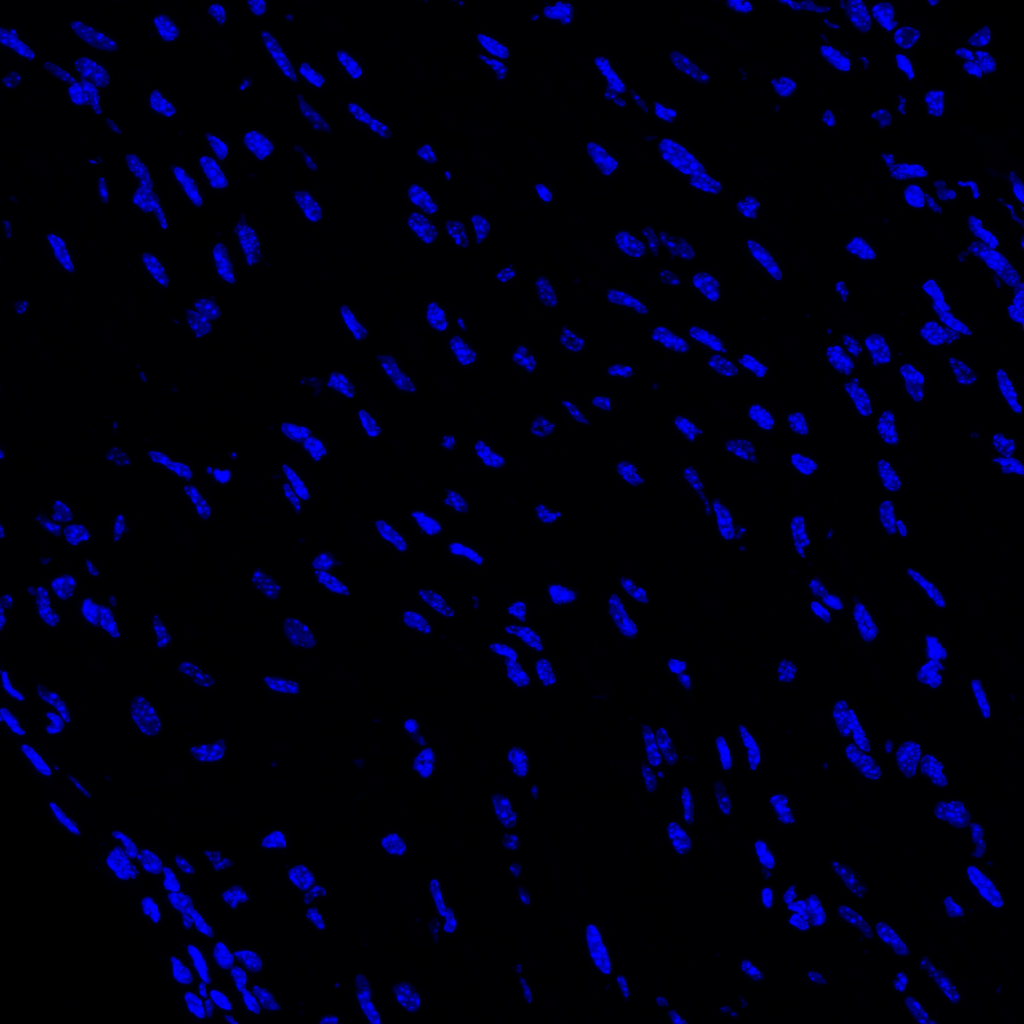

Supplement: Figure 4—source data 1. — This zip archive contains the IHC for one WT and one iDKO used for quantitative analysis shown in Figure 4E. Leica SP8 confocal lif images were processed using Imaris software and saved as tiffs. [file elife-50138-fig4-data1.zip › Figure 4 source data 1/iDKO #918 cJun/LHS b DAPI.tif]

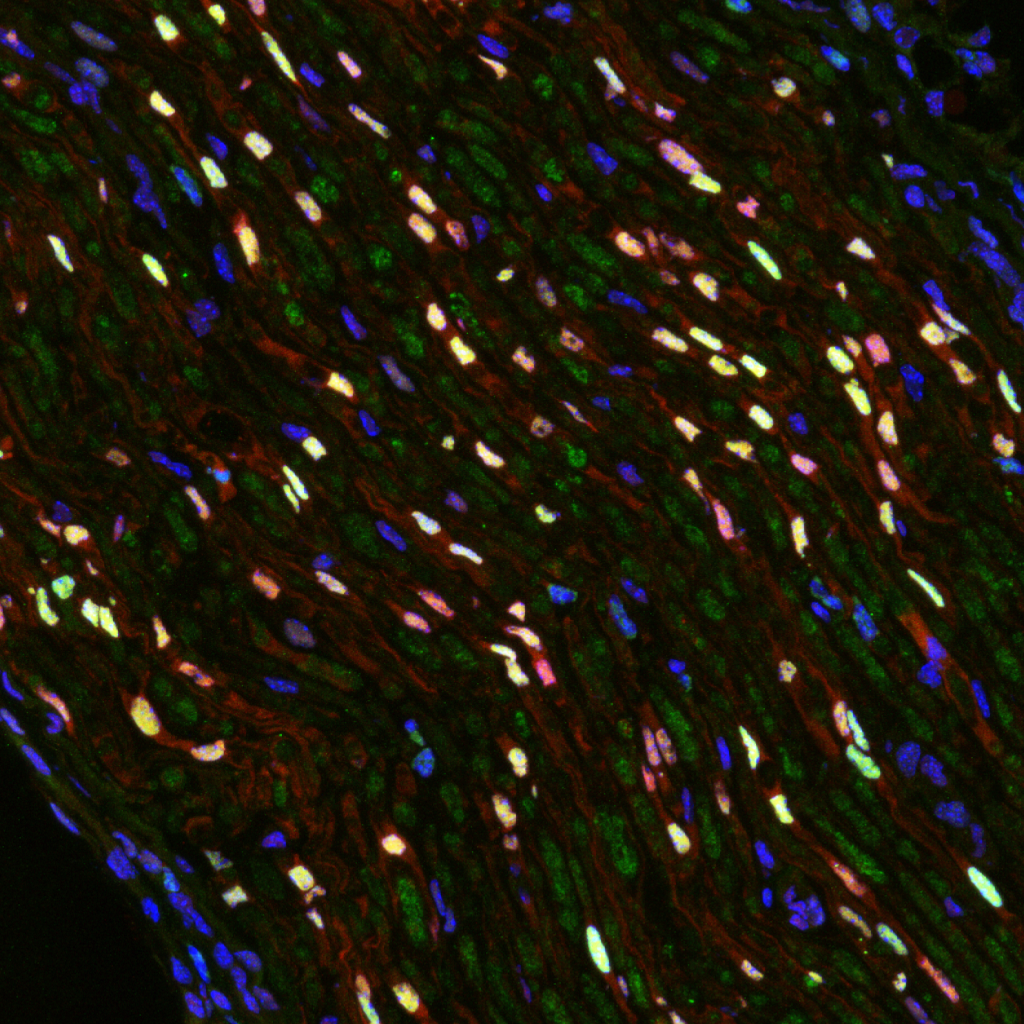

Supplement: Figure 4—source data 1. — This zip archive contains the IHC for one WT and one iDKO used for quantitative analysis shown in Figure 4E. Leica SP8 confocal lif images were processed using Imaris software and saved as tiffs. [file elife-50138-fig4-data1.zip › Figure 4 source data 1/iDKO #918 cJun/LHS b merge.tif]

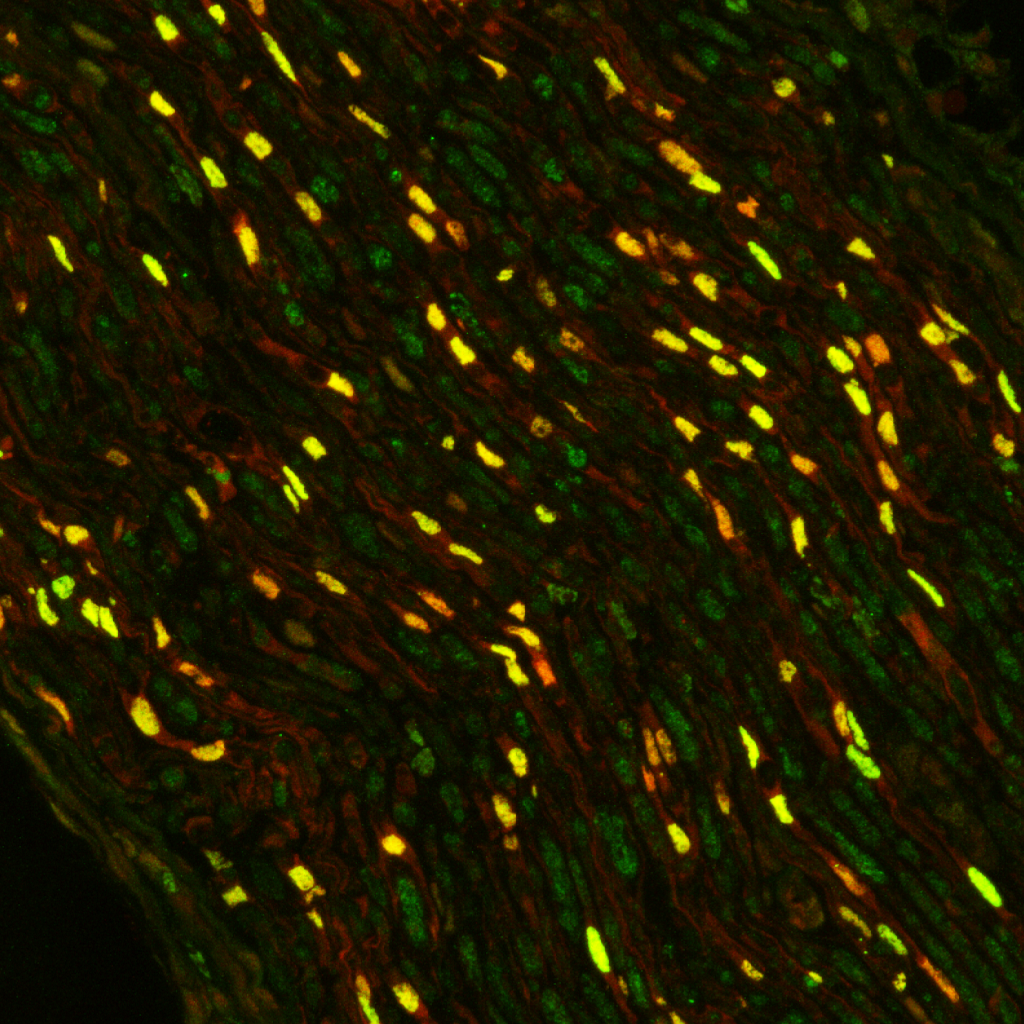

Supplement: Figure 4—source data 1. — This zip archive contains the IHC for one WT and one iDKO used for quantitative analysis shown in Figure 4E. Leica SP8 confocal lif images were processed using Imaris software and saved as tiffs. [file elife-50138-fig4-data1.zip › Figure 4 source data 1/iDKO #918 cJun/LHS b Sox10 + cJun.tif]

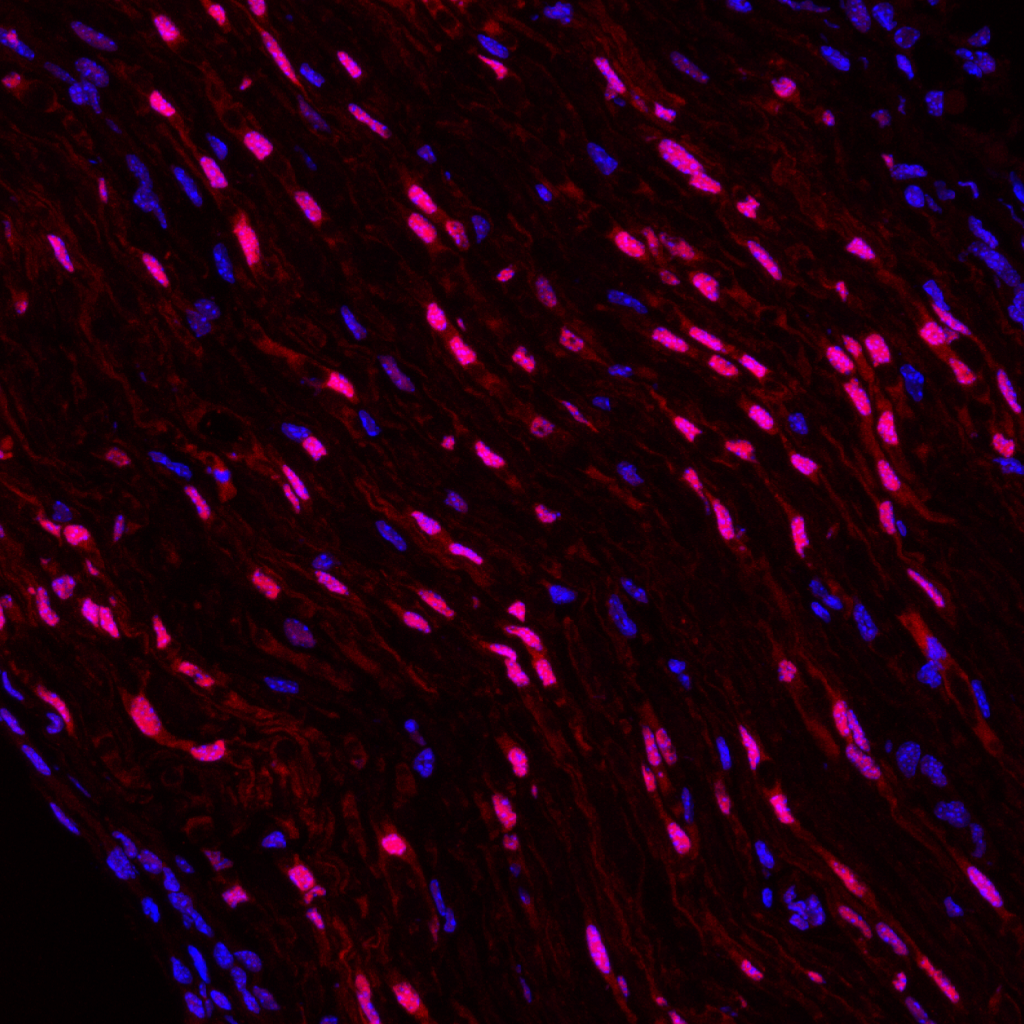

Supplement: Figure 4—source data 1. — This zip archive contains the IHC for one WT and one iDKO used for quantitative analysis shown in Figure 4E. Leica SP8 confocal lif images were processed using Imaris software and saved as tiffs. [file elife-50138-fig4-data1.zip › Figure 4 source data 1/iDKO #918 cJun/LHS b Sox10 + DAPI.tif]

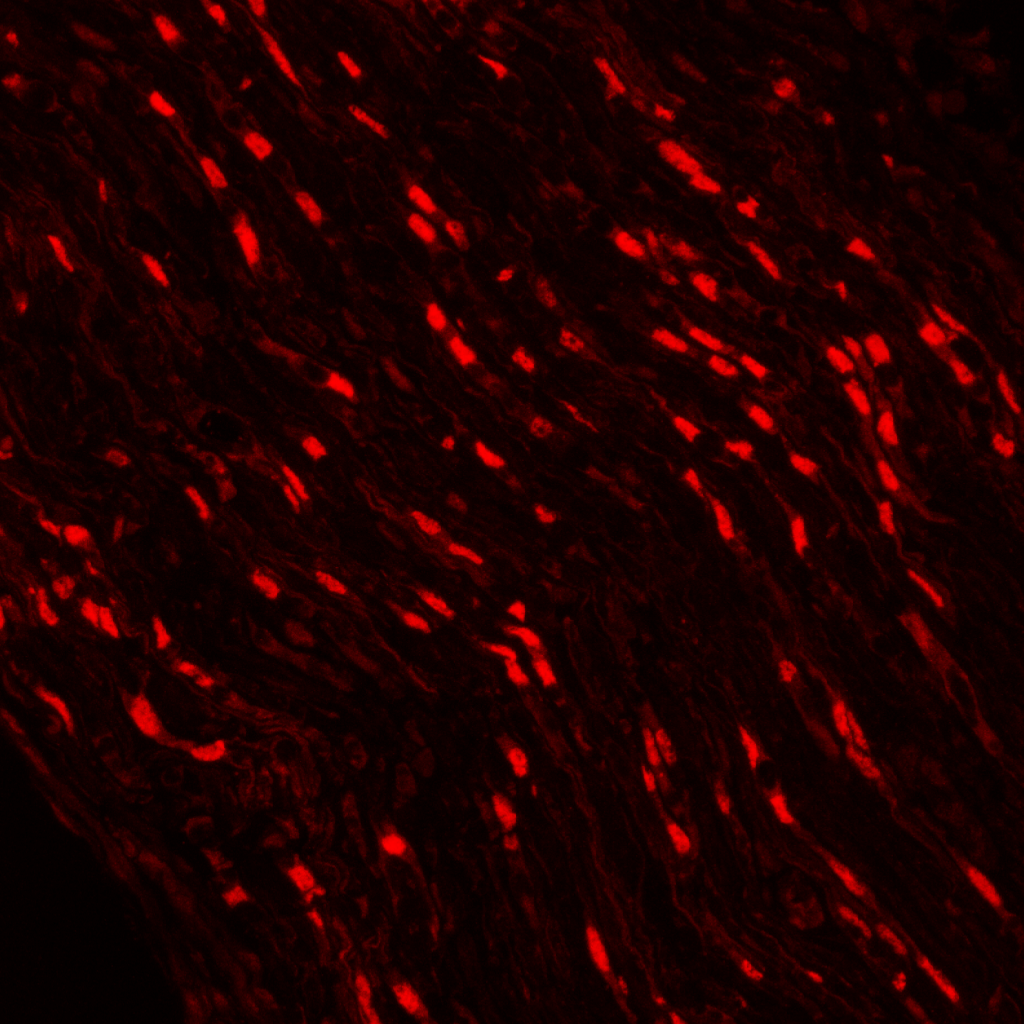

Supplement: Figure 4—source data 1. — This zip archive contains the IHC for one WT and one iDKO used for quantitative analysis shown in Figure 4E. Leica SP8 confocal lif images were processed using Imaris software and saved as tiffs. [file elife-50138-fig4-data1.zip › Figure 4 source data 1/iDKO #918 cJun/LHS b Sox10.tif]

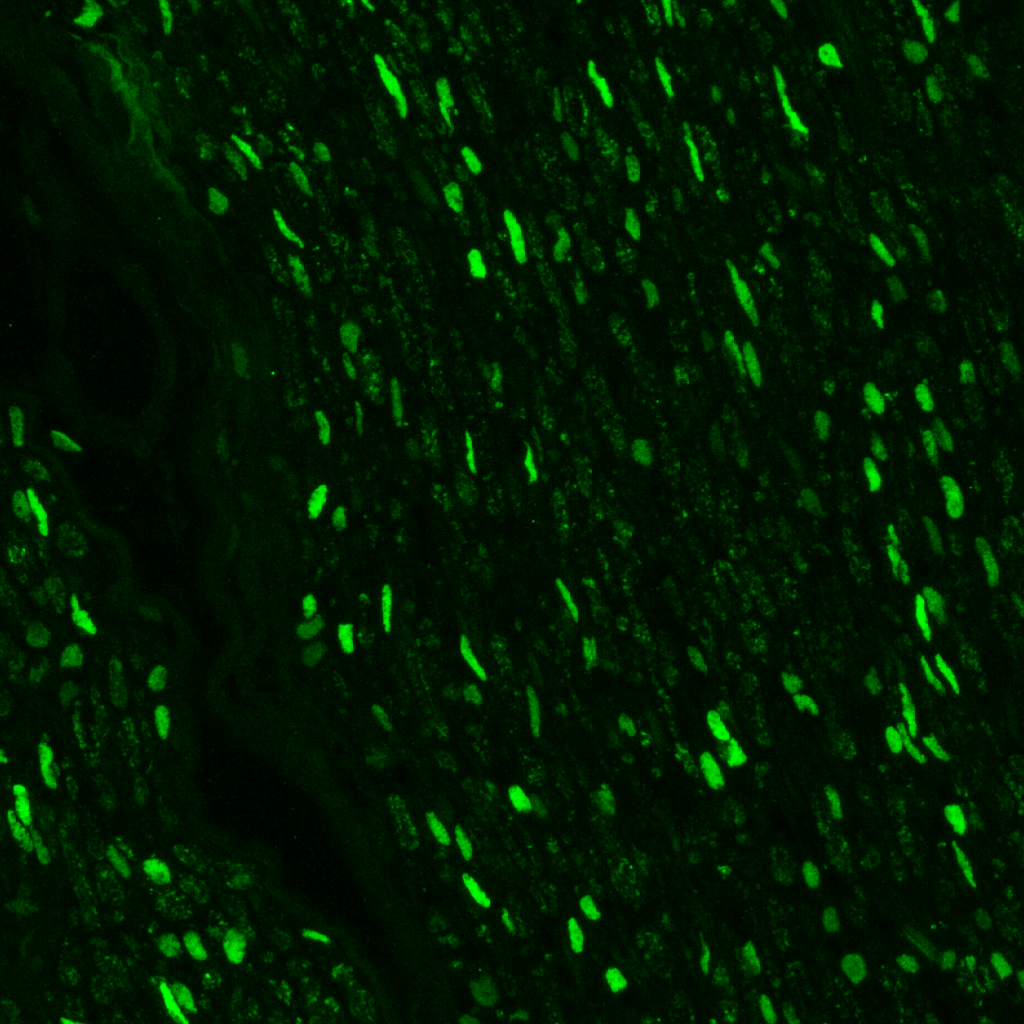

Supplement: Figure 4—source data 1. — This zip archive contains the IHC for one WT and one iDKO used for quantitative analysis shown in Figure 4E. Leica SP8 confocal lif images were processed using Imaris software and saved as tiffs. [file elife-50138-fig4-data1.zip › Figure 4 source data 1/iDKO #918 cJun/LHS c cJun.tif]

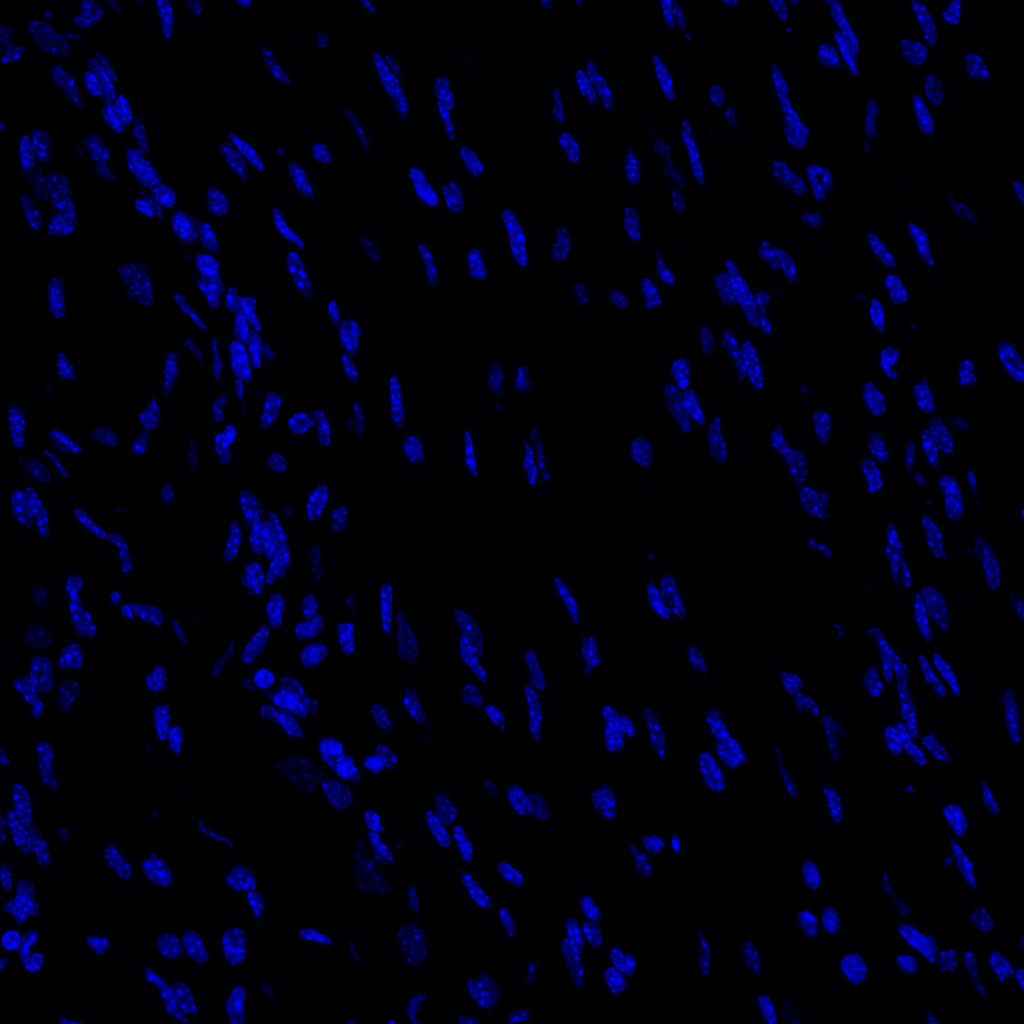

Supplement: Figure 4—source data 1. — This zip archive contains the IHC for one WT and one iDKO used for quantitative analysis shown in Figure 4E. Leica SP8 confocal lif images were processed using Imaris software and saved as tiffs. [file elife-50138-fig4-data1.zip › Figure 4 source data 1/iDKO #918 cJun/LHS c DAPI.tif]

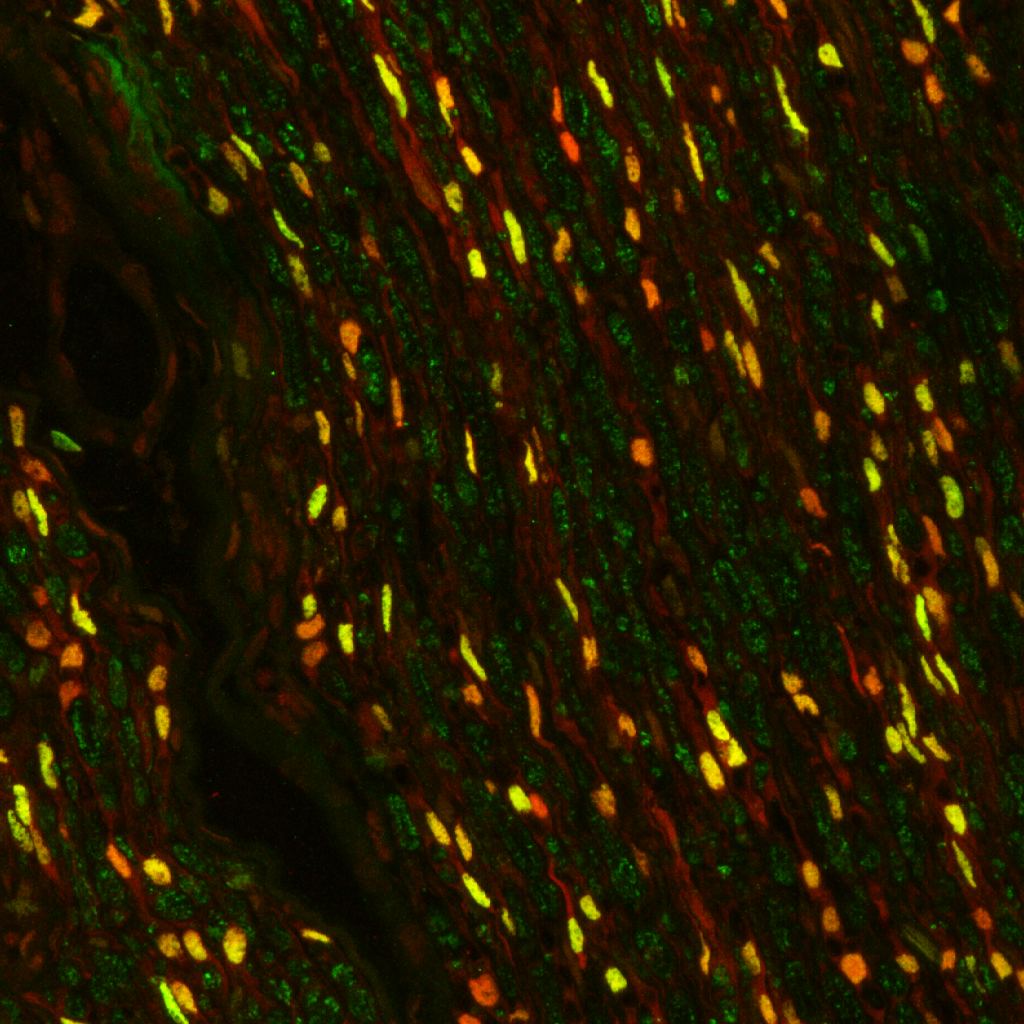

Supplement: Figure 4—source data 1. — This zip archive contains the IHC for one WT and one iDKO used for quantitative analysis shown in Figure 4E. Leica SP8 confocal lif images were processed using Imaris software and saved as tiffs. [file elife-50138-fig4-data1.zip › Figure 4 source data 1/iDKO #918 cJun/LHS c Sox10 + cJun.tif]

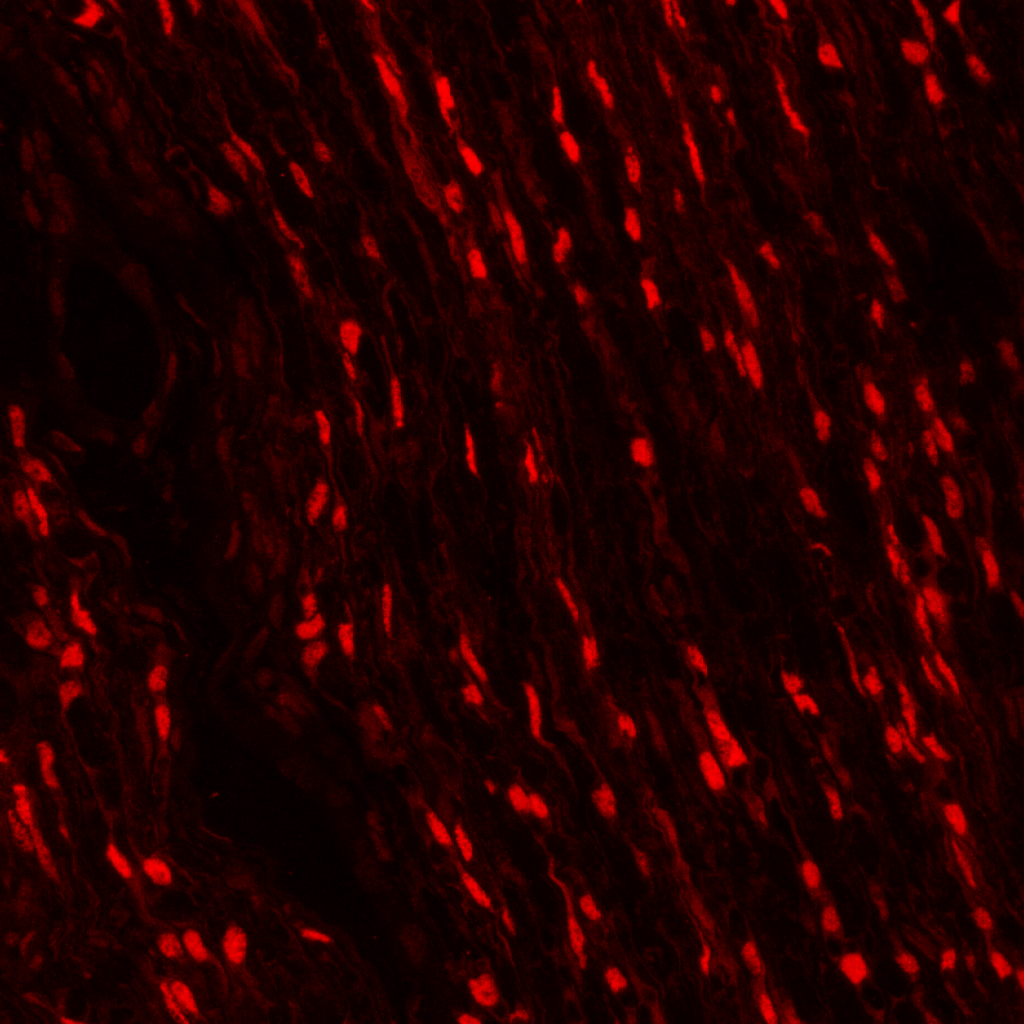

Supplement: Figure 4—source data 1. — This zip archive contains the IHC for one WT and one iDKO used for quantitative analysis shown in Figure 4E. Leica SP8 confocal lif images were processed using Imaris software and saved as tiffs. [file elife-50138-fig4-data1.zip › Figure 4 source data 1/iDKO #918 cJun/LHS c Sox10.tif]

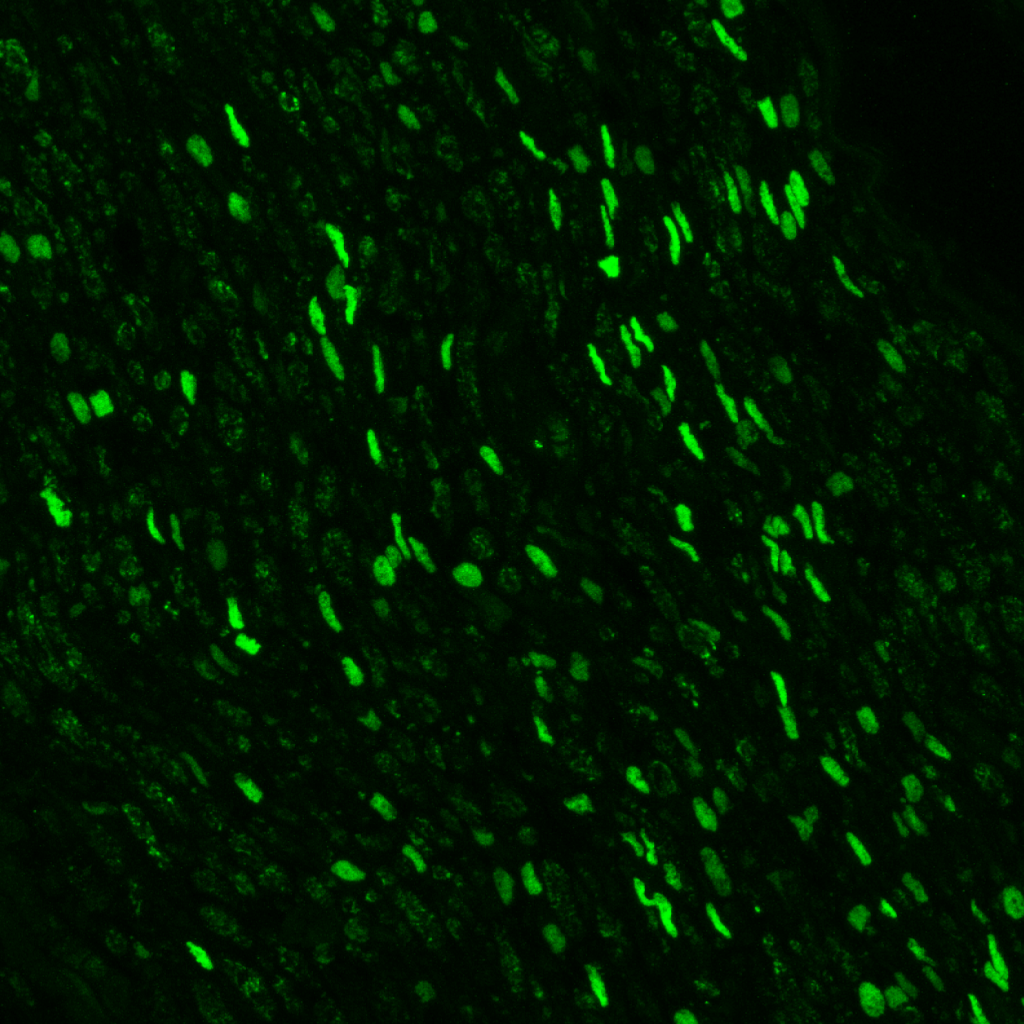

Supplement: Figure 4—source data 1. — This zip archive contains the IHC for one WT and one iDKO used for quantitative analysis shown in Figure 4E. Leica SP8 confocal lif images were processed using Imaris software and saved as tiffs. [file elife-50138-fig4-data1.zip › Figure 4 source data 1/iDKO #918 cJun/LHS d cJun.tif]

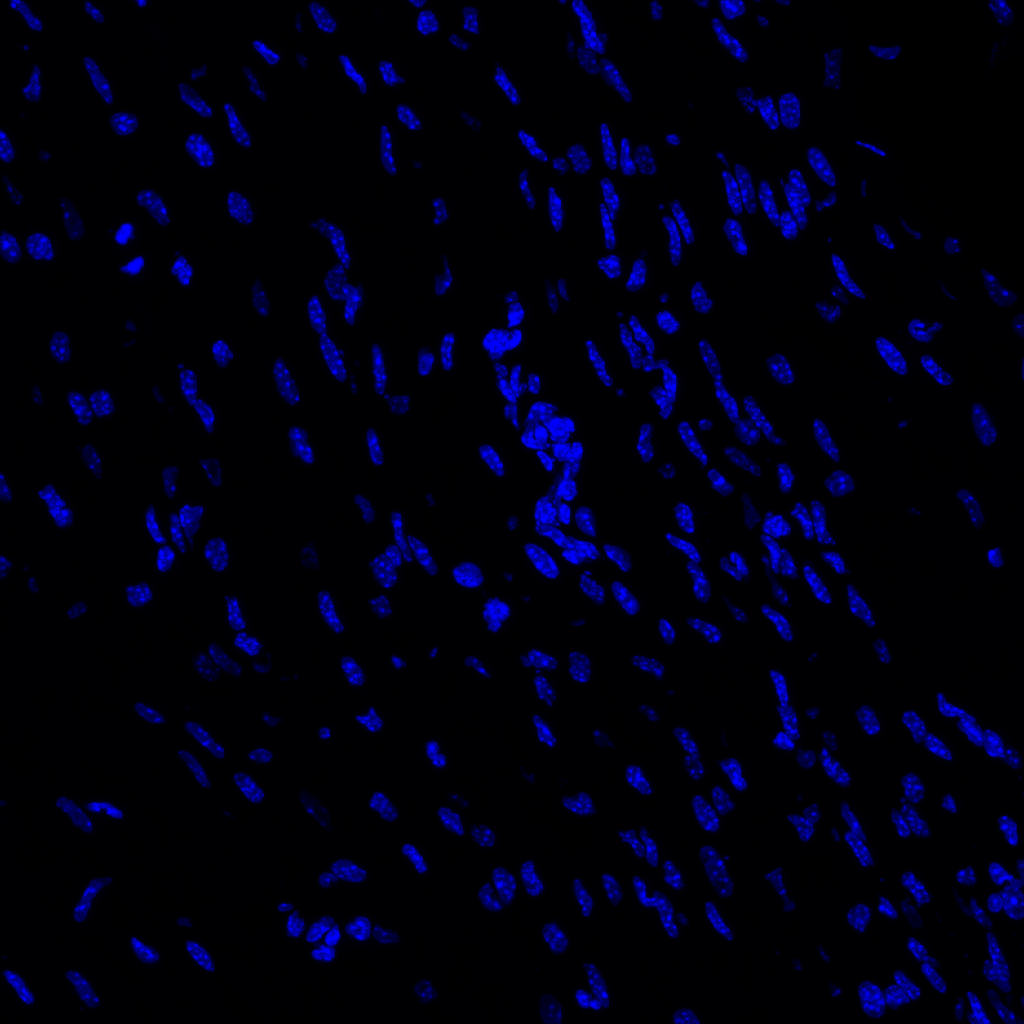

Supplement: Figure 4—source data 1. — This zip archive contains the IHC for one WT and one iDKO used for quantitative analysis shown in Figure 4E. Leica SP8 confocal lif images were processed using Imaris software and saved as tiffs. [file elife-50138-fig4-data1.zip › Figure 4 source data 1/iDKO #918 cJun/LHS d DAPI.tif]

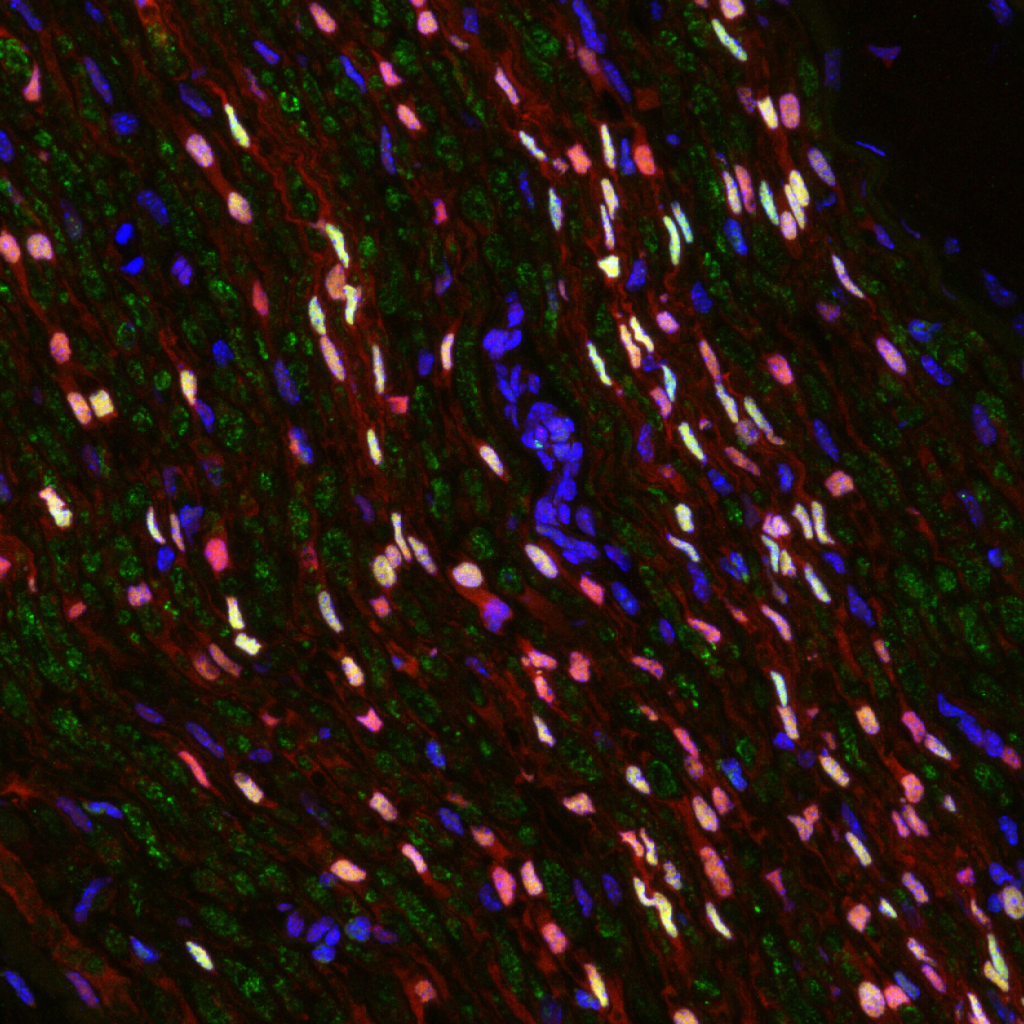

Supplement: Figure 4—source data 1. — This zip archive contains the IHC for one WT and one iDKO used for quantitative analysis shown in Figure 4E. Leica SP8 confocal lif images were processed using Imaris software and saved as tiffs. [file elife-50138-fig4-data1.zip › Figure 4 source data 1/iDKO #918 cJun/LHS d merge.tif]

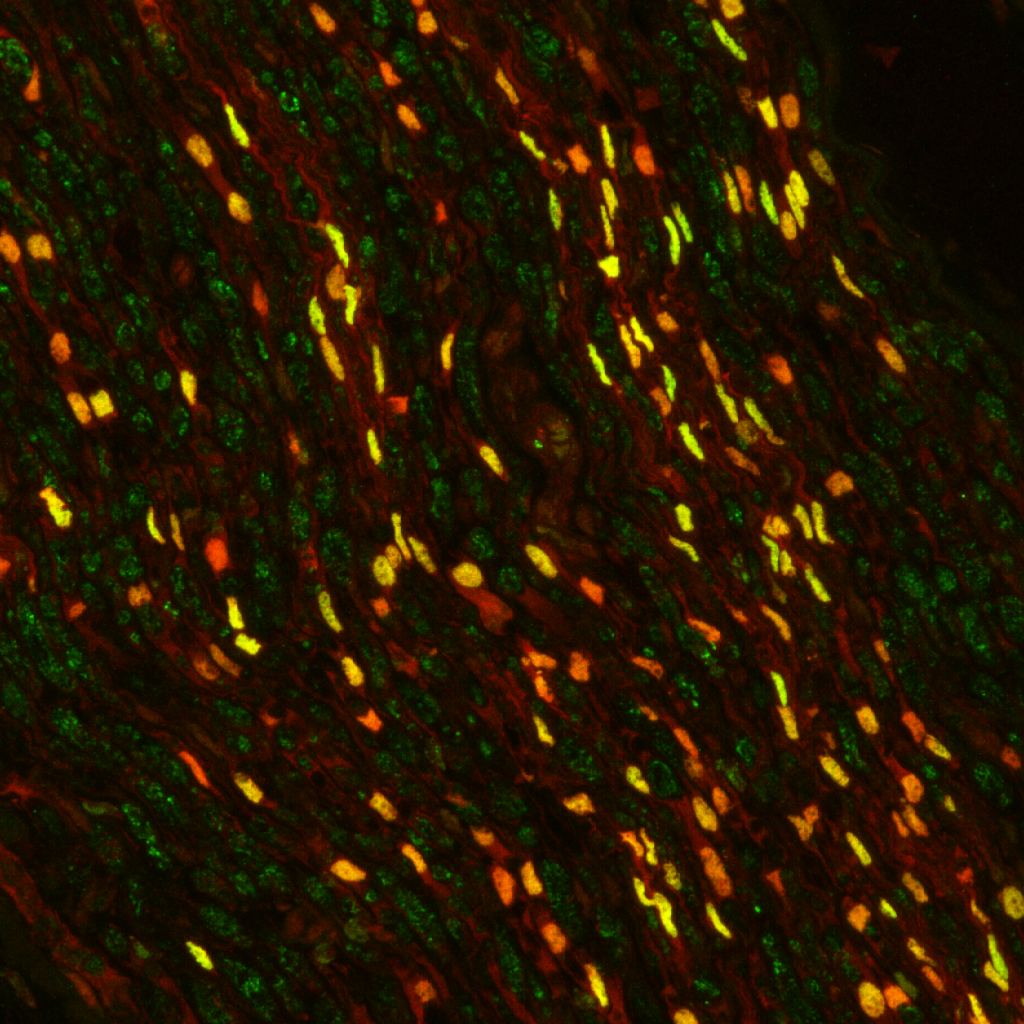

Supplement: Figure 4—source data 1. — This zip archive contains the IHC for one WT and one iDKO used for quantitative analysis shown in Figure 4E. Leica SP8 confocal lif images were processed using Imaris software and saved as tiffs. [file elife-50138-fig4-data1.zip › Figure 4 source data 1/iDKO #918 cJun/LHS d Sox10 + cJun.tif]

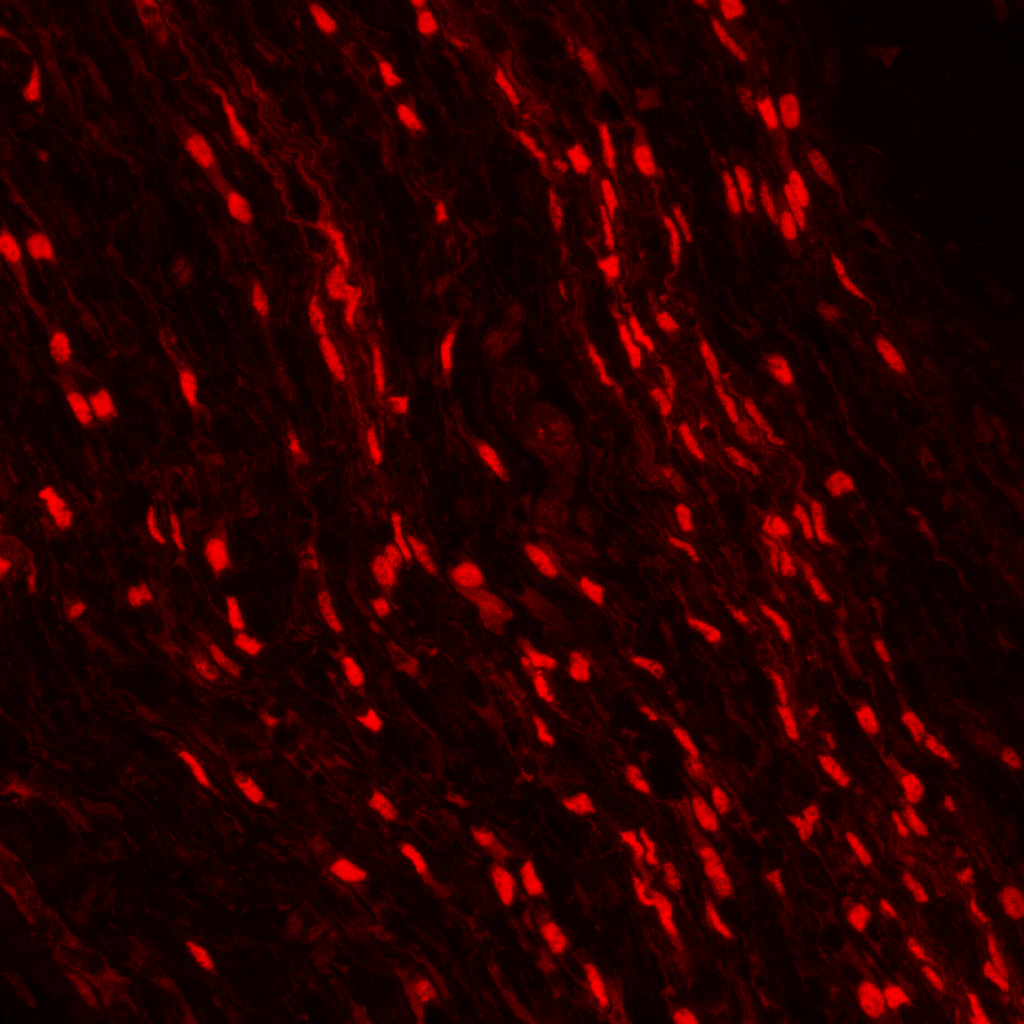

Supplement: Figure 4—source data 1. — This zip archive contains the IHC for one WT and one iDKO used for quantitative analysis shown in Figure 4E. Leica SP8 confocal lif images were processed using Imaris software and saved as tiffs. [file elife-50138-fig4-data1.zip › Figure 4 source data 1/iDKO #918 cJun/LHS d Sox10.tif]

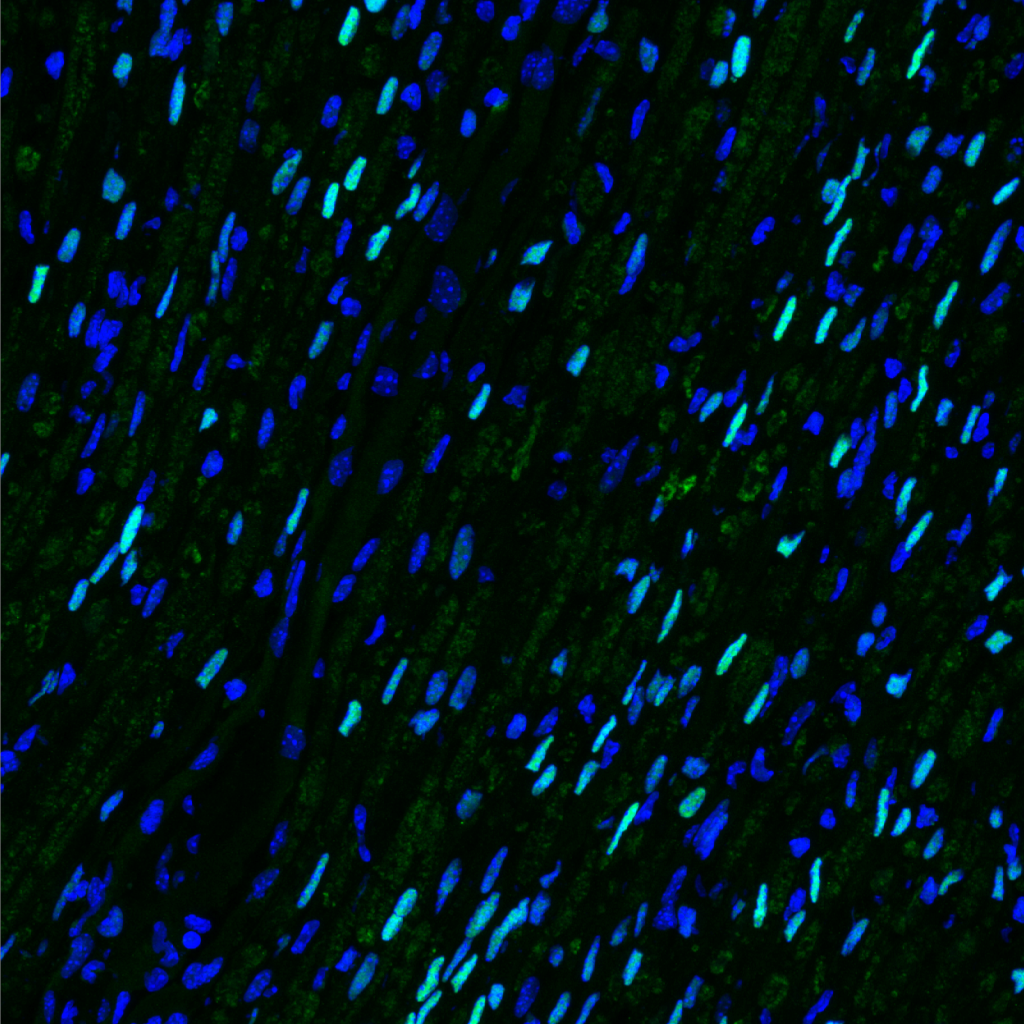

Supplement: Figure 4—source data 1. — This zip archive contains the IHC for one WT and one iDKO used for quantitative analysis shown in Figure 4E. Leica SP8 confocal lif images were processed using Imaris software and saved as tiffs. [file elife-50138-fig4-data1.zip › Figure 4 source data 1/WT #579 cJun/RHS a cJun + DAPI.tif]

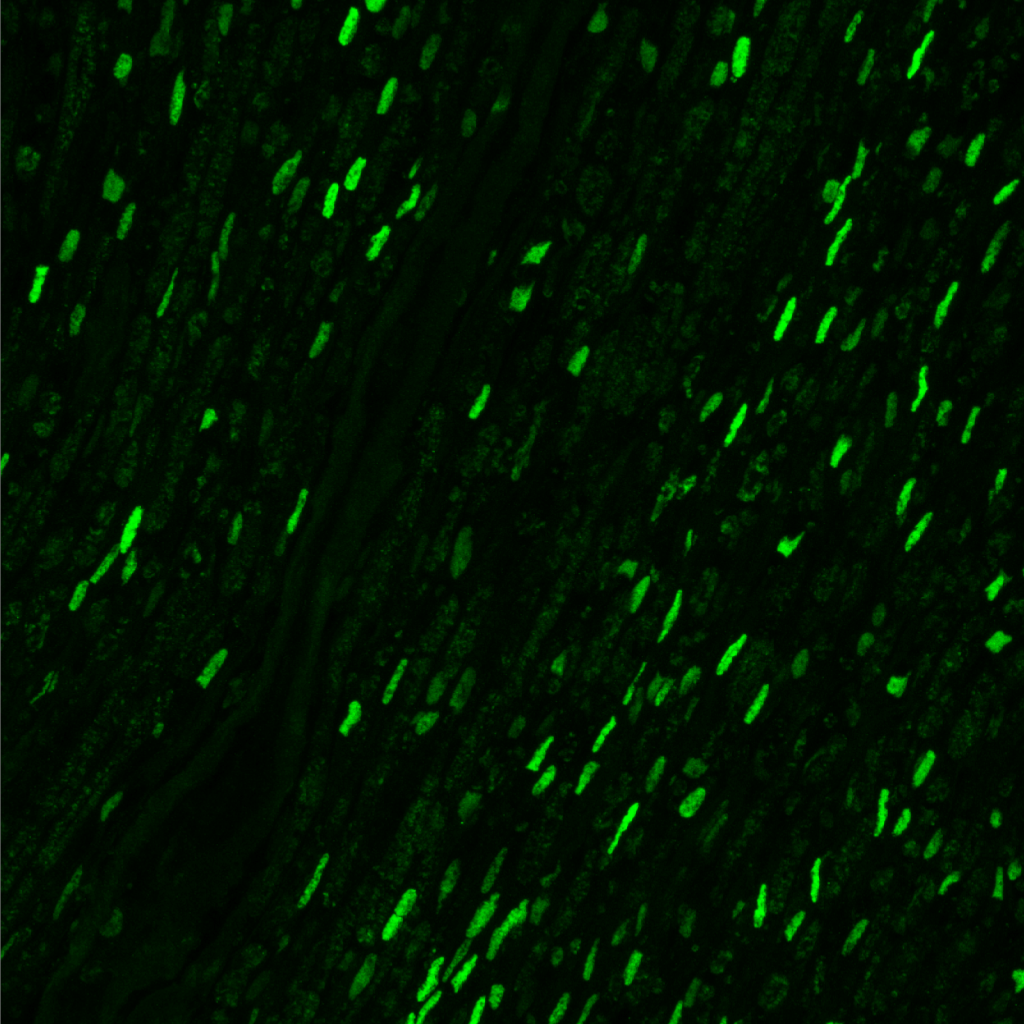

Supplement: Figure 4—source data 1. — This zip archive contains the IHC for one WT and one iDKO used for quantitative analysis shown in Figure 4E. Leica SP8 confocal lif images were processed using Imaris software and saved as tiffs. [file elife-50138-fig4-data1.zip › Figure 4 source data 1/WT #579 cJun/RHS a cJun.tif]

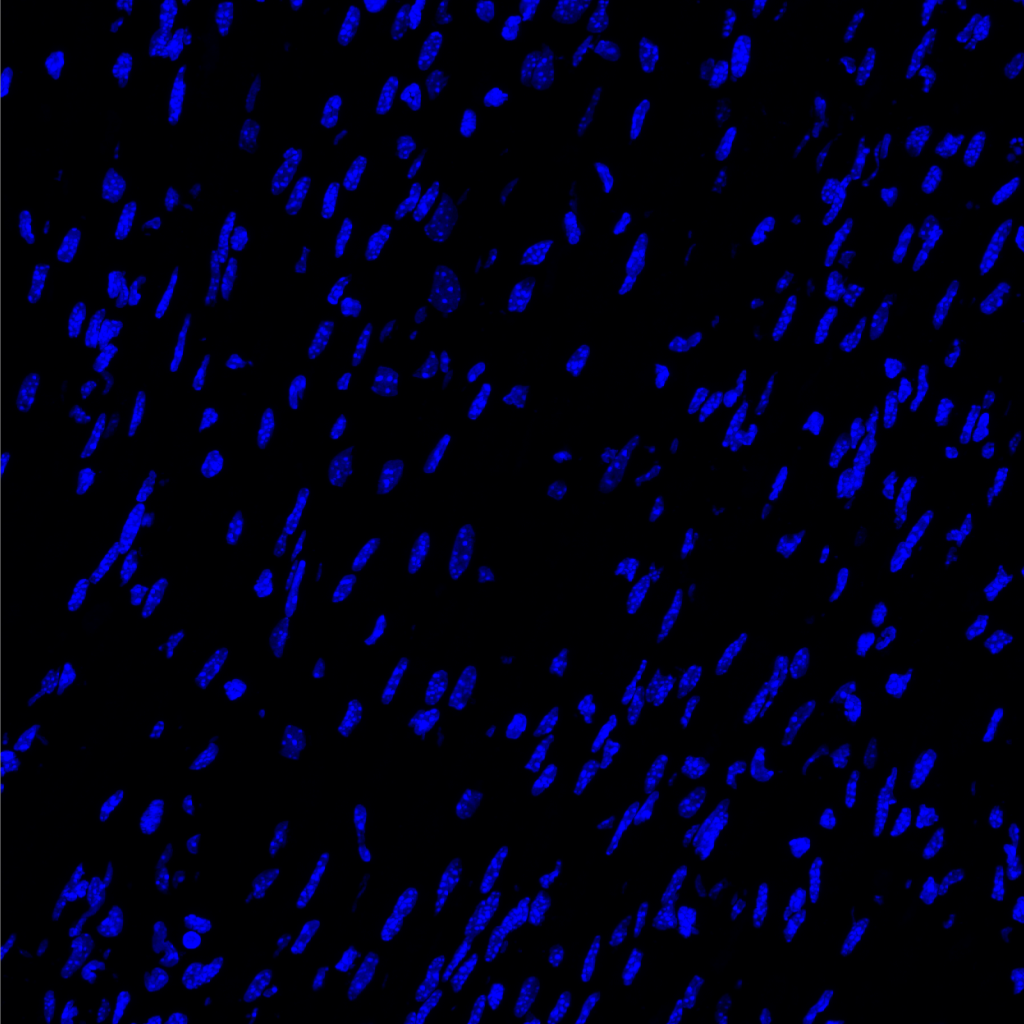

Supplement: Figure 4—source data 1. — This zip archive contains the IHC for one WT and one iDKO used for quantitative analysis shown in Figure 4E. Leica SP8 confocal lif images were processed using Imaris software and saved as tiffs. [file elife-50138-fig4-data1.zip › Figure 4 source data 1/WT #579 cJun/RHS a DAPI.tif]

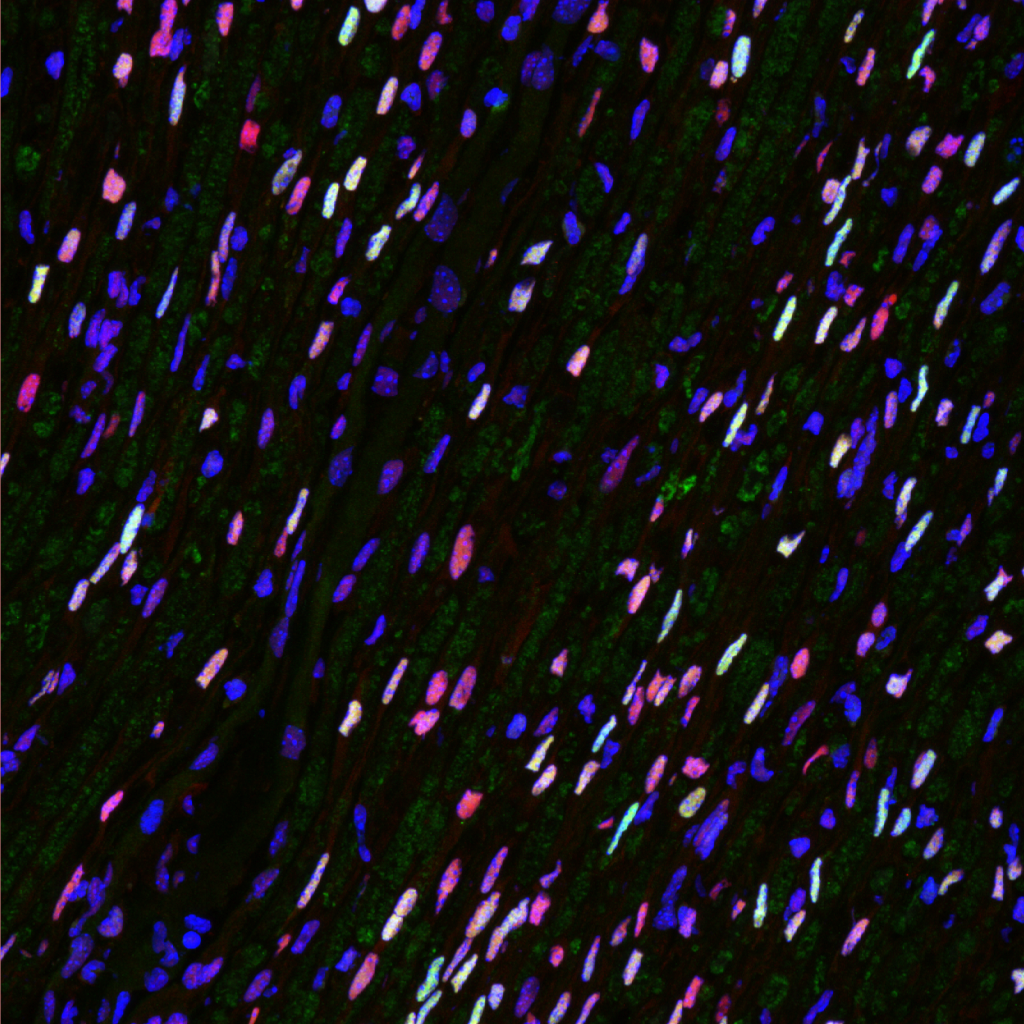

Supplement: Figure 4—source data 1. — This zip archive contains the IHC for one WT and one iDKO used for quantitative analysis shown in Figure 4E. Leica SP8 confocal lif images were processed using Imaris software and saved as tiffs. [file elife-50138-fig4-data1.zip › Figure 4 source data 1/WT #579 cJun/RHS a merge.tif]

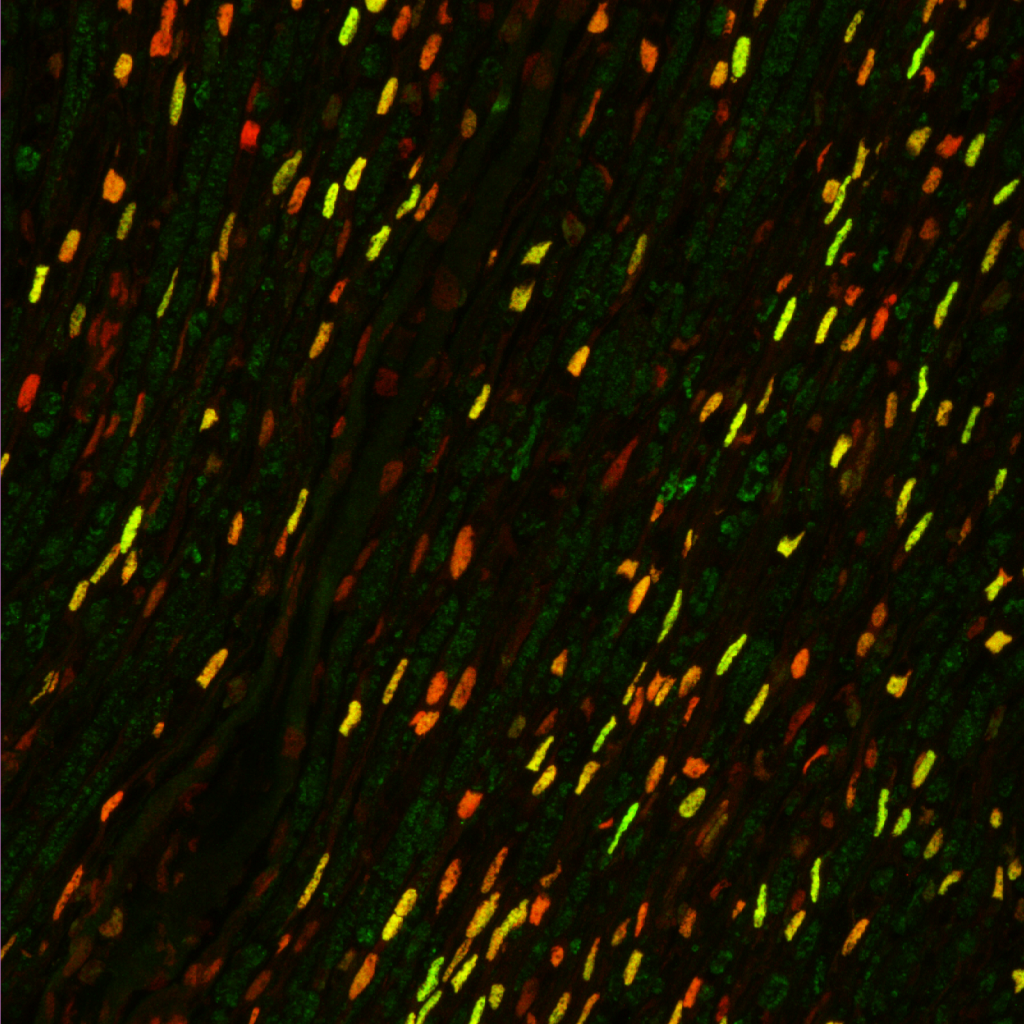

Supplement: Figure 4—source data 1. — This zip archive contains the IHC for one WT and one iDKO used for quantitative analysis shown in Figure 4E. Leica SP8 confocal lif images were processed using Imaris software and saved as tiffs. [file elife-50138-fig4-data1.zip › Figure 4 source data 1/WT #579 cJun/RHS a Sox10 + cJun.tif]

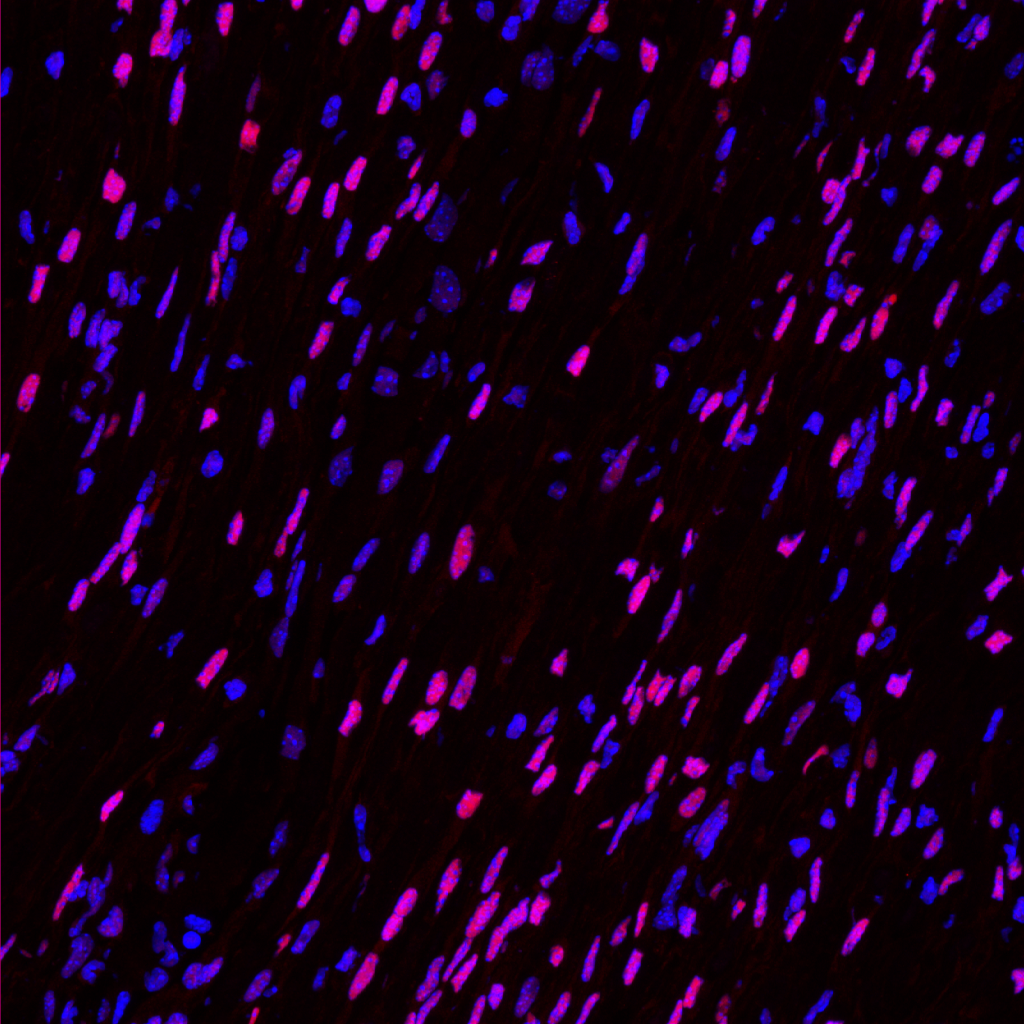

Supplement: Figure 4—source data 1. — This zip archive contains the IHC for one WT and one iDKO used for quantitative analysis shown in Figure 4E. Leica SP8 confocal lif images were processed using Imaris software and saved as tiffs. [file elife-50138-fig4-data1.zip › Figure 4 source data 1/WT #579 cJun/RHS a Sox10 + DAPI.tif]

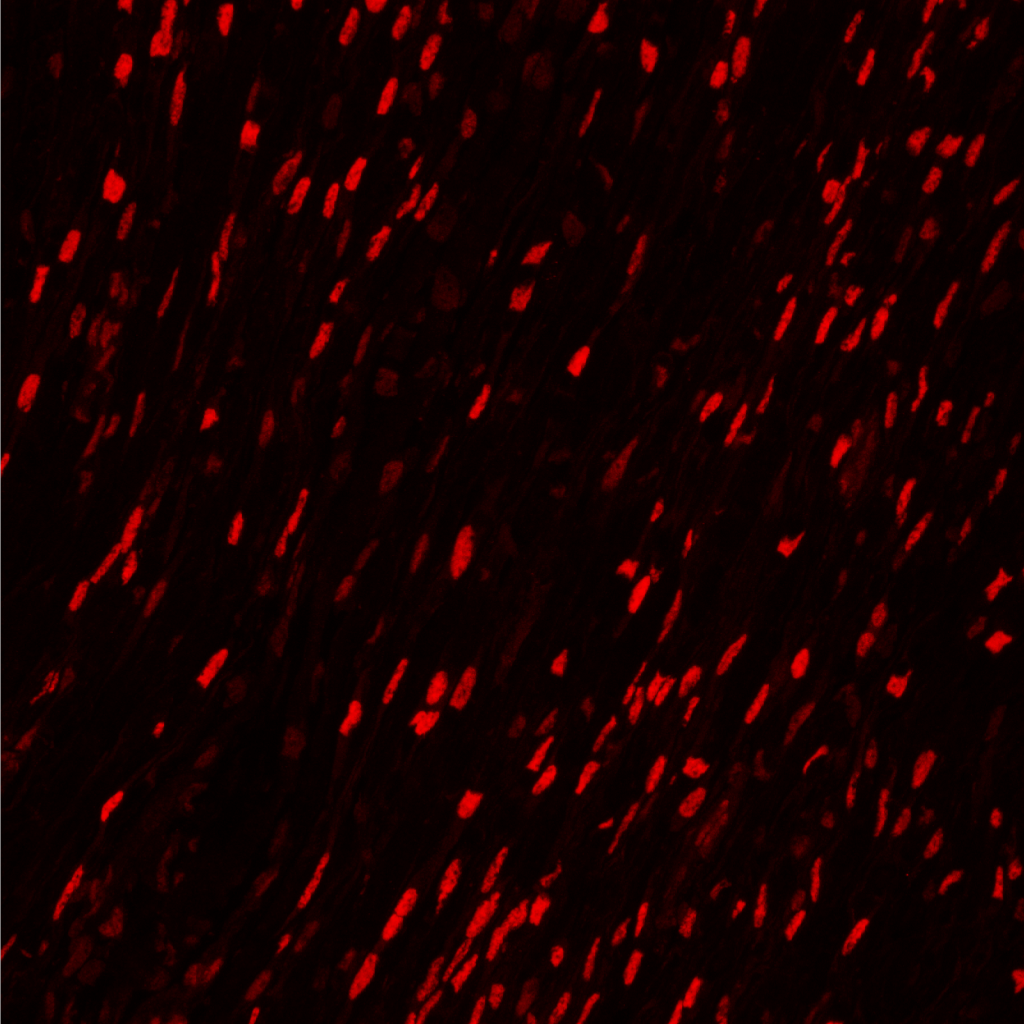

Supplement: Figure 4—source data 1. — This zip archive contains the IHC for one WT and one iDKO used for quantitative analysis shown in Figure 4E. Leica SP8 confocal lif images were processed using Imaris software and saved as tiffs. [file elife-50138-fig4-data1.zip › Figure 4 source data 1/WT #579 cJun/RHS a Sox10.tif]

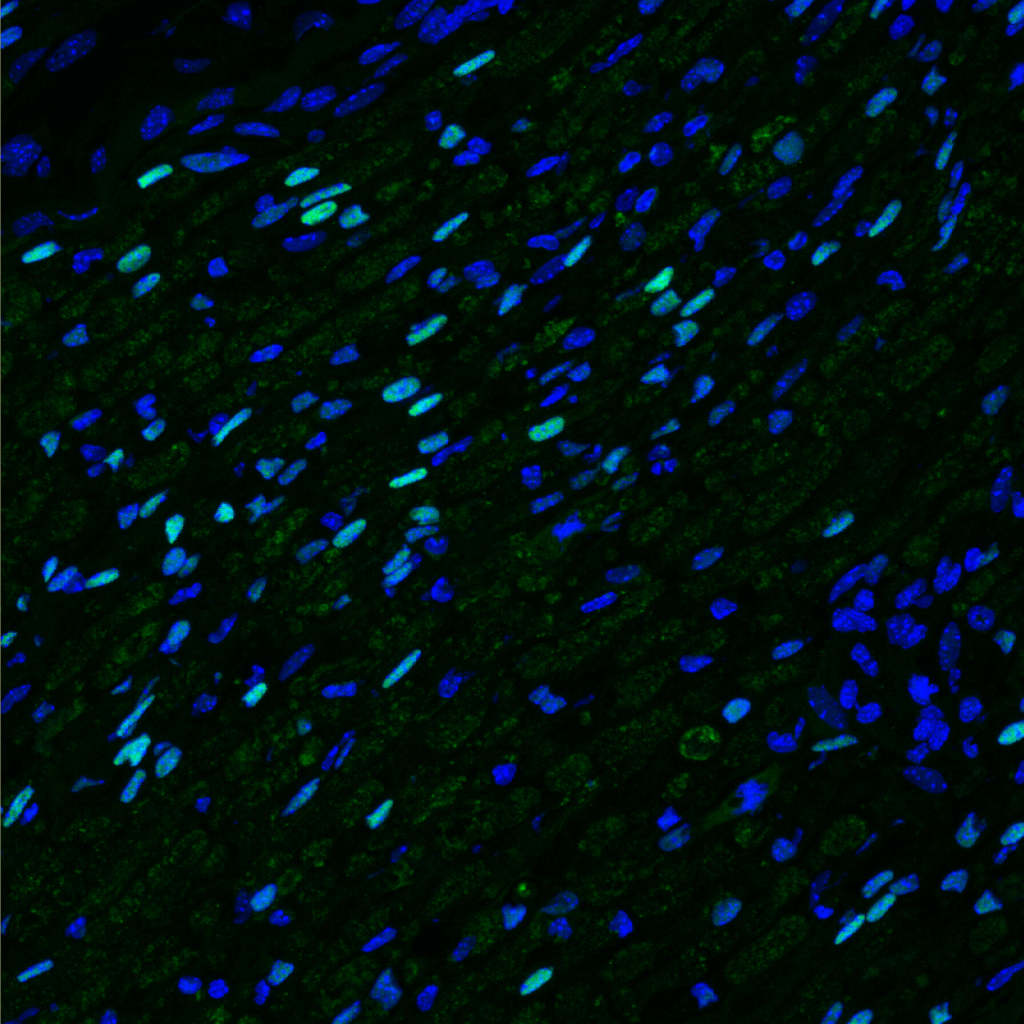

Supplement: Figure 4—source data 1. — This zip archive contains the IHC for one WT and one iDKO used for quantitative analysis shown in Figure 4E. Leica SP8 confocal lif images were processed using Imaris software and saved as tiffs. [file elife-50138-fig4-data1.zip › Figure 4 source data 1/WT #579 cJun/RHS b cJun + DAPI.tif]

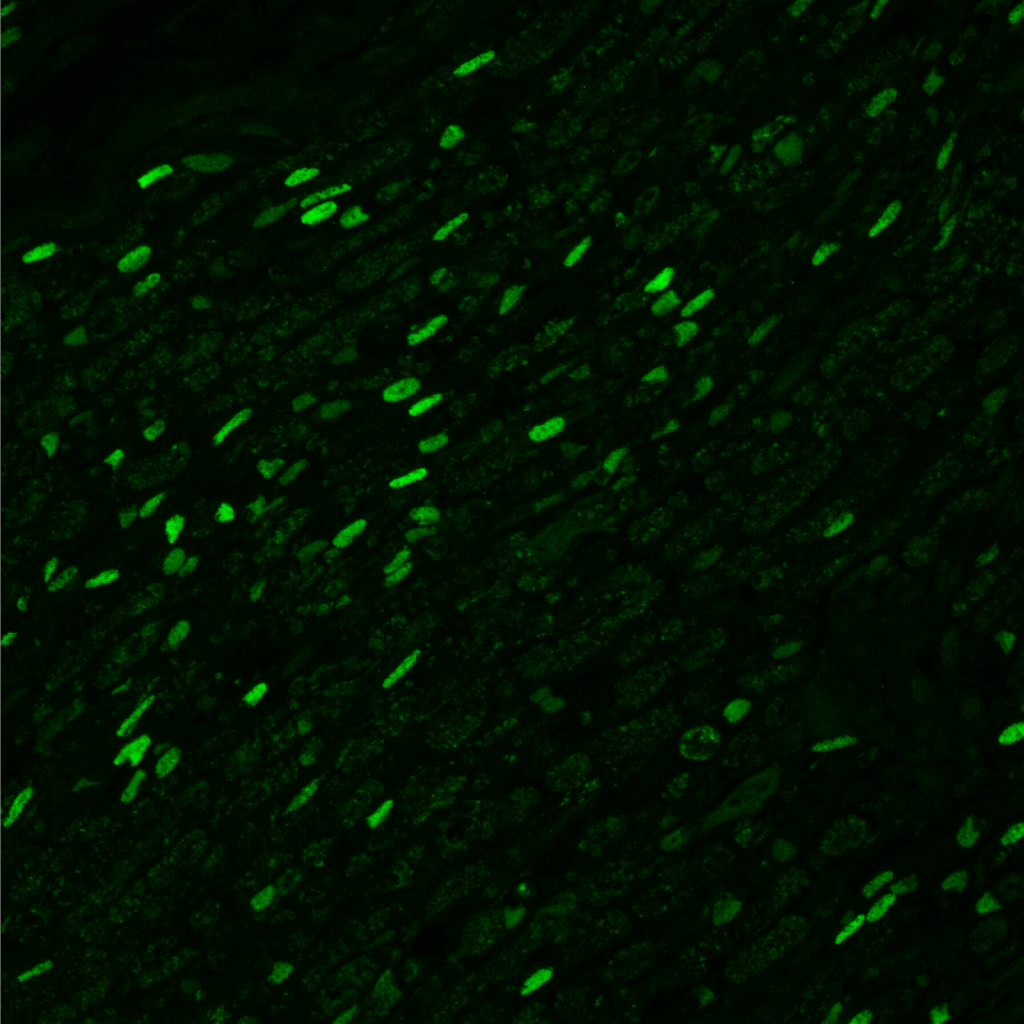

Supplement: Figure 4—source data 1. — This zip archive contains the IHC for one WT and one iDKO used for quantitative analysis shown in Figure 4E. Leica SP8 confocal lif images were processed using Imaris software and saved as tiffs. [file elife-50138-fig4-data1.zip › Figure 4 source data 1/WT #579 cJun/RHS b cJun.tif]

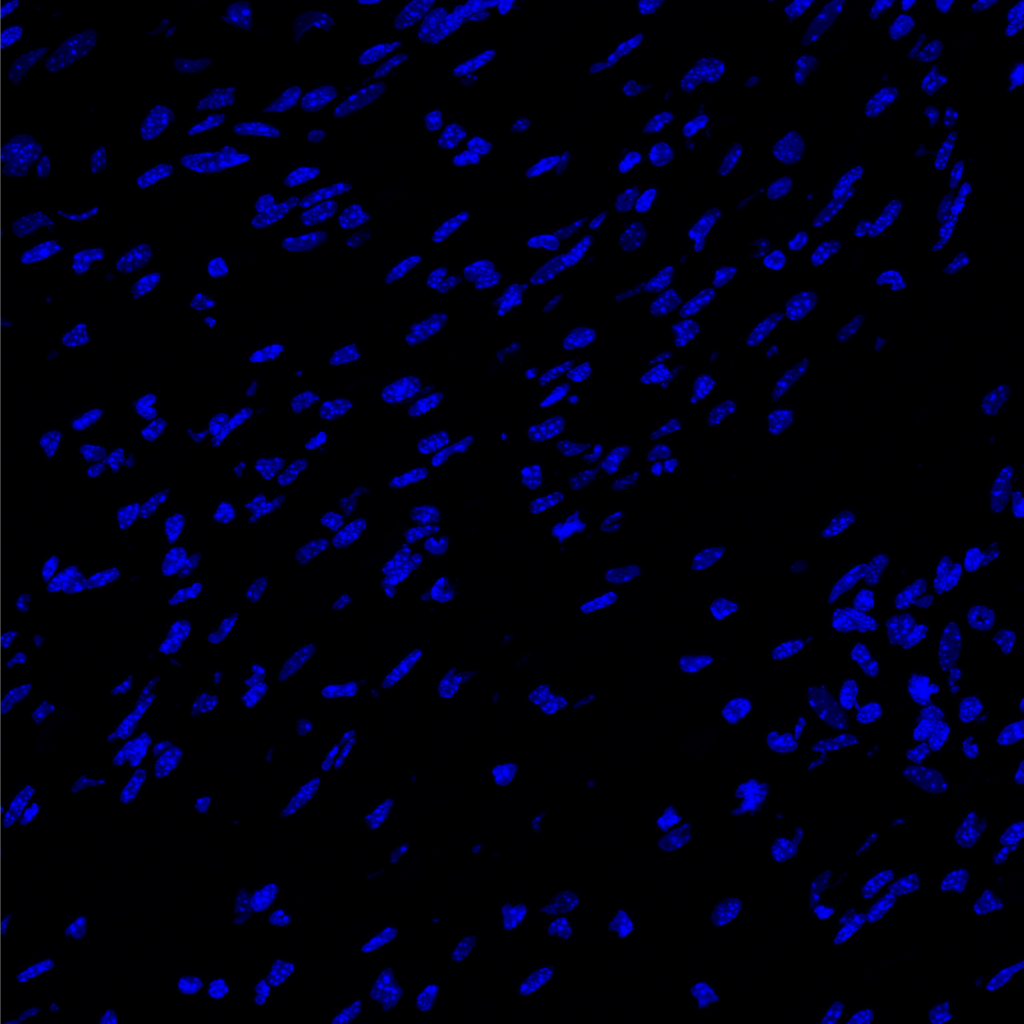

Supplement: Figure 4—source data 1. — This zip archive contains the IHC for one WT and one iDKO used for quantitative analysis shown in Figure 4E. Leica SP8 confocal lif images were processed using Imaris software and saved as tiffs. [file elife-50138-fig4-data1.zip › Figure 4 source data 1/WT #579 cJun/RHS b DAPI.tif]

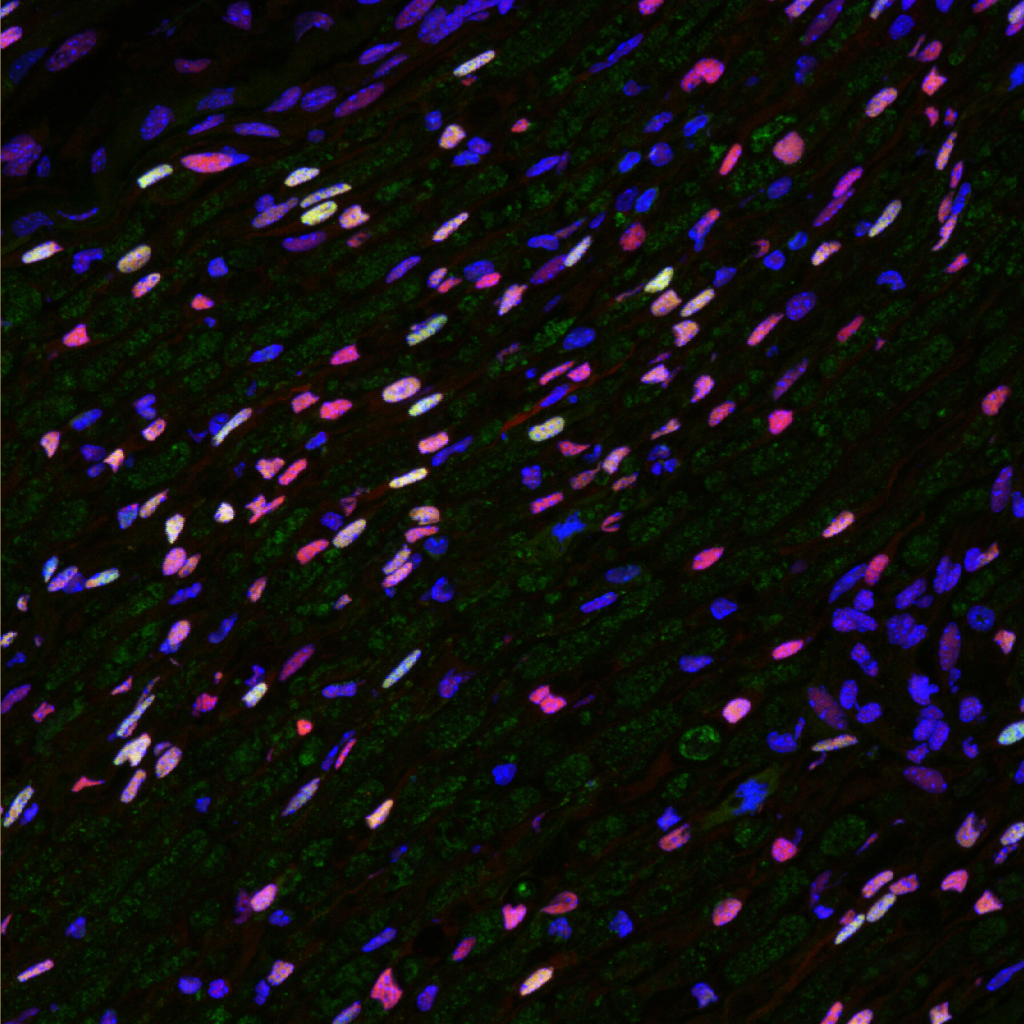

Supplement: Figure 4—source data 1. — This zip archive contains the IHC for one WT and one iDKO used for quantitative analysis shown in Figure 4E. Leica SP8 confocal lif images were processed using Imaris software and saved as tiffs. [file elife-50138-fig4-data1.zip › Figure 4 source data 1/WT #579 cJun/RHS b merge.tif]

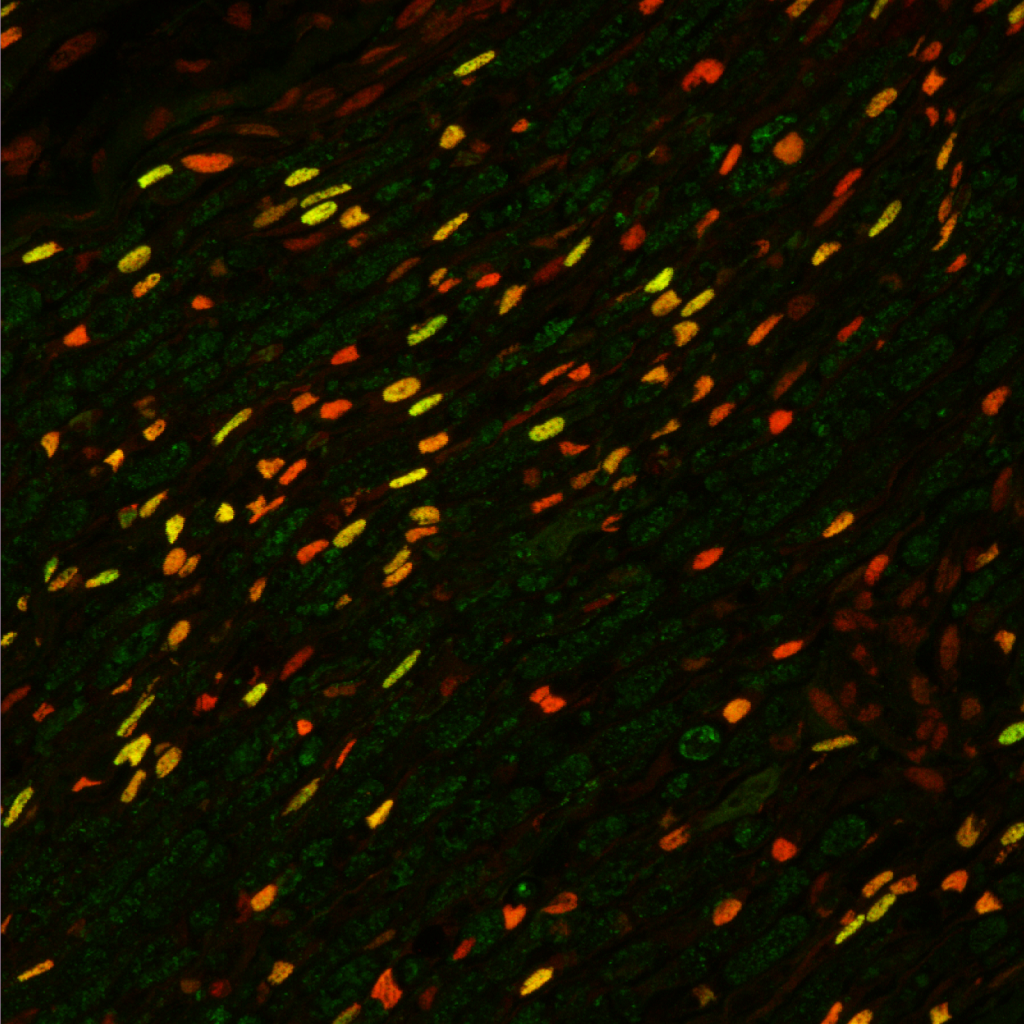

Supplement: Figure 4—source data 1. — This zip archive contains the IHC for one WT and one iDKO used for quantitative analysis shown in Figure 4E. Leica SP8 confocal lif images were processed using Imaris software and saved as tiffs. [file elife-50138-fig4-data1.zip › Figure 4 source data 1/WT #579 cJun/RHS b Sox10 + cJun.tif]

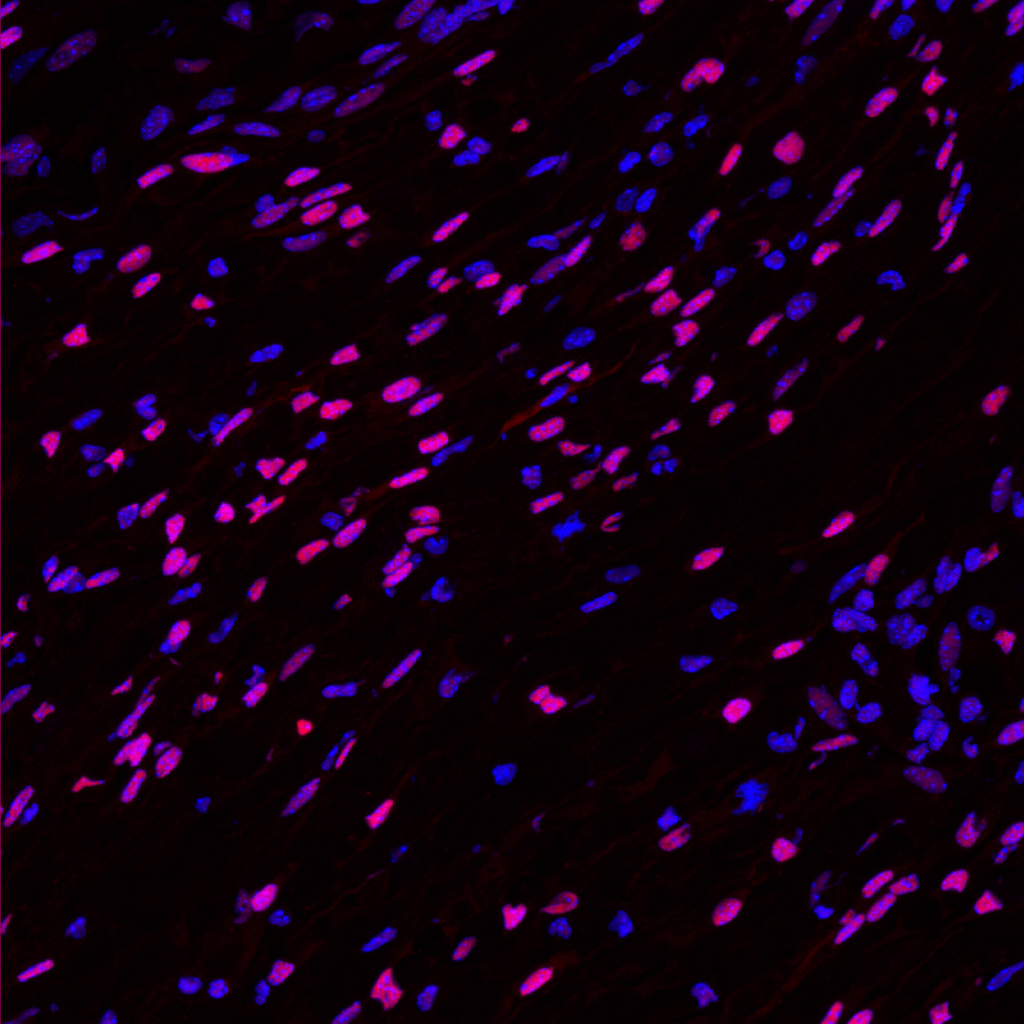

Supplement: Figure 4—source data 1. — This zip archive contains the IHC for one WT and one iDKO used for quantitative analysis shown in Figure 4E. Leica SP8 confocal lif images were processed using Imaris software and saved as tiffs. [file elife-50138-fig4-data1.zip › Figure 4 source data 1/WT #579 cJun/RHS b Sox10 + DAPI.tif]

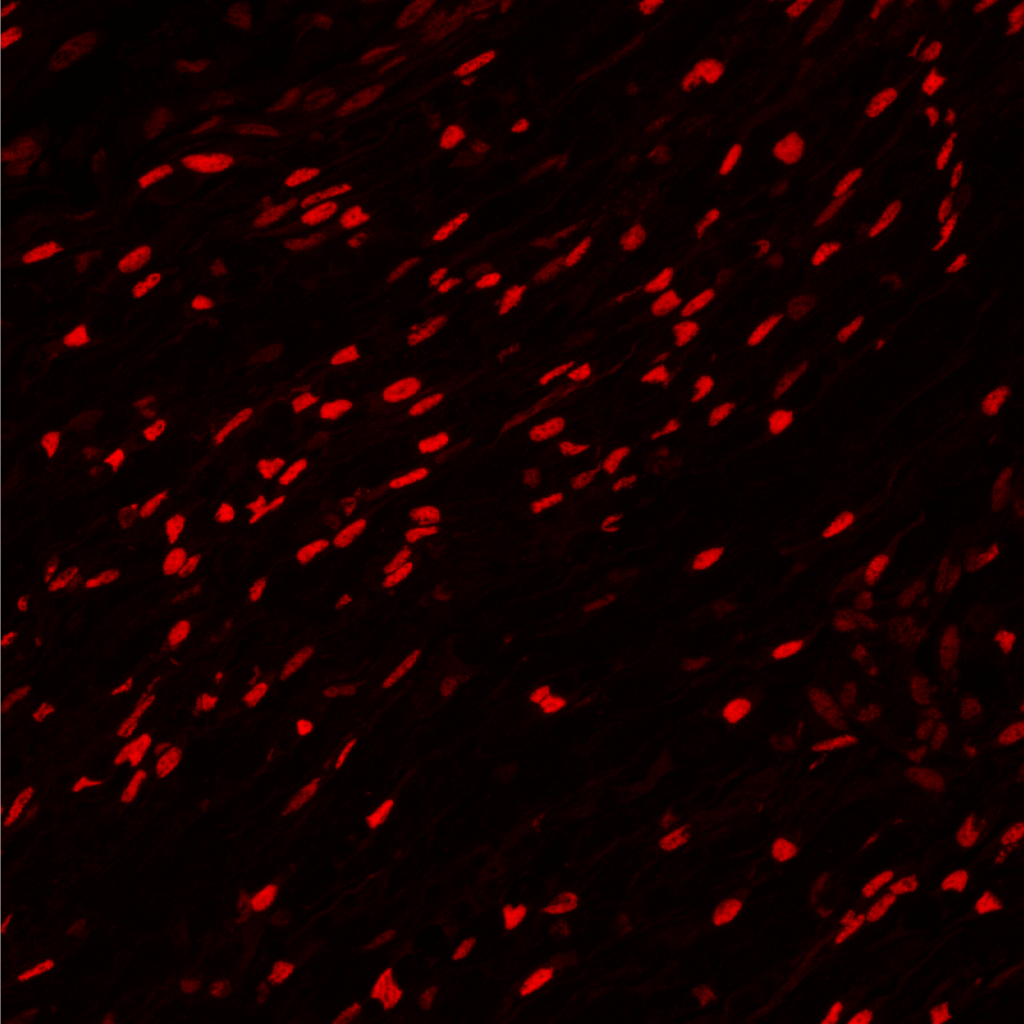

Supplement: Figure 4—source data 1. — This zip archive contains the IHC for one WT and one iDKO used for quantitative analysis shown in Figure 4E. Leica SP8 confocal lif images were processed using Imaris software and saved as tiffs. [file elife-50138-fig4-data1.zip › Figure 4 source data 1/WT #579 cJun/RHS b Sox10.tif]

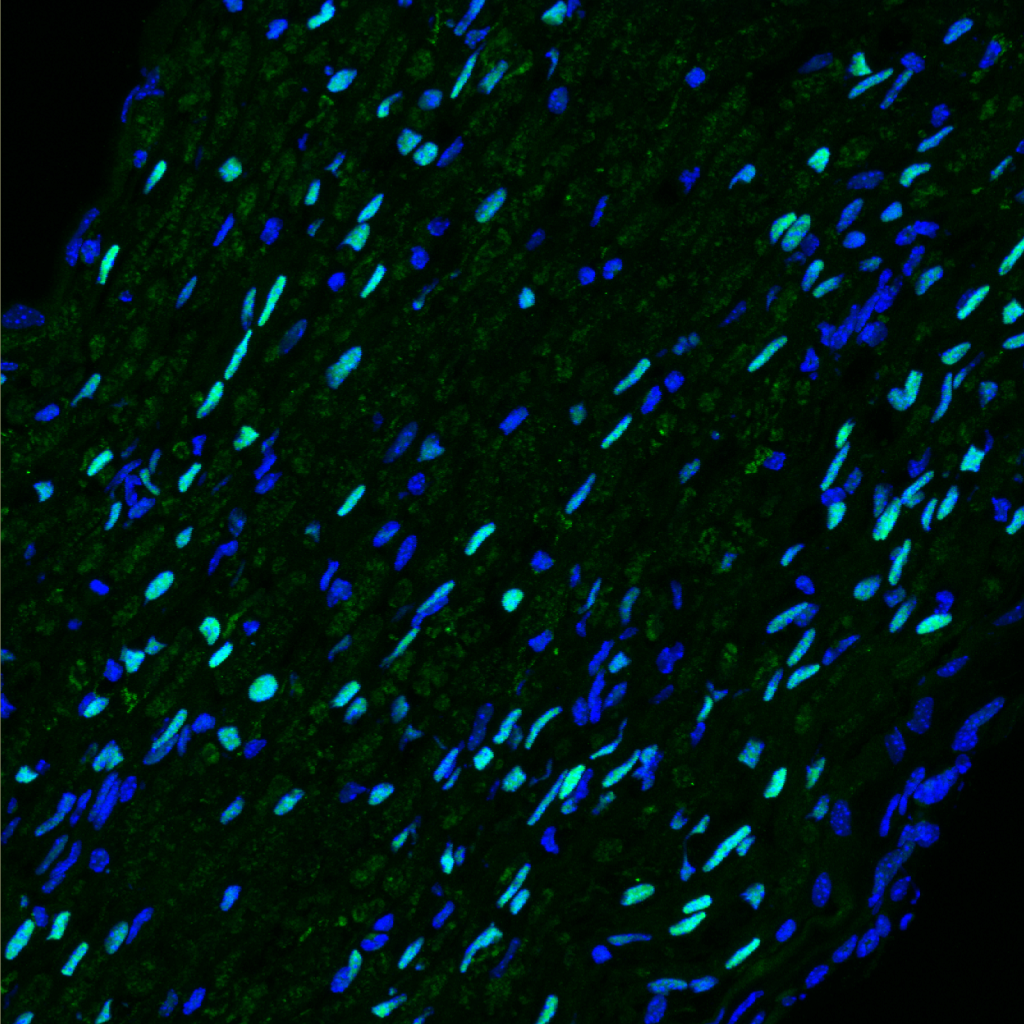

Supplement: Figure 4—source data 1. — This zip archive contains the IHC for one WT and one iDKO used for quantitative analysis shown in Figure 4E. Leica SP8 confocal lif images were processed using Imaris software and saved as tiffs. [file elife-50138-fig4-data1.zip › Figure 4 source data 1/WT #579 cJun/RHS c cJun + DAPI.tif]

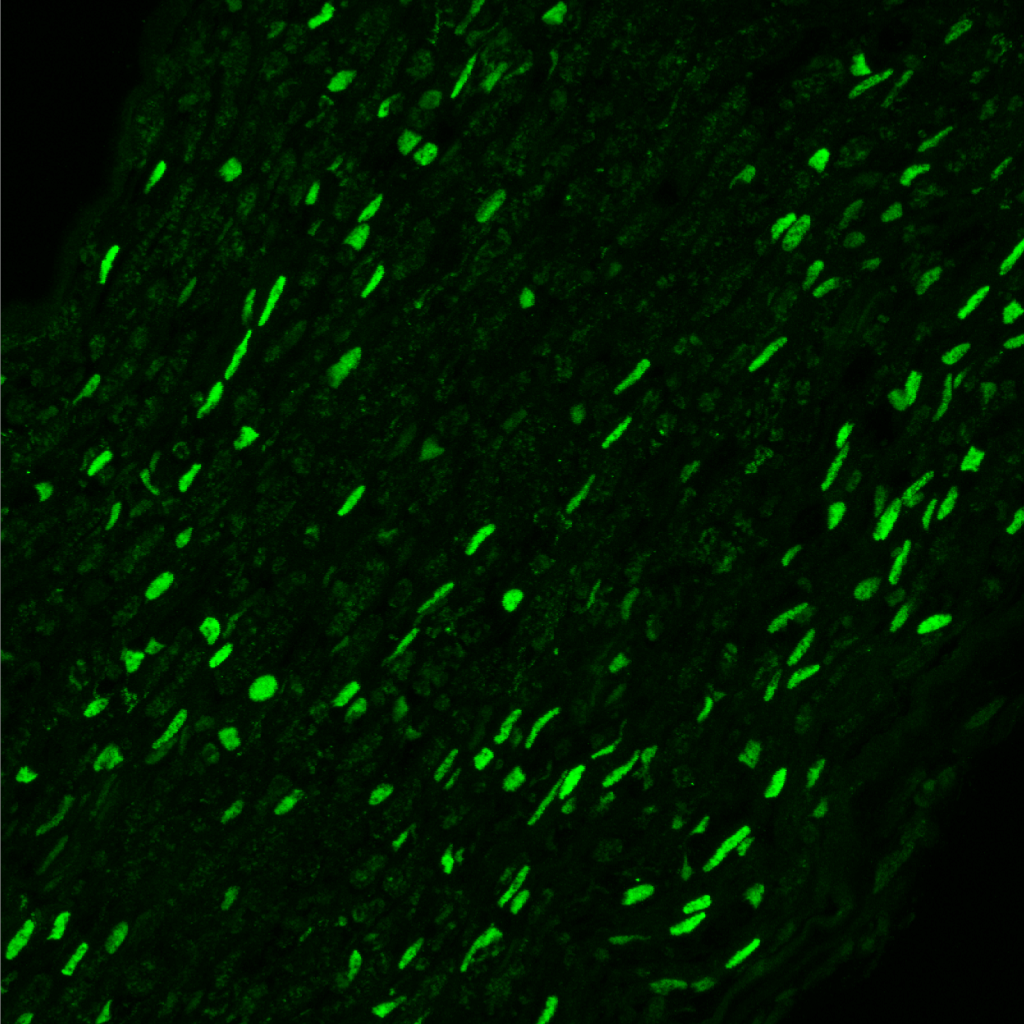

Supplement: Figure 4—source data 1. — This zip archive contains the IHC for one WT and one iDKO used for quantitative analysis shown in Figure 4E. Leica SP8 confocal lif images were processed using Imaris software and saved as tiffs. [file elife-50138-fig4-data1.zip › Figure 4 source data 1/WT #579 cJun/RHS c cJun.tif]

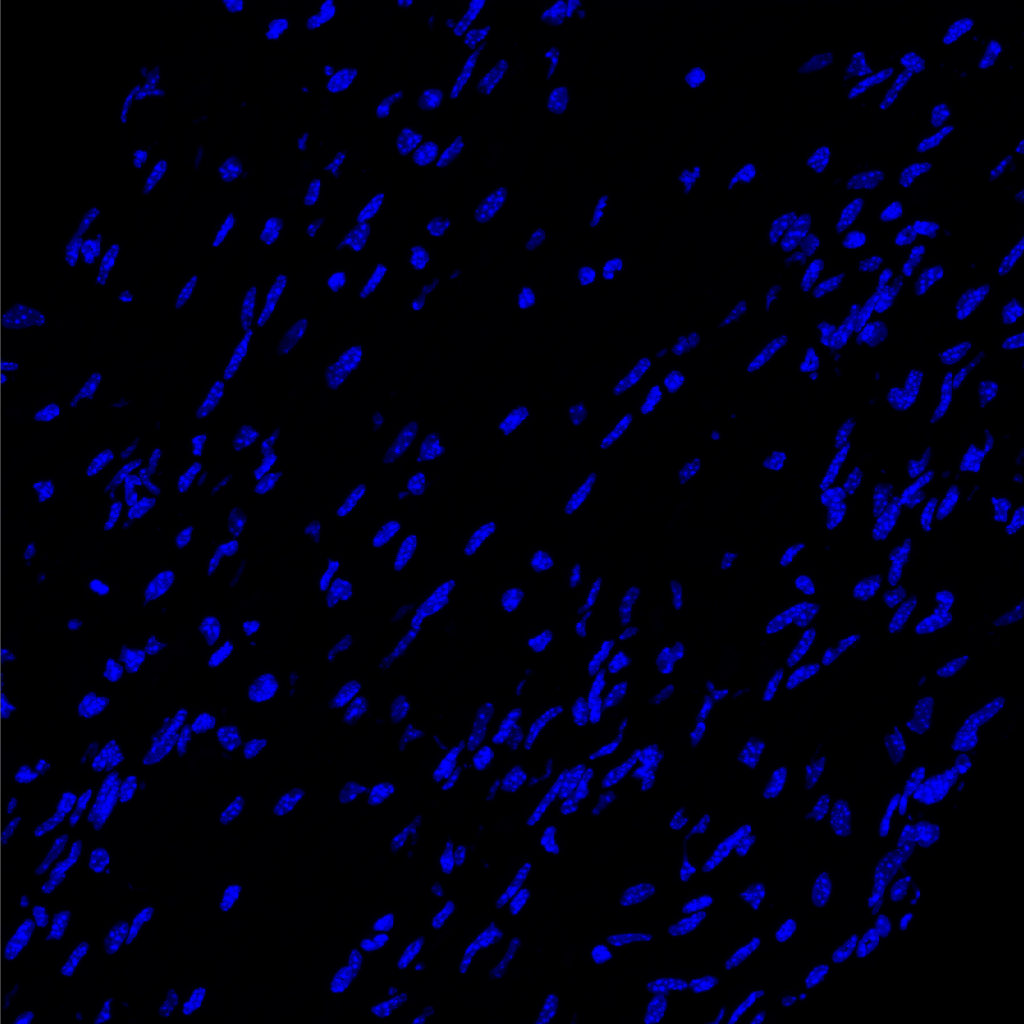

Supplement: Figure 4—source data 1. — This zip archive contains the IHC for one WT and one iDKO used for quantitative analysis shown in Figure 4E. Leica SP8 confocal lif images were processed using Imaris software and saved as tiffs. [file elife-50138-fig4-data1.zip › Figure 4 source data 1/WT #579 cJun/RHS c DAPI.tif]

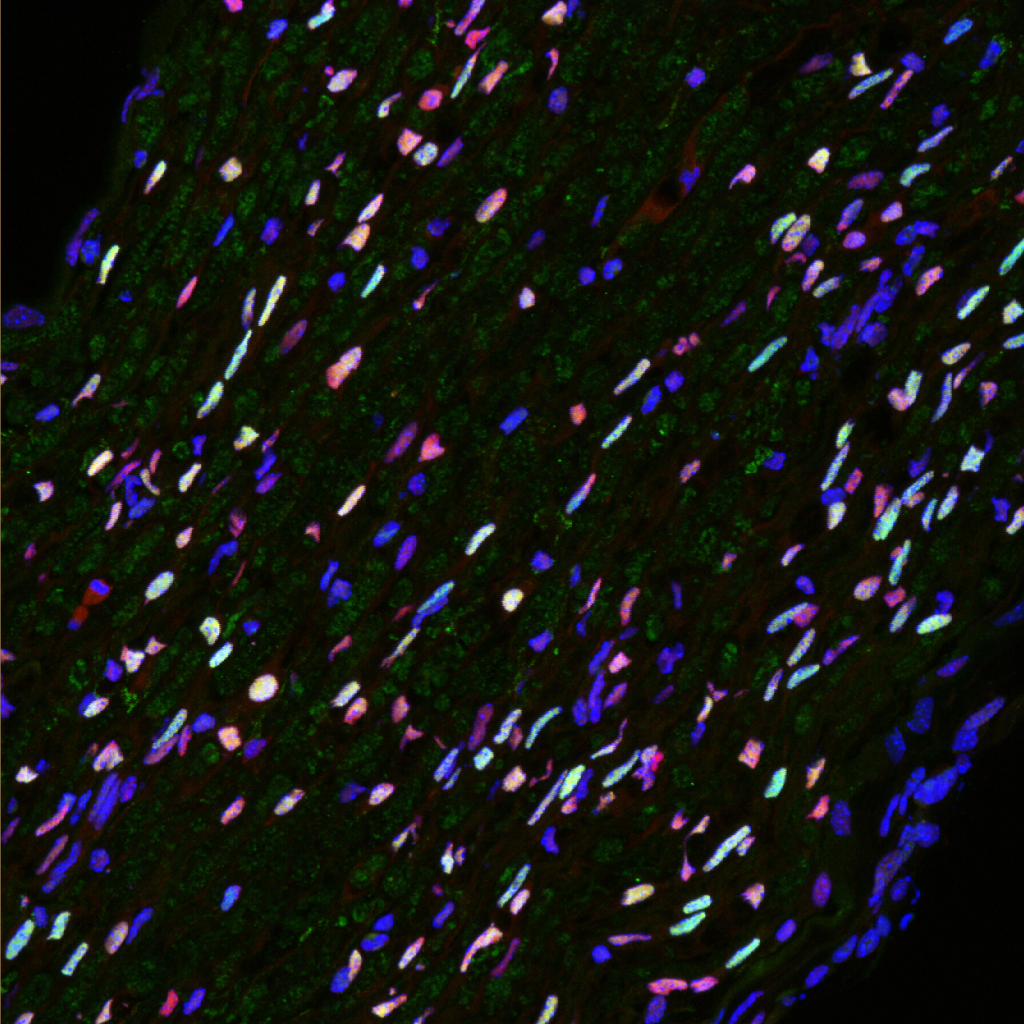

Supplement: Figure 4—source data 1. — This zip archive contains the IHC for one WT and one iDKO used for quantitative analysis shown in Figure 4E. Leica SP8 confocal lif images were processed using Imaris software and saved as tiffs. [file elife-50138-fig4-data1.zip › Figure 4 source data 1/WT #579 cJun/RHS c merge.tif]

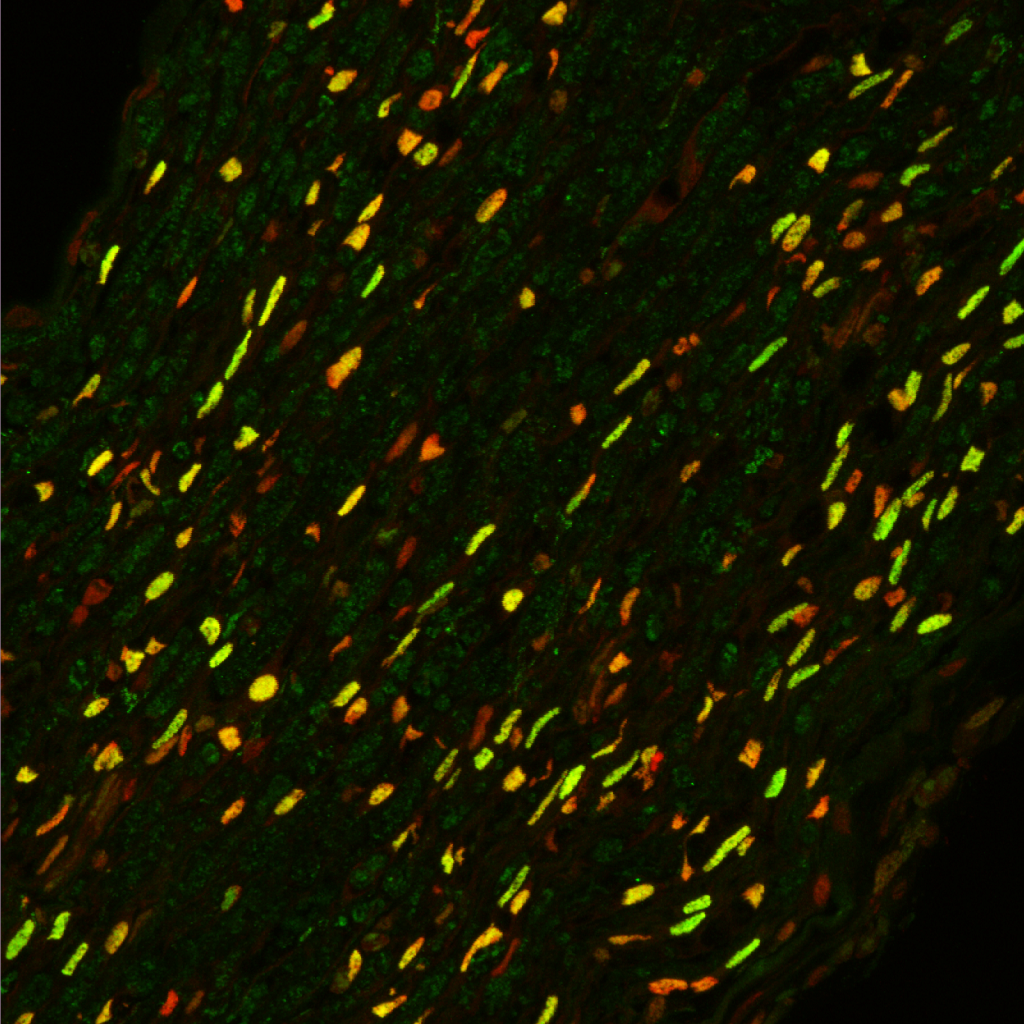

Supplement: Figure 4—source data 1. — This zip archive contains the IHC for one WT and one iDKO used for quantitative analysis shown in Figure 4E. Leica SP8 confocal lif images were processed using Imaris software and saved as tiffs. [file elife-50138-fig4-data1.zip › Figure 4 source data 1/WT #579 cJun/RHS c Sox10 + cJun.tif]

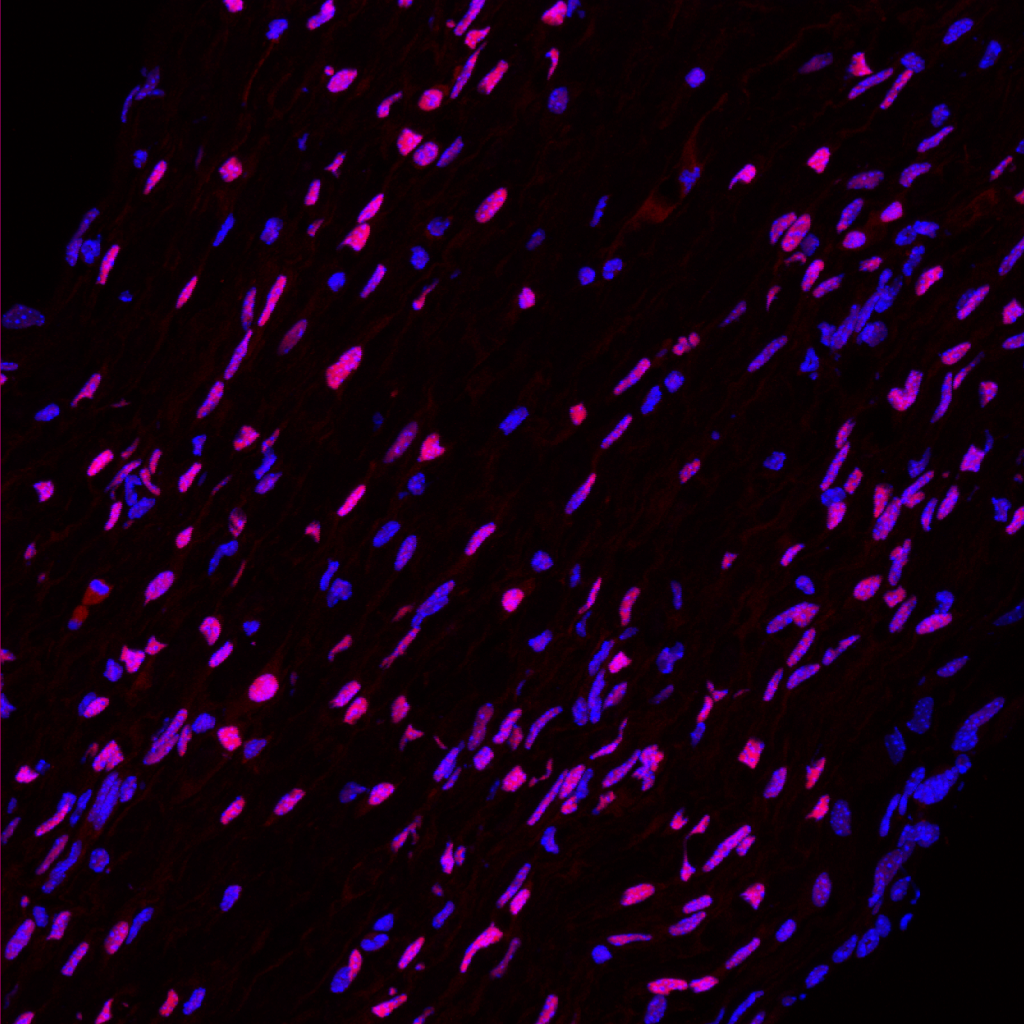

Supplement: Figure 4—source data 1. — This zip archive contains the IHC for one WT and one iDKO used for quantitative analysis shown in Figure 4E. Leica SP8 confocal lif images were processed using Imaris software and saved as tiffs. [file elife-50138-fig4-data1.zip › Figure 4 source data 1/WT #579 cJun/RHS c Sox10 + DAPI.tif]

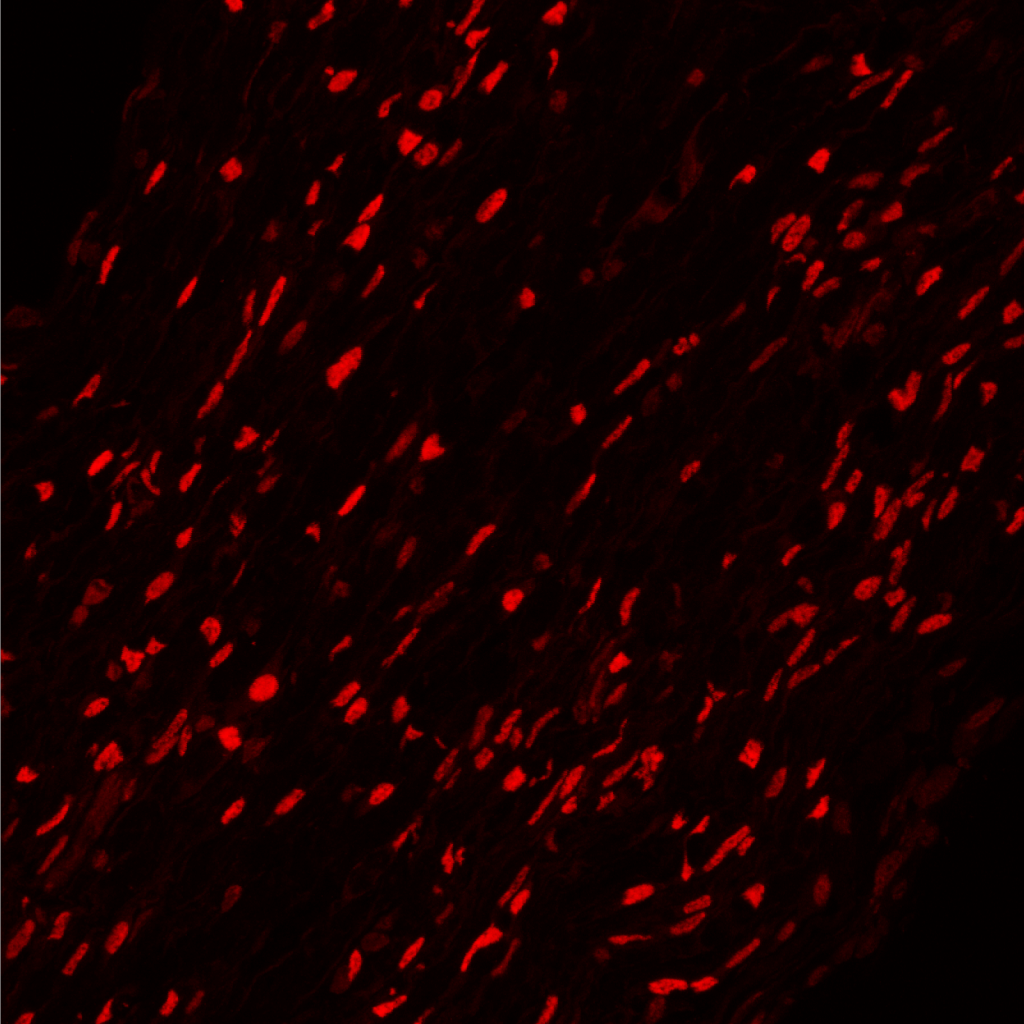

Supplement: Figure 4—source data 1. — This zip archive contains the IHC for one WT and one iDKO used for quantitative analysis shown in Figure 4E. Leica SP8 confocal lif images were processed using Imaris software and saved as tiffs. [file elife-50138-fig4-data1.zip › Figure 4 source data 1/WT #579 cJun/RHS c Sox10.tif]

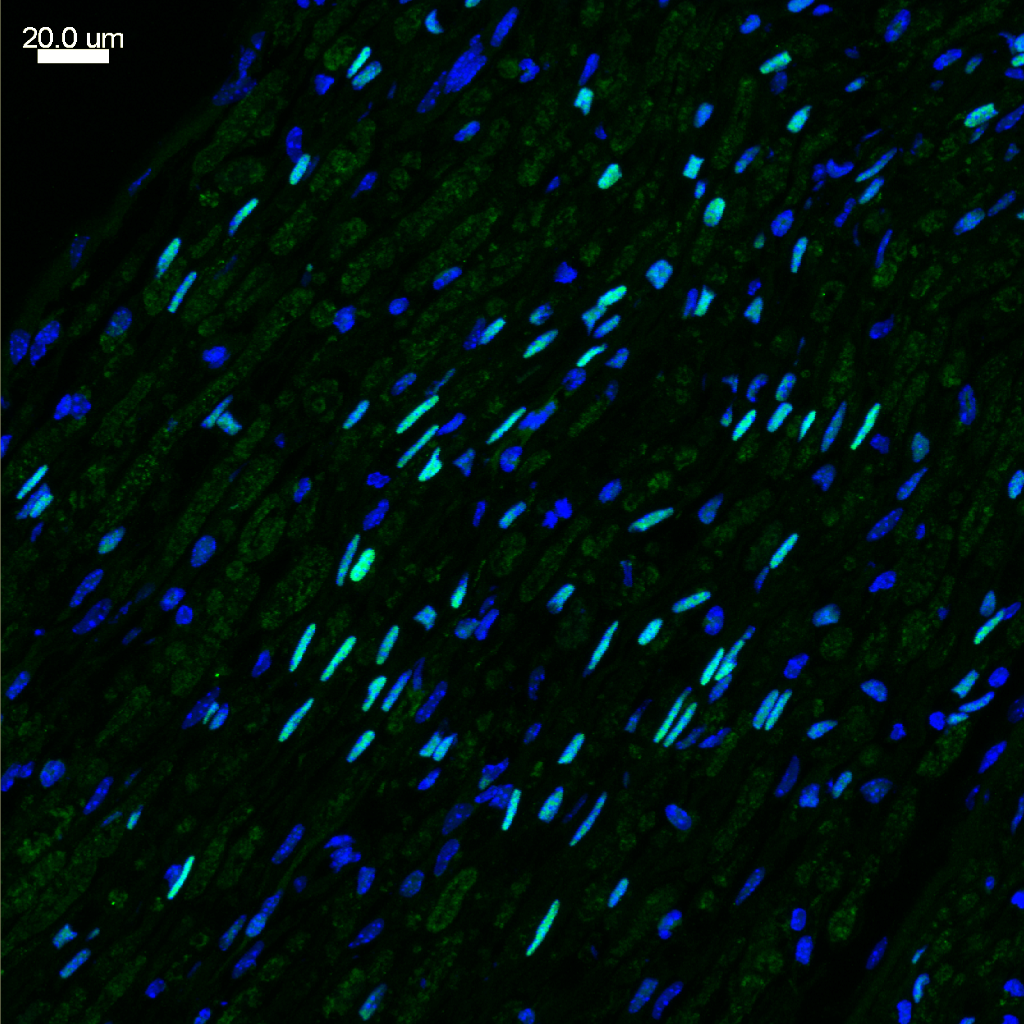

Supplement: Figure 4—source data 1. — This zip archive contains the IHC for one WT and one iDKO used for quantitative analysis shown in Figure 4E. Leica SP8 confocal lif images were processed using Imaris software and saved as tiffs. [file elife-50138-fig4-data1.zip › Figure 4 source data 1/WT #579 cJun/RHS d cJun + DAPI.tif]

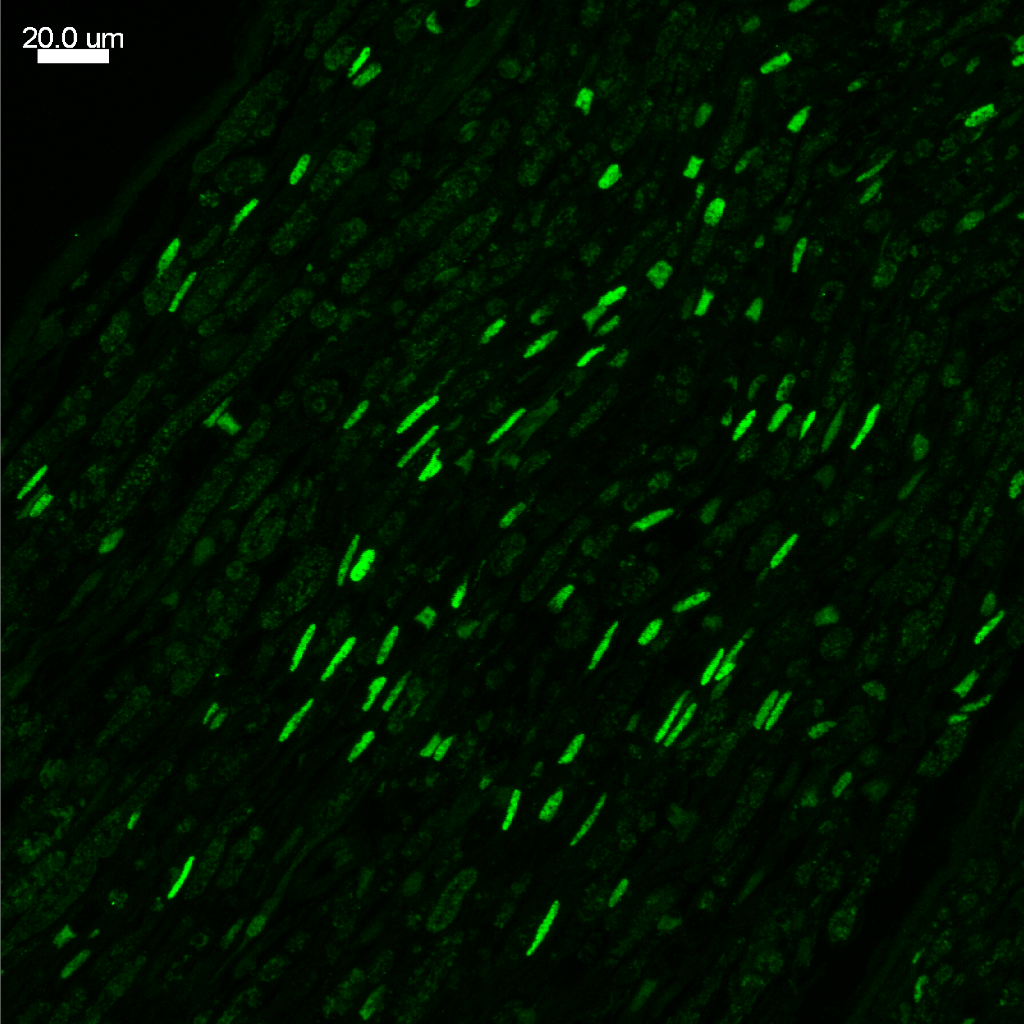

Supplement: Figure 4—source data 1. — This zip archive contains the IHC for one WT and one iDKO used for quantitative analysis shown in Figure 4E. Leica SP8 confocal lif images were processed using Imaris software and saved as tiffs. [file elife-50138-fig4-data1.zip › Figure 4 source data 1/WT #579 cJun/RHS d cJun.tif]

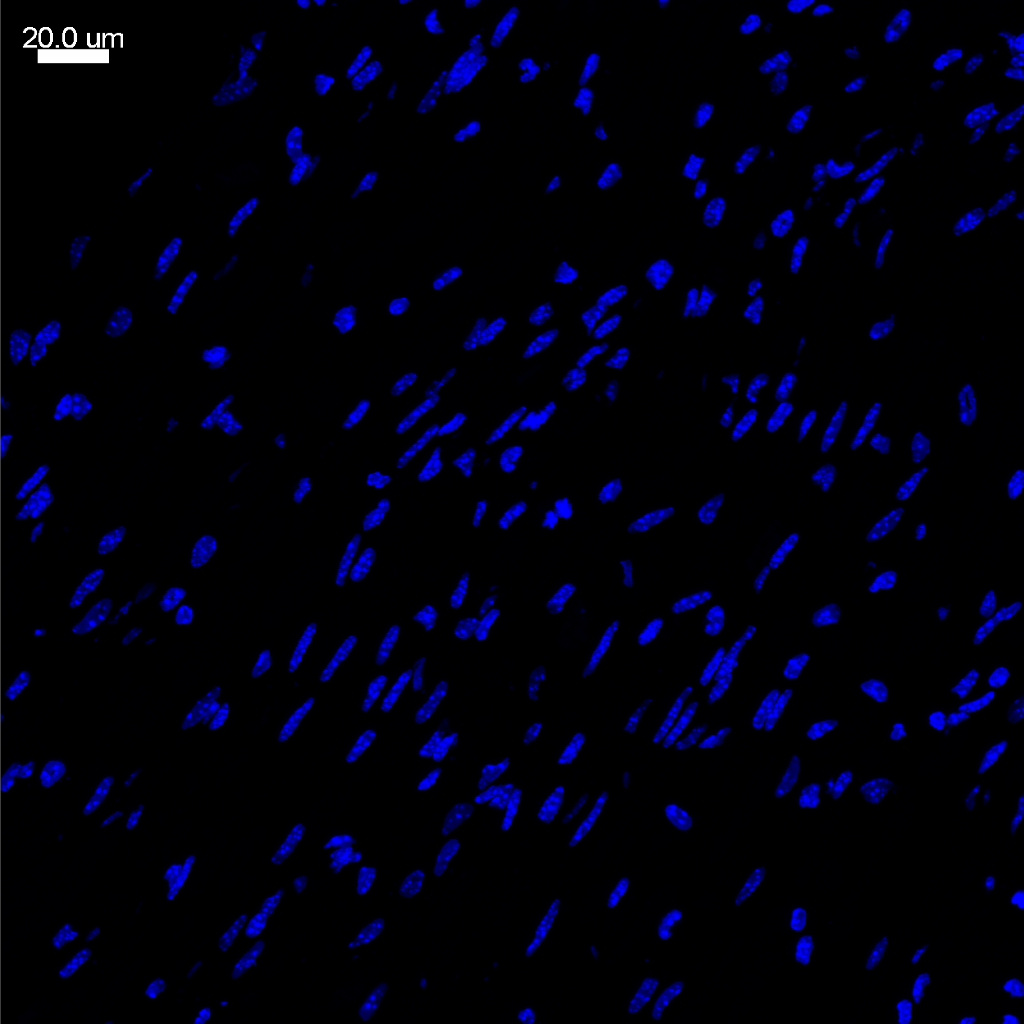

Supplement: Figure 4—source data 1. — This zip archive contains the IHC for one WT and one iDKO used for quantitative analysis shown in Figure 4E. Leica SP8 confocal lif images were processed using Imaris software and saved as tiffs. [file elife-50138-fig4-data1.zip › Figure 4 source data 1/WT #579 cJun/RHS d DAPI.tif]

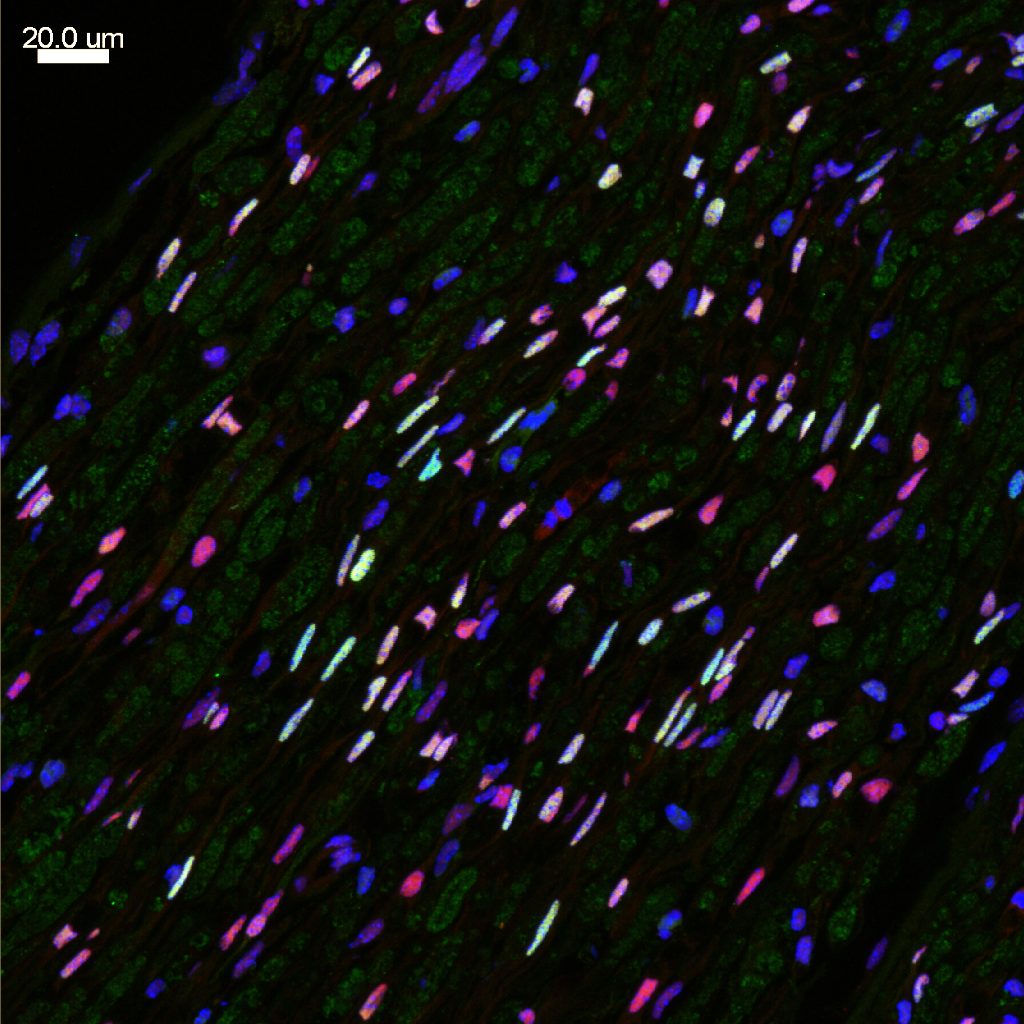

Supplement: Figure 4—source data 1. — This zip archive contains the IHC for one WT and one iDKO used for quantitative analysis shown in Figure 4E. Leica SP8 confocal lif images were processed using Imaris software and saved as tiffs. [file elife-50138-fig4-data1.zip › Figure 4 source data 1/WT #579 cJun/RHS d merge.tif]

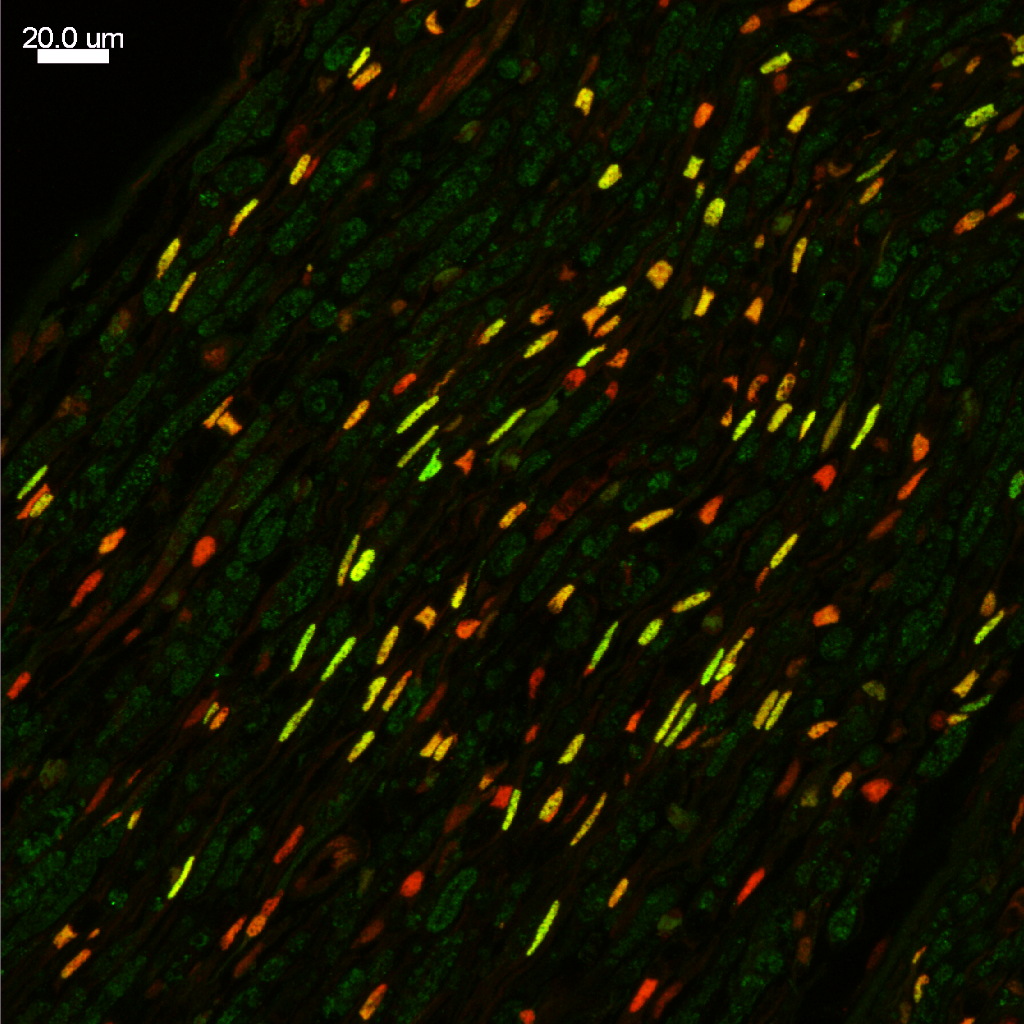

Supplement: Figure 4—source data 1. — This zip archive contains the IHC for one WT and one iDKO used for quantitative analysis shown in Figure 4E. Leica SP8 confocal lif images were processed using Imaris software and saved as tiffs. [file elife-50138-fig4-data1.zip › Figure 4 source data 1/WT #579 cJun/RHS d Sox10 + cJun.tif]

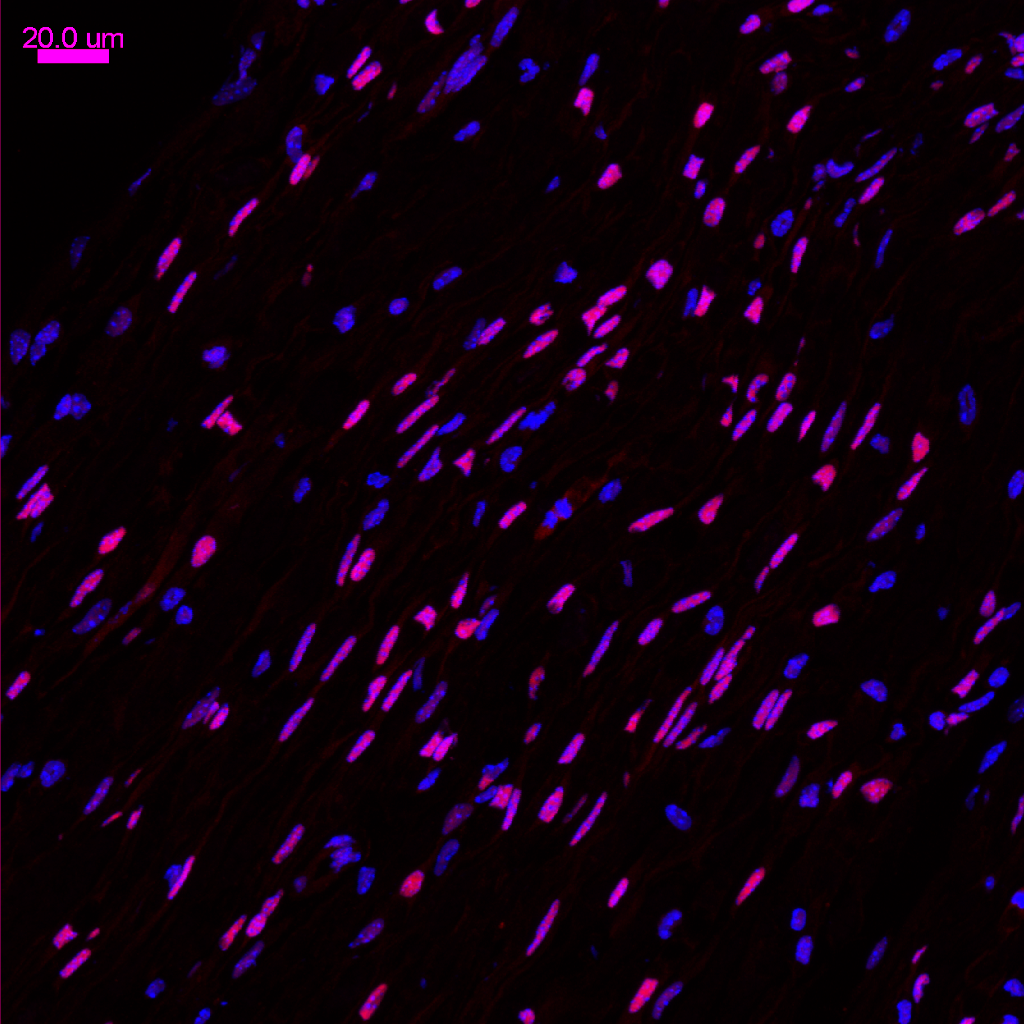

Supplement: Figure 4—source data 1. — This zip archive contains the IHC for one WT and one iDKO used for quantitative analysis shown in Figure 4E. Leica SP8 confocal lif images were processed using Imaris software and saved as tiffs. [file elife-50138-fig4-data1.zip › Figure 4 source data 1/WT #579 cJun/RHS d Sox10 + DAPI.tif]

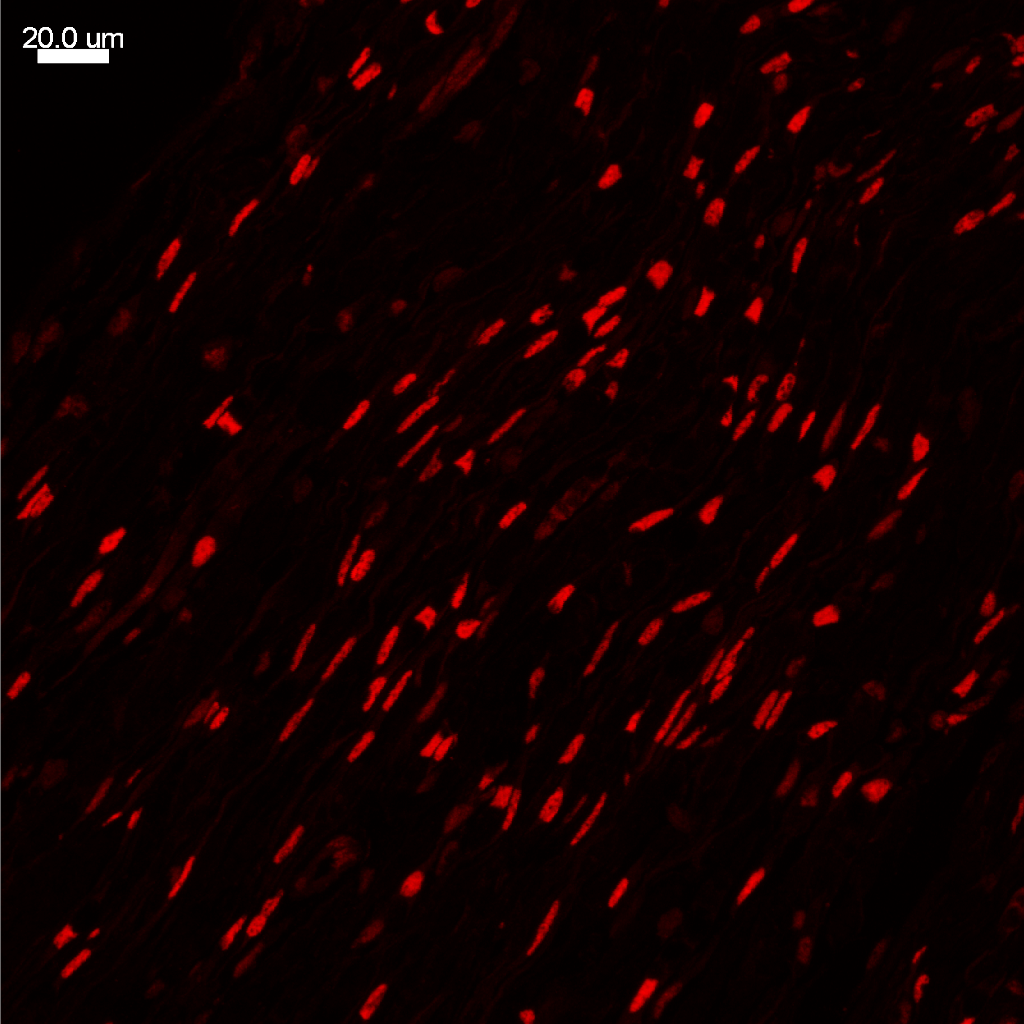

Supplement: Figure 4—source data 1. — This zip archive contains the IHC for one WT and one iDKO used for quantitative analysis shown in Figure 4E. Leica SP8 confocal lif images were processed using Imaris software and saved as tiffs. [file elife-50138-fig4-data1.zip › Figure 4 source data 1/WT #579 cJun/RHS d Sox10.tif]

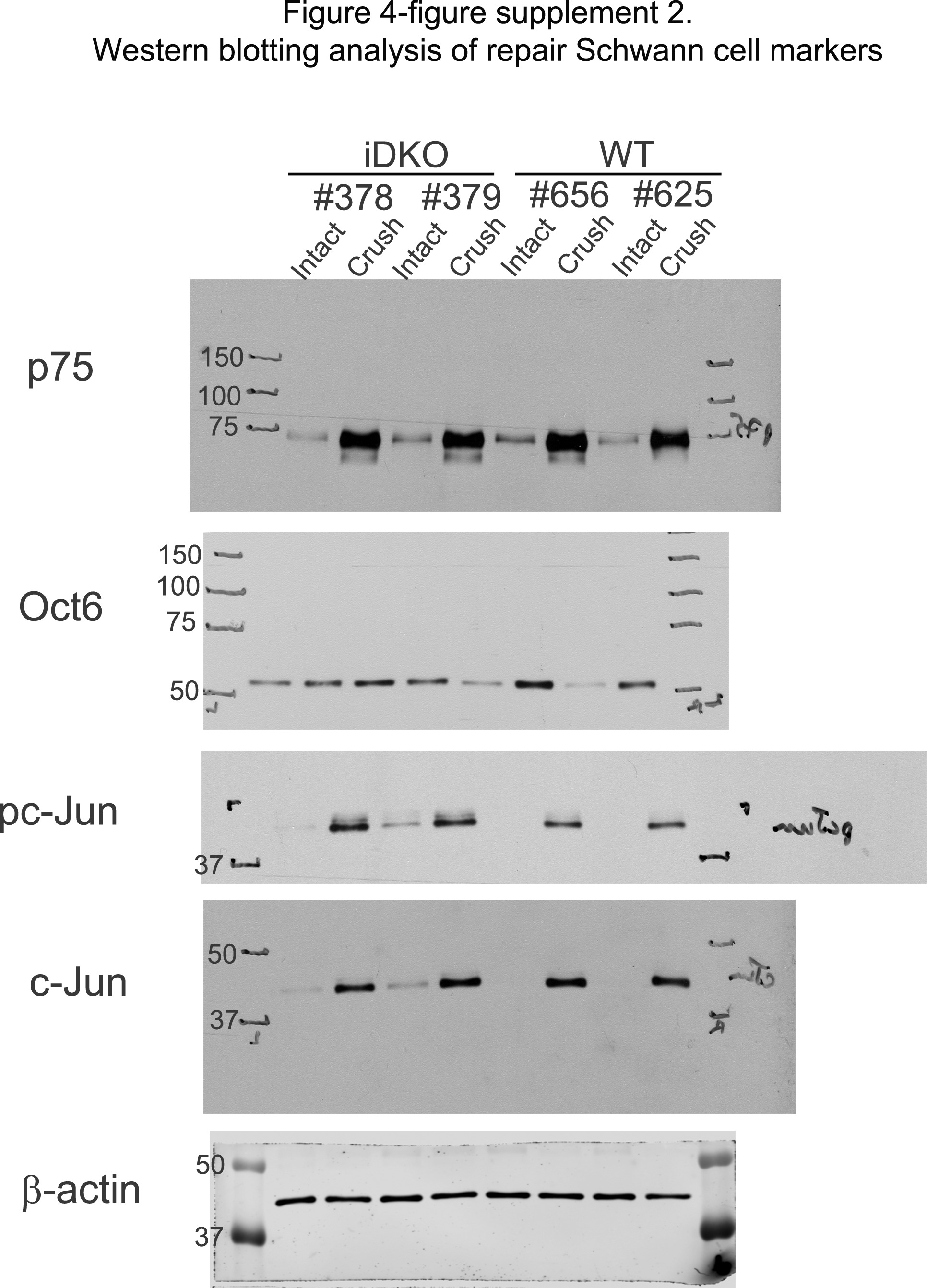

Supplement: Figure 4—source data 3. — Uncropped Western blots of images used to make Figure 4—figure supplement 1. Individually processed samples from 2 WT (#656, #625) and 2 iDKO mice (#378, #379) are shown and used for quantification. The following figure supplements are available for Figure 4. [file elife-50138-fig4-data3.jpg]

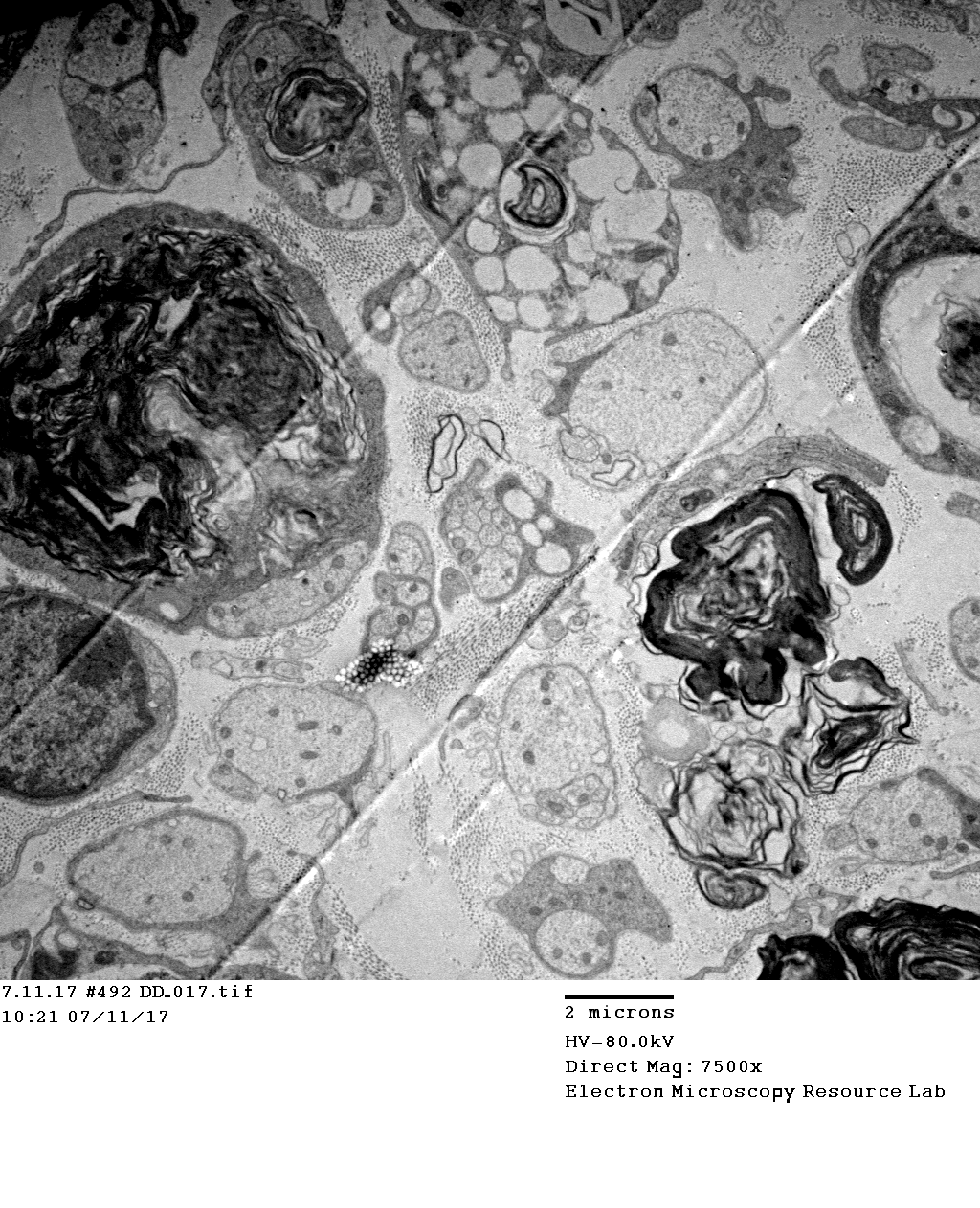

Supplement: Figure 5—source data 1. — This zip archive contains the TEM images for one WT and one iDKO used for quantitative analysis shown in Figure 5G–I. Images were taken using a JEOL 1010 electron microscope fitted with a Hamamatsu digital camera and AMT Advantage image capture software. Contrast of the images was adjusted using Photoshop software. The images in this archive were also used for the analysis in Figure 7. [file elife-50138-fig5-data1.zip › Figure 5 source data 1/iDKO #492 12d DD 7500X/7.11.17 #492 DD_017 Contrast .tif]

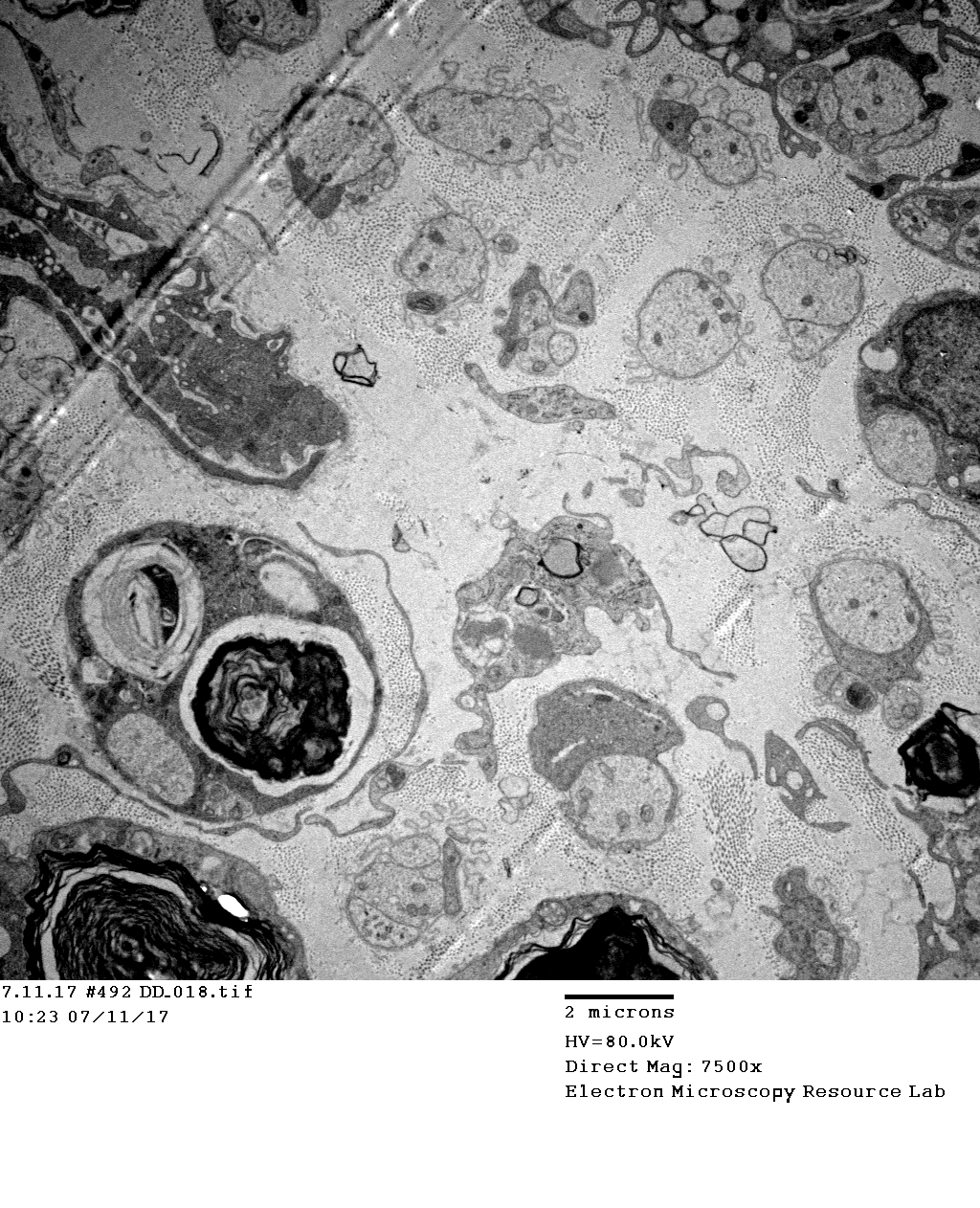

Supplement: Figure 5—source data 1. — This zip archive contains the TEM images for one WT and one iDKO used for quantitative analysis shown in Figure 5G–I. Images were taken using a JEOL 1010 electron microscope fitted with a Hamamatsu digital camera and AMT Advantage image capture software. Contrast of the images was adjusted using Photoshop software. The images in this archive were also used for the analysis in Figure 7. [file elife-50138-fig5-data1.zip › Figure 5 source data 1/iDKO #492 12d DD 7500X/7.11.17 #492 DD_018 Contrast .tif]

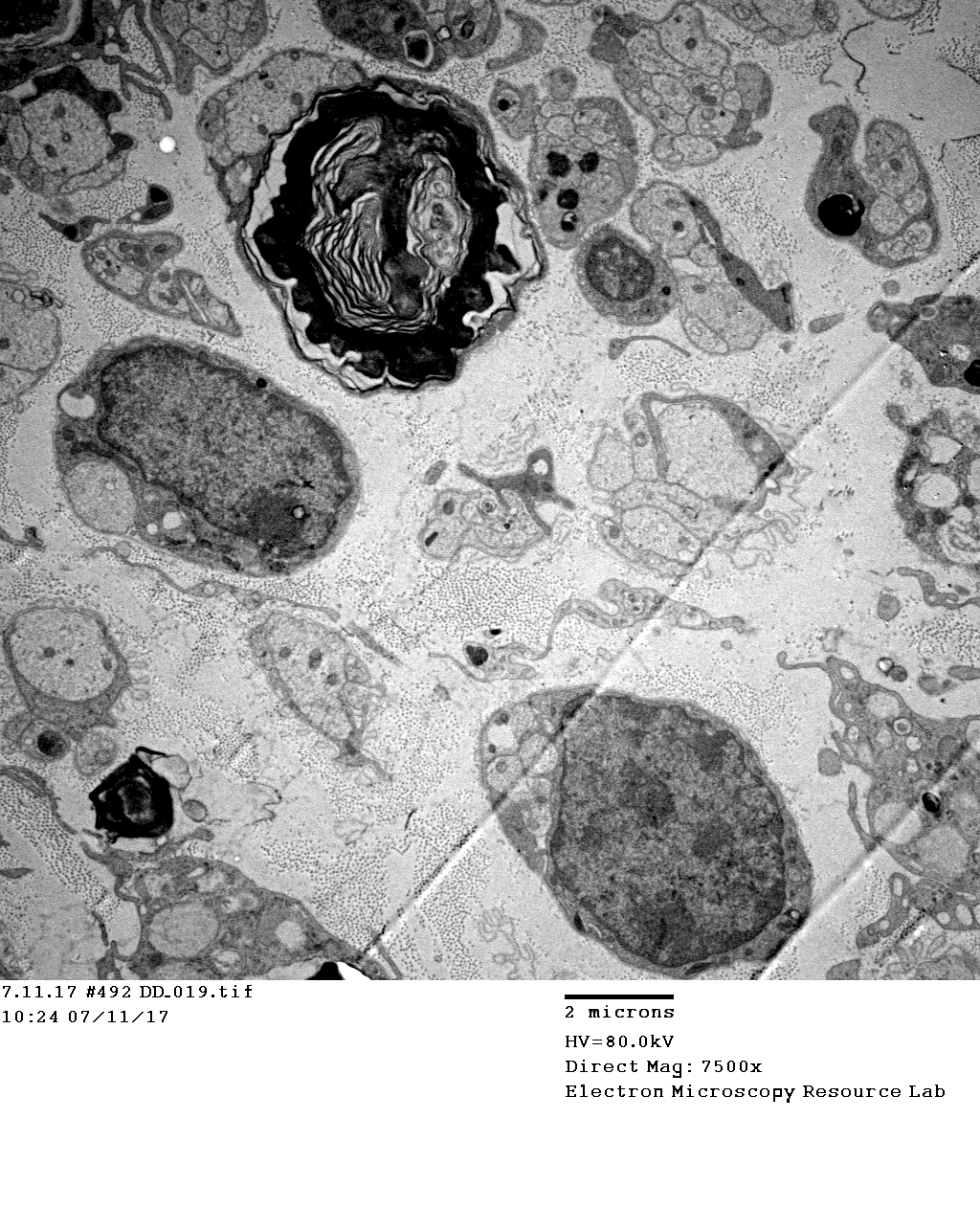

Supplement: Figure 5—source data 1. — This zip archive contains the TEM images for one WT and one iDKO used for quantitative analysis shown in Figure 5G–I. Images were taken using a JEOL 1010 electron microscope fitted with a Hamamatsu digital camera and AMT Advantage image capture software. Contrast of the images was adjusted using Photoshop software. The images in this archive were also used for the analysis in Figure 7. [file elife-50138-fig5-data1.zip › Figure 5 source data 1/iDKO #492 12d DD 7500X/7.11.17 #492 DD_019 Contrast .tif]

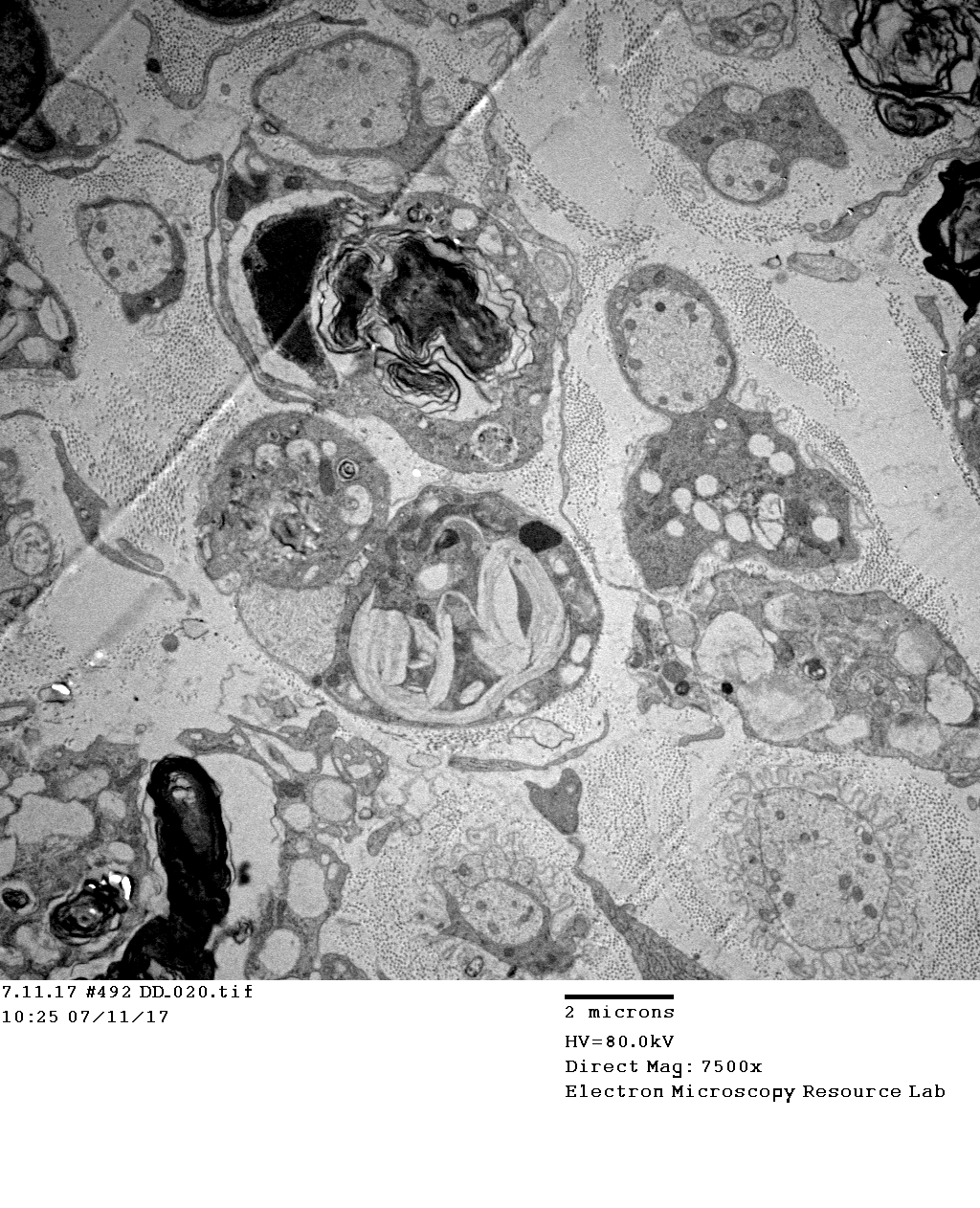

Supplement: Figure 5—source data 1. — This zip archive contains the TEM images for one WT and one iDKO used for quantitative analysis shown in Figure 5G–I. Images were taken using a JEOL 1010 electron microscope fitted with a Hamamatsu digital camera and AMT Advantage image capture software. Contrast of the images was adjusted using Photoshop software. The images in this archive were also used for the analysis in Figure 7. [file elife-50138-fig5-data1.zip › Figure 5 source data 1/iDKO #492 12d DD 7500X/7.11.17 #492 DD_020 Contrast .tif]

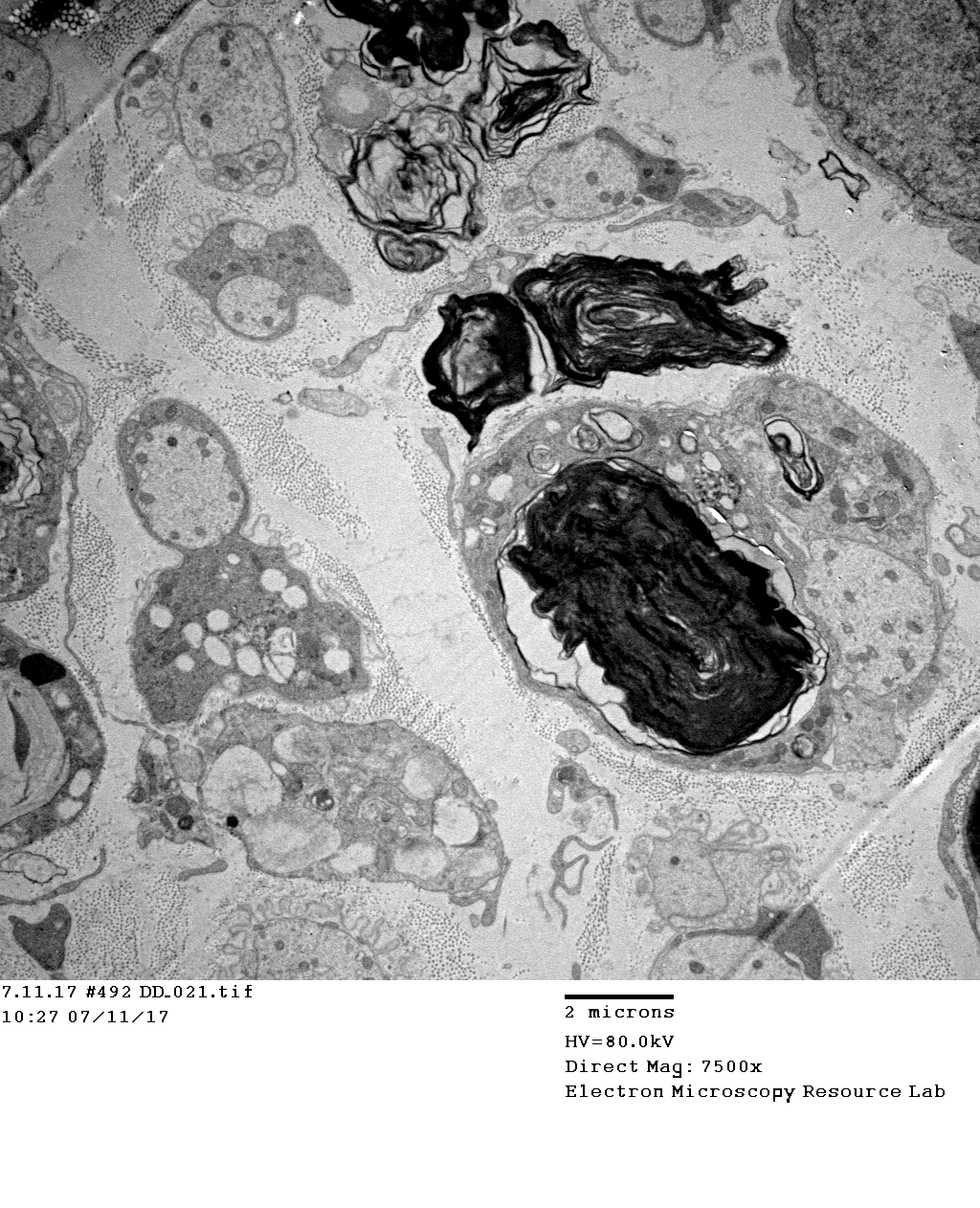

Supplement: Figure 5—source data 1. — This zip archive contains the TEM images for one WT and one iDKO used for quantitative analysis shown in Figure 5G–I. Images were taken using a JEOL 1010 electron microscope fitted with a Hamamatsu digital camera and AMT Advantage image capture software. Contrast of the images was adjusted using Photoshop software. The images in this archive were also used for the analysis in Figure 7. [file elife-50138-fig5-data1.zip › Figure 5 source data 1/iDKO #492 12d DD 7500X/7.11.17 #492 DD_021 Contrast .tif]

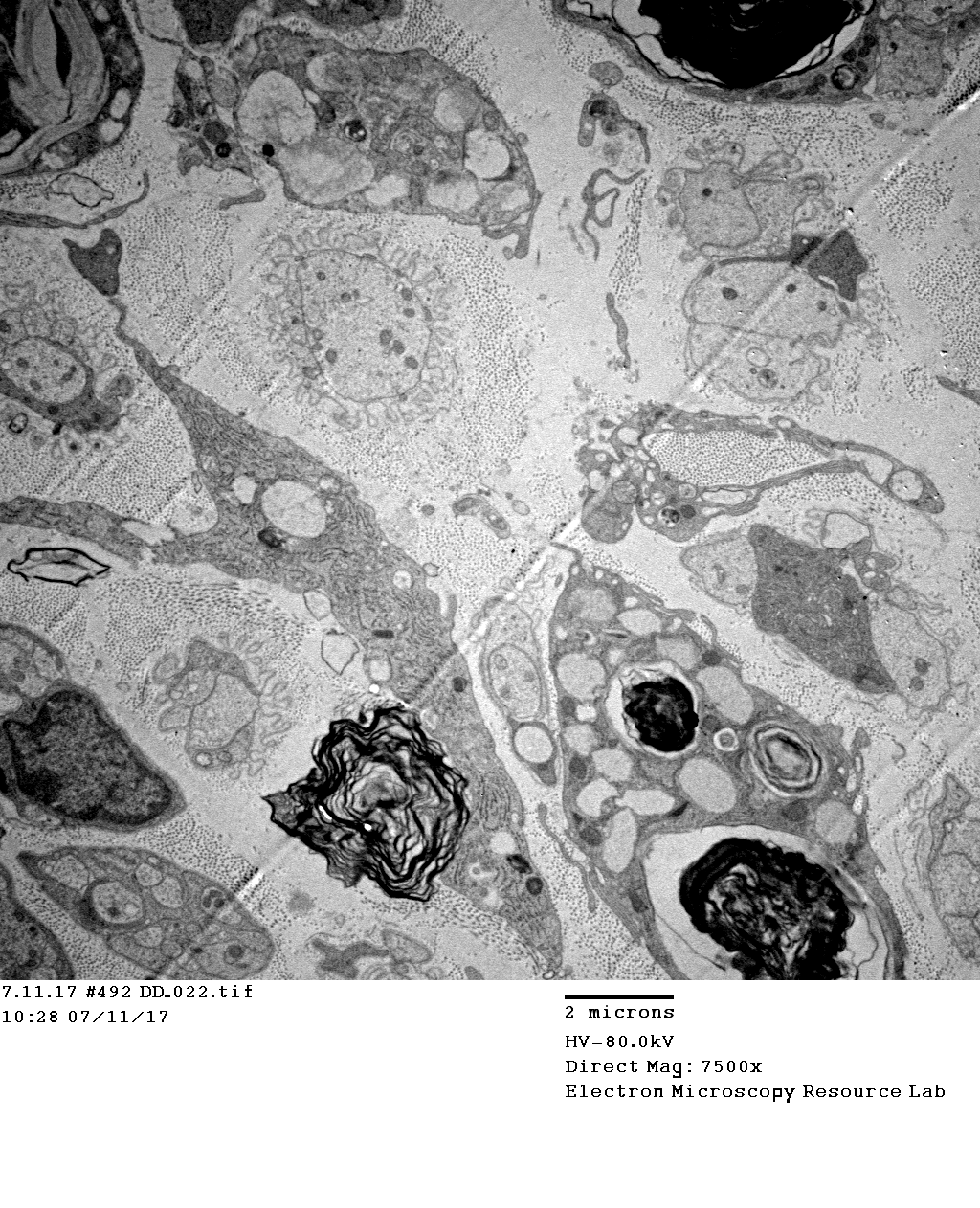

Supplement: Figure 5—source data 1. — This zip archive contains the TEM images for one WT and one iDKO used for quantitative analysis shown in Figure 5G–I. Images were taken using a JEOL 1010 electron microscope fitted with a Hamamatsu digital camera and AMT Advantage image capture software. Contrast of the images was adjusted using Photoshop software. The images in this archive were also used for the analysis in Figure 7. [file elife-50138-fig5-data1.zip › Figure 5 source data 1/iDKO #492 12d DD 7500X/7.11.17 #492 DD_022 Contrast.tif]

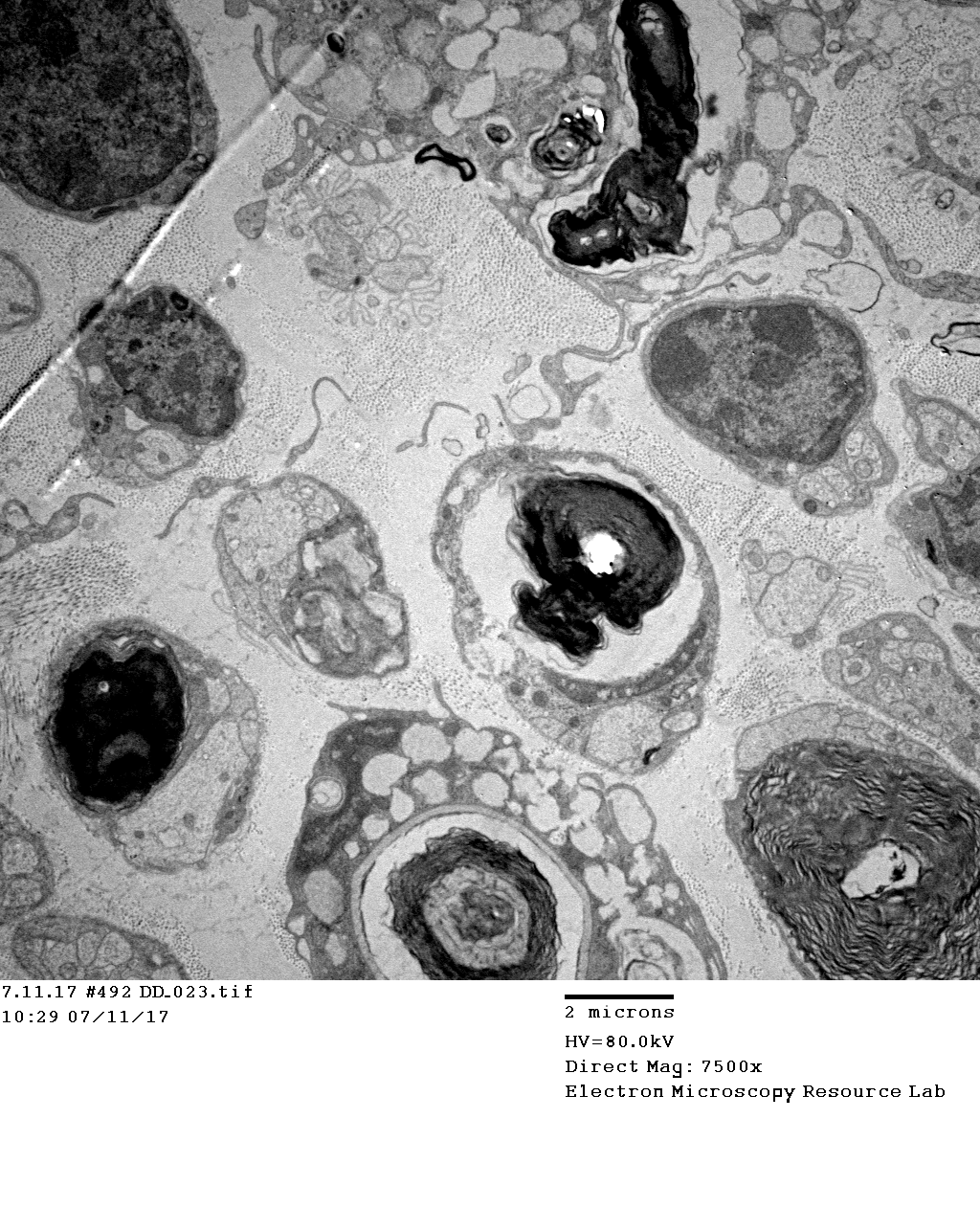

Supplement: Figure 5—source data 1. — This zip archive contains the TEM images for one WT and one iDKO used for quantitative analysis shown in Figure 5G–I. Images were taken using a JEOL 1010 electron microscope fitted with a Hamamatsu digital camera and AMT Advantage image capture software. Contrast of the images was adjusted using Photoshop software. The images in this archive were also used for the analysis in Figure 7. [file elife-50138-fig5-data1.zip › Figure 5 source data 1/iDKO #492 12d DD 7500X/7.11.17 #492 DD_023 Contrast .tif]

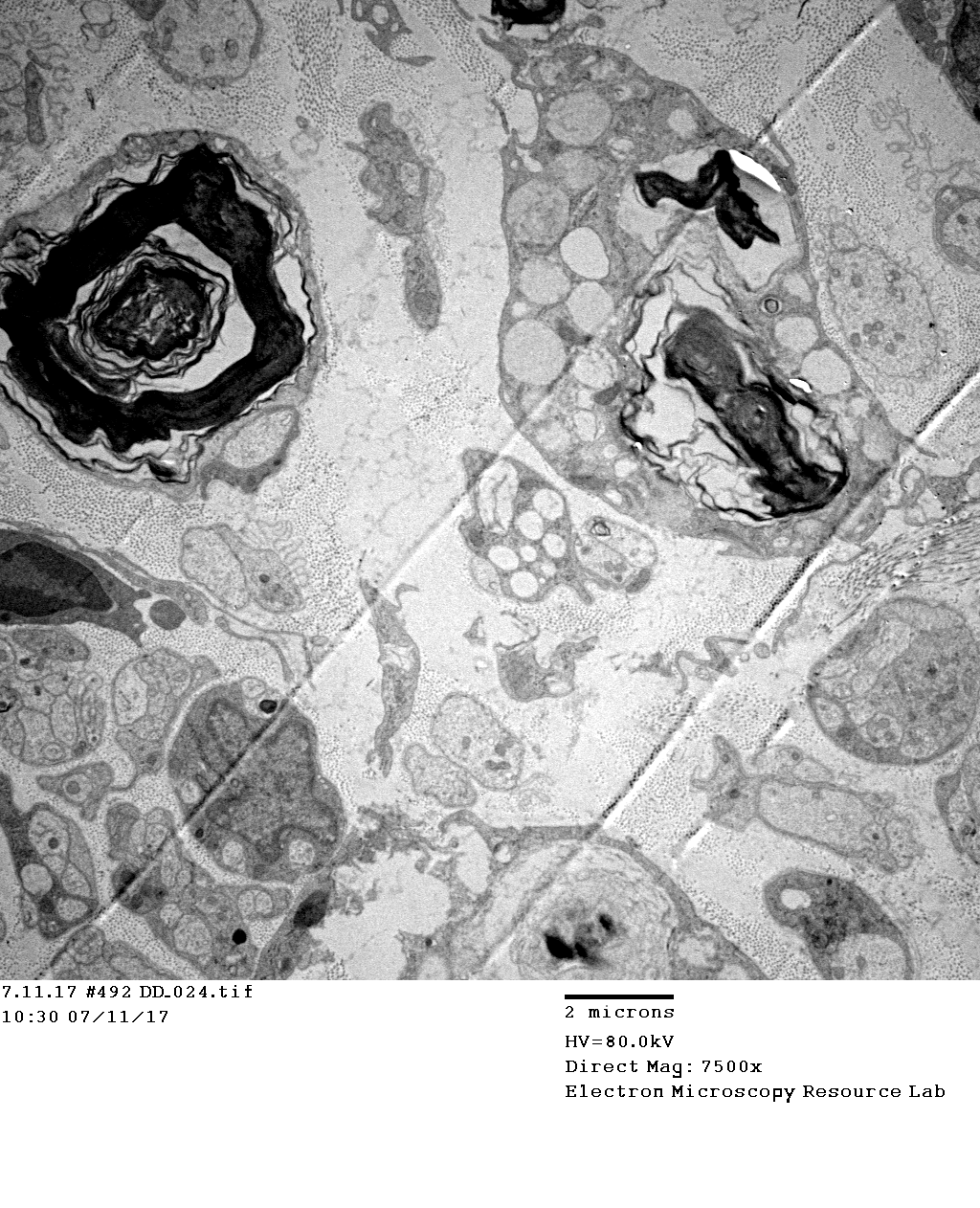

Supplement: Figure 5—source data 1. — This zip archive contains the TEM images for one WT and one iDKO used for quantitative analysis shown in Figure 5G–I. Images were taken using a JEOL 1010 electron microscope fitted with a Hamamatsu digital camera and AMT Advantage image capture software. Contrast of the images was adjusted using Photoshop software. The images in this archive were also used for the analysis in Figure 7. [file elife-50138-fig5-data1.zip › Figure 5 source data 1/iDKO #492 12d DD 7500X/7.11.17 #492 DD_024 Contrast .tif]

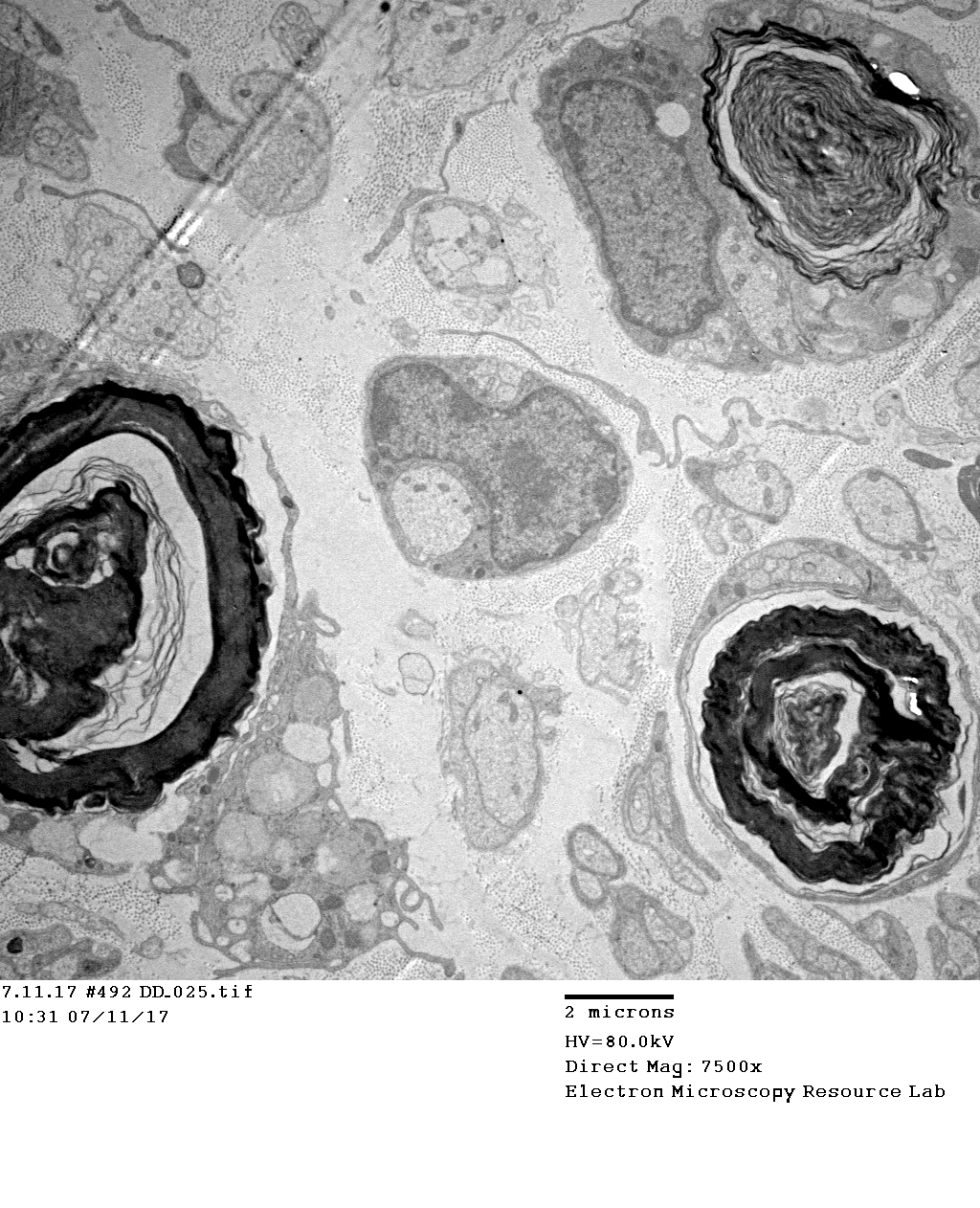

Supplement: Figure 5—source data 1. — This zip archive contains the TEM images for one WT and one iDKO used for quantitative analysis shown in Figure 5G–I. Images were taken using a JEOL 1010 electron microscope fitted with a Hamamatsu digital camera and AMT Advantage image capture software. Contrast of the images was adjusted using Photoshop software. The images in this archive were also used for the analysis in Figure 7. [file elife-50138-fig5-data1.zip › Figure 5 source data 1/iDKO #492 12d DD 7500X/7.11.17 #492 DD_025 Contrast .tif]

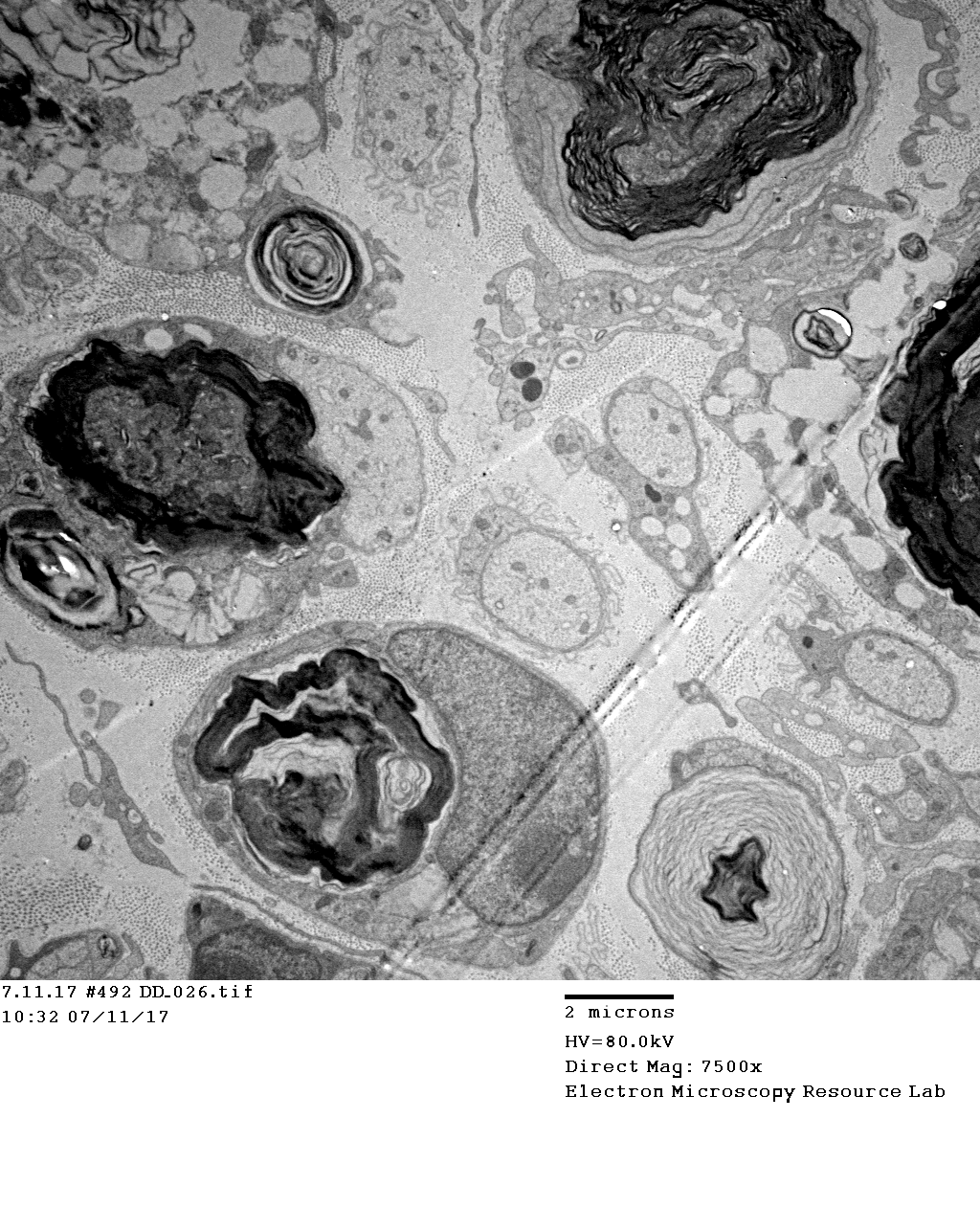

Supplement: Figure 5—source data 1. — This zip archive contains the TEM images for one WT and one iDKO used for quantitative analysis shown in Figure 5G–I. Images were taken using a JEOL 1010 electron microscope fitted with a Hamamatsu digital camera and AMT Advantage image capture software. Contrast of the images was adjusted using Photoshop software. The images in this archive were also used for the analysis in Figure 7. [file elife-50138-fig5-data1.zip › Figure 5 source data 1/iDKO #492 12d DD 7500X/7.11.17 #492 DD_026 Contrast .tif]

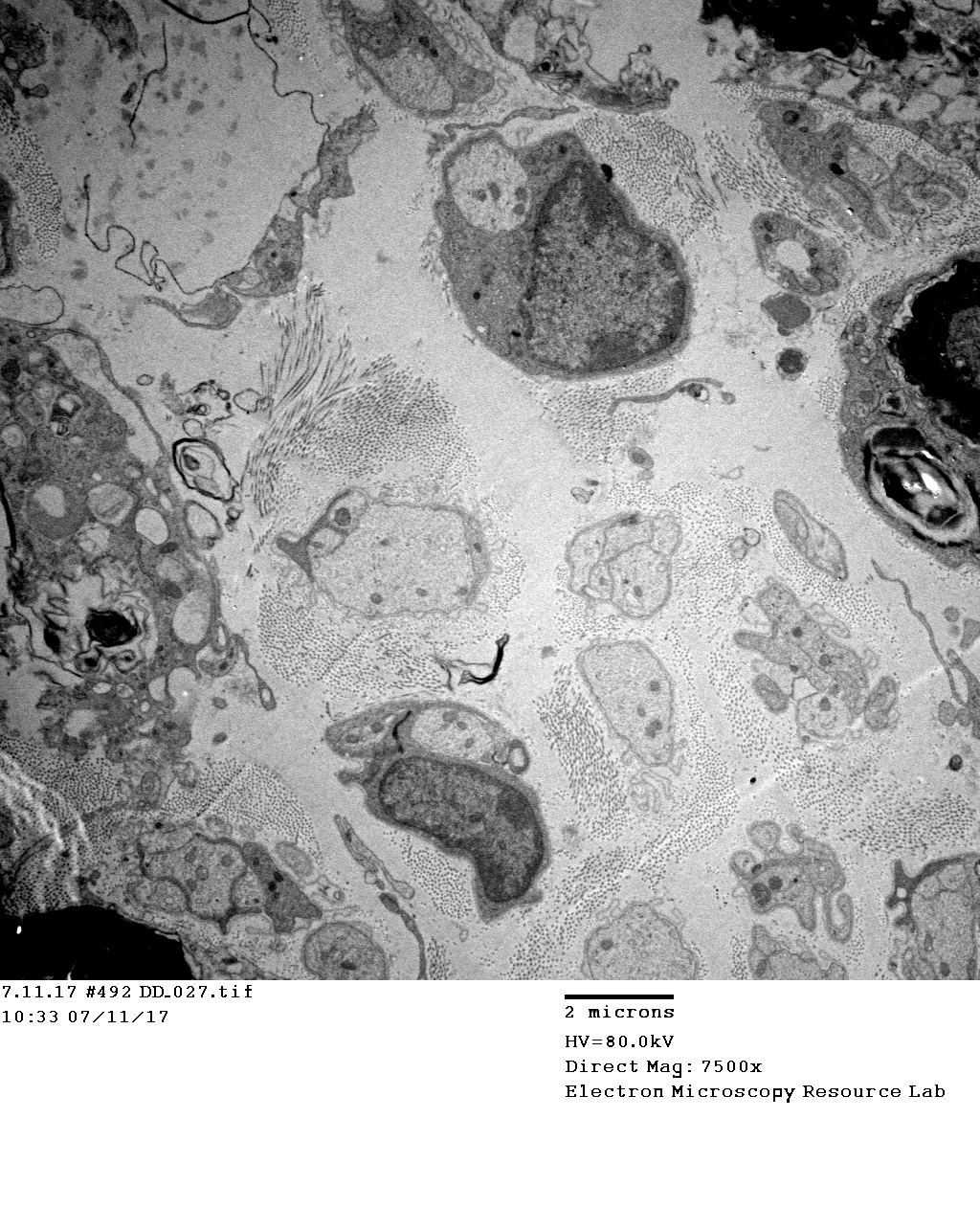

Supplement: Figure 5—source data 1. — This zip archive contains the TEM images for one WT and one iDKO used for quantitative analysis shown in Figure 5G–I. Images were taken using a JEOL 1010 electron microscope fitted with a Hamamatsu digital camera and AMT Advantage image capture software. Contrast of the images was adjusted using Photoshop software. The images in this archive were also used for the analysis in Figure 7. [file elife-50138-fig5-data1.zip › Figure 5 source data 1/iDKO #492 12d DD 7500X/7.11.17 #492 DD_027 Contrast .tif]

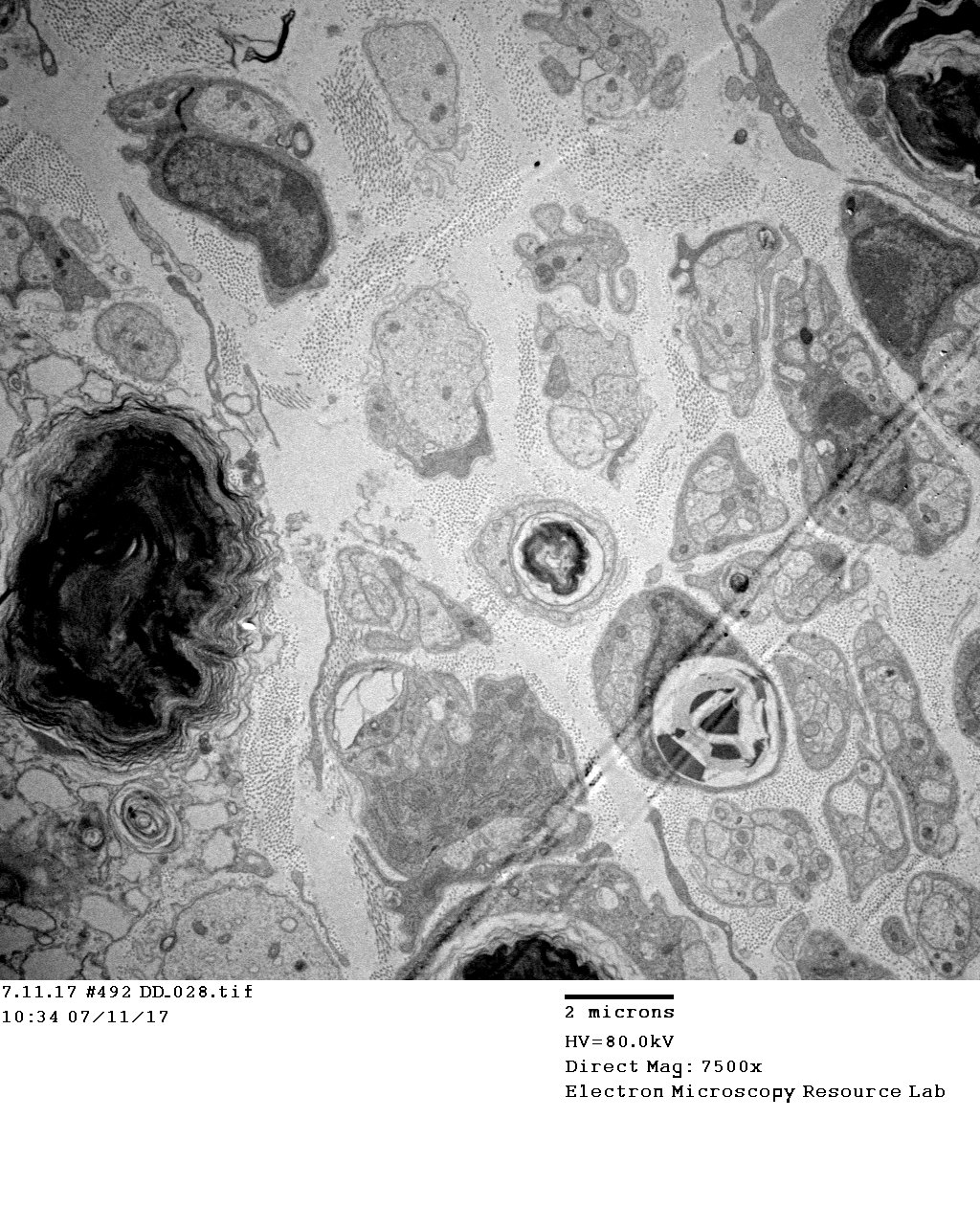

Supplement: Figure 5—source data 1. — This zip archive contains the TEM images for one WT and one iDKO used for quantitative analysis shown in Figure 5G–I. Images were taken using a JEOL 1010 electron microscope fitted with a Hamamatsu digital camera and AMT Advantage image capture software. Contrast of the images was adjusted using Photoshop software. The images in this archive were also used for the analysis in Figure 7. [file elife-50138-fig5-data1.zip › Figure 5 source data 1/iDKO #492 12d DD 7500X/7.11.17 #492 DD_028 Contrast .tif]

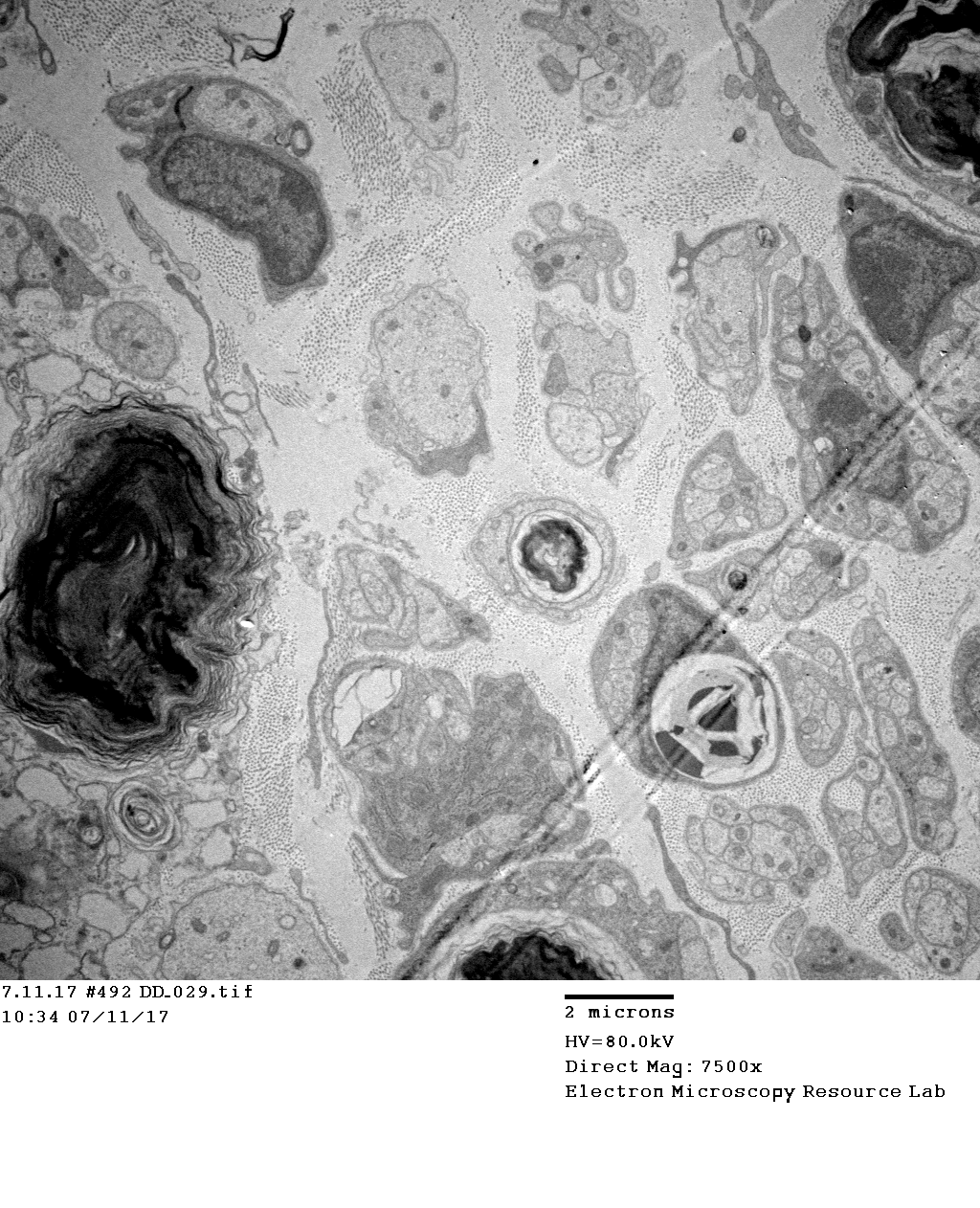

Supplement: Figure 5—source data 1. — This zip archive contains the TEM images for one WT and one iDKO used for quantitative analysis shown in Figure 5G–I. Images were taken using a JEOL 1010 electron microscope fitted with a Hamamatsu digital camera and AMT Advantage image capture software. Contrast of the images was adjusted using Photoshop software. The images in this archive were also used for the analysis in Figure 7. [file elife-50138-fig5-data1.zip › Figure 5 source data 1/iDKO #492 12d DD 7500X/7.11.17 #492 DD_029 Contrast .tif]

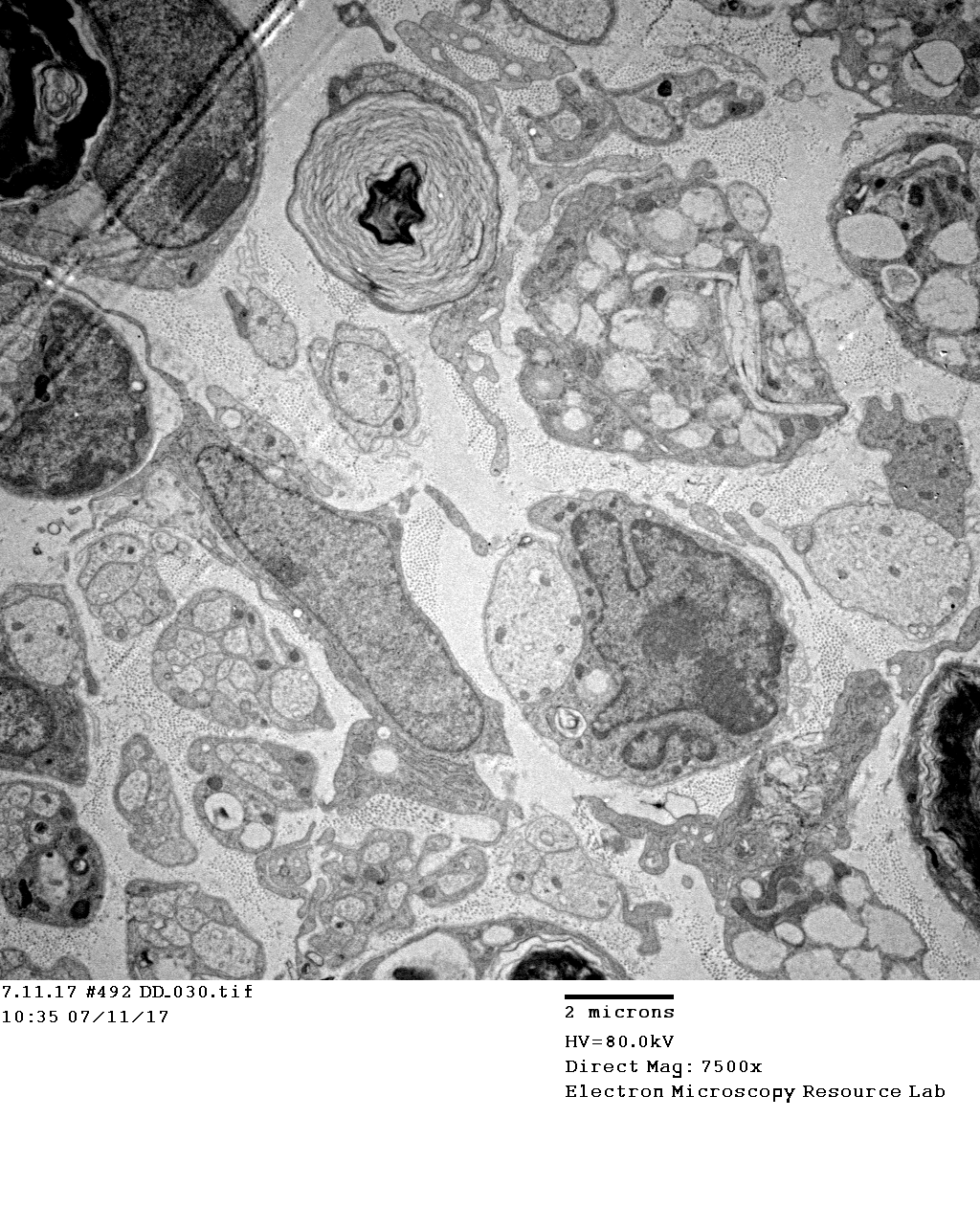

Supplement: Figure 5—source data 1. — This zip archive contains the TEM images for one WT and one iDKO used for quantitative analysis shown in Figure 5G–I. Images were taken using a JEOL 1010 electron microscope fitted with a Hamamatsu digital camera and AMT Advantage image capture software. Contrast of the images was adjusted using Photoshop software. The images in this archive were also used for the analysis in Figure 7. [file elife-50138-fig5-data1.zip › Figure 5 source data 1/iDKO #492 12d DD 7500X/7.11.17 #492 DD_030 Contrast .tif]

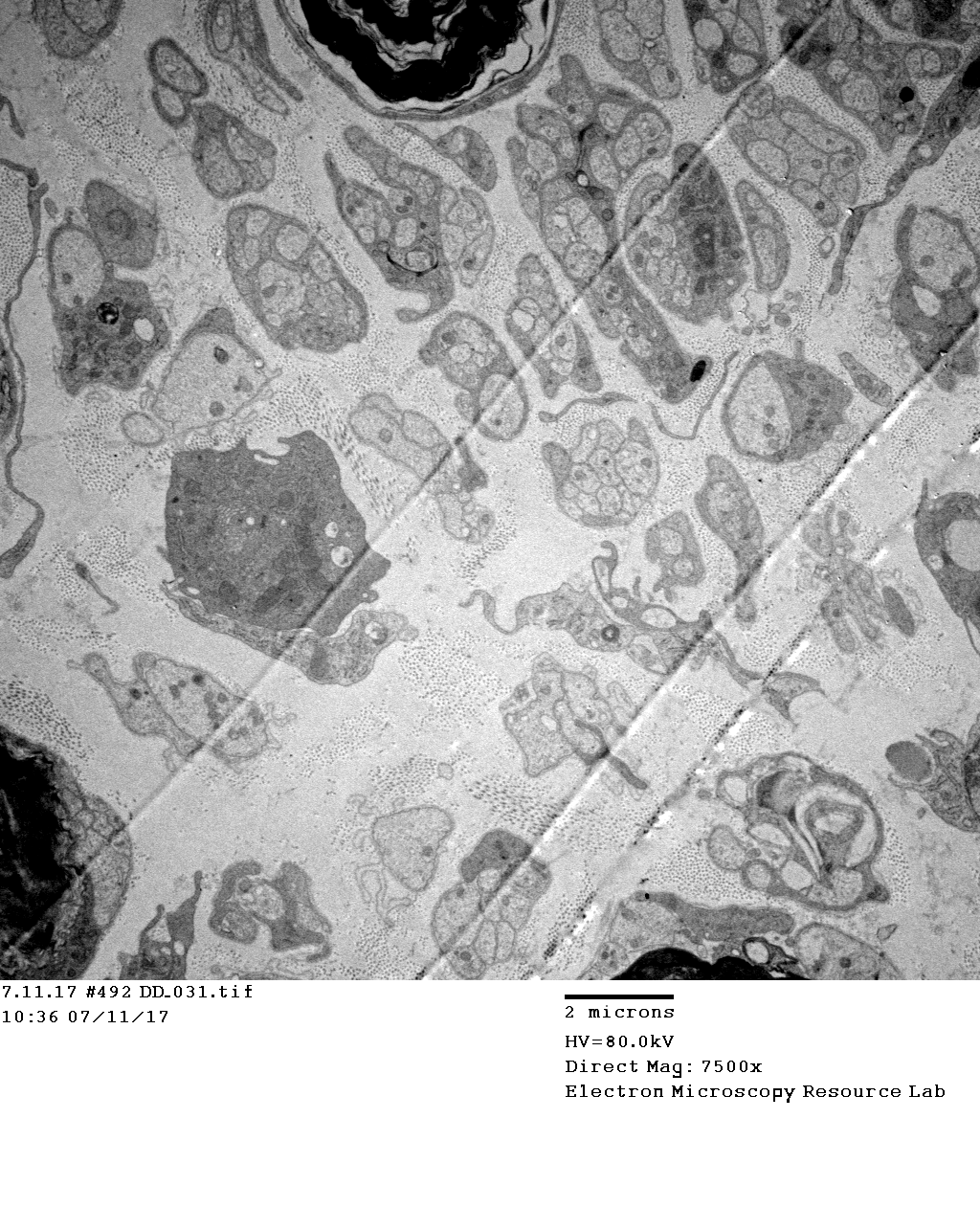

Supplement: Figure 5—source data 1. — This zip archive contains the TEM images for one WT and one iDKO used for quantitative analysis shown in Figure 5G–I. Images were taken using a JEOL 1010 electron microscope fitted with a Hamamatsu digital camera and AMT Advantage image capture software. Contrast of the images was adjusted using Photoshop software. The images in this archive were also used for the analysis in Figure 7. [file elife-50138-fig5-data1.zip › Figure 5 source data 1/iDKO #492 12d DD 7500X/7.11.17 #492 DD_031 Contrast .tif]

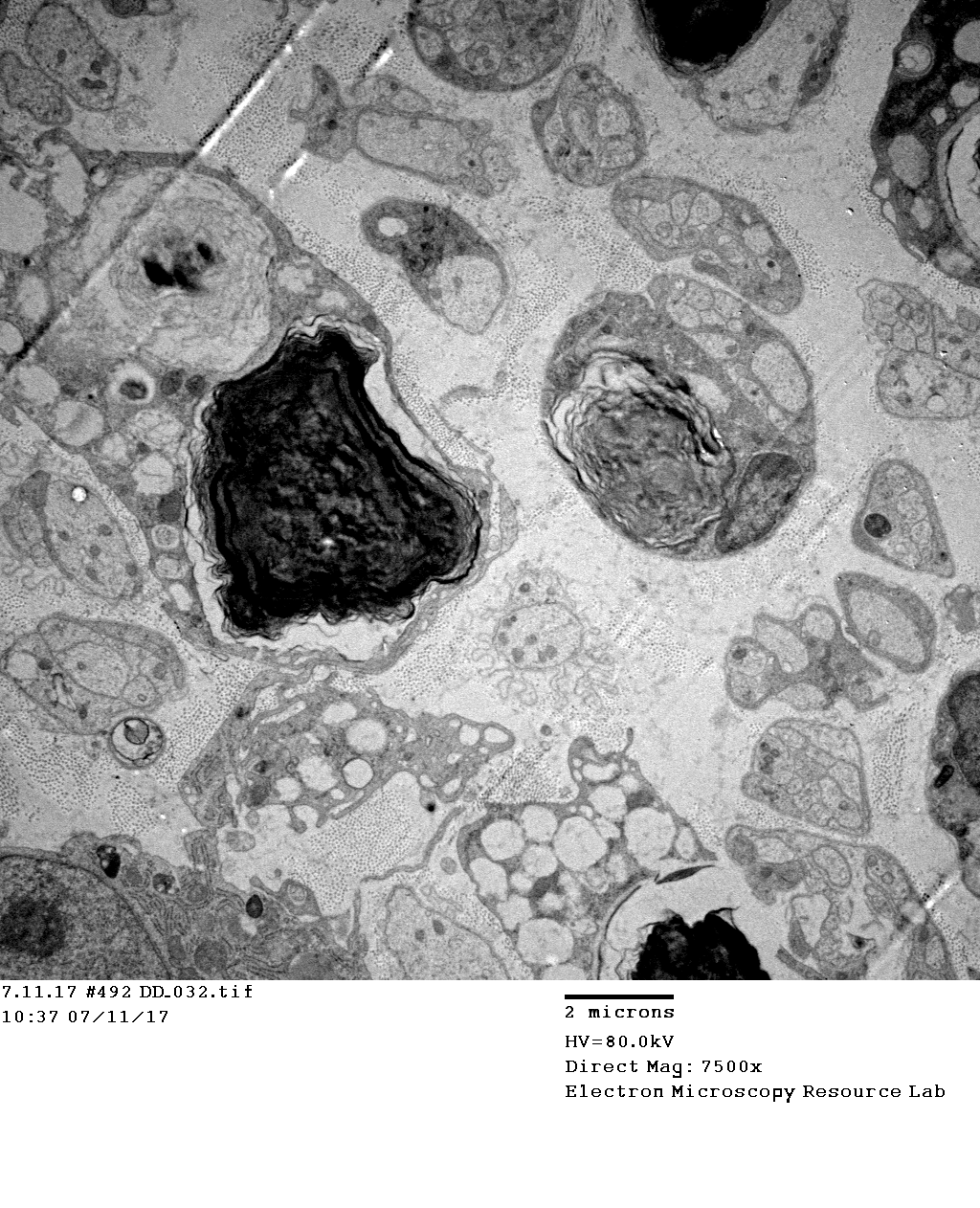

Supplement: Figure 5—source data 1. — This zip archive contains the TEM images for one WT and one iDKO used for quantitative analysis shown in Figure 5G–I. Images were taken using a JEOL 1010 electron microscope fitted with a Hamamatsu digital camera and AMT Advantage image capture software. Contrast of the images was adjusted using Photoshop software. The images in this archive were also used for the analysis in Figure 7. [file elife-50138-fig5-data1.zip › Figure 5 source data 1/iDKO #492 12d DD 7500X/7.11.17 #492 DD_032 Contrast .tif]

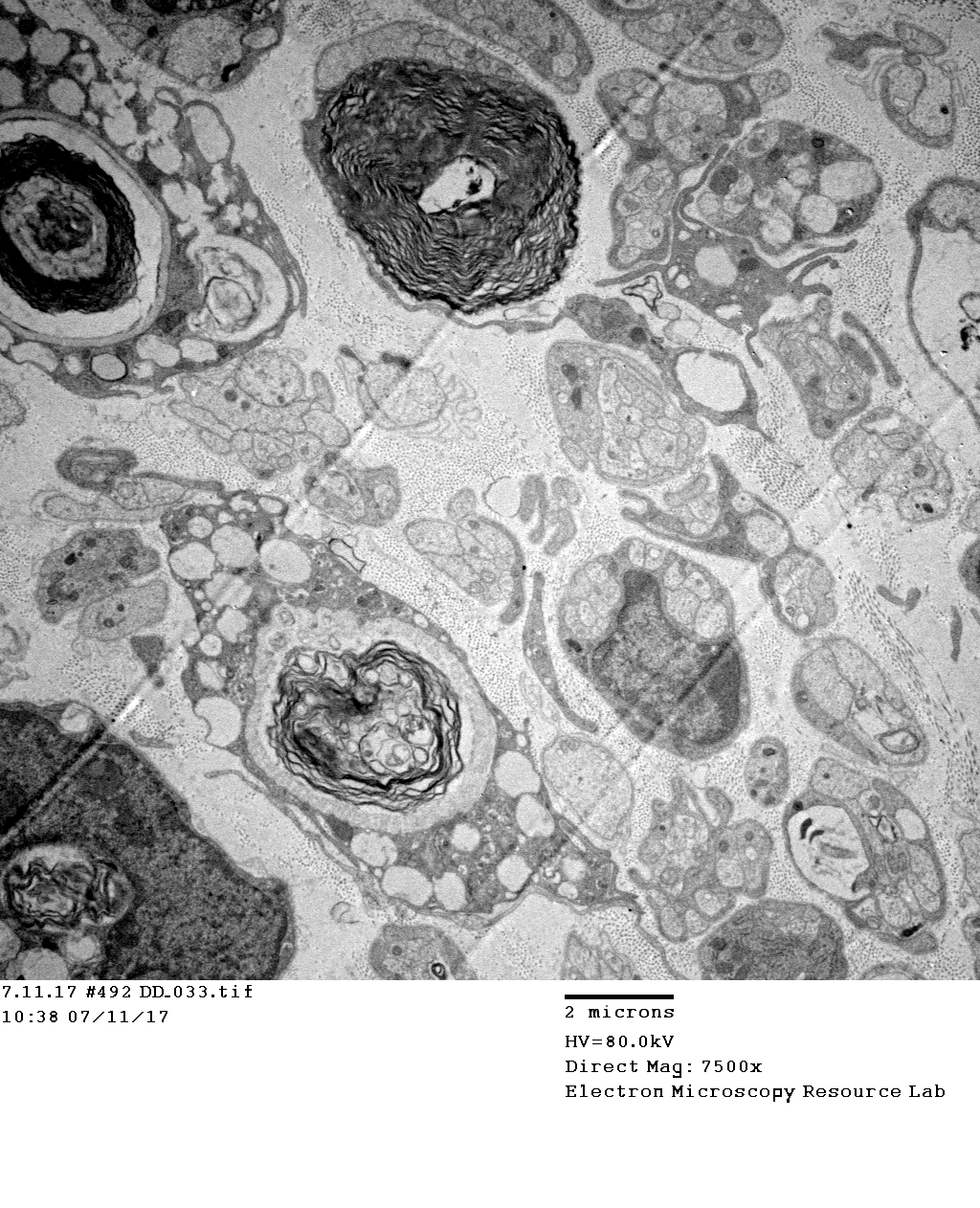

Supplement: Figure 5—source data 1. — This zip archive contains the TEM images for one WT and one iDKO used for quantitative analysis shown in Figure 5G–I. Images were taken using a JEOL 1010 electron microscope fitted with a Hamamatsu digital camera and AMT Advantage image capture software. Contrast of the images was adjusted using Photoshop software. The images in this archive were also used for the analysis in Figure 7. [file elife-50138-fig5-data1.zip › Figure 5 source data 1/iDKO #492 12d DD 7500X/7.11.17 #492 DD_033 Contrast .tif]

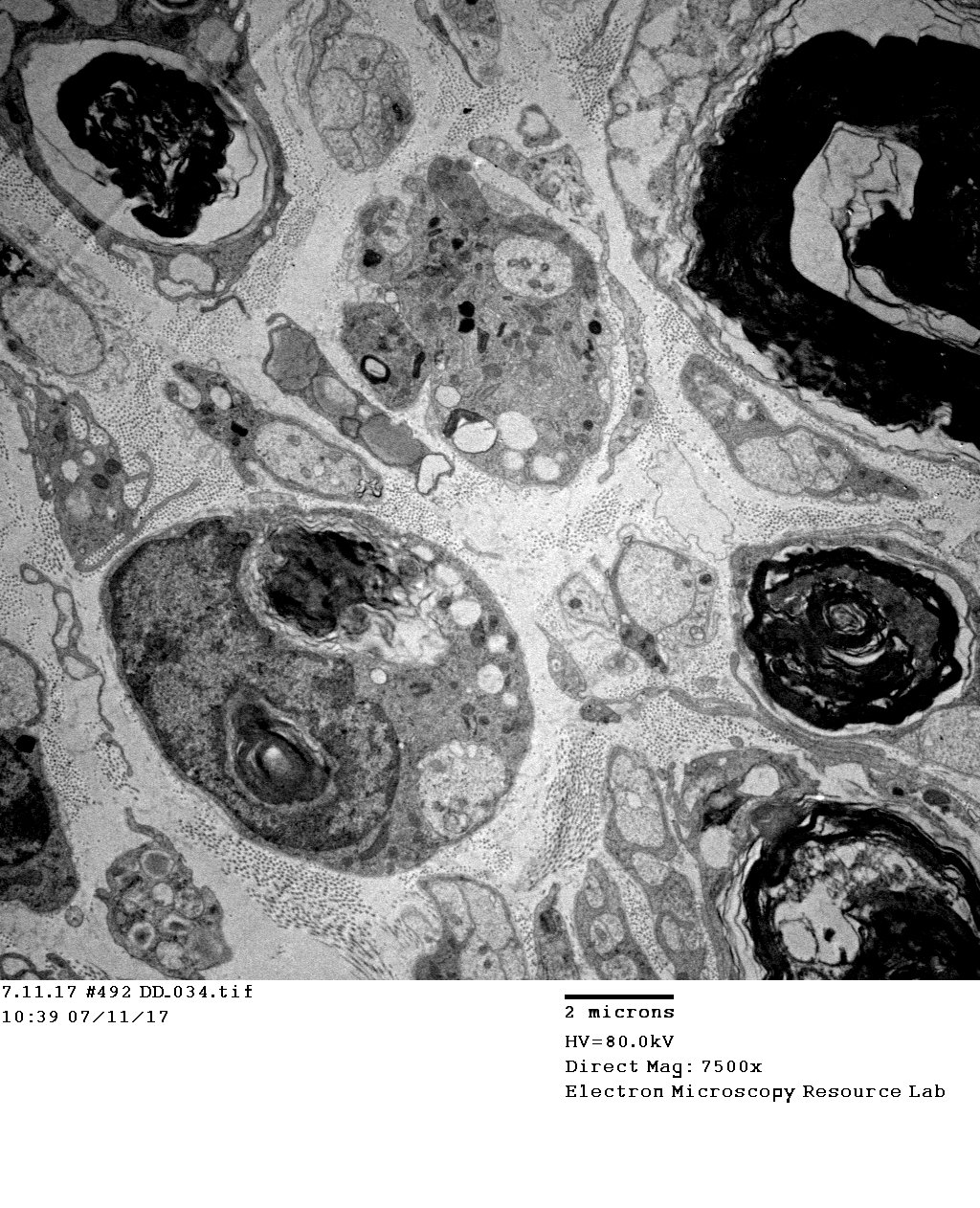

Supplement: Figure 5—source data 1. — This zip archive contains the TEM images for one WT and one iDKO used for quantitative analysis shown in Figure 5G–I. Images were taken using a JEOL 1010 electron microscope fitted with a Hamamatsu digital camera and AMT Advantage image capture software. Contrast of the images was adjusted using Photoshop software. The images in this archive were also used for the analysis in Figure 7. [file elife-50138-fig5-data1.zip › Figure 5 source data 1/iDKO #492 12d DD 7500X/7.11.17 #492 DD_034 Contrast .tif]

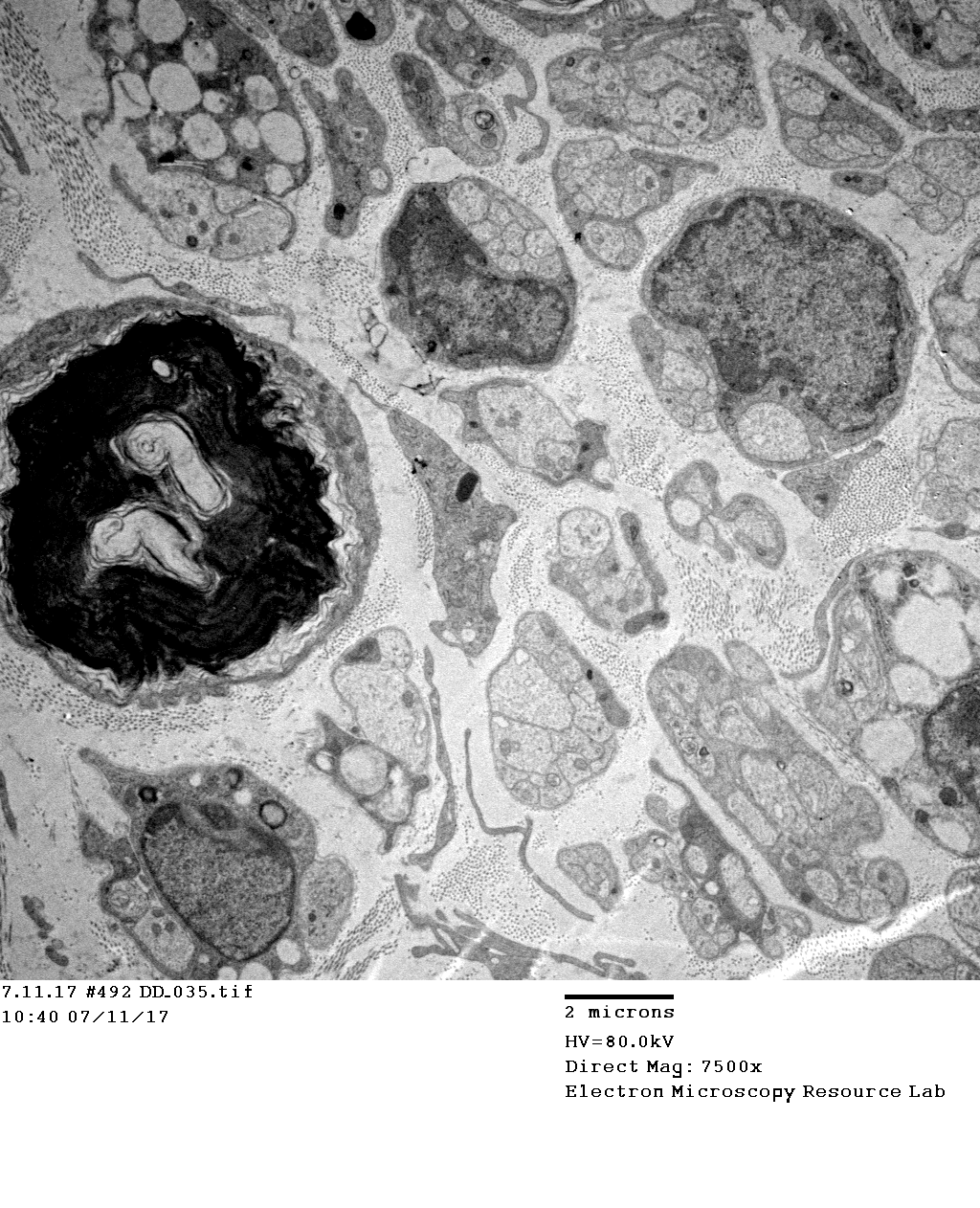

Supplement: Figure 5—source data 1. — This zip archive contains the TEM images for one WT and one iDKO used for quantitative analysis shown in Figure 5G–I. Images were taken using a JEOL 1010 electron microscope fitted with a Hamamatsu digital camera and AMT Advantage image capture software. Contrast of the images was adjusted using Photoshop software. The images in this archive were also used for the analysis in Figure 7. [file elife-50138-fig5-data1.zip › Figure 5 source data 1/iDKO #492 12d DD 7500X/7.11.17 #492 DD_035 Contrast .tif]

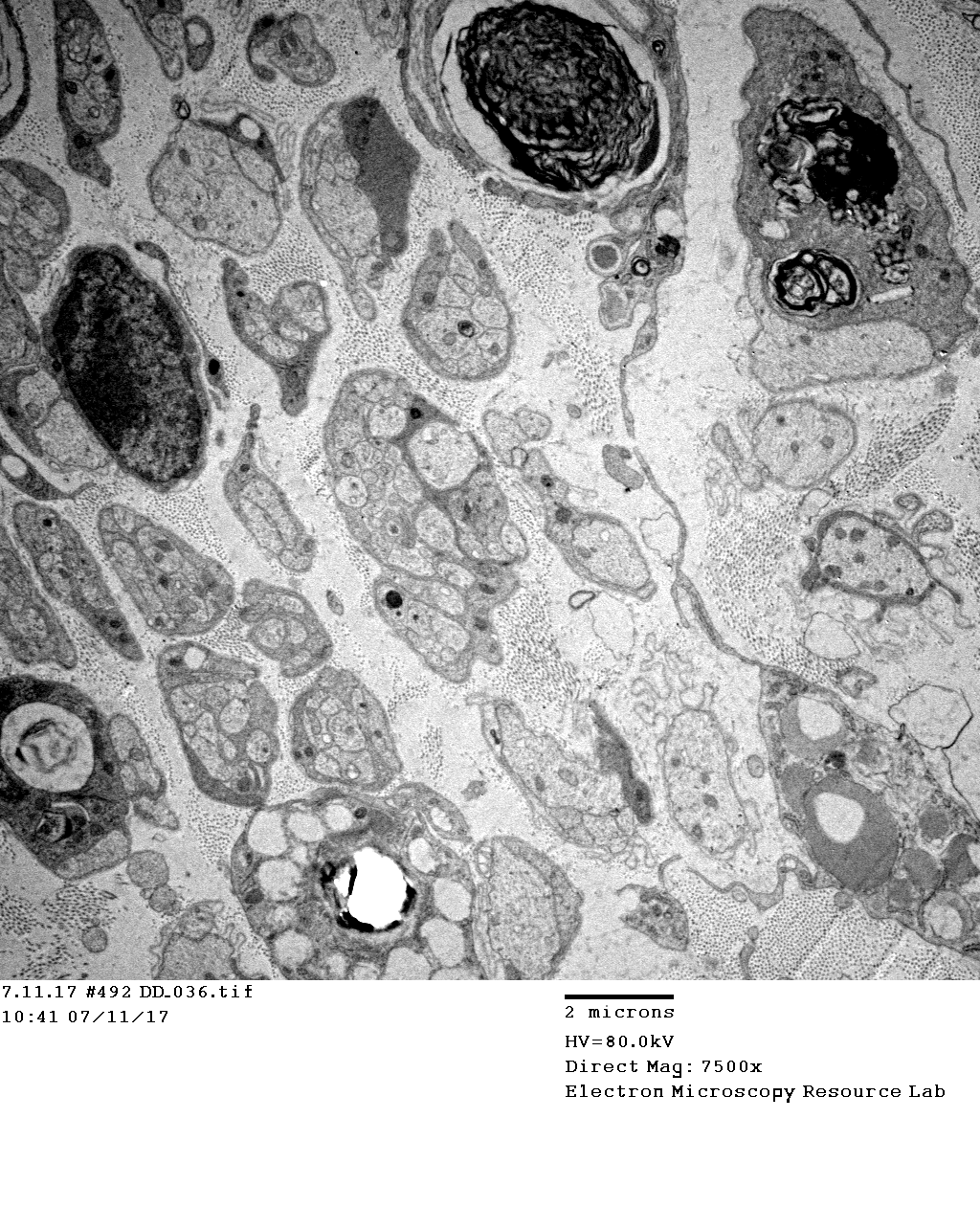

Supplement: Figure 5—source data 1. — This zip archive contains the TEM images for one WT and one iDKO used for quantitative analysis shown in Figure 5G–I. Images were taken using a JEOL 1010 electron microscope fitted with a Hamamatsu digital camera and AMT Advantage image capture software. Contrast of the images was adjusted using Photoshop software. The images in this archive were also used for the analysis in Figure 7. [file elife-50138-fig5-data1.zip › Figure 5 source data 1/iDKO #492 12d DD 7500X/7.11.17 #492 DD_036 Contrast .tif]

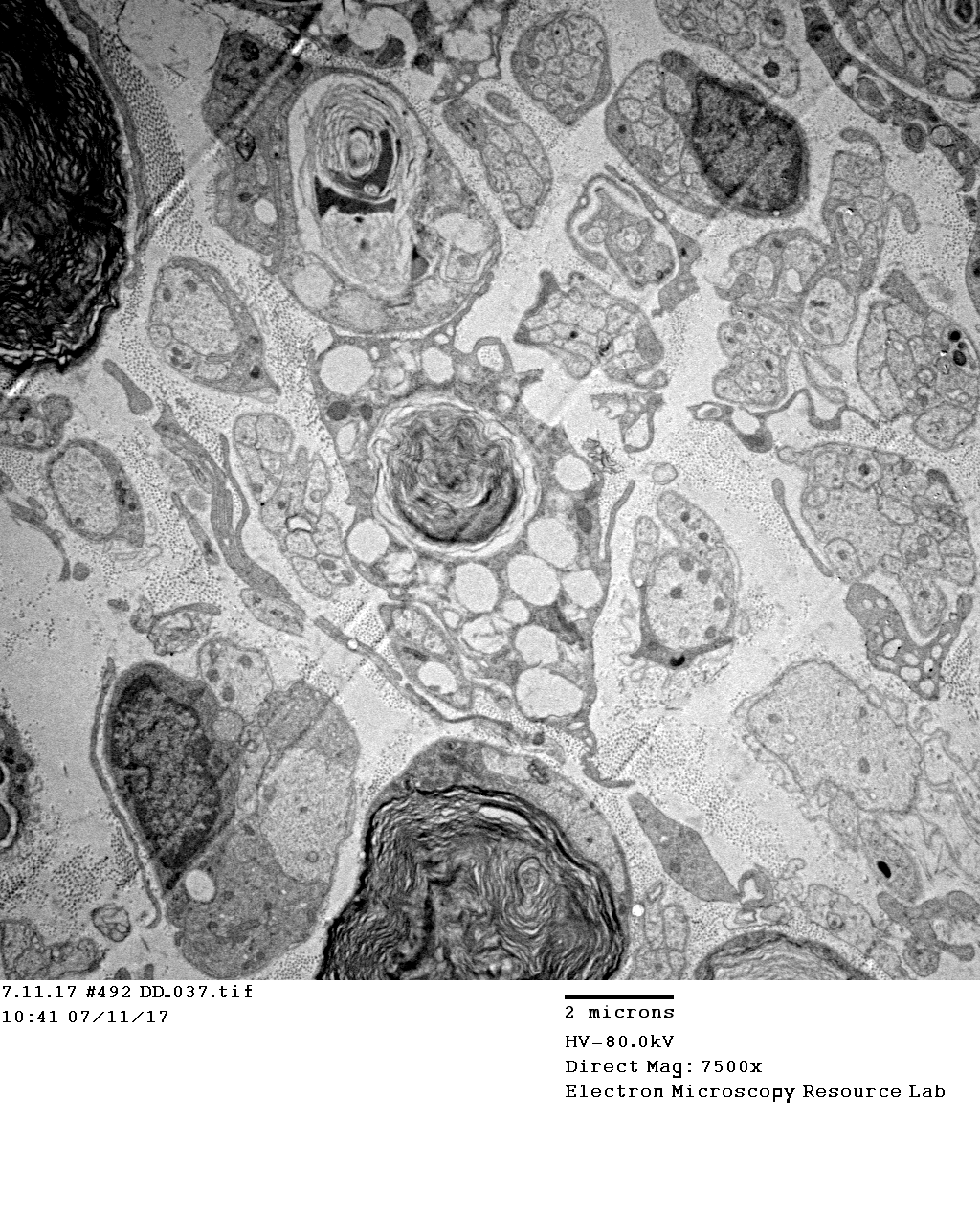

Supplement: Figure 5—source data 1. — This zip archive contains the TEM images for one WT and one iDKO used for quantitative analysis shown in Figure 5G–I. Images were taken using a JEOL 1010 electron microscope fitted with a Hamamatsu digital camera and AMT Advantage image capture software. Contrast of the images was adjusted using Photoshop software. The images in this archive were also used for the analysis in Figure 7. [file elife-50138-fig5-data1.zip › Figure 5 source data 1/iDKO #492 12d DD 7500X/7.11.17 #492 DD_037 Contrast .tif]

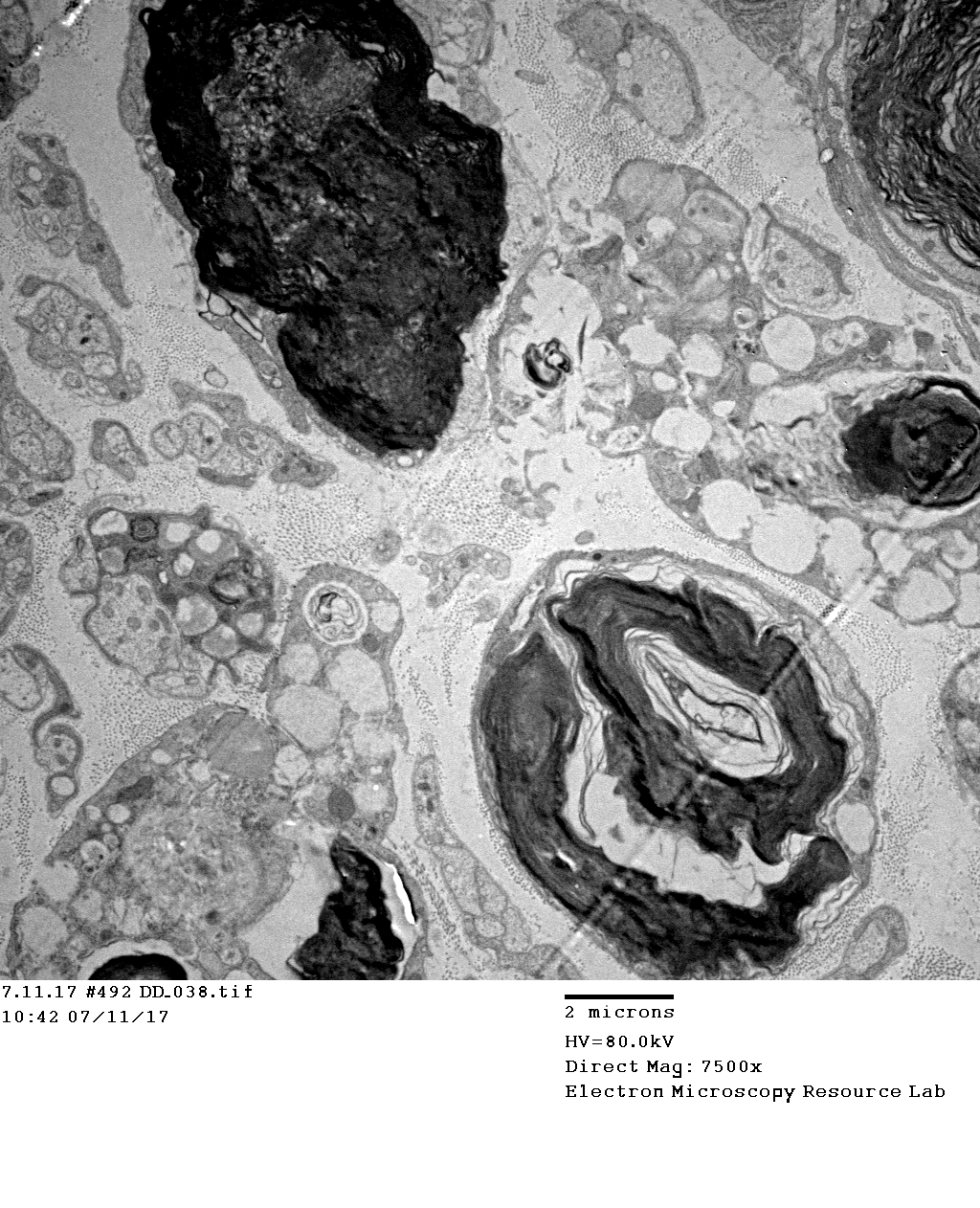

Supplement: Figure 5—source data 1. — This zip archive contains the TEM images for one WT and one iDKO used for quantitative analysis shown in Figure 5G–I. Images were taken using a JEOL 1010 electron microscope fitted with a Hamamatsu digital camera and AMT Advantage image capture software. Contrast of the images was adjusted using Photoshop software. The images in this archive were also used for the analysis in Figure 7. [file elife-50138-fig5-data1.zip › Figure 5 source data 1/iDKO #492 12d DD 7500X/7.11.17 #492 DD_038 Contrast .tif]

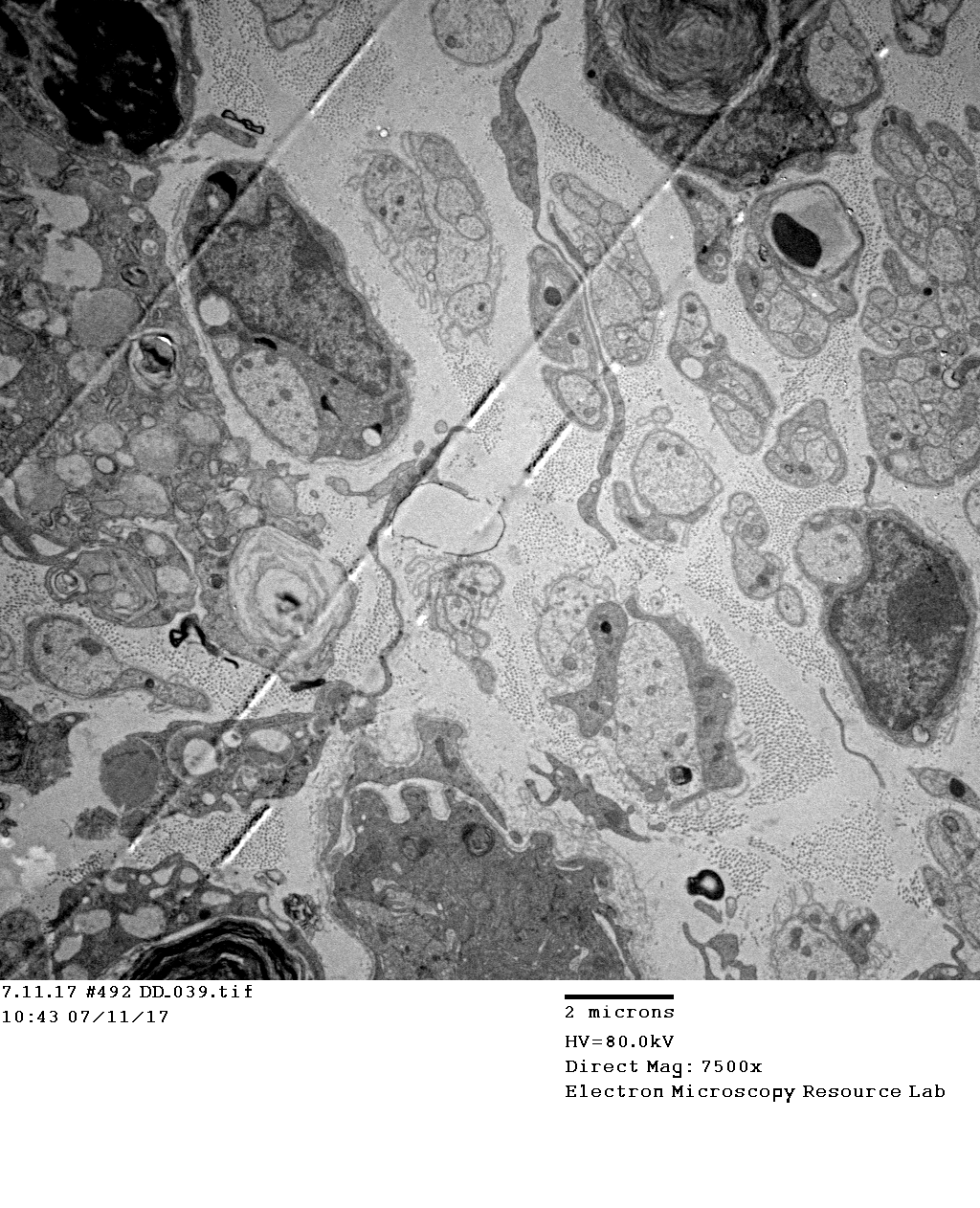

Supplement: Figure 5—source data 1. — This zip archive contains the TEM images for one WT and one iDKO used for quantitative analysis shown in Figure 5G–I. Images were taken using a JEOL 1010 electron microscope fitted with a Hamamatsu digital camera and AMT Advantage image capture software. Contrast of the images was adjusted using Photoshop software. The images in this archive were also used for the analysis in Figure 7. [file elife-50138-fig5-data1.zip › Figure 5 source data 1/iDKO #492 12d DD 7500X/7.11.17 #492 DD_039 Contrast .tif]

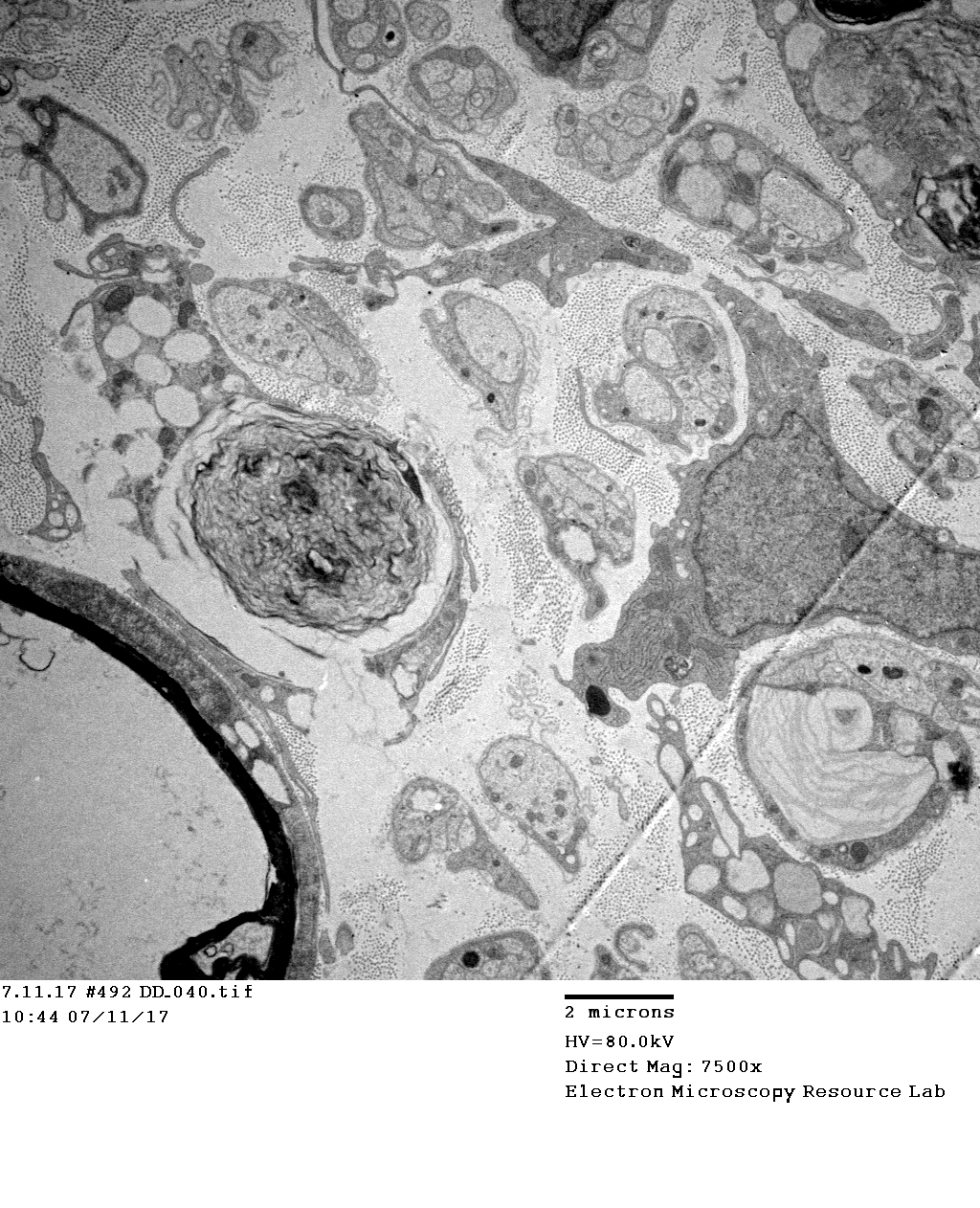

Supplement: Figure 5—source data 1. — This zip archive contains the TEM images for one WT and one iDKO used for quantitative analysis shown in Figure 5G–I. Images were taken using a JEOL 1010 electron microscope fitted with a Hamamatsu digital camera and AMT Advantage image capture software. Contrast of the images was adjusted using Photoshop software. The images in this archive were also used for the analysis in Figure 7. [file elife-50138-fig5-data1.zip › Figure 5 source data 1/iDKO #492 12d DD 7500X/7.11.17 #492 DD_040 Contrast .tif]

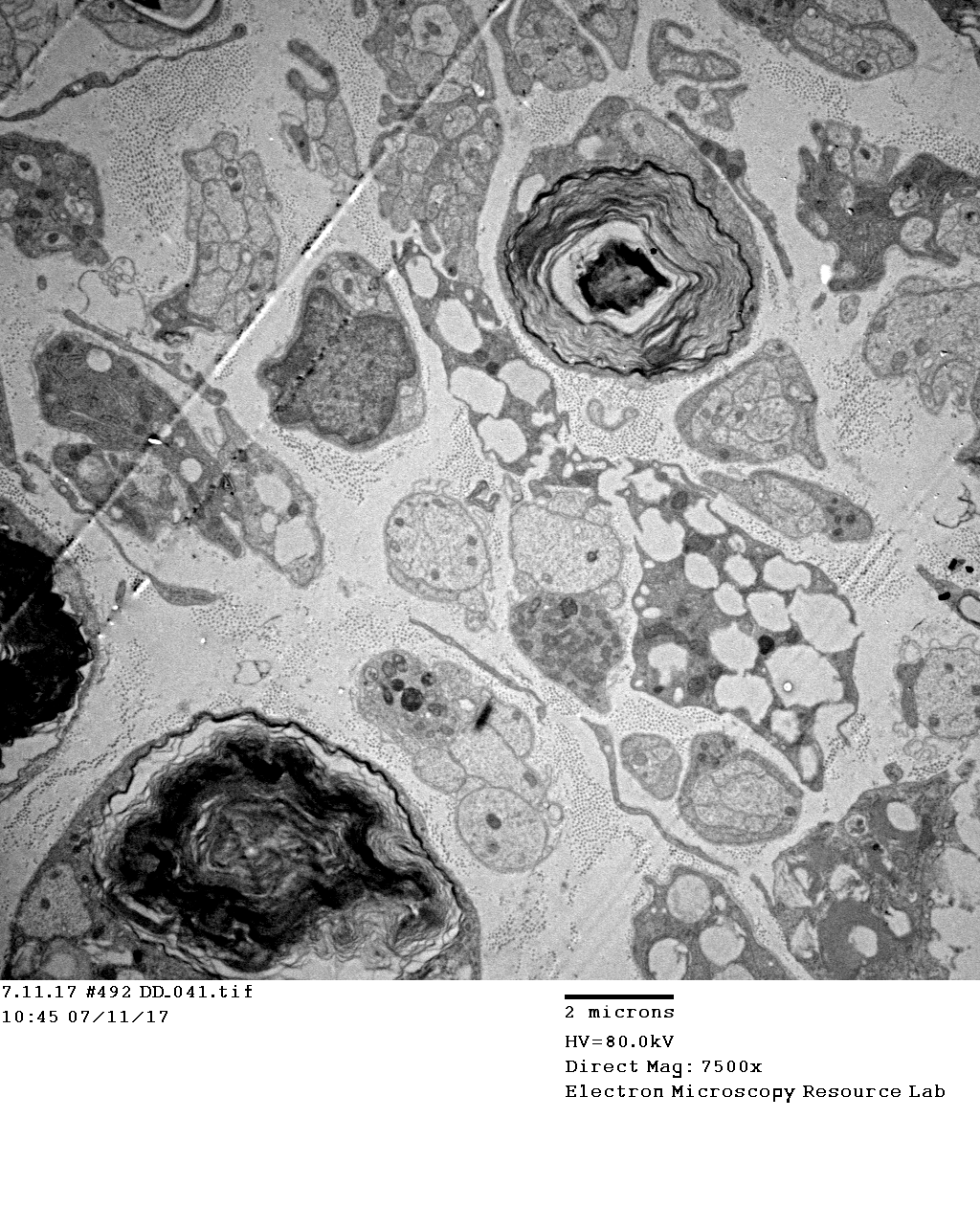

Supplement: Figure 5—source data 1. — This zip archive contains the TEM images for one WT and one iDKO used for quantitative analysis shown in Figure 5G–I. Images were taken using a JEOL 1010 electron microscope fitted with a Hamamatsu digital camera and AMT Advantage image capture software. Contrast of the images was adjusted using Photoshop software. The images in this archive were also used for the analysis in Figure 7. [file elife-50138-fig5-data1.zip › Figure 5 source data 1/iDKO #492 12d DD 7500X/7.11.17 #492 DD_041 Contrast .tif]

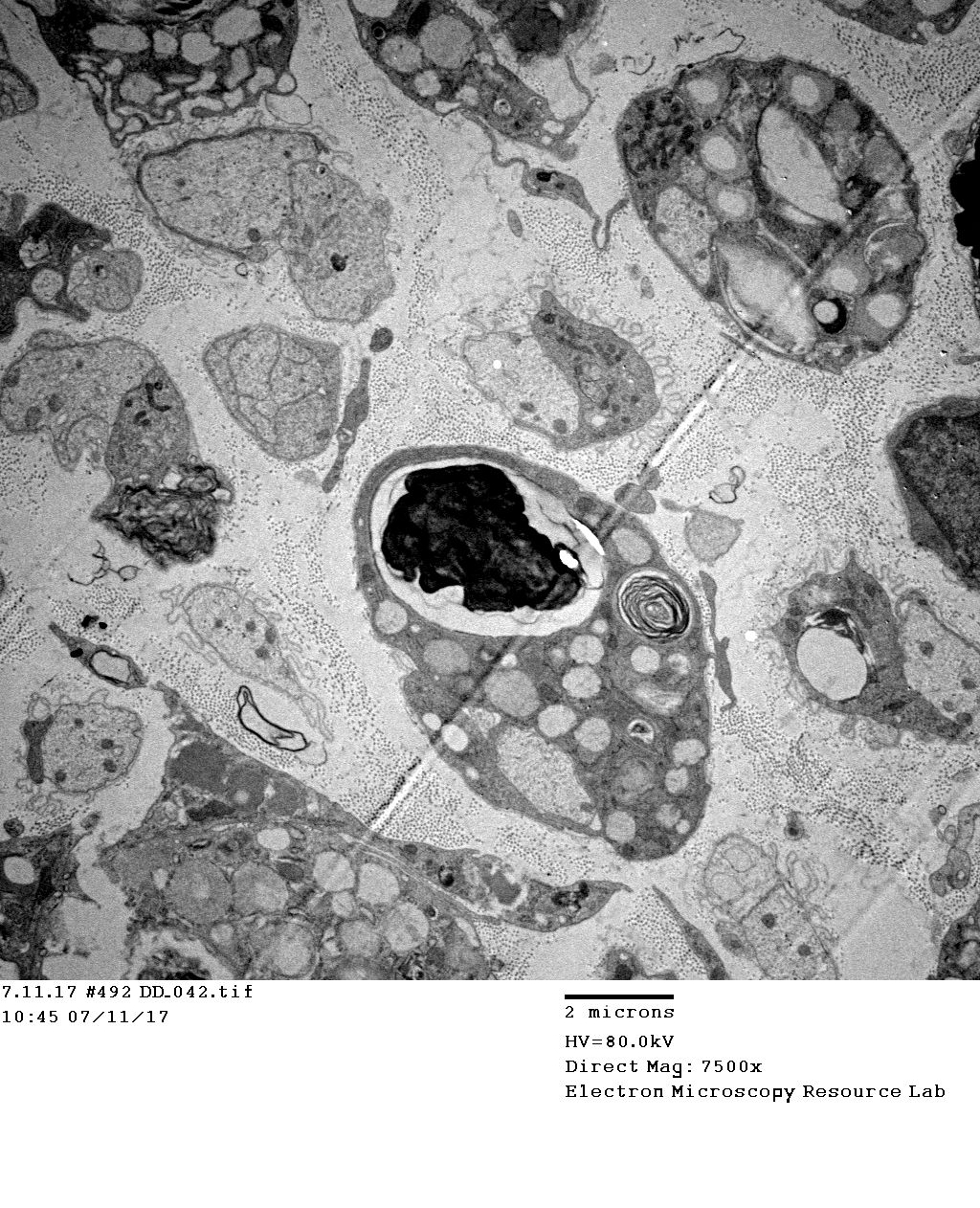

Supplement: Figure 5—source data 1. — This zip archive contains the TEM images for one WT and one iDKO used for quantitative analysis shown in Figure 5G–I. Images were taken using a JEOL 1010 electron microscope fitted with a Hamamatsu digital camera and AMT Advantage image capture software. Contrast of the images was adjusted using Photoshop software. The images in this archive were also used for the analysis in Figure 7. [file elife-50138-fig5-data1.zip › Figure 5 source data 1/iDKO #492 12d DD 7500X/7.11.17 #492 DD_042 Contrast .tif]

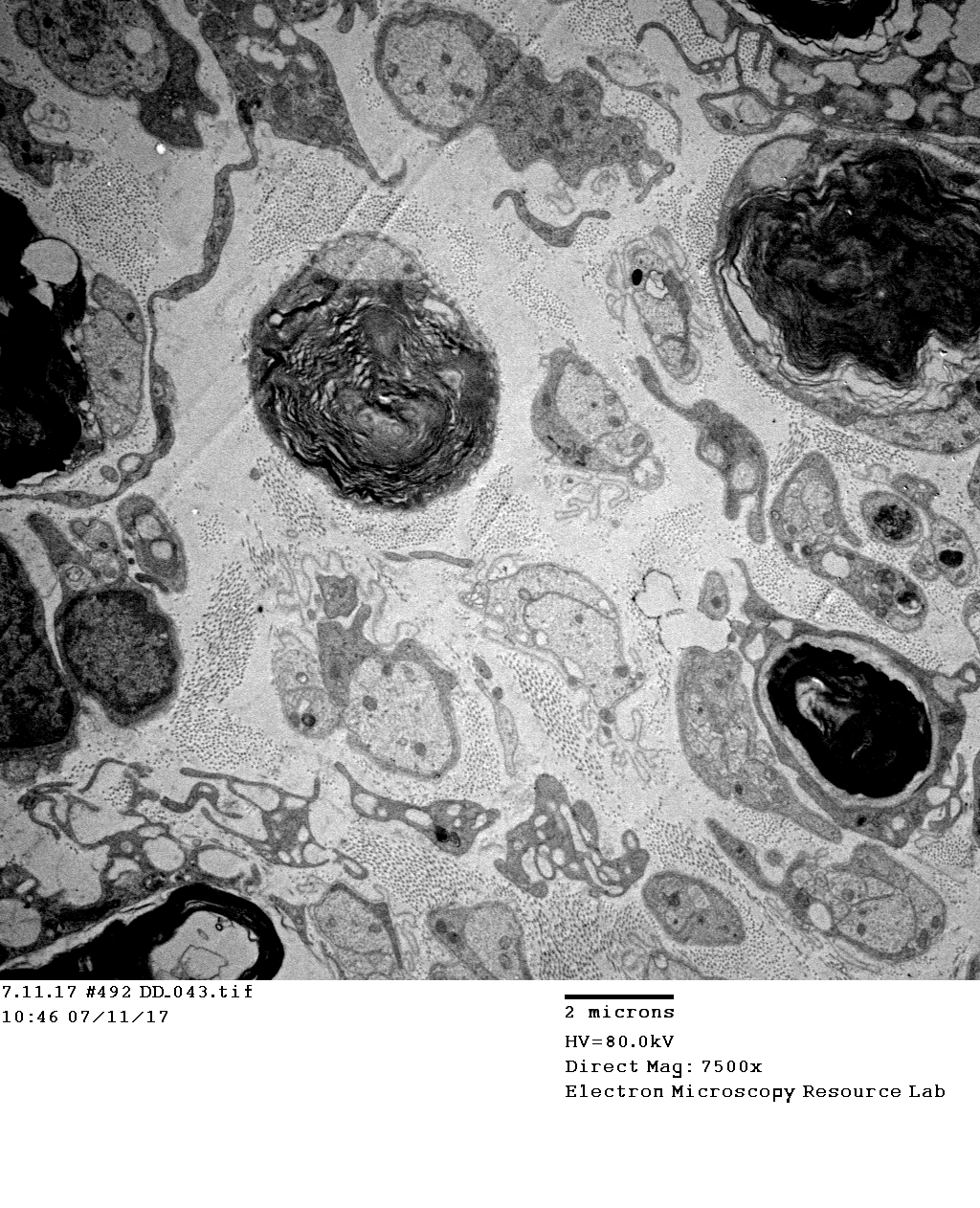

Supplement: Figure 5—source data 1. — This zip archive contains the TEM images for one WT and one iDKO used for quantitative analysis shown in Figure 5G–I. Images were taken using a JEOL 1010 electron microscope fitted with a Hamamatsu digital camera and AMT Advantage image capture software. Contrast of the images was adjusted using Photoshop software. The images in this archive were also used for the analysis in Figure 7. [file elife-50138-fig5-data1.zip › Figure 5 source data 1/iDKO #492 12d DD 7500X/7.11.17 #492 DD_043 Contrast .tif]

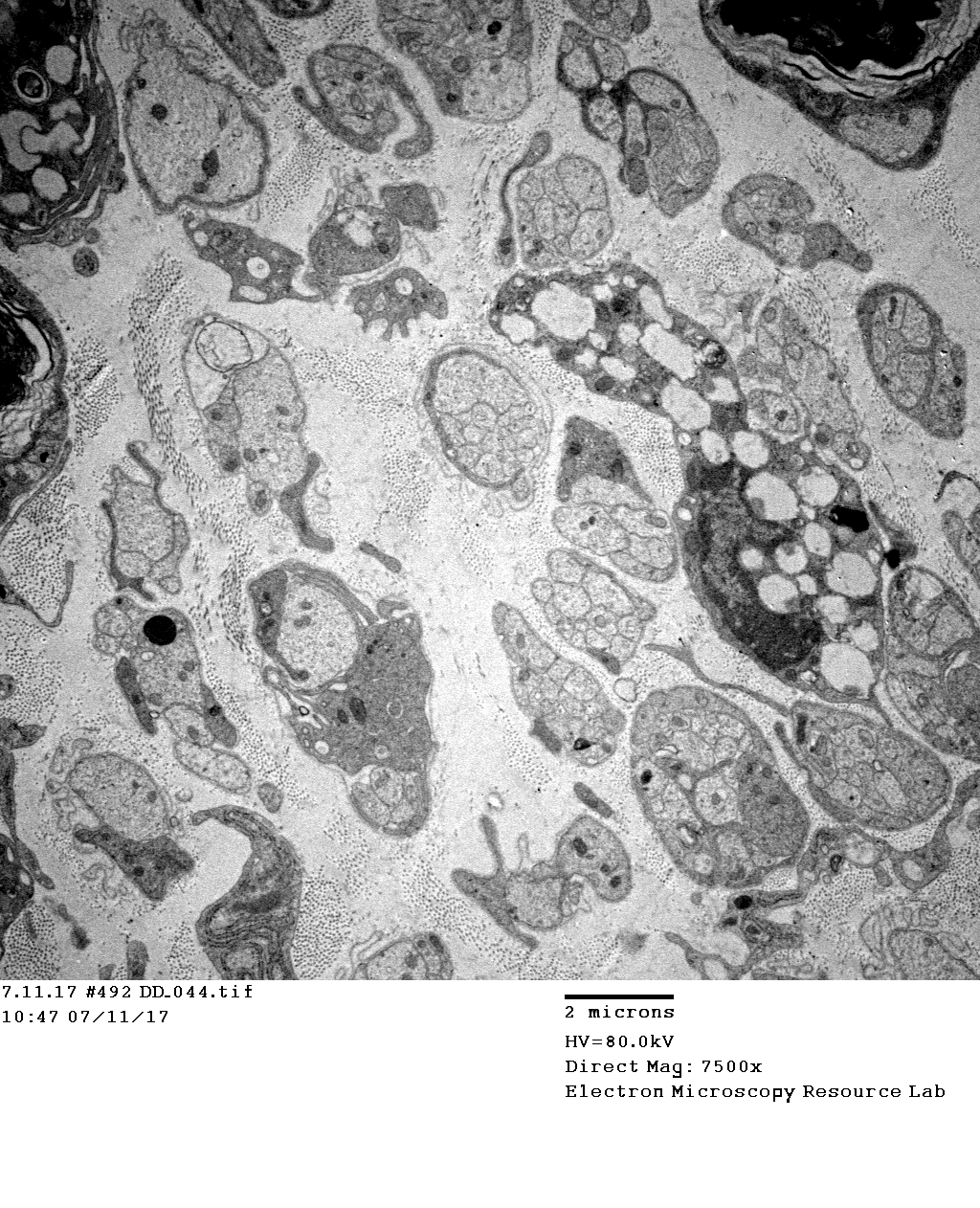

Supplement: Figure 5—source data 1. — This zip archive contains the TEM images for one WT and one iDKO used for quantitative analysis shown in Figure 5G–I. Images were taken using a JEOL 1010 electron microscope fitted with a Hamamatsu digital camera and AMT Advantage image capture software. Contrast of the images was adjusted using Photoshop software. The images in this archive were also used for the analysis in Figure 7. [file elife-50138-fig5-data1.zip › Figure 5 source data 1/iDKO #492 12d DD 7500X/7.11.17 #492 DD_044 Contrast .tif]

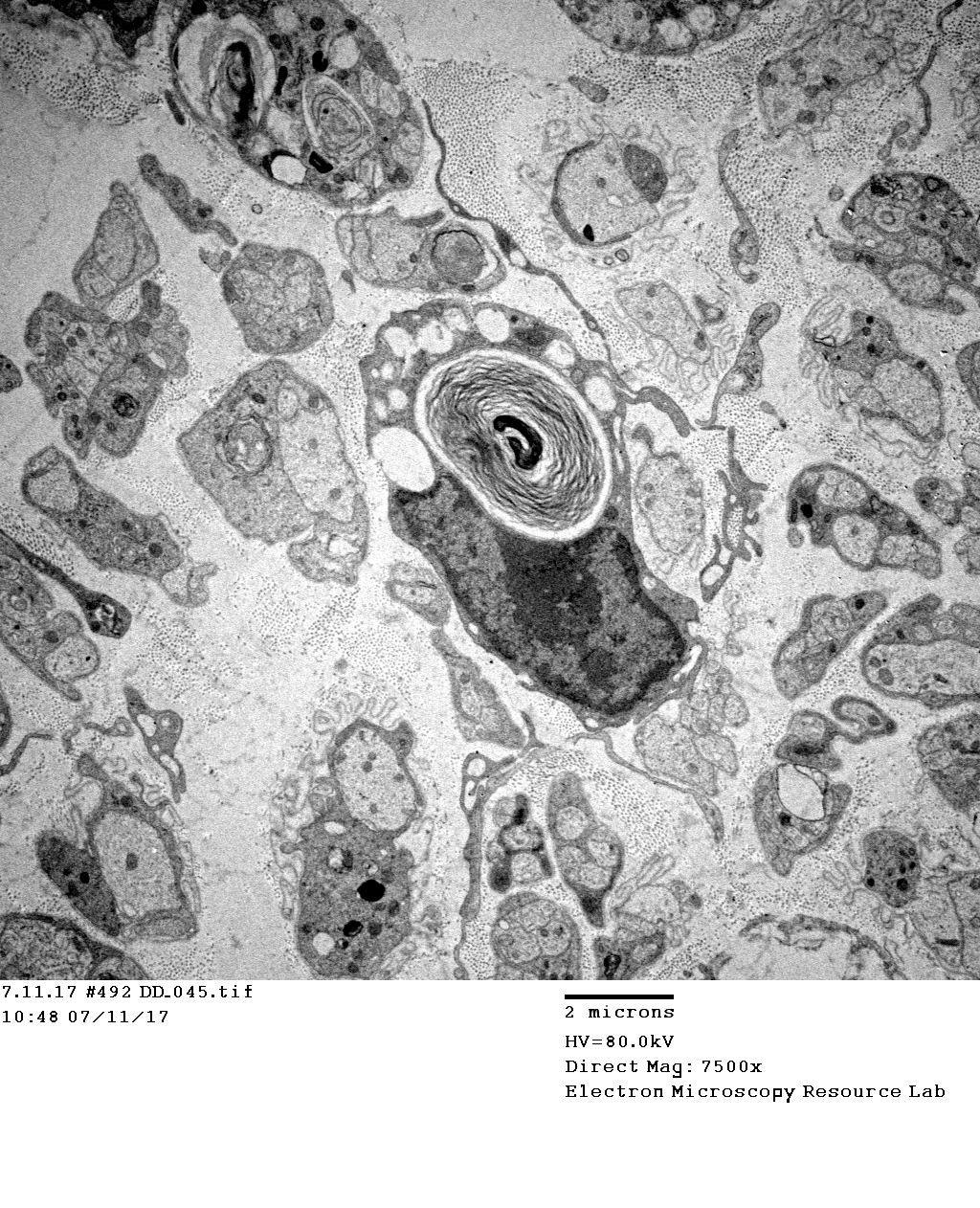

Supplement: Figure 5—source data 1. — This zip archive contains the TEM images for one WT and one iDKO used for quantitative analysis shown in Figure 5G–I. Images were taken using a JEOL 1010 electron microscope fitted with a Hamamatsu digital camera and AMT Advantage image capture software. Contrast of the images was adjusted using Photoshop software. The images in this archive were also used for the analysis in Figure 7. [file elife-50138-fig5-data1.zip › Figure 5 source data 1/iDKO #492 12d DD 7500X/7.11.17 #492 DD_045 Contrast .tif]

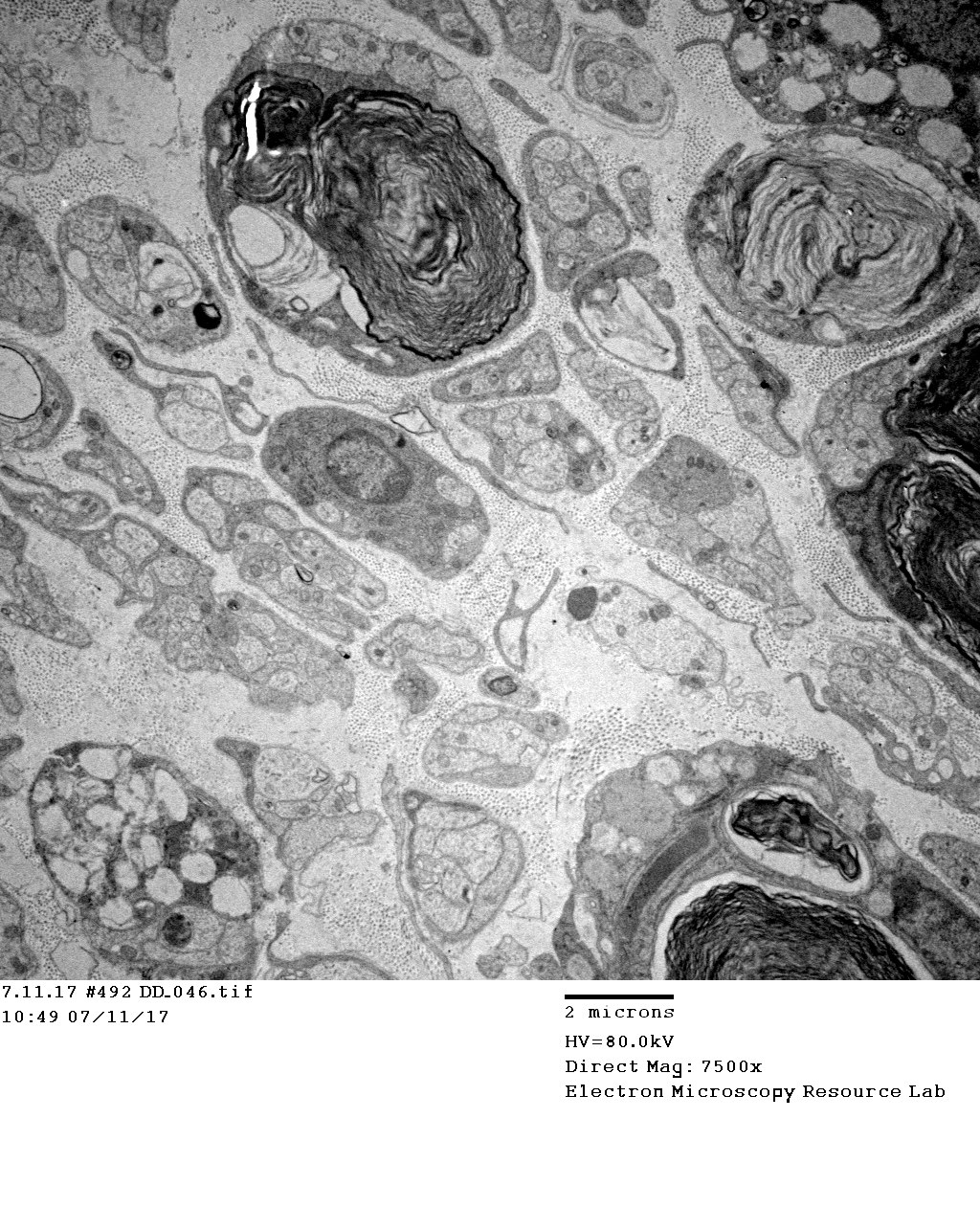

Supplement: Figure 5—source data 1. — This zip archive contains the TEM images for one WT and one iDKO used for quantitative analysis shown in Figure 5G–I. Images were taken using a JEOL 1010 electron microscope fitted with a Hamamatsu digital camera and AMT Advantage image capture software. Contrast of the images was adjusted using Photoshop software. The images in this archive were also used for the analysis in Figure 7. [file elife-50138-fig5-data1.zip › Figure 5 source data 1/iDKO #492 12d DD 7500X/7.11.17 #492 DD_046 Contrast .tif]

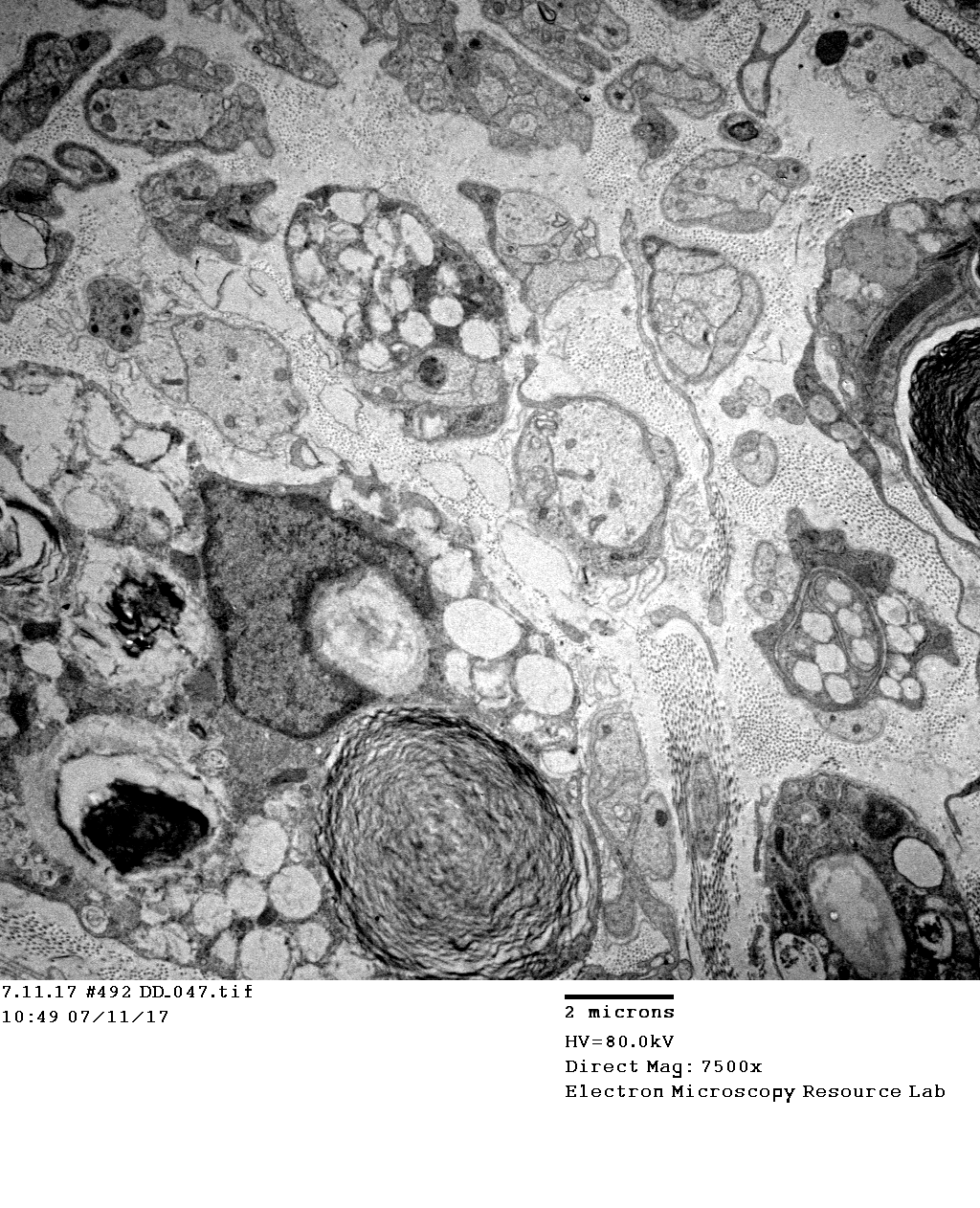

Supplement: Figure 5—source data 1. — This zip archive contains the TEM images for one WT and one iDKO used for quantitative analysis shown in Figure 5G–I. Images were taken using a JEOL 1010 electron microscope fitted with a Hamamatsu digital camera and AMT Advantage image capture software. Contrast of the images was adjusted using Photoshop software. The images in this archive were also used for the analysis in Figure 7. [file elife-50138-fig5-data1.zip › Figure 5 source data 1/iDKO #492 12d DD 7500X/7.11.17 #492 DD_047 Contrast .tif]

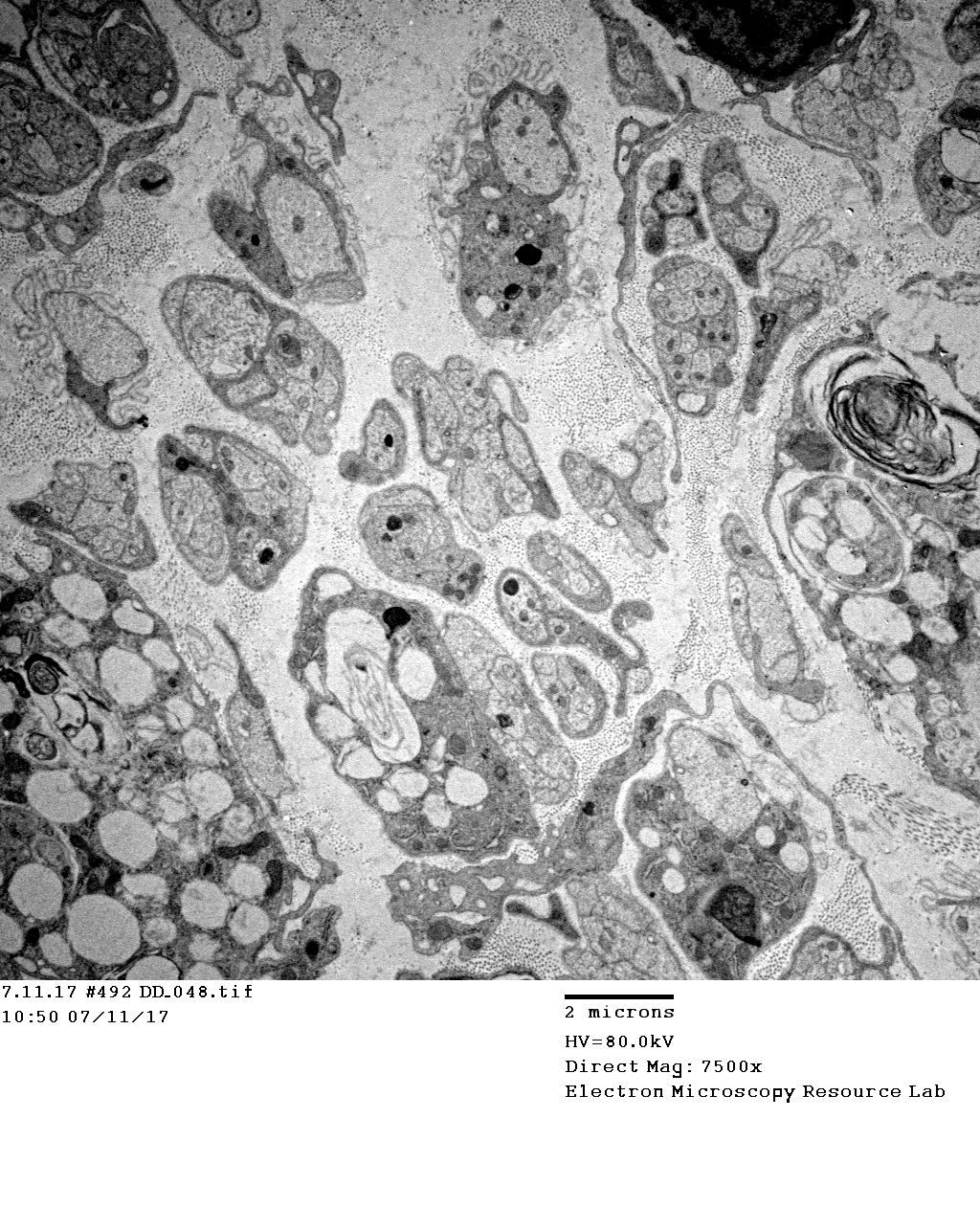

Supplement: Figure 5—source data 1. — This zip archive contains the TEM images for one WT and one iDKO used for quantitative analysis shown in Figure 5G–I. Images were taken using a JEOL 1010 electron microscope fitted with a Hamamatsu digital camera and AMT Advantage image capture software. Contrast of the images was adjusted using Photoshop software. The images in this archive were also used for the analysis in Figure 7. [file elife-50138-fig5-data1.zip › Figure 5 source data 1/iDKO #492 12d DD 7500X/7.11.17 #492 DD_048 Contrast .tif]

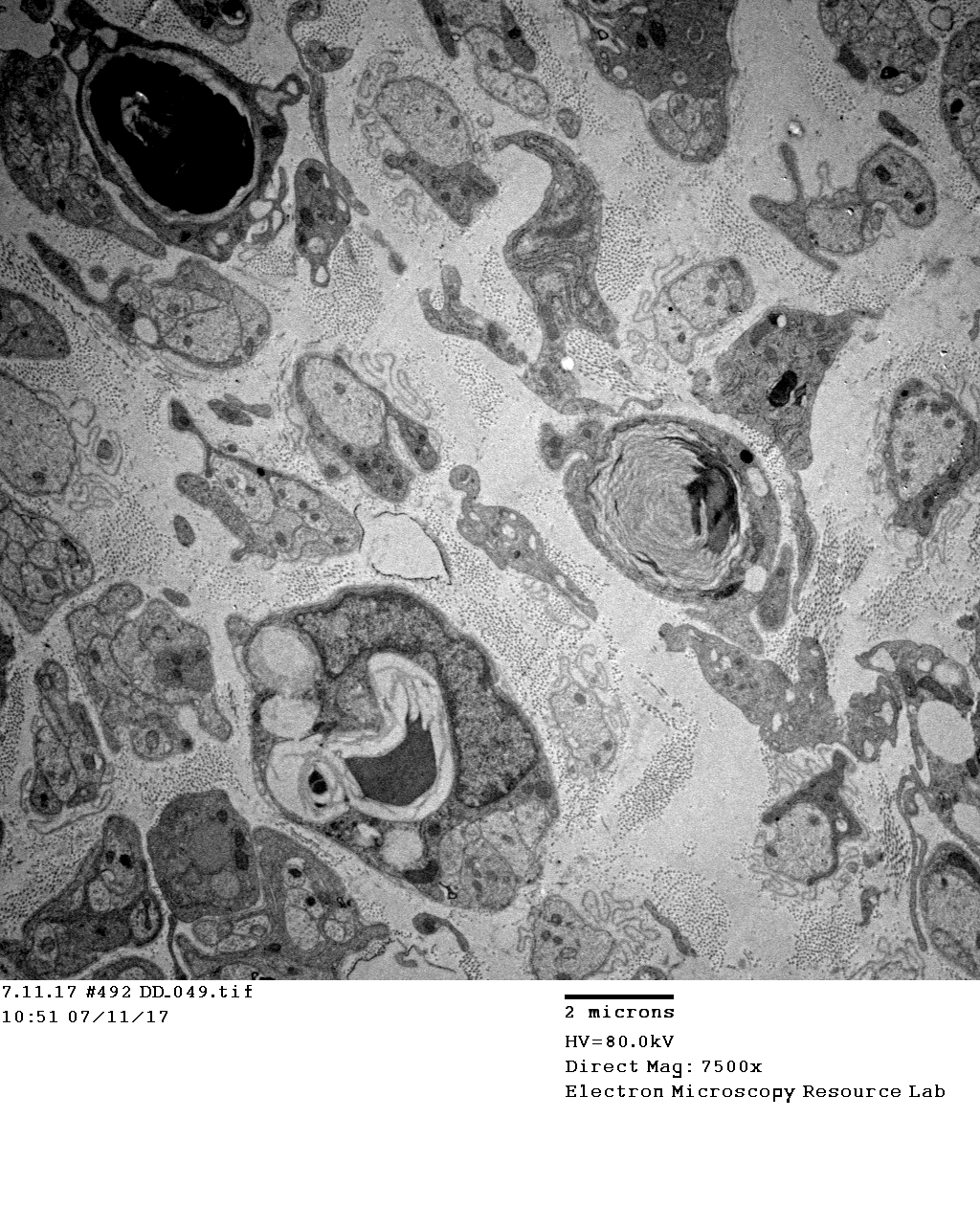

Supplement: Figure 5—source data 1. — This zip archive contains the TEM images for one WT and one iDKO used for quantitative analysis shown in Figure 5G–I. Images were taken using a JEOL 1010 electron microscope fitted with a Hamamatsu digital camera and AMT Advantage image capture software. Contrast of the images was adjusted using Photoshop software. The images in this archive were also used for the analysis in Figure 7. [file elife-50138-fig5-data1.zip › Figure 5 source data 1/iDKO #492 12d DD 7500X/7.11.17 #492 DD_049 Contrast .tif]

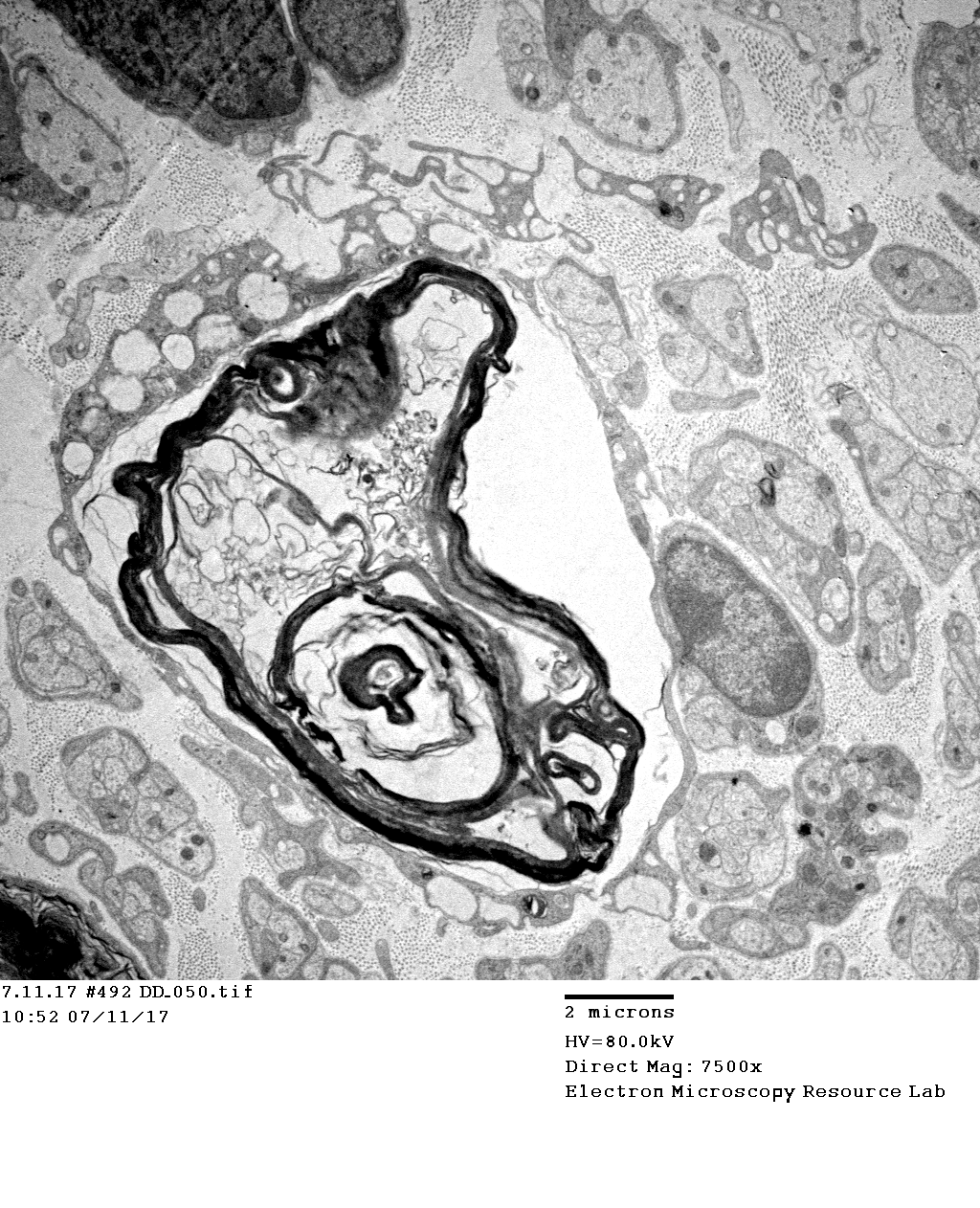

Supplement: Figure 5—source data 1. — This zip archive contains the TEM images for one WT and one iDKO used for quantitative analysis shown in Figure 5G–I. Images were taken using a JEOL 1010 electron microscope fitted with a Hamamatsu digital camera and AMT Advantage image capture software. Contrast of the images was adjusted using Photoshop software. The images in this archive were also used for the analysis in Figure 7. [file elife-50138-fig5-data1.zip › Figure 5 source data 1/iDKO #492 12d DD 7500X/7.11.17 #492 DD_050 Contrast .tif]

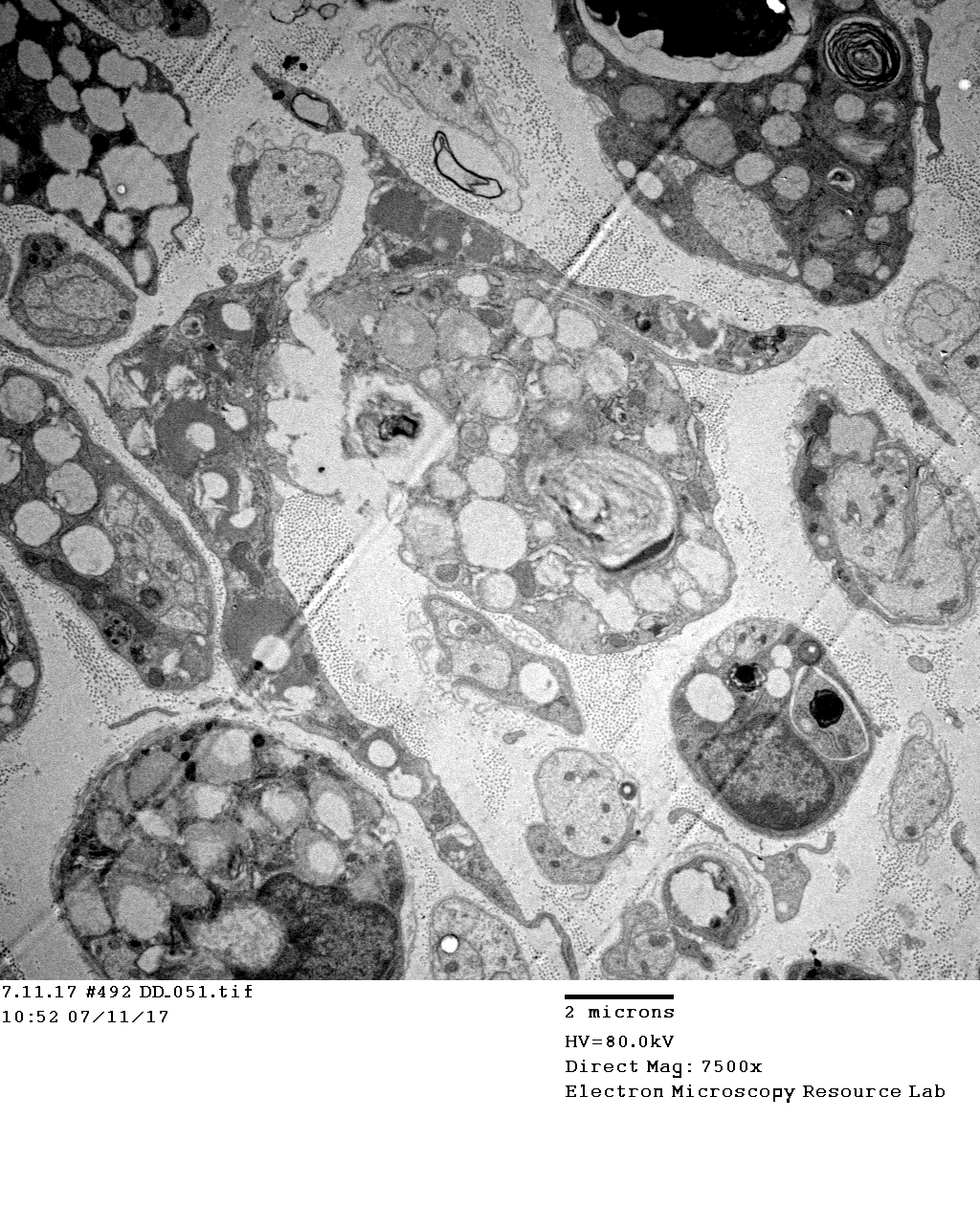

Supplement: Figure 5—source data 1. — This zip archive contains the TEM images for one WT and one iDKO used for quantitative analysis shown in Figure 5G–I. Images were taken using a JEOL 1010 electron microscope fitted with a Hamamatsu digital camera and AMT Advantage image capture software. Contrast of the images was adjusted using Photoshop software. The images in this archive were also used for the analysis in Figure 7. [file elife-50138-fig5-data1.zip › Figure 5 source data 1/iDKO #492 12d DD 7500X/7.11.17 #492 DD_051 Contrast .tif]

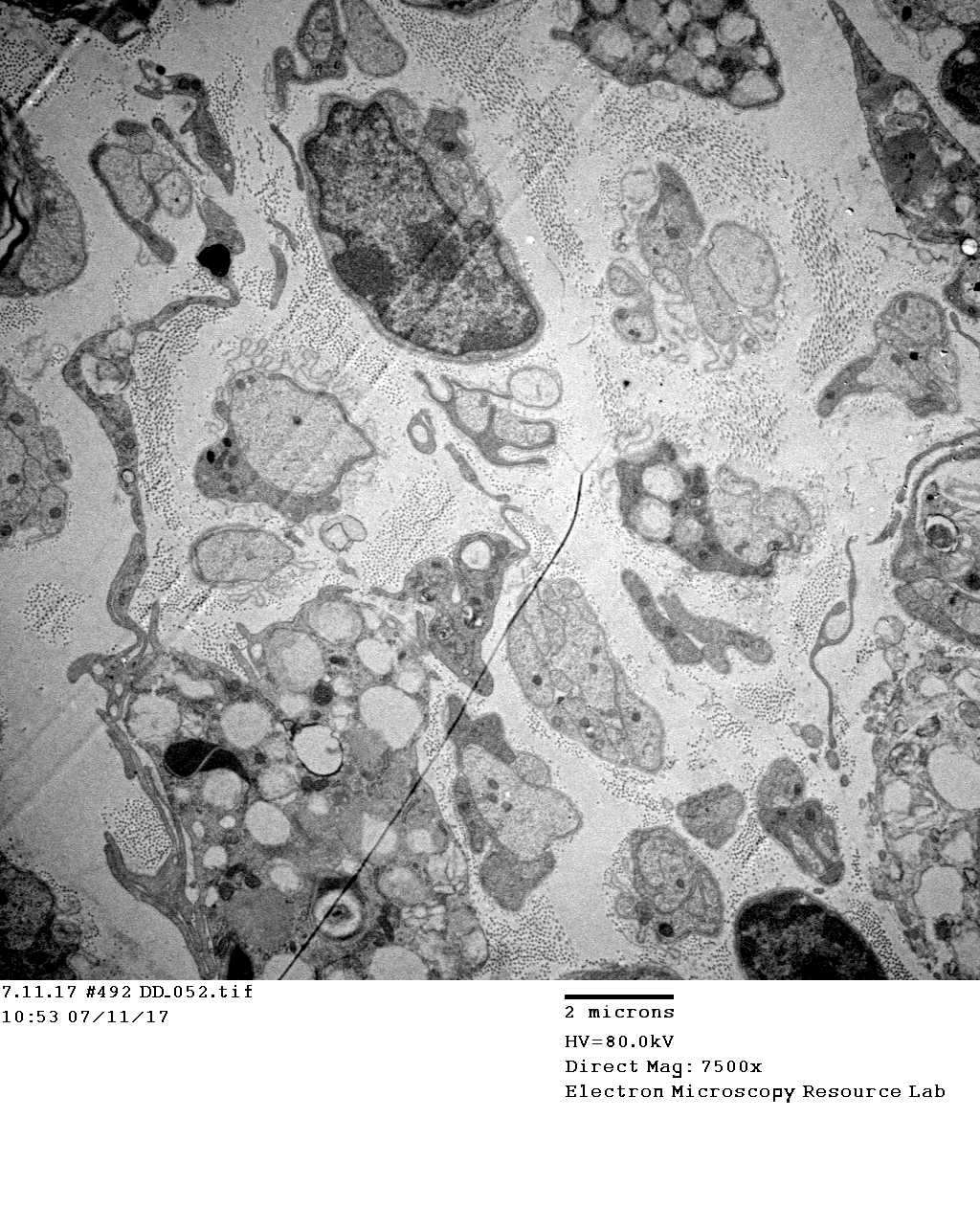

Supplement: Figure 5—source data 1. — This zip archive contains the TEM images for one WT and one iDKO used for quantitative analysis shown in Figure 5G–I. Images were taken using a JEOL 1010 electron microscope fitted with a Hamamatsu digital camera and AMT Advantage image capture software. Contrast of the images was adjusted using Photoshop software. The images in this archive were also used for the analysis in Figure 7. [file elife-50138-fig5-data1.zip › Figure 5 source data 1/iDKO #492 12d DD 7500X/7.11.17 #492 DD_052 Contrast .tif]

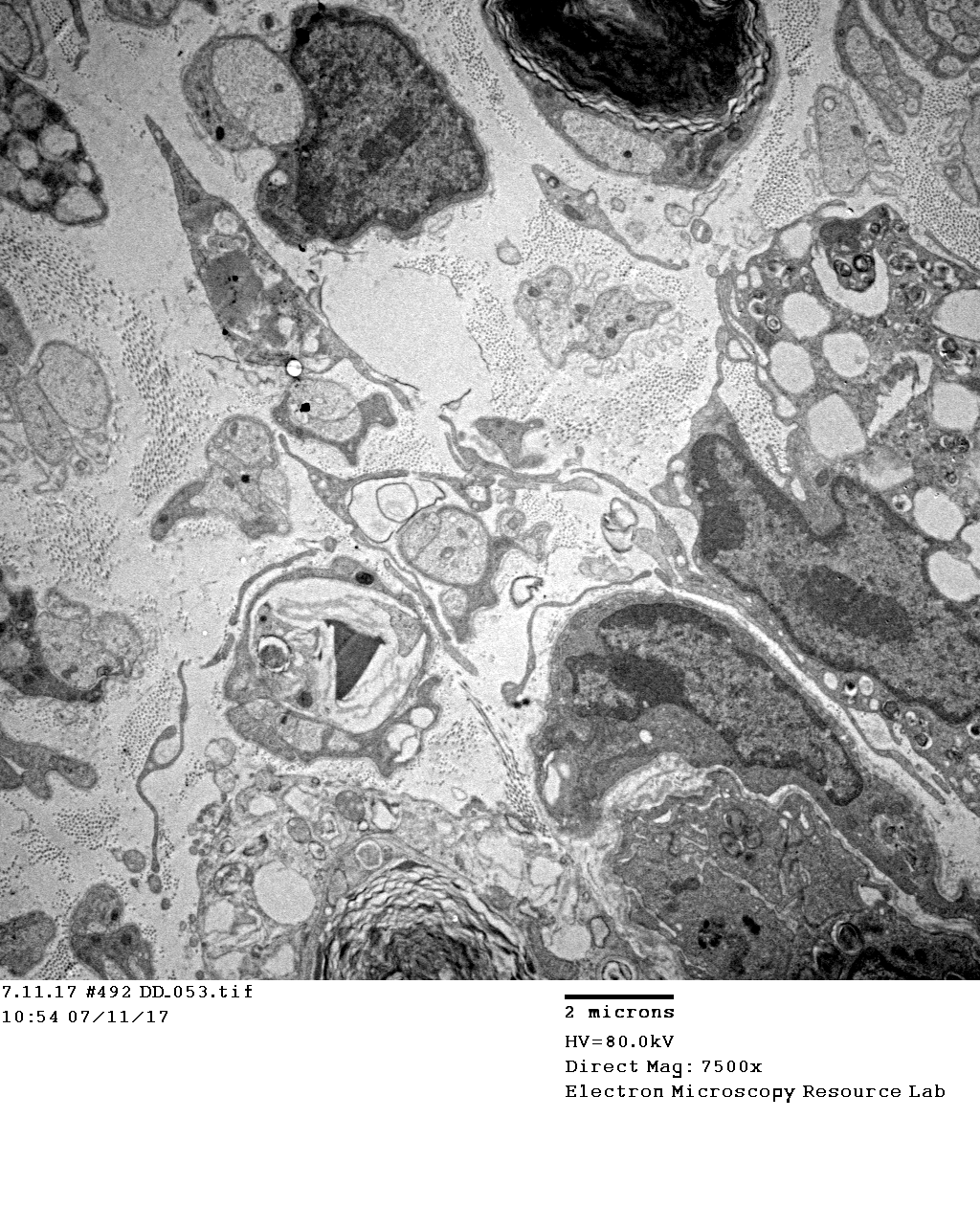

Supplement: Figure 5—source data 1. — This zip archive contains the TEM images for one WT and one iDKO used for quantitative analysis shown in Figure 5G–I. Images were taken using a JEOL 1010 electron microscope fitted with a Hamamatsu digital camera and AMT Advantage image capture software. Contrast of the images was adjusted using Photoshop software. The images in this archive were also used for the analysis in Figure 7. [file elife-50138-fig5-data1.zip › Figure 5 source data 1/iDKO #492 12d DD 7500X/7.11.17 #492 DD_053 Contrast .tif]

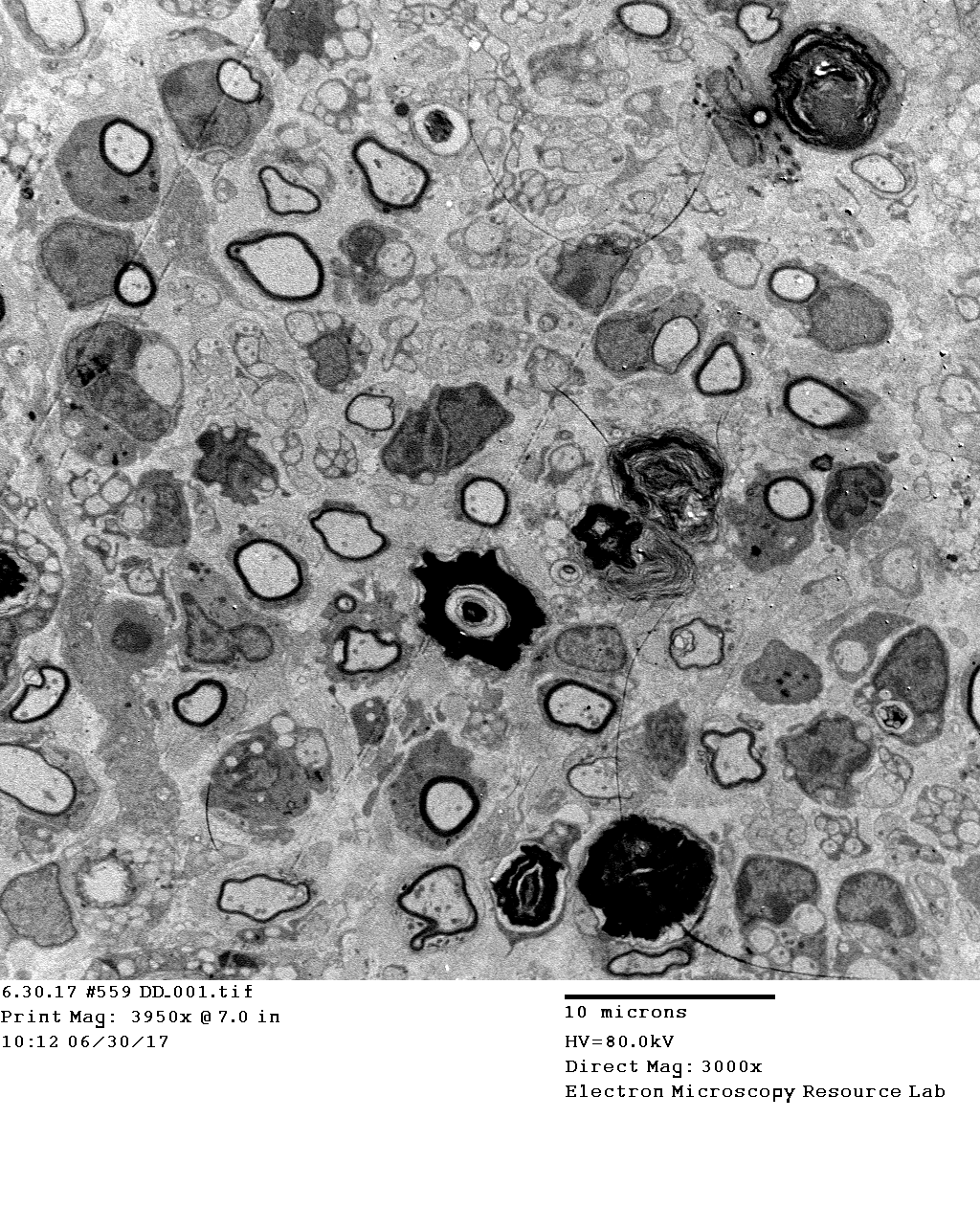

Supplement: Figure 5—source data 1. — This zip archive contains the TEM images for one WT and one iDKO used for quantitative analysis shown in Figure 5G–I. Images were taken using a JEOL 1010 electron microscope fitted with a Hamamatsu digital camera and AMT Advantage image capture software. Contrast of the images was adjusted using Photoshop software. The images in this archive were also used for the analysis in Figure 7. [file elife-50138-fig5-data1.zip › Figure 5 source data 1/WT #559 12d DD 7500X/6.30.17 #559 DD_001 contrast .tif]

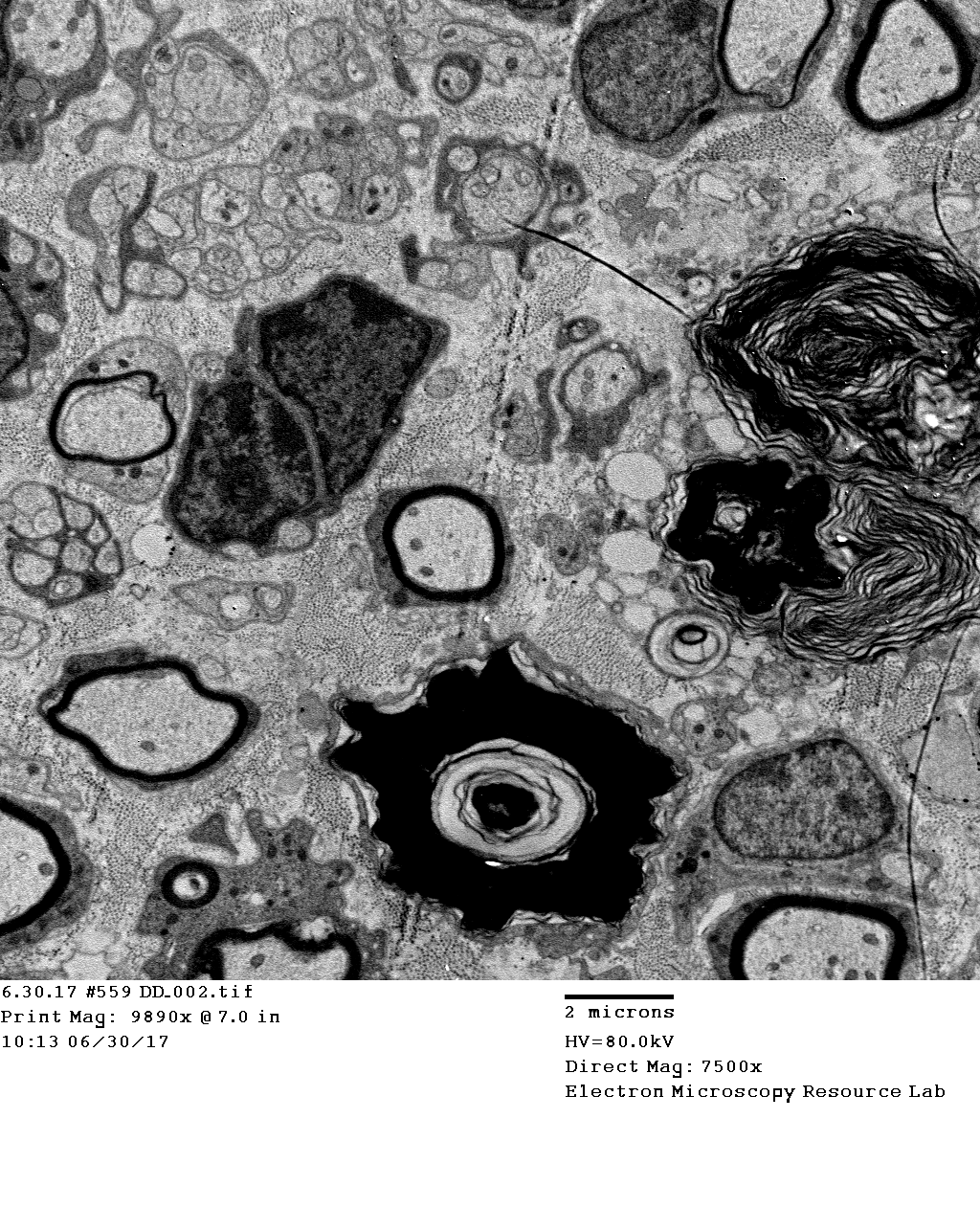

Supplement: Figure 5—source data 1. — This zip archive contains the TEM images for one WT and one iDKO used for quantitative analysis shown in Figure 5G–I. Images were taken using a JEOL 1010 electron microscope fitted with a Hamamatsu digital camera and AMT Advantage image capture software. Contrast of the images was adjusted using Photoshop software. The images in this archive were also used for the analysis in Figure 7. [file elife-50138-fig5-data1.zip › Figure 5 source data 1/WT #559 12d DD 7500X/6.30.17 #559 DD_002 contrast .tif]

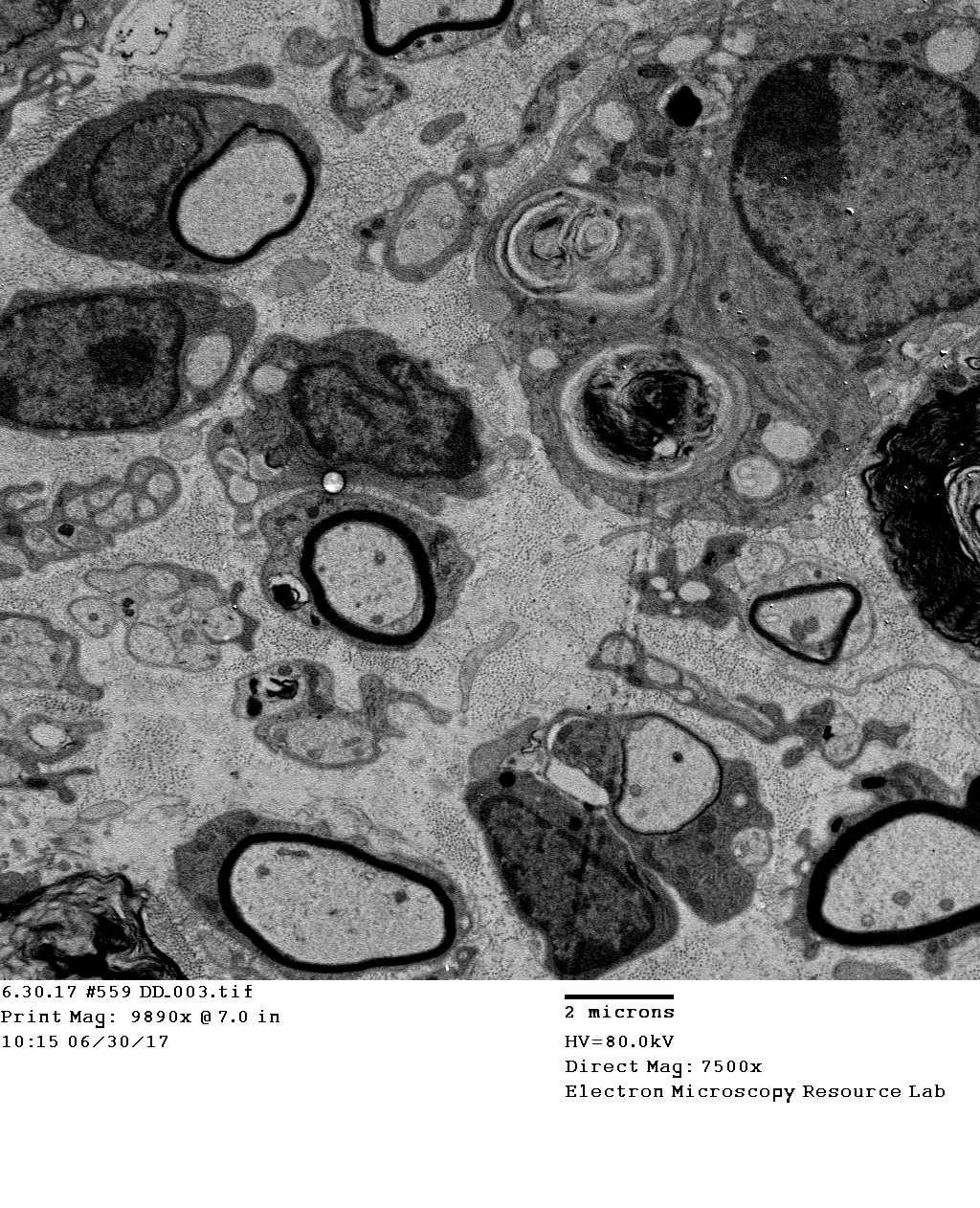

Supplement: Figure 5—source data 1. — This zip archive contains the TEM images for one WT and one iDKO used for quantitative analysis shown in Figure 5G–I. Images were taken using a JEOL 1010 electron microscope fitted with a Hamamatsu digital camera and AMT Advantage image capture software. Contrast of the images was adjusted using Photoshop software. The images in this archive were also used for the analysis in Figure 7. [file elife-50138-fig5-data1.zip › Figure 5 source data 1/WT #559 12d DD 7500X/6.30.17 #559 DD_003 contrast .tif]

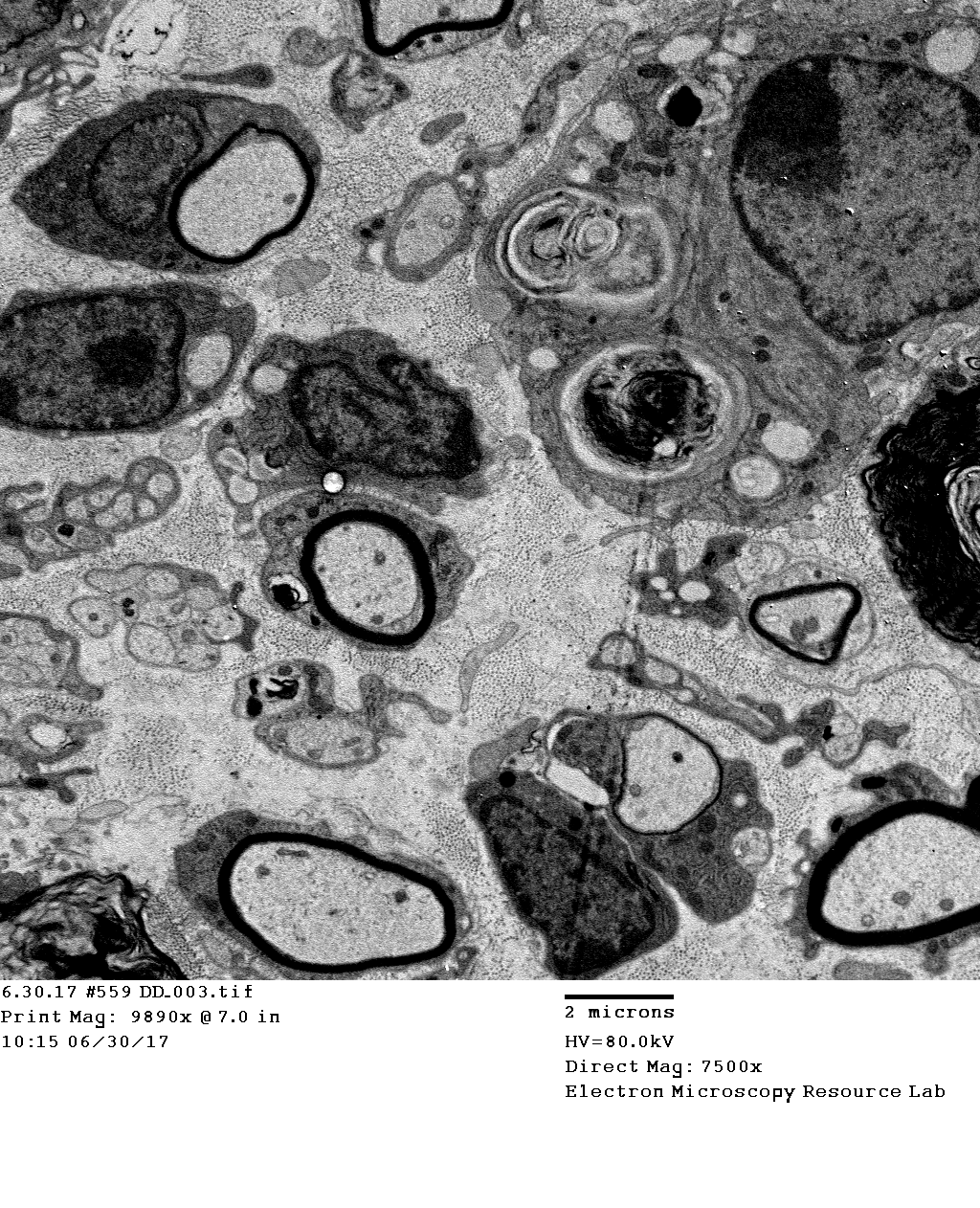

Supplement: Figure 5—source data 1. — This zip archive contains the TEM images for one WT and one iDKO used for quantitative analysis shown in Figure 5G–I. Images were taken using a JEOL 1010 electron microscope fitted with a Hamamatsu digital camera and AMT Advantage image capture software. Contrast of the images was adjusted using Photoshop software. The images in this archive were also used for the analysis in Figure 7. [file elife-50138-fig5-data1.zip › Figure 5 source data 1/WT #559 12d DD 7500X/6.30.17 #559 DD_003 contrast b .tif]

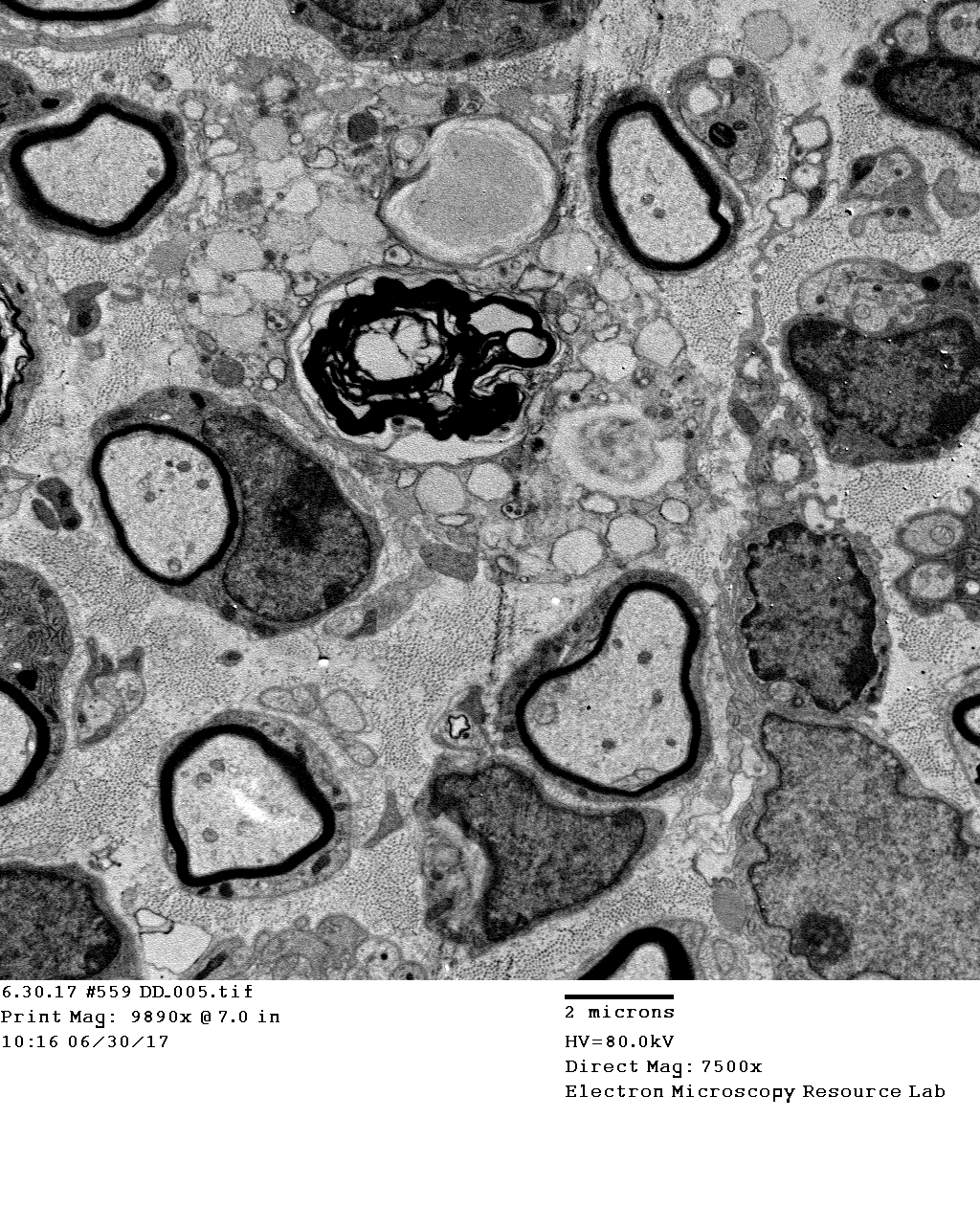

Supplement: Figure 5—source data 1. — This zip archive contains the TEM images for one WT and one iDKO used for quantitative analysis shown in Figure 5G–I. Images were taken using a JEOL 1010 electron microscope fitted with a Hamamatsu digital camera and AMT Advantage image capture software. Contrast of the images was adjusted using Photoshop software. The images in this archive were also used for the analysis in Figure 7. [file elife-50138-fig5-data1.zip › Figure 5 source data 1/WT #559 12d DD 7500X/6.30.17 #559 DD_005 contrast.tif]

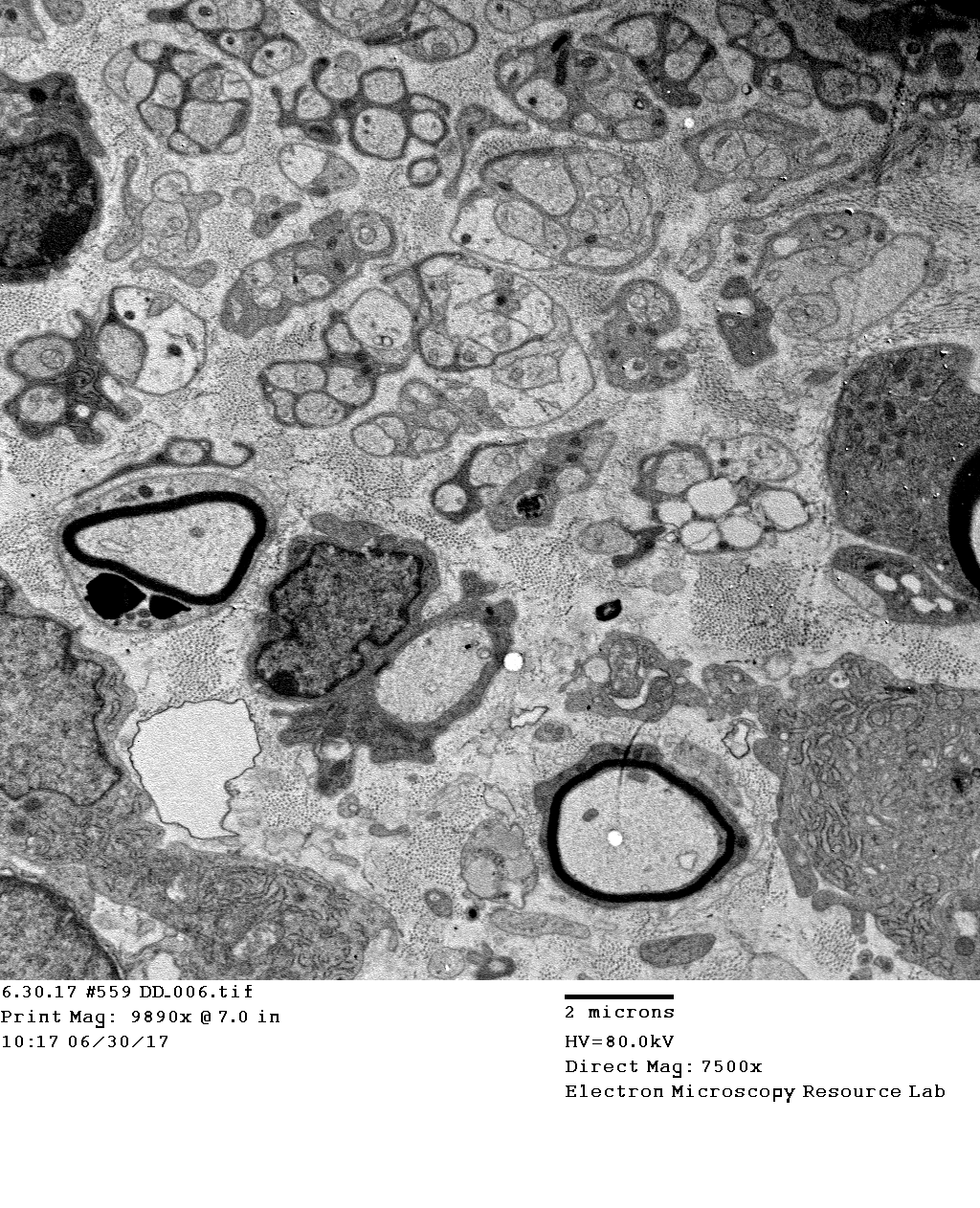

Supplement: Figure 5—source data 1. — This zip archive contains the TEM images for one WT and one iDKO used for quantitative analysis shown in Figure 5G–I. Images were taken using a JEOL 1010 electron microscope fitted with a Hamamatsu digital camera and AMT Advantage image capture software. Contrast of the images was adjusted using Photoshop software. The images in this archive were also used for the analysis in Figure 7. [file elife-50138-fig5-data1.zip › Figure 5 source data 1/WT #559 12d DD 7500X/6.30.17 #559 DD_006 contrast .tif]

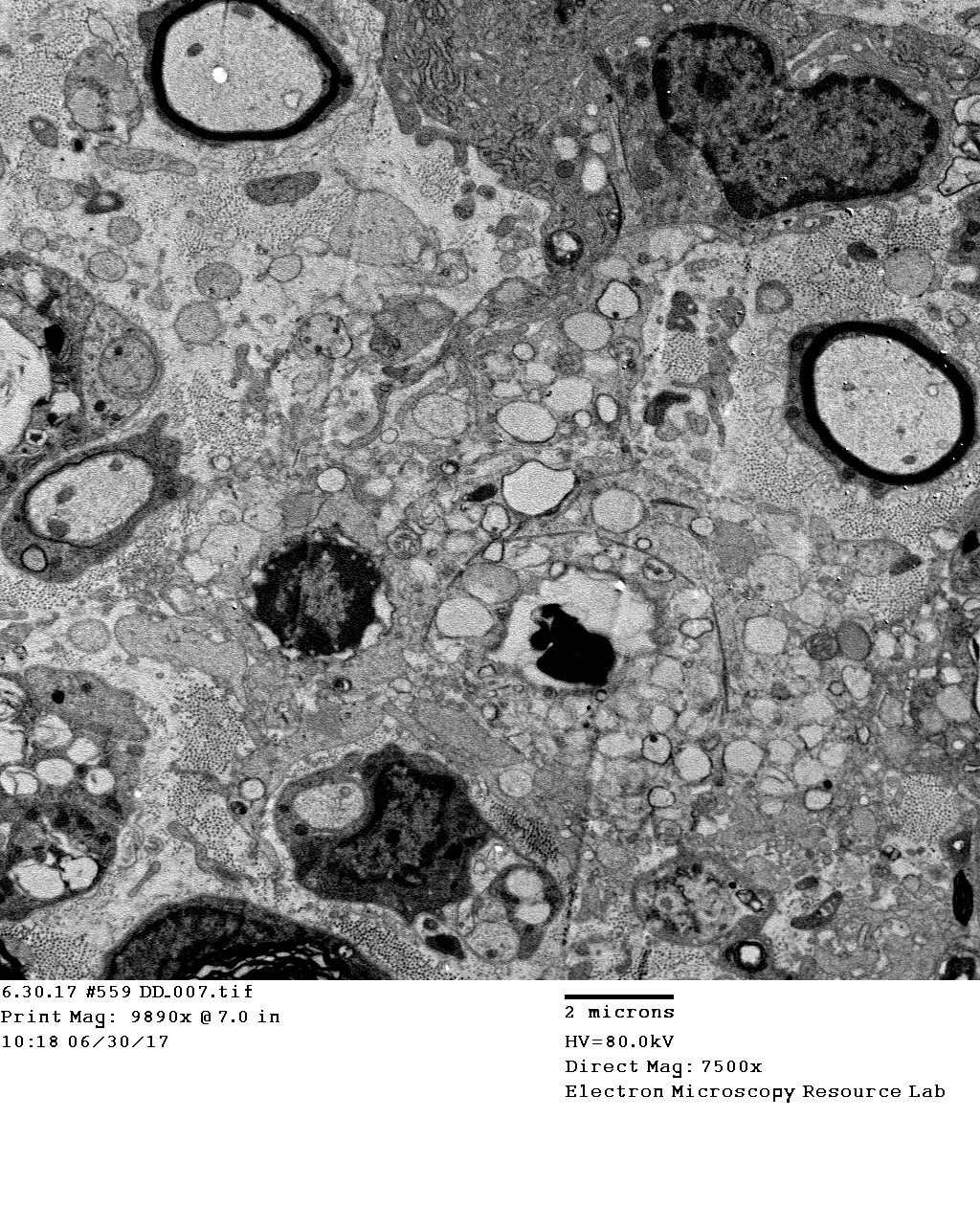

Supplement: Figure 5—source data 1. — This zip archive contains the TEM images for one WT and one iDKO used for quantitative analysis shown in Figure 5G–I. Images were taken using a JEOL 1010 electron microscope fitted with a Hamamatsu digital camera and AMT Advantage image capture software. Contrast of the images was adjusted using Photoshop software. The images in this archive were also used for the analysis in Figure 7. [file elife-50138-fig5-data1.zip › Figure 5 source data 1/WT #559 12d DD 7500X/6.30.17 #559 DD_007 contrast .tif]

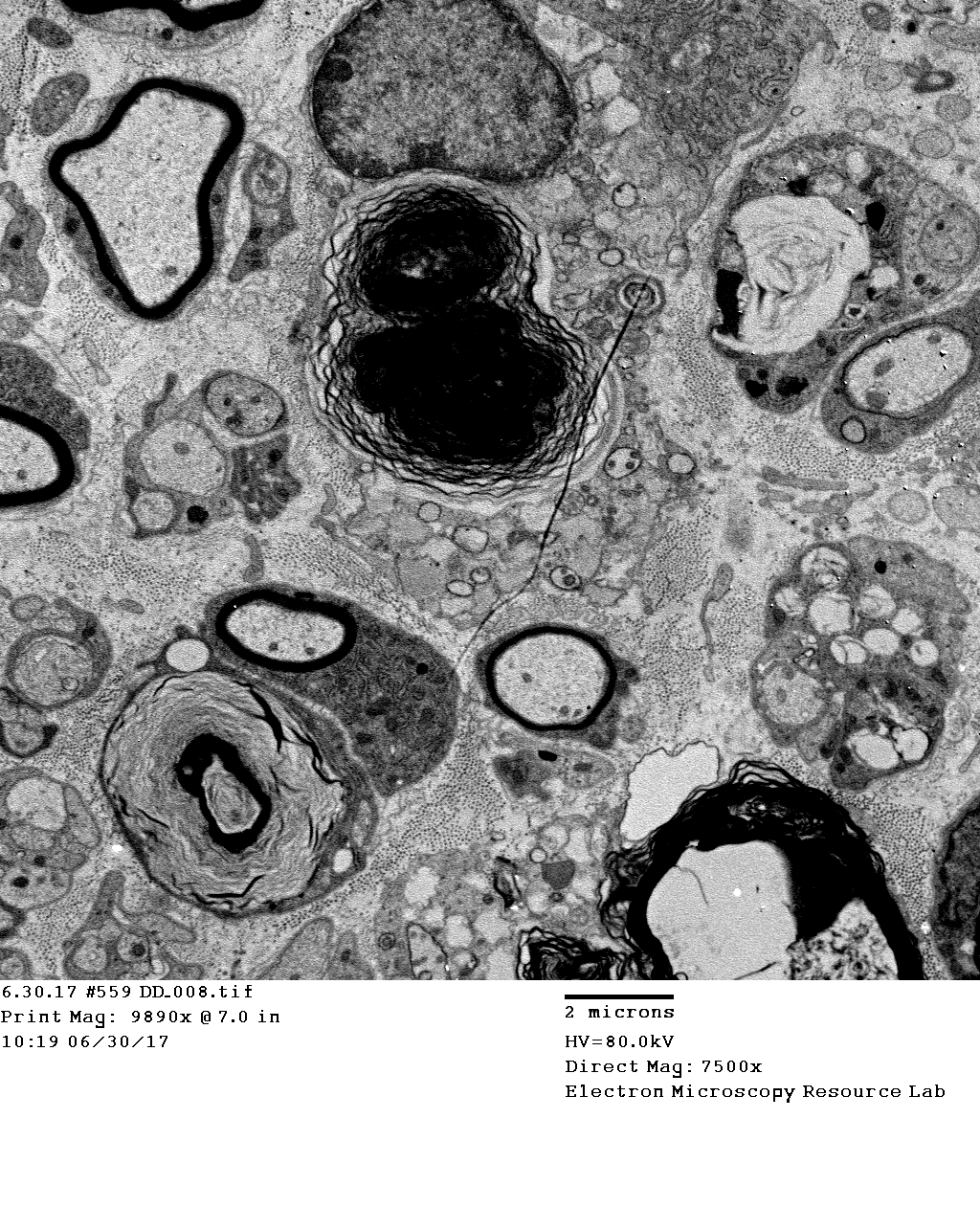

Supplement: Figure 5—source data 1. — This zip archive contains the TEM images for one WT and one iDKO used for quantitative analysis shown in Figure 5G–I. Images were taken using a JEOL 1010 electron microscope fitted with a Hamamatsu digital camera and AMT Advantage image capture software. Contrast of the images was adjusted using Photoshop software. The images in this archive were also used for the analysis in Figure 7. [file elife-50138-fig5-data1.zip › Figure 5 source data 1/WT #559 12d DD 7500X/6.30.17 #559 DD_008 contrast .tif]

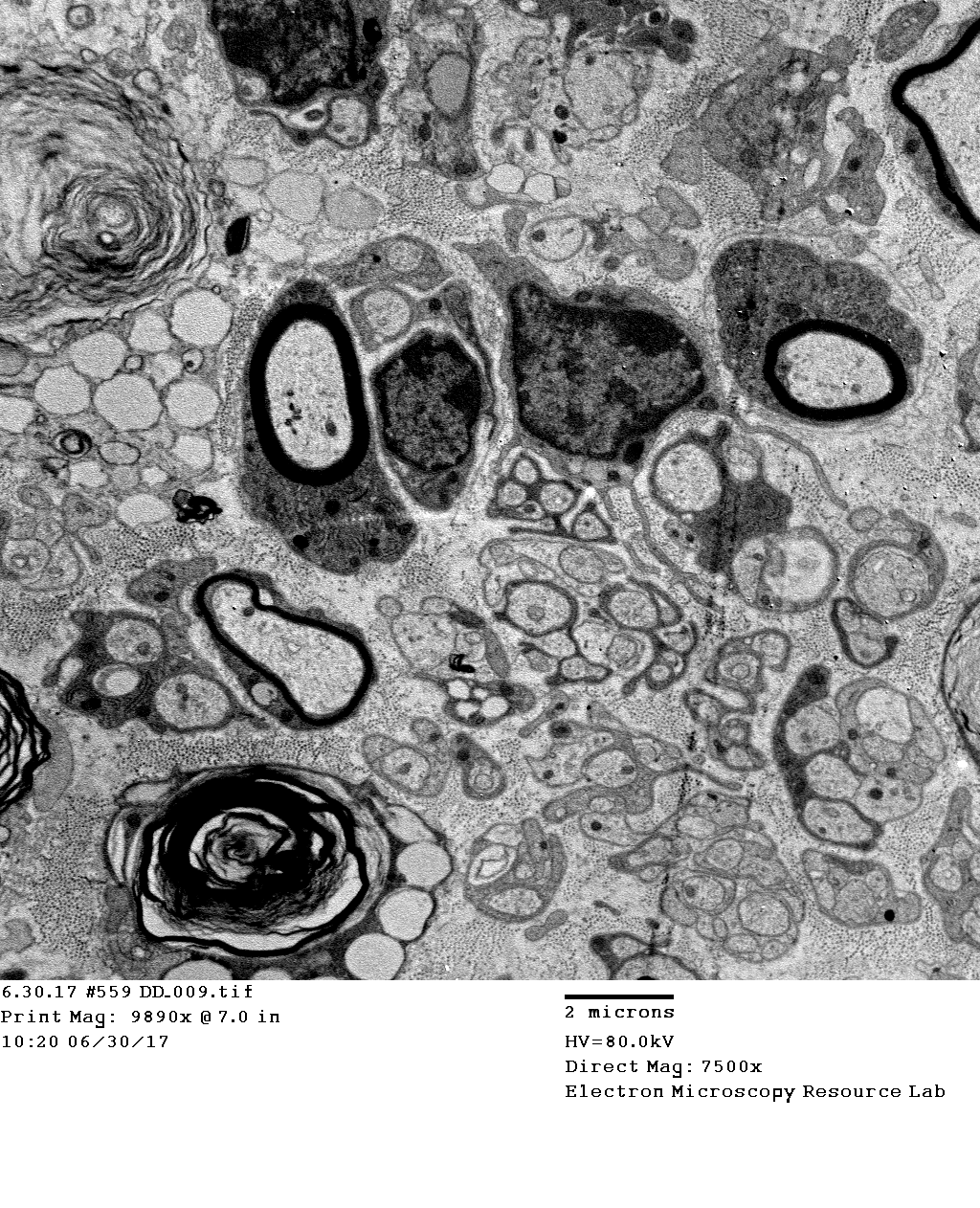

Supplement: Figure 5—source data 1. — This zip archive contains the TEM images for one WT and one iDKO used for quantitative analysis shown in Figure 5G–I. Images were taken using a JEOL 1010 electron microscope fitted with a Hamamatsu digital camera and AMT Advantage image capture software. Contrast of the images was adjusted using Photoshop software. The images in this archive were also used for the analysis in Figure 7. [file elife-50138-fig5-data1.zip › Figure 5 source data 1/WT #559 12d DD 7500X/6.30.17 #559 DD_009 contrast .tif]

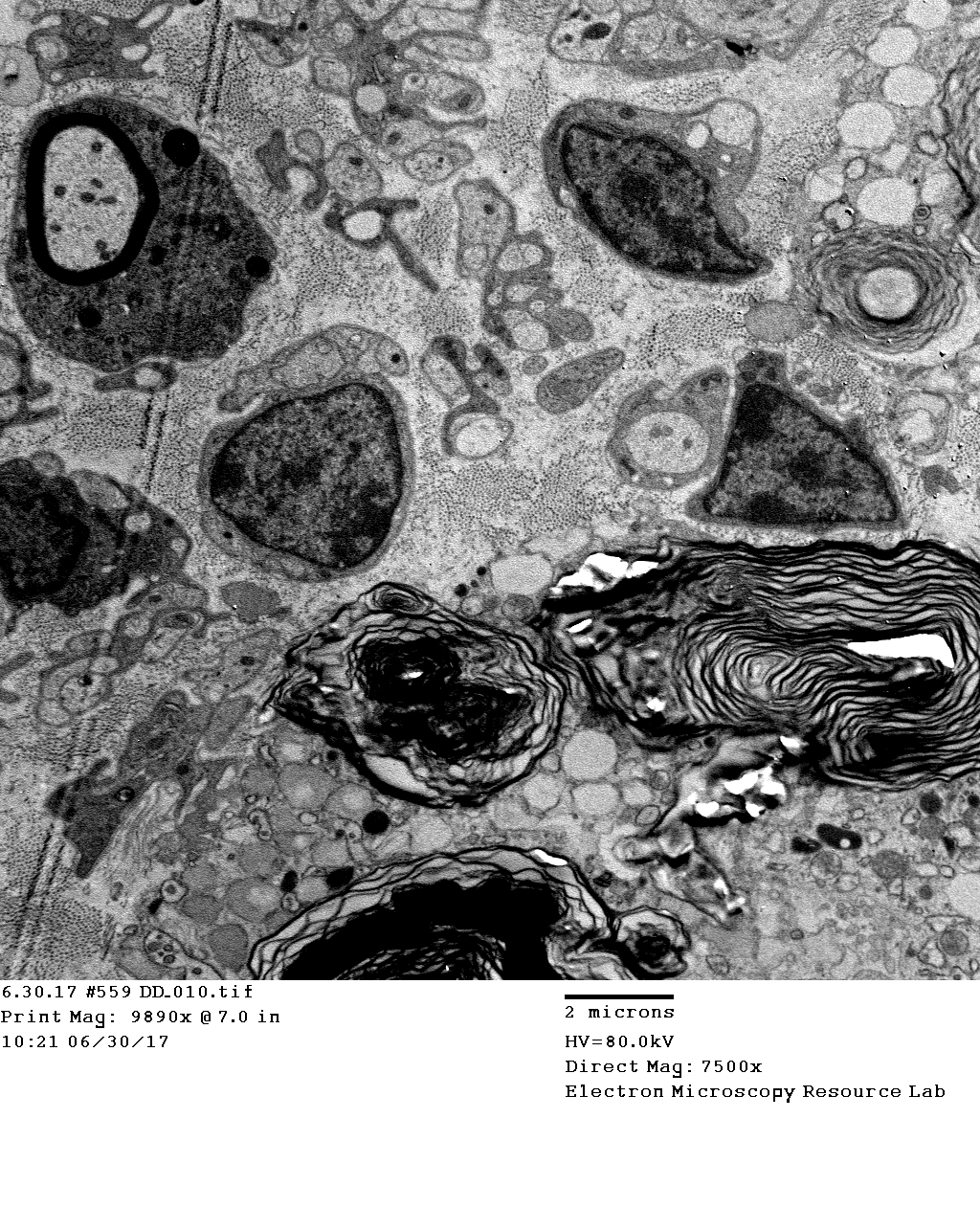

Supplement: Figure 5—source data 1. — This zip archive contains the TEM images for one WT and one iDKO used for quantitative analysis shown in Figure 5G–I. Images were taken using a JEOL 1010 electron microscope fitted with a Hamamatsu digital camera and AMT Advantage image capture software. Contrast of the images was adjusted using Photoshop software. The images in this archive were also used for the analysis in Figure 7. [file elife-50138-fig5-data1.zip › Figure 5 source data 1/WT #559 12d DD 7500X/6.30.17 #559 DD_010 contrast .tif]

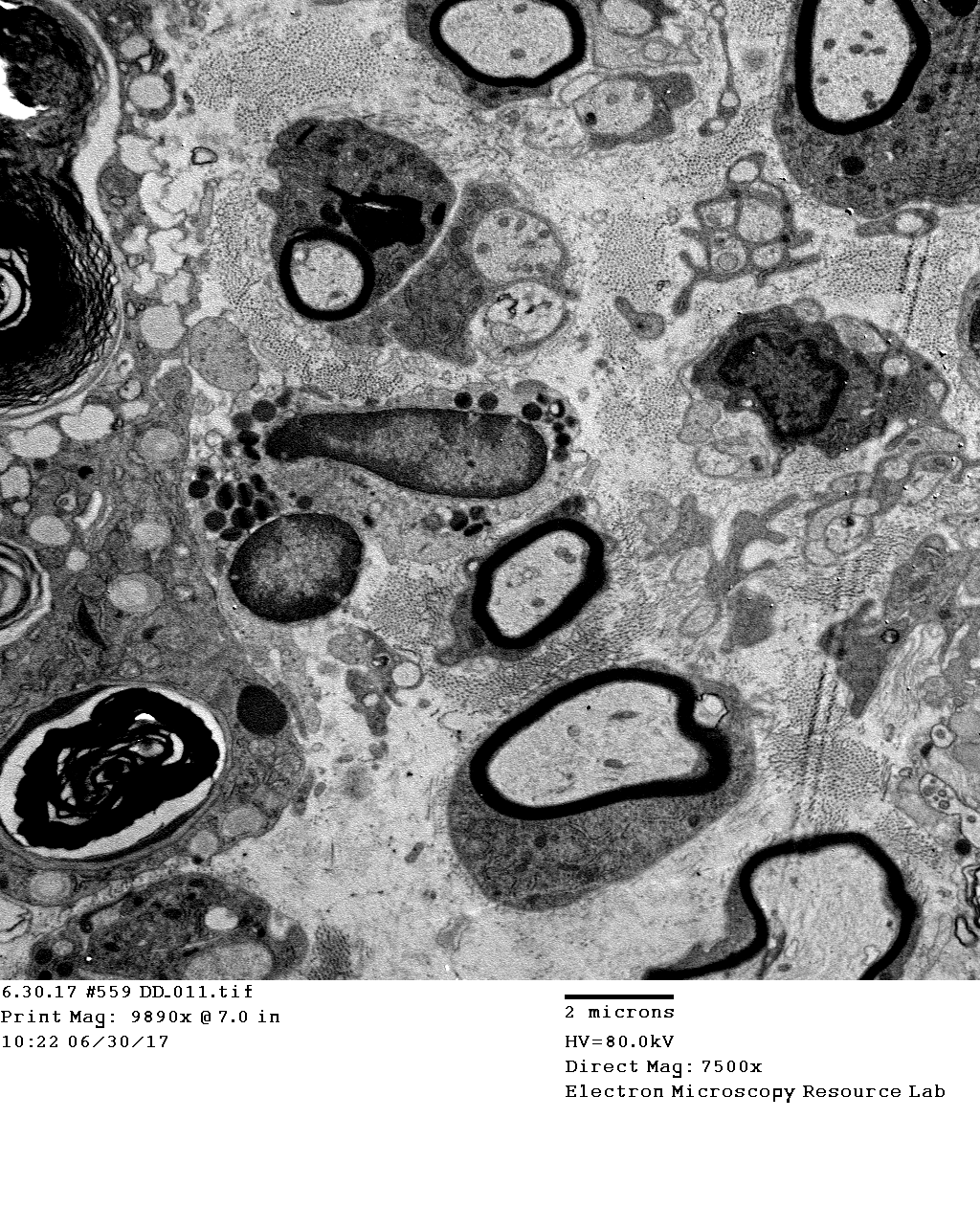

Supplement: Figure 5—source data 1. — This zip archive contains the TEM images for one WT and one iDKO used for quantitative analysis shown in Figure 5G–I. Images were taken using a JEOL 1010 electron microscope fitted with a Hamamatsu digital camera and AMT Advantage image capture software. Contrast of the images was adjusted using Photoshop software. The images in this archive were also used for the analysis in Figure 7. [file elife-50138-fig5-data1.zip › Figure 5 source data 1/WT #559 12d DD 7500X/6.30.17 #559 DD_011 contrast Y .tif]

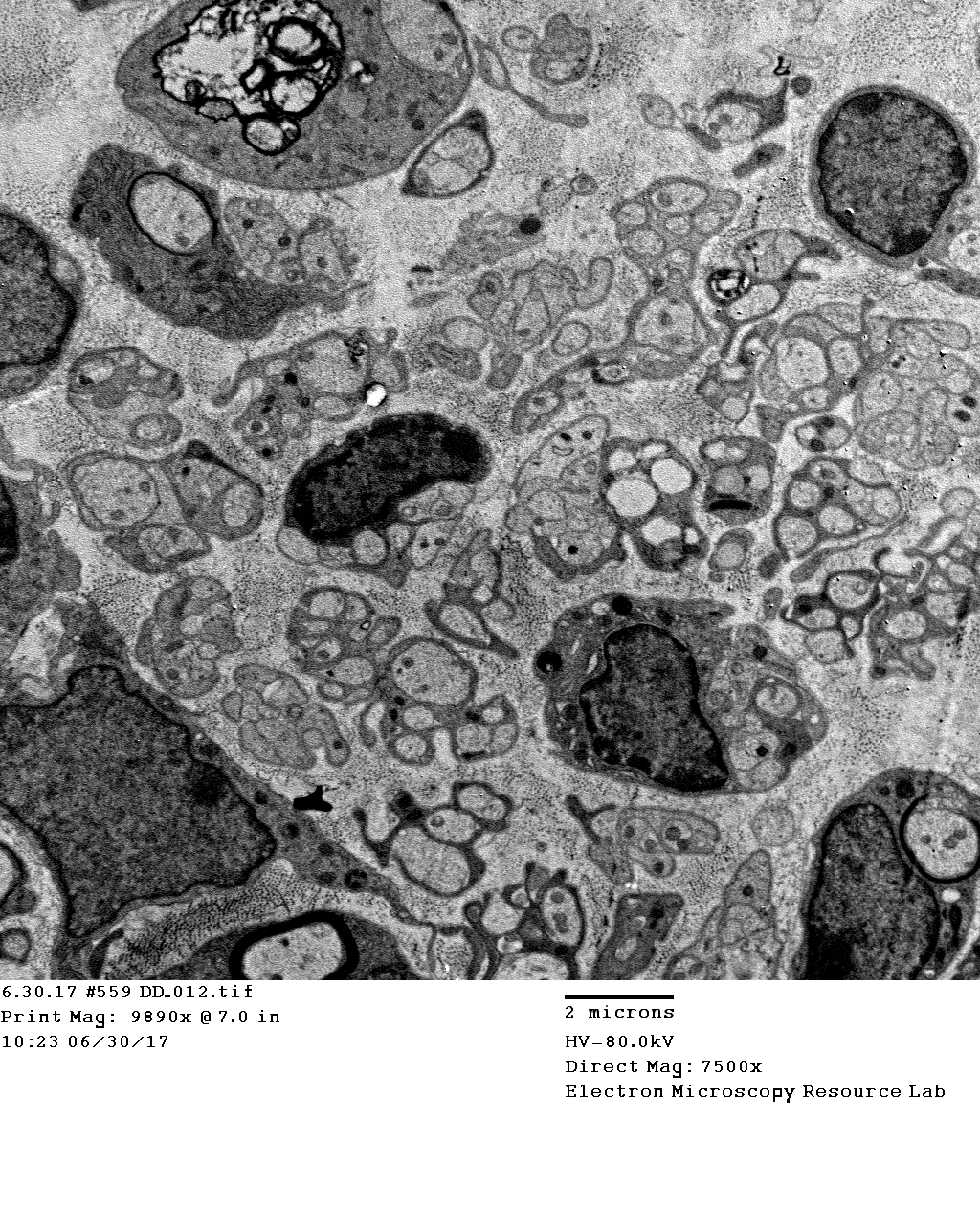

Supplement: Figure 5—source data 1. — This zip archive contains the TEM images for one WT and one iDKO used for quantitative analysis shown in Figure 5G–I. Images were taken using a JEOL 1010 electron microscope fitted with a Hamamatsu digital camera and AMT Advantage image capture software. Contrast of the images was adjusted using Photoshop software. The images in this archive were also used for the analysis in Figure 7. [file elife-50138-fig5-data1.zip › Figure 5 source data 1/WT #559 12d DD 7500X/6.30.17 #559 DD_012 contrast .tif]

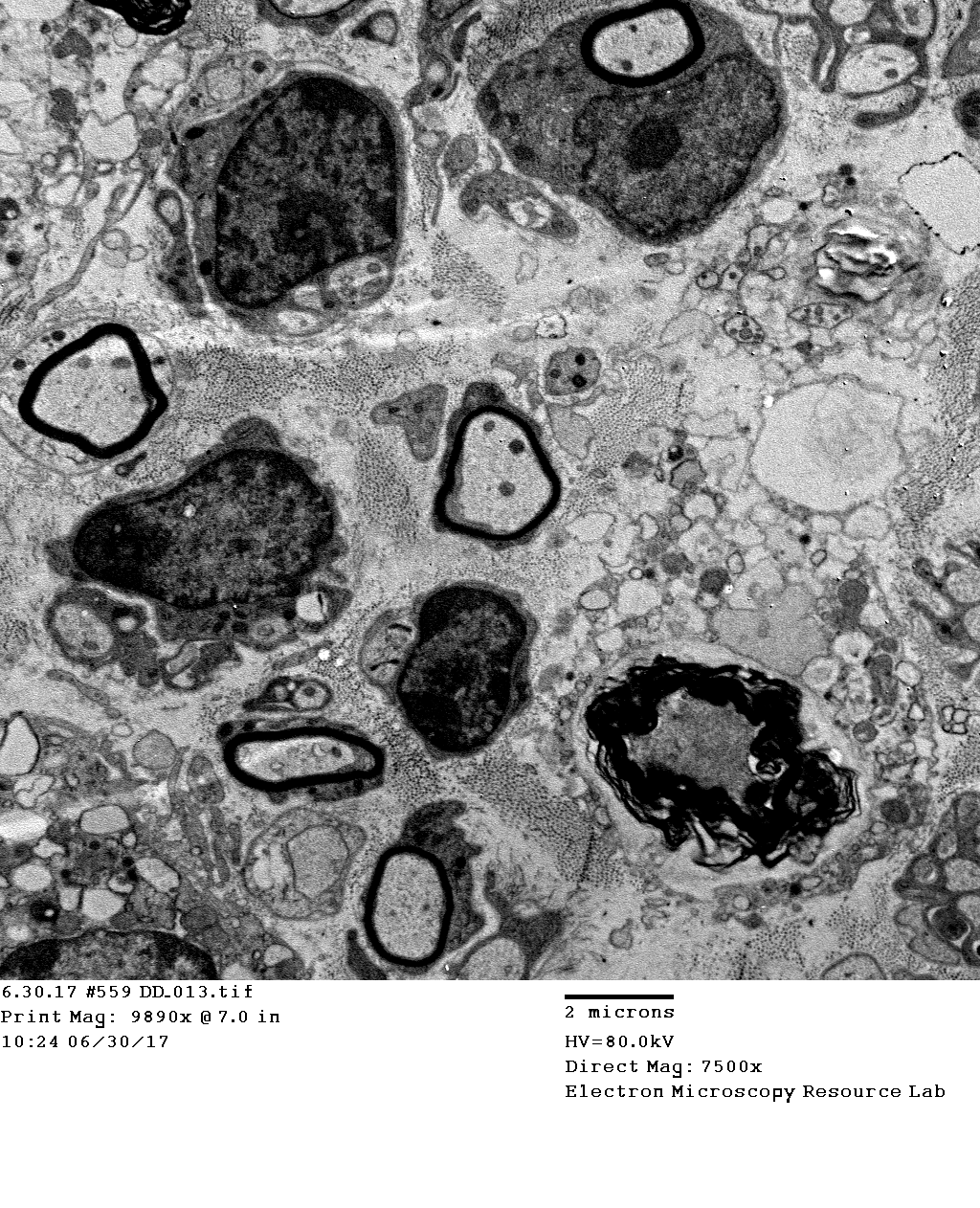

Supplement: Figure 5—source data 1. — This zip archive contains the TEM images for one WT and one iDKO used for quantitative analysis shown in Figure 5G–I. Images were taken using a JEOL 1010 electron microscope fitted with a Hamamatsu digital camera and AMT Advantage image capture software. Contrast of the images was adjusted using Photoshop software. The images in this archive were also used for the analysis in Figure 7. [file elife-50138-fig5-data1.zip › Figure 5 source data 1/WT #559 12d DD 7500X/6.30.17 #559 DD_013 contrast .tif]

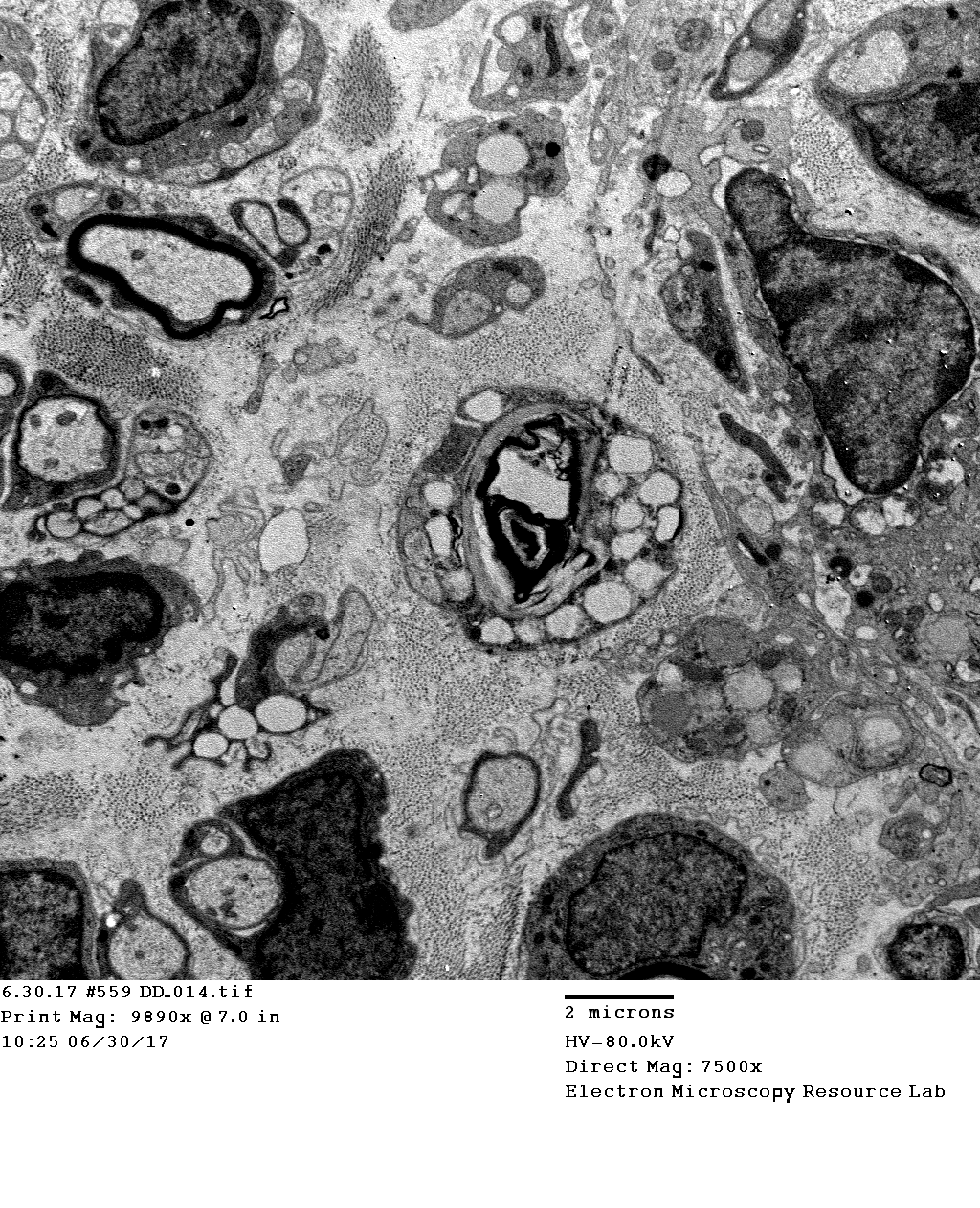

Supplement: Figure 5—source data 1. — This zip archive contains the TEM images for one WT and one iDKO used for quantitative analysis shown in Figure 5G–I. Images were taken using a JEOL 1010 electron microscope fitted with a Hamamatsu digital camera and AMT Advantage image capture software. Contrast of the images was adjusted using Photoshop software. The images in this archive were also used for the analysis in Figure 7. [file elife-50138-fig5-data1.zip › Figure 5 source data 1/WT #559 12d DD 7500X/6.30.17 #559 DD_014 contrast .tif]

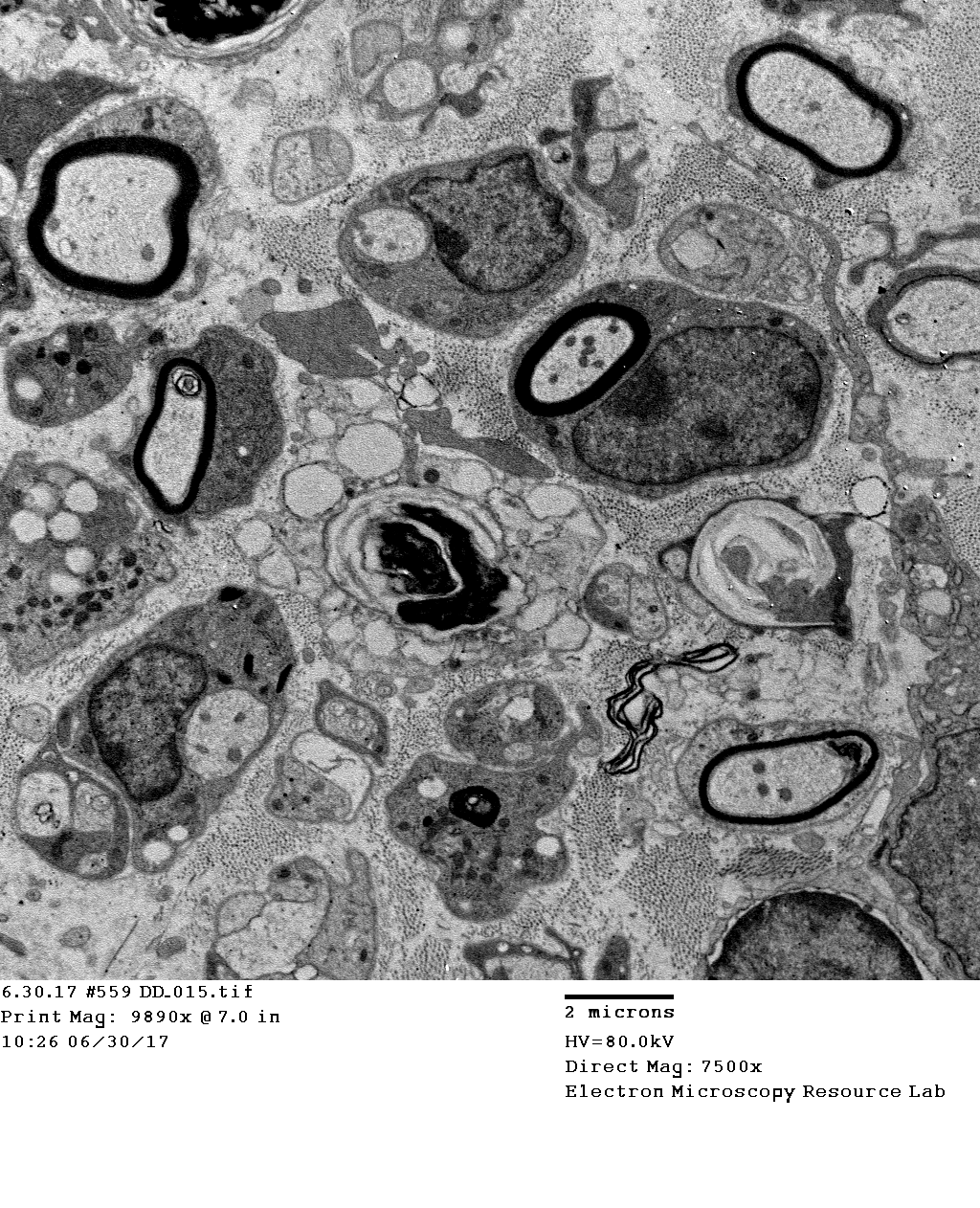

Supplement: Figure 5—source data 1. — This zip archive contains the TEM images for one WT and one iDKO used for quantitative analysis shown in Figure 5G–I. Images were taken using a JEOL 1010 electron microscope fitted with a Hamamatsu digital camera and AMT Advantage image capture software. Contrast of the images was adjusted using Photoshop software. The images in this archive were also used for the analysis in Figure 7. [file elife-50138-fig5-data1.zip › Figure 5 source data 1/WT #559 12d DD 7500X/6.30.17 #559 DD_015 contrast .tif]

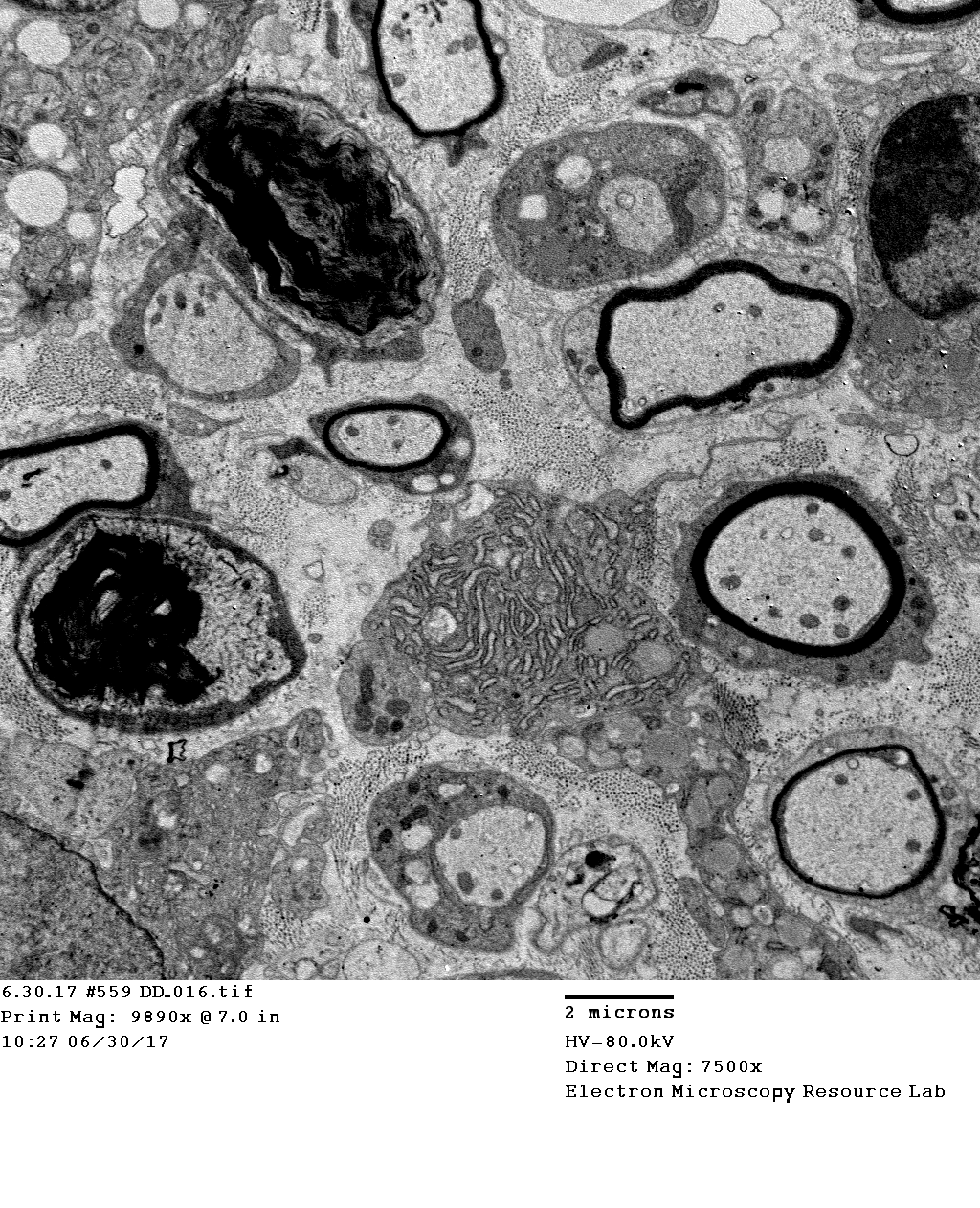

Supplement: Figure 5—source data 1. — This zip archive contains the TEM images for one WT and one iDKO used for quantitative analysis shown in Figure 5G–I. Images were taken using a JEOL 1010 electron microscope fitted with a Hamamatsu digital camera and AMT Advantage image capture software. Contrast of the images was adjusted using Photoshop software. The images in this archive were also used for the analysis in Figure 7. [file elife-50138-fig5-data1.zip › Figure 5 source data 1/WT #559 12d DD 7500X/6.30.17 #559 DD_016 contrast Y .tif]
