# Supplementary figures and images for: Axon-dependent expression of YAP/TAZ mediates Schwann cell remyelination but not proliferation after nerve injury (part 3 of 4)
Source: eLife. 2020 May 21;9:e50138. doi: 10.7554/eLife.50138 (PMC7259960; doi:10.7554/eLife.50138)

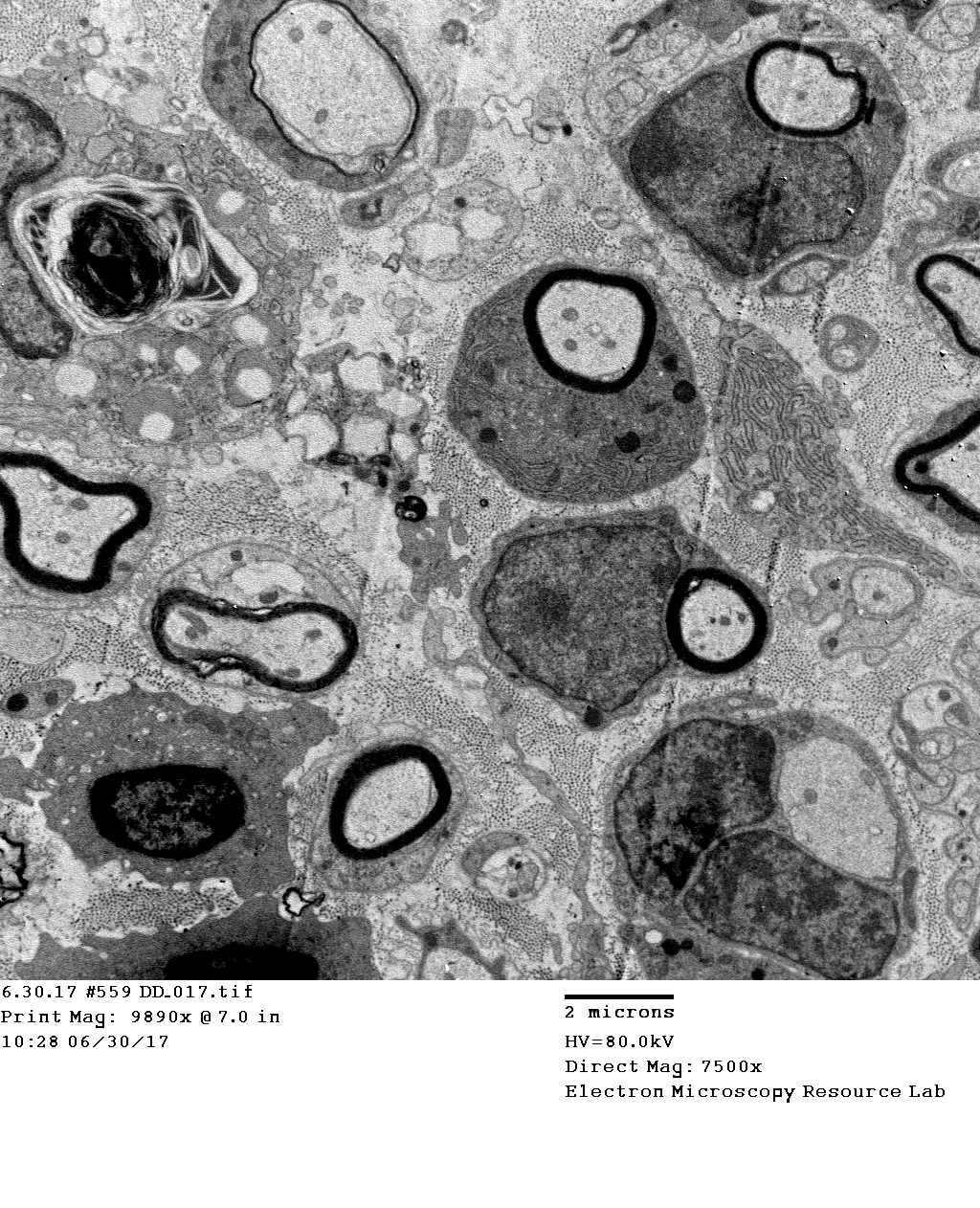

Supplement: Figure 5—source data 1. — This zip archive contains the TEM images for one WT and one iDKO used for quantitative analysis shown in Figure 5G–I. Images were taken using a JEOL 1010 electron microscope fitted with a Hamamatsu digital camera and AMT Advantage image capture software. Contrast of the images was adjusted using Photoshop software. The images in this archive were also used for the analysis in Figure 7. [file elife-50138-fig5-data1.zip › Figure 5 source data 1/WT #559 12d DD 7500X/6.30.17 #559 DD_017 contrast Y .tif]

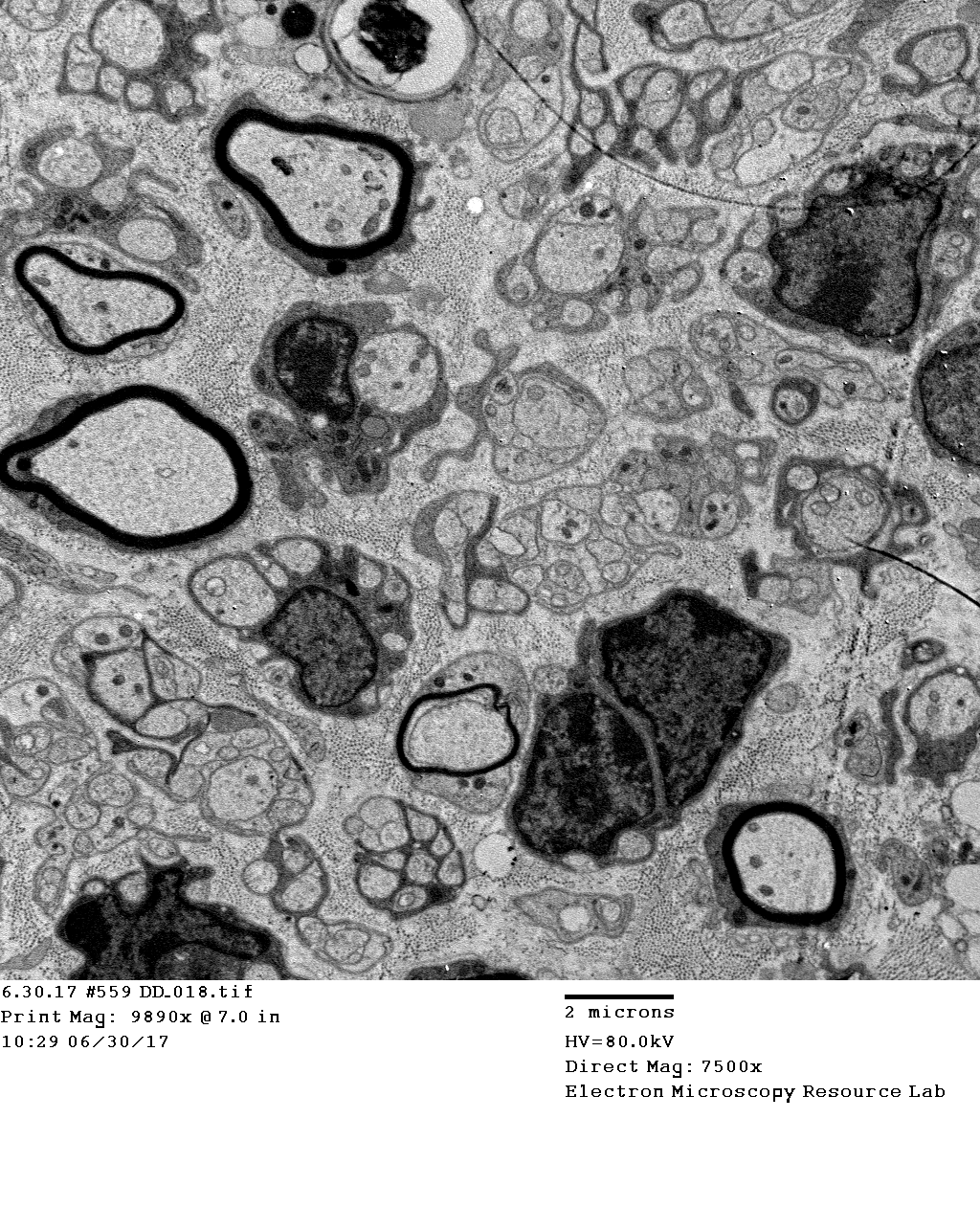

Supplement: Figure 5—source data 1. — This zip archive contains the TEM images for one WT and one iDKO used for quantitative analysis shown in Figure 5G–I. Images were taken using a JEOL 1010 electron microscope fitted with a Hamamatsu digital camera and AMT Advantage image capture software. Contrast of the images was adjusted using Photoshop software. The images in this archive were also used for the analysis in Figure 7. [file elife-50138-fig5-data1.zip › Figure 5 source data 1/WT #559 12d DD 7500X/6.30.17 #559 DD_018 contrast Y .tif]

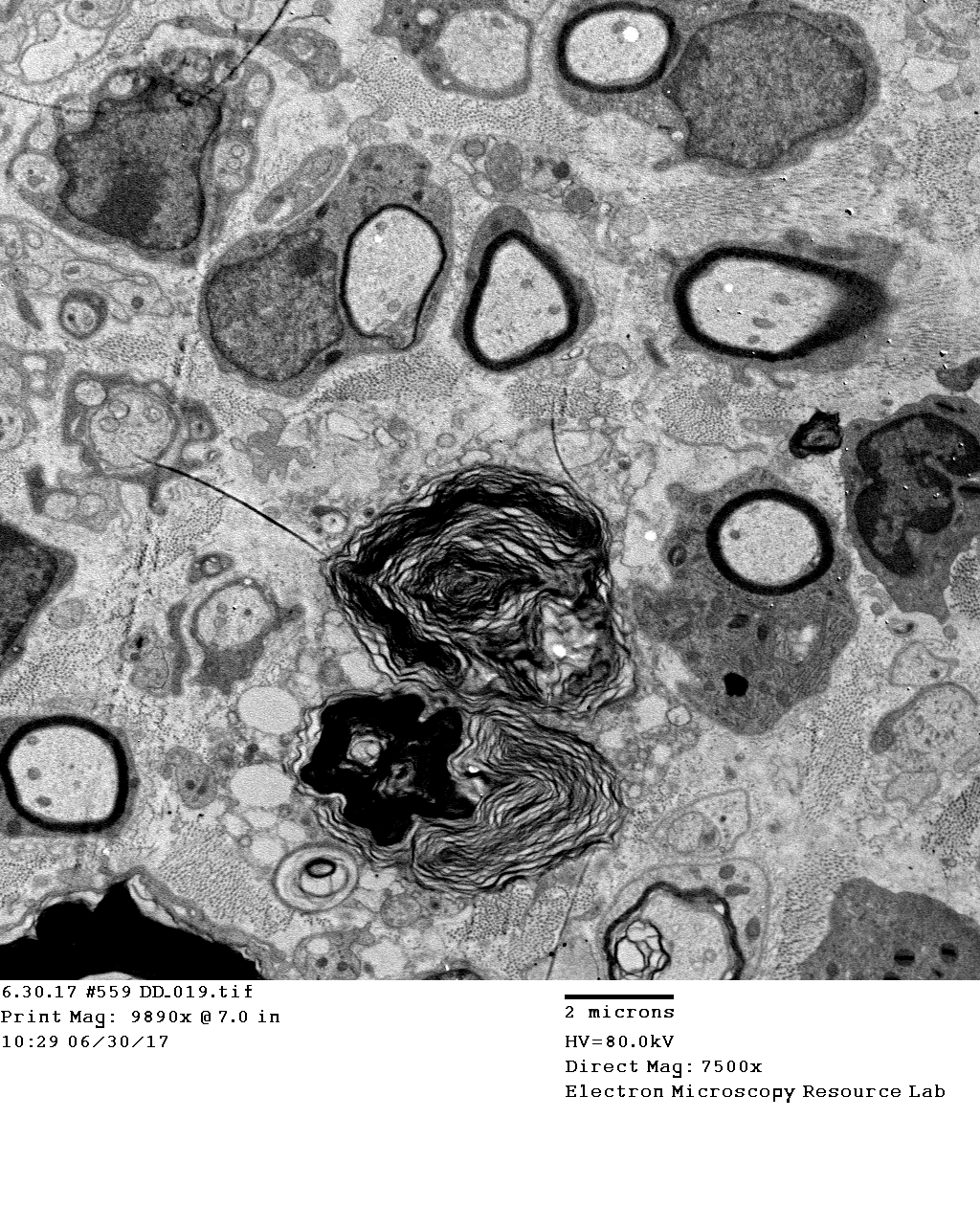

Supplement: Figure 5—source data 1. — This zip archive contains the TEM images for one WT and one iDKO used for quantitative analysis shown in Figure 5G–I. Images were taken using a JEOL 1010 electron microscope fitted with a Hamamatsu digital camera and AMT Advantage image capture software. Contrast of the images was adjusted using Photoshop software. The images in this archive were also used for the analysis in Figure 7. [file elife-50138-fig5-data1.zip › Figure 5 source data 1/WT #559 12d DD 7500X/6.30.17 #559 DD_019 contrast .tif]

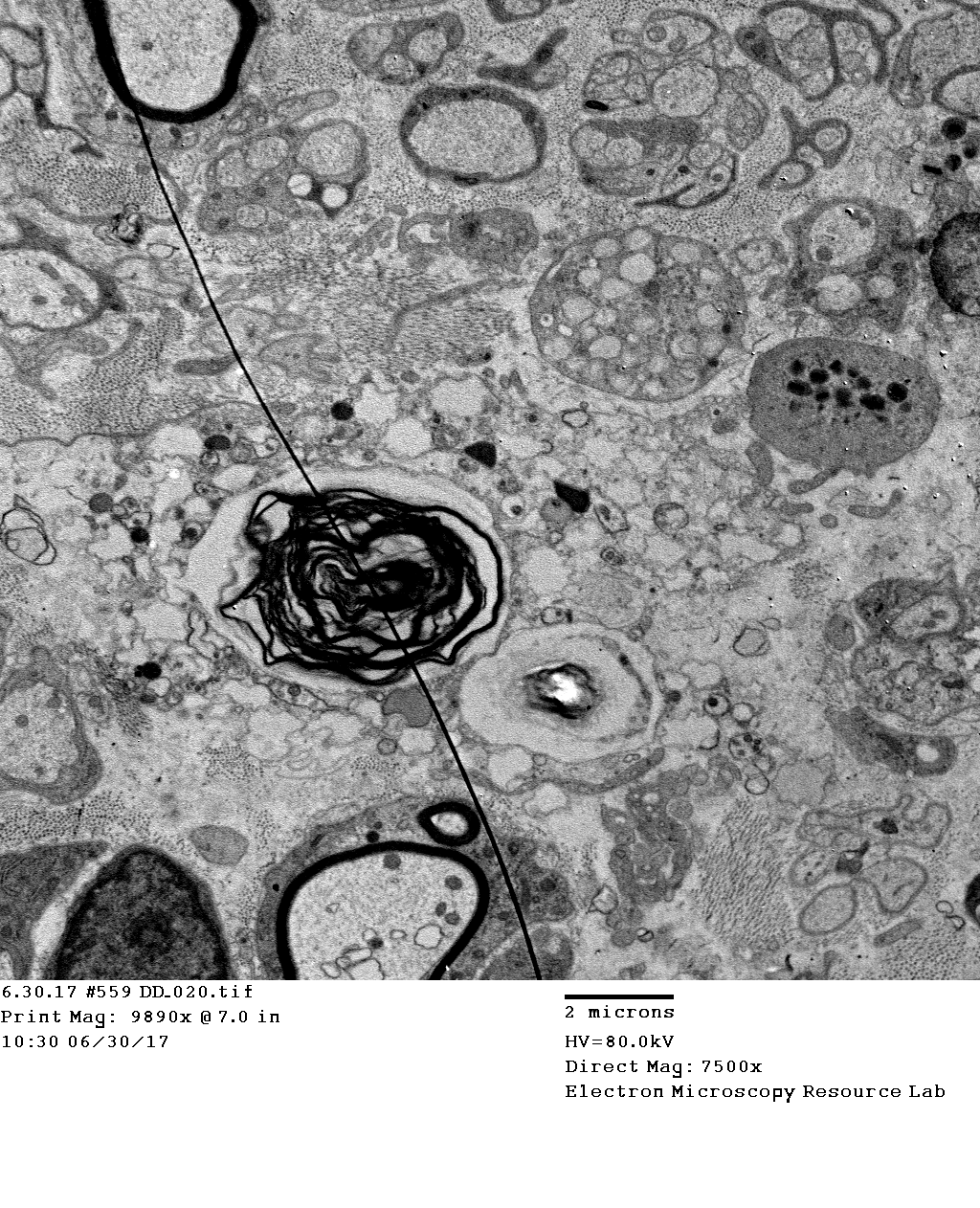

Supplement: Figure 5—source data 1. — This zip archive contains the TEM images for one WT and one iDKO used for quantitative analysis shown in Figure 5G–I. Images were taken using a JEOL 1010 electron microscope fitted with a Hamamatsu digital camera and AMT Advantage image capture software. Contrast of the images was adjusted using Photoshop software. The images in this archive were also used for the analysis in Figure 7. [file elife-50138-fig5-data1.zip › Figure 5 source data 1/WT #559 12d DD 7500X/6.30.17 #559 DD_020 contrast .tif]

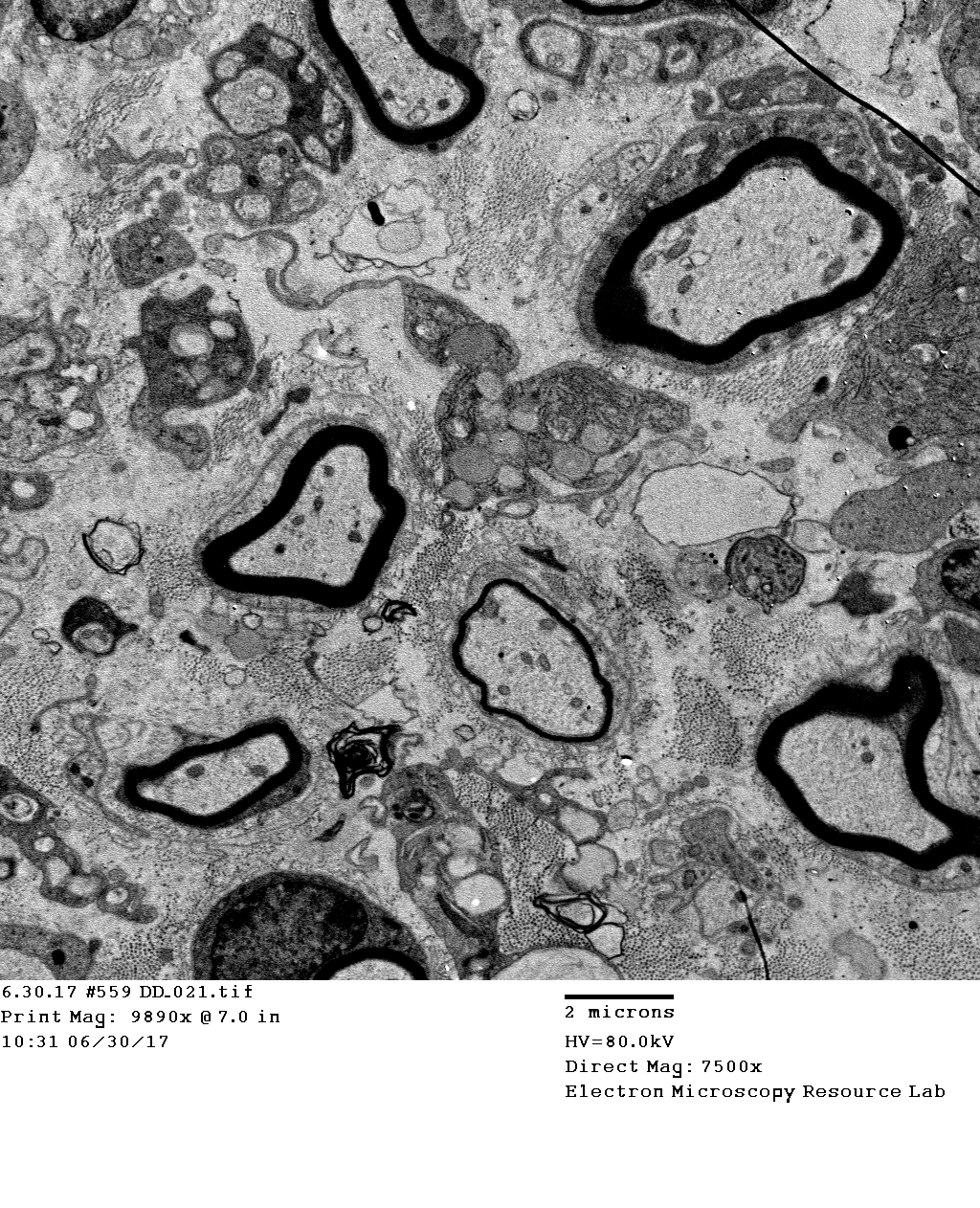

Supplement: Figure 5—source data 1. — This zip archive contains the TEM images for one WT and one iDKO used for quantitative analysis shown in Figure 5G–I. Images were taken using a JEOL 1010 electron microscope fitted with a Hamamatsu digital camera and AMT Advantage image capture software. Contrast of the images was adjusted using Photoshop software. The images in this archive were also used for the analysis in Figure 7. [file elife-50138-fig5-data1.zip › Figure 5 source data 1/WT #559 12d DD 7500X/6.30.17 #559 DD_021 contrast .tif]

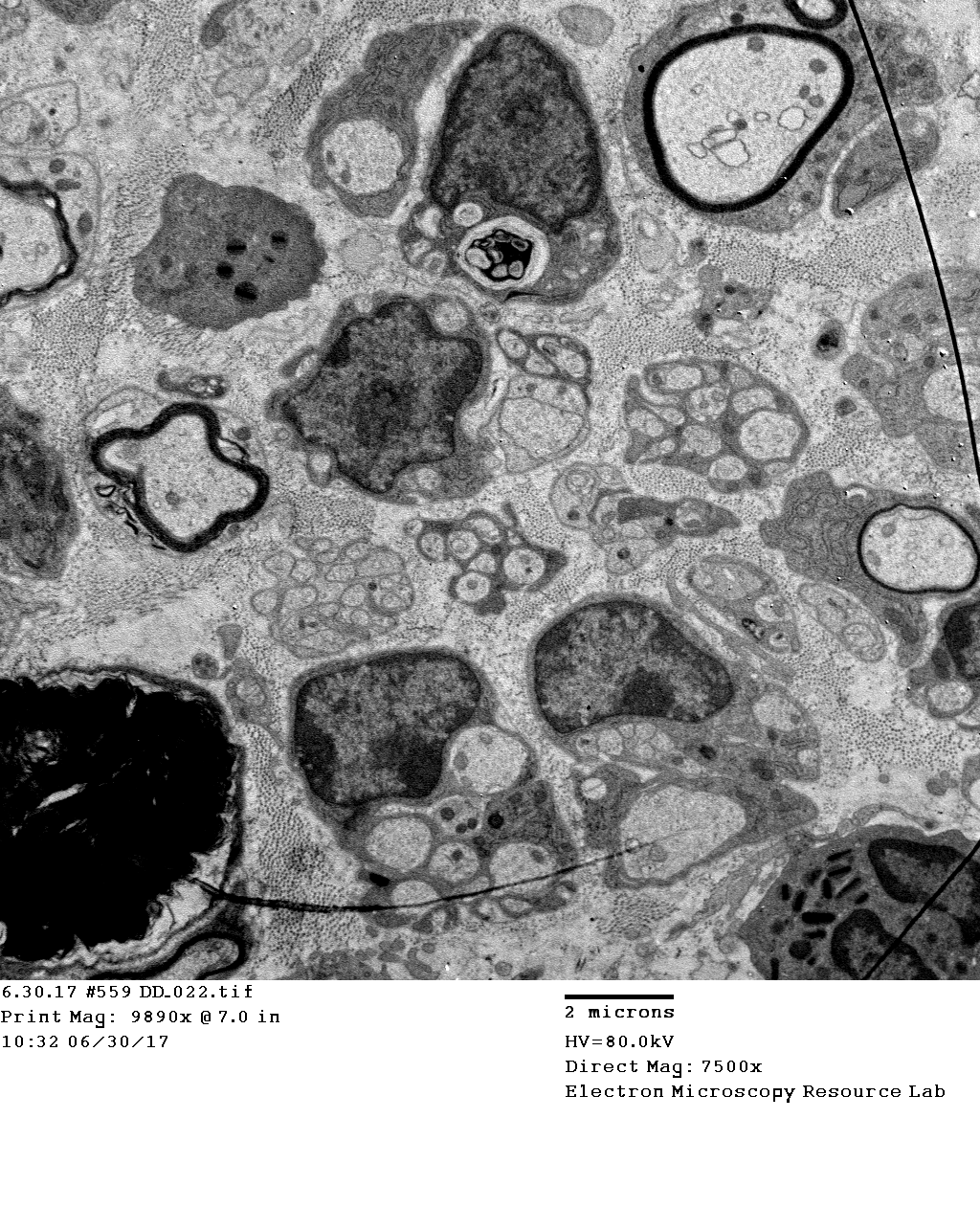

Supplement: Figure 5—source data 1. — This zip archive contains the TEM images for one WT and one iDKO used for quantitative analysis shown in Figure 5G–I. Images were taken using a JEOL 1010 electron microscope fitted with a Hamamatsu digital camera and AMT Advantage image capture software. Contrast of the images was adjusted using Photoshop software. The images in this archive were also used for the analysis in Figure 7. [file elife-50138-fig5-data1.zip › Figure 5 source data 1/WT #559 12d DD 7500X/6.30.17 #559 DD_022 contrast .tif]

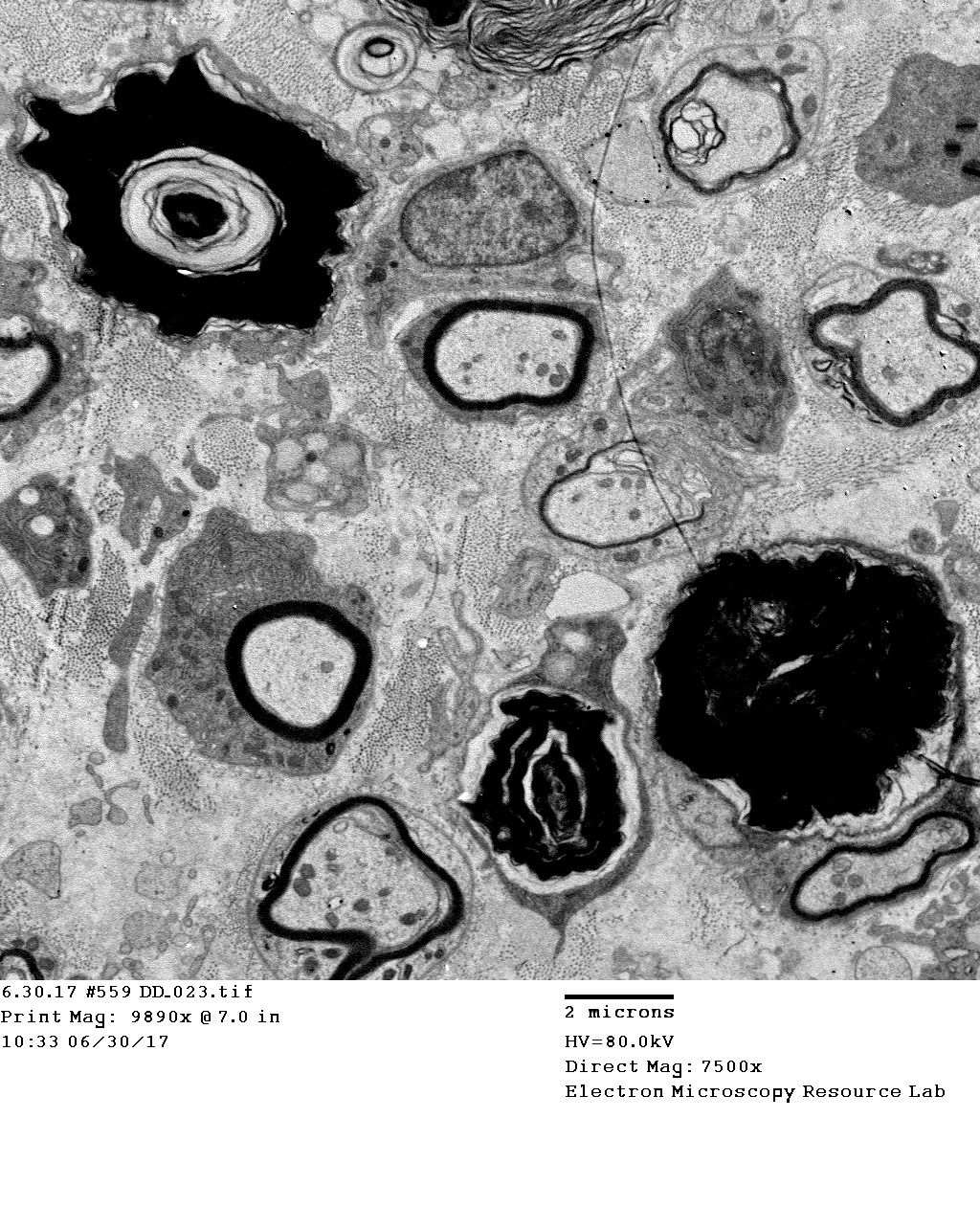

Supplement: Figure 5—source data 1. — This zip archive contains the TEM images for one WT and one iDKO used for quantitative analysis shown in Figure 5G–I. Images were taken using a JEOL 1010 electron microscope fitted with a Hamamatsu digital camera and AMT Advantage image capture software. Contrast of the images was adjusted using Photoshop software. The images in this archive were also used for the analysis in Figure 7. [file elife-50138-fig5-data1.zip › Figure 5 source data 1/WT #559 12d DD 7500X/6.30.17 #559 DD_023 contrast .tif]

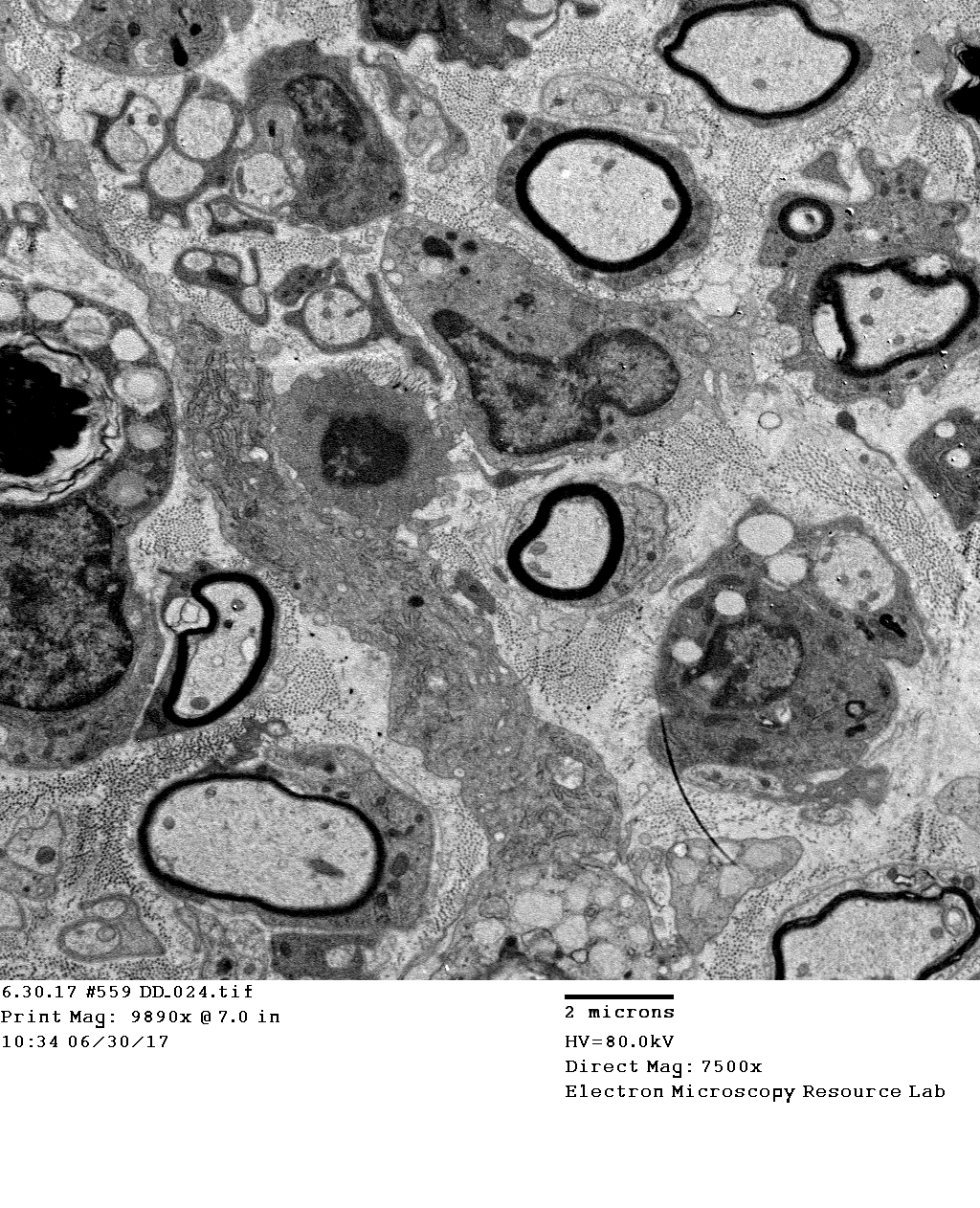

Supplement: Figure 5—source data 1. — This zip archive contains the TEM images for one WT and one iDKO used for quantitative analysis shown in Figure 5G–I. Images were taken using a JEOL 1010 electron microscope fitted with a Hamamatsu digital camera and AMT Advantage image capture software. Contrast of the images was adjusted using Photoshop software. The images in this archive were also used for the analysis in Figure 7. [file elife-50138-fig5-data1.zip › Figure 5 source data 1/WT #559 12d DD 7500X/6.30.17 #559 DD_024 contrast .tif]

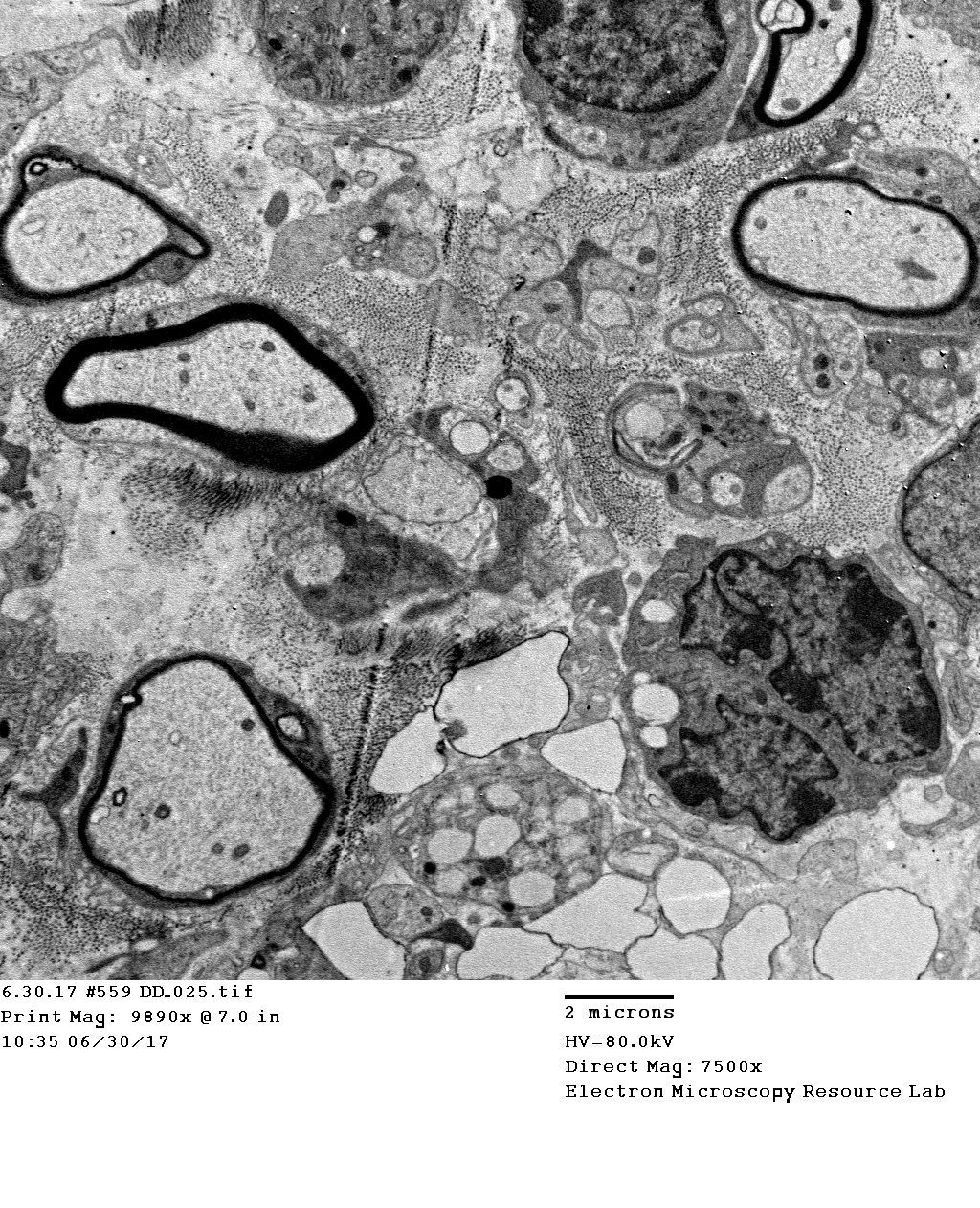

Supplement: Figure 5—source data 1. — This zip archive contains the TEM images for one WT and one iDKO used for quantitative analysis shown in Figure 5G–I. Images were taken using a JEOL 1010 electron microscope fitted with a Hamamatsu digital camera and AMT Advantage image capture software. Contrast of the images was adjusted using Photoshop software. The images in this archive were also used for the analysis in Figure 7. [file elife-50138-fig5-data1.zip › Figure 5 source data 1/WT #559 12d DD 7500X/6.30.17 #559 DD_025 contrast .tif]

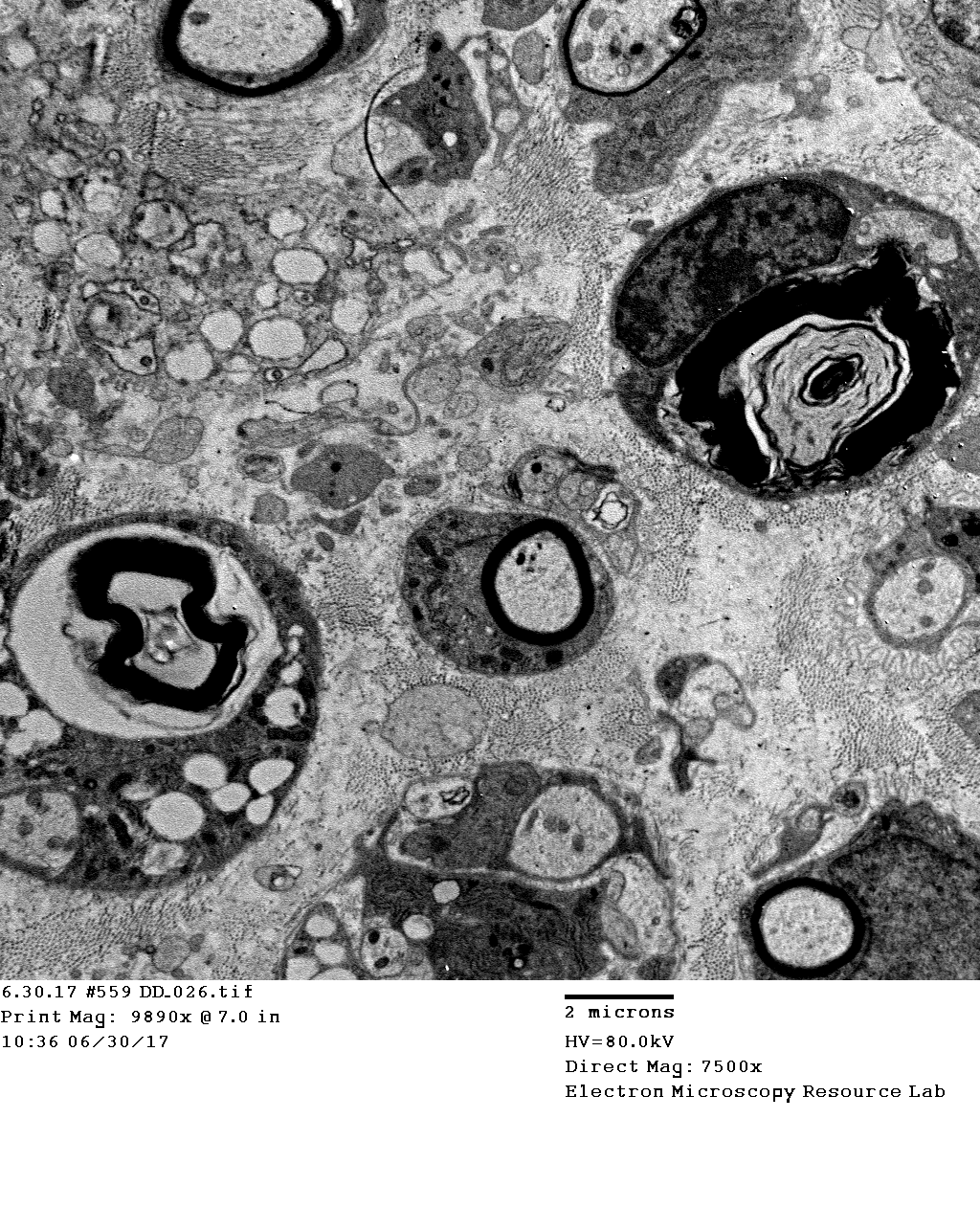

Supplement: Figure 5—source data 1. — This zip archive contains the TEM images for one WT and one iDKO used for quantitative analysis shown in Figure 5G–I. Images were taken using a JEOL 1010 electron microscope fitted with a Hamamatsu digital camera and AMT Advantage image capture software. Contrast of the images was adjusted using Photoshop software. The images in this archive were also used for the analysis in Figure 7. [file elife-50138-fig5-data1.zip › Figure 5 source data 1/WT #559 12d DD 7500X/6.30.17 #559 DD_026 contrast .tif]

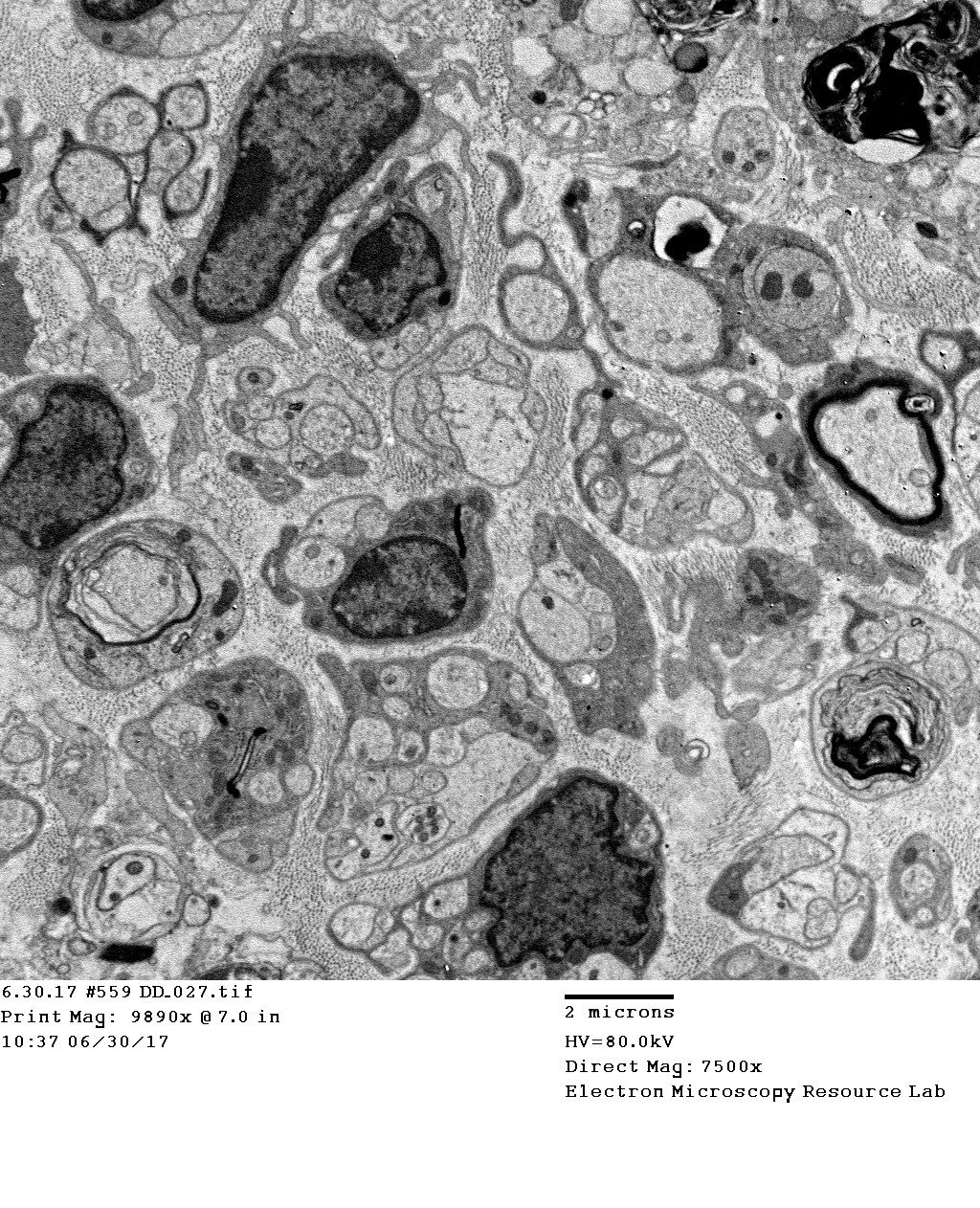

Supplement: Figure 5—source data 1. — This zip archive contains the TEM images for one WT and one iDKO used for quantitative analysis shown in Figure 5G–I. Images were taken using a JEOL 1010 electron microscope fitted with a Hamamatsu digital camera and AMT Advantage image capture software. Contrast of the images was adjusted using Photoshop software. The images in this archive were also used for the analysis in Figure 7. [file elife-50138-fig5-data1.zip › Figure 5 source data 1/WT #559 12d DD 7500X/6.30.17 #559 DD_027 contrast .tif]

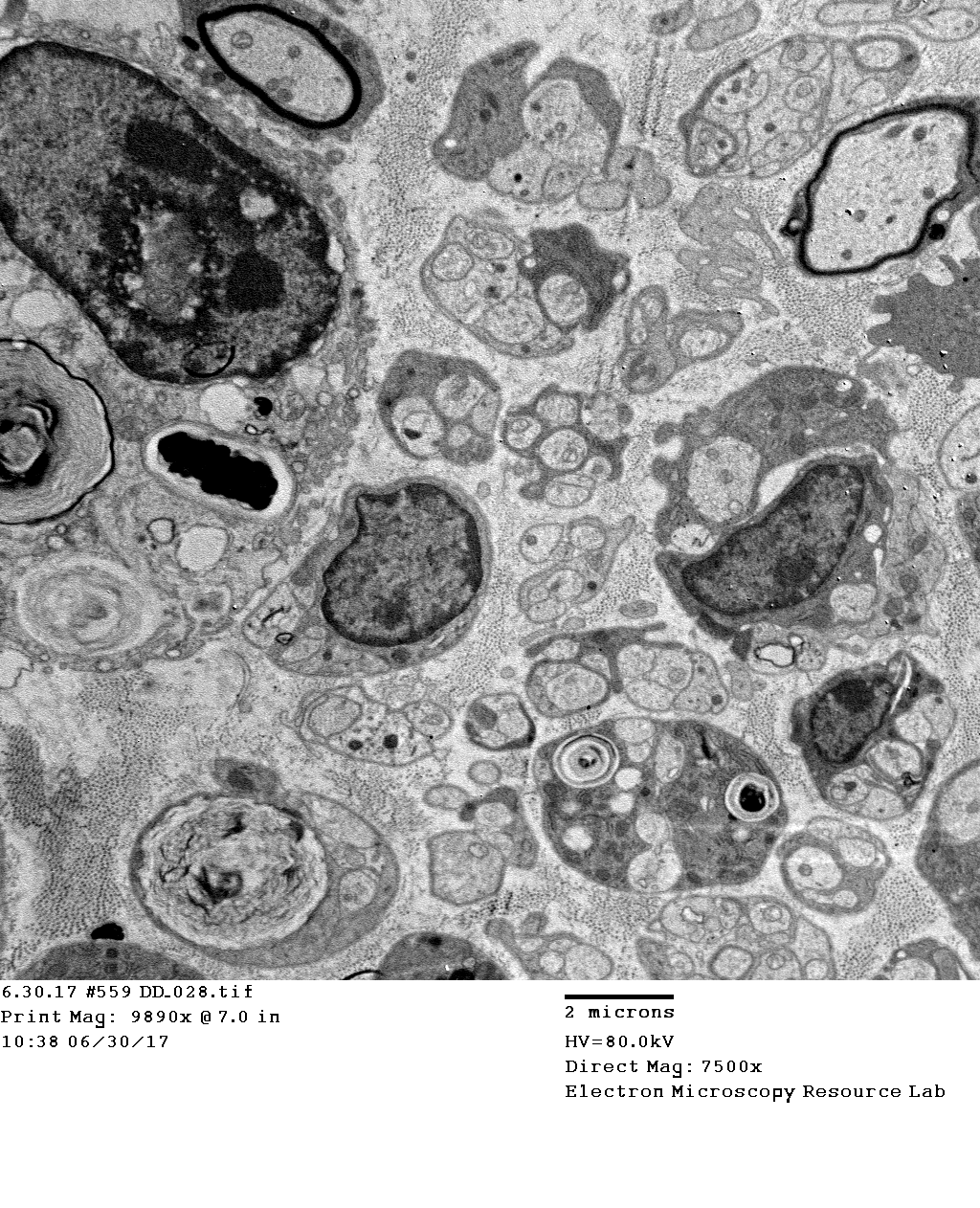

Supplement: Figure 5—source data 1. — This zip archive contains the TEM images for one WT and one iDKO used for quantitative analysis shown in Figure 5G–I. Images were taken using a JEOL 1010 electron microscope fitted with a Hamamatsu digital camera and AMT Advantage image capture software. Contrast of the images was adjusted using Photoshop software. The images in this archive were also used for the analysis in Figure 7. [file elife-50138-fig5-data1.zip › Figure 5 source data 1/WT #559 12d DD 7500X/6.30.17 #559 DD_028 contrast .tif]

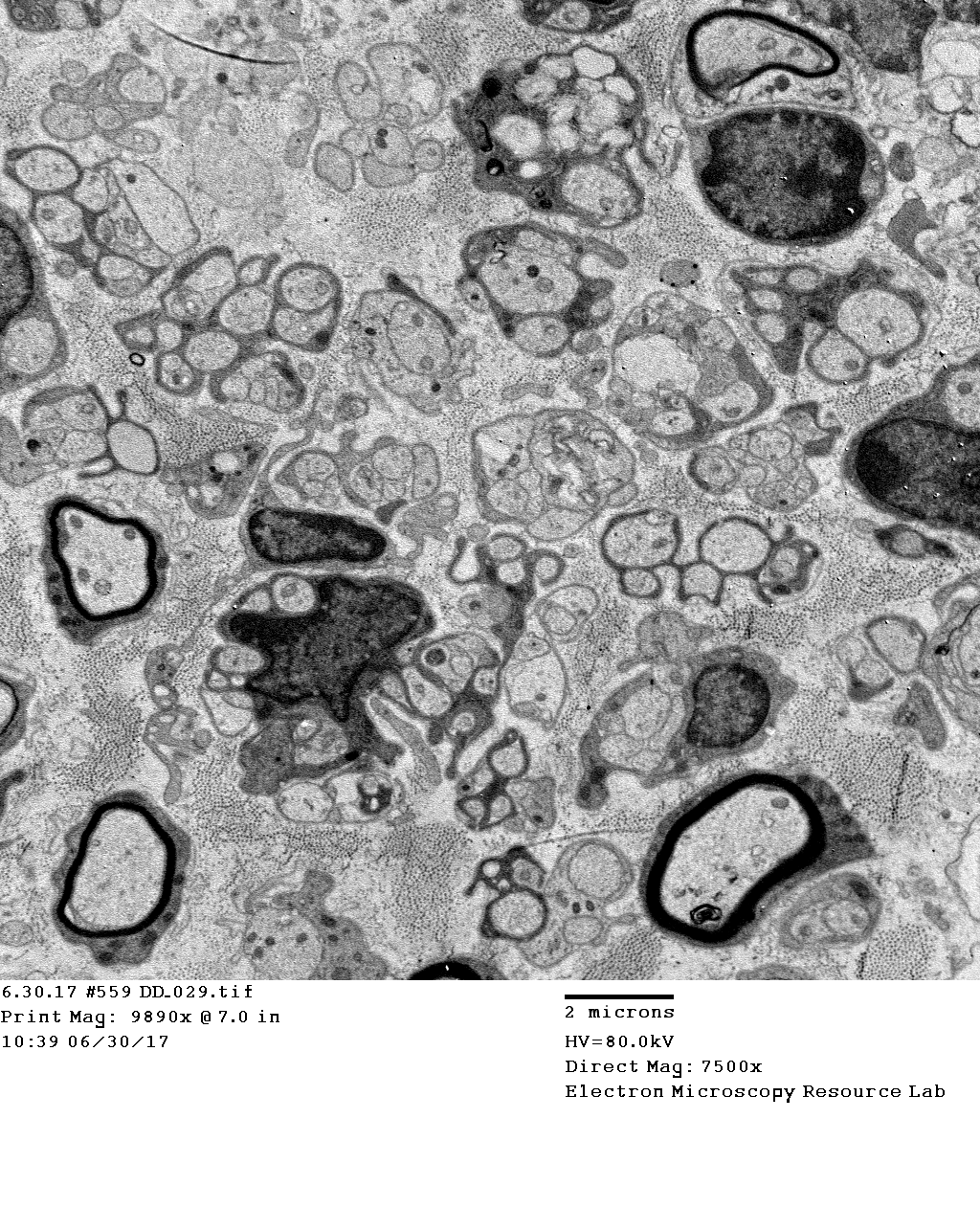

Supplement: Figure 5—source data 1. — This zip archive contains the TEM images for one WT and one iDKO used for quantitative analysis shown in Figure 5G–I. Images were taken using a JEOL 1010 electron microscope fitted with a Hamamatsu digital camera and AMT Advantage image capture software. Contrast of the images was adjusted using Photoshop software. The images in this archive were also used for the analysis in Figure 7. [file elife-50138-fig5-data1.zip › Figure 5 source data 1/WT #559 12d DD 7500X/6.30.17 #559 DD_029 contrast .tif]

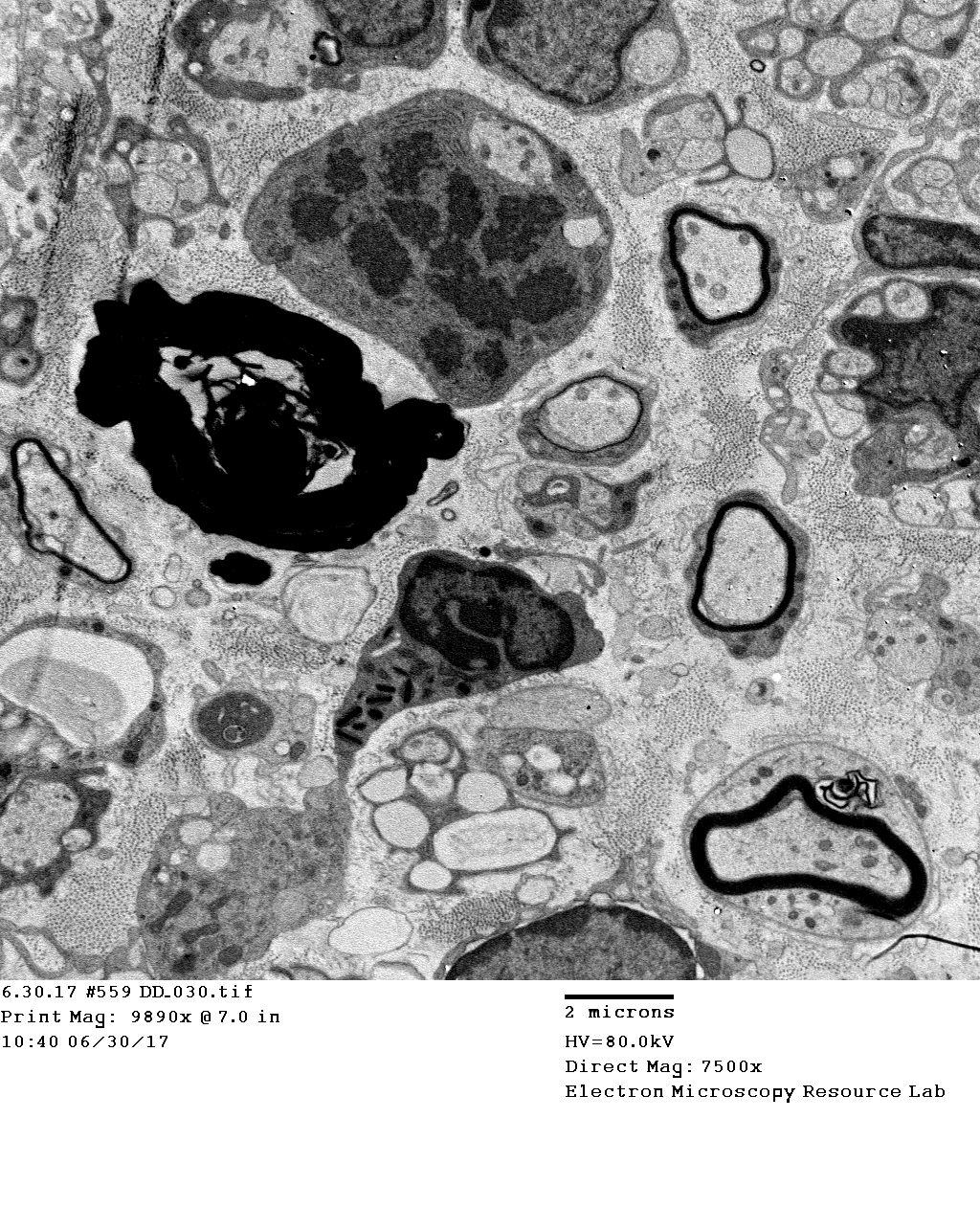

Supplement: Figure 5—source data 1. — This zip archive contains the TEM images for one WT and one iDKO used for quantitative analysis shown in Figure 5G–I. Images were taken using a JEOL 1010 electron microscope fitted with a Hamamatsu digital camera and AMT Advantage image capture software. Contrast of the images was adjusted using Photoshop software. The images in this archive were also used for the analysis in Figure 7. [file elife-50138-fig5-data1.zip › Figure 5 source data 1/WT #559 12d DD 7500X/6.30.17 #559 DD_030 contrast .tif]

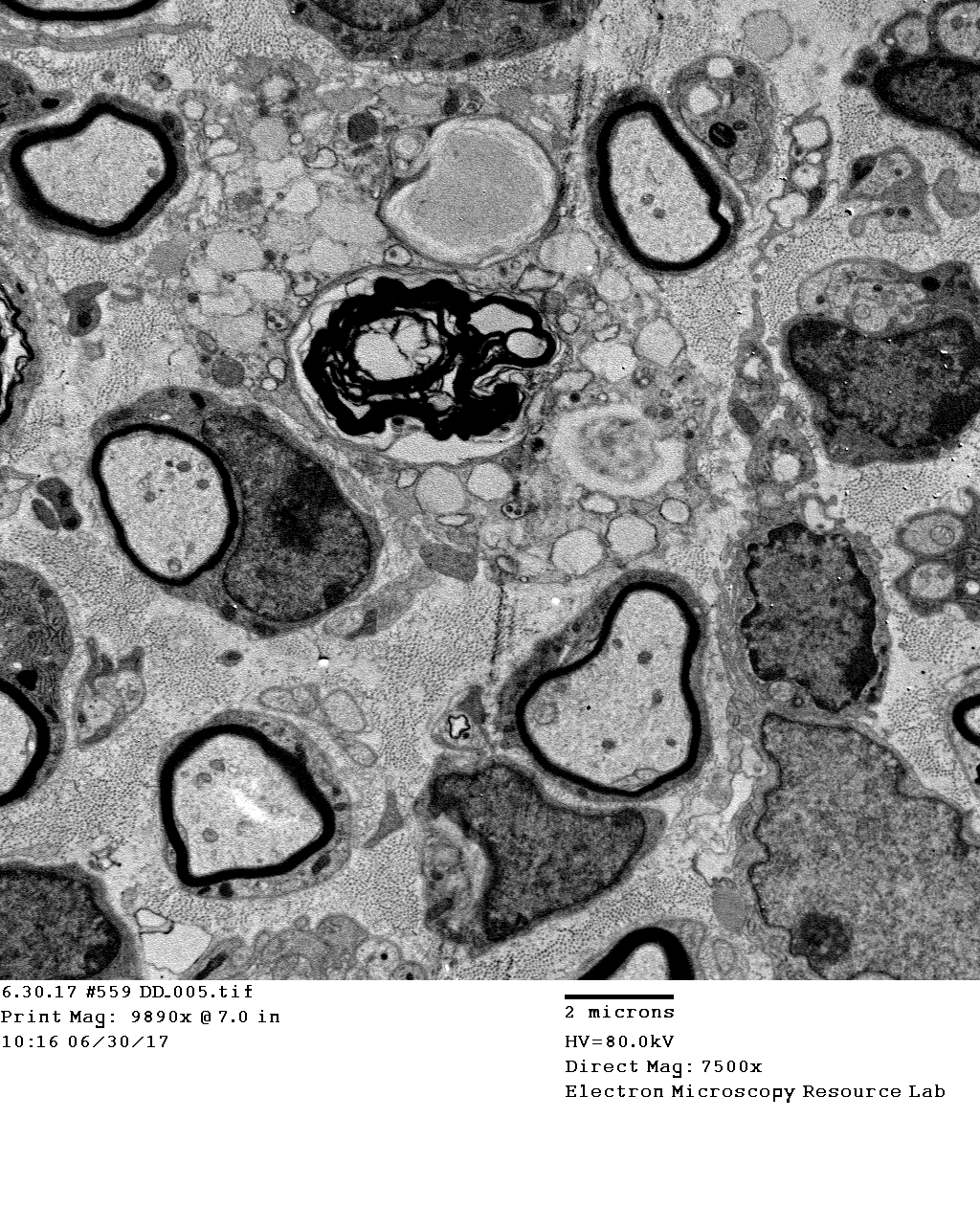

Supplement: Figure 5—source data 1. — This zip archive contains the TEM images for one WT and one iDKO used for quantitative analysis shown in Figure 5G–I. Images were taken using a JEOL 1010 electron microscope fitted with a Hamamatsu digital camera and AMT Advantage image capture software. Contrast of the images was adjusted using Photoshop software. The images in this archive were also used for the analysis in Figure 7. [file elife-50138-fig5-data1.zip › Figure 5 source data 1/WT #559 12d DD 7500X/GRATIO-6.30.17 #559 DD_005 contrast Y.tif]

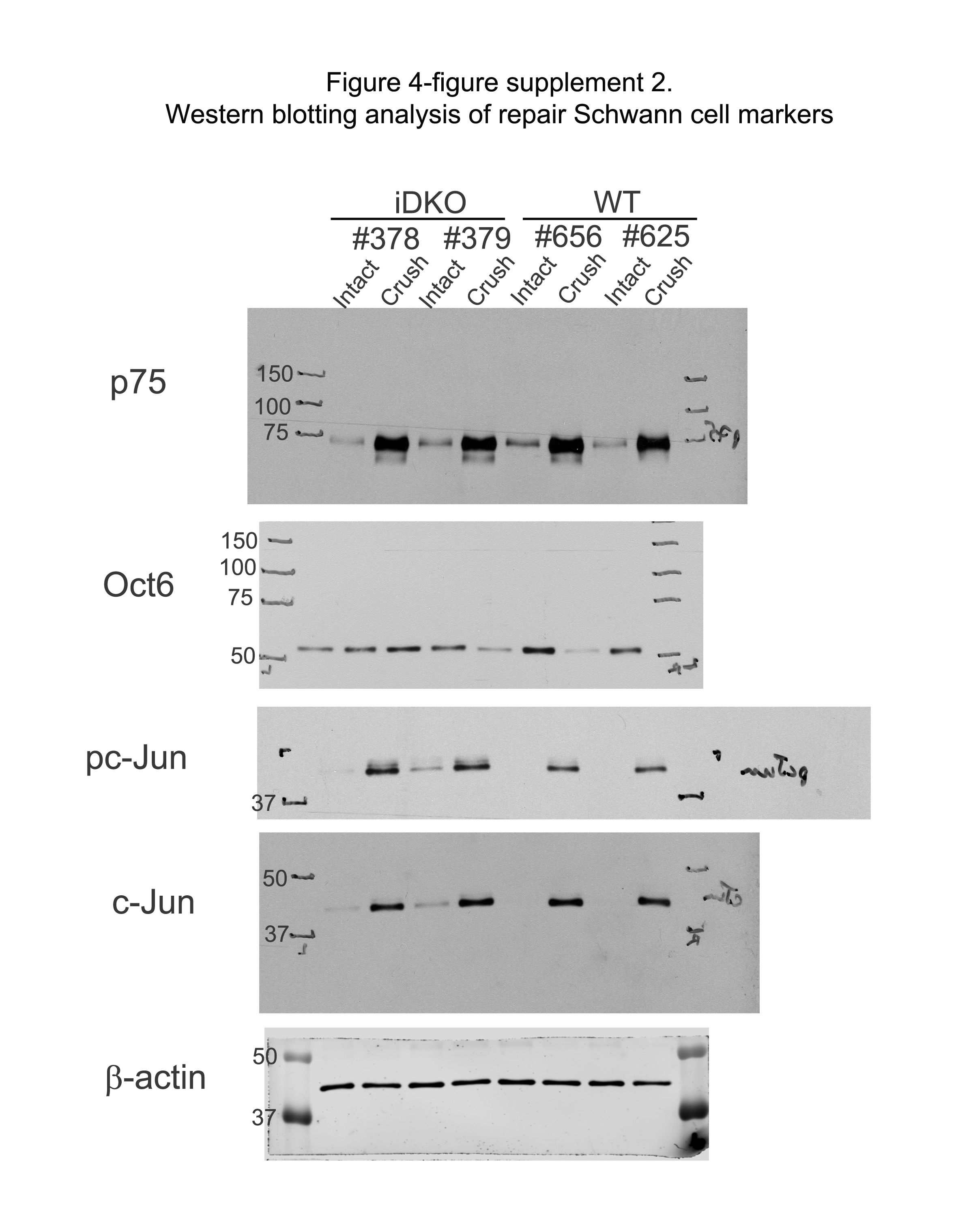

Supplement: Figure 6—source data 1. — This zip archive contains the raw data for WT and iDKO used for the quantitative analysis shown in Figure 6E and F. The data are contained in both a text document and an Excel file, both labeled as Mann Whitney data. These files also contain data for Figure 3—figure supplement 1, Figures 3, 4, 5, 7, 8A, Figure 8—figure supplement 1E. [file elife-50138-fig6-data1.zip › Figure 4figure supplement 2 R2.tif]

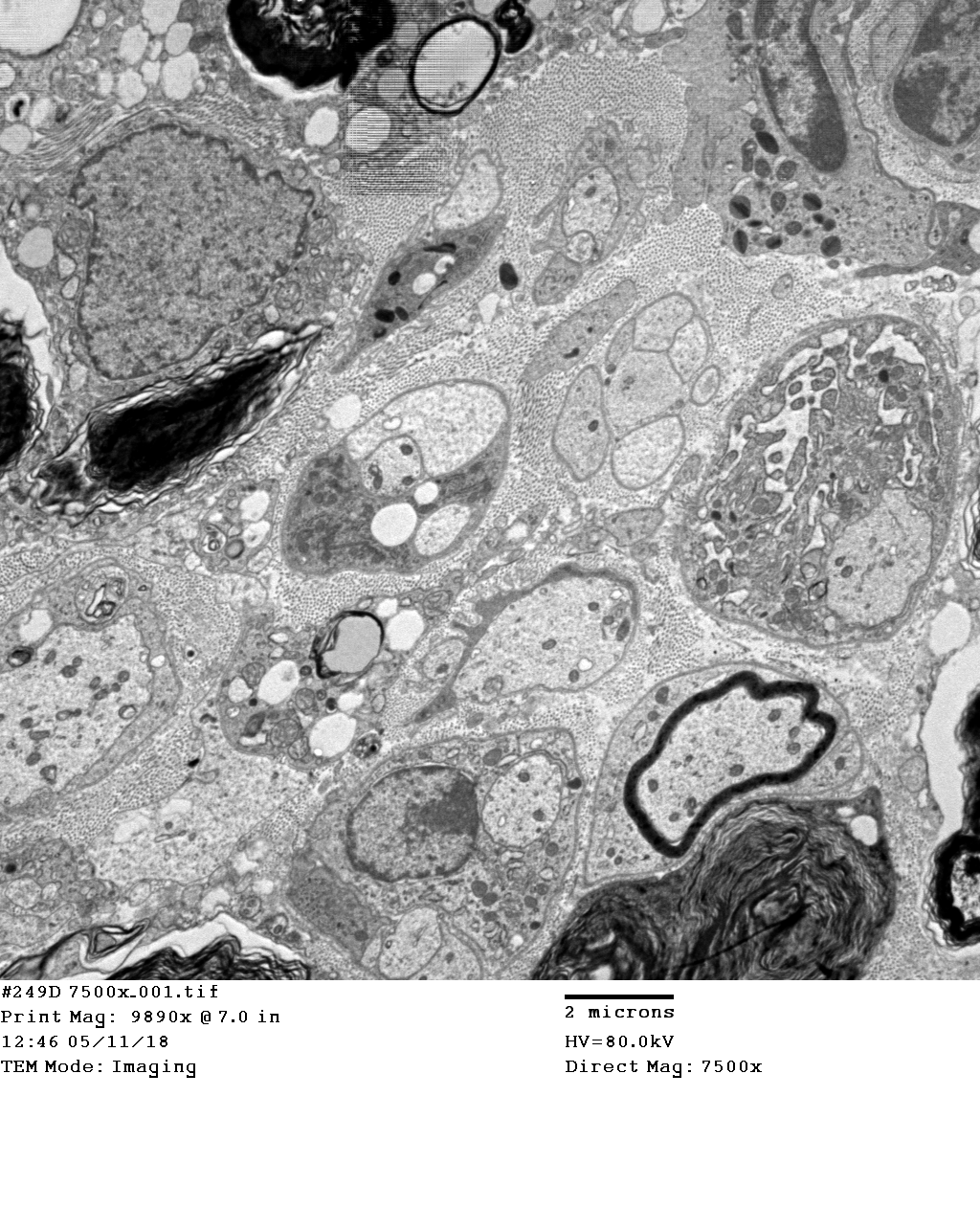

Supplement: Figure 8—source data 1. — This zip archive contains the TEM images for one WT and one Taz iKO used for quantitative analysis shown in Figure 8D–G. Images were taken using a JEOL 1010 electron microscope fitted with a Hamamatsu digital camera and AMT Advantage image capture software. Contrast of the images was adjusted using Photoshop software. [file elife-50138-fig8-data1.zip › Figure 8 source data 1/Taz iKO #249D 7500x/#249D 7500x_001 adjusted.tif]

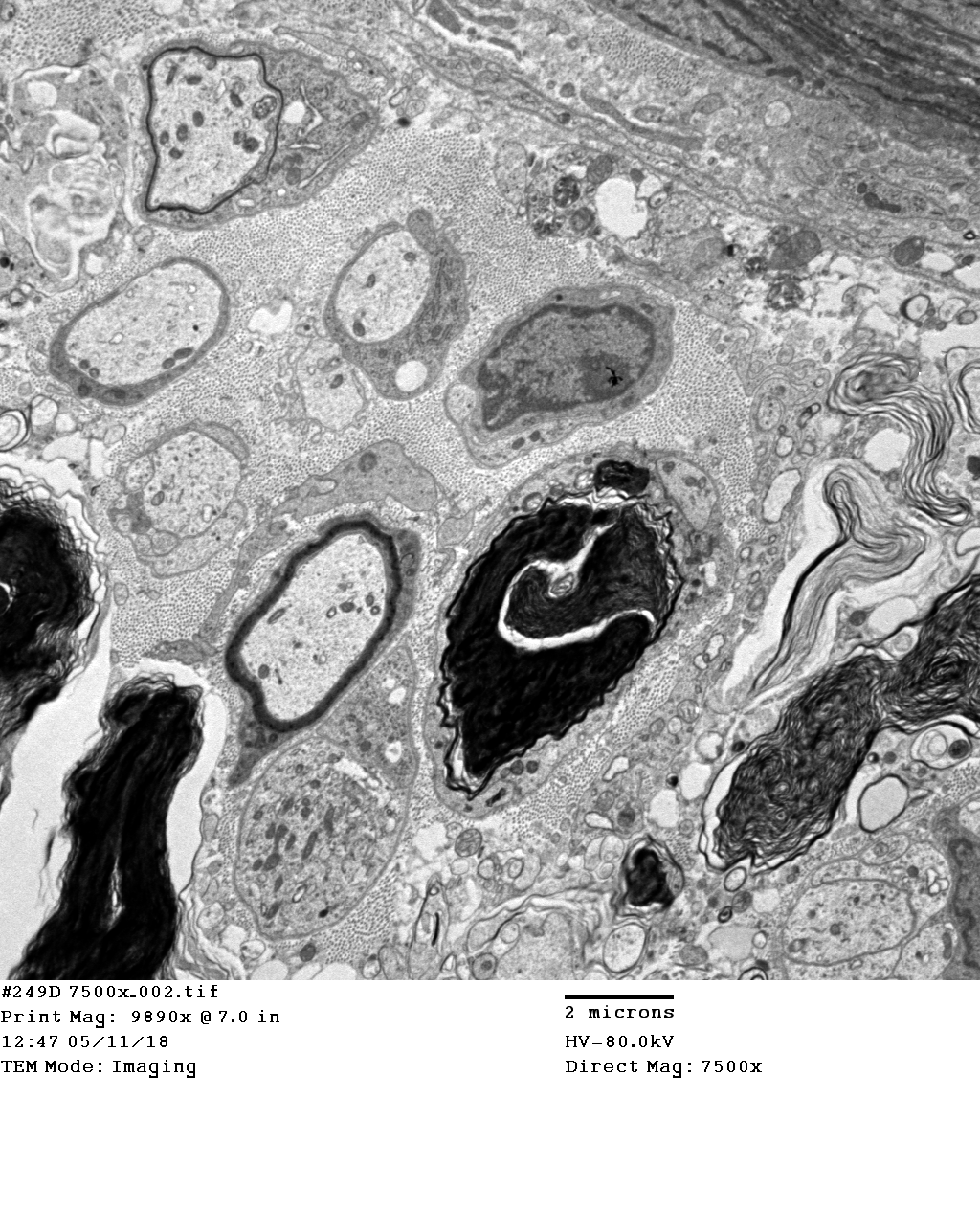

Supplement: Figure 8—source data 1. — This zip archive contains the TEM images for one WT and one Taz iKO used for quantitative analysis shown in Figure 8D–G. Images were taken using a JEOL 1010 electron microscope fitted with a Hamamatsu digital camera and AMT Advantage image capture software. Contrast of the images was adjusted using Photoshop software. [file elife-50138-fig8-data1.zip › Figure 8 source data 1/Taz iKO #249D 7500x/#249D 7500x_002 adjusted.tif]

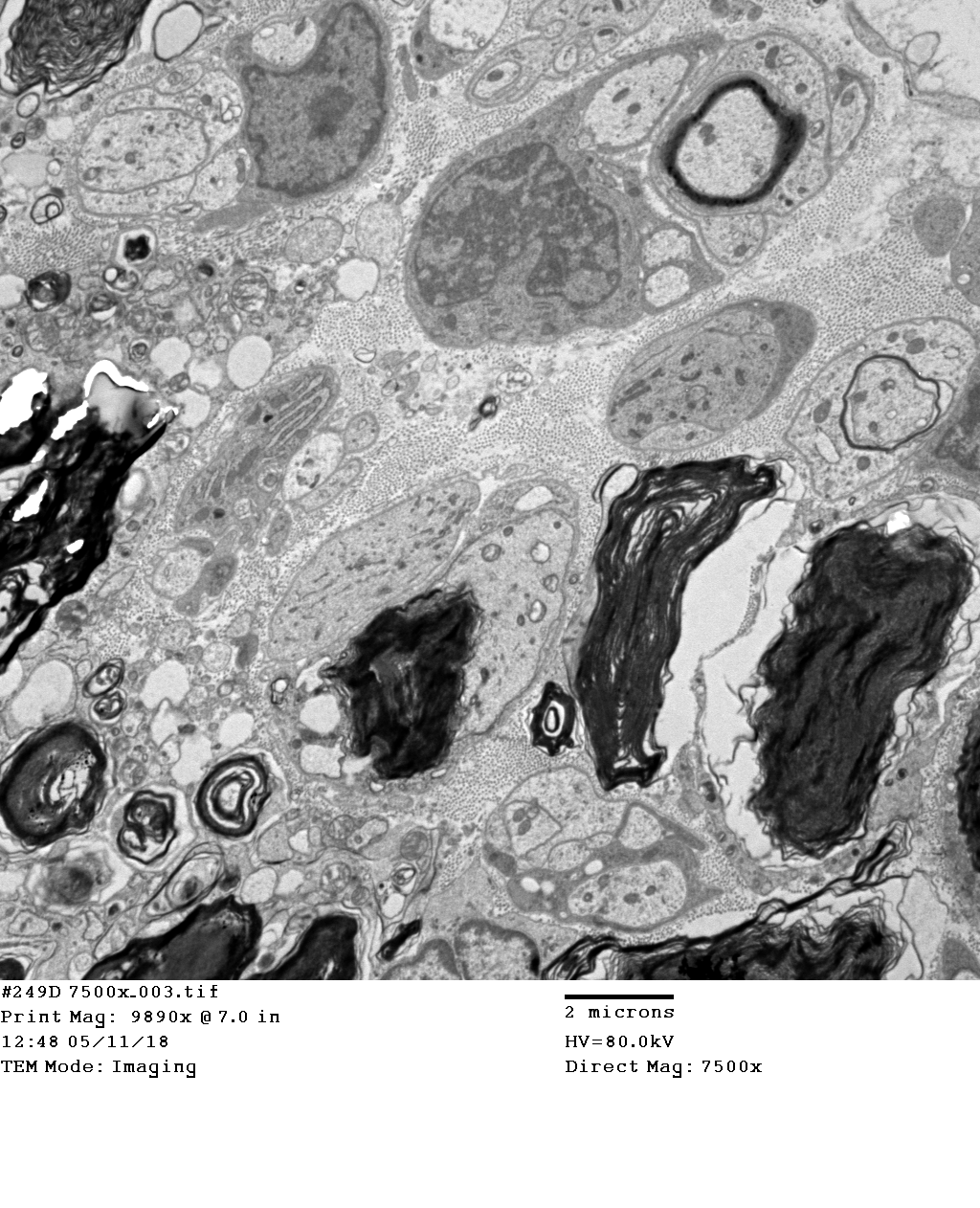

Supplement: Figure 8—source data 1. — This zip archive contains the TEM images for one WT and one Taz iKO used for quantitative analysis shown in Figure 8D–G. Images were taken using a JEOL 1010 electron microscope fitted with a Hamamatsu digital camera and AMT Advantage image capture software. Contrast of the images was adjusted using Photoshop software. [file elife-50138-fig8-data1.zip › Figure 8 source data 1/Taz iKO #249D 7500x/#249D 7500x_003 adjusted.tif]

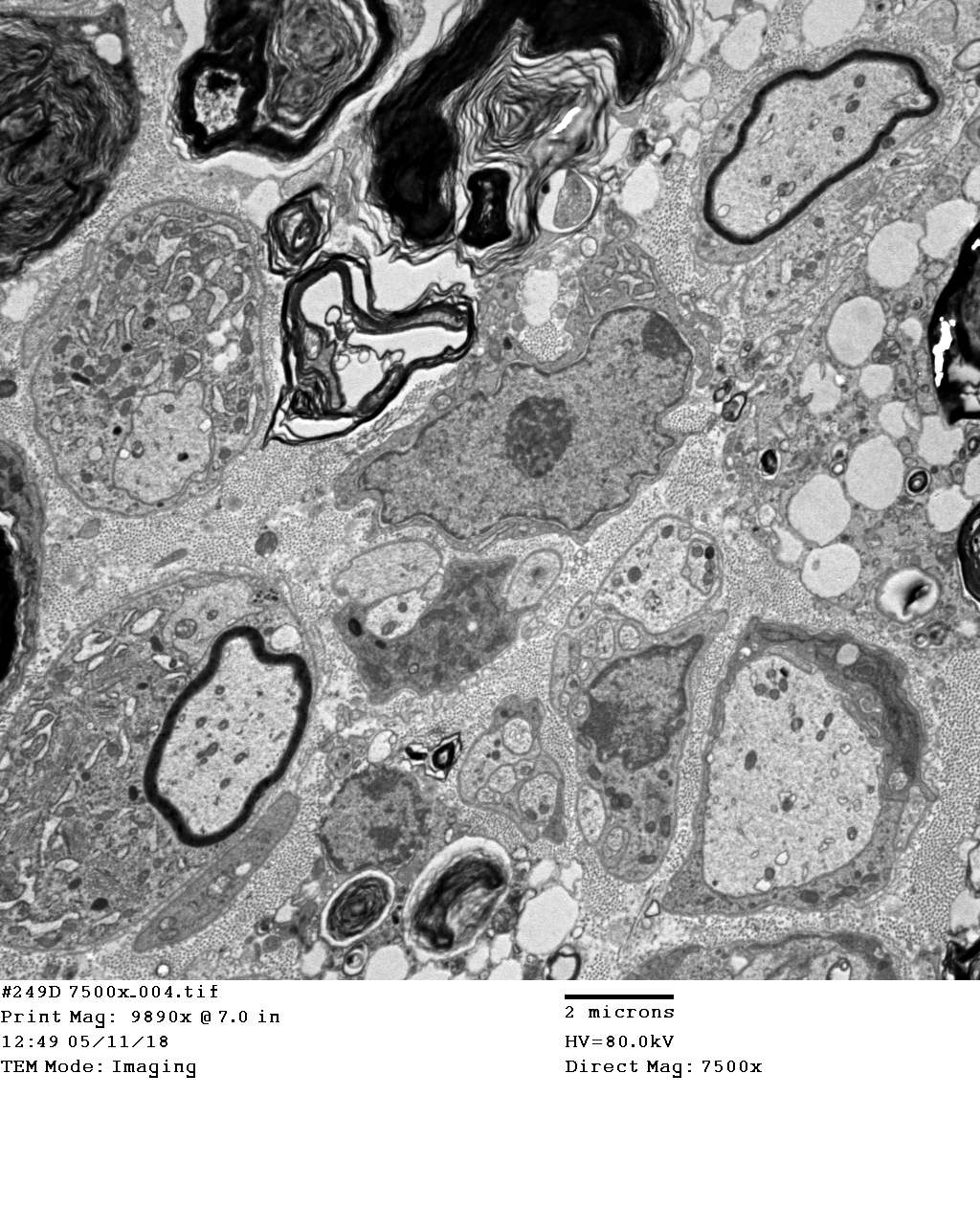

Supplement: Figure 8—source data 1. — This zip archive contains the TEM images for one WT and one Taz iKO used for quantitative analysis shown in Figure 8D–G. Images were taken using a JEOL 1010 electron microscope fitted with a Hamamatsu digital camera and AMT Advantage image capture software. Contrast of the images was adjusted using Photoshop software. [file elife-50138-fig8-data1.zip › Figure 8 source data 1/Taz iKO #249D 7500x/#249D 7500x_004 adjusted.tif]

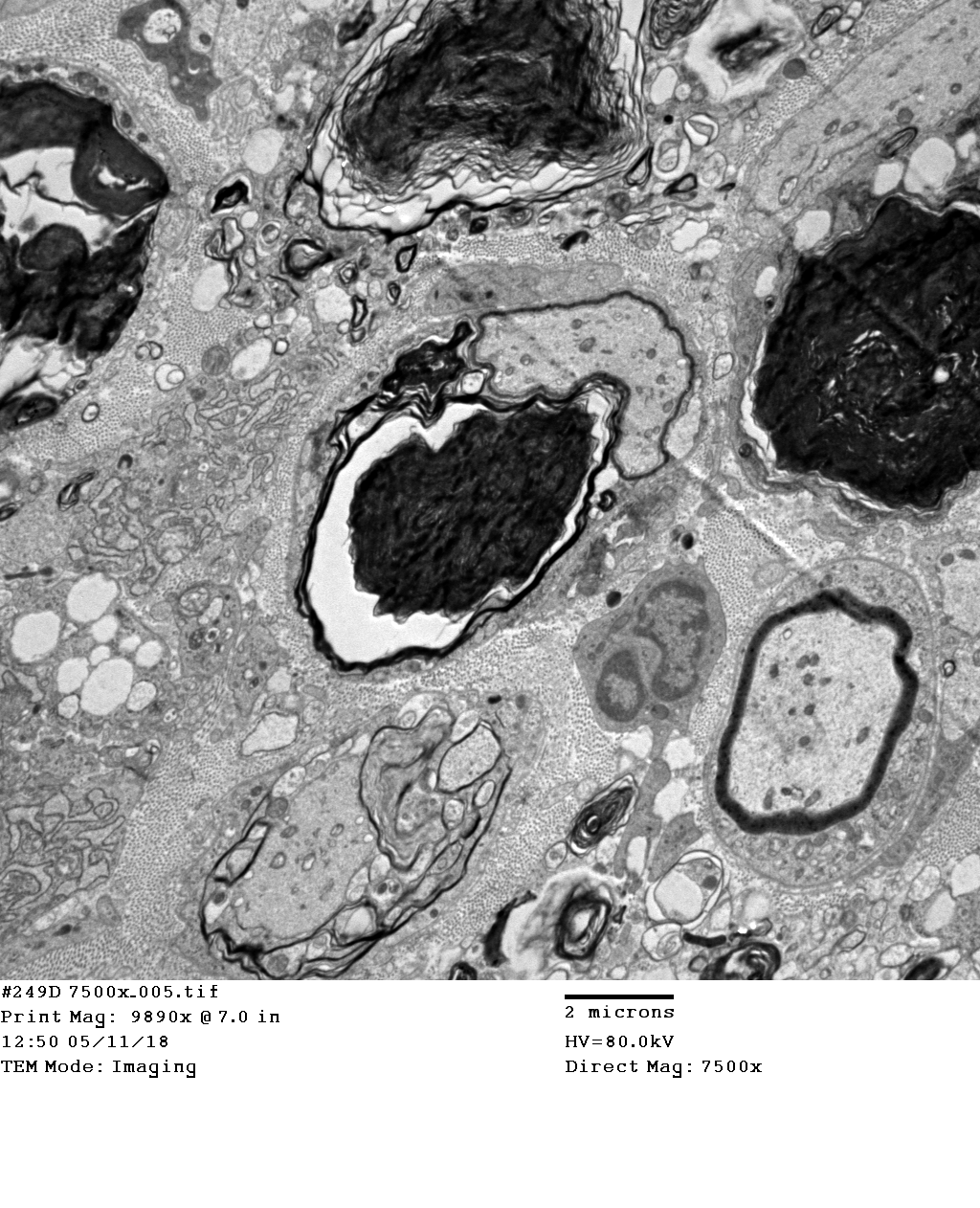

Supplement: Figure 8—source data 1. — This zip archive contains the TEM images for one WT and one Taz iKO used for quantitative analysis shown in Figure 8D–G. Images were taken using a JEOL 1010 electron microscope fitted with a Hamamatsu digital camera and AMT Advantage image capture software. Contrast of the images was adjusted using Photoshop software. [file elife-50138-fig8-data1.zip › Figure 8 source data 1/Taz iKO #249D 7500x/#249D 7500x_005 adjusted.tif]

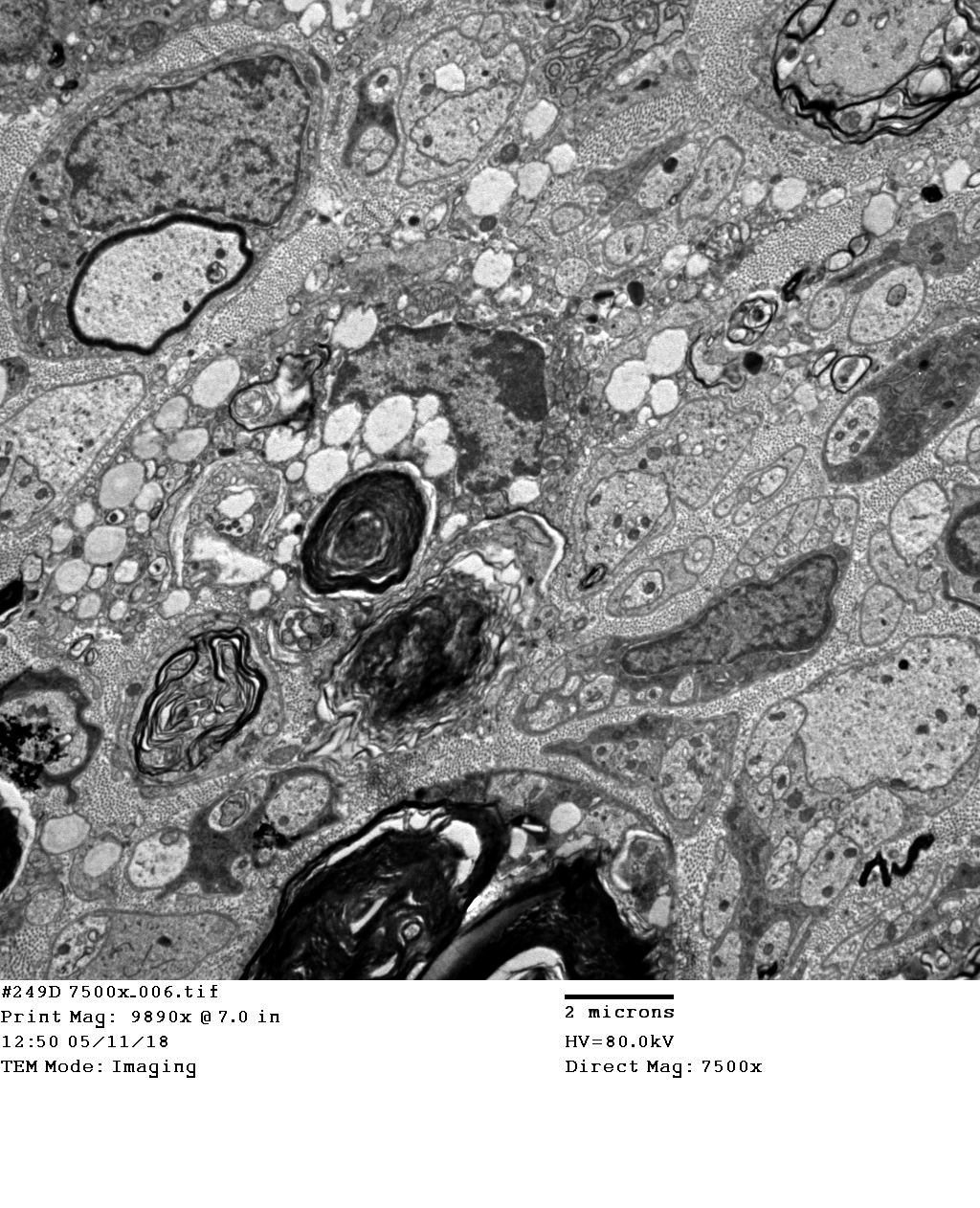

Supplement: Figure 8—source data 1. — This zip archive contains the TEM images for one WT and one Taz iKO used for quantitative analysis shown in Figure 8D–G. Images were taken using a JEOL 1010 electron microscope fitted with a Hamamatsu digital camera and AMT Advantage image capture software. Contrast of the images was adjusted using Photoshop software. [file elife-50138-fig8-data1.zip › Figure 8 source data 1/Taz iKO #249D 7500x/#249D 7500x_006 adjusted.tif]

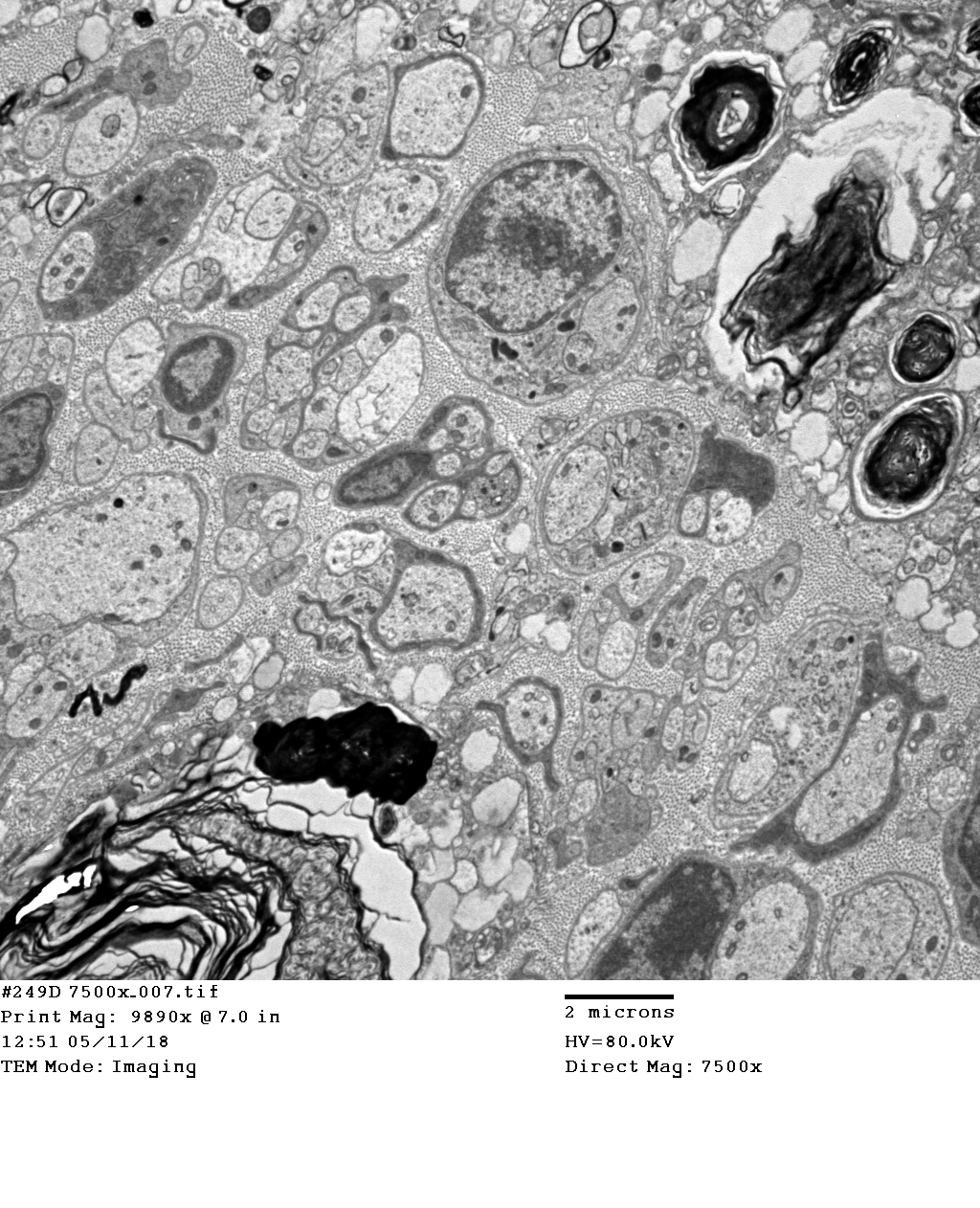

Supplement: Figure 8—source data 1. — This zip archive contains the TEM images for one WT and one Taz iKO used for quantitative analysis shown in Figure 8D–G. Images were taken using a JEOL 1010 electron microscope fitted with a Hamamatsu digital camera and AMT Advantage image capture software. Contrast of the images was adjusted using Photoshop software. [file elife-50138-fig8-data1.zip › Figure 8 source data 1/Taz iKO #249D 7500x/#249D 7500x_007 adjusted.tif]

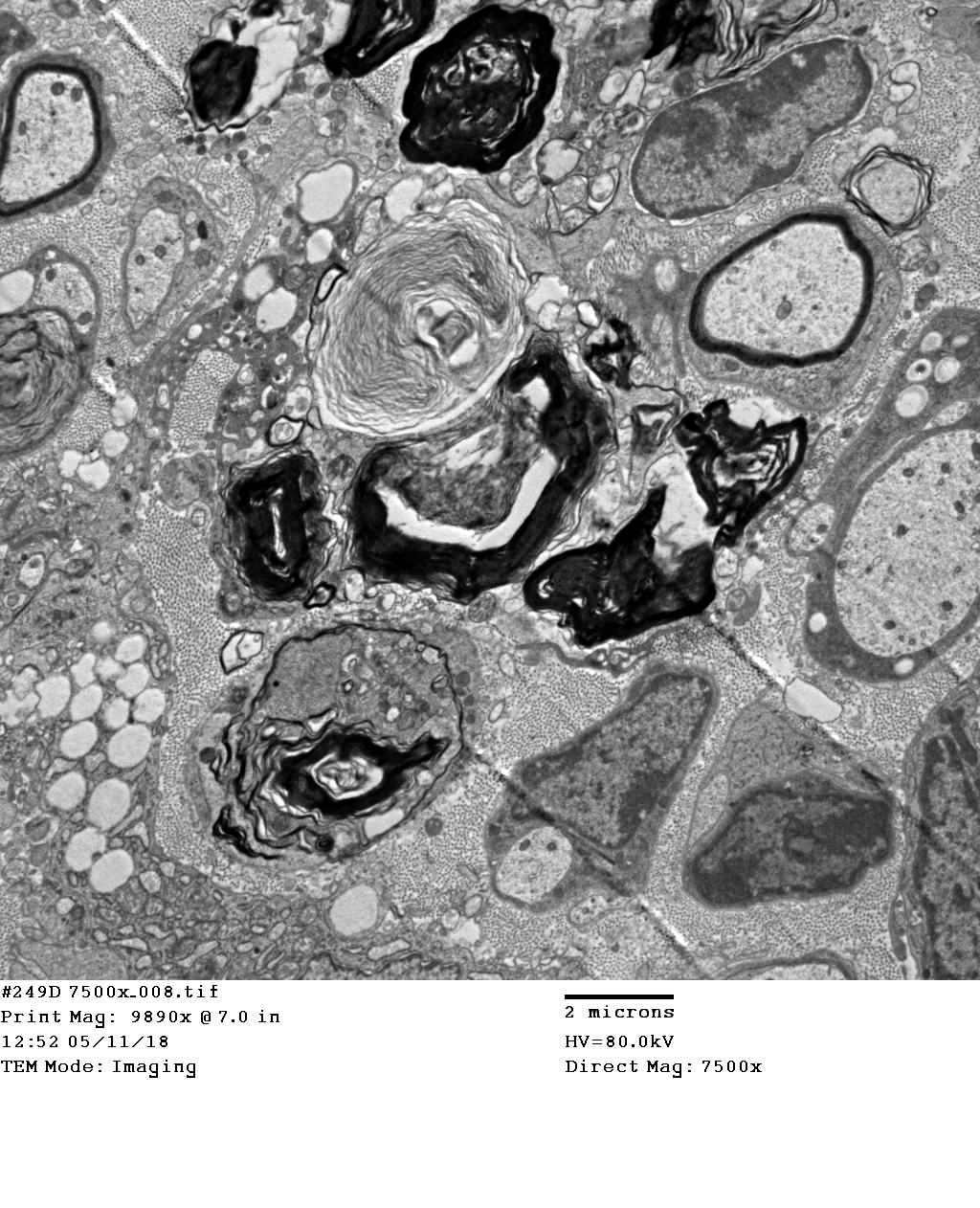

Supplement: Figure 8—source data 1. — This zip archive contains the TEM images for one WT and one Taz iKO used for quantitative analysis shown in Figure 8D–G. Images were taken using a JEOL 1010 electron microscope fitted with a Hamamatsu digital camera and AMT Advantage image capture software. Contrast of the images was adjusted using Photoshop software. [file elife-50138-fig8-data1.zip › Figure 8 source data 1/Taz iKO #249D 7500x/#249D 7500x_008 adjusted.tif]

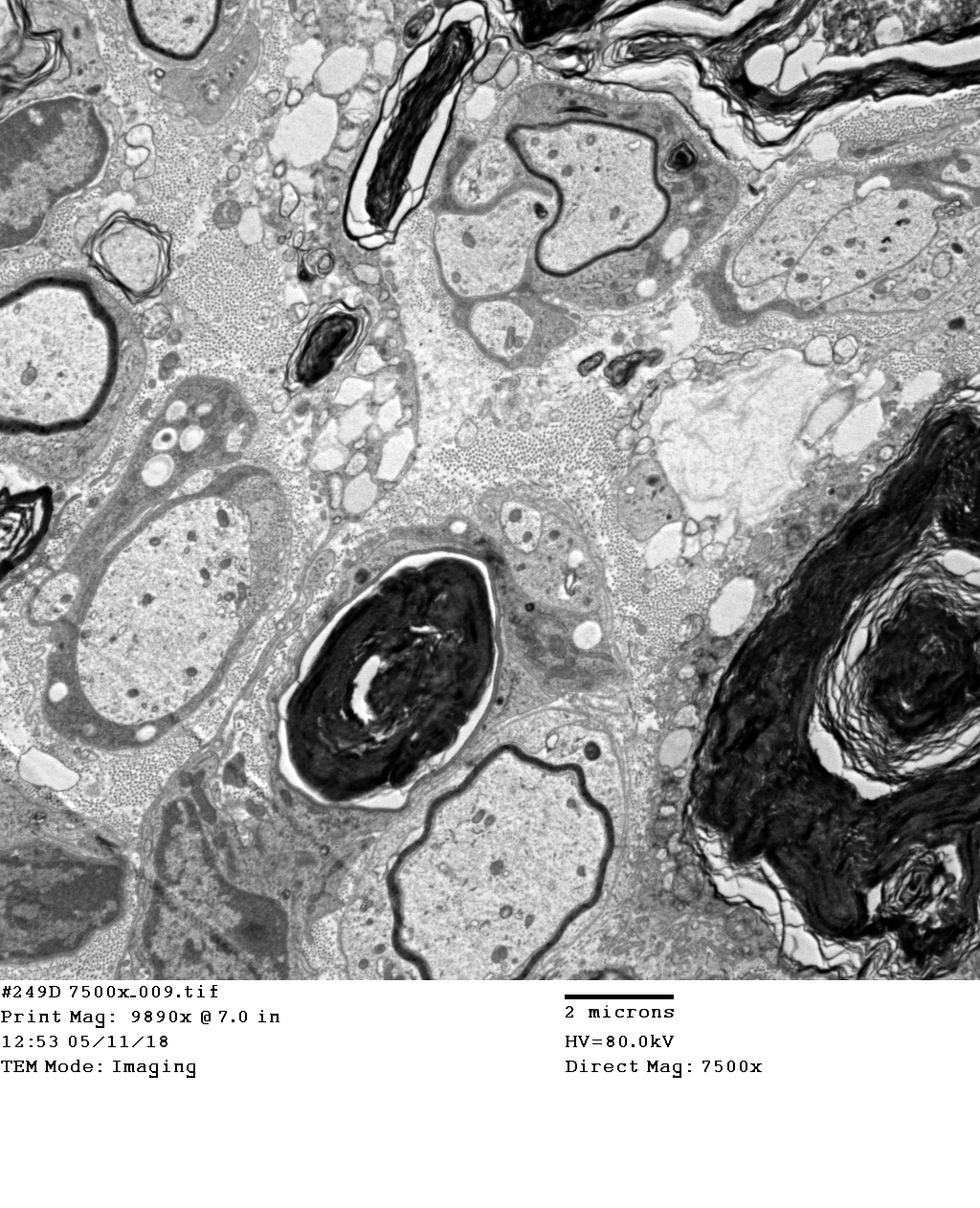

Supplement: Figure 8—source data 1. — This zip archive contains the TEM images for one WT and one Taz iKO used for quantitative analysis shown in Figure 8D–G. Images were taken using a JEOL 1010 electron microscope fitted with a Hamamatsu digital camera and AMT Advantage image capture software. Contrast of the images was adjusted using Photoshop software. [file elife-50138-fig8-data1.zip › Figure 8 source data 1/Taz iKO #249D 7500x/#249D 7500x_009 adjusted.tif]

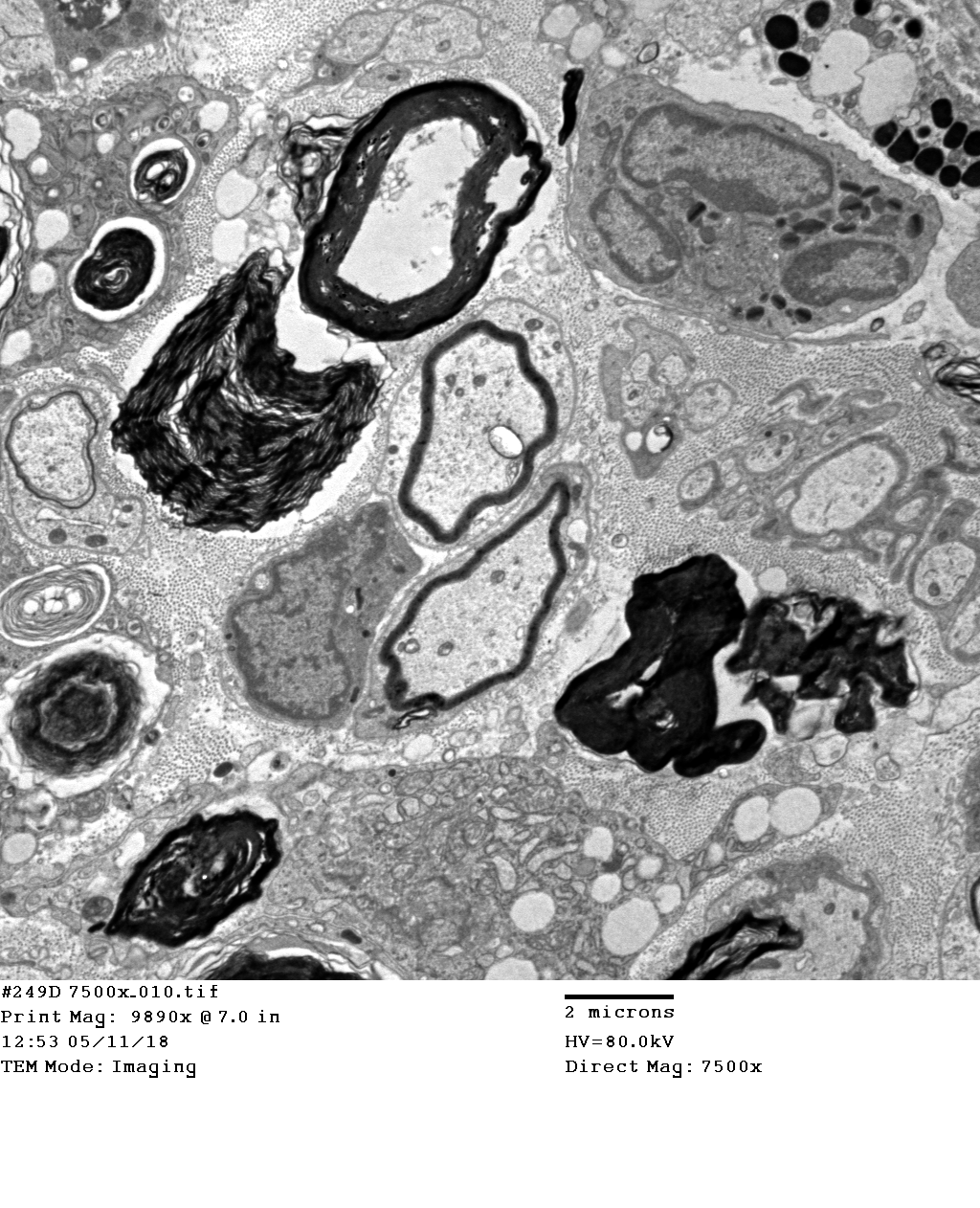

Supplement: Figure 8—source data 1. — This zip archive contains the TEM images for one WT and one Taz iKO used for quantitative analysis shown in Figure 8D–G. Images were taken using a JEOL 1010 electron microscope fitted with a Hamamatsu digital camera and AMT Advantage image capture software. Contrast of the images was adjusted using Photoshop software. [file elife-50138-fig8-data1.zip › Figure 8 source data 1/Taz iKO #249D 7500x/#249D 7500x_010 adjusted.tif]

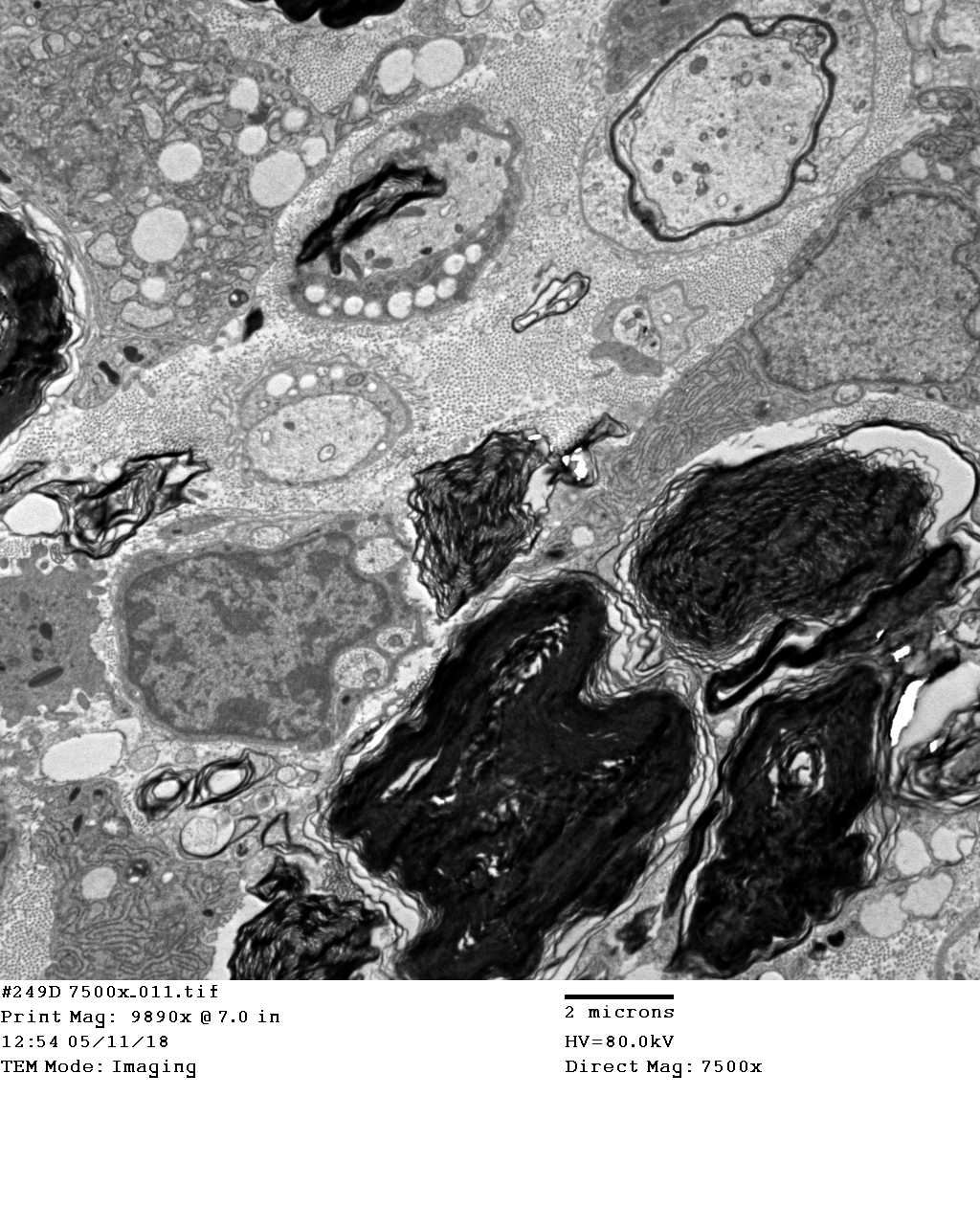

Supplement: Figure 8—source data 1. — This zip archive contains the TEM images for one WT and one Taz iKO used for quantitative analysis shown in Figure 8D–G. Images were taken using a JEOL 1010 electron microscope fitted with a Hamamatsu digital camera and AMT Advantage image capture software. Contrast of the images was adjusted using Photoshop software. [file elife-50138-fig8-data1.zip › Figure 8 source data 1/Taz iKO #249D 7500x/#249D 7500x_011 adjusted.tif]

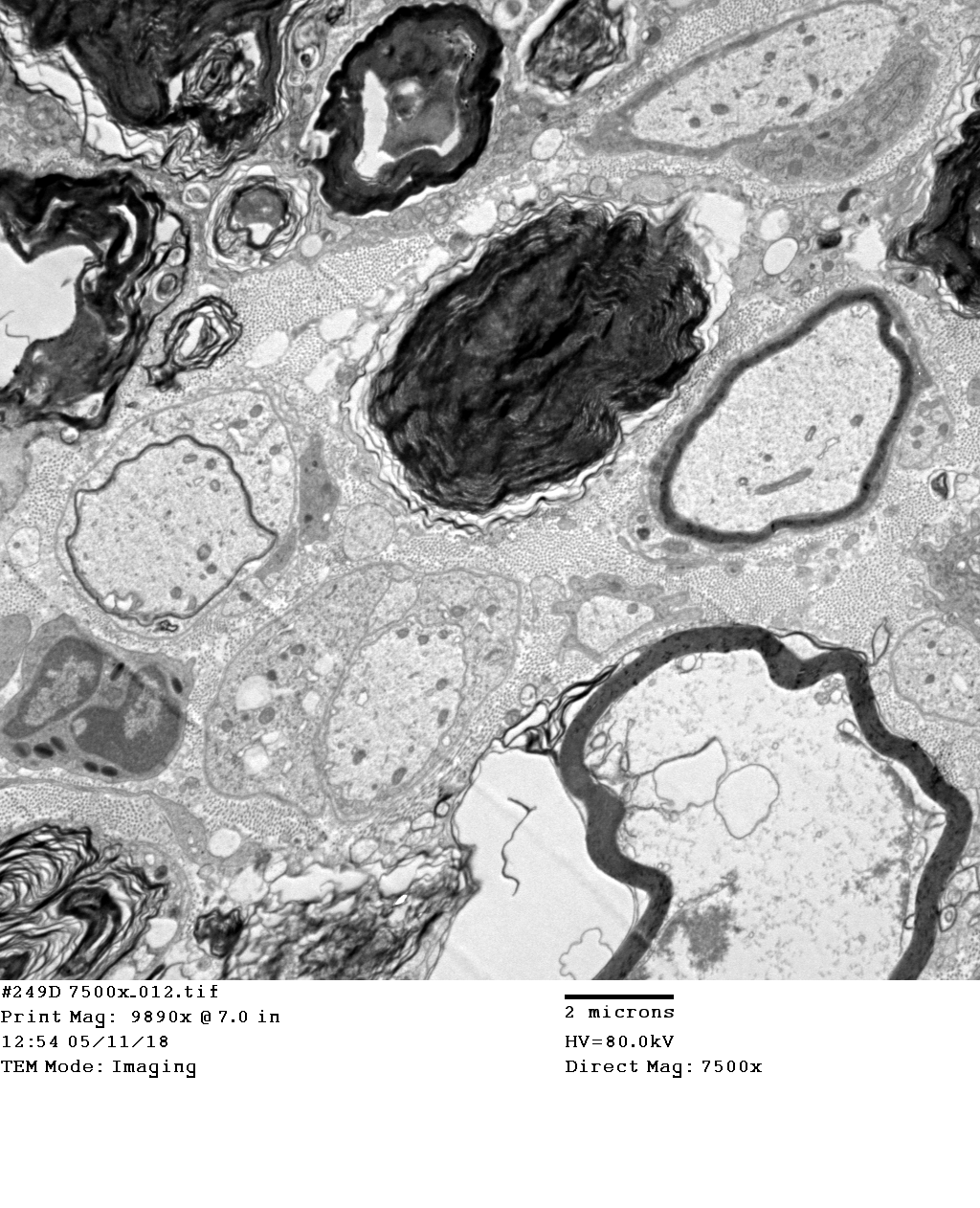

Supplement: Figure 8—source data 1. — This zip archive contains the TEM images for one WT and one Taz iKO used for quantitative analysis shown in Figure 8D–G. Images were taken using a JEOL 1010 electron microscope fitted with a Hamamatsu digital camera and AMT Advantage image capture software. Contrast of the images was adjusted using Photoshop software. [file elife-50138-fig8-data1.zip › Figure 8 source data 1/Taz iKO #249D 7500x/#249D 7500x_012 adjusted.tif]

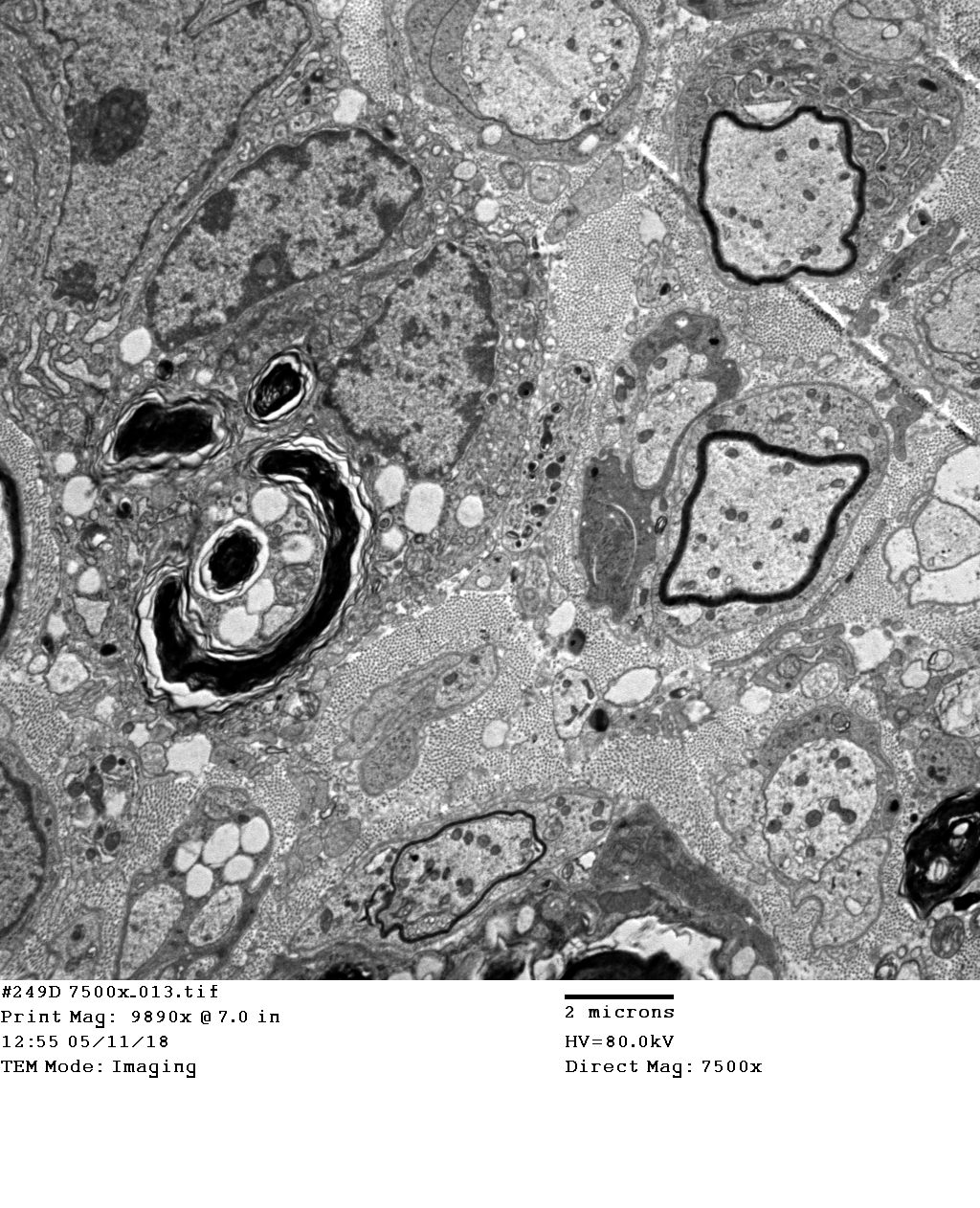

Supplement: Figure 8—source data 1. — This zip archive contains the TEM images for one WT and one Taz iKO used for quantitative analysis shown in Figure 8D–G. Images were taken using a JEOL 1010 electron microscope fitted with a Hamamatsu digital camera and AMT Advantage image capture software. Contrast of the images was adjusted using Photoshop software. [file elife-50138-fig8-data1.zip › Figure 8 source data 1/Taz iKO #249D 7500x/#249D 7500x_013 adjusted.tif]

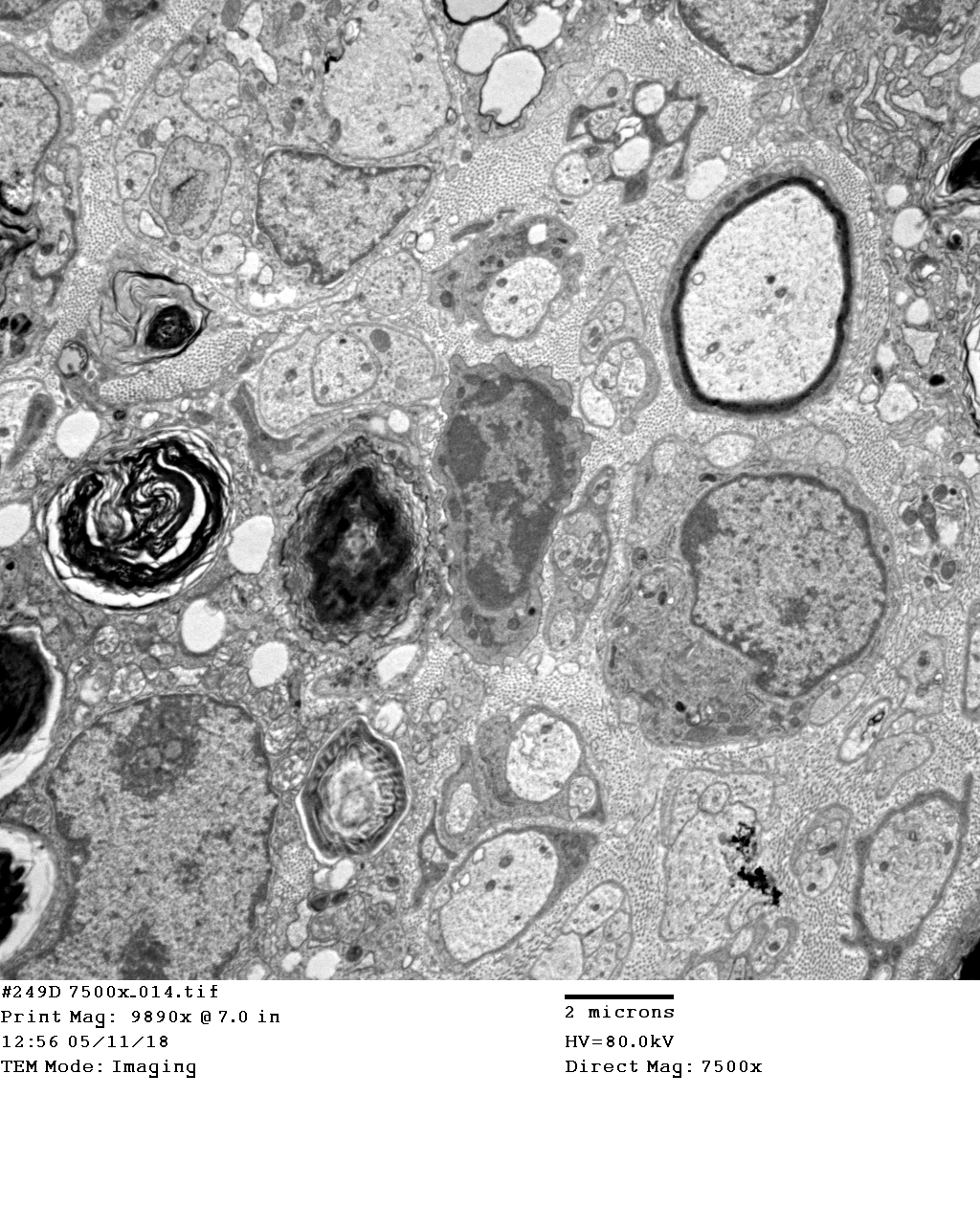

Supplement: Figure 8—source data 1. — This zip archive contains the TEM images for one WT and one Taz iKO used for quantitative analysis shown in Figure 8D–G. Images were taken using a JEOL 1010 electron microscope fitted with a Hamamatsu digital camera and AMT Advantage image capture software. Contrast of the images was adjusted using Photoshop software. [file elife-50138-fig8-data1.zip › Figure 8 source data 1/Taz iKO #249D 7500x/#249D 7500x_014 adjusted.tif]

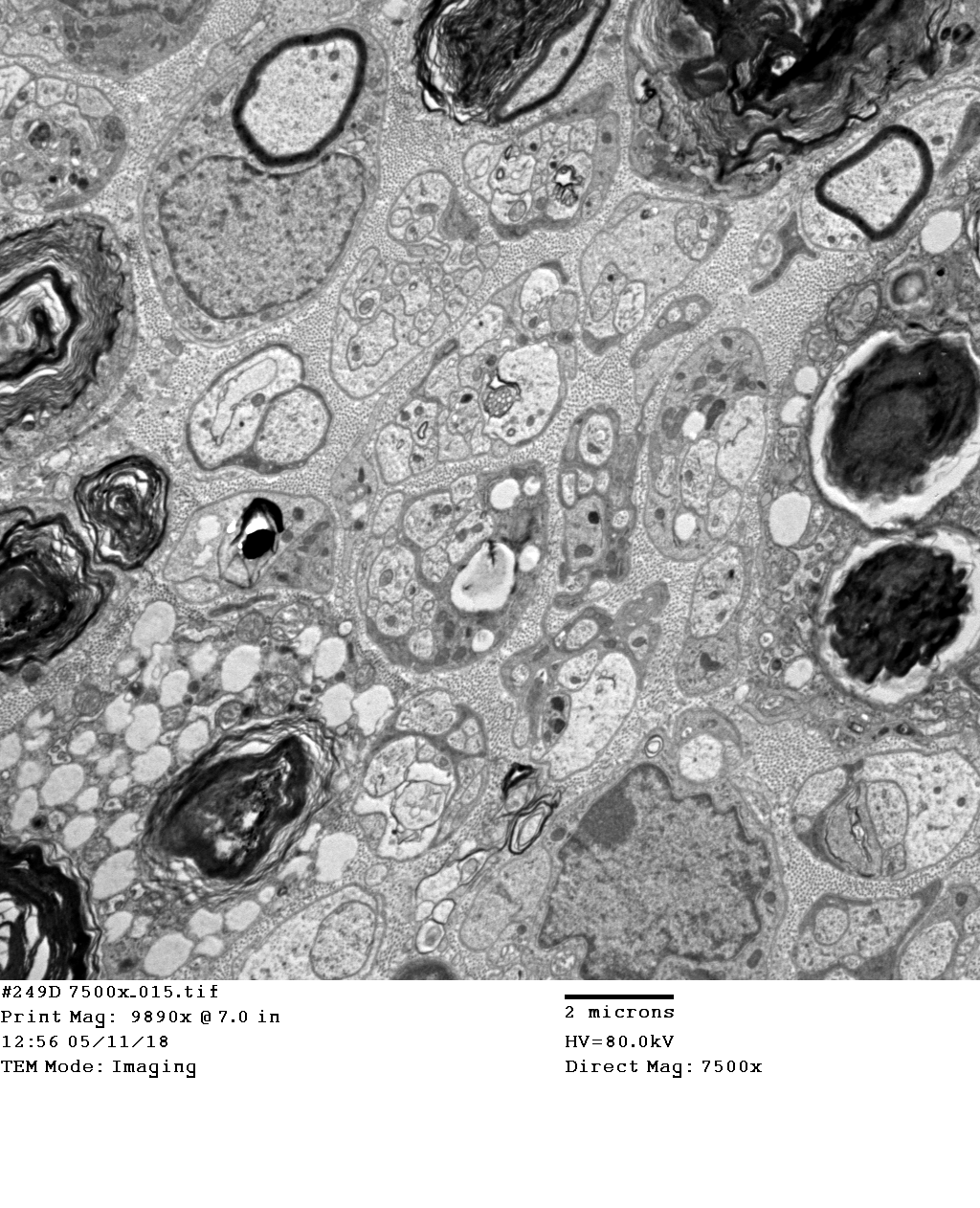

Supplement: Figure 8—source data 1. — This zip archive contains the TEM images for one WT and one Taz iKO used for quantitative analysis shown in Figure 8D–G. Images were taken using a JEOL 1010 electron microscope fitted with a Hamamatsu digital camera and AMT Advantage image capture software. Contrast of the images was adjusted using Photoshop software. [file elife-50138-fig8-data1.zip › Figure 8 source data 1/Taz iKO #249D 7500x/#249D 7500x_015 adjusted.tif]

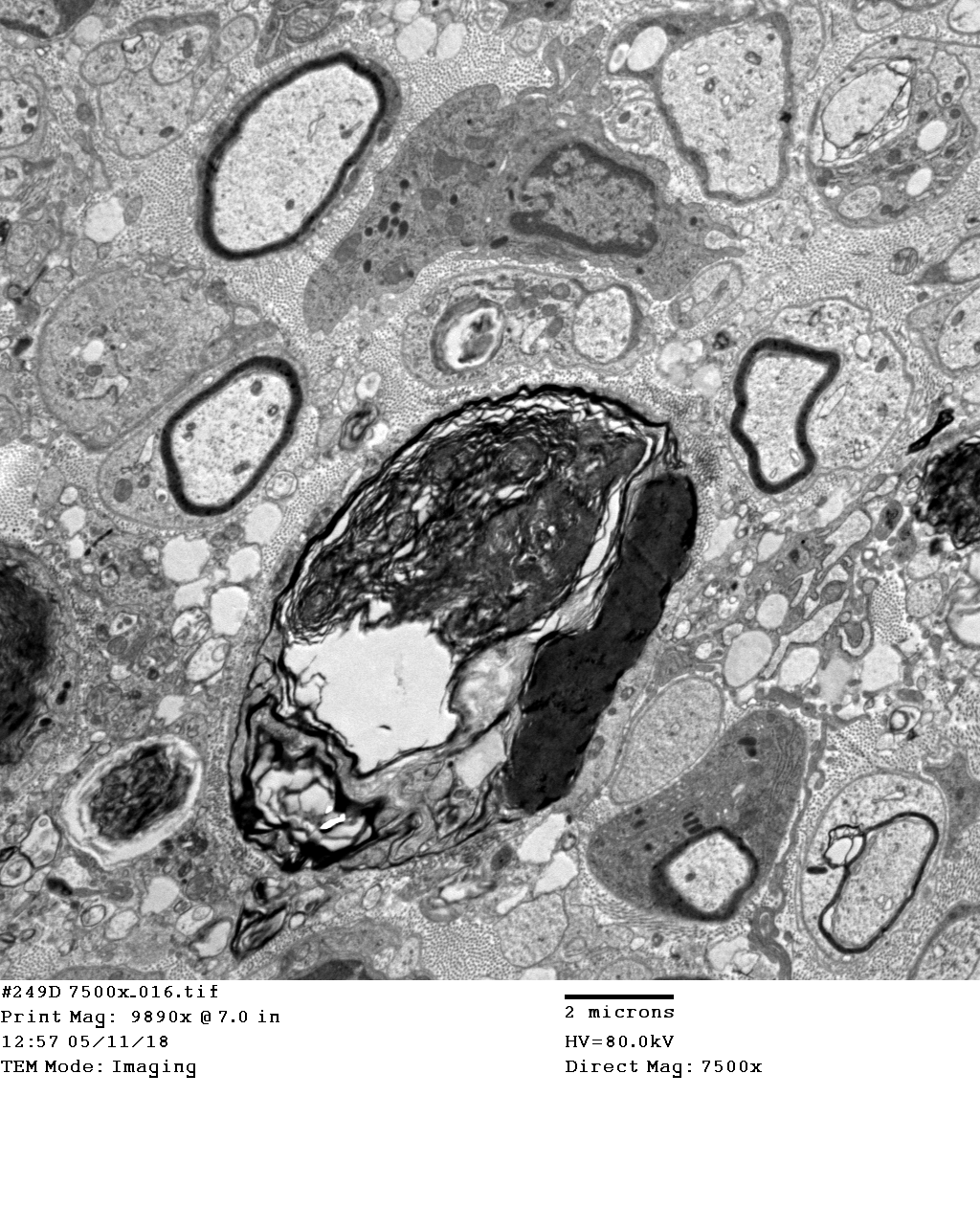

Supplement: Figure 8—source data 1. — This zip archive contains the TEM images for one WT and one Taz iKO used for quantitative analysis shown in Figure 8D–G. Images were taken using a JEOL 1010 electron microscope fitted with a Hamamatsu digital camera and AMT Advantage image capture software. Contrast of the images was adjusted using Photoshop software. [file elife-50138-fig8-data1.zip › Figure 8 source data 1/Taz iKO #249D 7500x/#249D 7500x_016 adjusted.tif]

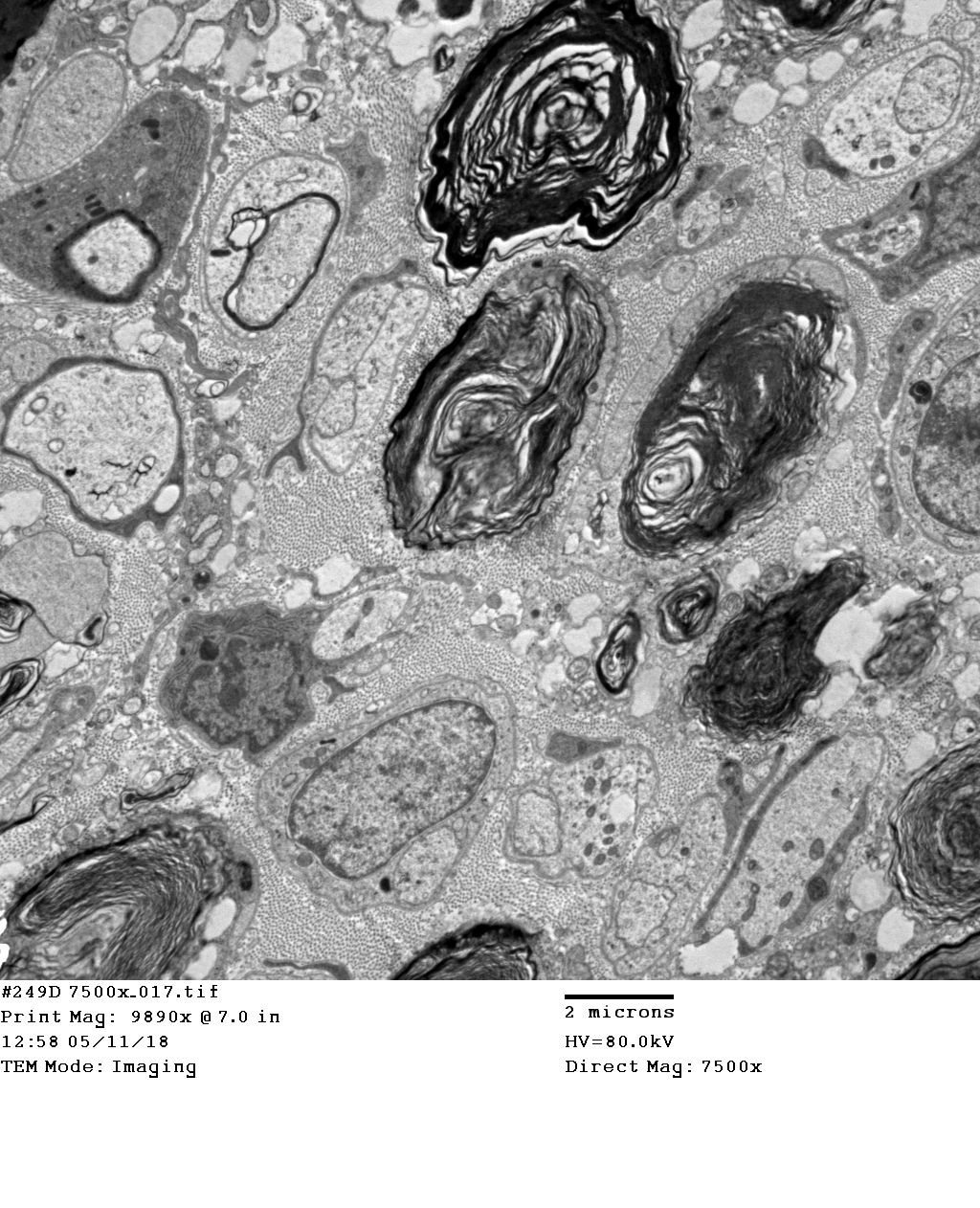

Supplement: Figure 8—source data 1. — This zip archive contains the TEM images for one WT and one Taz iKO used for quantitative analysis shown in Figure 8D–G. Images were taken using a JEOL 1010 electron microscope fitted with a Hamamatsu digital camera and AMT Advantage image capture software. Contrast of the images was adjusted using Photoshop software. [file elife-50138-fig8-data1.zip › Figure 8 source data 1/Taz iKO #249D 7500x/#249D 7500x_017 adjusted.tif]

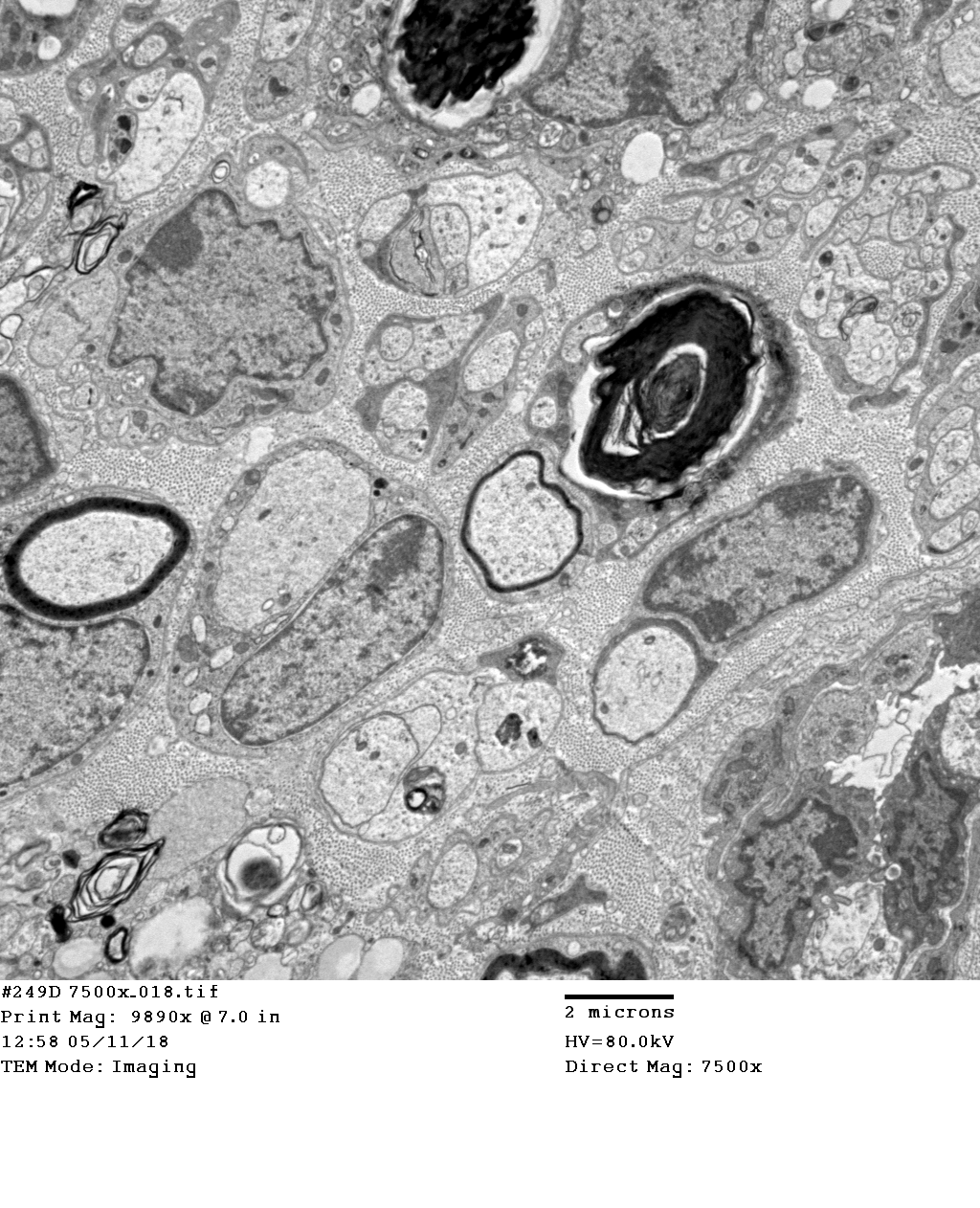

Supplement: Figure 8—source data 1. — This zip archive contains the TEM images for one WT and one Taz iKO used for quantitative analysis shown in Figure 8D–G. Images were taken using a JEOL 1010 electron microscope fitted with a Hamamatsu digital camera and AMT Advantage image capture software. Contrast of the images was adjusted using Photoshop software. [file elife-50138-fig8-data1.zip › Figure 8 source data 1/Taz iKO #249D 7500x/#249D 7500x_018 adjusted.tif]

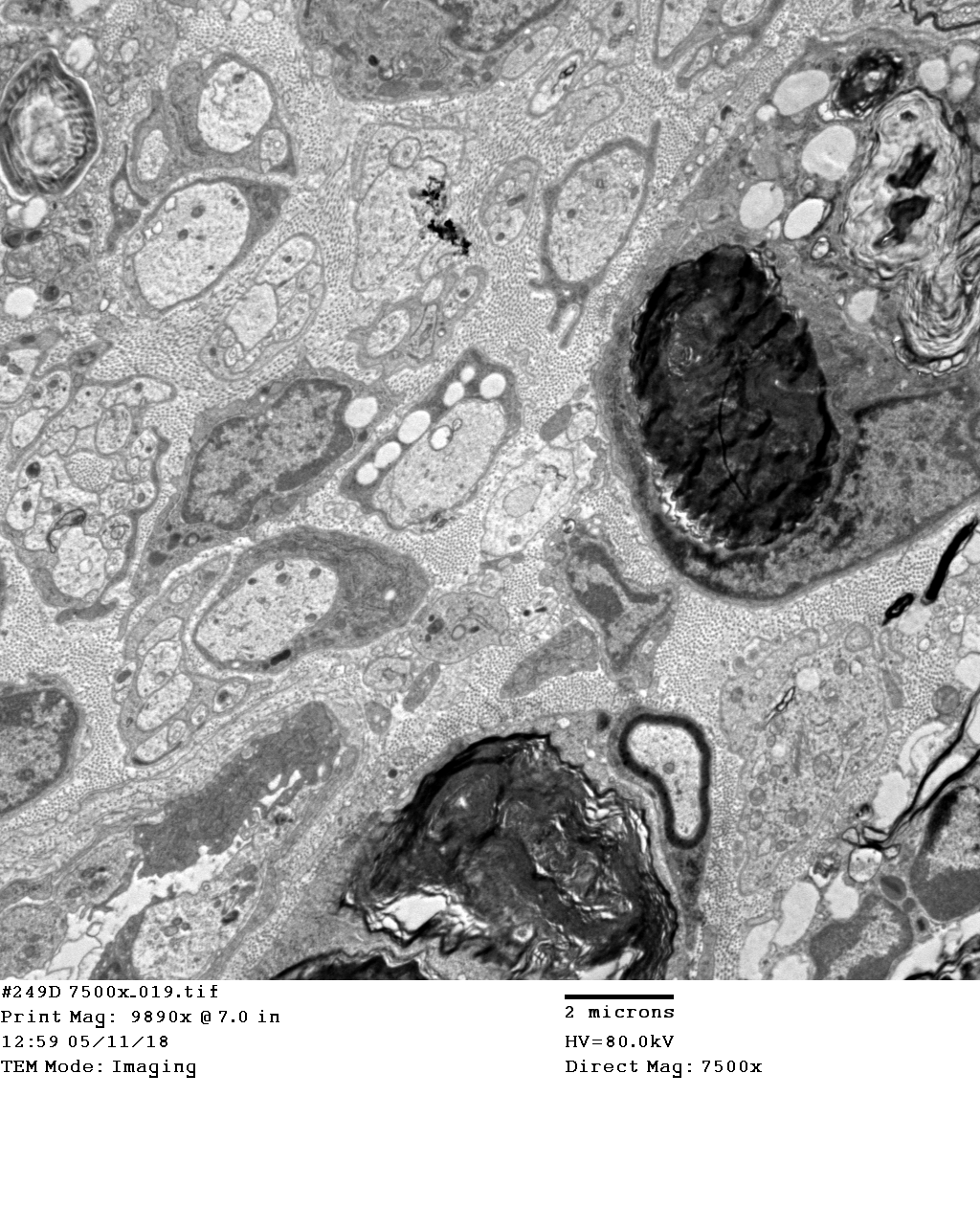

Supplement: Figure 8—source data 1. — This zip archive contains the TEM images for one WT and one Taz iKO used for quantitative analysis shown in Figure 8D–G. Images were taken using a JEOL 1010 electron microscope fitted with a Hamamatsu digital camera and AMT Advantage image capture software. Contrast of the images was adjusted using Photoshop software. [file elife-50138-fig8-data1.zip › Figure 8 source data 1/Taz iKO #249D 7500x/#249D 7500x_019 adjusted.tif]

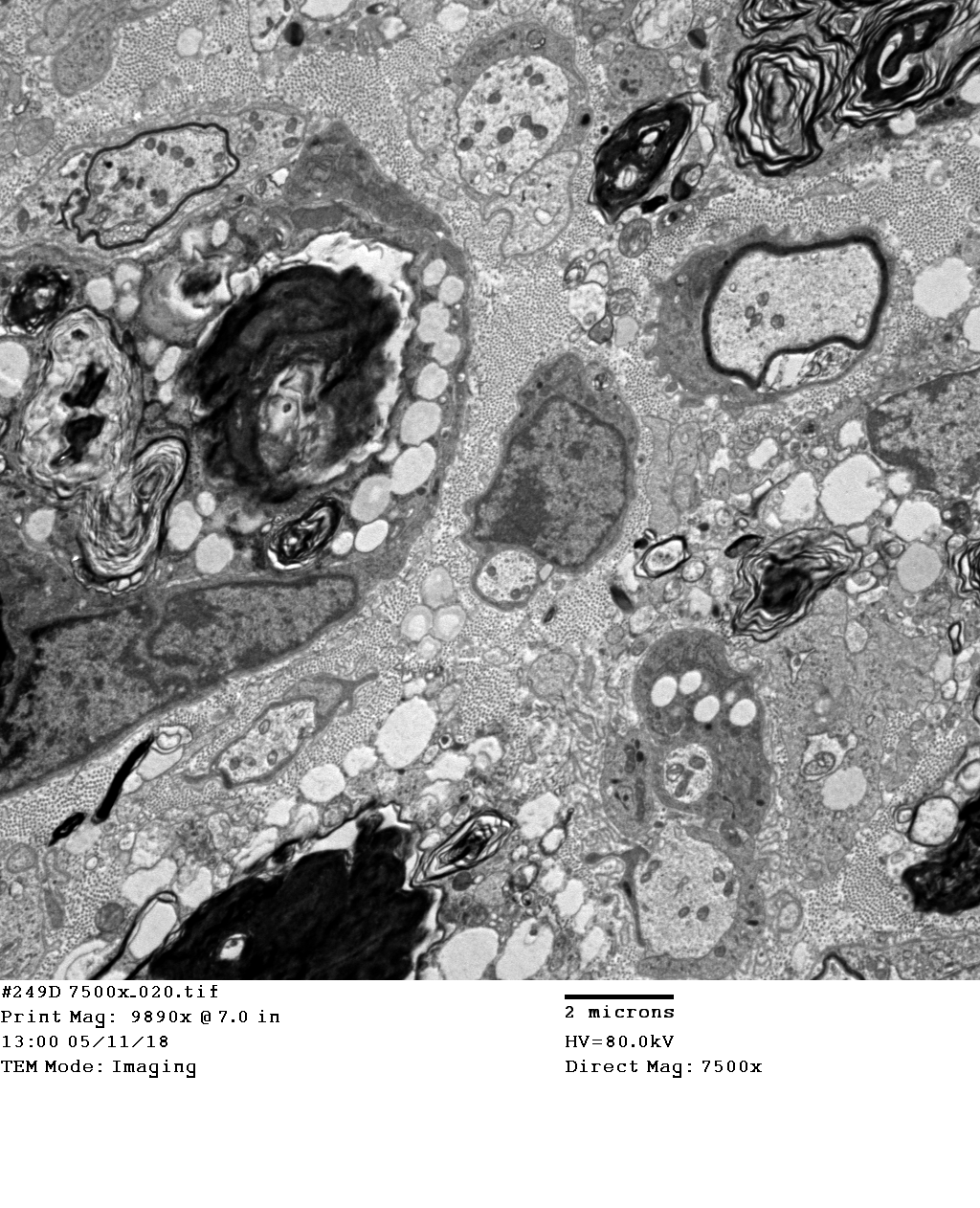

Supplement: Figure 8—source data 1. — This zip archive contains the TEM images for one WT and one Taz iKO used for quantitative analysis shown in Figure 8D–G. Images were taken using a JEOL 1010 electron microscope fitted with a Hamamatsu digital camera and AMT Advantage image capture software. Contrast of the images was adjusted using Photoshop software. [file elife-50138-fig8-data1.zip › Figure 8 source data 1/Taz iKO #249D 7500x/#249D 7500x_020 adjusted.tif]

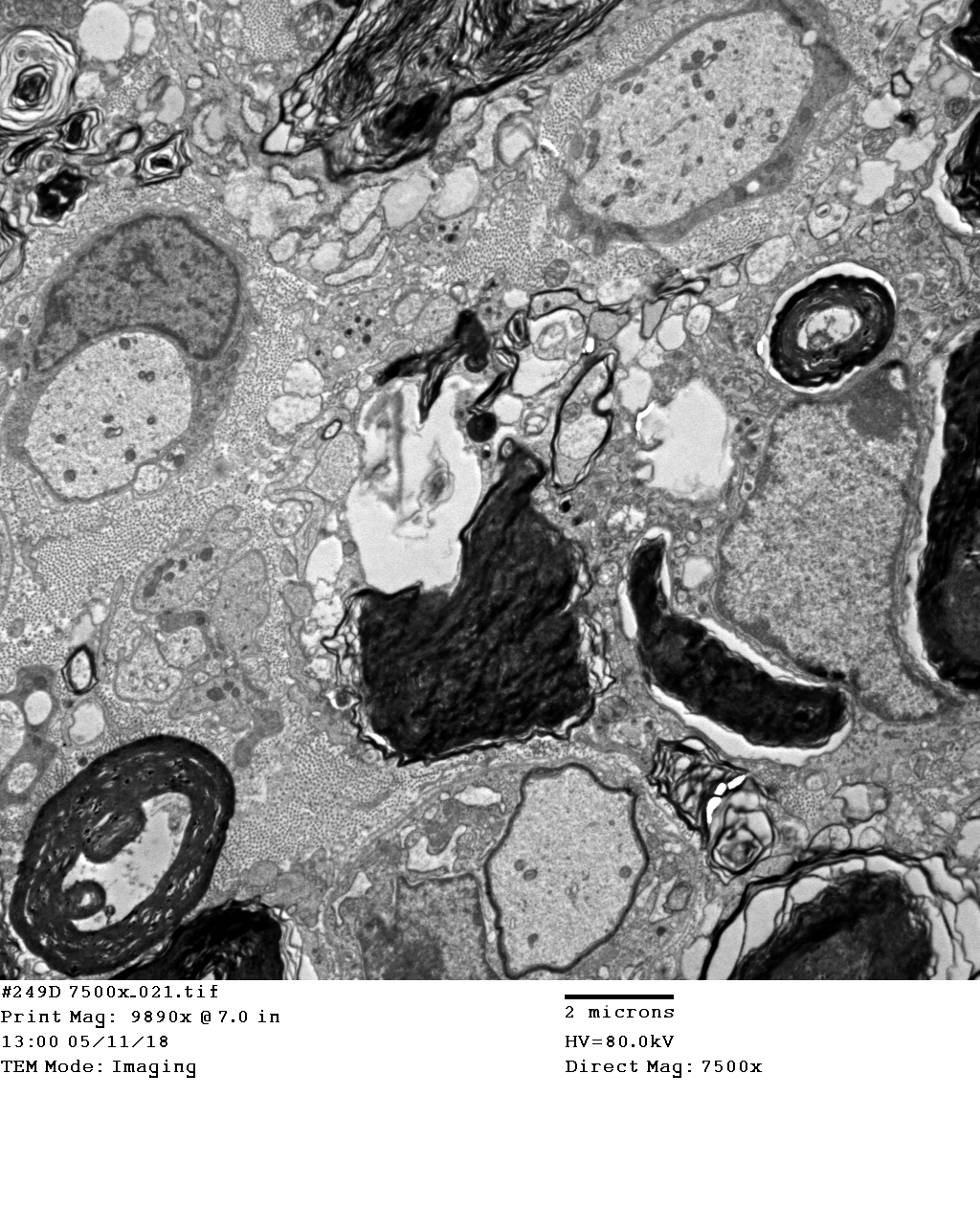

Supplement: Figure 8—source data 1. — This zip archive contains the TEM images for one WT and one Taz iKO used for quantitative analysis shown in Figure 8D–G. Images were taken using a JEOL 1010 electron microscope fitted with a Hamamatsu digital camera and AMT Advantage image capture software. Contrast of the images was adjusted using Photoshop software. [file elife-50138-fig8-data1.zip › Figure 8 source data 1/Taz iKO #249D 7500x/#249D 7500x_021 adjusted.tif]

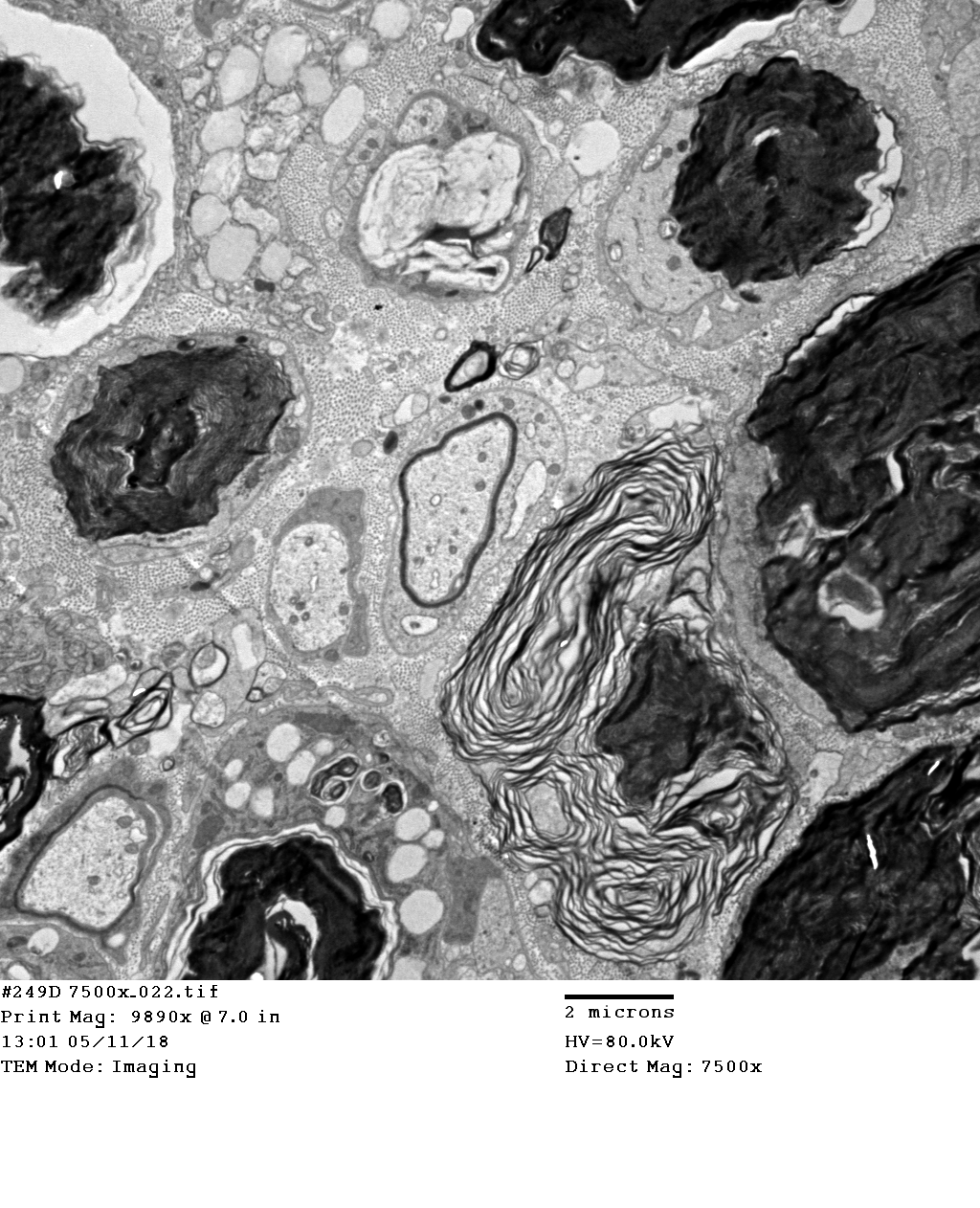

Supplement: Figure 8—source data 1. — This zip archive contains the TEM images for one WT and one Taz iKO used for quantitative analysis shown in Figure 8D–G. Images were taken using a JEOL 1010 electron microscope fitted with a Hamamatsu digital camera and AMT Advantage image capture software. Contrast of the images was adjusted using Photoshop software. [file elife-50138-fig8-data1.zip › Figure 8 source data 1/Taz iKO #249D 7500x/#249D 7500x_022 adjusted.tif]

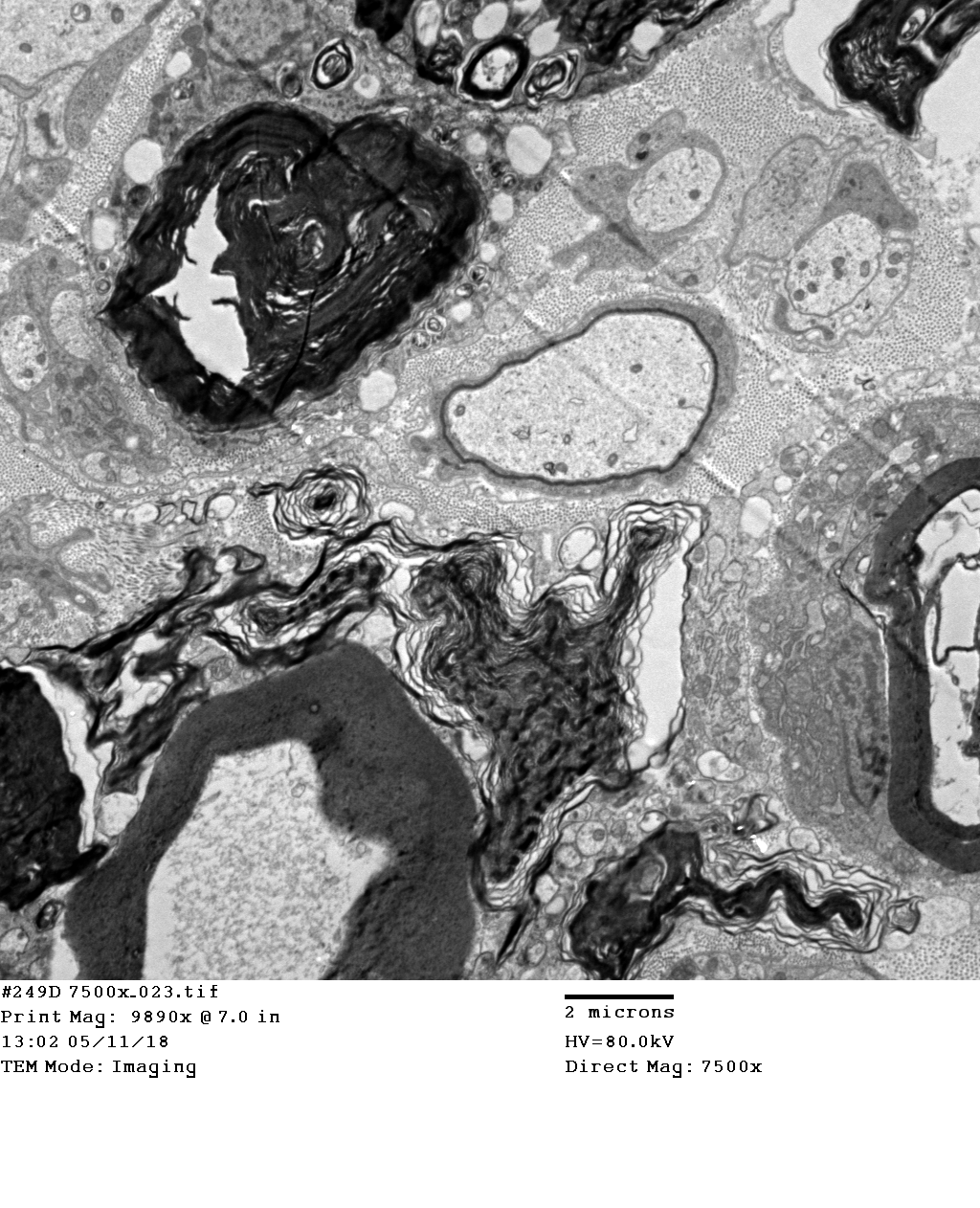

Supplement: Figure 8—source data 1. — This zip archive contains the TEM images for one WT and one Taz iKO used for quantitative analysis shown in Figure 8D–G. Images were taken using a JEOL 1010 electron microscope fitted with a Hamamatsu digital camera and AMT Advantage image capture software. Contrast of the images was adjusted using Photoshop software. [file elife-50138-fig8-data1.zip › Figure 8 source data 1/Taz iKO #249D 7500x/#249D 7500x_023 adjusted.tif]

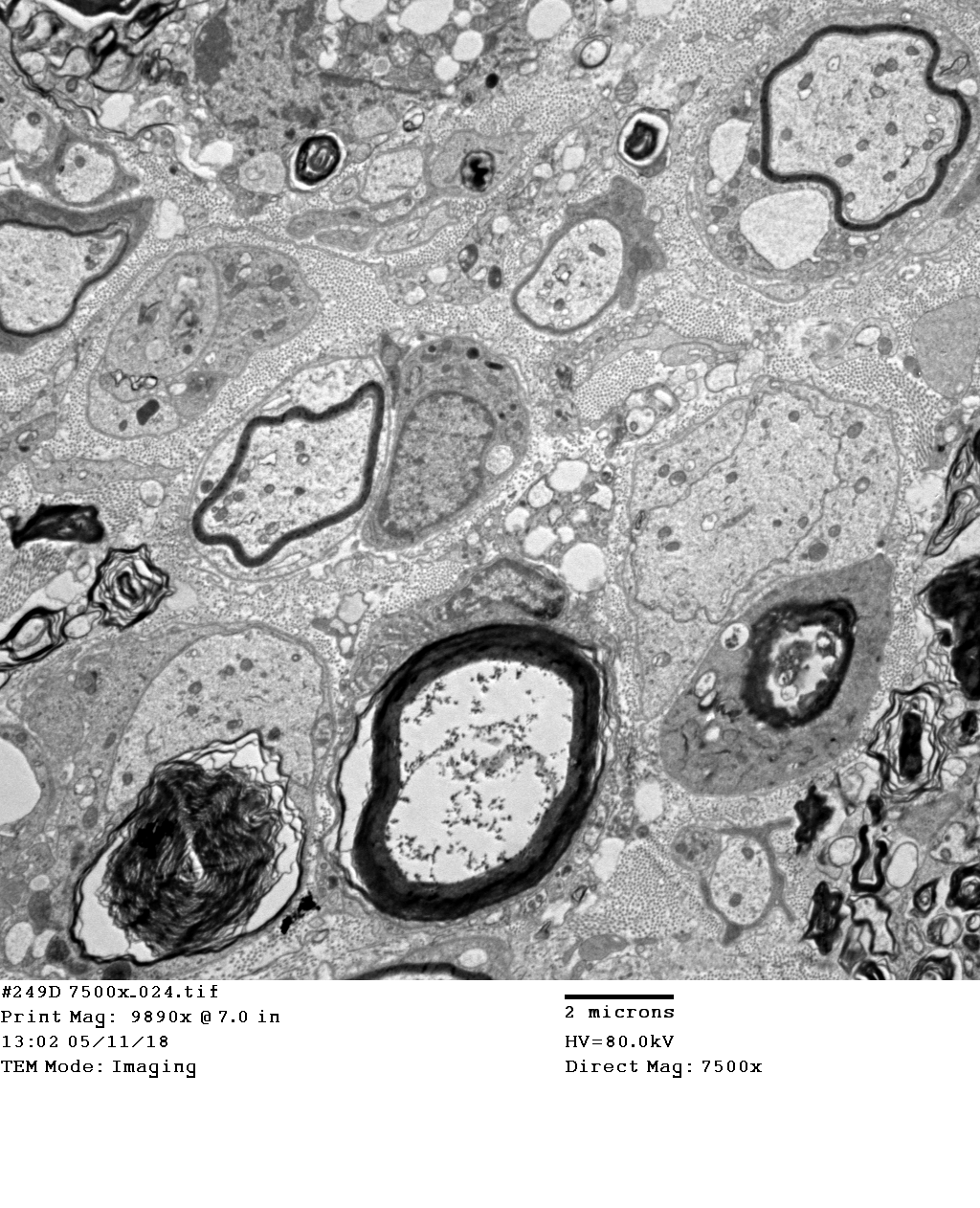

Supplement: Figure 8—source data 1. — This zip archive contains the TEM images for one WT and one Taz iKO used for quantitative analysis shown in Figure 8D–G. Images were taken using a JEOL 1010 electron microscope fitted with a Hamamatsu digital camera and AMT Advantage image capture software. Contrast of the images was adjusted using Photoshop software. [file elife-50138-fig8-data1.zip › Figure 8 source data 1/Taz iKO #249D 7500x/#249D 7500x_024 adjusted.tif]

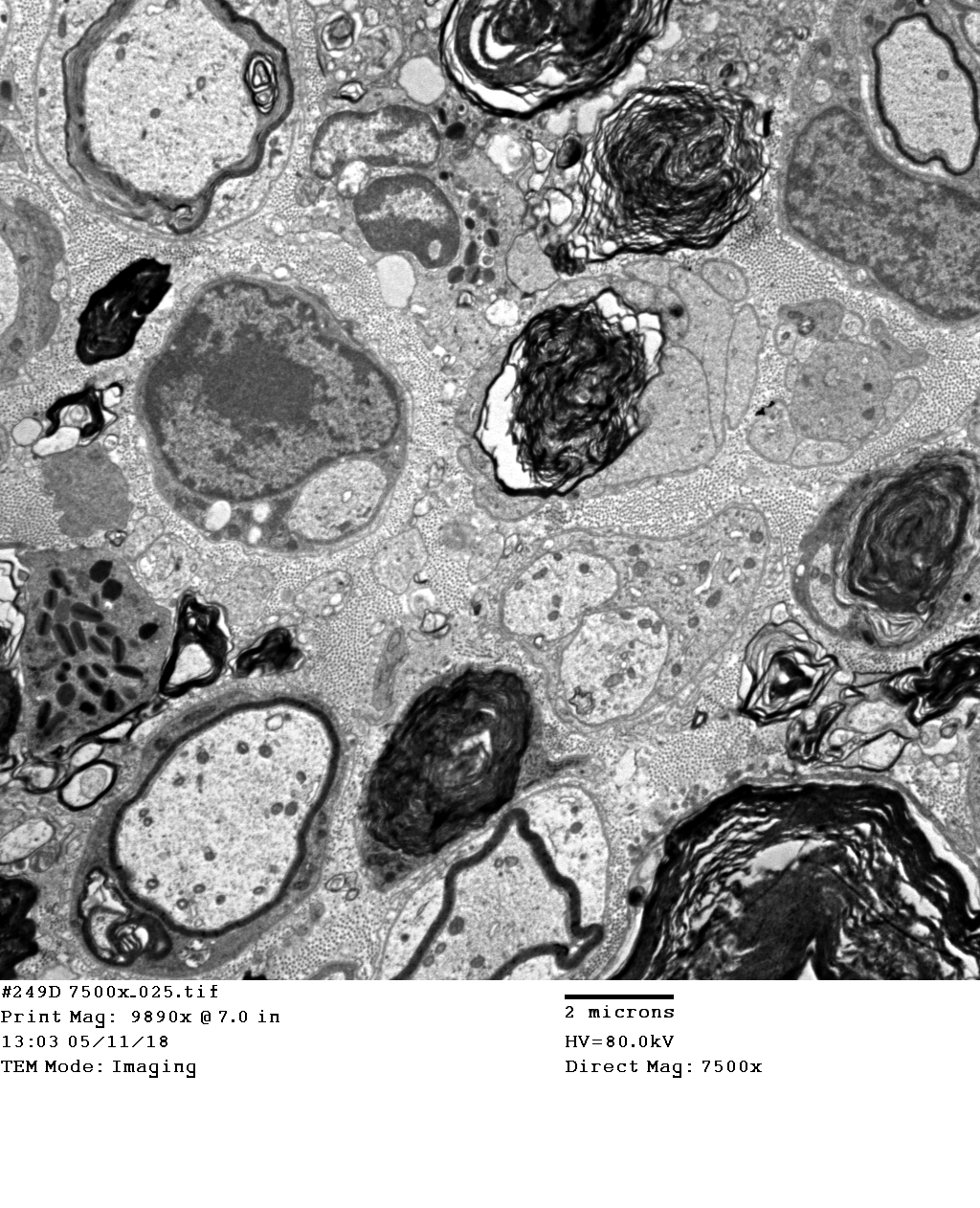

Supplement: Figure 8—source data 1. — This zip archive contains the TEM images for one WT and one Taz iKO used for quantitative analysis shown in Figure 8D–G. Images were taken using a JEOL 1010 electron microscope fitted with a Hamamatsu digital camera and AMT Advantage image capture software. Contrast of the images was adjusted using Photoshop software. [file elife-50138-fig8-data1.zip › Figure 8 source data 1/Taz iKO #249D 7500x/#249D 7500x_025 adjusted.tif]

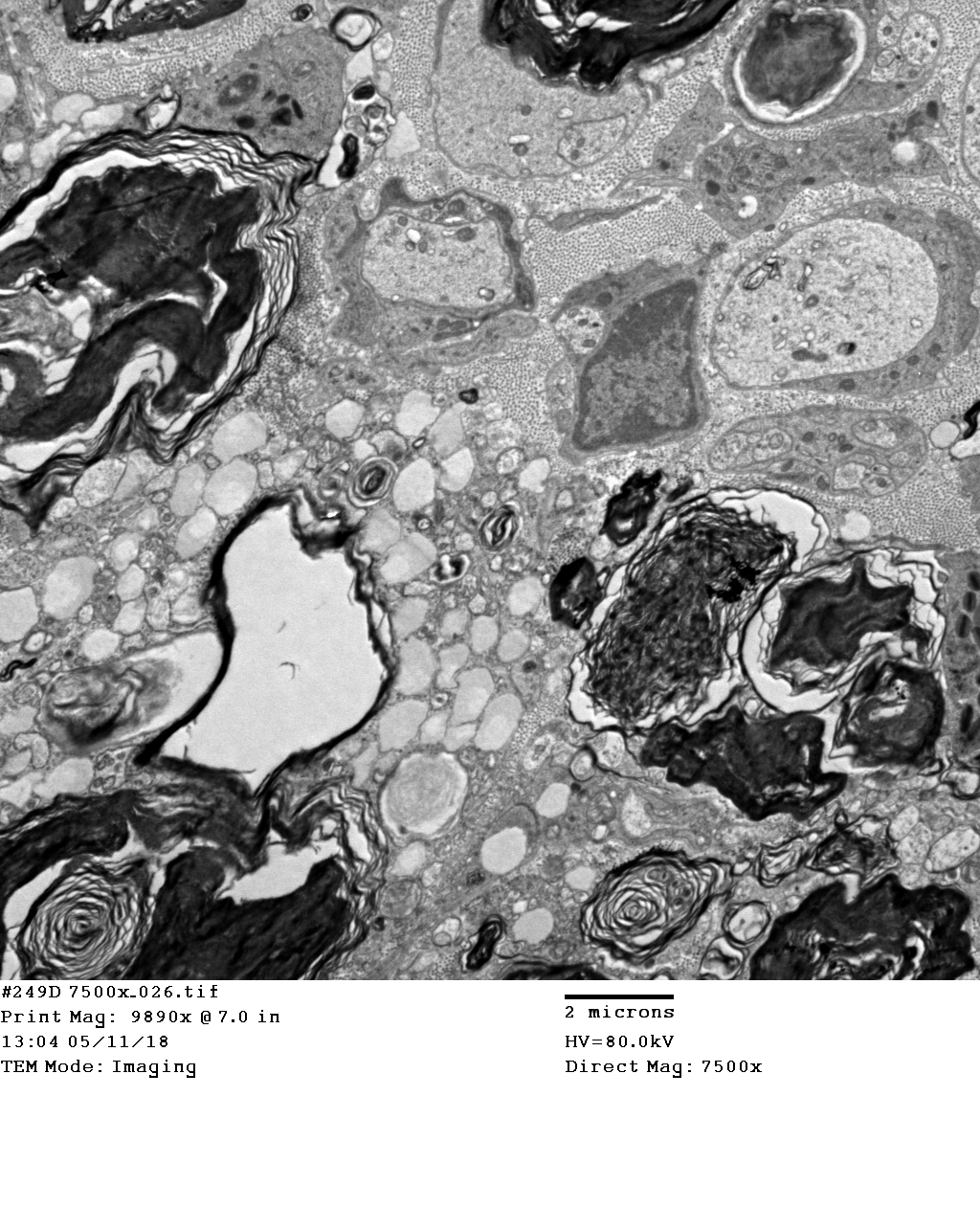

Supplement: Figure 8—source data 1. — This zip archive contains the TEM images for one WT and one Taz iKO used for quantitative analysis shown in Figure 8D–G. Images were taken using a JEOL 1010 electron microscope fitted with a Hamamatsu digital camera and AMT Advantage image capture software. Contrast of the images was adjusted using Photoshop software. [file elife-50138-fig8-data1.zip › Figure 8 source data 1/Taz iKO #249D 7500x/#249D 7500x_026 adjusted.tif]

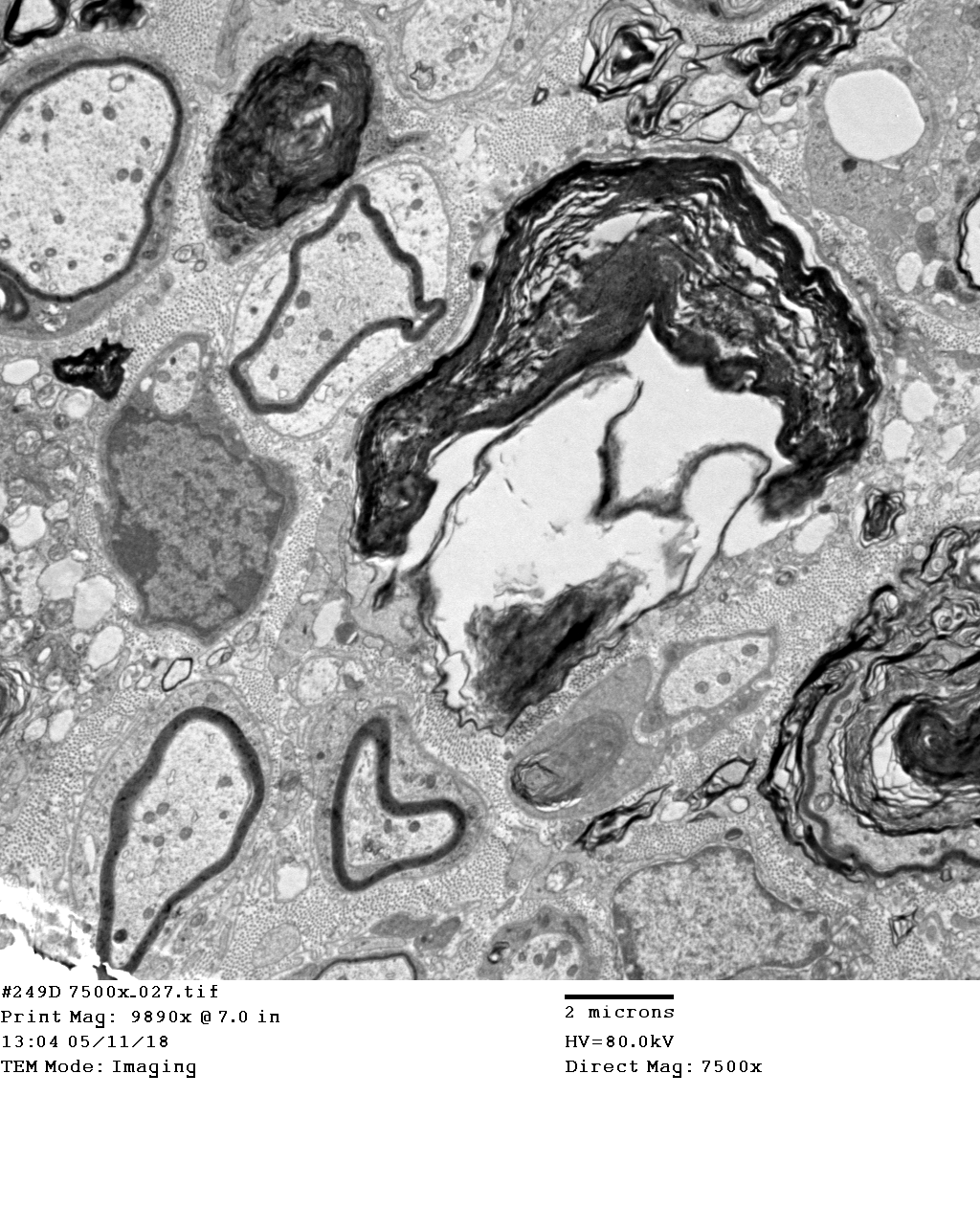

Supplement: Figure 8—source data 1. — This zip archive contains the TEM images for one WT and one Taz iKO used for quantitative analysis shown in Figure 8D–G. Images were taken using a JEOL 1010 electron microscope fitted with a Hamamatsu digital camera and AMT Advantage image capture software. Contrast of the images was adjusted using Photoshop software. [file elife-50138-fig8-data1.zip › Figure 8 source data 1/Taz iKO #249D 7500x/#249D 7500x_027 adjusted.tif]

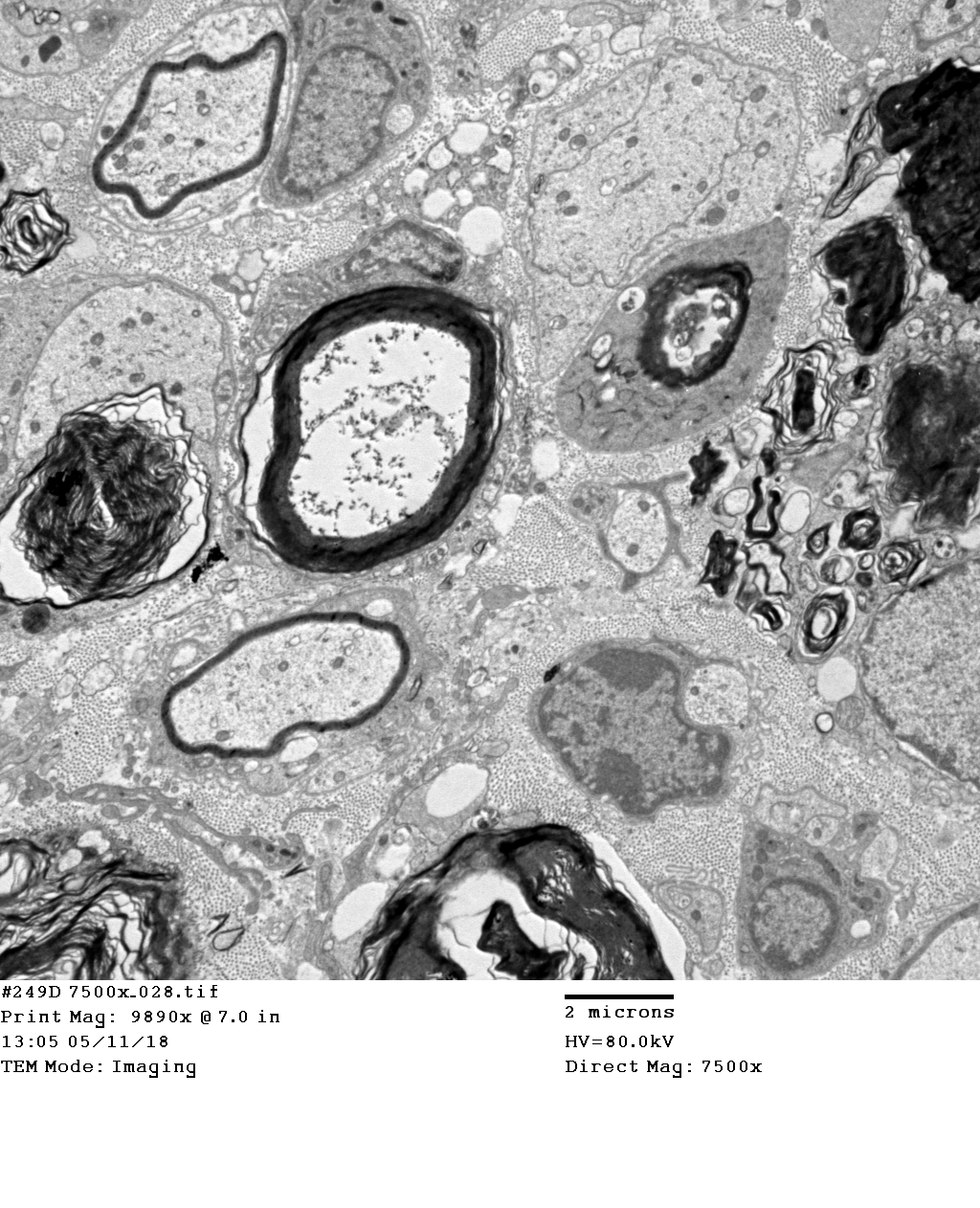

Supplement: Figure 8—source data 1. — This zip archive contains the TEM images for one WT and one Taz iKO used for quantitative analysis shown in Figure 8D–G. Images were taken using a JEOL 1010 electron microscope fitted with a Hamamatsu digital camera and AMT Advantage image capture software. Contrast of the images was adjusted using Photoshop software. [file elife-50138-fig8-data1.zip › Figure 8 source data 1/Taz iKO #249D 7500x/#249D 7500x_028 adjusted.tif]

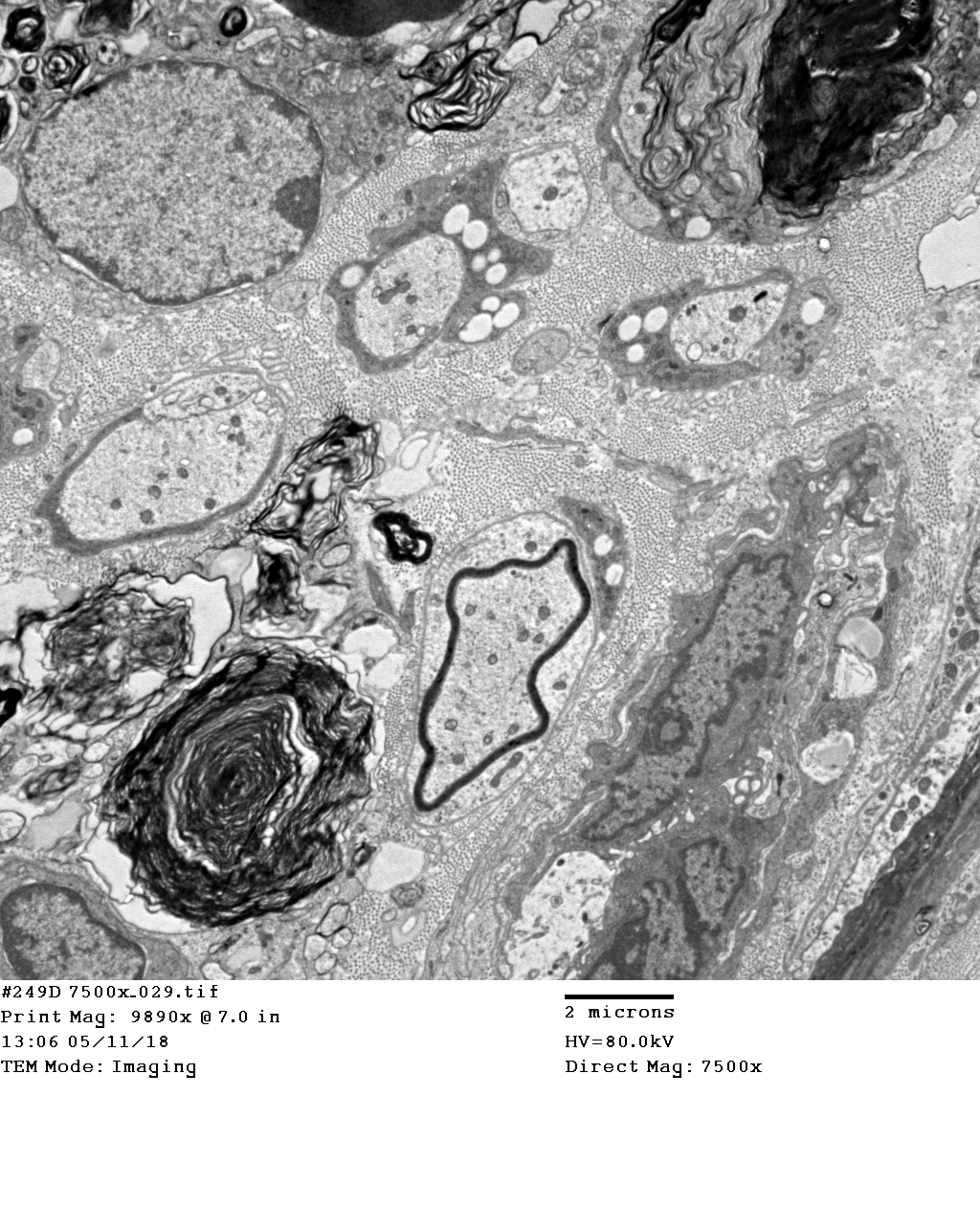

Supplement: Figure 8—source data 1. — This zip archive contains the TEM images for one WT and one Taz iKO used for quantitative analysis shown in Figure 8D–G. Images were taken using a JEOL 1010 electron microscope fitted with a Hamamatsu digital camera and AMT Advantage image capture software. Contrast of the images was adjusted using Photoshop software. [file elife-50138-fig8-data1.zip › Figure 8 source data 1/Taz iKO #249D 7500x/#249D 7500x_029 adjusted.tif]

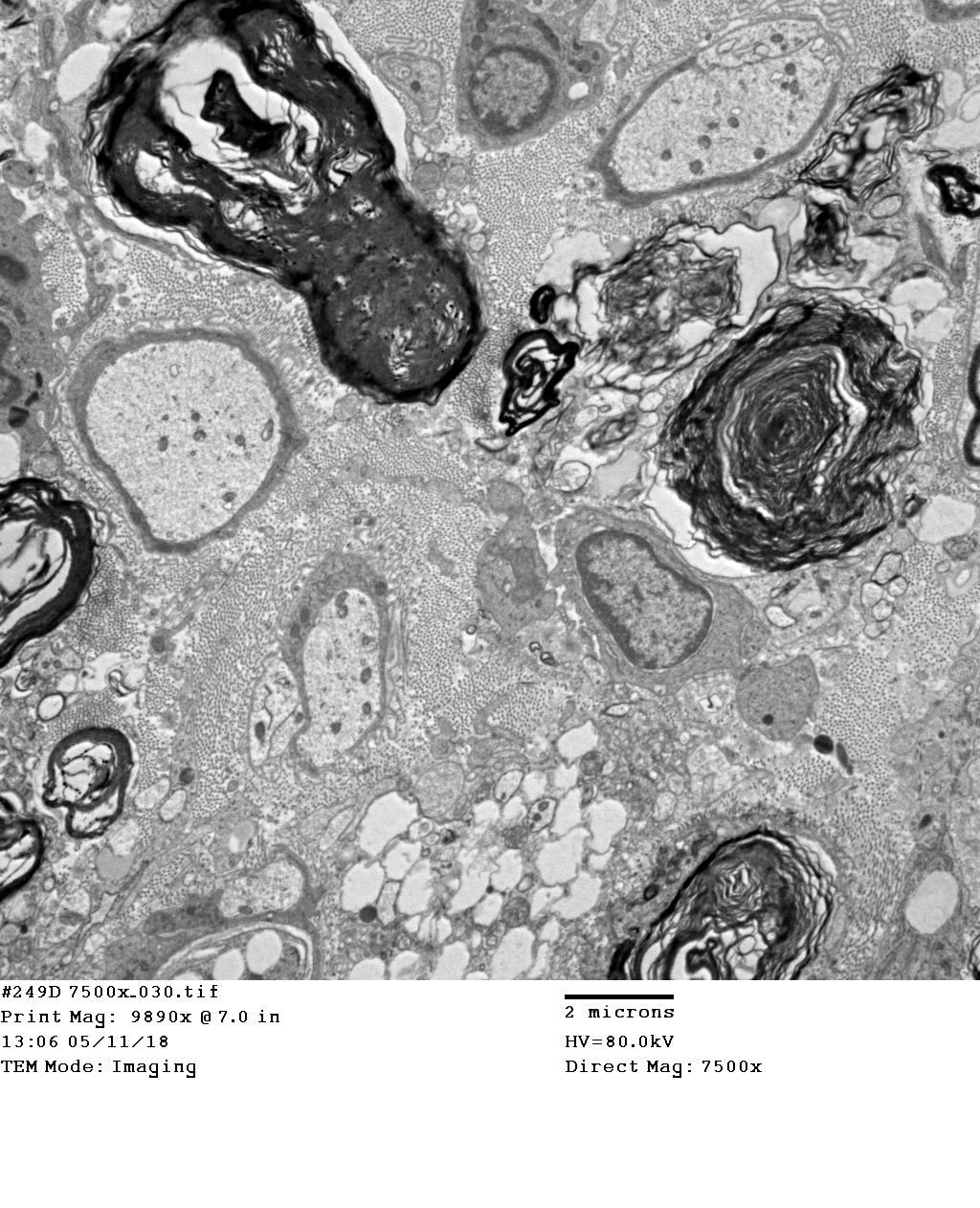

Supplement: Figure 8—source data 1. — This zip archive contains the TEM images for one WT and one Taz iKO used for quantitative analysis shown in Figure 8D–G. Images were taken using a JEOL 1010 electron microscope fitted with a Hamamatsu digital camera and AMT Advantage image capture software. Contrast of the images was adjusted using Photoshop software. [file elife-50138-fig8-data1.zip › Figure 8 source data 1/Taz iKO #249D 7500x/#249D 7500x_030 adjusted.tif]

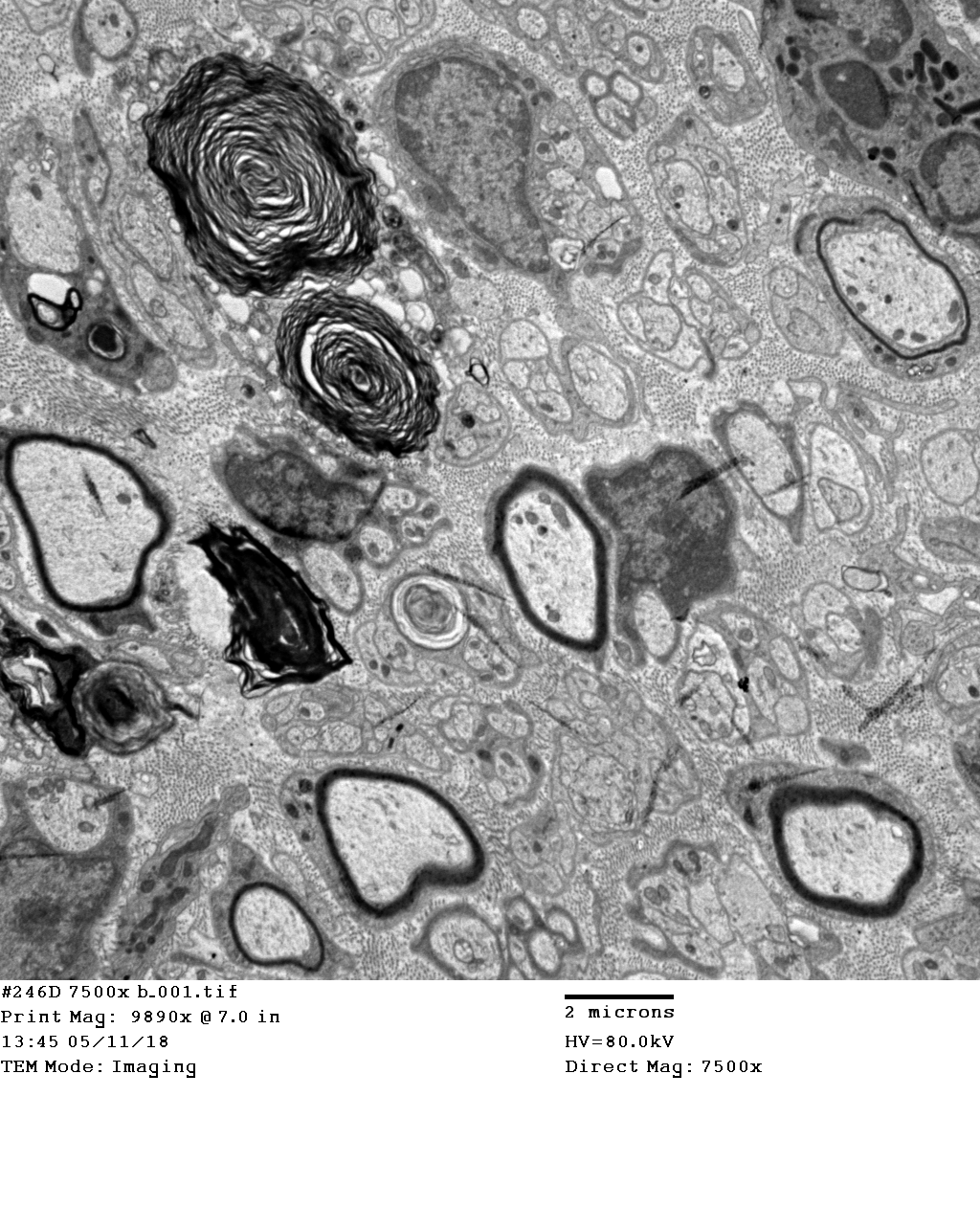

Supplement: Figure 8—source data 1. — This zip archive contains the TEM images for one WT and one Taz iKO used for quantitative analysis shown in Figure 8D–G. Images were taken using a JEOL 1010 electron microscope fitted with a Hamamatsu digital camera and AMT Advantage image capture software. Contrast of the images was adjusted using Photoshop software. [file elife-50138-fig8-data1.zip › Figure 8 source data 1/WT #246D 7500x b/#246D 7500x b_001 adjusted.tif]

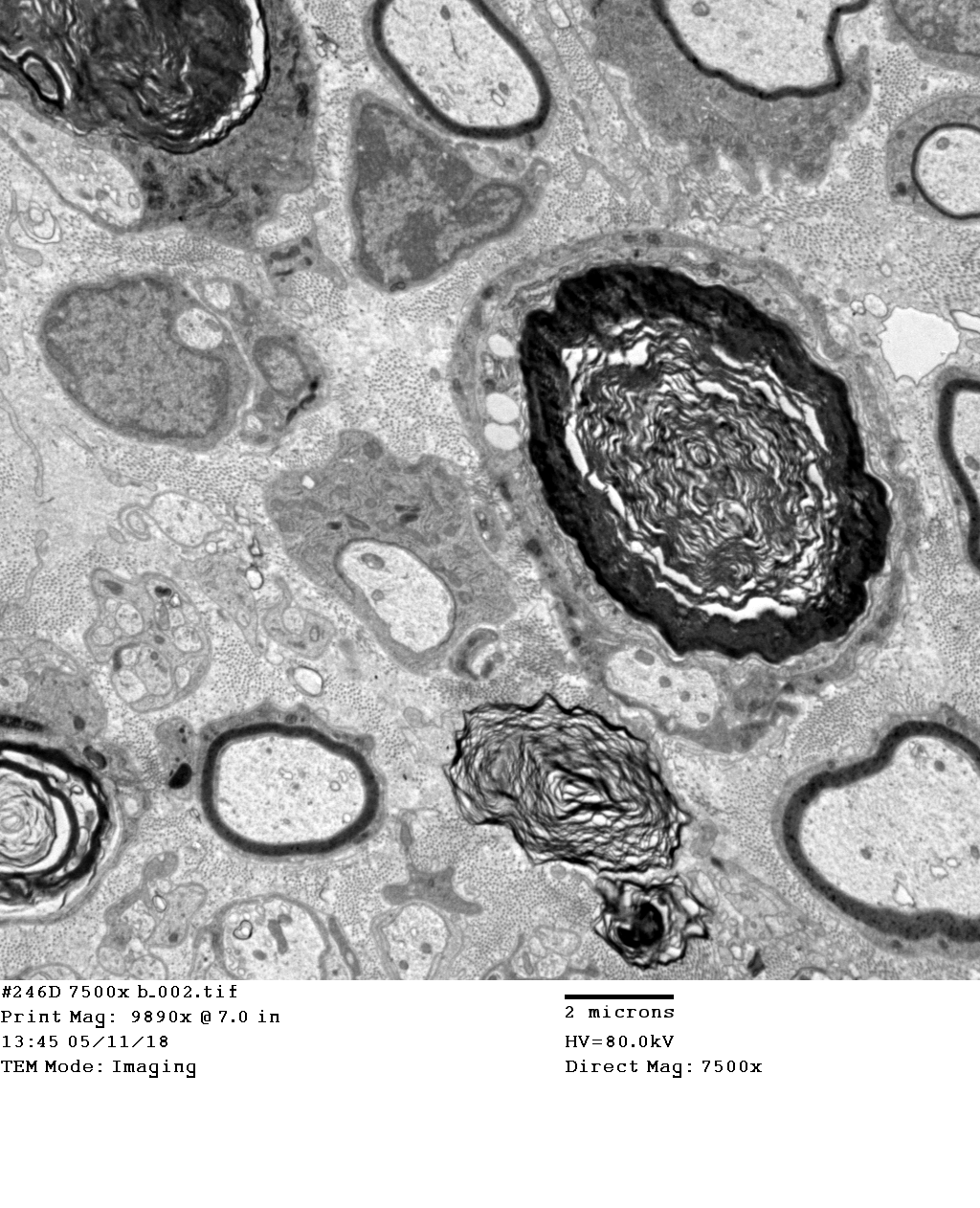

Supplement: Figure 8—source data 1. — This zip archive contains the TEM images for one WT and one Taz iKO used for quantitative analysis shown in Figure 8D–G. Images were taken using a JEOL 1010 electron microscope fitted with a Hamamatsu digital camera and AMT Advantage image capture software. Contrast of the images was adjusted using Photoshop software. [file elife-50138-fig8-data1.zip › Figure 8 source data 1/WT #246D 7500x b/#246D 7500x b_002 adjusted.tif]

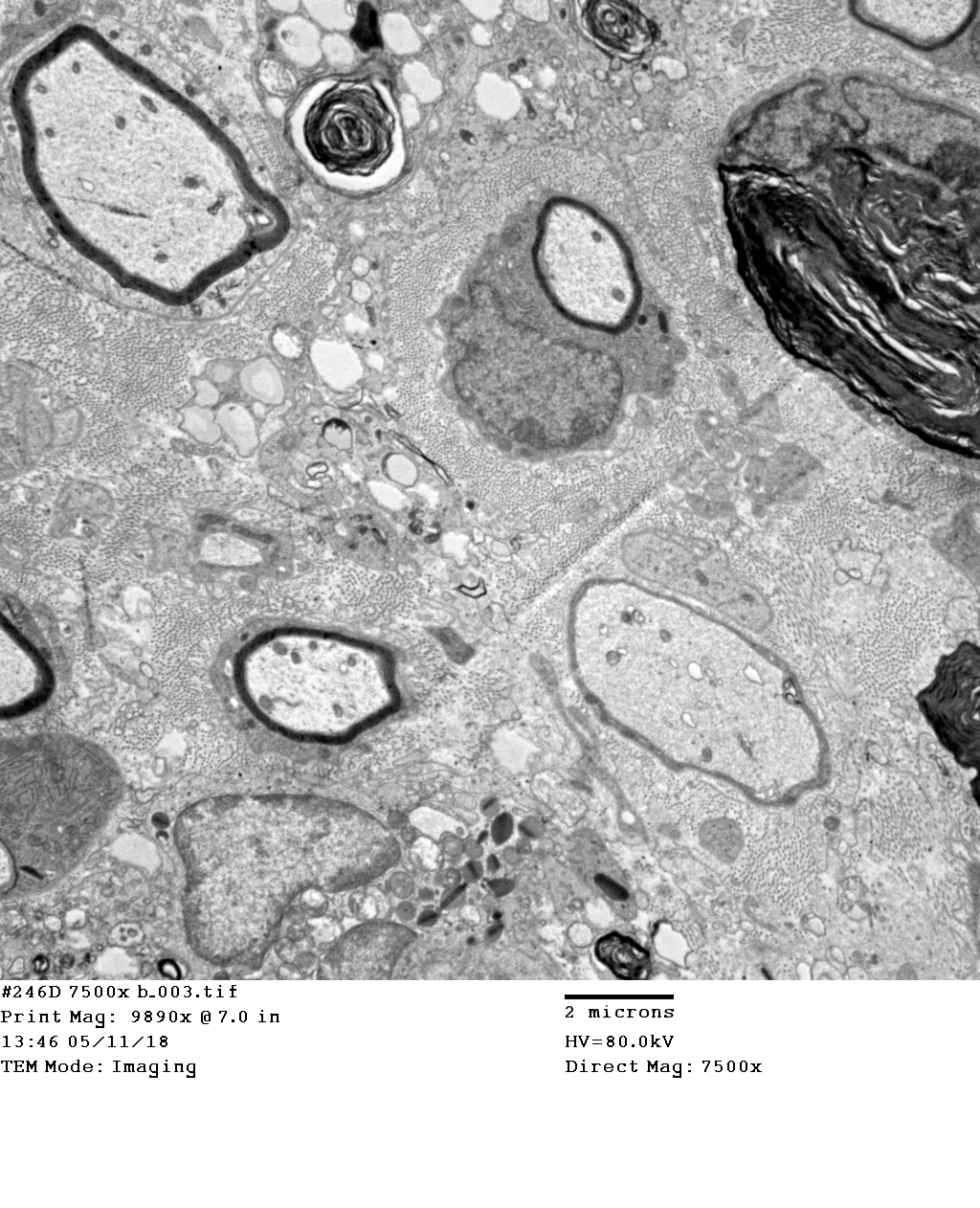

Supplement: Figure 8—source data 1. — This zip archive contains the TEM images for one WT and one Taz iKO used for quantitative analysis shown in Figure 8D–G. Images were taken using a JEOL 1010 electron microscope fitted with a Hamamatsu digital camera and AMT Advantage image capture software. Contrast of the images was adjusted using Photoshop software. [file elife-50138-fig8-data1.zip › Figure 8 source data 1/WT #246D 7500x b/#246D 7500x b_003 adjusted.tif]

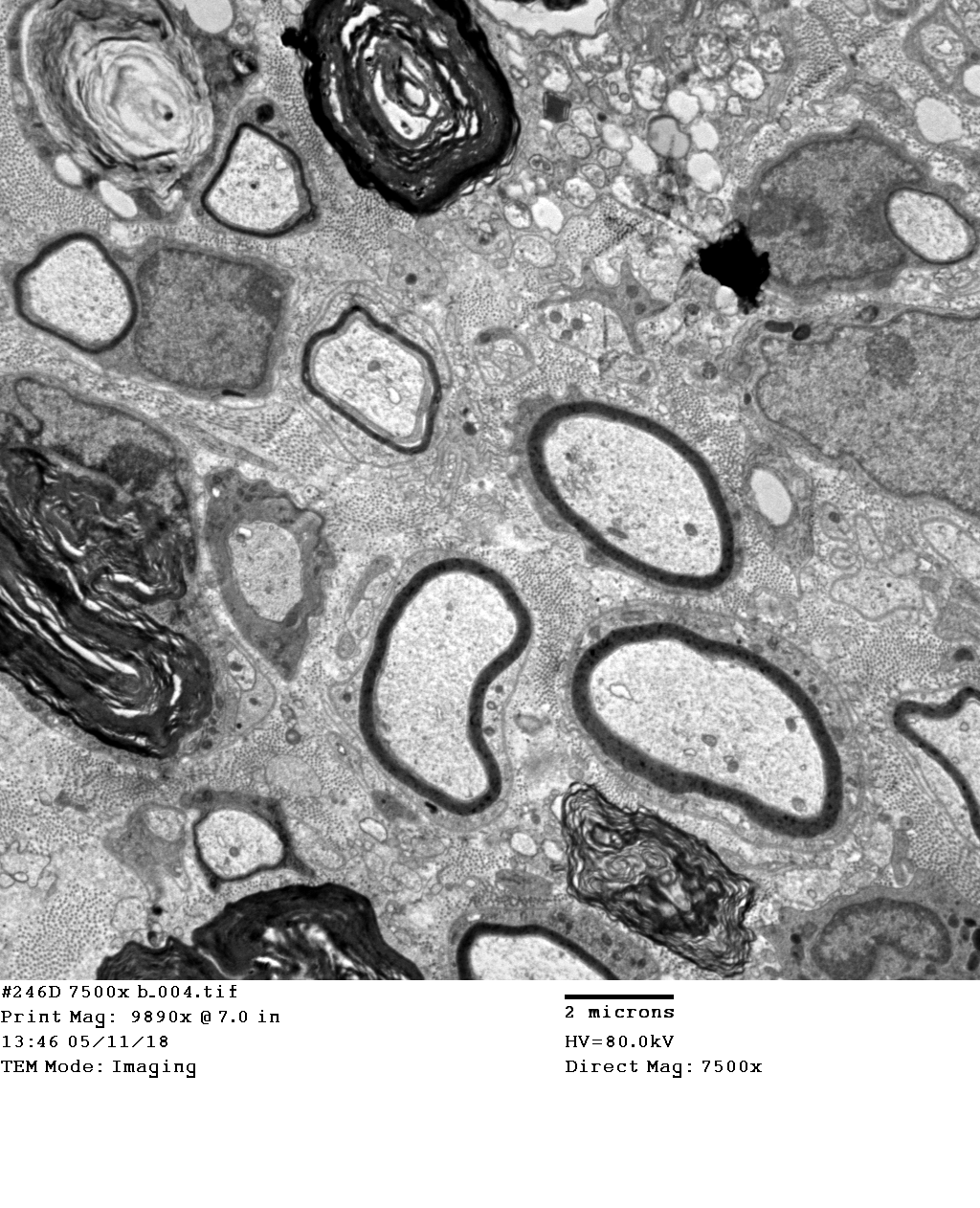

Supplement: Figure 8—source data 1. — This zip archive contains the TEM images for one WT and one Taz iKO used for quantitative analysis shown in Figure 8D–G. Images were taken using a JEOL 1010 electron microscope fitted with a Hamamatsu digital camera and AMT Advantage image capture software. Contrast of the images was adjusted using Photoshop software. [file elife-50138-fig8-data1.zip › Figure 8 source data 1/WT #246D 7500x b/#246D 7500x b_004 adjusted.tif]

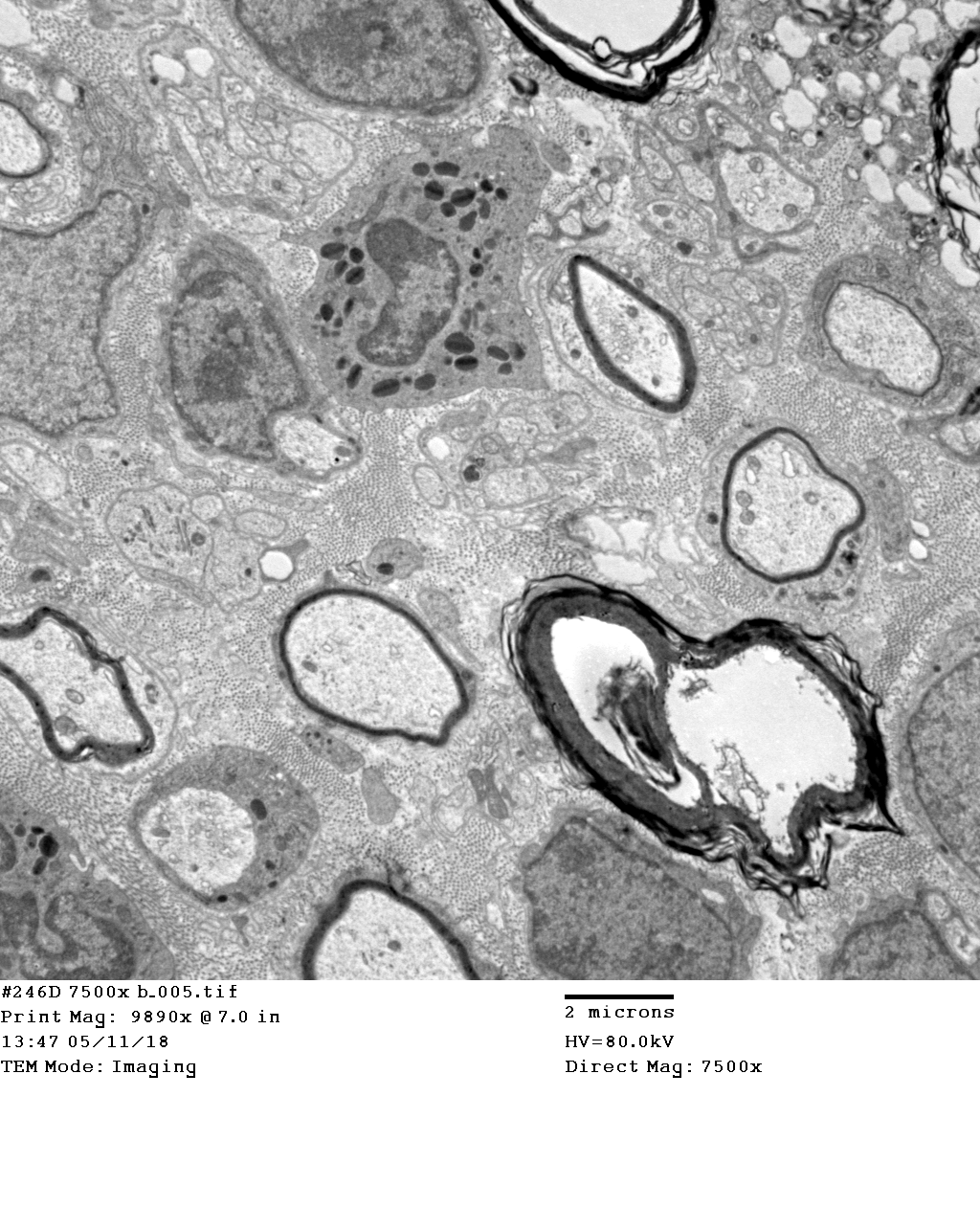

Supplement: Figure 8—source data 1. — This zip archive contains the TEM images for one WT and one Taz iKO used for quantitative analysis shown in Figure 8D–G. Images were taken using a JEOL 1010 electron microscope fitted with a Hamamatsu digital camera and AMT Advantage image capture software. Contrast of the images was adjusted using Photoshop software. [file elife-50138-fig8-data1.zip › Figure 8 source data 1/WT #246D 7500x b/#246D 7500x b_005 adjusted.tif]

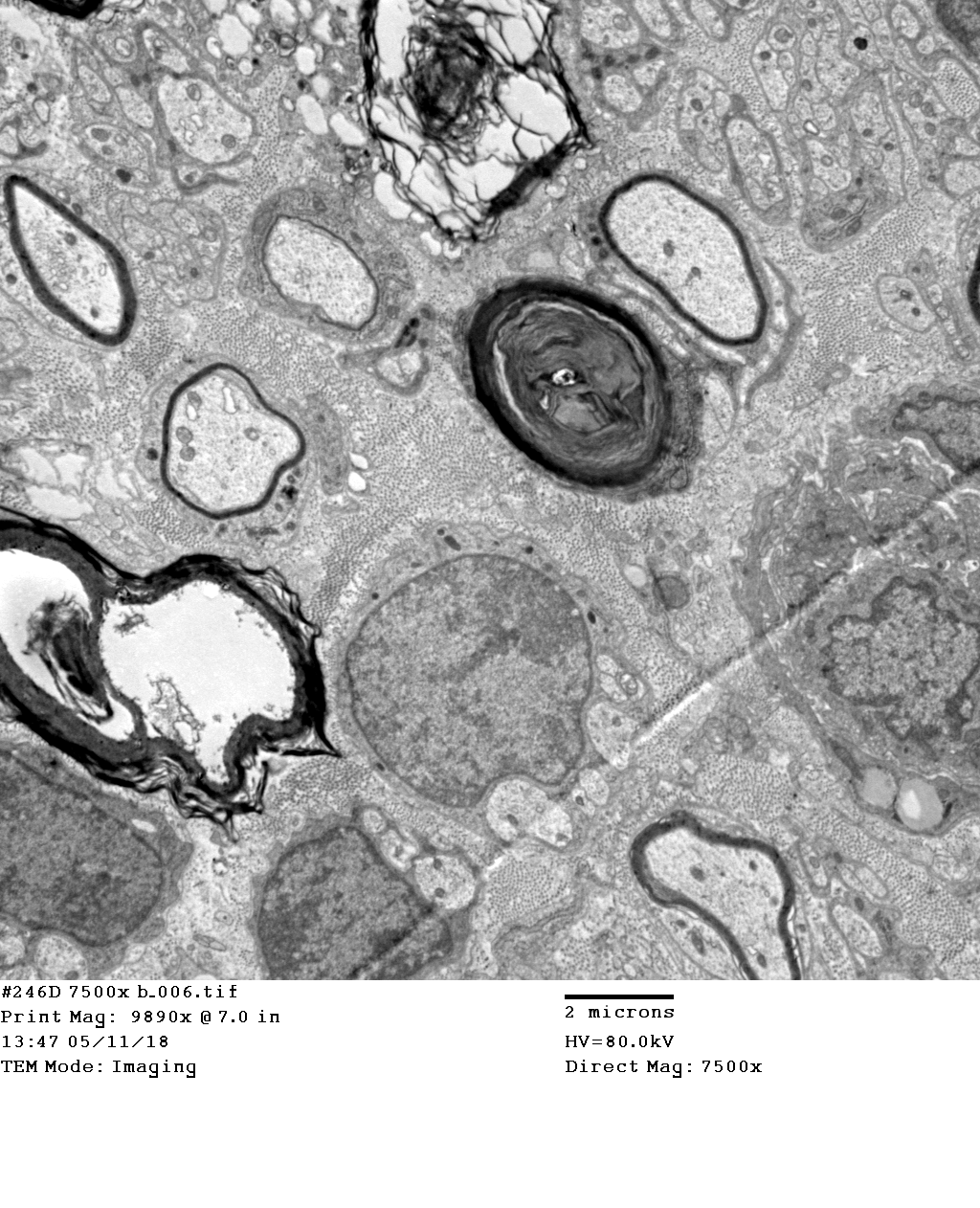

Supplement: Figure 8—source data 1. — This zip archive contains the TEM images for one WT and one Taz iKO used for quantitative analysis shown in Figure 8D–G. Images were taken using a JEOL 1010 electron microscope fitted with a Hamamatsu digital camera and AMT Advantage image capture software. Contrast of the images was adjusted using Photoshop software. [file elife-50138-fig8-data1.zip › Figure 8 source data 1/WT #246D 7500x b/#246D 7500x b_006 adjusted.tif]

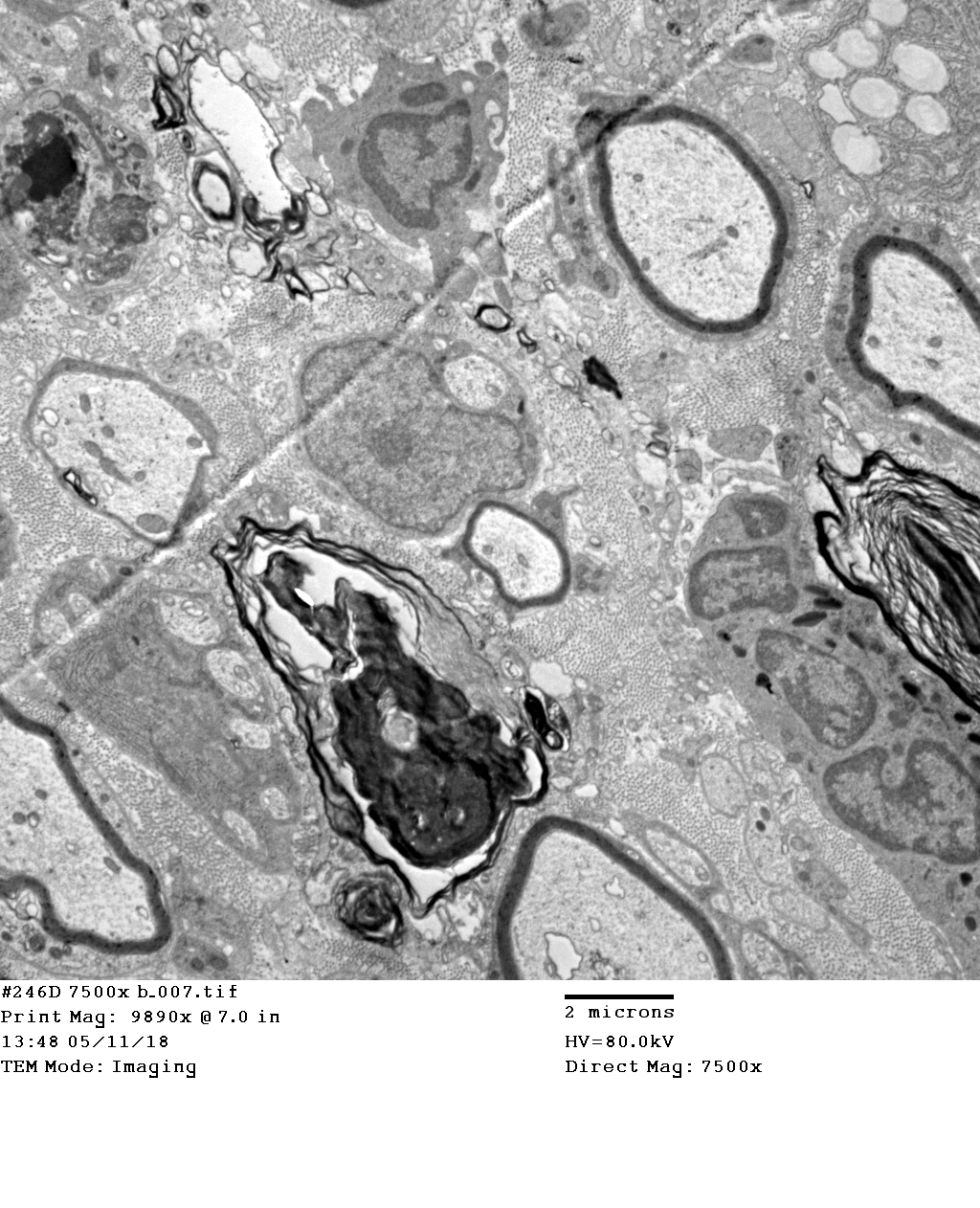

Supplement: Figure 8—source data 1. — This zip archive contains the TEM images for one WT and one Taz iKO used for quantitative analysis shown in Figure 8D–G. Images were taken using a JEOL 1010 electron microscope fitted with a Hamamatsu digital camera and AMT Advantage image capture software. Contrast of the images was adjusted using Photoshop software. [file elife-50138-fig8-data1.zip › Figure 8 source data 1/WT #246D 7500x b/#246D 7500x b_007 adjusted.tif]

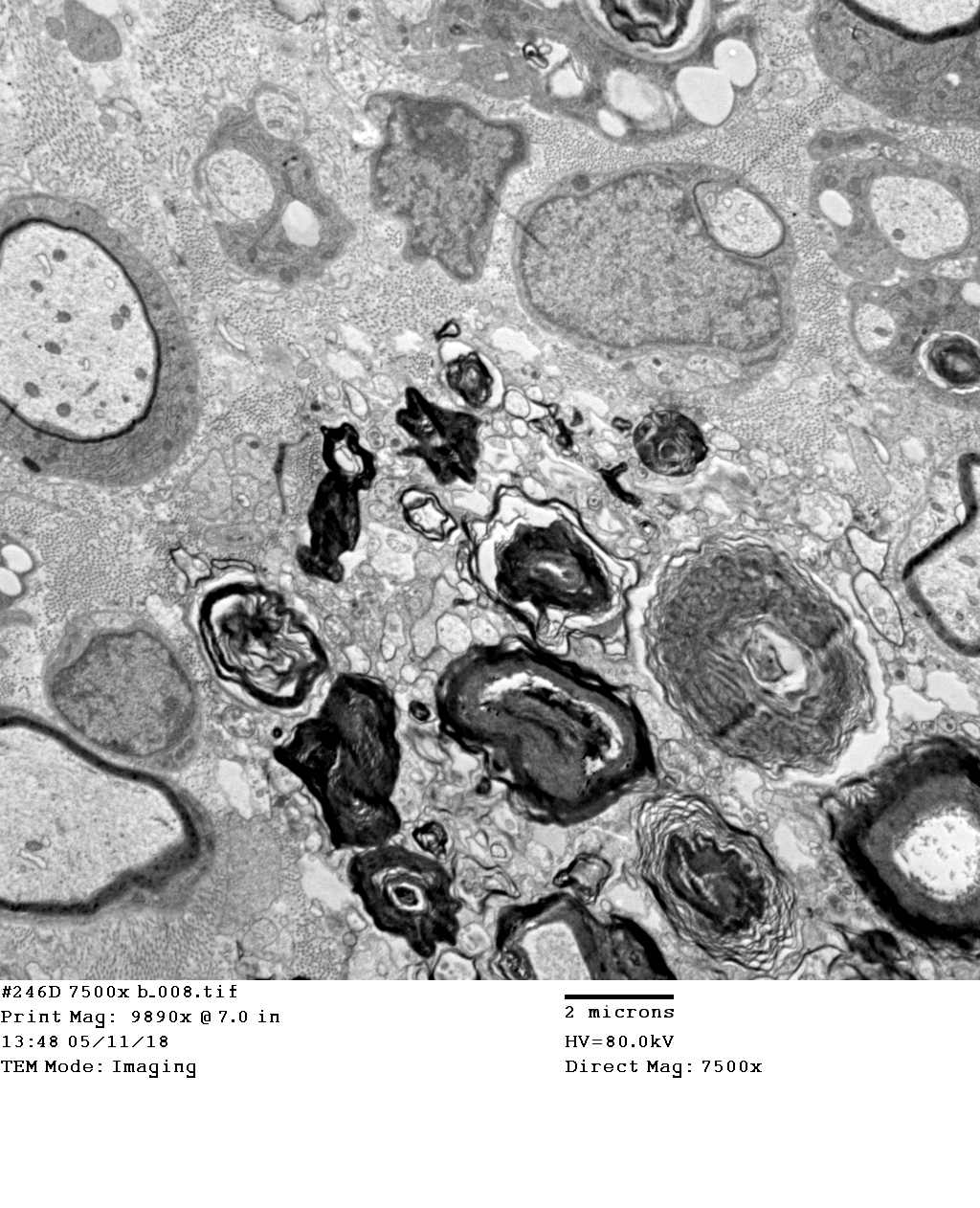

Supplement: Figure 8—source data 1. — This zip archive contains the TEM images for one WT and one Taz iKO used for quantitative analysis shown in Figure 8D–G. Images were taken using a JEOL 1010 electron microscope fitted with a Hamamatsu digital camera and AMT Advantage image capture software. Contrast of the images was adjusted using Photoshop software. [file elife-50138-fig8-data1.zip › Figure 8 source data 1/WT #246D 7500x b/#246D 7500x b_008 adjusted.tif]

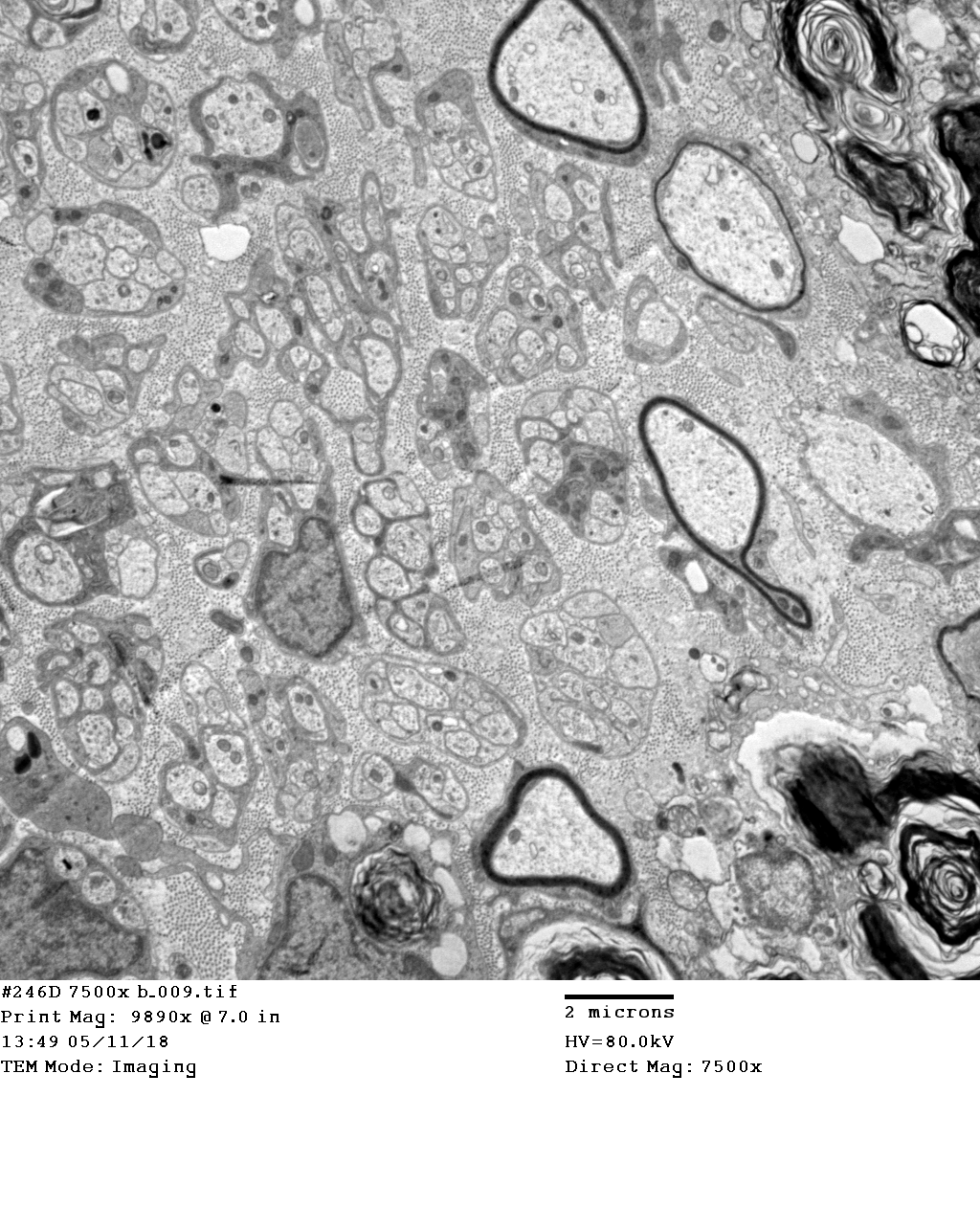

Supplement: Figure 8—source data 1. — This zip archive contains the TEM images for one WT and one Taz iKO used for quantitative analysis shown in Figure 8D–G. Images were taken using a JEOL 1010 electron microscope fitted with a Hamamatsu digital camera and AMT Advantage image capture software. Contrast of the images was adjusted using Photoshop software. [file elife-50138-fig8-data1.zip › Figure 8 source data 1/WT #246D 7500x b/#246D 7500x b_009 adjusted.tif]

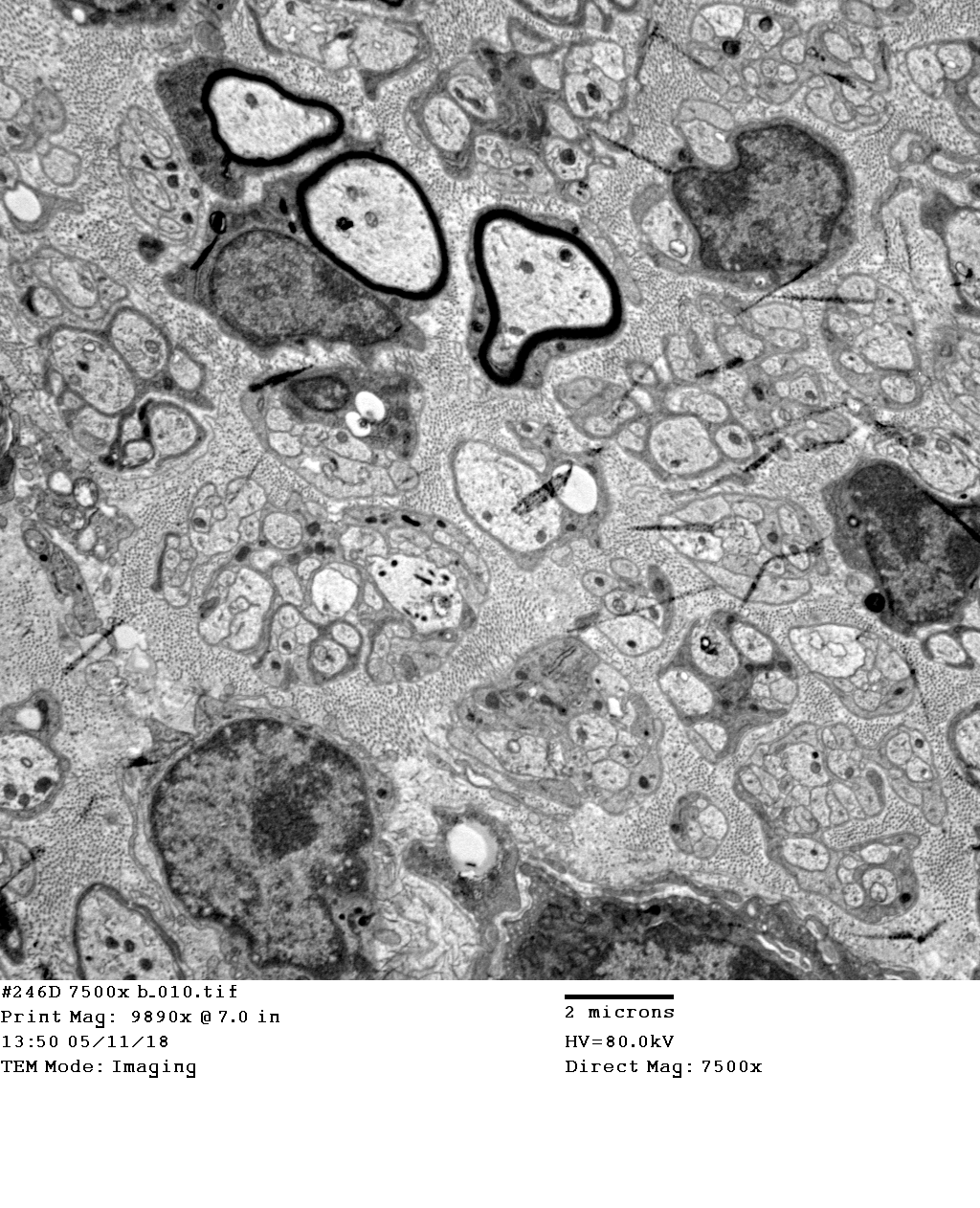

Supplement: Figure 8—source data 1. — This zip archive contains the TEM images for one WT and one Taz iKO used for quantitative analysis shown in Figure 8D–G. Images were taken using a JEOL 1010 electron microscope fitted with a Hamamatsu digital camera and AMT Advantage image capture software. Contrast of the images was adjusted using Photoshop software. [file elife-50138-fig8-data1.zip › Figure 8 source data 1/WT #246D 7500x b/#246D 7500x b_010 adjusted.tif]

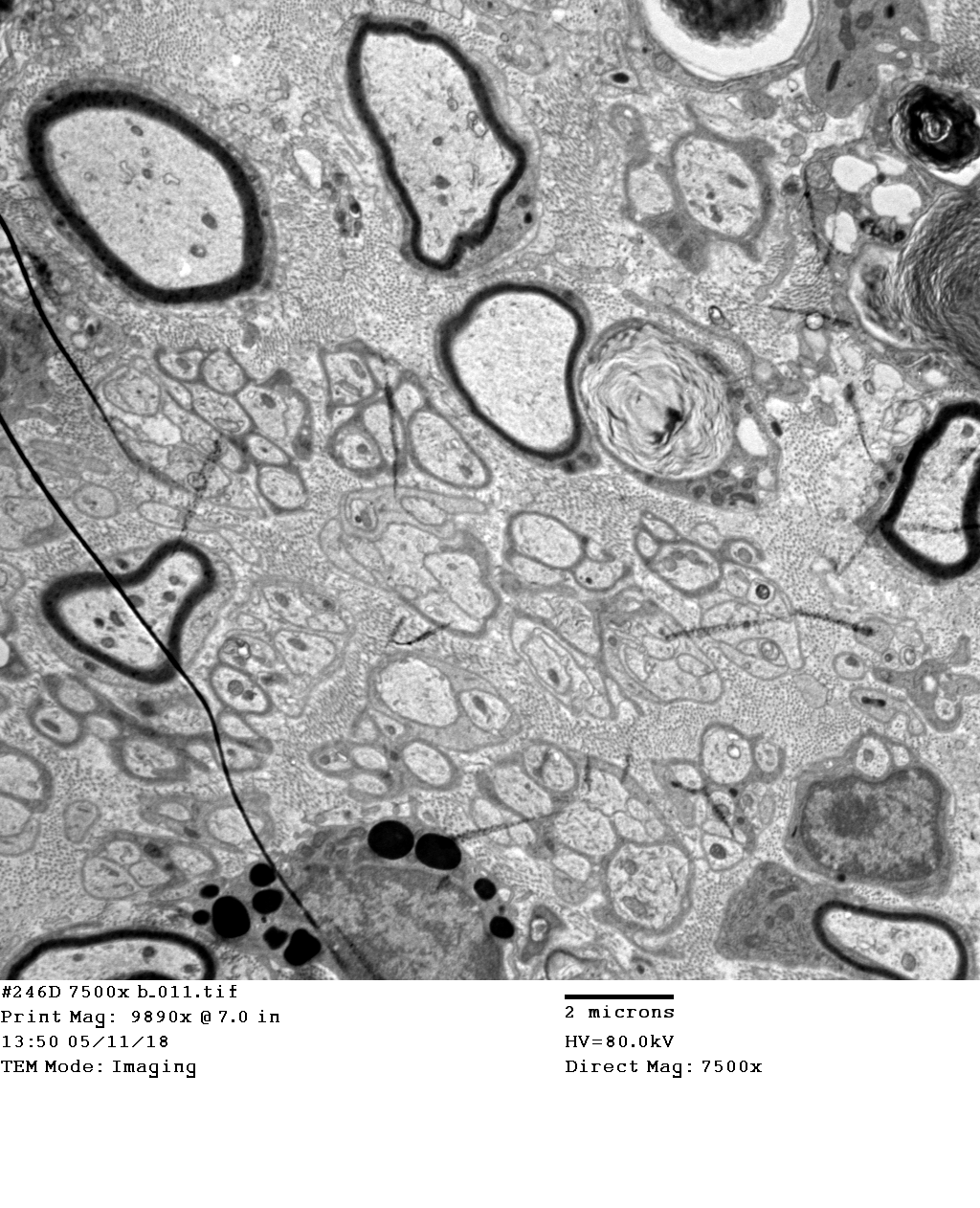

Supplement: Figure 8—source data 1. — This zip archive contains the TEM images for one WT and one Taz iKO used for quantitative analysis shown in Figure 8D–G. Images were taken using a JEOL 1010 electron microscope fitted with a Hamamatsu digital camera and AMT Advantage image capture software. Contrast of the images was adjusted using Photoshop software. [file elife-50138-fig8-data1.zip › Figure 8 source data 1/WT #246D 7500x b/#246D 7500x b_011 adjusted.tif]

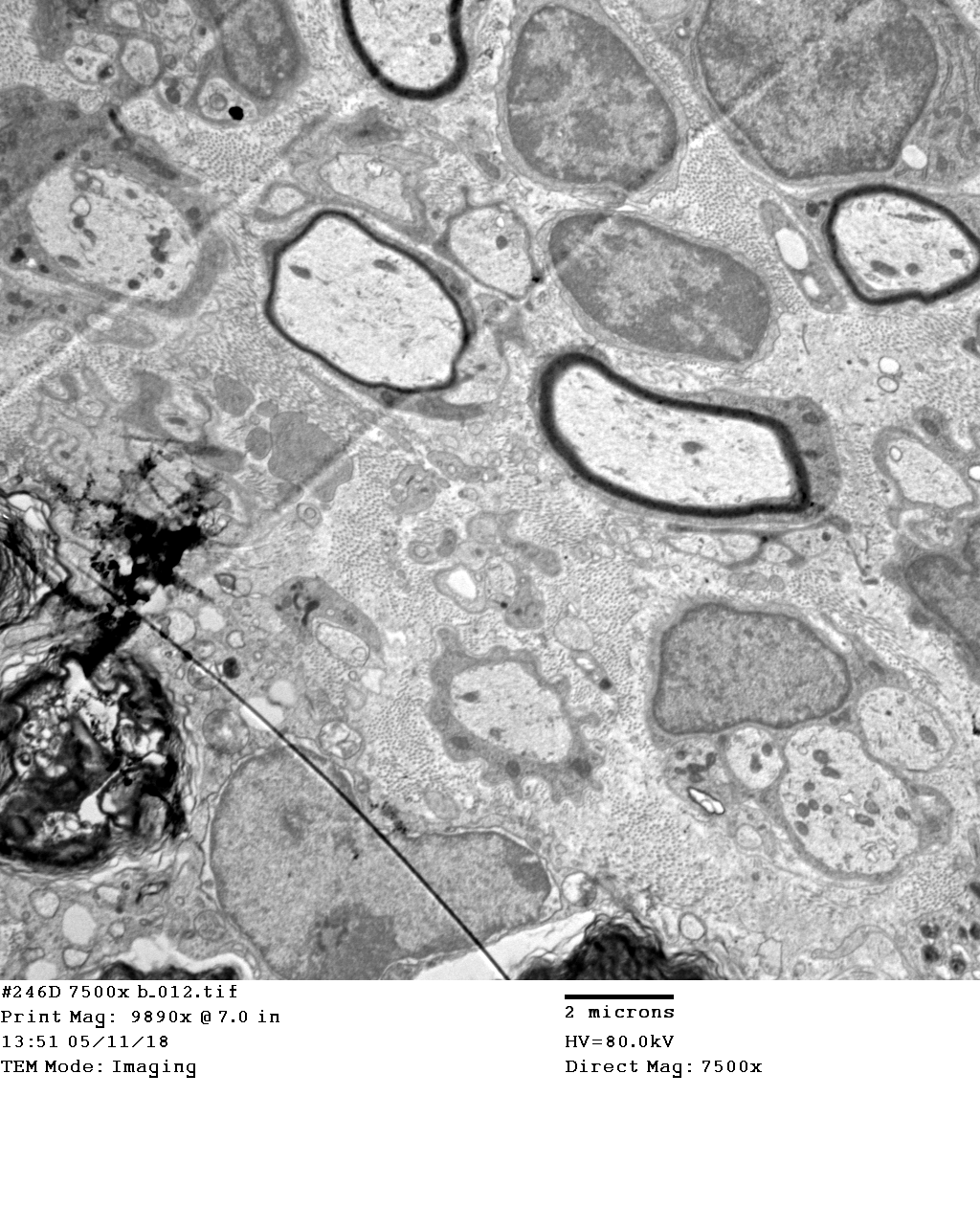

Supplement: Figure 8—source data 1. — This zip archive contains the TEM images for one WT and one Taz iKO used for quantitative analysis shown in Figure 8D–G. Images were taken using a JEOL 1010 electron microscope fitted with a Hamamatsu digital camera and AMT Advantage image capture software. Contrast of the images was adjusted using Photoshop software. [file elife-50138-fig8-data1.zip › Figure 8 source data 1/WT #246D 7500x b/#246D 7500x b_012 adjusted.tif]

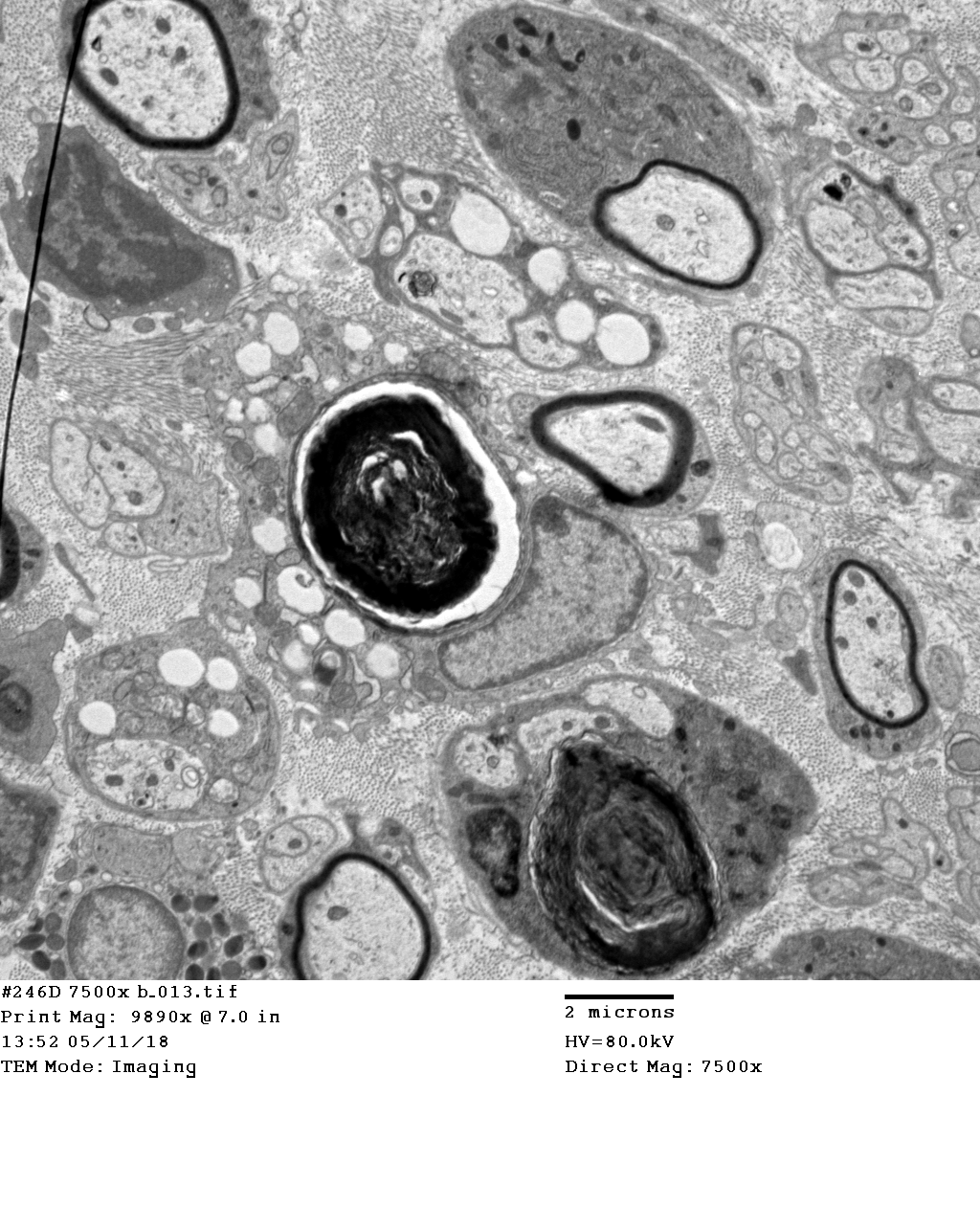

Supplement: Figure 8—source data 1. — This zip archive contains the TEM images for one WT and one Taz iKO used for quantitative analysis shown in Figure 8D–G. Images were taken using a JEOL 1010 electron microscope fitted with a Hamamatsu digital camera and AMT Advantage image capture software. Contrast of the images was adjusted using Photoshop software. [file elife-50138-fig8-data1.zip › Figure 8 source data 1/WT #246D 7500x b/#246D 7500x b_013 adjusted.tif]

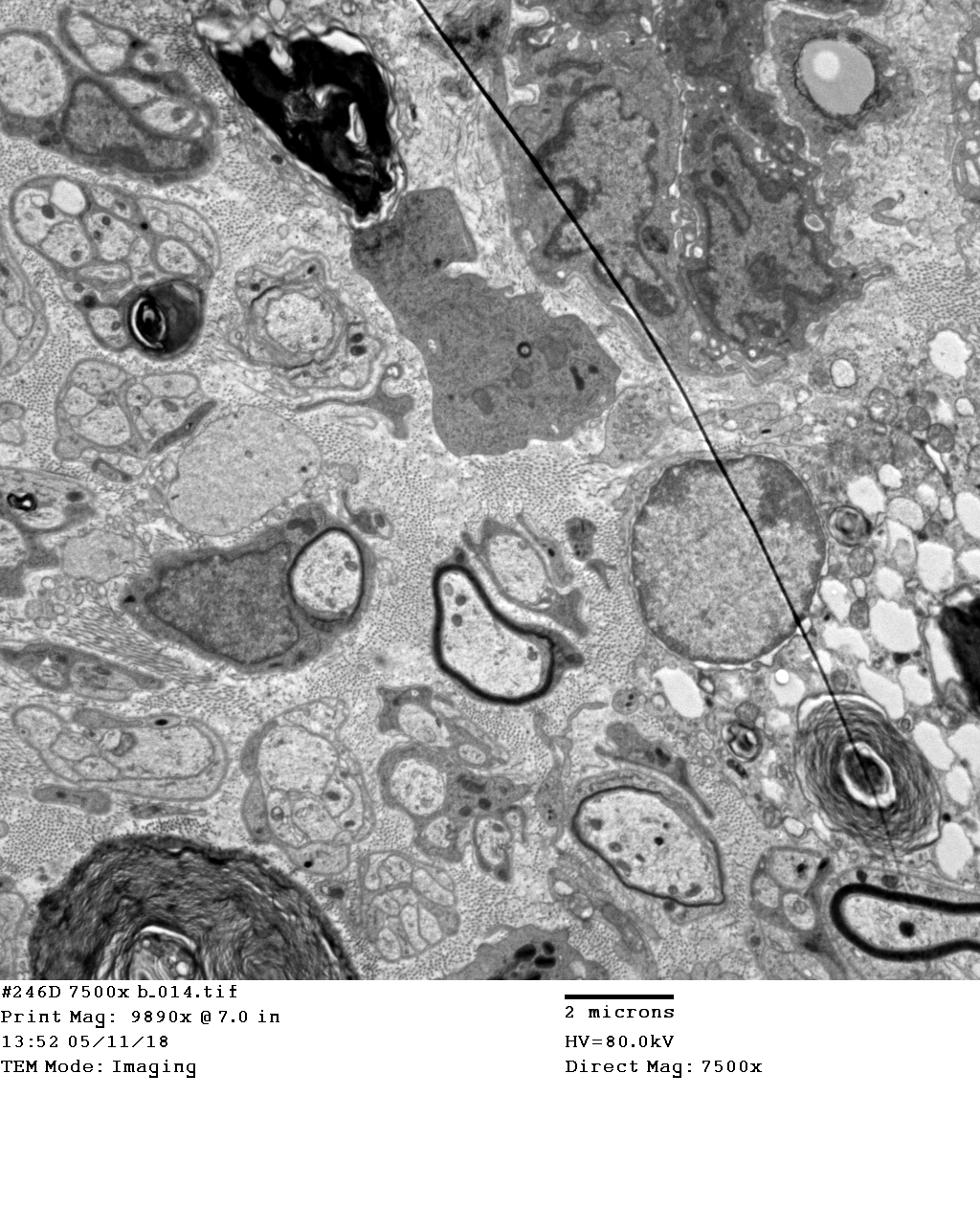

Supplement: Figure 8—source data 1. — This zip archive contains the TEM images for one WT and one Taz iKO used for quantitative analysis shown in Figure 8D–G. Images were taken using a JEOL 1010 electron microscope fitted with a Hamamatsu digital camera and AMT Advantage image capture software. Contrast of the images was adjusted using Photoshop software. [file elife-50138-fig8-data1.zip › Figure 8 source data 1/WT #246D 7500x b/#246D 7500x b_014 adjusted.tif]

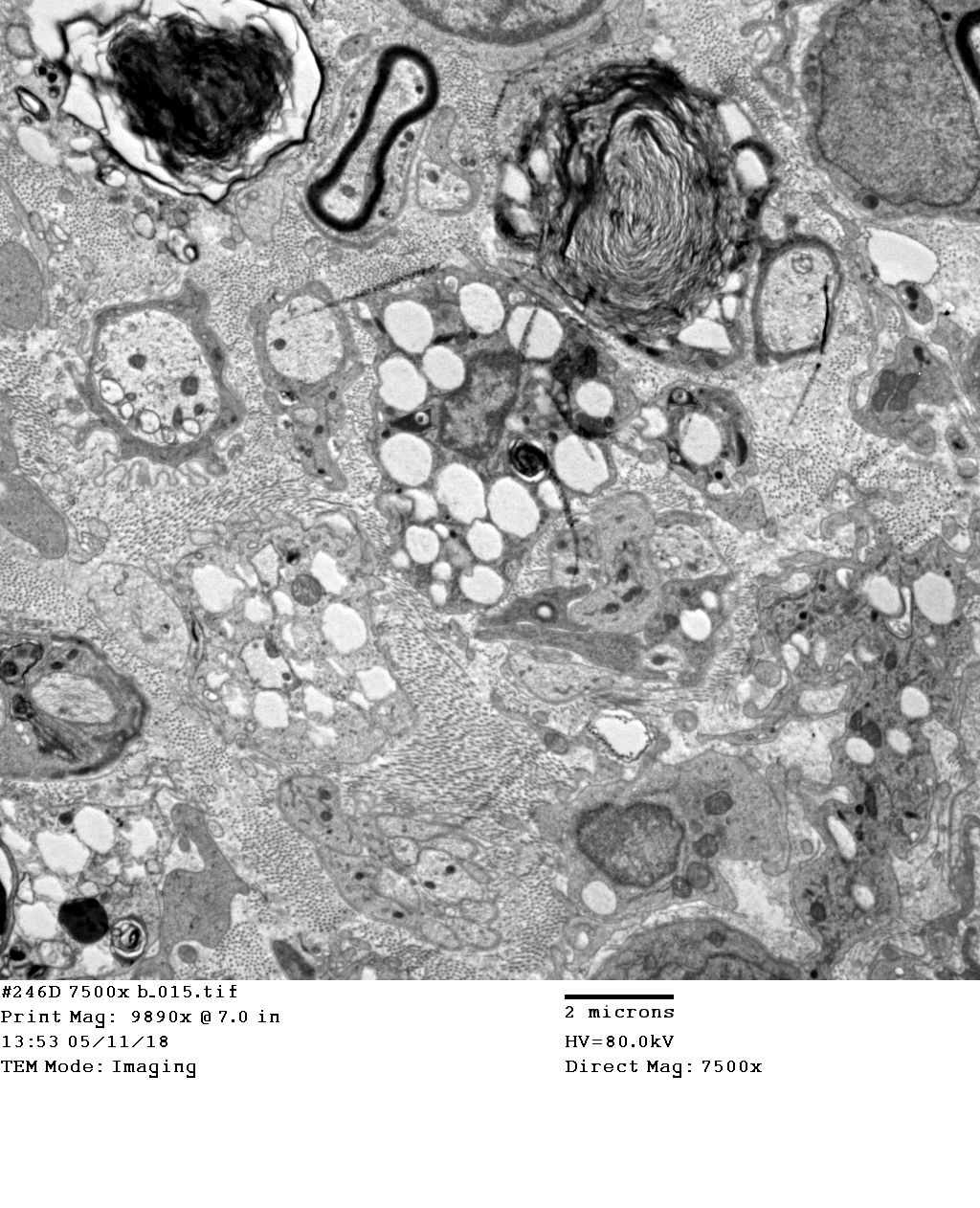

Supplement: Figure 8—source data 1. — This zip archive contains the TEM images for one WT and one Taz iKO used for quantitative analysis shown in Figure 8D–G. Images were taken using a JEOL 1010 electron microscope fitted with a Hamamatsu digital camera and AMT Advantage image capture software. Contrast of the images was adjusted using Photoshop software. [file elife-50138-fig8-data1.zip › Figure 8 source data 1/WT #246D 7500x b/#246D 7500x b_015 adjusted.tif]

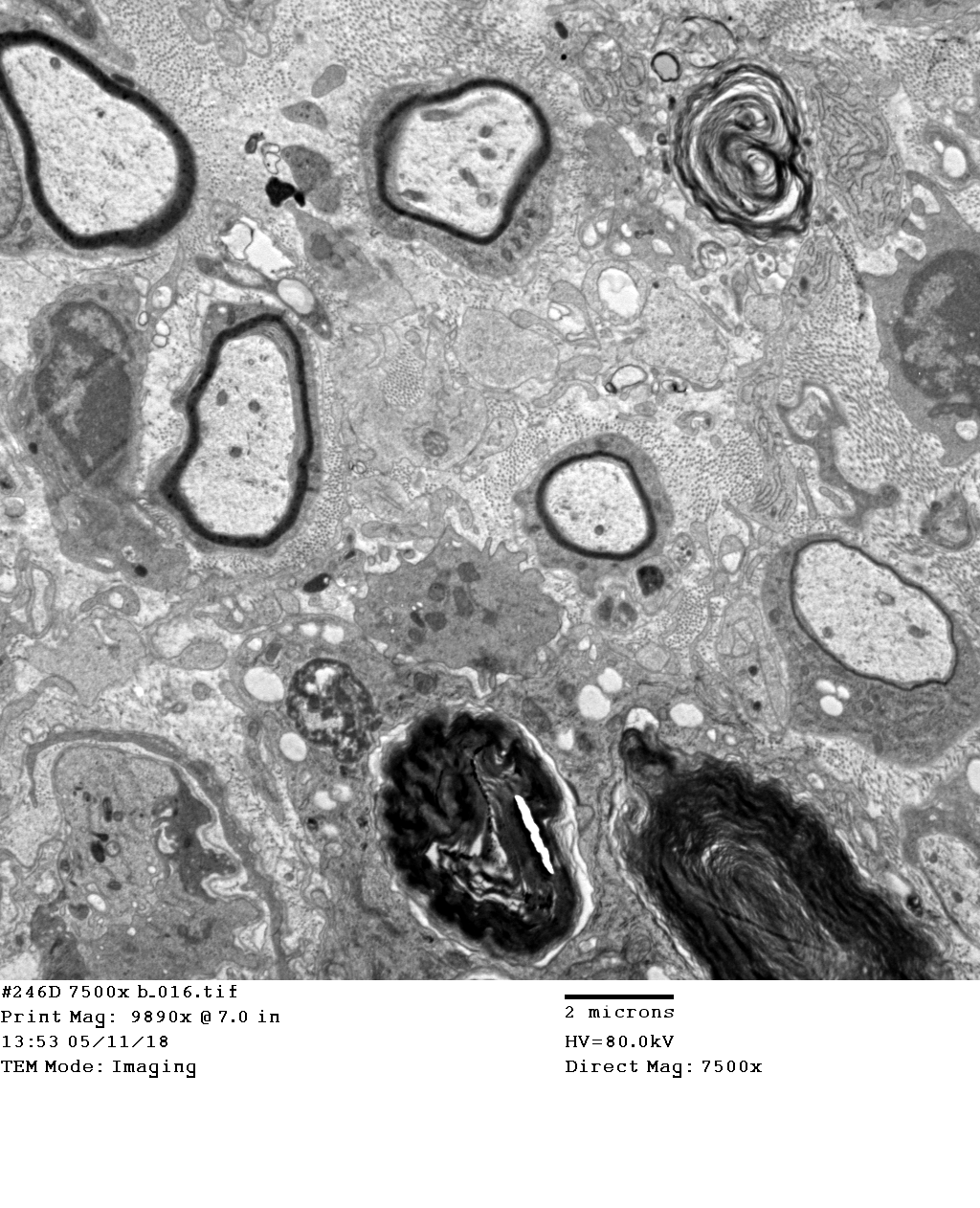

Supplement: Figure 8—source data 1. — This zip archive contains the TEM images for one WT and one Taz iKO used for quantitative analysis shown in Figure 8D–G. Images were taken using a JEOL 1010 electron microscope fitted with a Hamamatsu digital camera and AMT Advantage image capture software. Contrast of the images was adjusted using Photoshop software. [file elife-50138-fig8-data1.zip › Figure 8 source data 1/WT #246D 7500x b/#246D 7500x b_016 adjusted.tif]

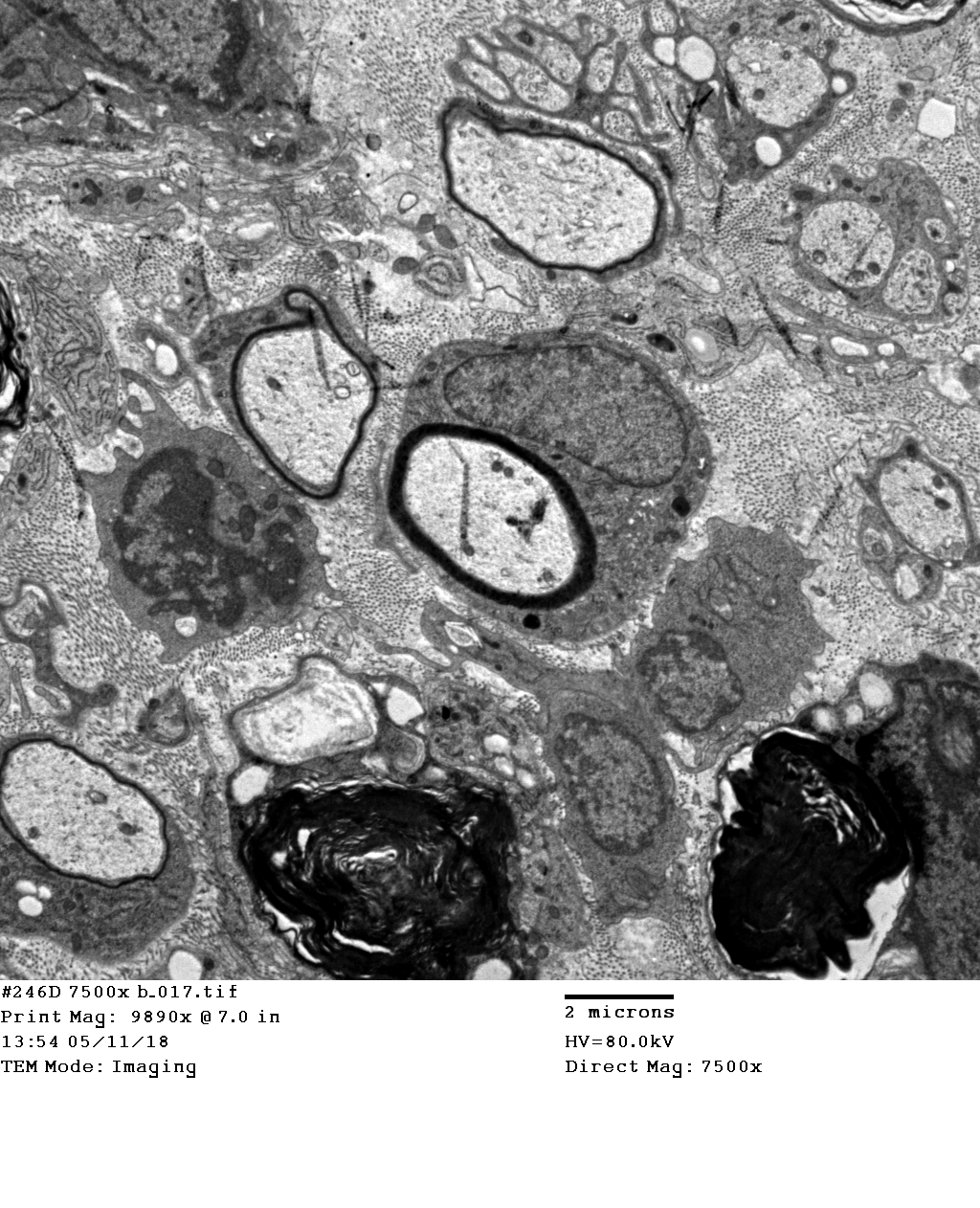

Supplement: Figure 8—source data 1. — This zip archive contains the TEM images for one WT and one Taz iKO used for quantitative analysis shown in Figure 8D–G. Images were taken using a JEOL 1010 electron microscope fitted with a Hamamatsu digital camera and AMT Advantage image capture software. Contrast of the images was adjusted using Photoshop software. [file elife-50138-fig8-data1.zip › Figure 8 source data 1/WT #246D 7500x b/#246D 7500x b_017 adjusted.tif]

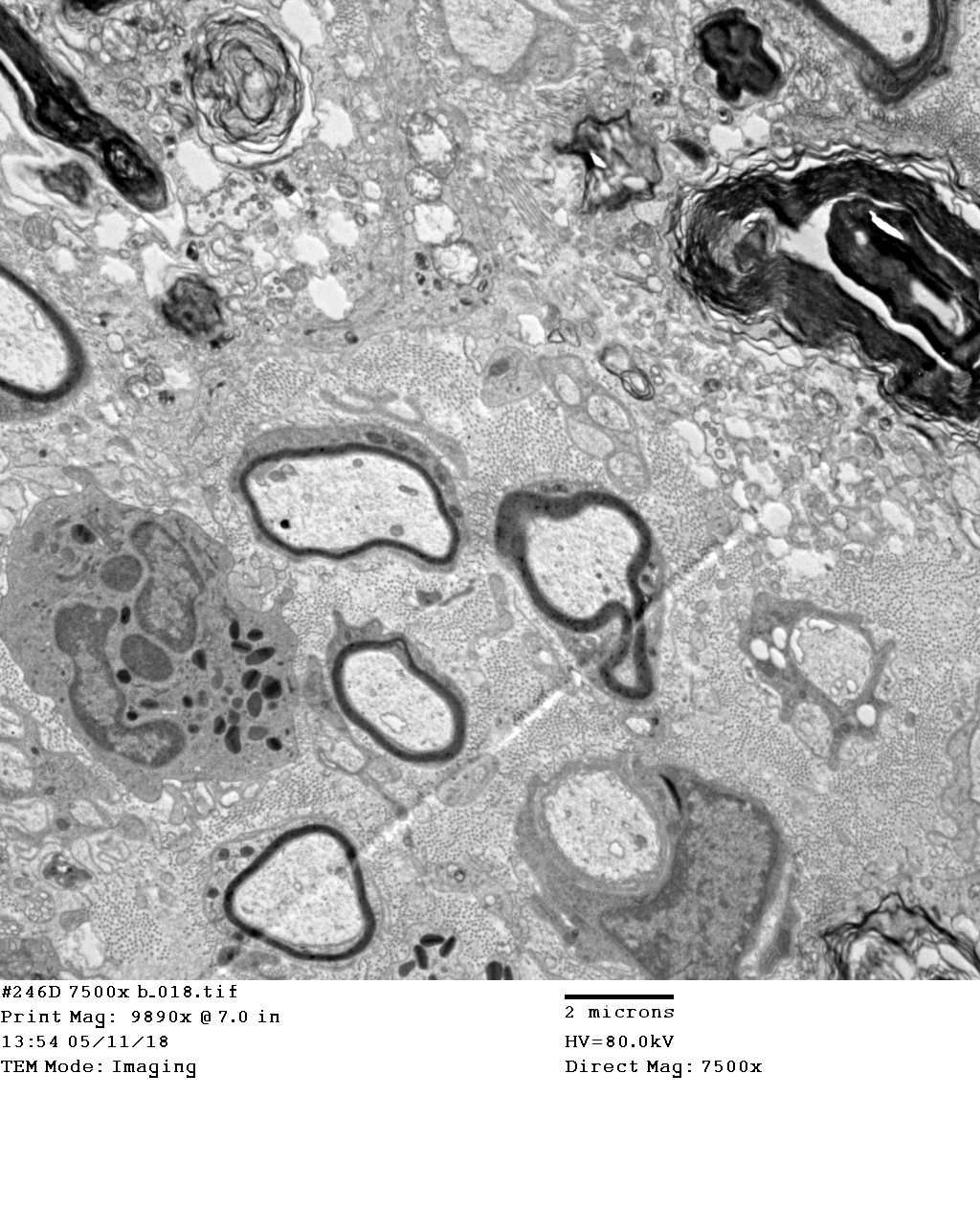

Supplement: Figure 8—source data 1. — This zip archive contains the TEM images for one WT and one Taz iKO used for quantitative analysis shown in Figure 8D–G. Images were taken using a JEOL 1010 electron microscope fitted with a Hamamatsu digital camera and AMT Advantage image capture software. Contrast of the images was adjusted using Photoshop software. [file elife-50138-fig8-data1.zip › Figure 8 source data 1/WT #246D 7500x b/#246D 7500x b_018 adjusted.tif]

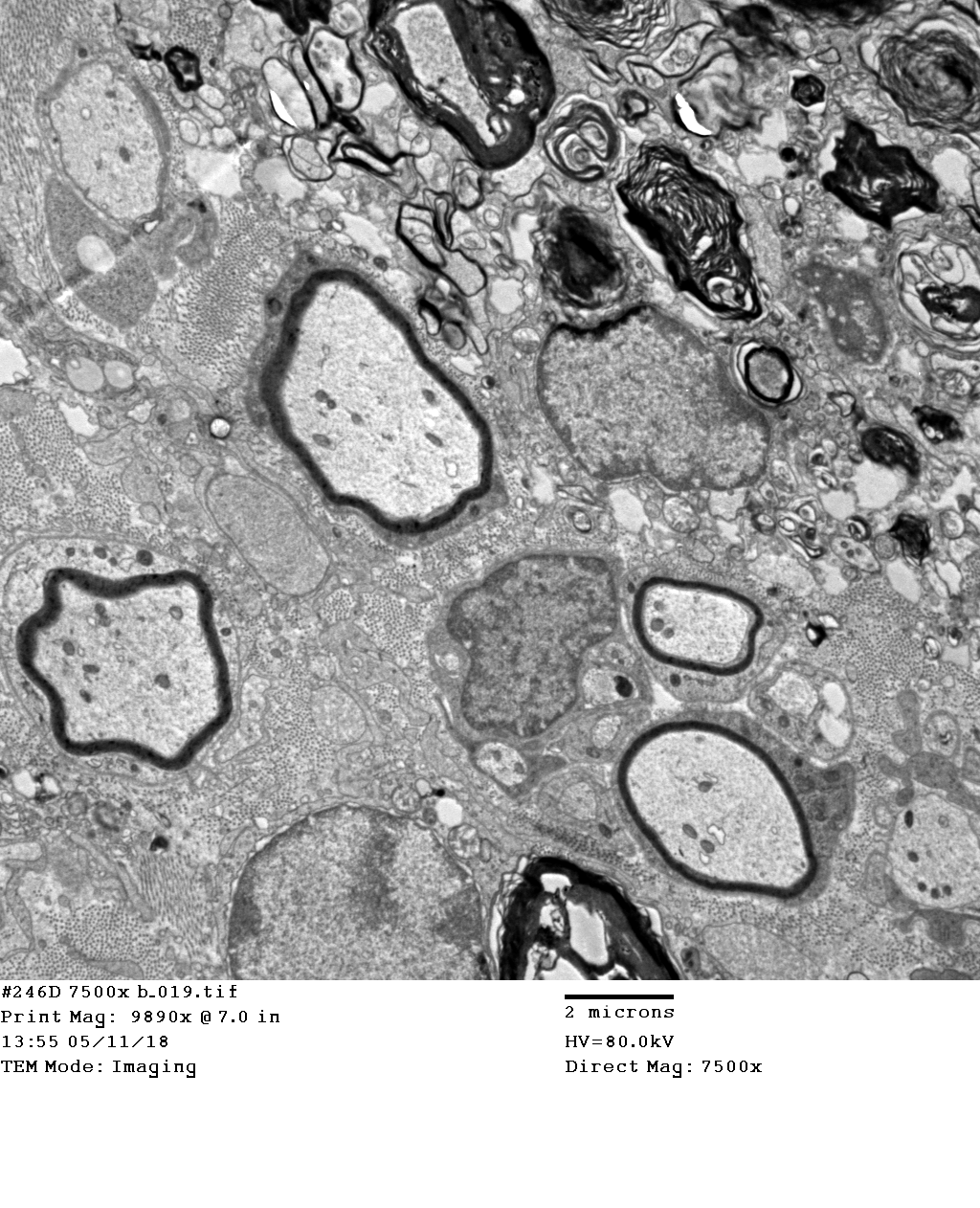

Supplement: Figure 8—source data 1. — This zip archive contains the TEM images for one WT and one Taz iKO used for quantitative analysis shown in Figure 8D–G. Images were taken using a JEOL 1010 electron microscope fitted with a Hamamatsu digital camera and AMT Advantage image capture software. Contrast of the images was adjusted using Photoshop software. [file elife-50138-fig8-data1.zip › Figure 8 source data 1/WT #246D 7500x b/#246D 7500x b_019 adjusted.tif]

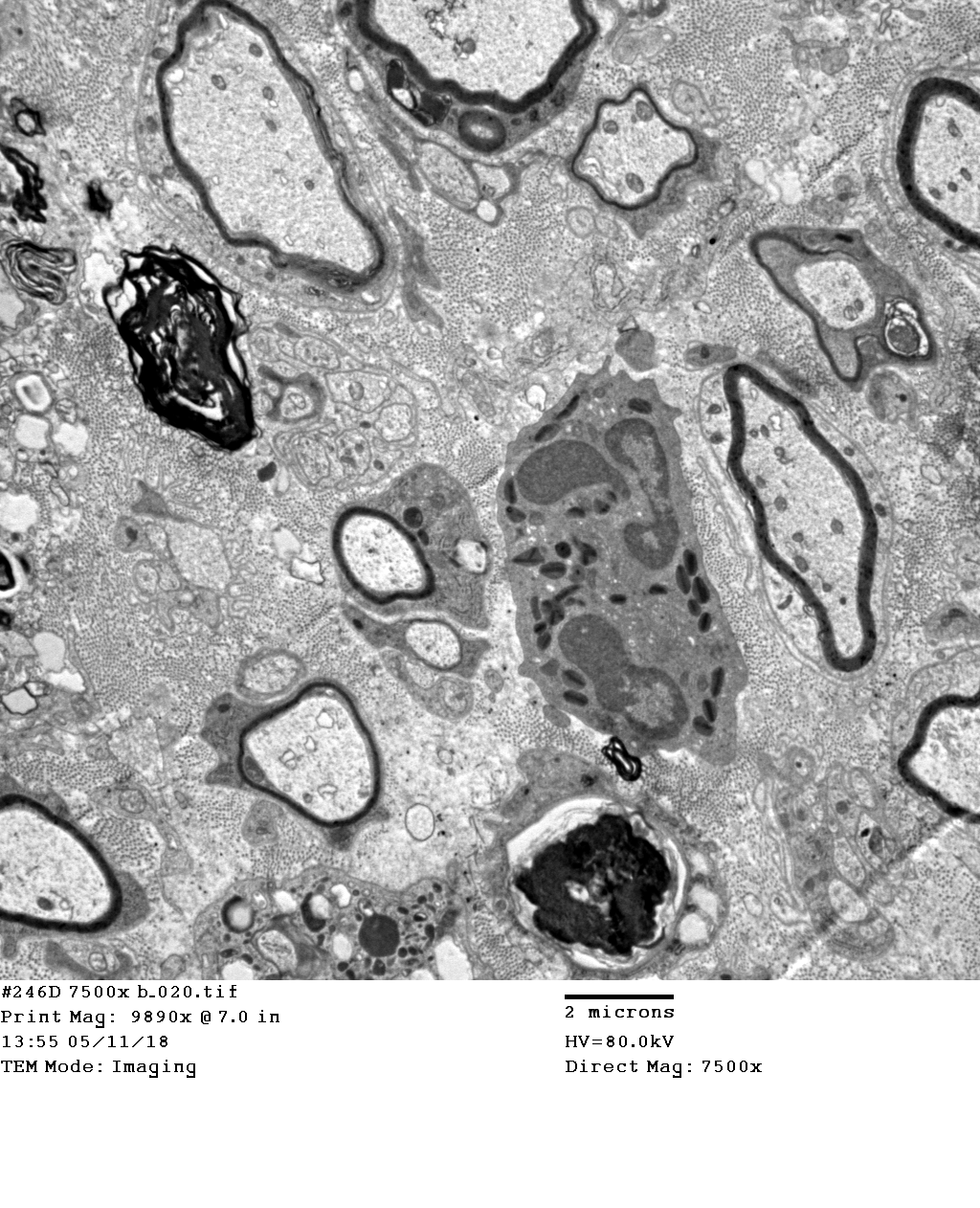

Supplement: Figure 8—source data 1. — This zip archive contains the TEM images for one WT and one Taz iKO used for quantitative analysis shown in Figure 8D–G. Images were taken using a JEOL 1010 electron microscope fitted with a Hamamatsu digital camera and AMT Advantage image capture software. Contrast of the images was adjusted using Photoshop software. [file elife-50138-fig8-data1.zip › Figure 8 source data 1/WT #246D 7500x b/#246D 7500x b_020 adjusted.tif]

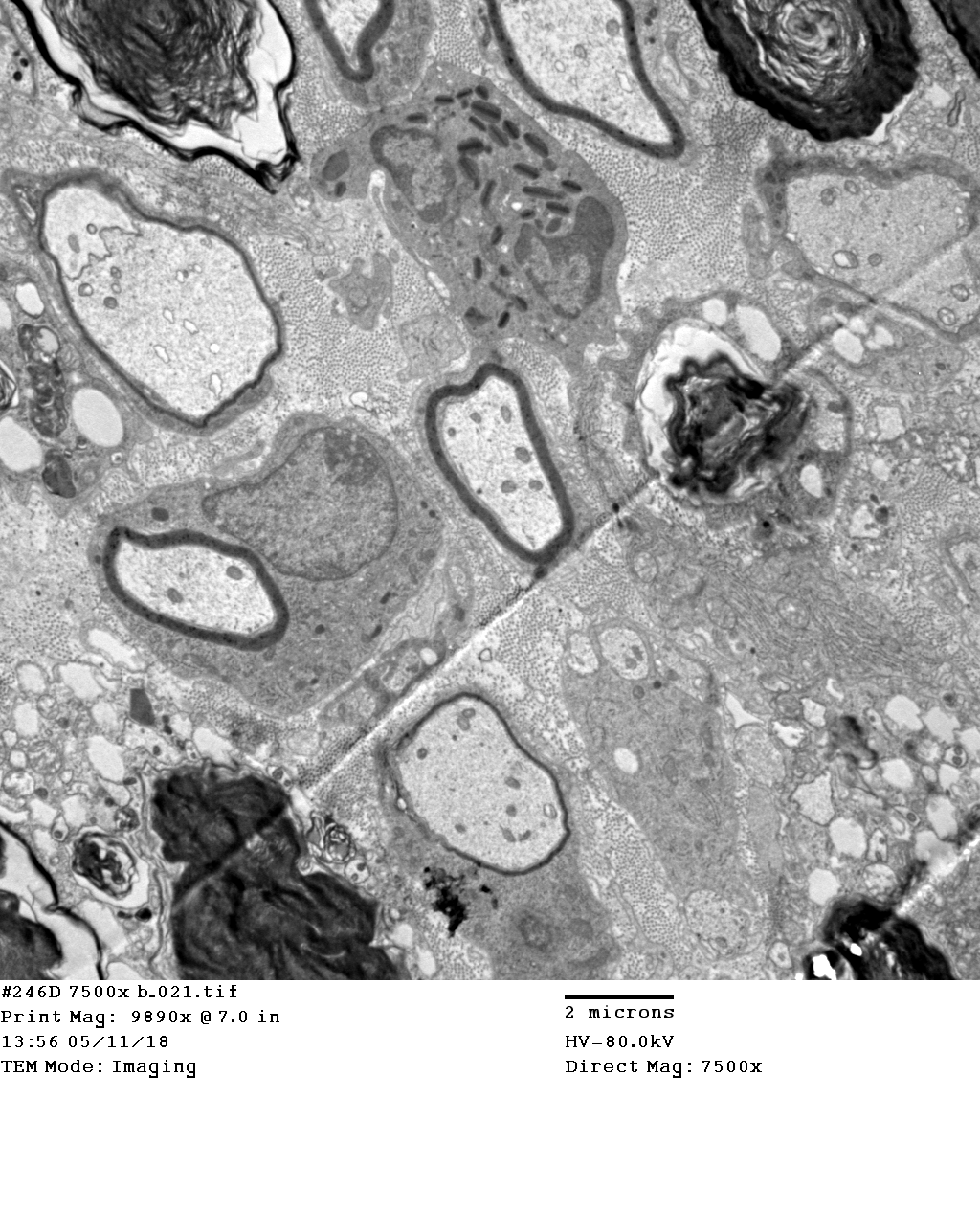

Supplement: Figure 8—source data 1. — This zip archive contains the TEM images for one WT and one Taz iKO used for quantitative analysis shown in Figure 8D–G. Images were taken using a JEOL 1010 electron microscope fitted with a Hamamatsu digital camera and AMT Advantage image capture software. Contrast of the images was adjusted using Photoshop software. [file elife-50138-fig8-data1.zip › Figure 8 source data 1/WT #246D 7500x b/#246D 7500x b_021 adjusted.tif]

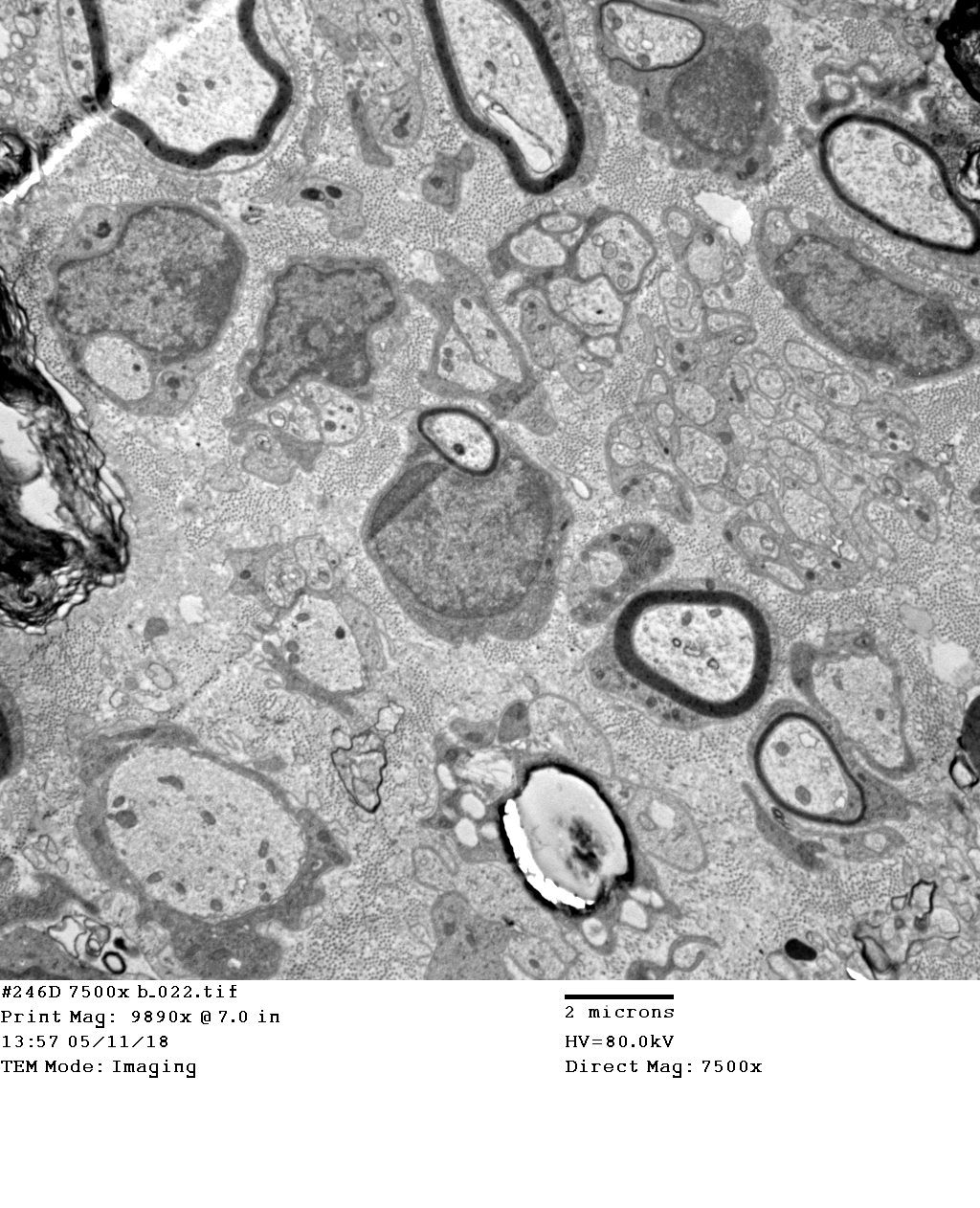

Supplement: Figure 8—source data 1. — This zip archive contains the TEM images for one WT and one Taz iKO used for quantitative analysis shown in Figure 8D–G. Images were taken using a JEOL 1010 electron microscope fitted with a Hamamatsu digital camera and AMT Advantage image capture software. Contrast of the images was adjusted using Photoshop software. [file elife-50138-fig8-data1.zip › Figure 8 source data 1/WT #246D 7500x b/#246D 7500x b_022 adjusted.tif]

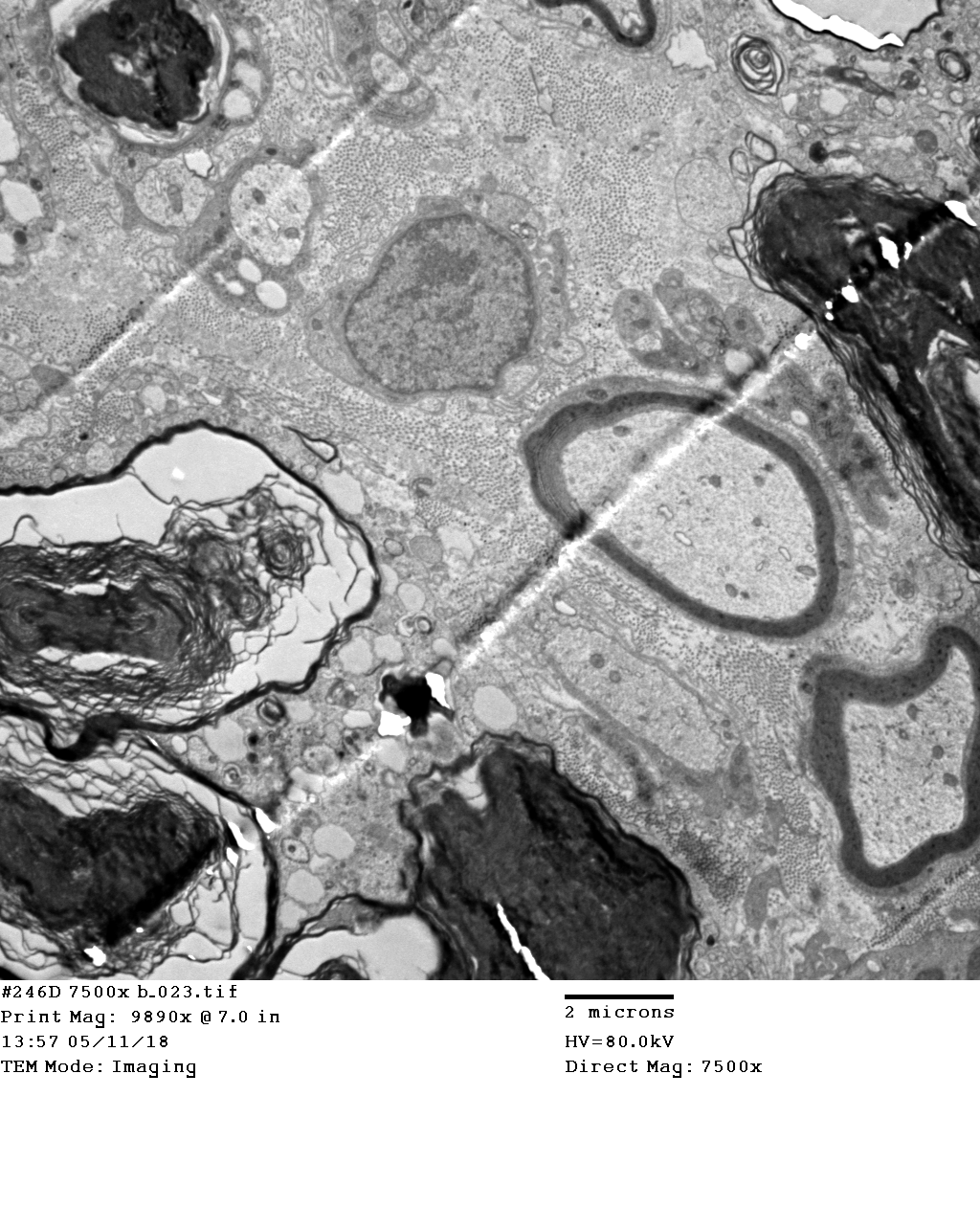

Supplement: Figure 8—source data 1. — This zip archive contains the TEM images for one WT and one Taz iKO used for quantitative analysis shown in Figure 8D–G. Images were taken using a JEOL 1010 electron microscope fitted with a Hamamatsu digital camera and AMT Advantage image capture software. Contrast of the images was adjusted using Photoshop software. [file elife-50138-fig8-data1.zip › Figure 8 source data 1/WT #246D 7500x b/#246D 7500x b_023 adjusted.tif]

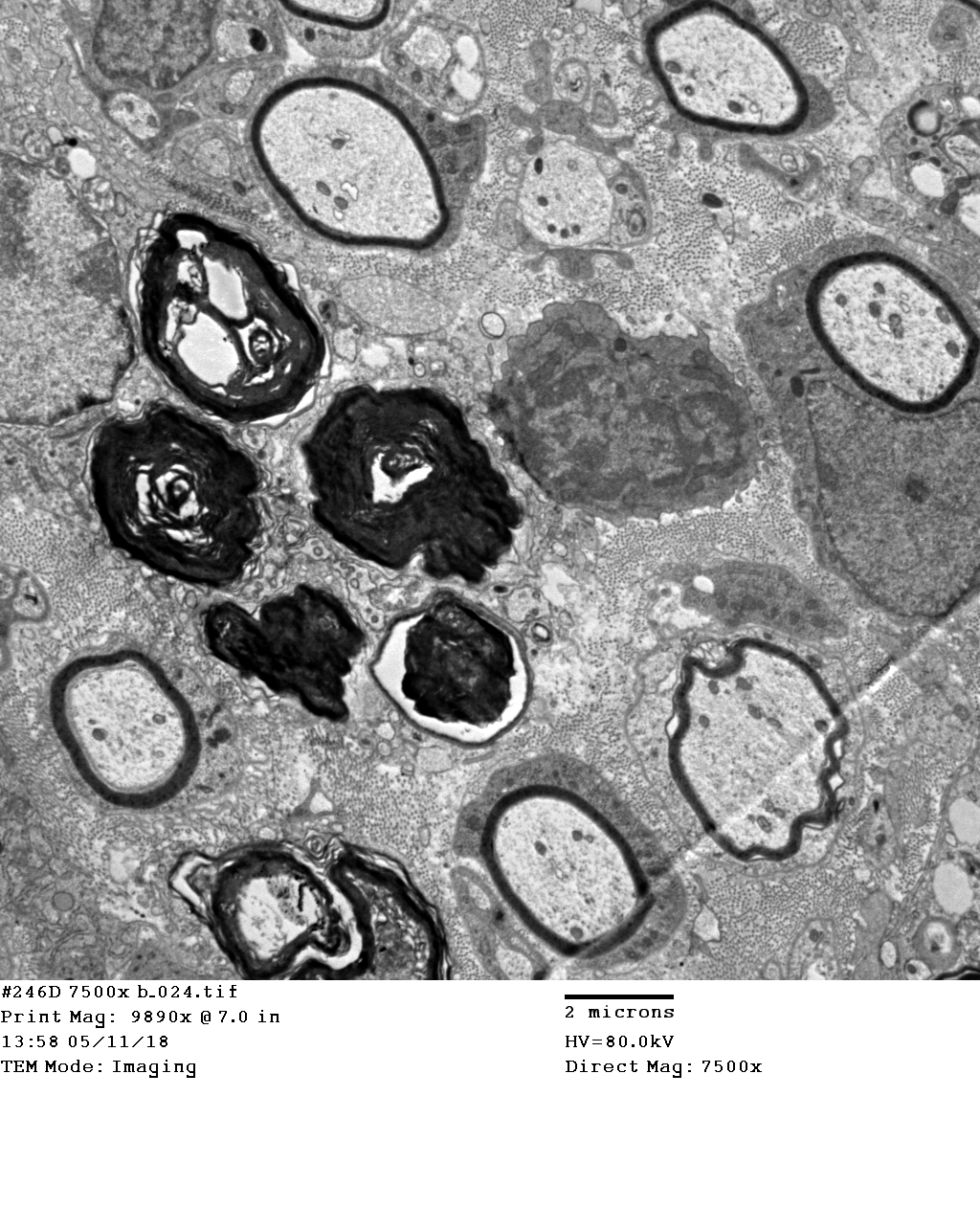

Supplement: Figure 8—source data 1. — This zip archive contains the TEM images for one WT and one Taz iKO used for quantitative analysis shown in Figure 8D–G. Images were taken using a JEOL 1010 electron microscope fitted with a Hamamatsu digital camera and AMT Advantage image capture software. Contrast of the images was adjusted using Photoshop software. [file elife-50138-fig8-data1.zip › Figure 8 source data 1/WT #246D 7500x b/#246D 7500x b_024 adjusted.tif]

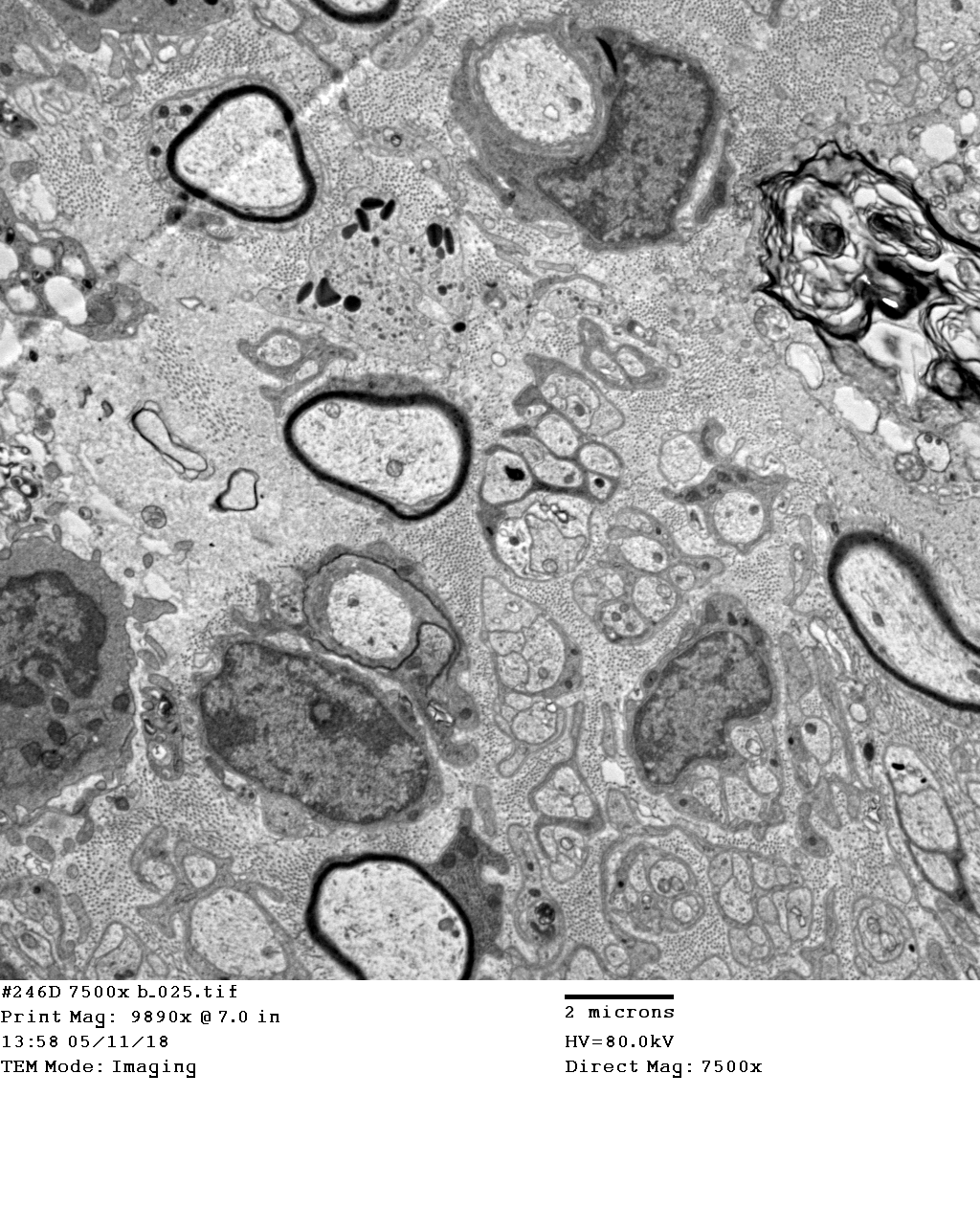

Supplement: Figure 8—source data 1. — This zip archive contains the TEM images for one WT and one Taz iKO used for quantitative analysis shown in Figure 8D–G. Images were taken using a JEOL 1010 electron microscope fitted with a Hamamatsu digital camera and AMT Advantage image capture software. Contrast of the images was adjusted using Photoshop software. [file elife-50138-fig8-data1.zip › Figure 8 source data 1/WT #246D 7500x b/#246D 7500x b_025 adjusted.tif]

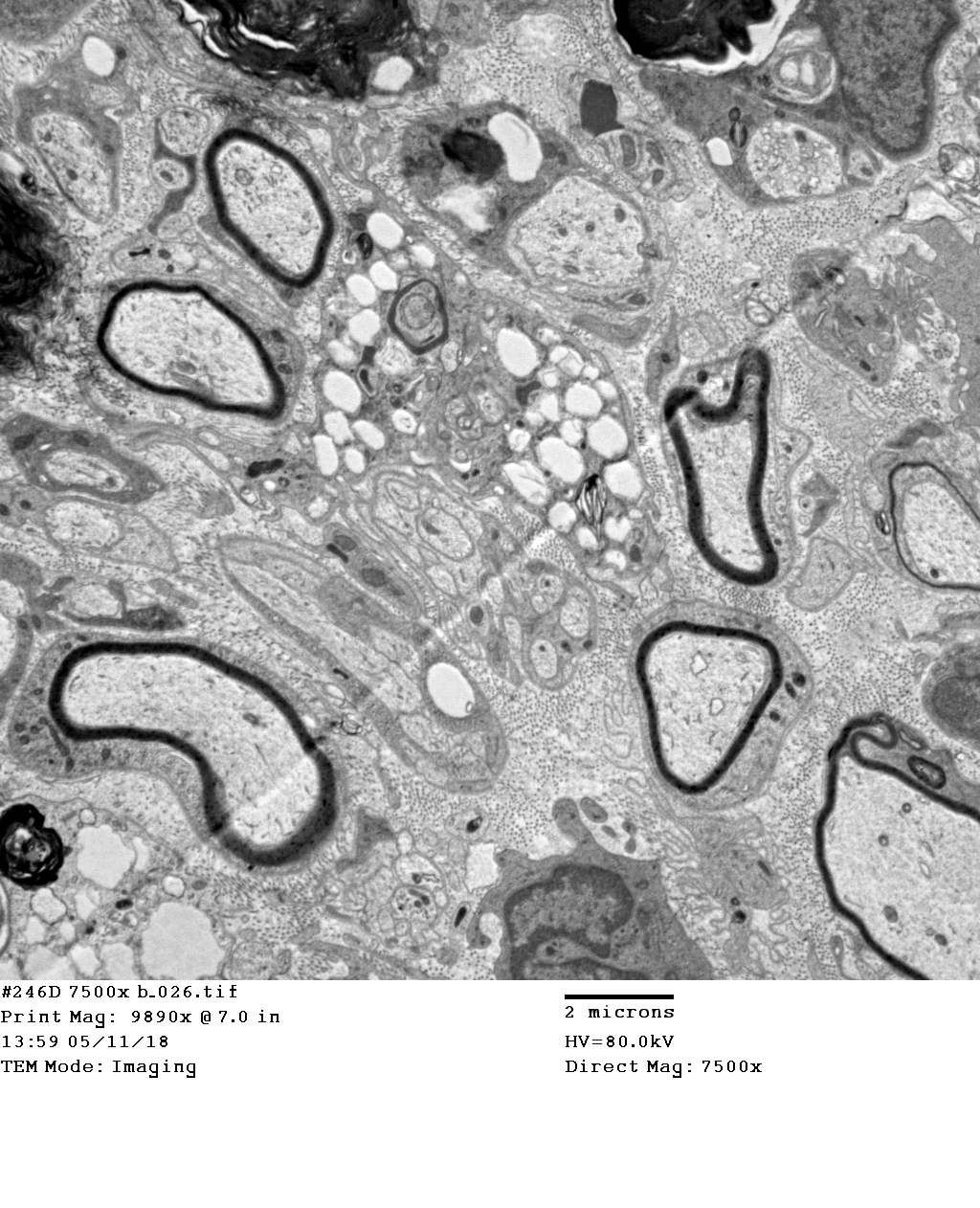

Supplement: Figure 8—source data 1. — This zip archive contains the TEM images for one WT and one Taz iKO used for quantitative analysis shown in Figure 8D–G. Images were taken using a JEOL 1010 electron microscope fitted with a Hamamatsu digital camera and AMT Advantage image capture software. Contrast of the images was adjusted using Photoshop software. [file elife-50138-fig8-data1.zip › Figure 8 source data 1/WT #246D 7500x b/#246D 7500x b_026 adjusted.tif]

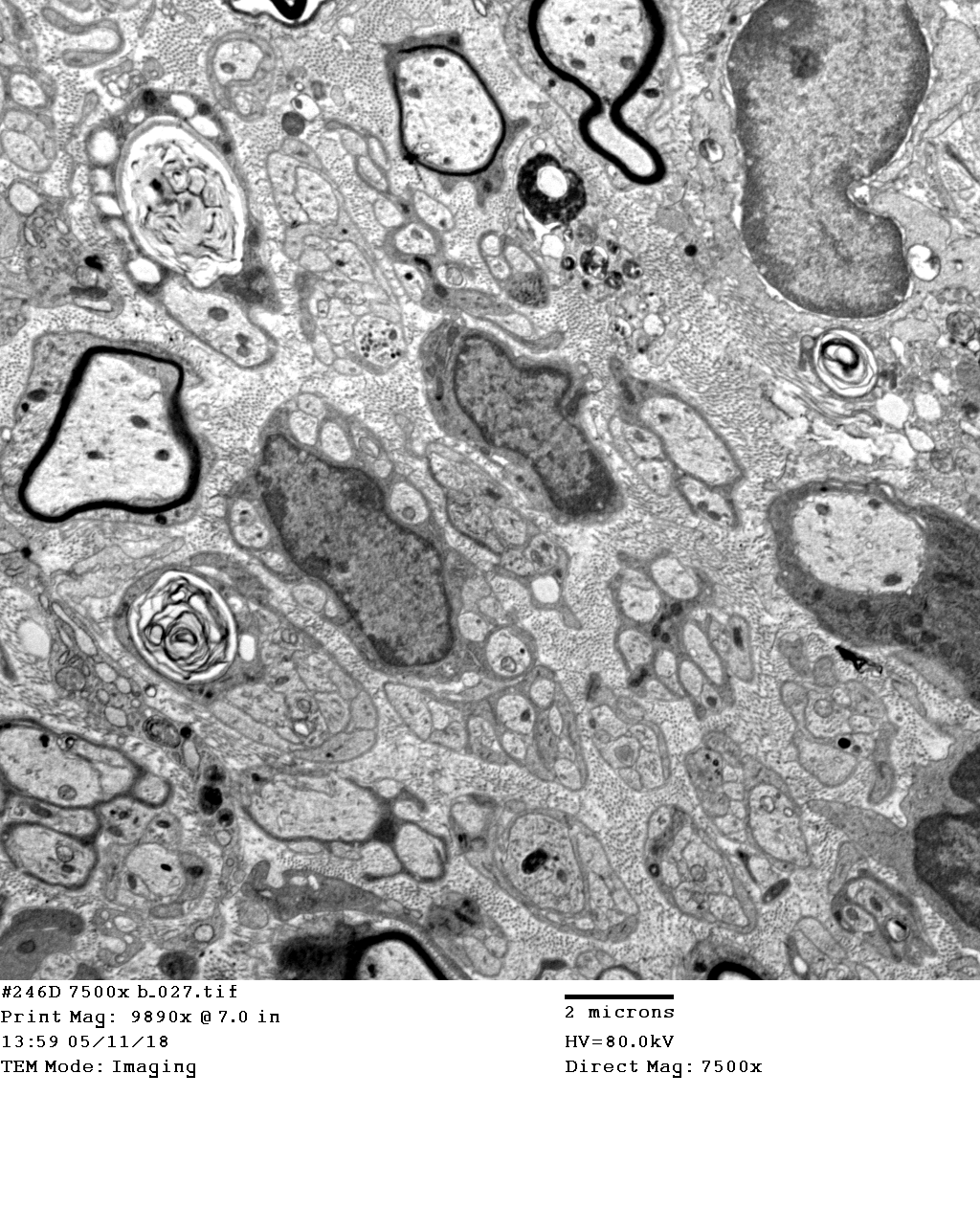

Supplement: Figure 8—source data 1. — This zip archive contains the TEM images for one WT and one Taz iKO used for quantitative analysis shown in Figure 8D–G. Images were taken using a JEOL 1010 electron microscope fitted with a Hamamatsu digital camera and AMT Advantage image capture software. Contrast of the images was adjusted using Photoshop software. [file elife-50138-fig8-data1.zip › Figure 8 source data 1/WT #246D 7500x b/#246D 7500x b_027 adjusted.tif]

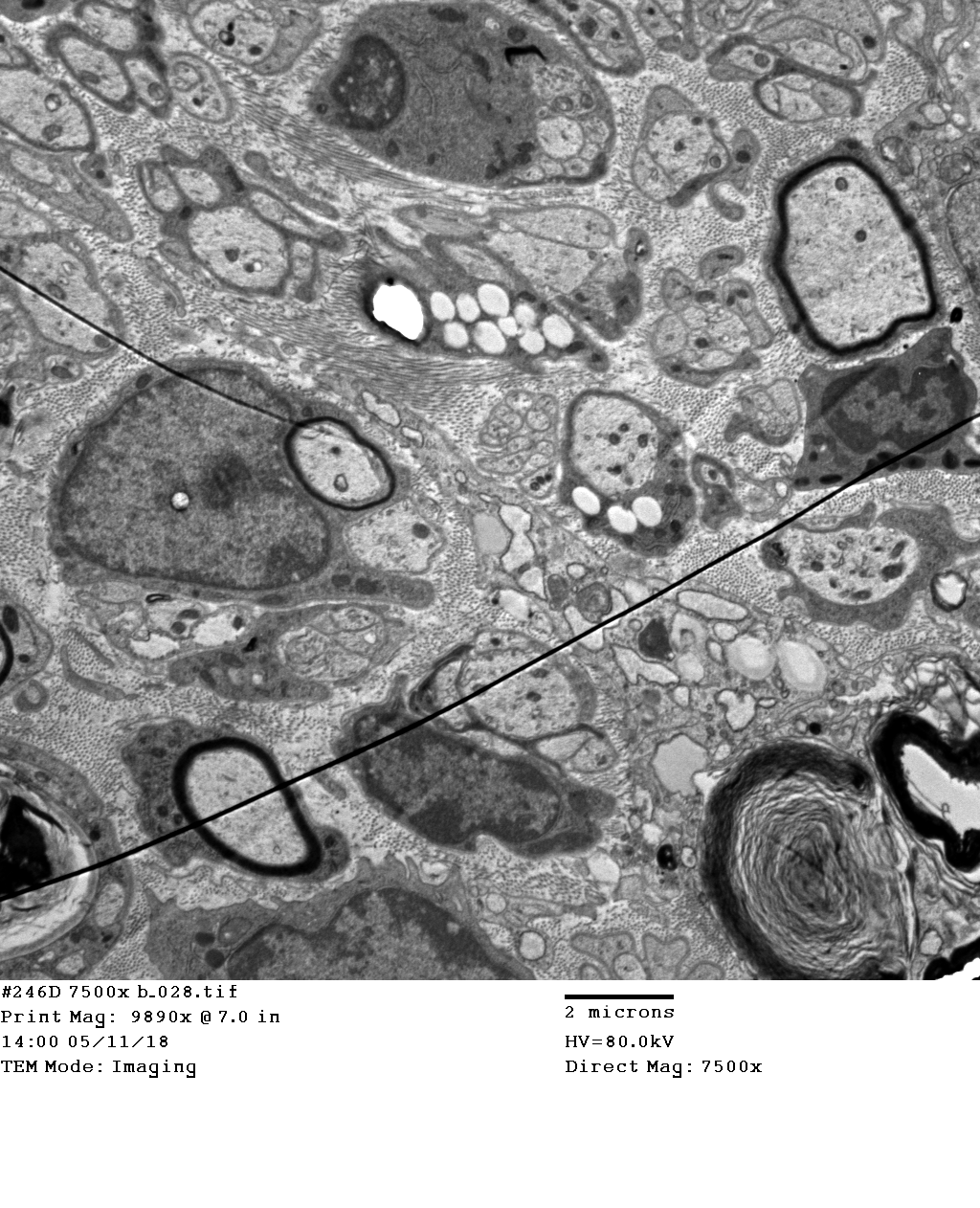

Supplement: Figure 8—source data 1. — This zip archive contains the TEM images for one WT and one Taz iKO used for quantitative analysis shown in Figure 8D–G. Images were taken using a JEOL 1010 electron microscope fitted with a Hamamatsu digital camera and AMT Advantage image capture software. Contrast of the images was adjusted using Photoshop software. [file elife-50138-fig8-data1.zip › Figure 8 source data 1/WT #246D 7500x b/#246D 7500x b_028 adjusted.tif]

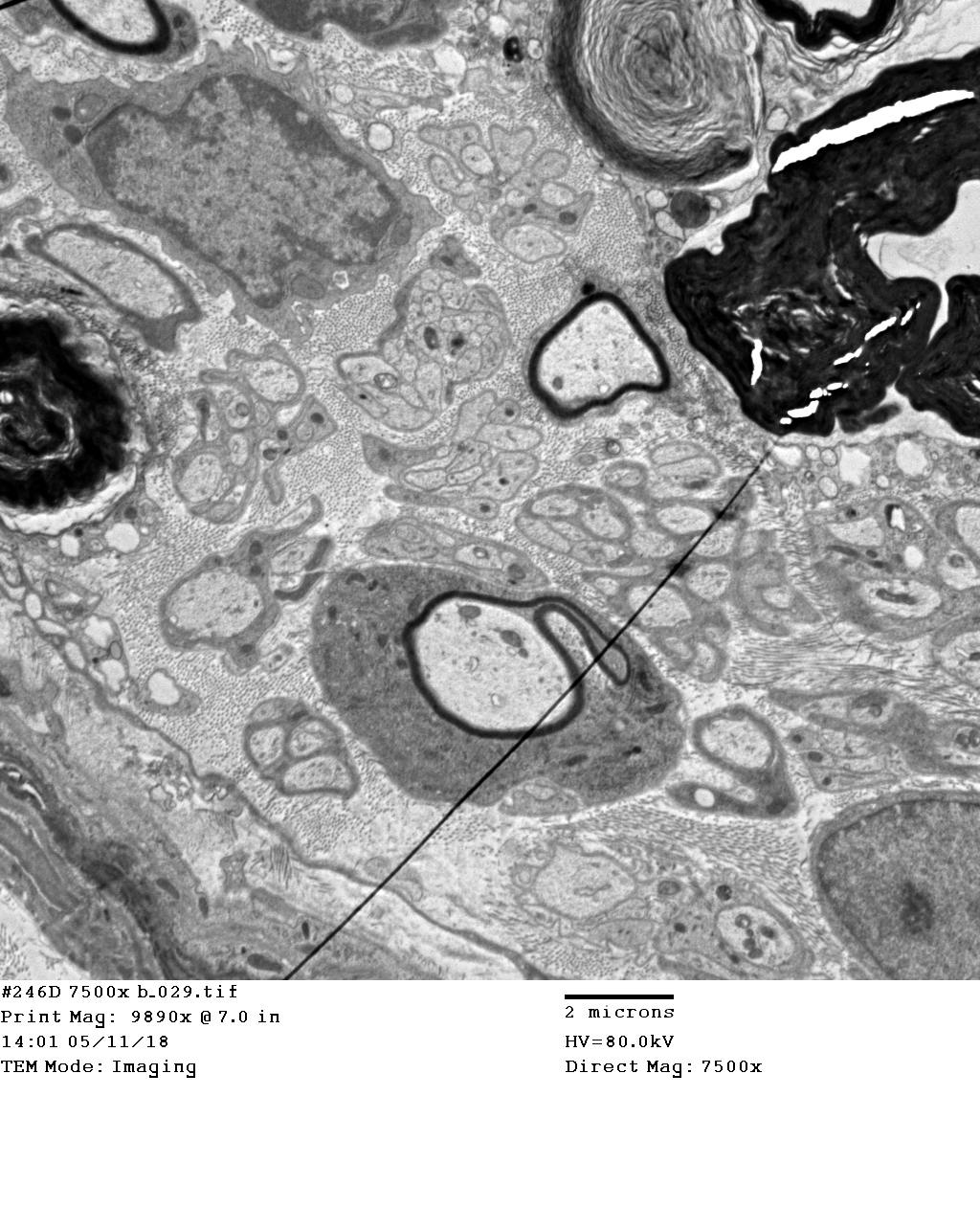

Supplement: Figure 8—source data 1. — This zip archive contains the TEM images for one WT and one Taz iKO used for quantitative analysis shown in Figure 8D–G. Images were taken using a JEOL 1010 electron microscope fitted with a Hamamatsu digital camera and AMT Advantage image capture software. Contrast of the images was adjusted using Photoshop software. [file elife-50138-fig8-data1.zip › Figure 8 source data 1/WT #246D 7500x b/#246D 7500x b_029 adjusted.tif]

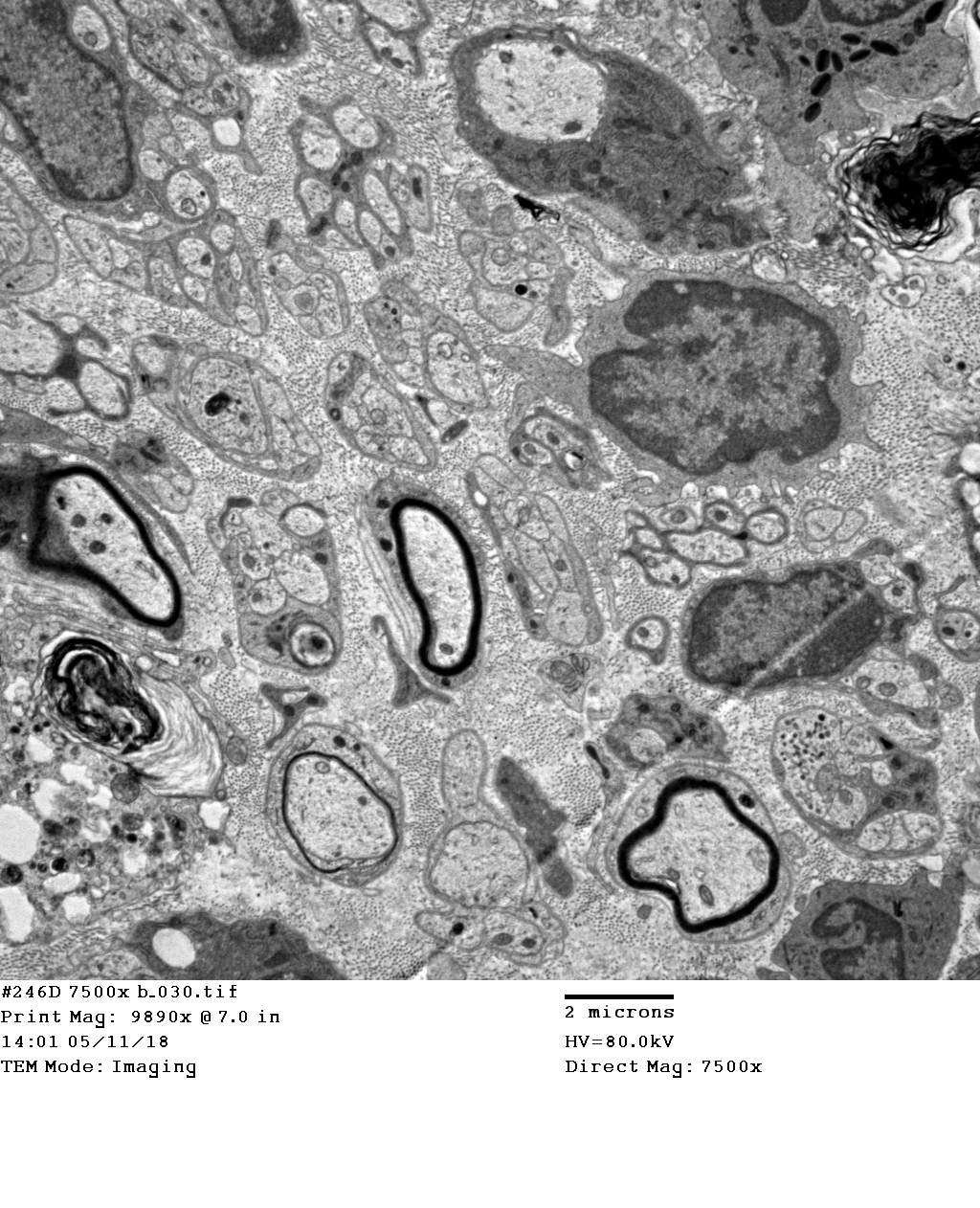

Supplement: Figure 8—source data 1. — This zip archive contains the TEM images for one WT and one Taz iKO used for quantitative analysis shown in Figure 8D–G. Images were taken using a JEOL 1010 electron microscope fitted with a Hamamatsu digital camera and AMT Advantage image capture software. Contrast of the images was adjusted using Photoshop software. [file elife-50138-fig8-data1.zip › Figure 8 source data 1/WT #246D 7500x b/#246D 7500x b_030 adjusted.tif]

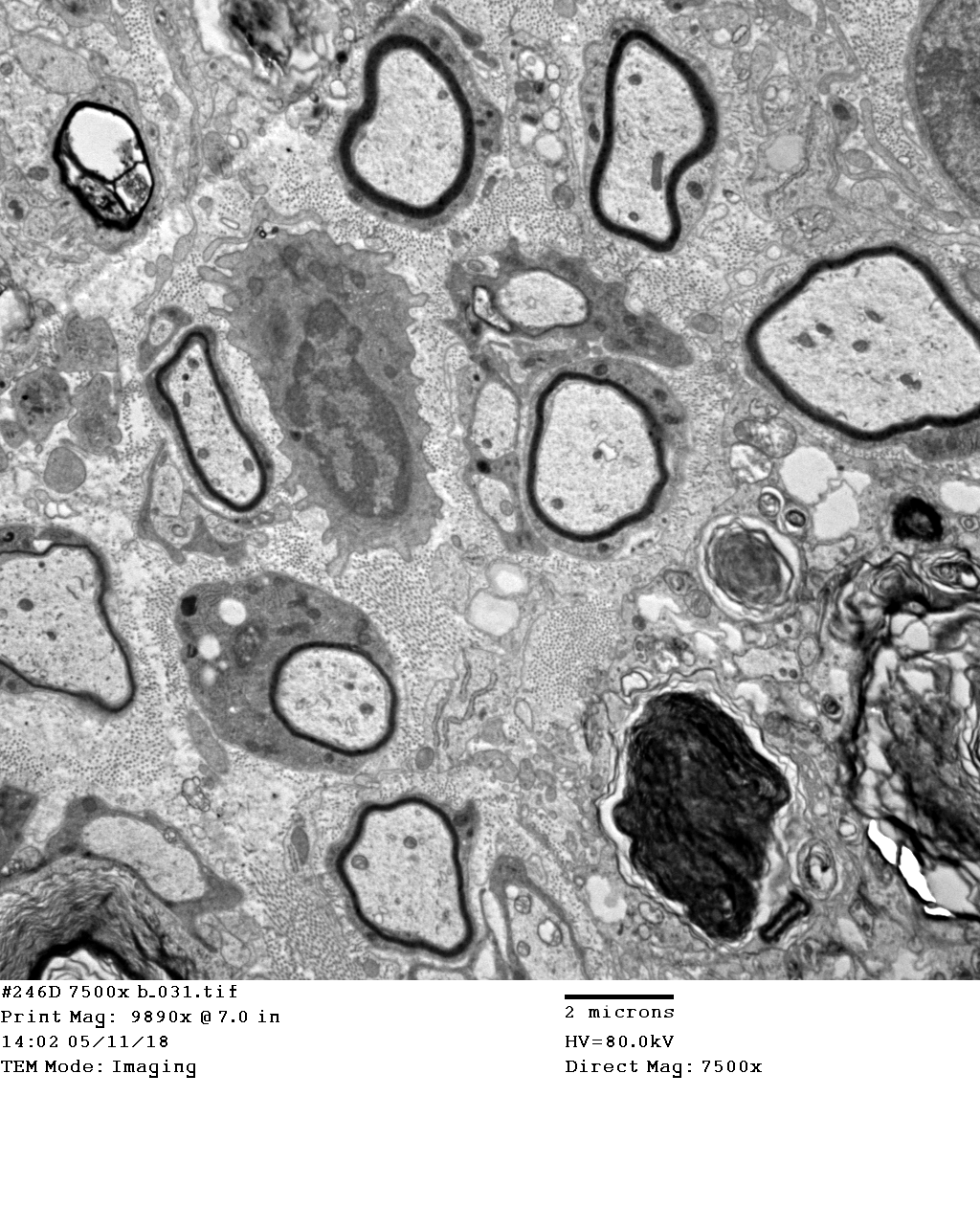

Supplement: Figure 8—source data 1. — This zip archive contains the TEM images for one WT and one Taz iKO used for quantitative analysis shown in Figure 8D–G. Images were taken using a JEOL 1010 electron microscope fitted with a Hamamatsu digital camera and AMT Advantage image capture software. Contrast of the images was adjusted using Photoshop software. [file elife-50138-fig8-data1.zip › Figure 8 source data 1/WT #246D 7500x b/#246D 7500x b_031 adjusted.tif]

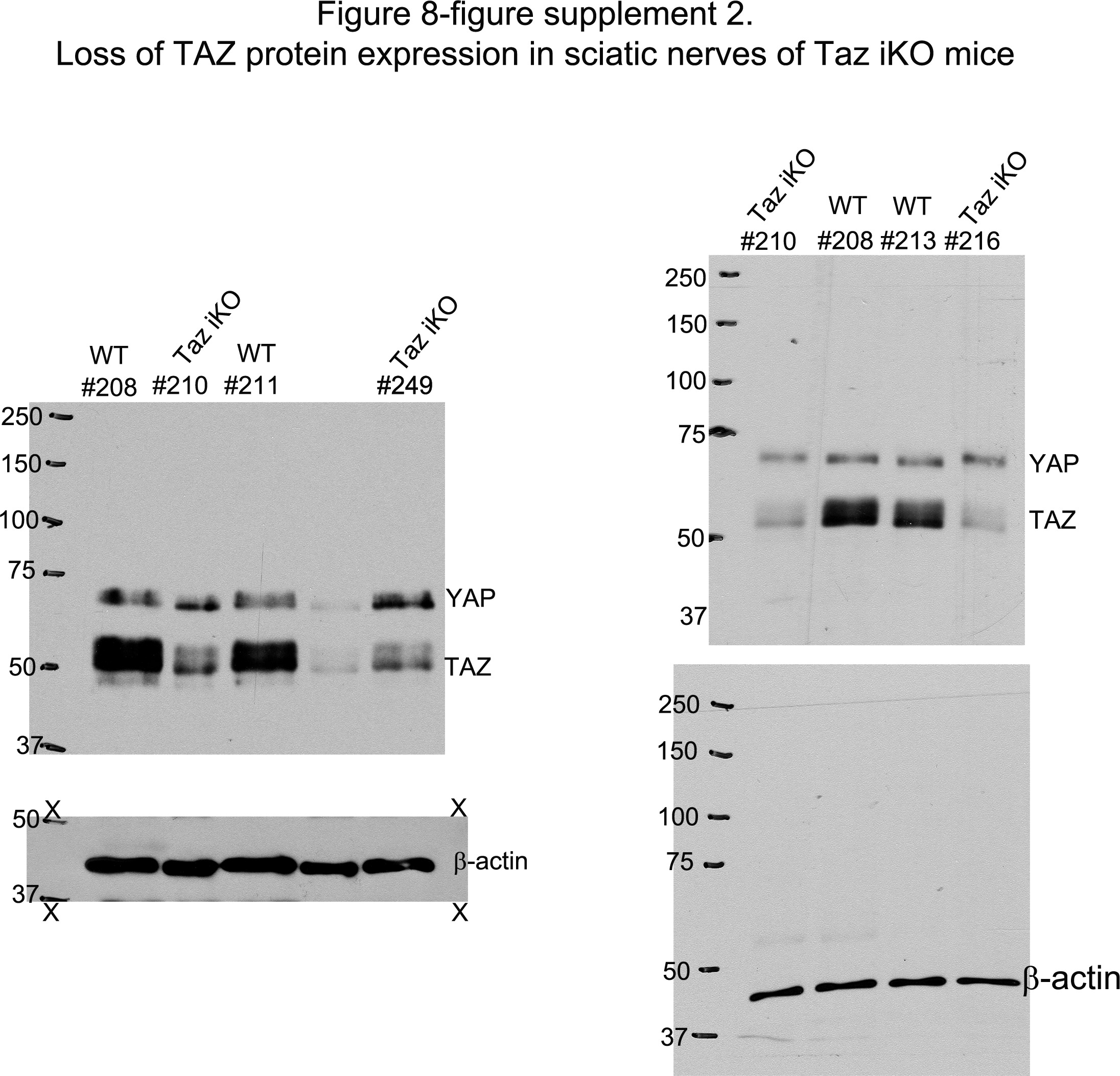

Supplement: Figure 8—source data 4. — Uncropped Western blots of images used to make Figure 8A. Individually processed samples from 3 WT mice (#208,#211,#213) and 3 Taz iKO mice (#210,#216,#249) are shown and used for quantification. X…….X denotes the line along which membranes were cut prior to probing with the relevant antibodies. The following figure supplements are available for Figure 8. [file elife-50138-fig8-data4.jpg]

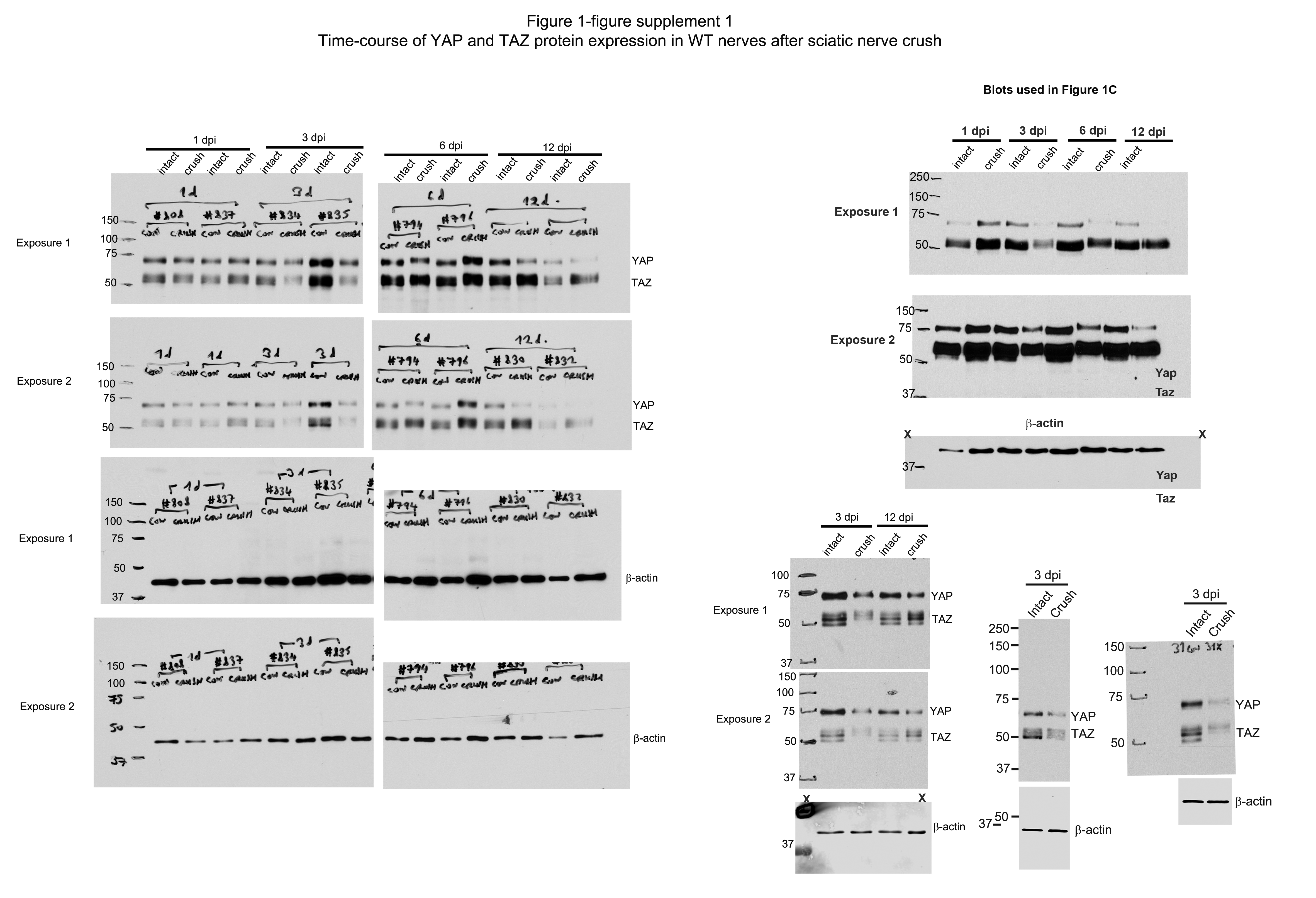

Supplement: Figure 8—figure supplement 1—source data 1. — This zip archive contains the raw data for WT and Taz iKO used for the quantitative analysis shown in Figure 8—figure supplement 1E. The data are contained in both a text document and an Excel file, both labeled as Mann Whitney data. These files also contain data for Figure 3—figure supplement 1, Figures 3, 4, 6, 7, 8. [file elife-50138-fig8-figsupp1-data1.zip › Figure 1figure supplement 1 R2.tif]

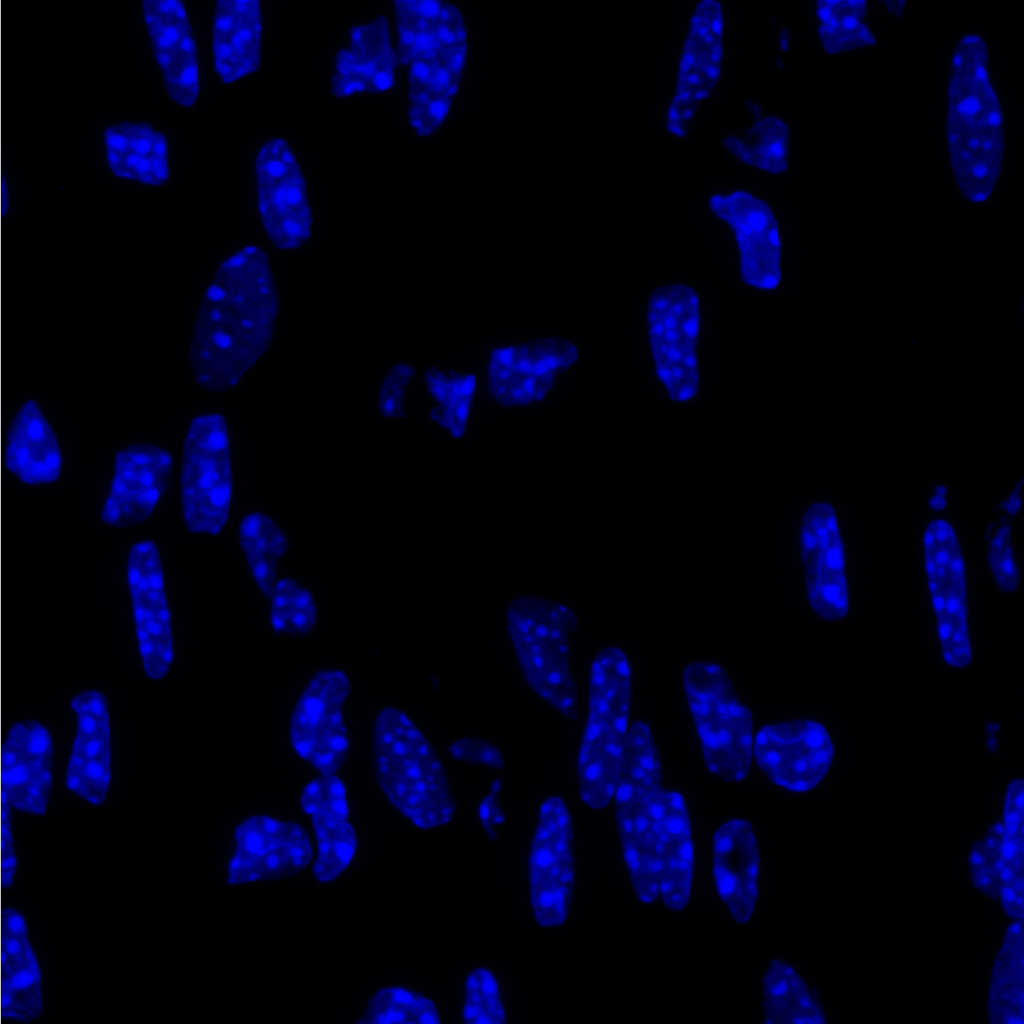

Supplement: Figure 9—source data 1. — This zip archive contains the IHC for one WT and one iDKO used for quantitative analysis shown in Figure 9E. Leica SP8 confocal lif images were processed using Imaris software and saved as tiffs. [file elife-50138-fig9-data1.zip › Figure 9 source data 1/iDKO #492 Krox20/Series 6 DAPI.tif]

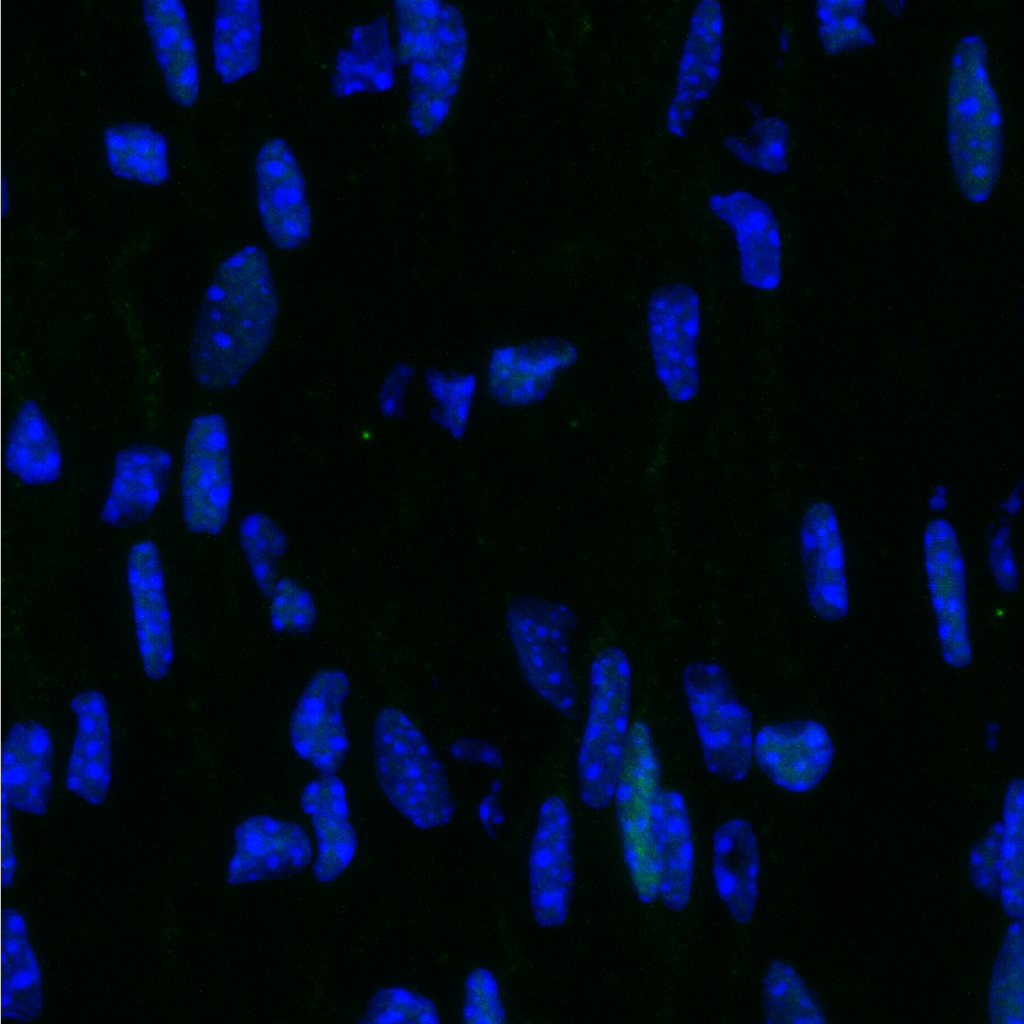

Supplement: Figure 9—source data 1. — This zip archive contains the IHC for one WT and one iDKO used for quantitative analysis shown in Figure 9E. Leica SP8 confocal lif images were processed using Imaris software and saved as tiffs. [file elife-50138-fig9-data1.zip › Figure 9 source data 1/iDKO #492 Krox20/Series 6 Krox20 + DAPI.tif]

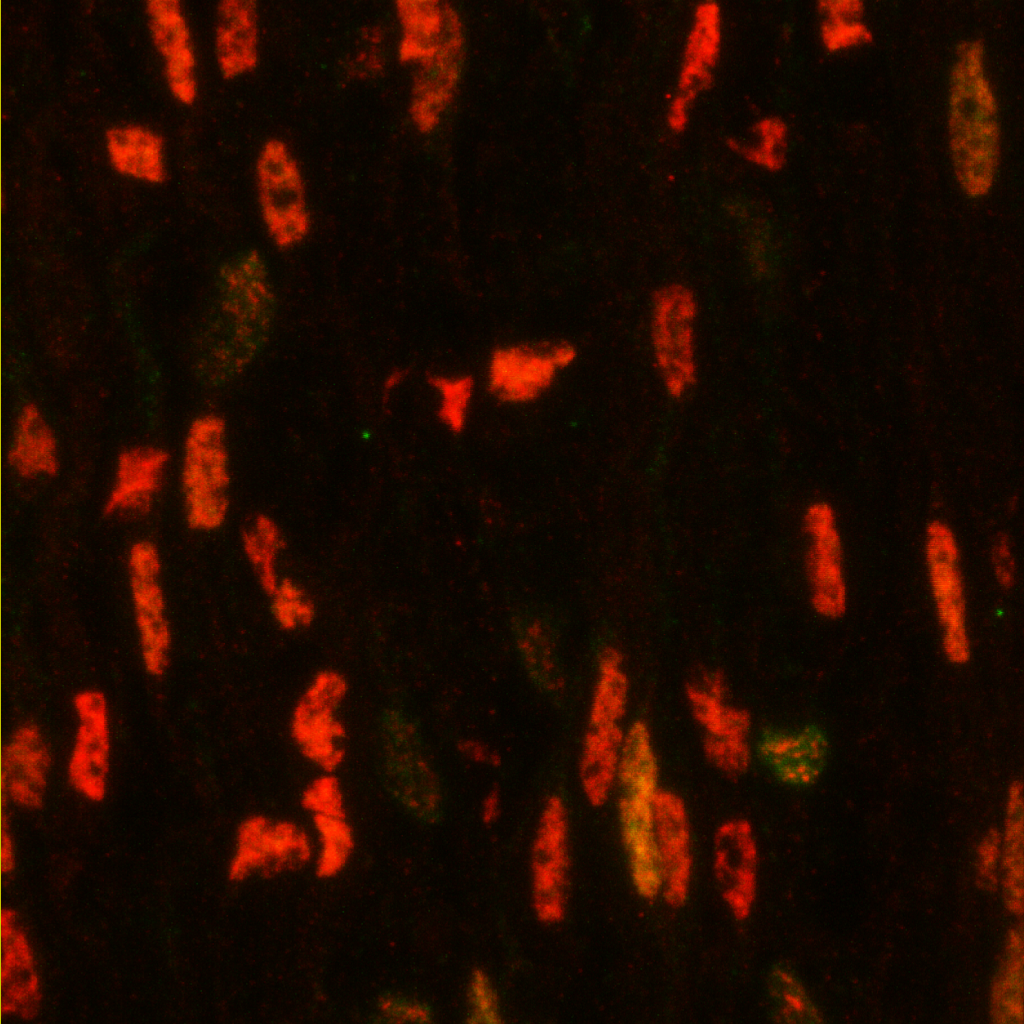

Supplement: Figure 9—source data 1. — This zip archive contains the IHC for one WT and one iDKO used for quantitative analysis shown in Figure 9E. Leica SP8 confocal lif images were processed using Imaris software and saved as tiffs. [file elife-50138-fig9-data1.zip › Figure 9 source data 1/iDKO #492 Krox20/Series 6 Krox20 + Sox10.tif]

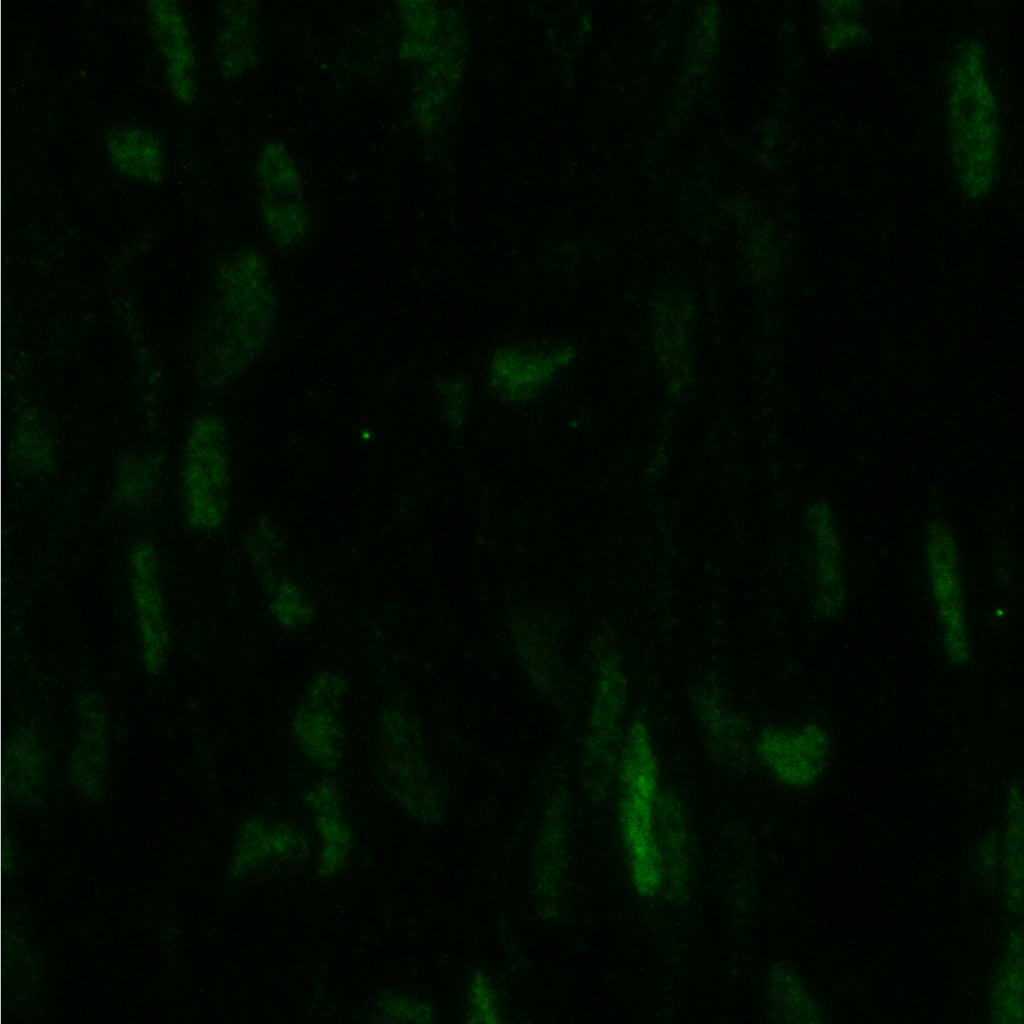

Supplement: Figure 9—source data 1. — This zip archive contains the IHC for one WT and one iDKO used for quantitative analysis shown in Figure 9E. Leica SP8 confocal lif images were processed using Imaris software and saved as tiffs. [file elife-50138-fig9-data1.zip › Figure 9 source data 1/iDKO #492 Krox20/Series 6 Krox20.tif]

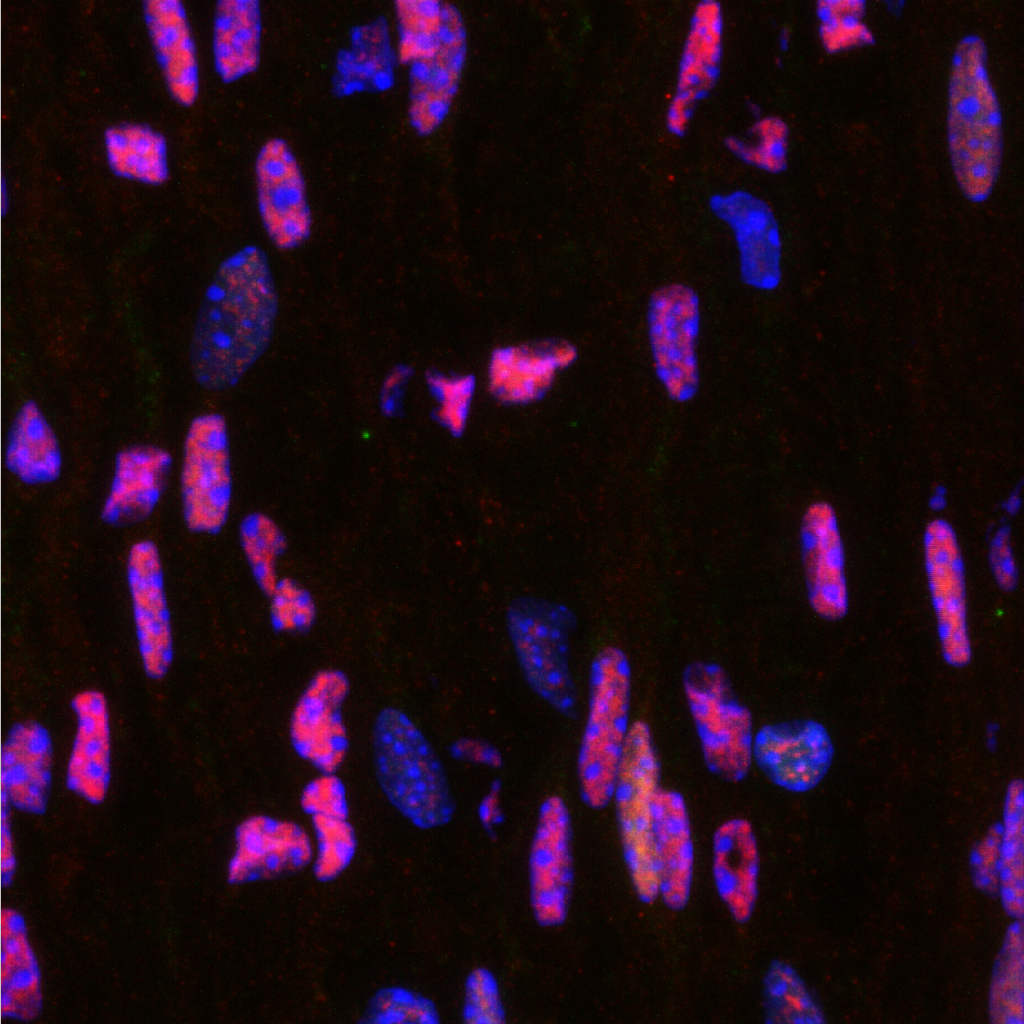

Supplement: Figure 9—source data 1. — This zip archive contains the IHC for one WT and one iDKO used for quantitative analysis shown in Figure 9E. Leica SP8 confocal lif images were processed using Imaris software and saved as tiffs. [file elife-50138-fig9-data1.zip › Figure 9 source data 1/iDKO #492 Krox20/Series 6 merge.tif]

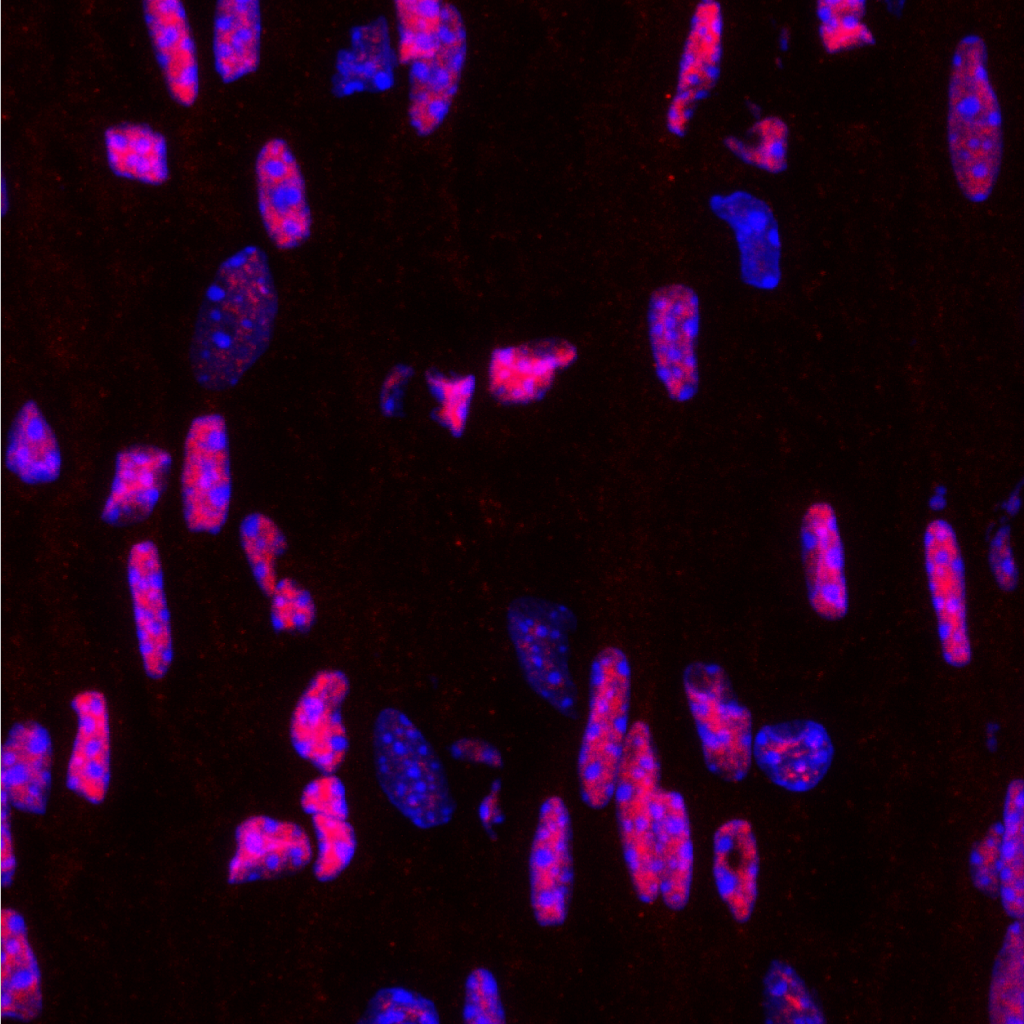

Supplement: Figure 9—source data 1. — This zip archive contains the IHC for one WT and one iDKO used for quantitative analysis shown in Figure 9E. Leica SP8 confocal lif images were processed using Imaris software and saved as tiffs. [file elife-50138-fig9-data1.zip › Figure 9 source data 1/iDKO #492 Krox20/Series 6 Sox10 + DAPI.tif]

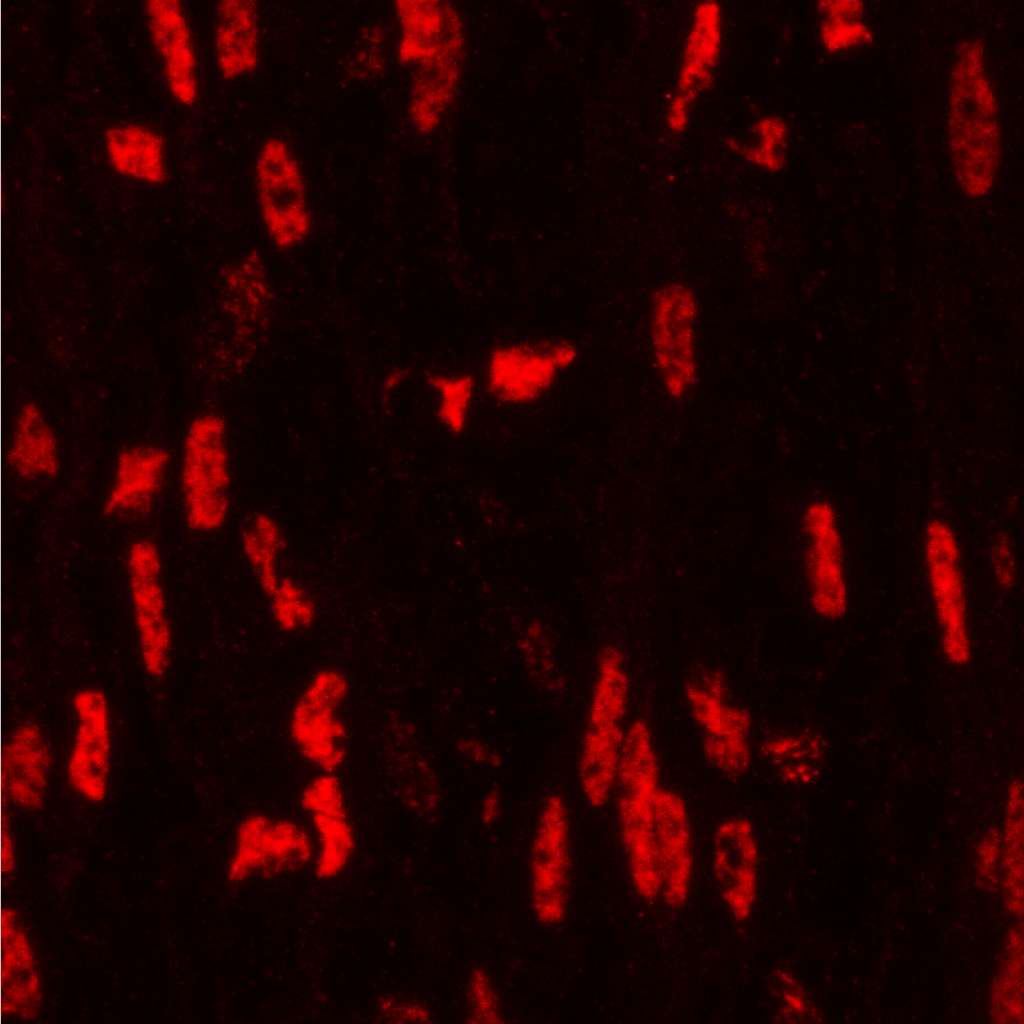

Supplement: Figure 9—source data 1. — This zip archive contains the IHC for one WT and one iDKO used for quantitative analysis shown in Figure 9E. Leica SP8 confocal lif images were processed using Imaris software and saved as tiffs. [file elife-50138-fig9-data1.zip › Figure 9 source data 1/iDKO #492 Krox20/Series 6 Sox10.tif]

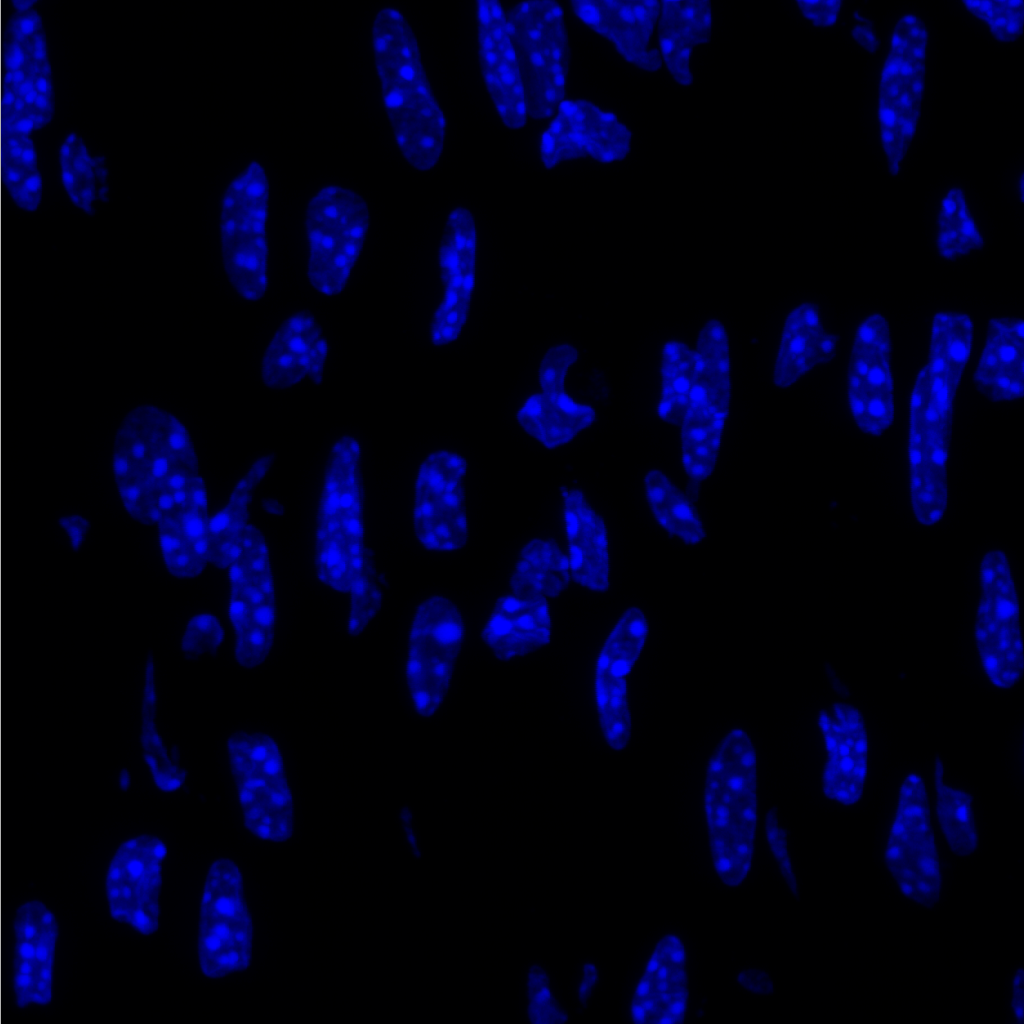

Supplement: Figure 9—source data 1. — This zip archive contains the IHC for one WT and one iDKO used for quantitative analysis shown in Figure 9E. Leica SP8 confocal lif images were processed using Imaris software and saved as tiffs. [file elife-50138-fig9-data1.zip › Figure 9 source data 1/iDKO #492 Krox20/Series 7 DAPI.tif]

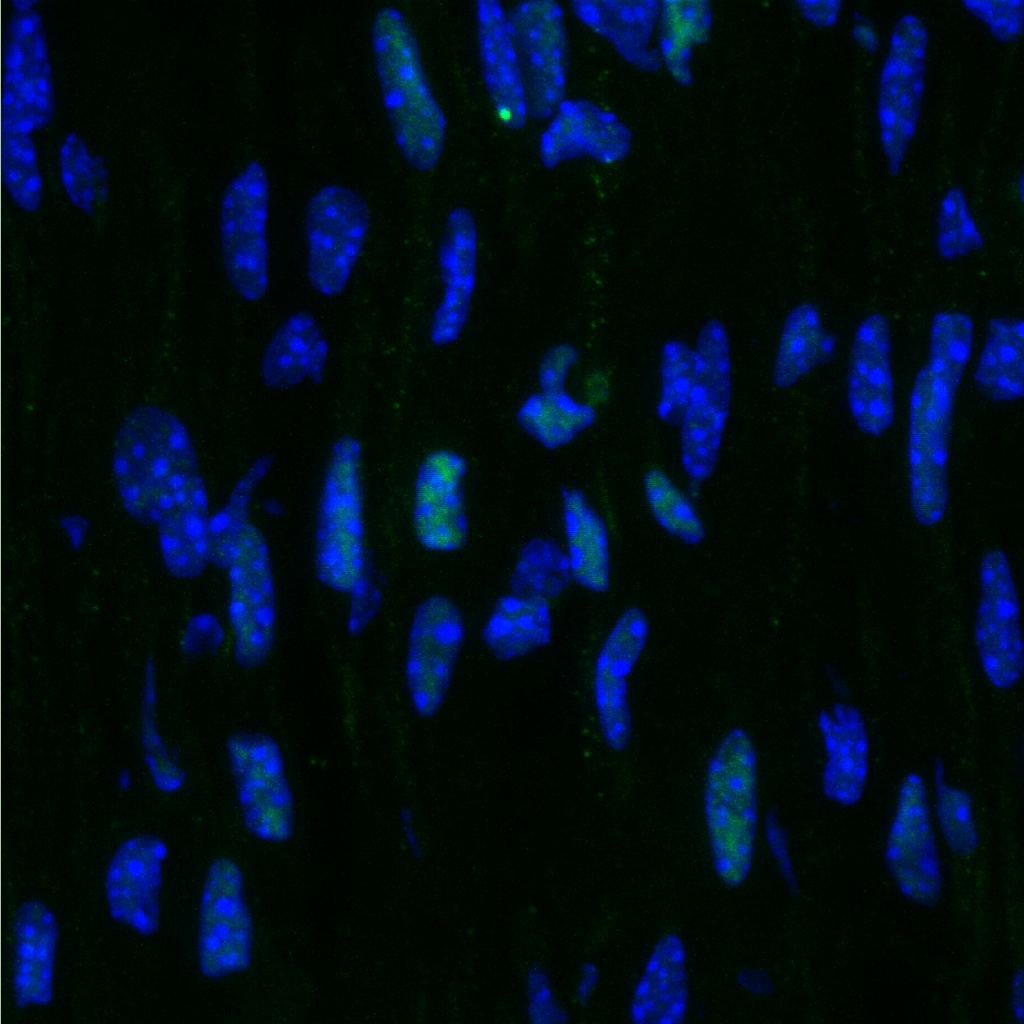

Supplement: Figure 9—source data 1. — This zip archive contains the IHC for one WT and one iDKO used for quantitative analysis shown in Figure 9E. Leica SP8 confocal lif images were processed using Imaris software and saved as tiffs. [file elife-50138-fig9-data1.zip › Figure 9 source data 1/iDKO #492 Krox20/Series 7 Krox20 + DAPI.tif]

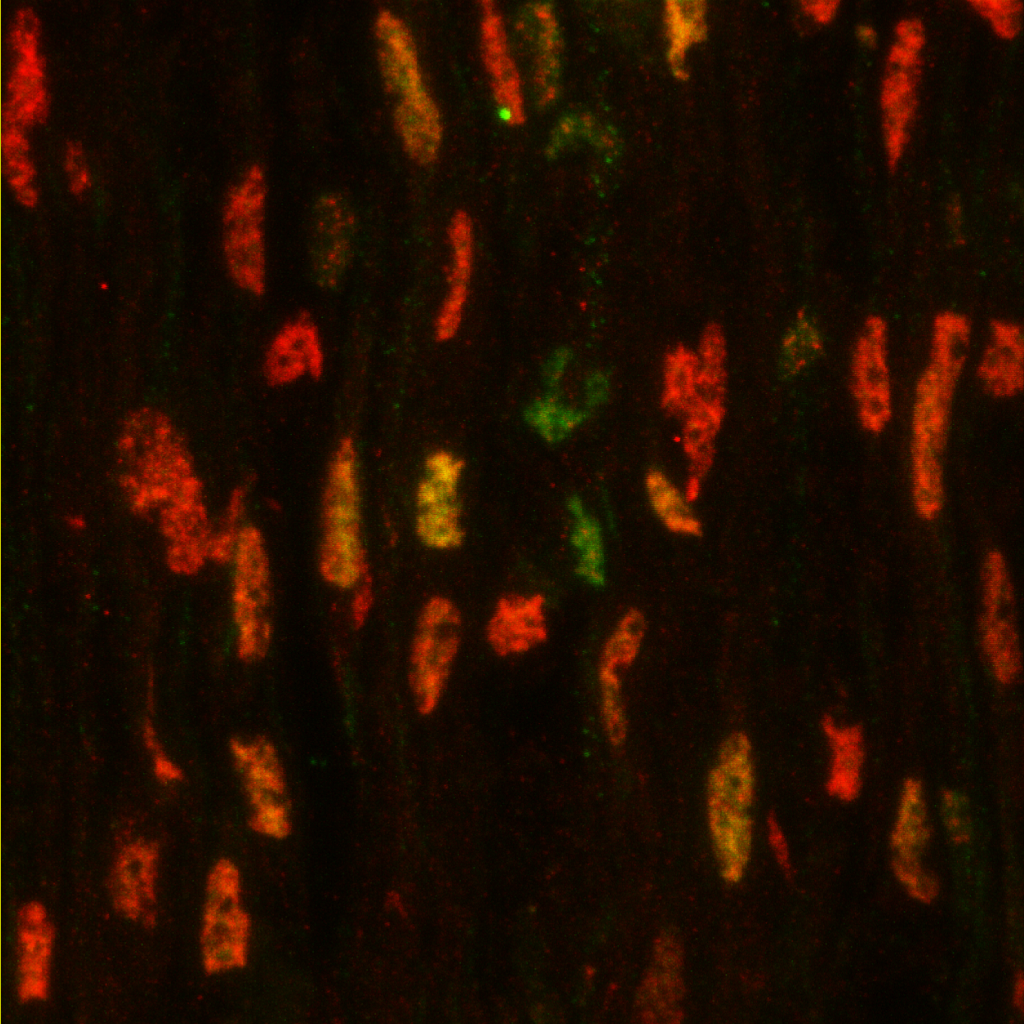

Supplement: Figure 9—source data 1. — This zip archive contains the IHC for one WT and one iDKO used for quantitative analysis shown in Figure 9E. Leica SP8 confocal lif images were processed using Imaris software and saved as tiffs. [file elife-50138-fig9-data1.zip › Figure 9 source data 1/iDKO #492 Krox20/Series 7 Krox20 + Sox10.tif]

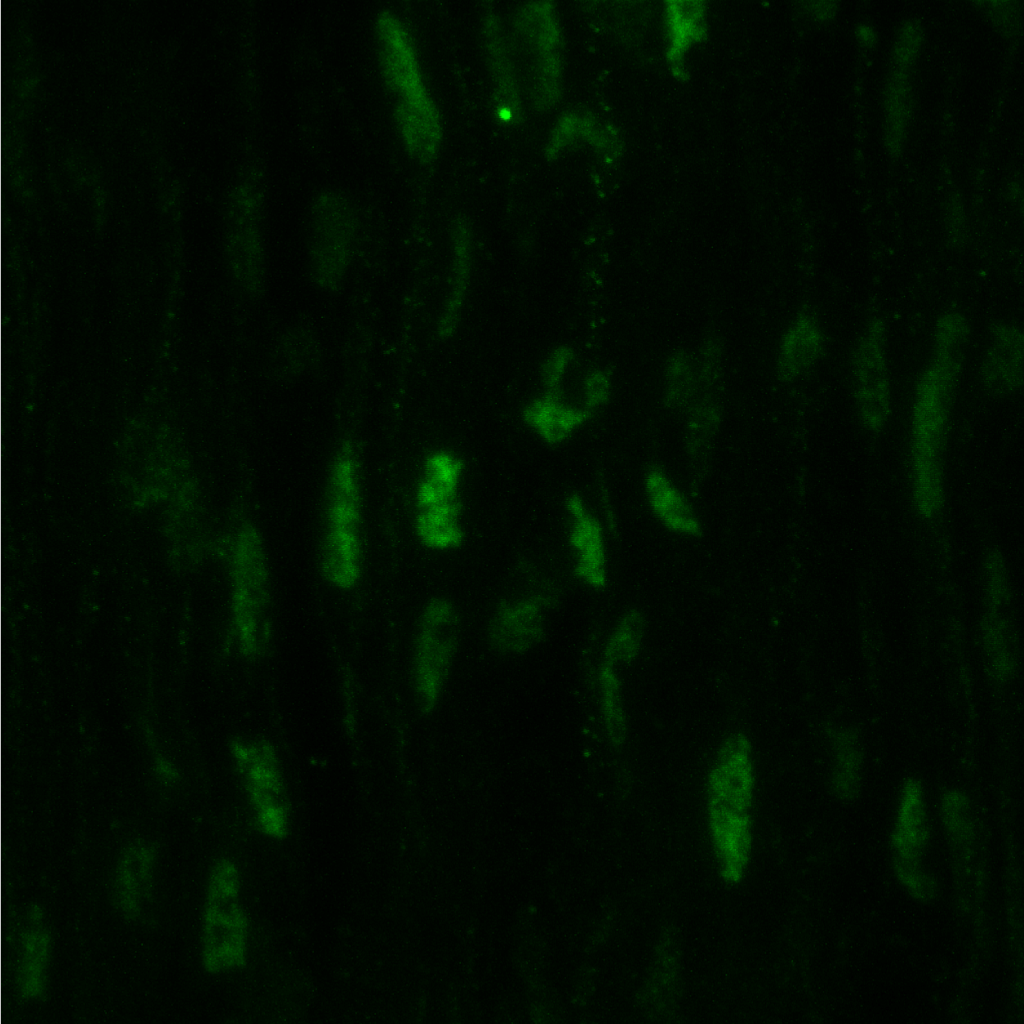

Supplement: Figure 9—source data 1. — This zip archive contains the IHC for one WT and one iDKO used for quantitative analysis shown in Figure 9E. Leica SP8 confocal lif images were processed using Imaris software and saved as tiffs. [file elife-50138-fig9-data1.zip › Figure 9 source data 1/iDKO #492 Krox20/Series 7 Krox20.tif]

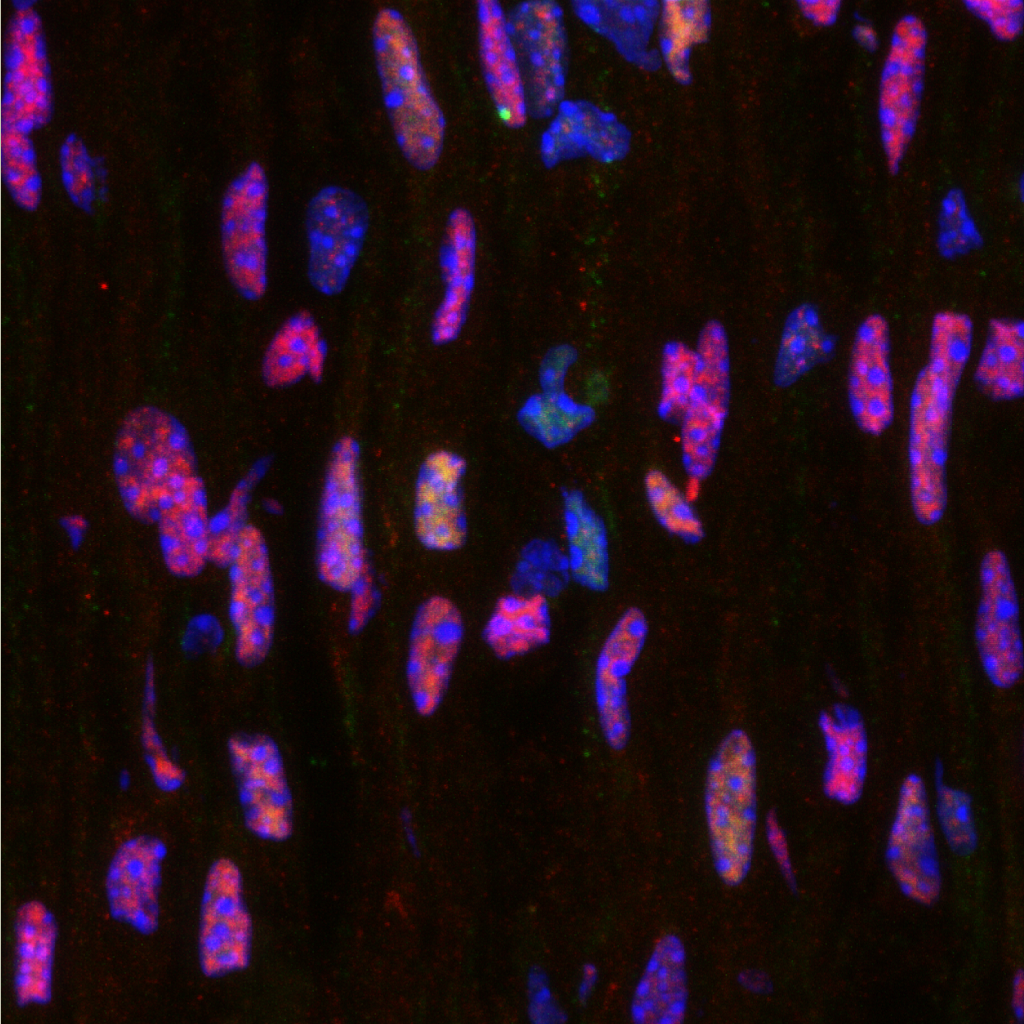

Supplement: Figure 9—source data 1. — This zip archive contains the IHC for one WT and one iDKO used for quantitative analysis shown in Figure 9E. Leica SP8 confocal lif images were processed using Imaris software and saved as tiffs. [file elife-50138-fig9-data1.zip › Figure 9 source data 1/iDKO #492 Krox20/Series 7 merge.tif]

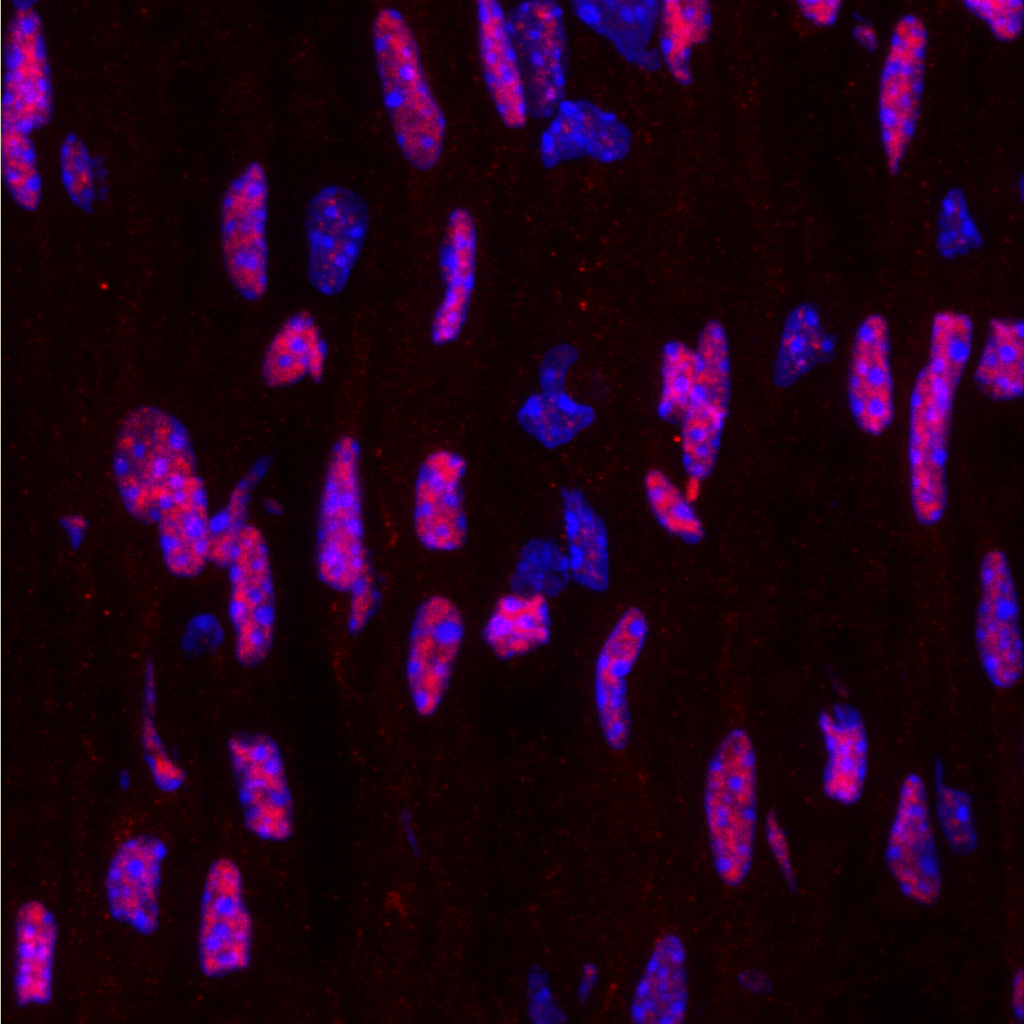

Supplement: Figure 9—source data 1. — This zip archive contains the IHC for one WT and one iDKO used for quantitative analysis shown in Figure 9E. Leica SP8 confocal lif images were processed using Imaris software and saved as tiffs. [file elife-50138-fig9-data1.zip › Figure 9 source data 1/iDKO #492 Krox20/Series 7 Sox10 + DAPI.tif]

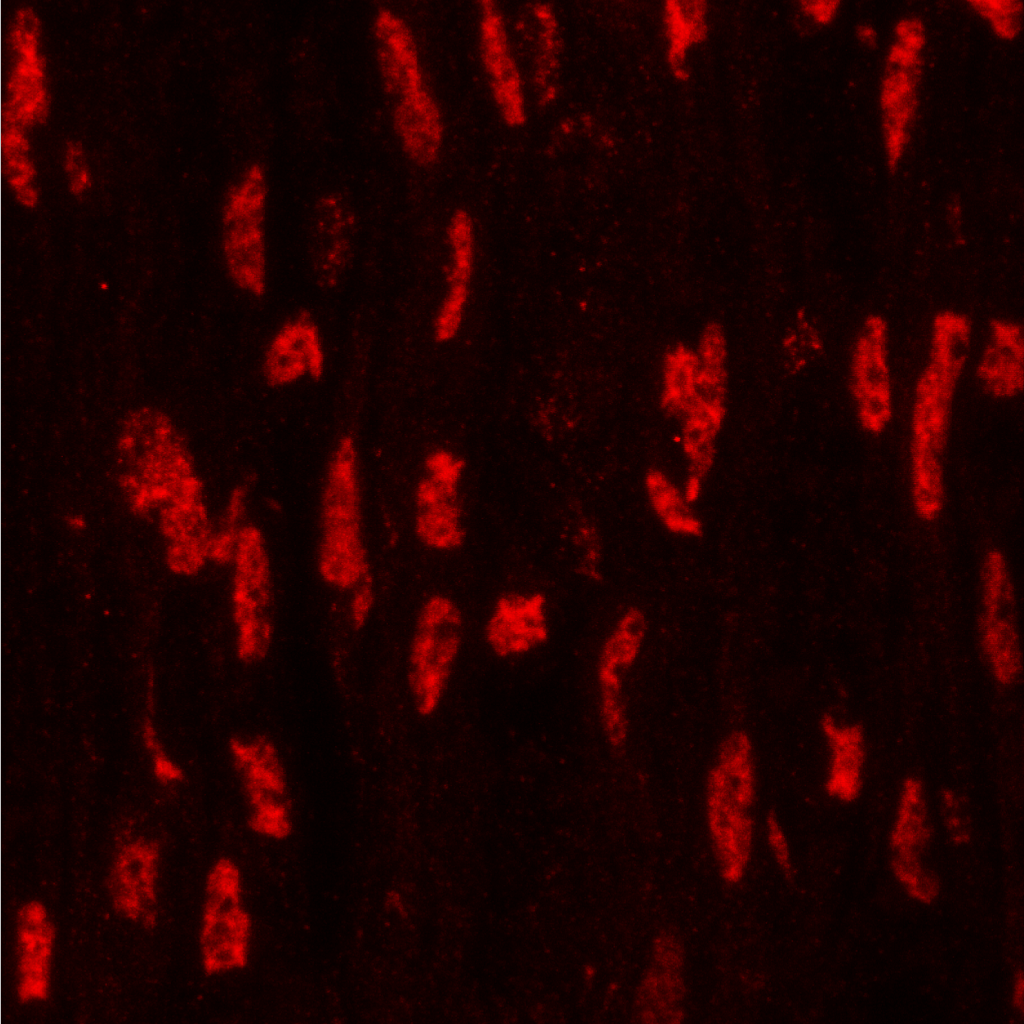

Supplement: Figure 9—source data 1. — This zip archive contains the IHC for one WT and one iDKO used for quantitative analysis shown in Figure 9E. Leica SP8 confocal lif images were processed using Imaris software and saved as tiffs. [file elife-50138-fig9-data1.zip › Figure 9 source data 1/iDKO #492 Krox20/Series 7 Sox10.tif]

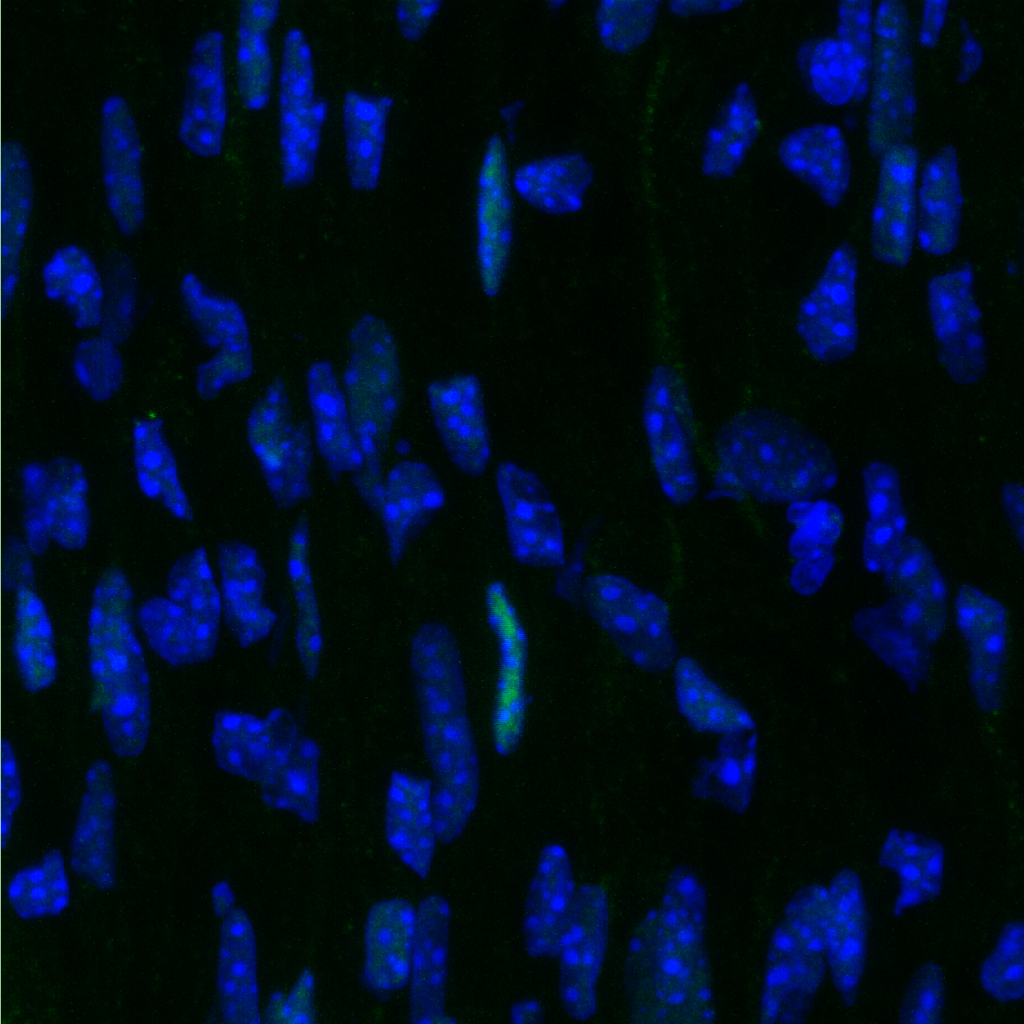

Supplement: Figure 9—source data 1. — This zip archive contains the IHC for one WT and one iDKO used for quantitative analysis shown in Figure 9E. Leica SP8 confocal lif images were processed using Imaris software and saved as tiffs. [file elife-50138-fig9-data1.zip › Figure 9 source data 1/iDKO #492 Krox20/Series 8 Krox20 + DAPI.tif]

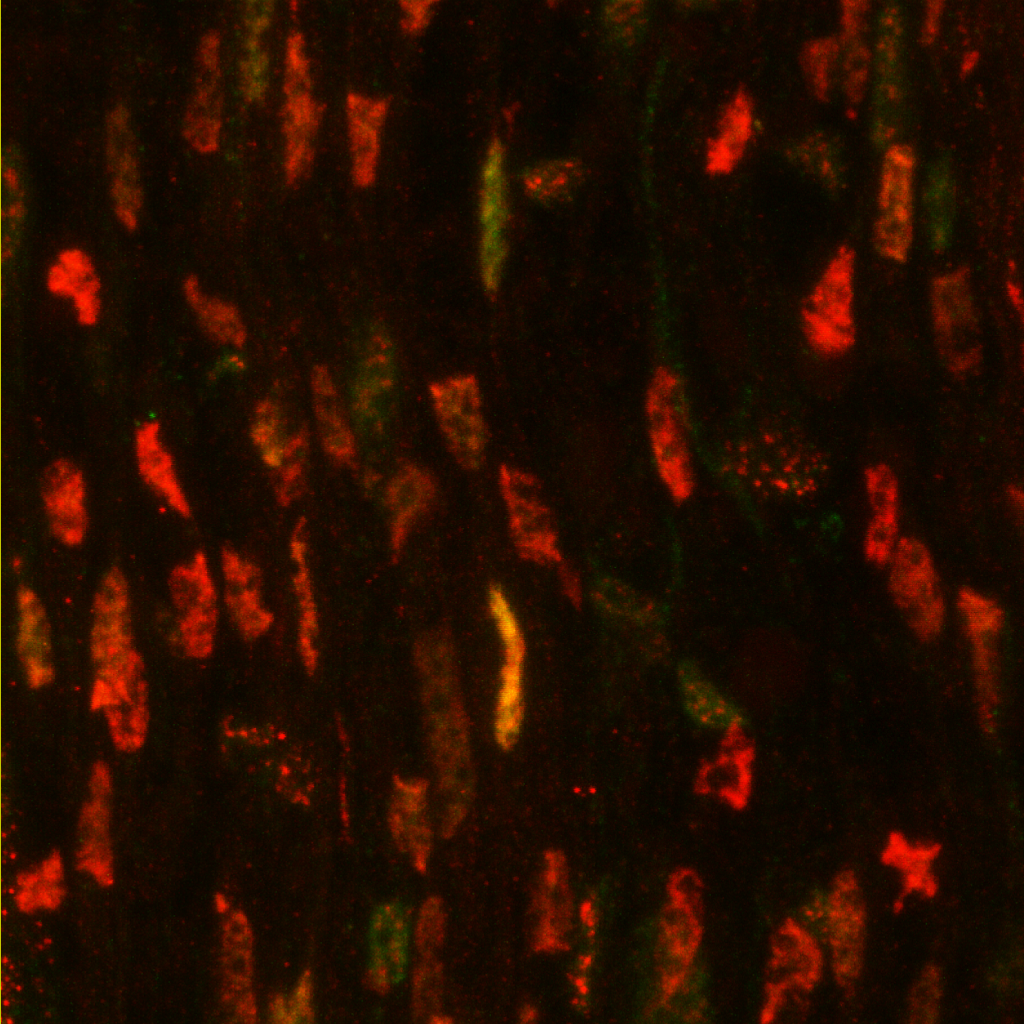

Supplement: Figure 9—source data 1. — This zip archive contains the IHC for one WT and one iDKO used for quantitative analysis shown in Figure 9E. Leica SP8 confocal lif images were processed using Imaris software and saved as tiffs. [file elife-50138-fig9-data1.zip › Figure 9 source data 1/iDKO #492 Krox20/Series 8 Krox20 + Sox10.tif]

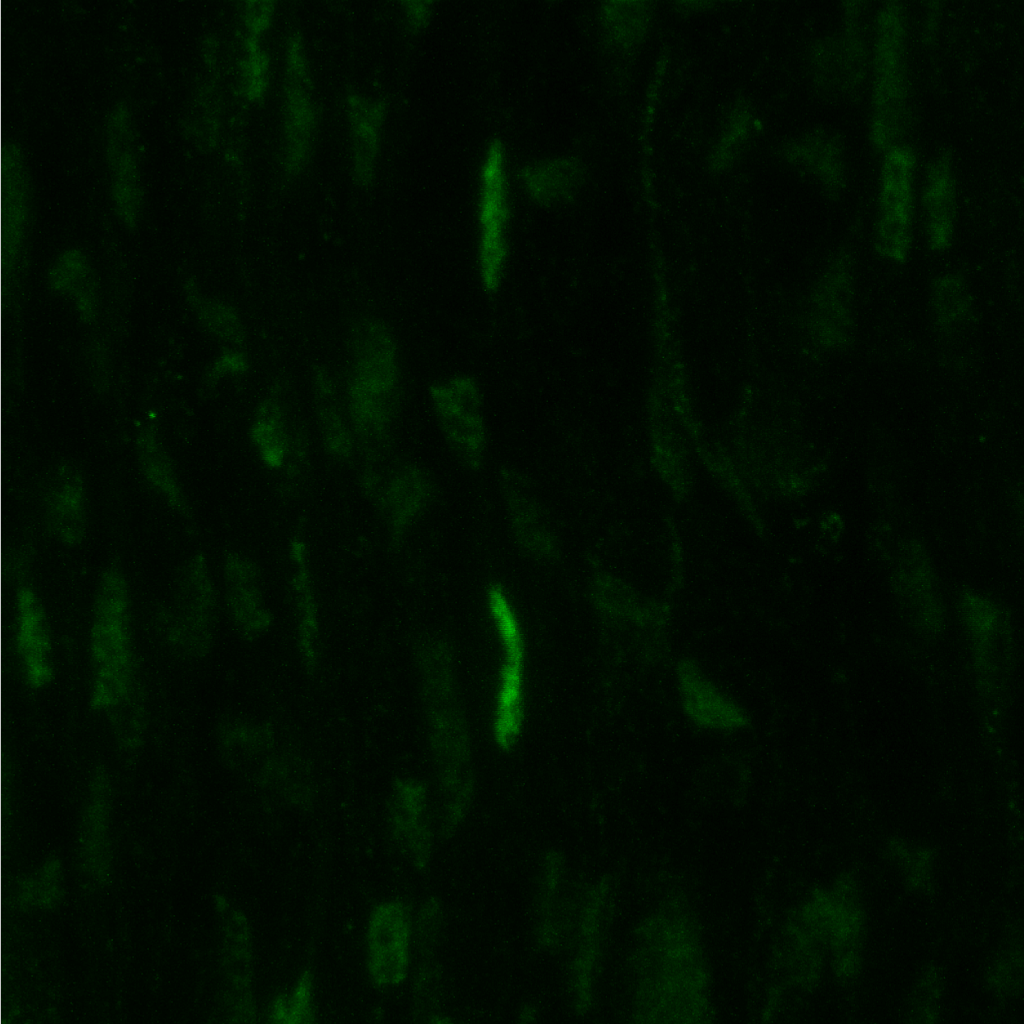

Supplement: Figure 9—source data 1. — This zip archive contains the IHC for one WT and one iDKO used for quantitative analysis shown in Figure 9E. Leica SP8 confocal lif images were processed using Imaris software and saved as tiffs. [file elife-50138-fig9-data1.zip › Figure 9 source data 1/iDKO #492 Krox20/Series 8 Krox20.tif]

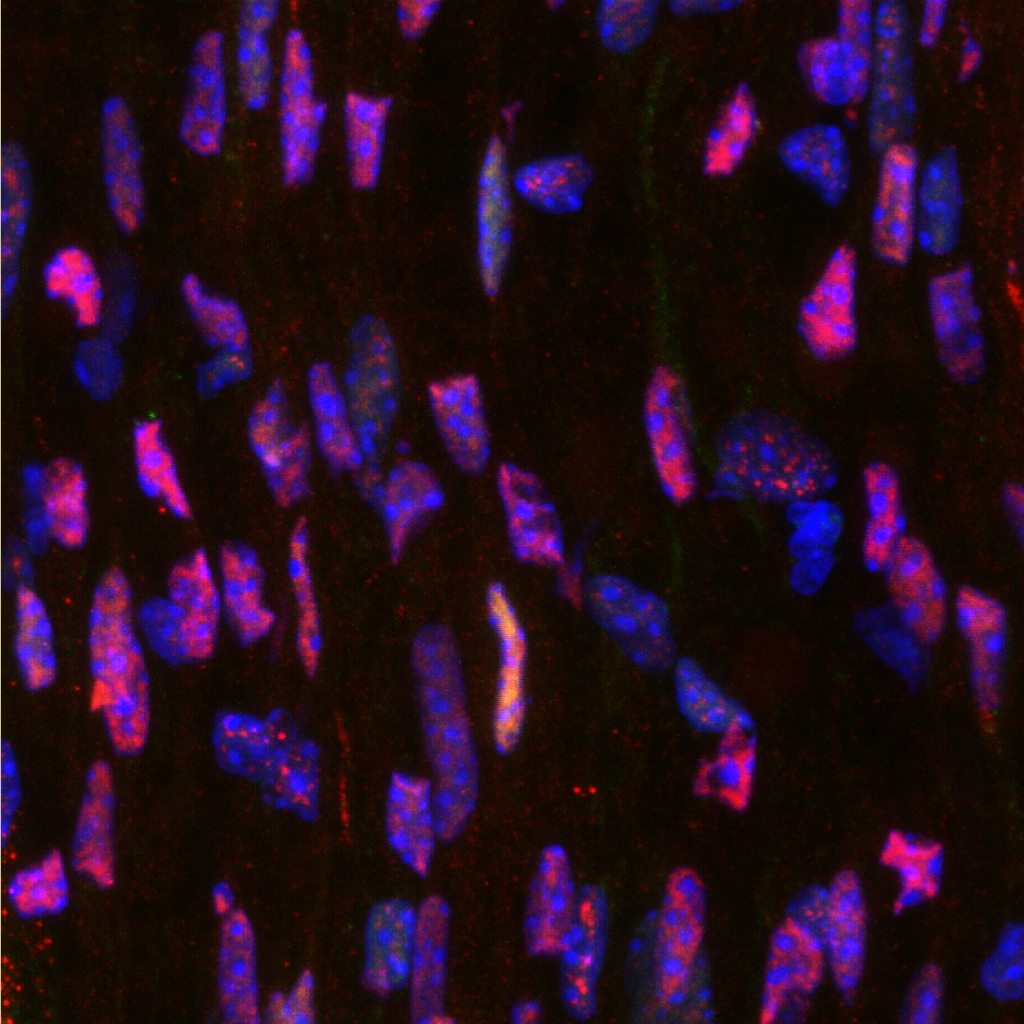

Supplement: Figure 9—source data 1. — This zip archive contains the IHC for one WT and one iDKO used for quantitative analysis shown in Figure 9E. Leica SP8 confocal lif images were processed using Imaris software and saved as tiffs. [file elife-50138-fig9-data1.zip › Figure 9 source data 1/iDKO #492 Krox20/Series 8 merge.tif]

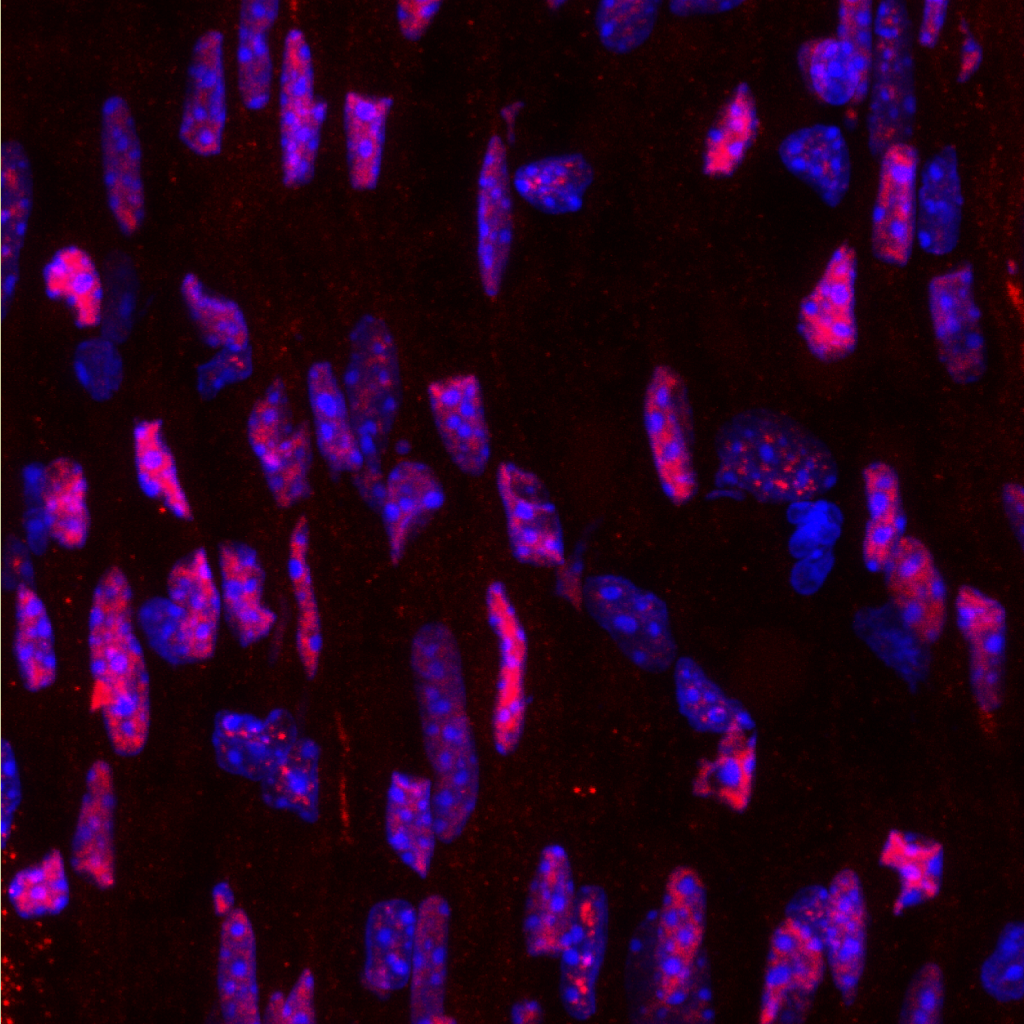

Supplement: Figure 9—source data 1. — This zip archive contains the IHC for one WT and one iDKO used for quantitative analysis shown in Figure 9E. Leica SP8 confocal lif images were processed using Imaris software and saved as tiffs. [file elife-50138-fig9-data1.zip › Figure 9 source data 1/iDKO #492 Krox20/Series 8 Sox10 + DAPI.tif]

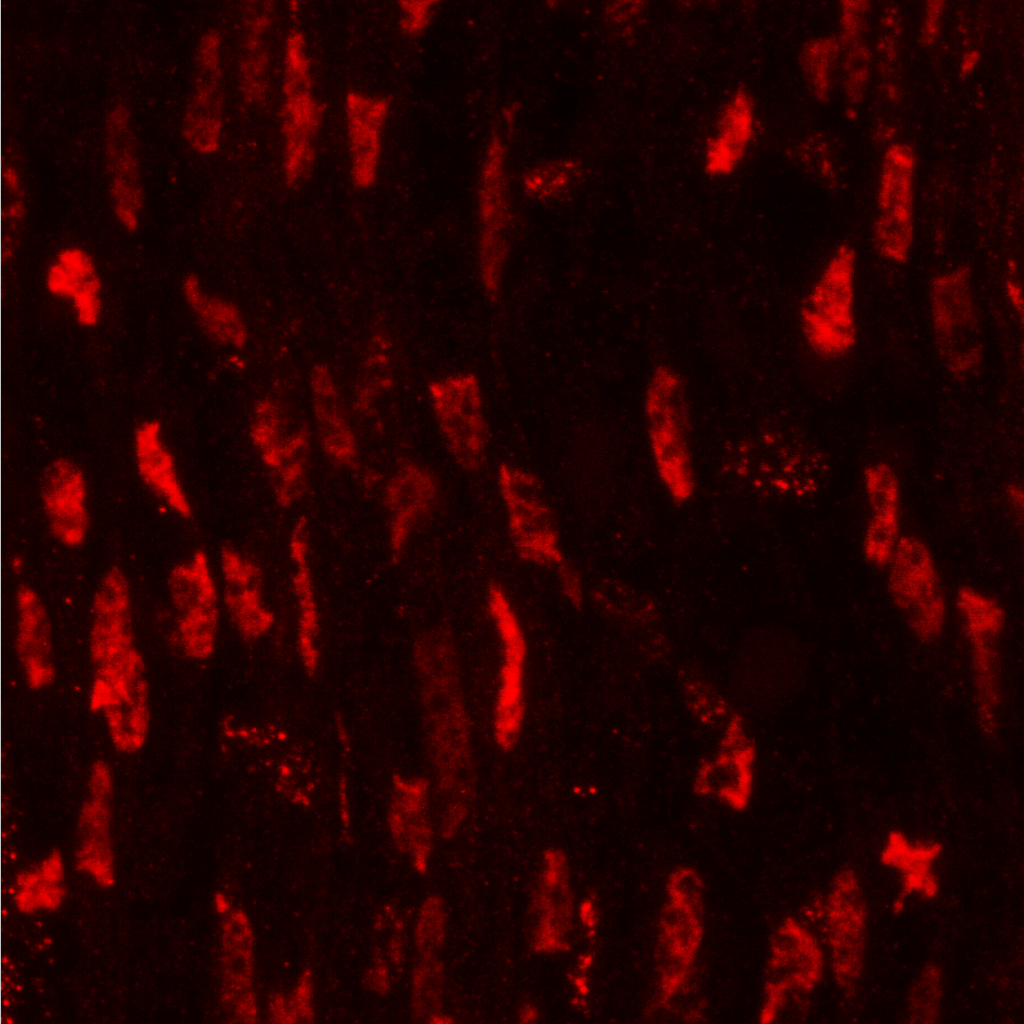

Supplement: Figure 9—source data 1. — This zip archive contains the IHC for one WT and one iDKO used for quantitative analysis shown in Figure 9E. Leica SP8 confocal lif images were processed using Imaris software and saved as tiffs. [file elife-50138-fig9-data1.zip › Figure 9 source data 1/iDKO #492 Krox20/Series 8 Sox10.tif]

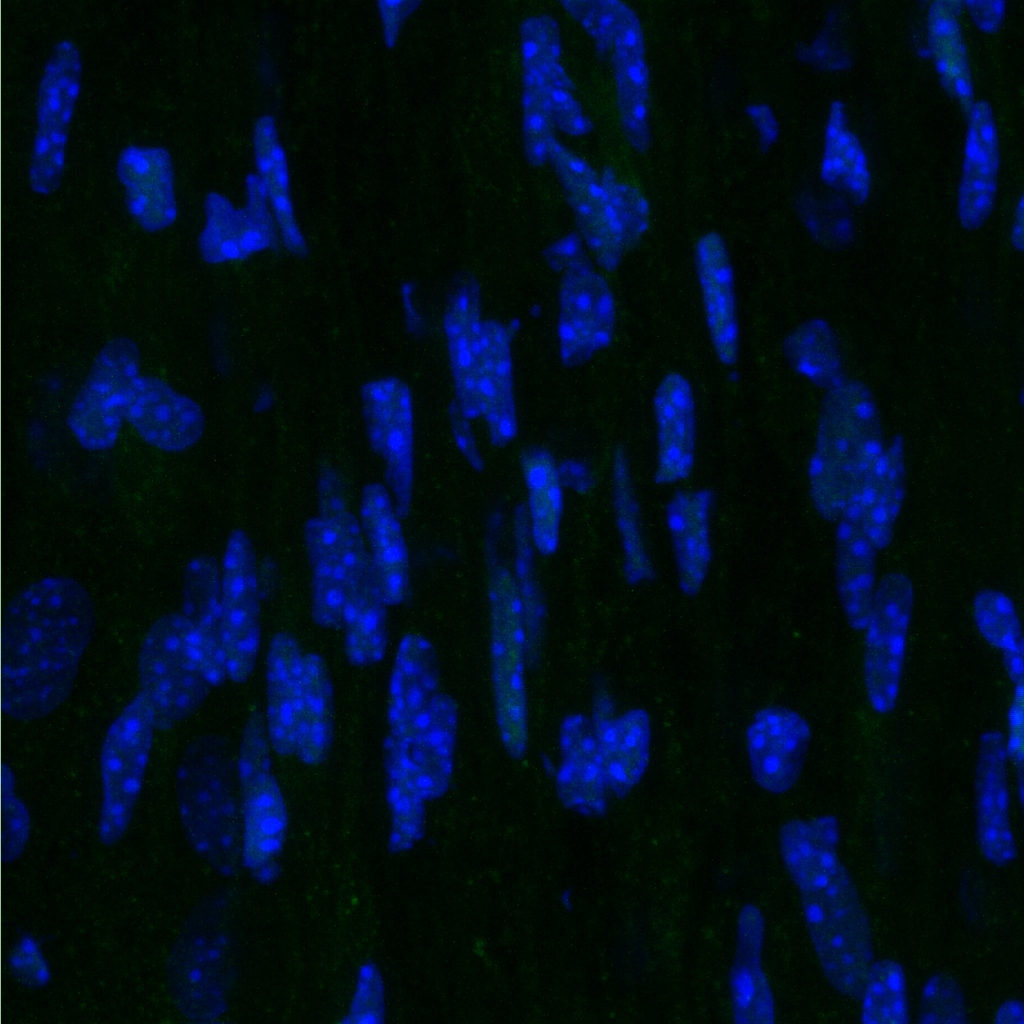

Supplement: Figure 9—source data 1. — This zip archive contains the IHC for one WT and one iDKO used for quantitative analysis shown in Figure 9E. Leica SP8 confocal lif images were processed using Imaris software and saved as tiffs. [file elife-50138-fig9-data1.zip › Figure 9 source data 1/iDKO #492 Krox20/Series 9 Krox20 + DAPI.tif]
