# Supplementary figures and images for: Axon-dependent expression of YAP/TAZ mediates Schwann cell remyelination but not proliferation after nerve injury (part 4 of 4)
Source: eLife. 2020 May 21;9:e50138. doi: 10.7554/eLife.50138 (PMC7259960; doi:10.7554/eLife.50138)

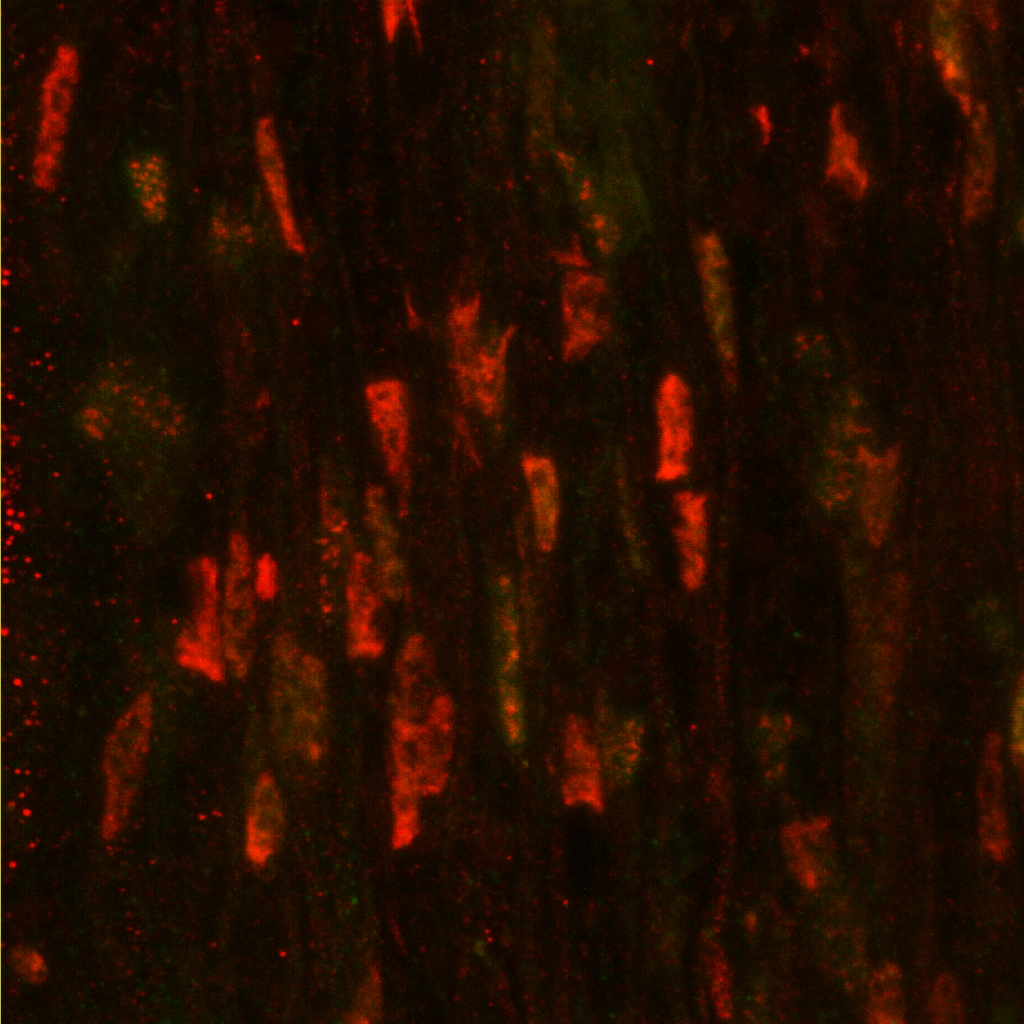

Supplement: Figure 9—source data 1. — This zip archive contains the IHC for one WT and one iDKO used for quantitative analysis shown in Figure 9E. Leica SP8 confocal lif images were processed using Imaris software and saved as tiffs. [file elife-50138-fig9-data1.zip › Figure 9 source data 1/iDKO #492 Krox20/Series 9 Krox20 + Sox10.tif]

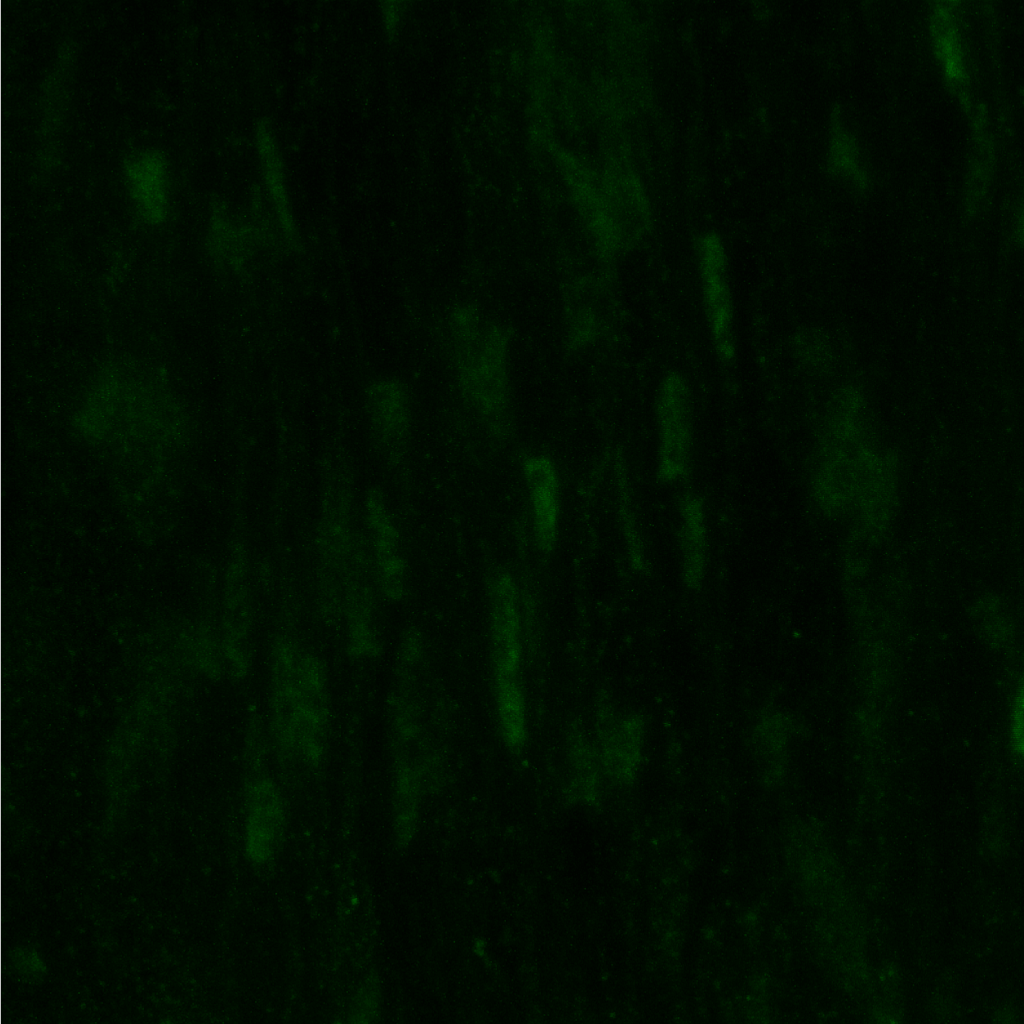

Supplement: Figure 9—source data 1. — This zip archive contains the IHC for one WT and one iDKO used for quantitative analysis shown in Figure 9E. Leica SP8 confocal lif images were processed using Imaris software and saved as tiffs. [file elife-50138-fig9-data1.zip › Figure 9 source data 1/iDKO #492 Krox20/Series 9 Krox20.tif]

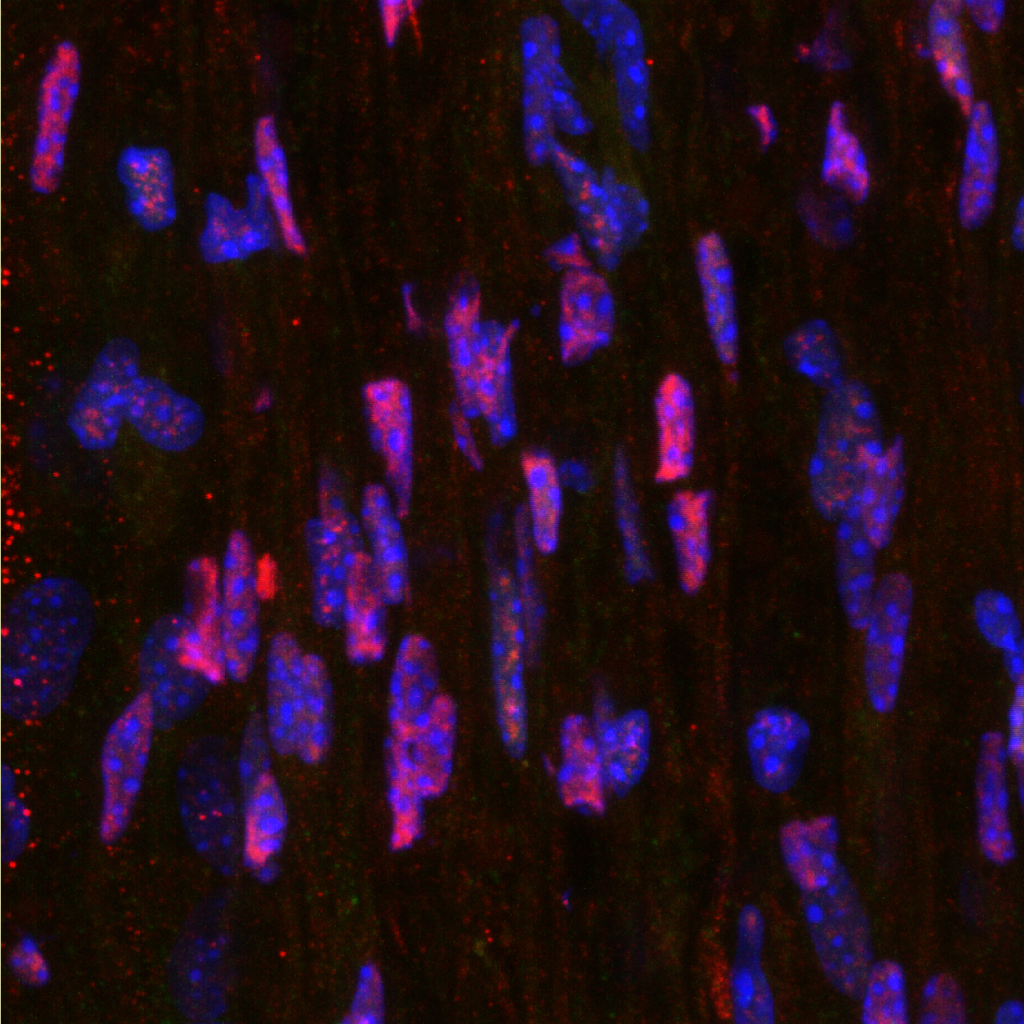

Supplement: Figure 9—source data 1. — This zip archive contains the IHC for one WT and one iDKO used for quantitative analysis shown in Figure 9E. Leica SP8 confocal lif images were processed using Imaris software and saved as tiffs. [file elife-50138-fig9-data1.zip › Figure 9 source data 1/iDKO #492 Krox20/Series 9 merge.tif]

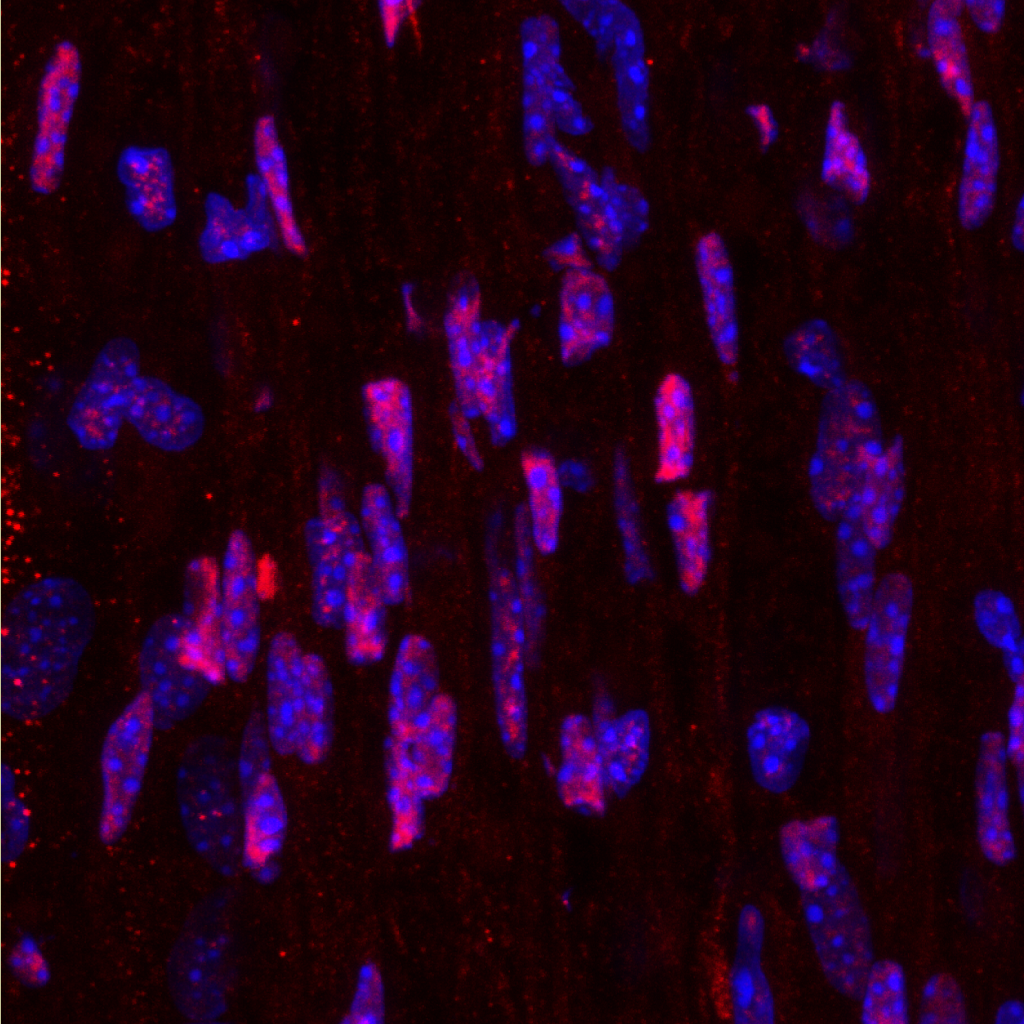

Supplement: Figure 9—source data 1. — This zip archive contains the IHC for one WT and one iDKO used for quantitative analysis shown in Figure 9E. Leica SP8 confocal lif images were processed using Imaris software and saved as tiffs. [file elife-50138-fig9-data1.zip › Figure 9 source data 1/iDKO #492 Krox20/Series 9 Sox10 + DAPI.tif]

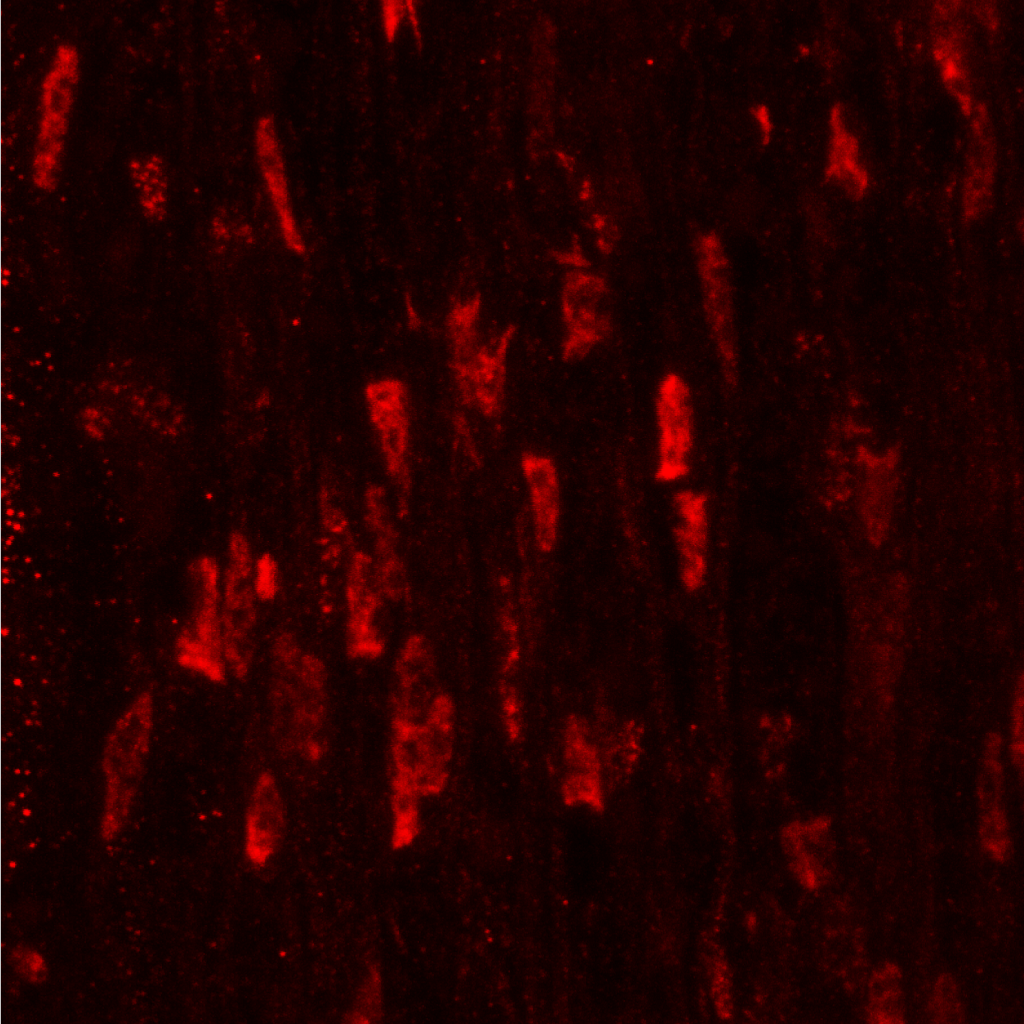

Supplement: Figure 9—source data 1. — This zip archive contains the IHC for one WT and one iDKO used for quantitative analysis shown in Figure 9E. Leica SP8 confocal lif images were processed using Imaris software and saved as tiffs. [file elife-50138-fig9-data1.zip › Figure 9 source data 1/iDKO #492 Krox20/Series 9 Sox10.tif]

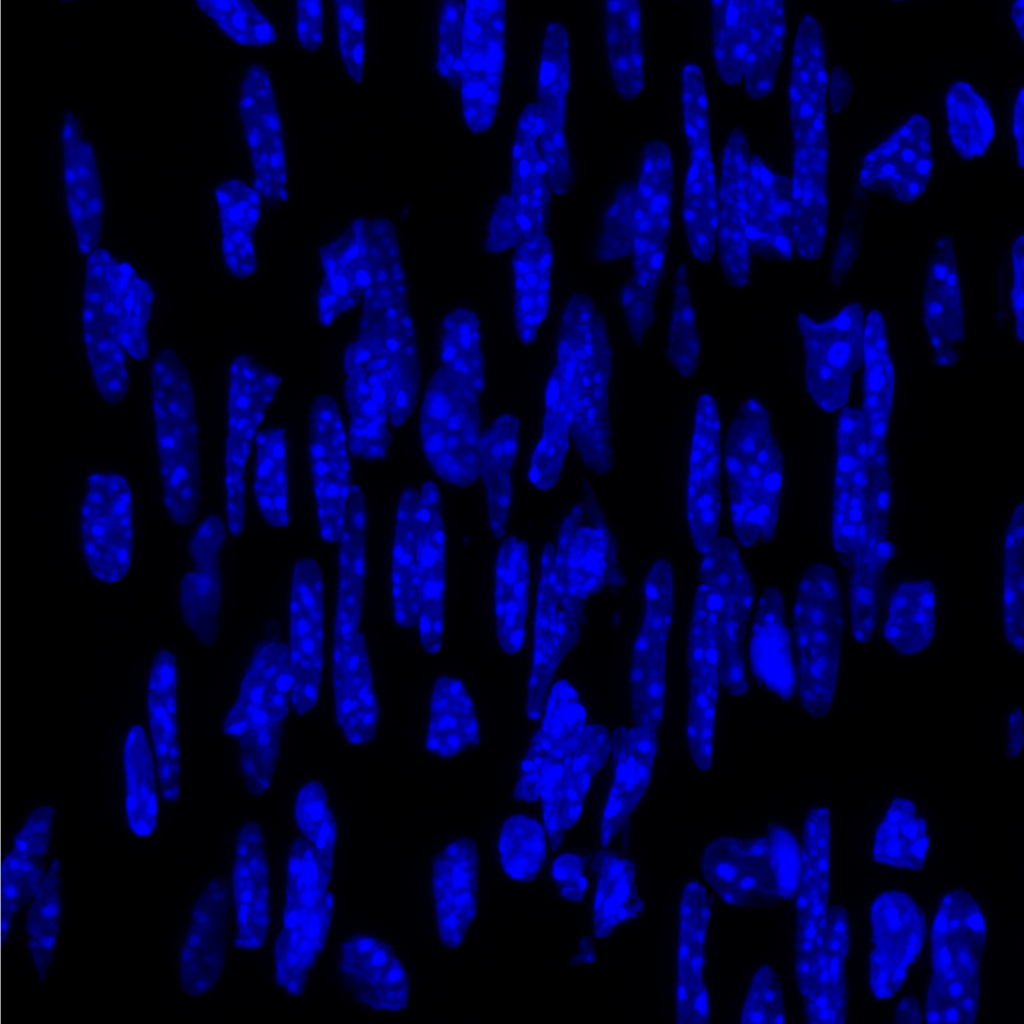

Supplement: Figure 9—source data 1. — This zip archive contains the IHC for one WT and one iDKO used for quantitative analysis shown in Figure 9E. Leica SP8 confocal lif images were processed using Imaris software and saved as tiffs. [file elife-50138-fig9-data1.zip › Figure 9 source data 1/WT #559 Krox20/Series 1 DAPI.tif]

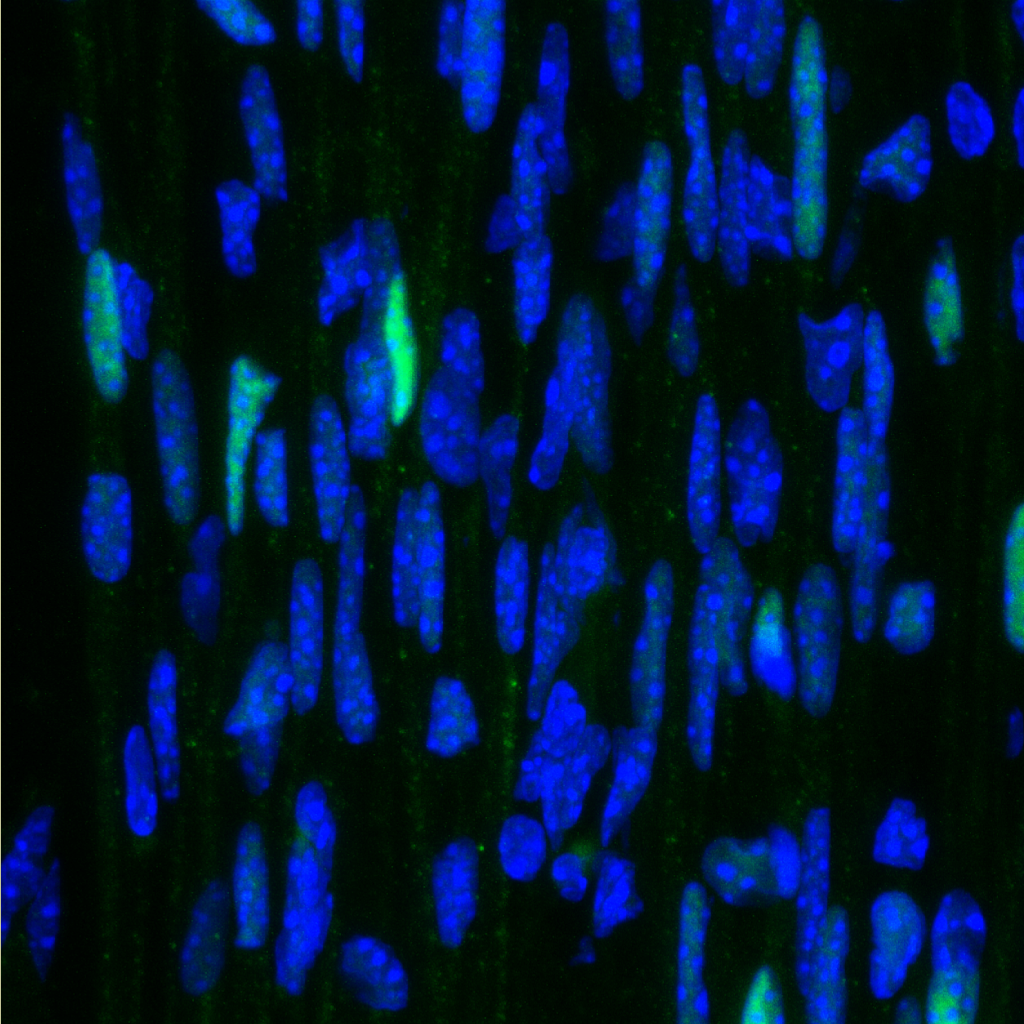

Supplement: Figure 9—source data 1. — This zip archive contains the IHC for one WT and one iDKO used for quantitative analysis shown in Figure 9E. Leica SP8 confocal lif images were processed using Imaris software and saved as tiffs. [file elife-50138-fig9-data1.zip › Figure 9 source data 1/WT #559 Krox20/Series 1 Krox20 + DAPI.tif]

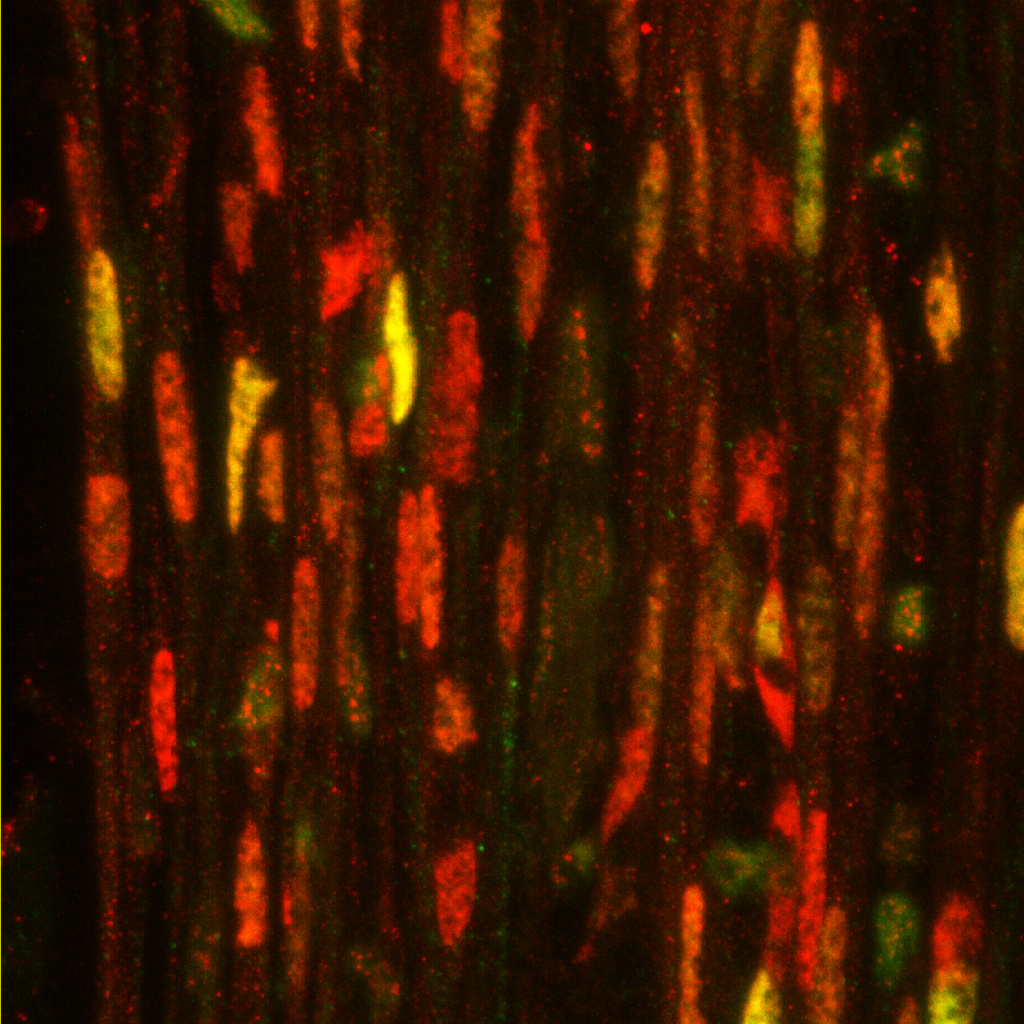

Supplement: Figure 9—source data 1. — This zip archive contains the IHC for one WT and one iDKO used for quantitative analysis shown in Figure 9E. Leica SP8 confocal lif images were processed using Imaris software and saved as tiffs. [file elife-50138-fig9-data1.zip › Figure 9 source data 1/WT #559 Krox20/Series 1 Krox20 + Sox10.tif]

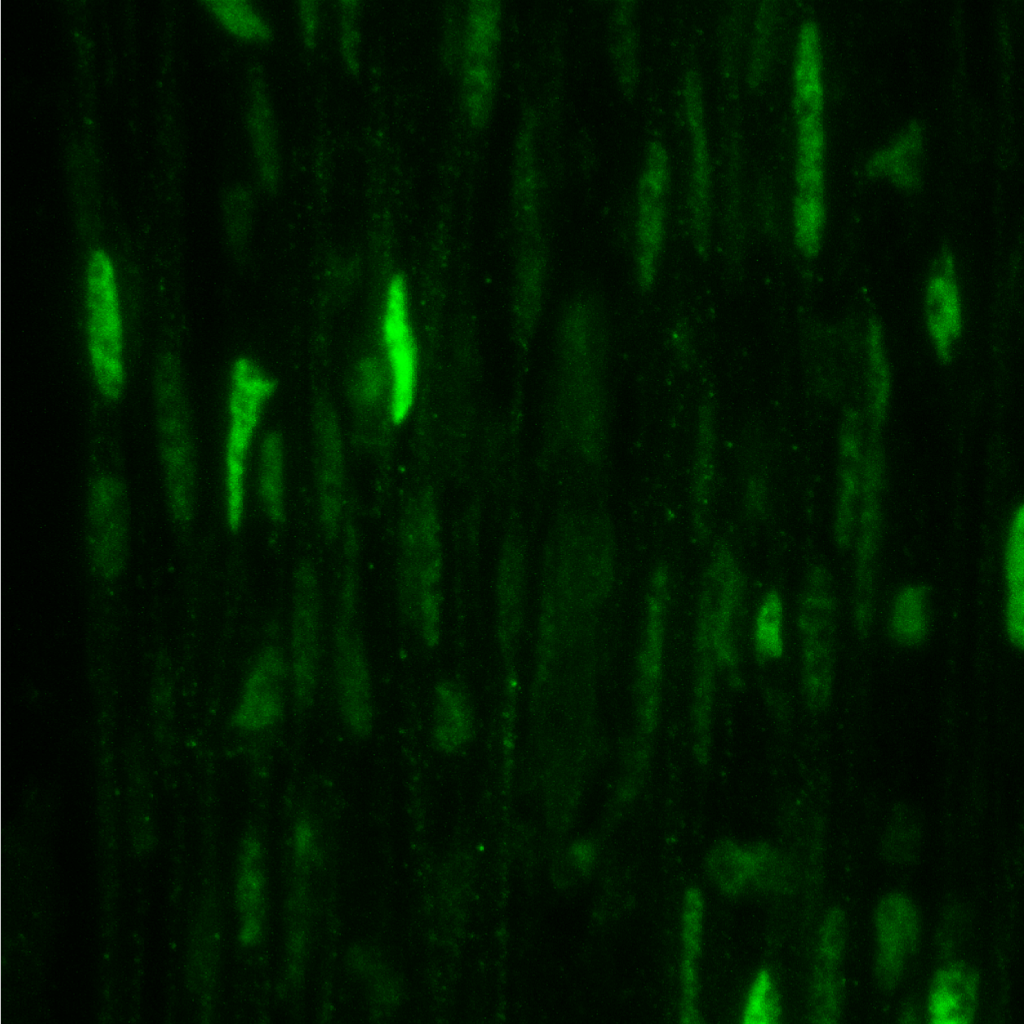

Supplement: Figure 9—source data 1. — This zip archive contains the IHC for one WT and one iDKO used for quantitative analysis shown in Figure 9E. Leica SP8 confocal lif images were processed using Imaris software and saved as tiffs. [file elife-50138-fig9-data1.zip › Figure 9 source data 1/WT #559 Krox20/Series 1 Krox20.tif]

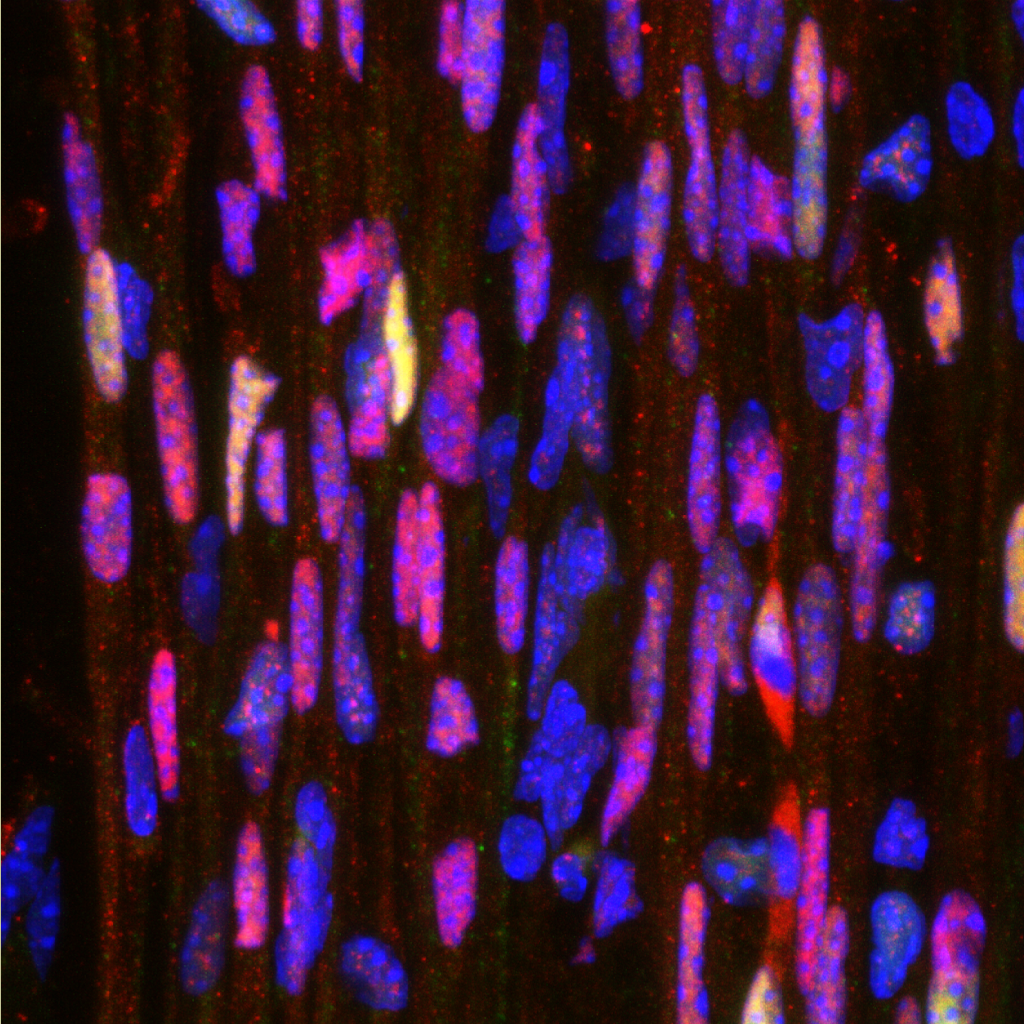

Supplement: Figure 9—source data 1. — This zip archive contains the IHC for one WT and one iDKO used for quantitative analysis shown in Figure 9E. Leica SP8 confocal lif images were processed using Imaris software and saved as tiffs. [file elife-50138-fig9-data1.zip › Figure 9 source data 1/WT #559 Krox20/Series 1 merge.tif]

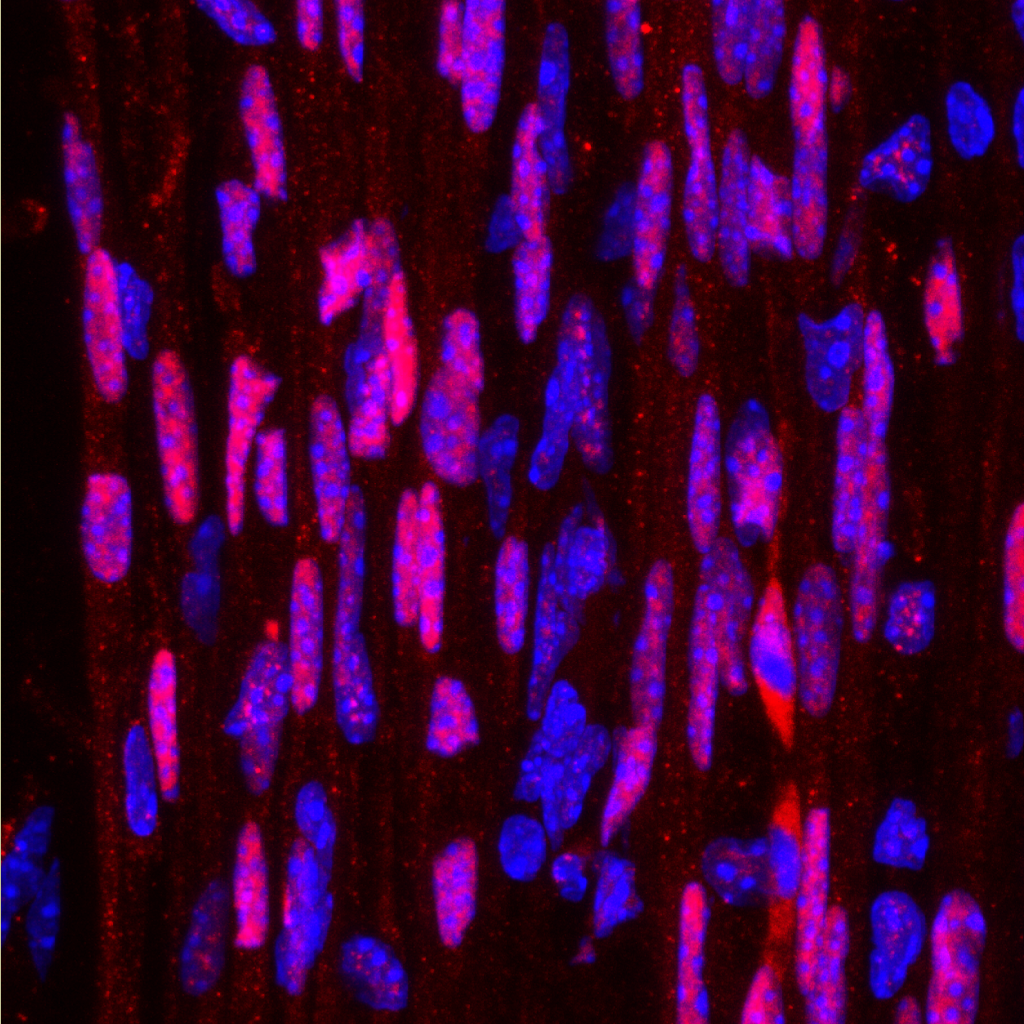

Supplement: Figure 9—source data 1. — This zip archive contains the IHC for one WT and one iDKO used for quantitative analysis shown in Figure 9E. Leica SP8 confocal lif images were processed using Imaris software and saved as tiffs. [file elife-50138-fig9-data1.zip › Figure 9 source data 1/WT #559 Krox20/Series 1 Sox10 + DAPI.tif]

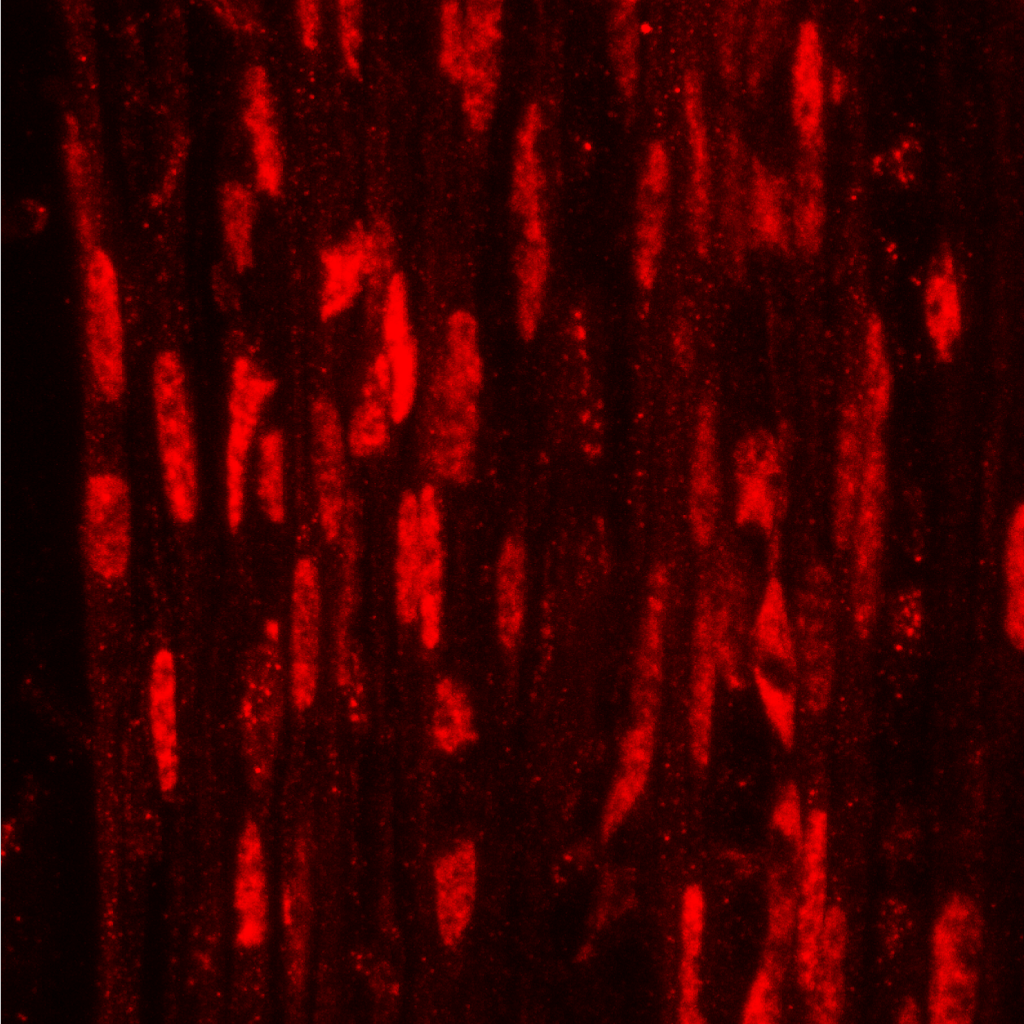

Supplement: Figure 9—source data 1. — This zip archive contains the IHC for one WT and one iDKO used for quantitative analysis shown in Figure 9E. Leica SP8 confocal lif images were processed using Imaris software and saved as tiffs. [file elife-50138-fig9-data1.zip › Figure 9 source data 1/WT #559 Krox20/Series 1 Sox10.tif]

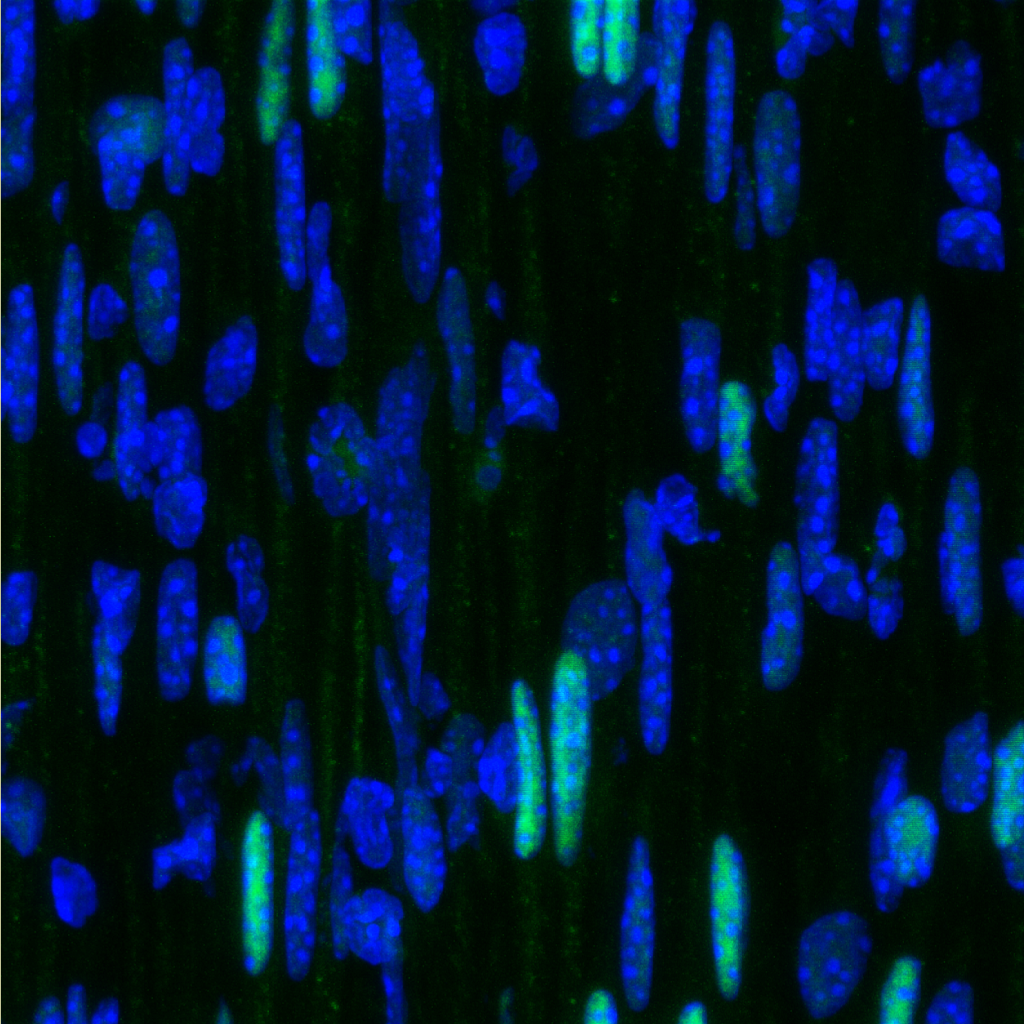

Supplement: Figure 9—source data 1. — This zip archive contains the IHC for one WT and one iDKO used for quantitative analysis shown in Figure 9E. Leica SP8 confocal lif images were processed using Imaris software and saved as tiffs. [file elife-50138-fig9-data1.zip › Figure 9 source data 1/WT #559 Krox20/Series 3 Krox20 + DAPI.tif]

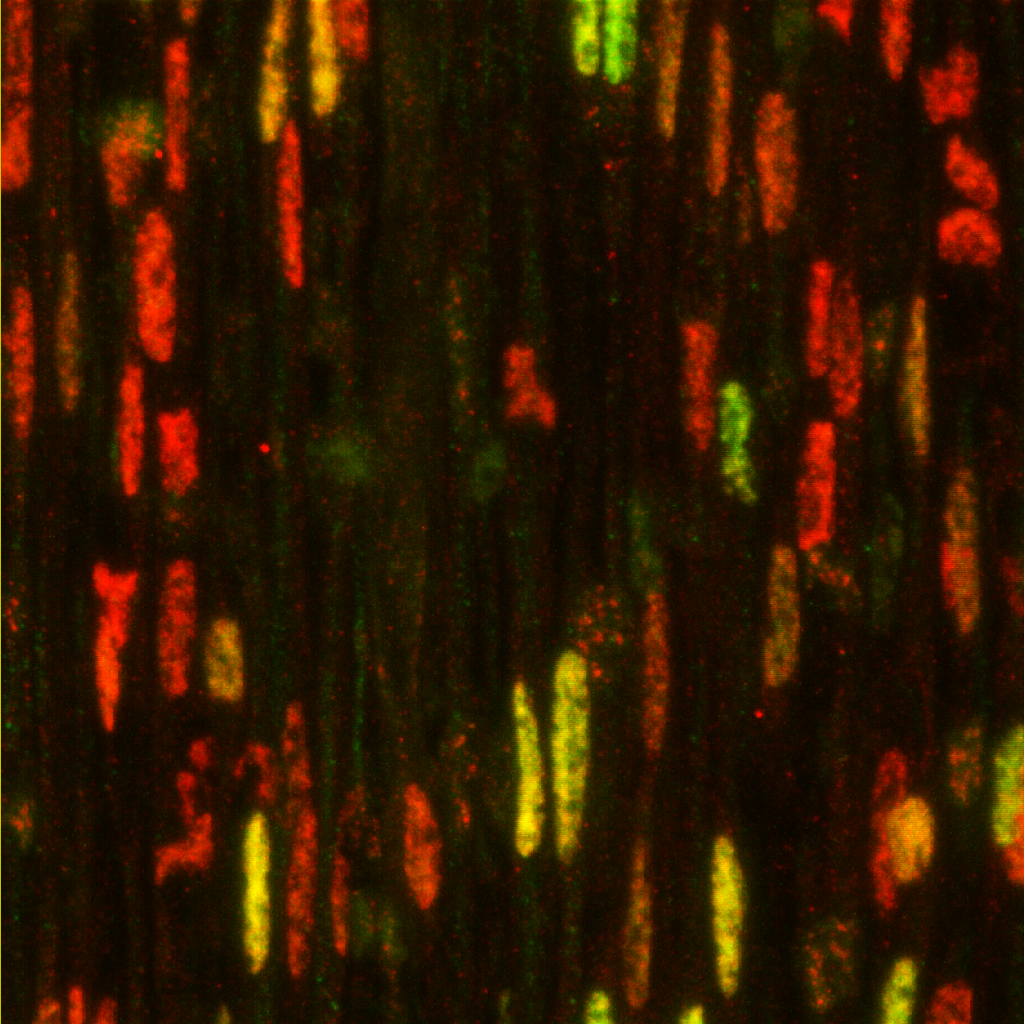

Supplement: Figure 9—source data 1. — This zip archive contains the IHC for one WT and one iDKO used for quantitative analysis shown in Figure 9E. Leica SP8 confocal lif images were processed using Imaris software and saved as tiffs. [file elife-50138-fig9-data1.zip › Figure 9 source data 1/WT #559 Krox20/Series 3 Krox20 + Sox10.tif]

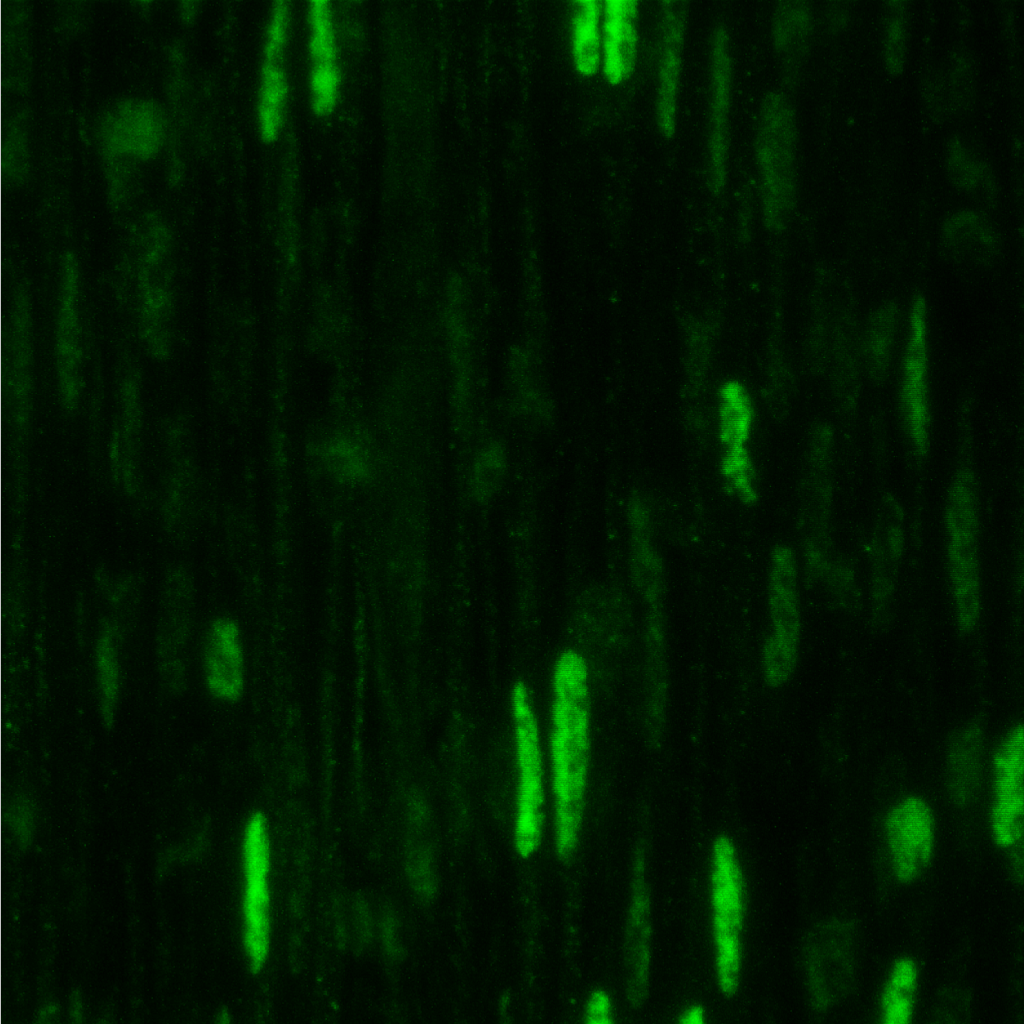

Supplement: Figure 9—source data 1. — This zip archive contains the IHC for one WT and one iDKO used for quantitative analysis shown in Figure 9E. Leica SP8 confocal lif images were processed using Imaris software and saved as tiffs. [file elife-50138-fig9-data1.zip › Figure 9 source data 1/WT #559 Krox20/Series 3 Krox20.tif]

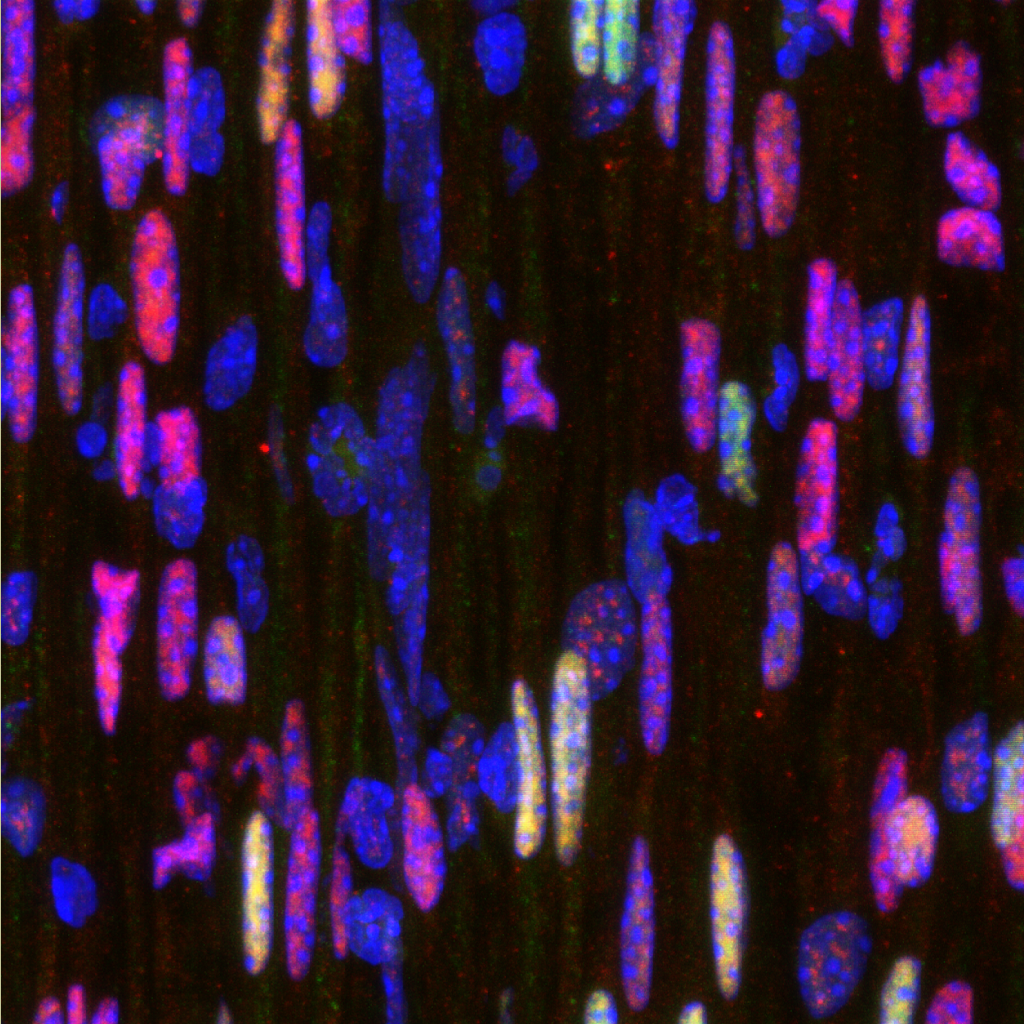

Supplement: Figure 9—source data 1. — This zip archive contains the IHC for one WT and one iDKO used for quantitative analysis shown in Figure 9E. Leica SP8 confocal lif images were processed using Imaris software and saved as tiffs. [file elife-50138-fig9-data1.zip › Figure 9 source data 1/WT #559 Krox20/Series 3 merge.tif]

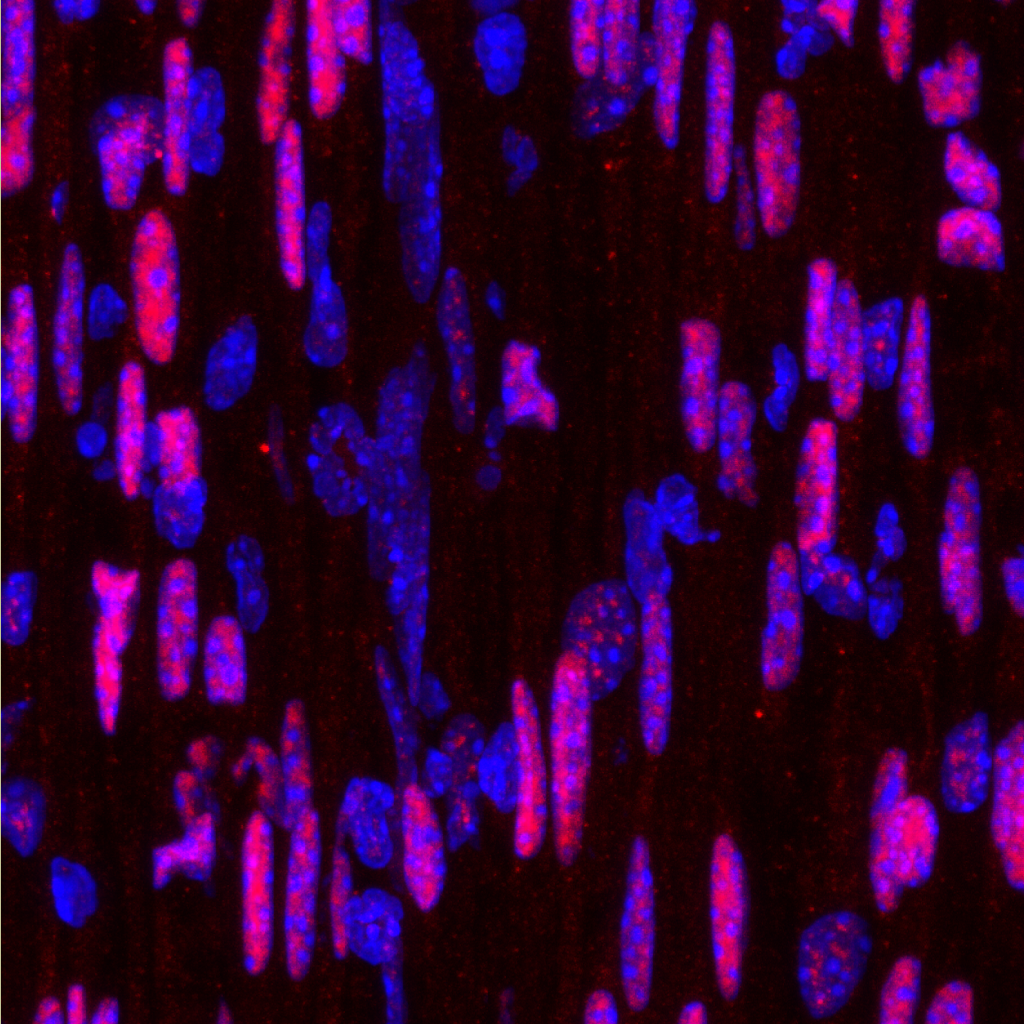

Supplement: Figure 9—source data 1. — This zip archive contains the IHC for one WT and one iDKO used for quantitative analysis shown in Figure 9E. Leica SP8 confocal lif images were processed using Imaris software and saved as tiffs. [file elife-50138-fig9-data1.zip › Figure 9 source data 1/WT #559 Krox20/Series 3 Sox10 + DAPI.tif]

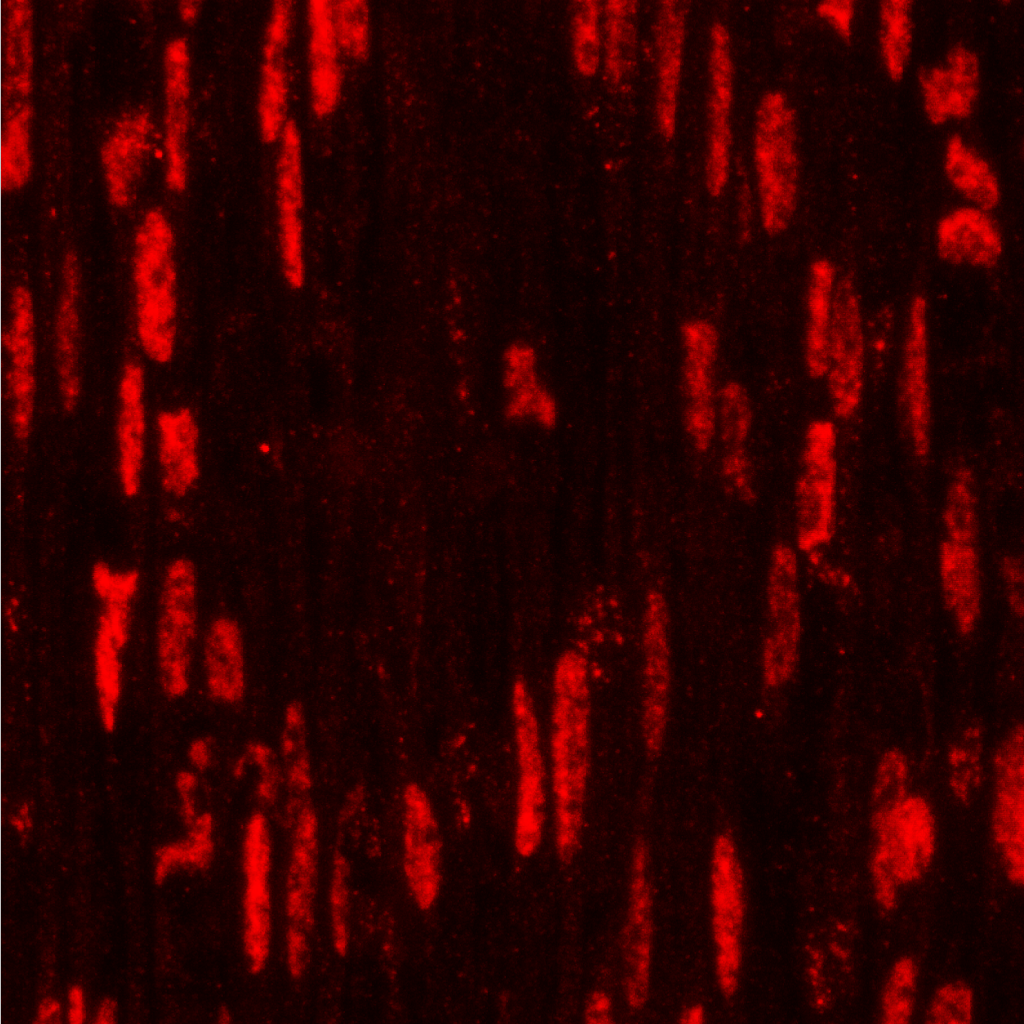

Supplement: Figure 9—source data 1. — This zip archive contains the IHC for one WT and one iDKO used for quantitative analysis shown in Figure 9E. Leica SP8 confocal lif images were processed using Imaris software and saved as tiffs. [file elife-50138-fig9-data1.zip › Figure 9 source data 1/WT #559 Krox20/Series 3 Sox10.tif]

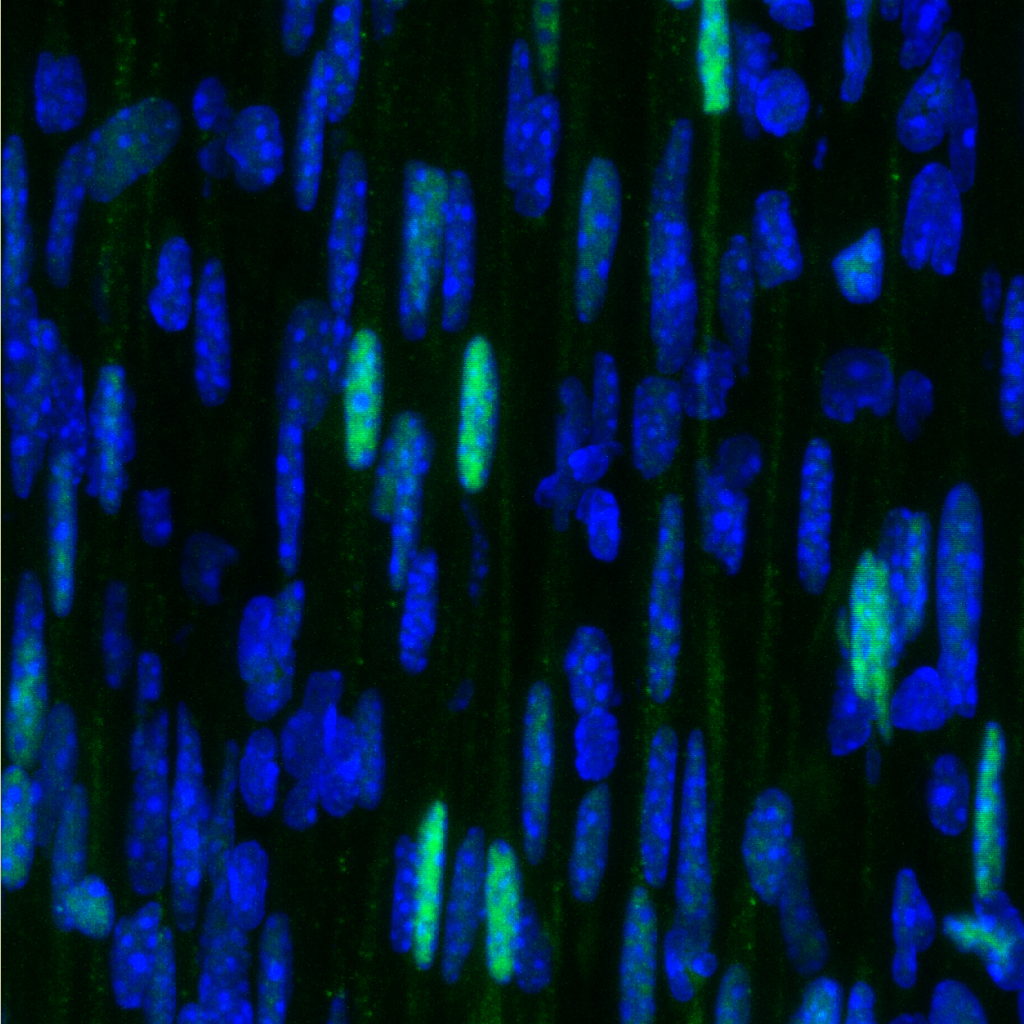

Supplement: Figure 9—source data 1. — This zip archive contains the IHC for one WT and one iDKO used for quantitative analysis shown in Figure 9E. Leica SP8 confocal lif images were processed using Imaris software and saved as tiffs. [file elife-50138-fig9-data1.zip › Figure 9 source data 1/WT #559 Krox20/Series 4 Krox20 + DAPI.tif]

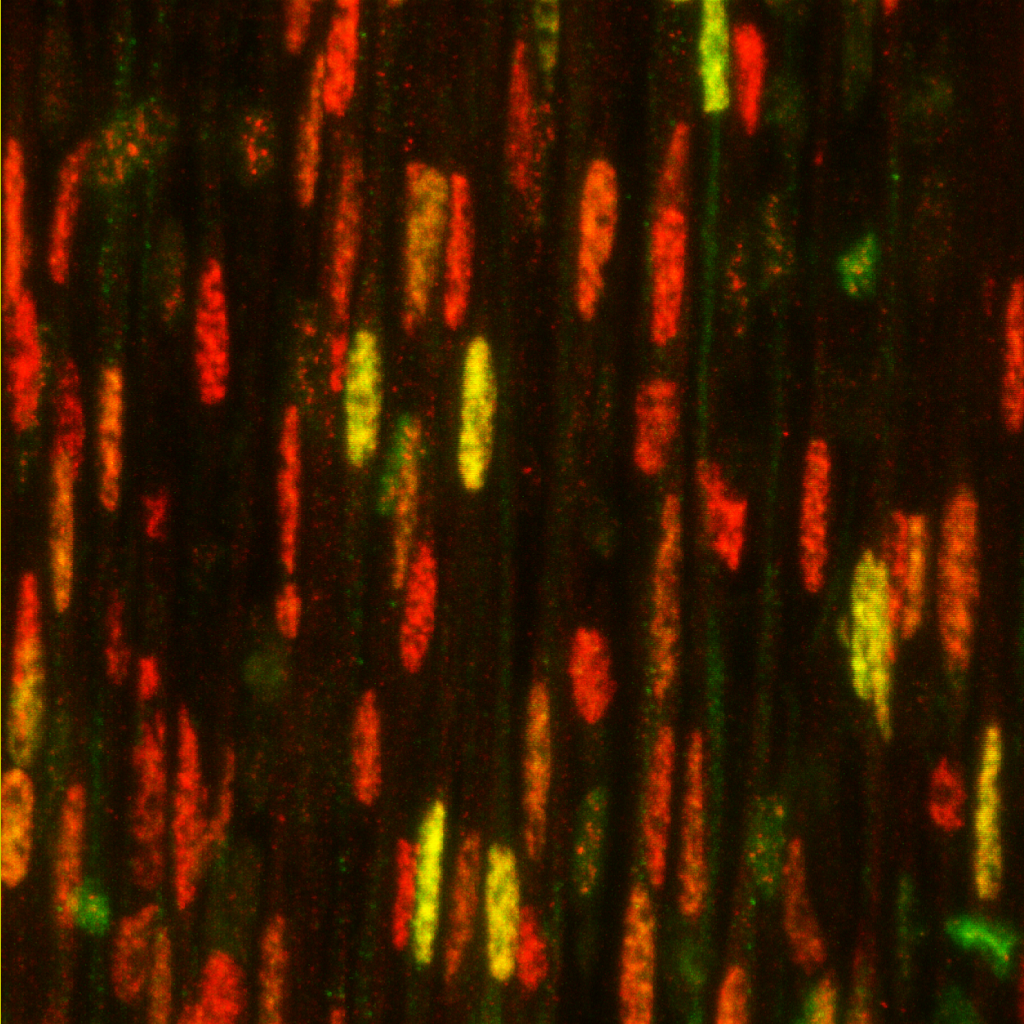

Supplement: Figure 9—source data 1. — This zip archive contains the IHC for one WT and one iDKO used for quantitative analysis shown in Figure 9E. Leica SP8 confocal lif images were processed using Imaris software and saved as tiffs. [file elife-50138-fig9-data1.zip › Figure 9 source data 1/WT #559 Krox20/Series 4 Krox20 + Sox10.tif]

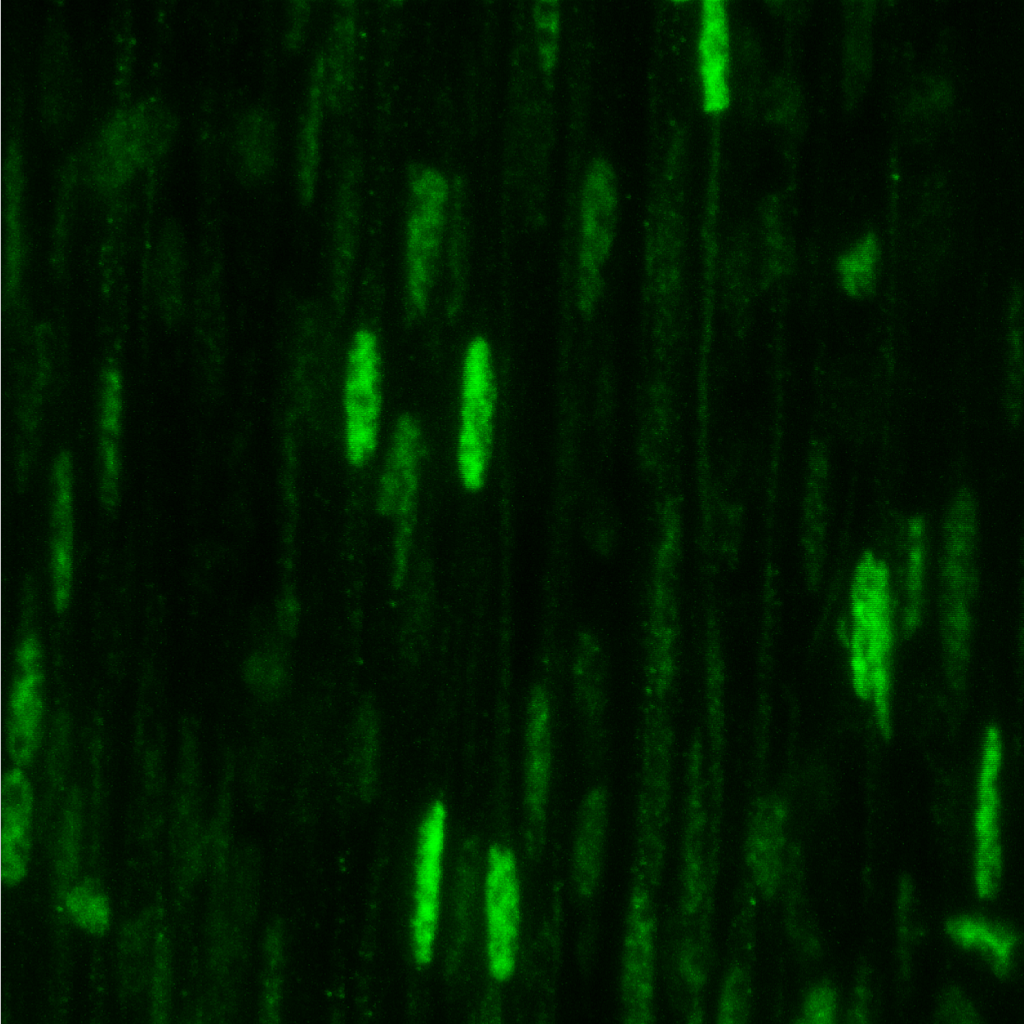

Supplement: Figure 9—source data 1. — This zip archive contains the IHC for one WT and one iDKO used for quantitative analysis shown in Figure 9E. Leica SP8 confocal lif images were processed using Imaris software and saved as tiffs. [file elife-50138-fig9-data1.zip › Figure 9 source data 1/WT #559 Krox20/Series 4 Krox20.tif]

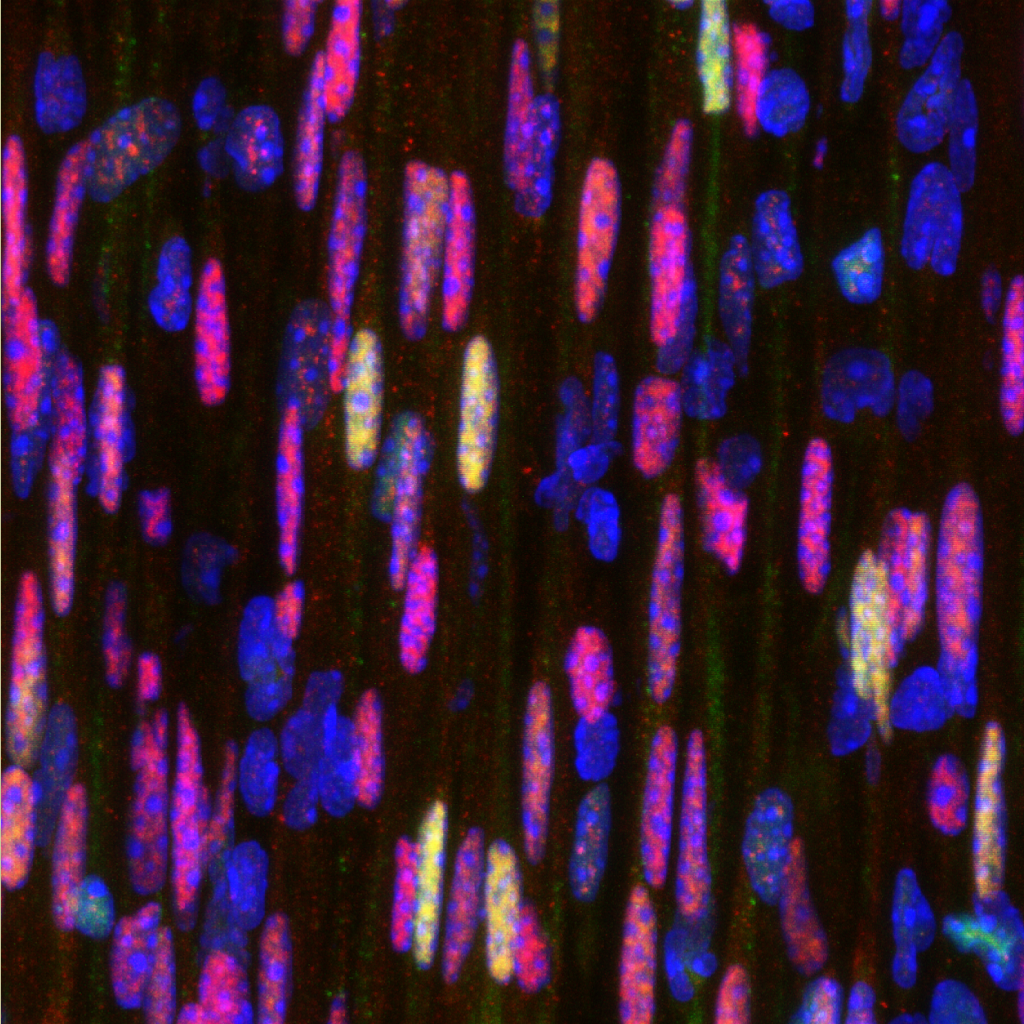

Supplement: Figure 9—source data 1. — This zip archive contains the IHC for one WT and one iDKO used for quantitative analysis shown in Figure 9E. Leica SP8 confocal lif images were processed using Imaris software and saved as tiffs. [file elife-50138-fig9-data1.zip › Figure 9 source data 1/WT #559 Krox20/Series 4 merge.tif]

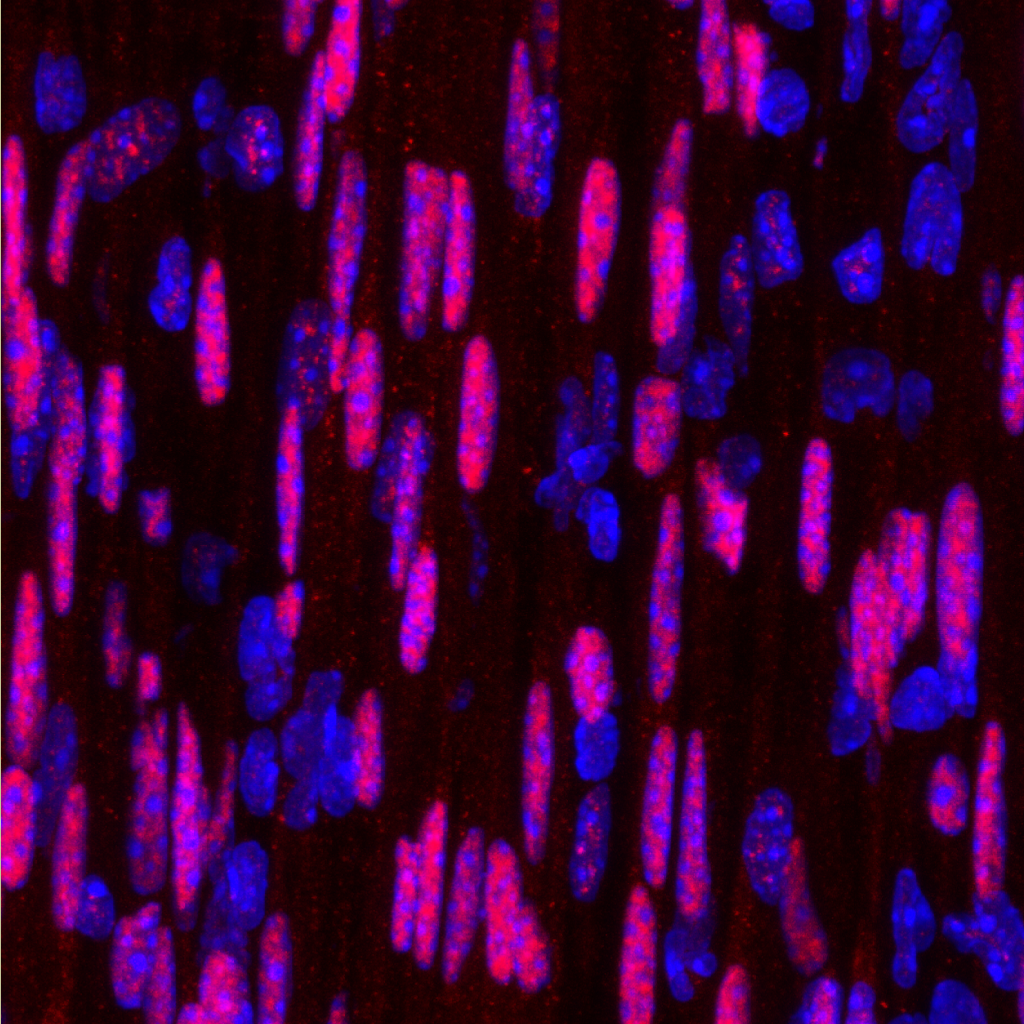

Supplement: Figure 9—source data 1. — This zip archive contains the IHC for one WT and one iDKO used for quantitative analysis shown in Figure 9E. Leica SP8 confocal lif images were processed using Imaris software and saved as tiffs. [file elife-50138-fig9-data1.zip › Figure 9 source data 1/WT #559 Krox20/Series 4 Sox10 + DAPI.tif]

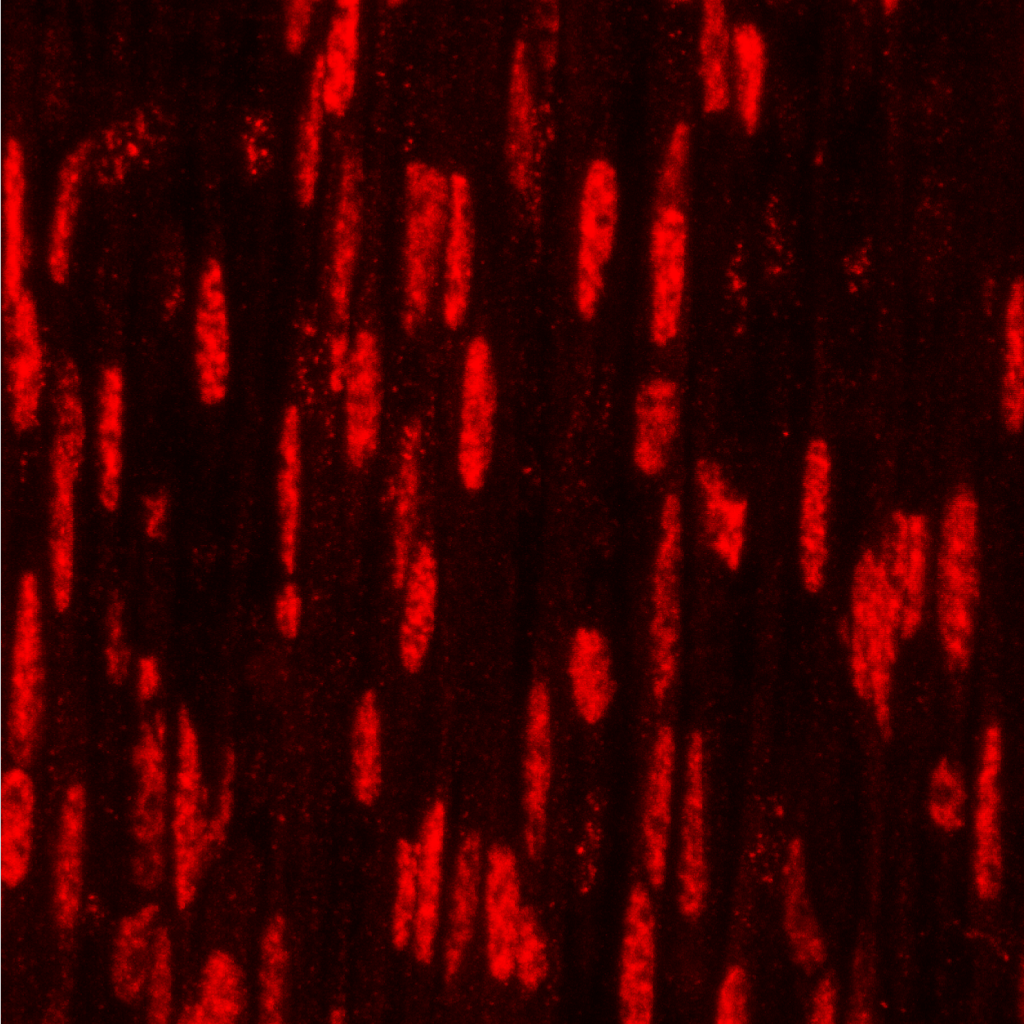

Supplement: Figure 9—source data 1. — This zip archive contains the IHC for one WT and one iDKO used for quantitative analysis shown in Figure 9E. Leica SP8 confocal lif images were processed using Imaris software and saved as tiffs. [file elife-50138-fig9-data1.zip › Figure 9 source data 1/WT #559 Krox20/Series 4 Sox10.tif]

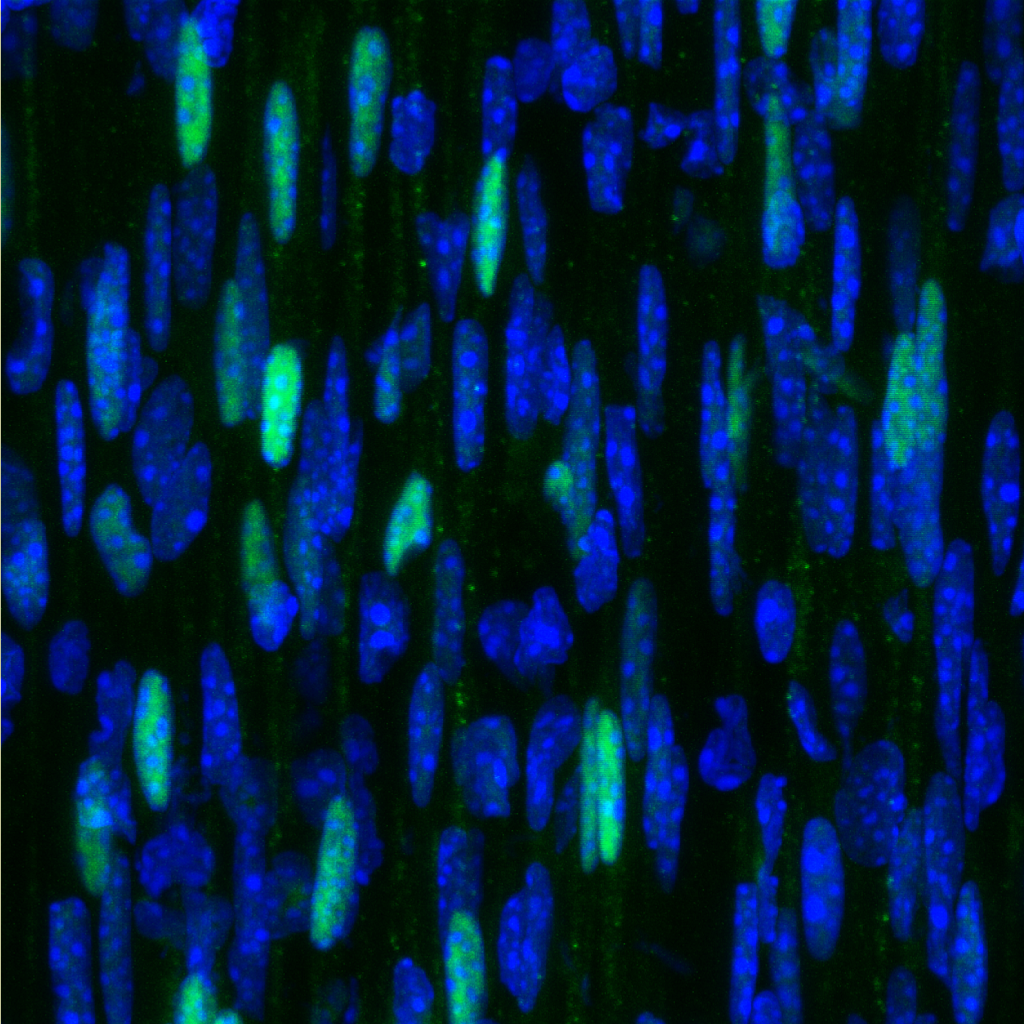

Supplement: Figure 9—source data 1. — This zip archive contains the IHC for one WT and one iDKO used for quantitative analysis shown in Figure 9E. Leica SP8 confocal lif images were processed using Imaris software and saved as tiffs. [file elife-50138-fig9-data1.zip › Figure 9 source data 1/WT #559 Krox20/Series 5 Krox20 + DAPI.tif]

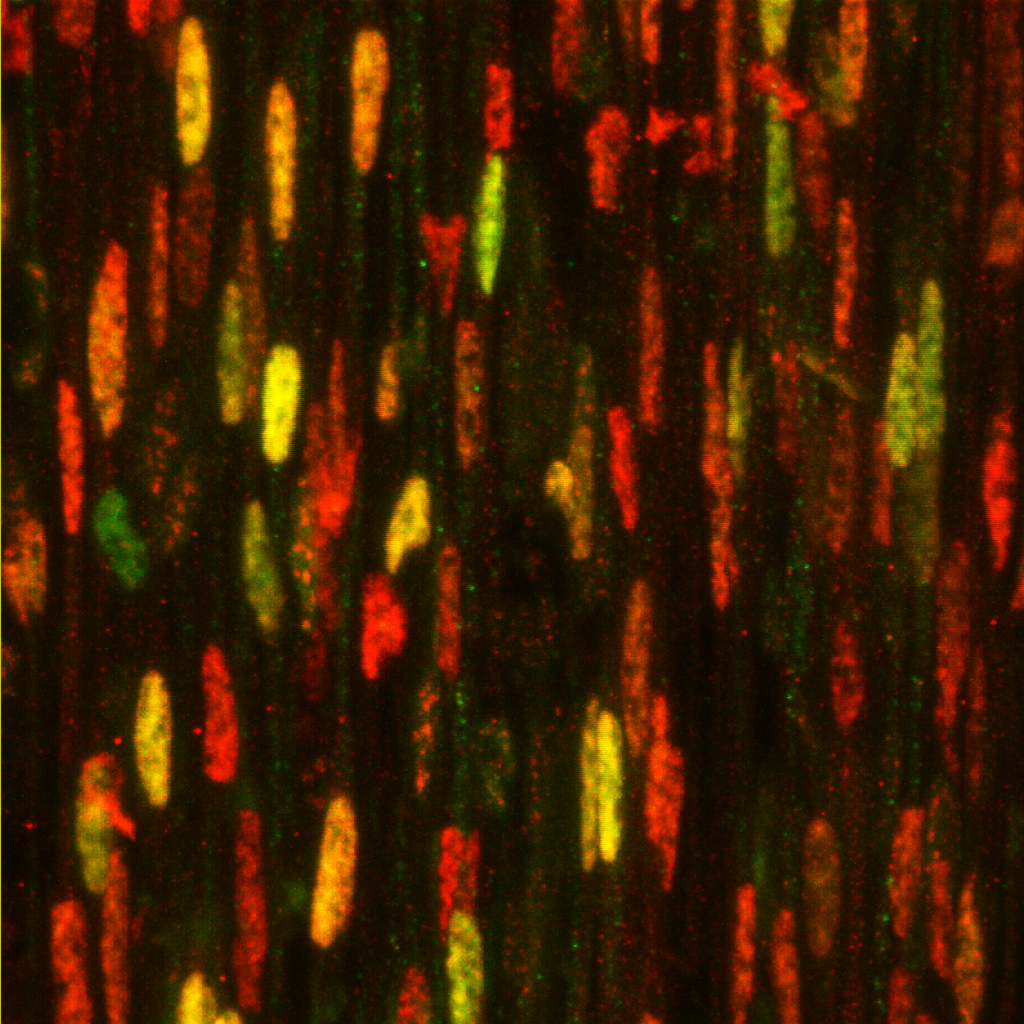

Supplement: Figure 9—source data 1. — This zip archive contains the IHC for one WT and one iDKO used for quantitative analysis shown in Figure 9E. Leica SP8 confocal lif images were processed using Imaris software and saved as tiffs. [file elife-50138-fig9-data1.zip › Figure 9 source data 1/WT #559 Krox20/Series 5 Krox20 + Sox10.tif]

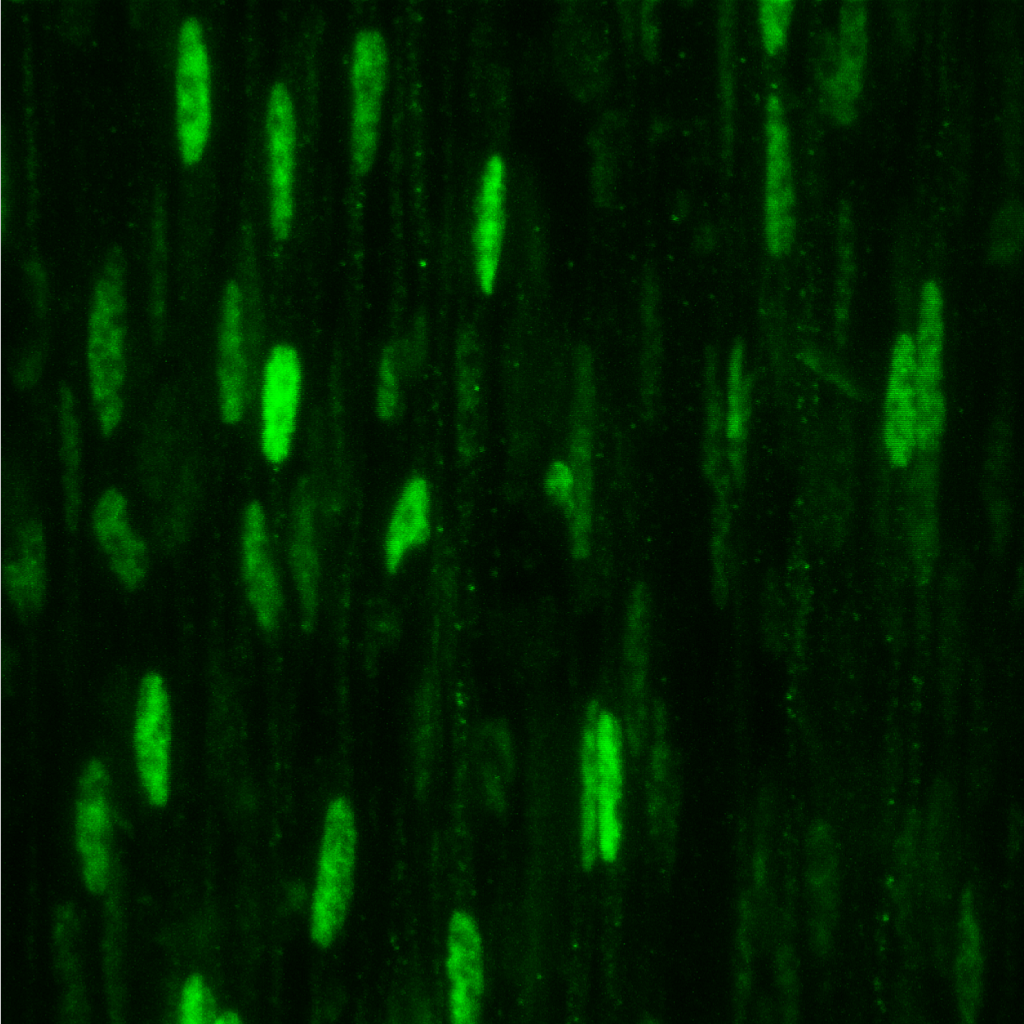

Supplement: Figure 9—source data 1. — This zip archive contains the IHC for one WT and one iDKO used for quantitative analysis shown in Figure 9E. Leica SP8 confocal lif images were processed using Imaris software and saved as tiffs. [file elife-50138-fig9-data1.zip › Figure 9 source data 1/WT #559 Krox20/Series 5 Krox20.tif]

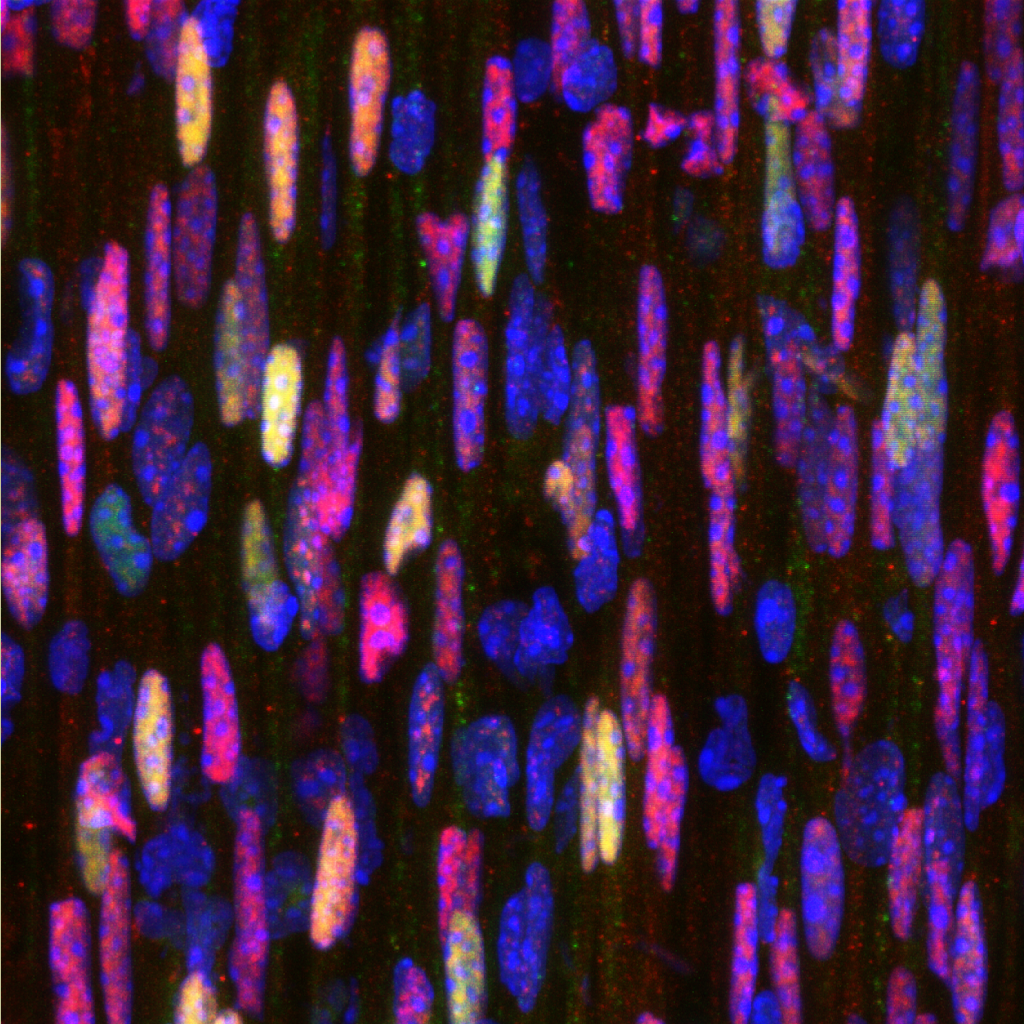

Supplement: Figure 9—source data 1. — This zip archive contains the IHC for one WT and one iDKO used for quantitative analysis shown in Figure 9E. Leica SP8 confocal lif images were processed using Imaris software and saved as tiffs. [file elife-50138-fig9-data1.zip › Figure 9 source data 1/WT #559 Krox20/Series 5 merge.tif]

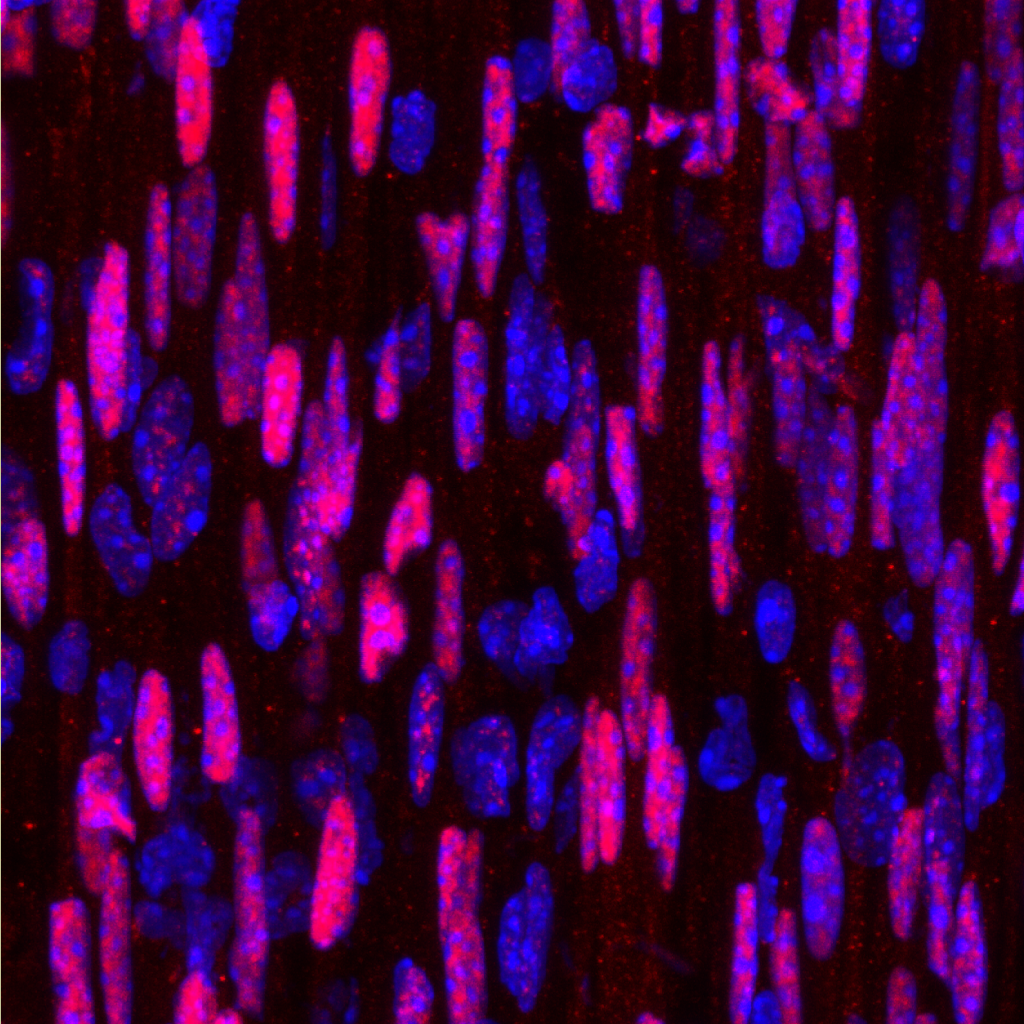

Supplement: Figure 9—source data 1. — This zip archive contains the IHC for one WT and one iDKO used for quantitative analysis shown in Figure 9E. Leica SP8 confocal lif images were processed using Imaris software and saved as tiffs. [file elife-50138-fig9-data1.zip › Figure 9 source data 1/WT #559 Krox20/Series 5 Sox10 + DAPI.tif]

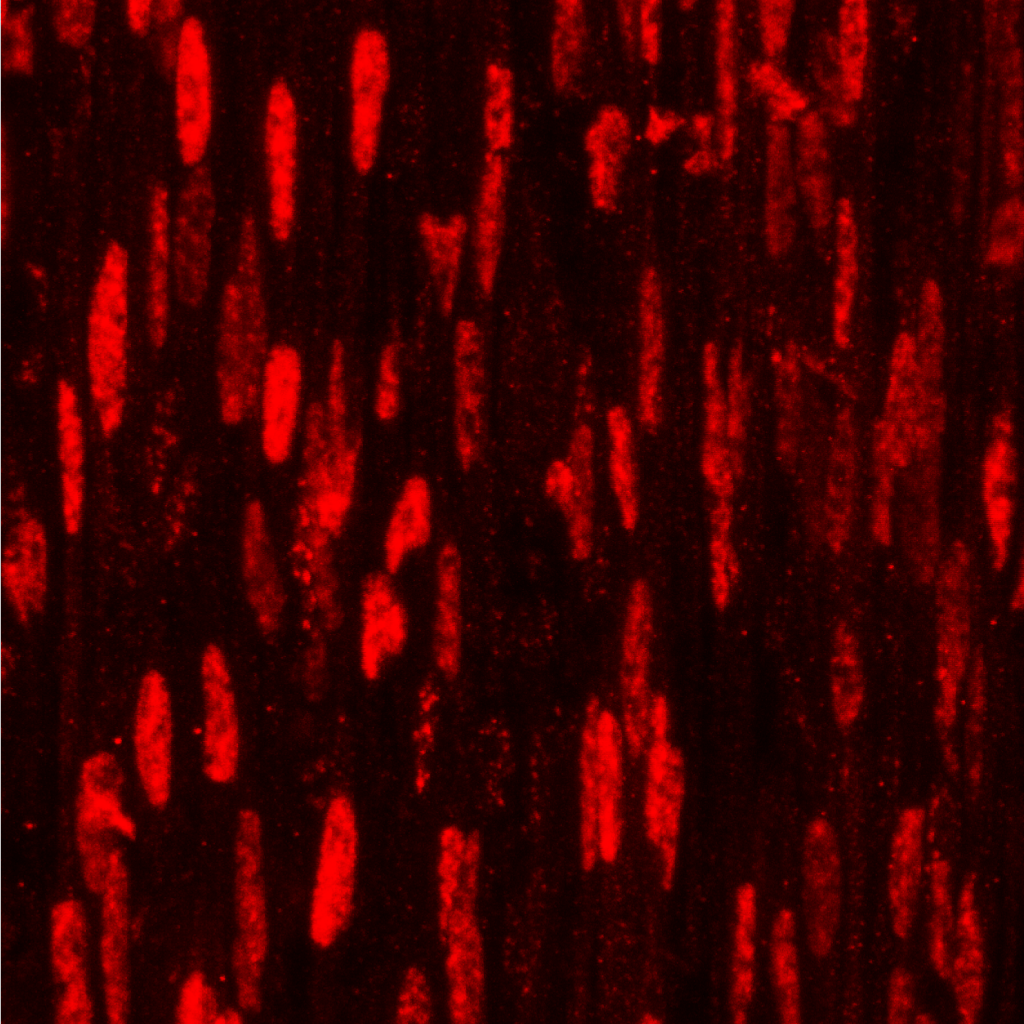

Supplement: Figure 9—source data 1. — This zip archive contains the IHC for one WT and one iDKO used for quantitative analysis shown in Figure 9E. Leica SP8 confocal lif images were processed using Imaris software and saved as tiffs. [file elife-50138-fig9-data1.zip › Figure 9 source data 1/WT #559 Krox20/Series 5 Sox10.tif]
